# Brain volume trajectories in Down syndrome and autosomal dominant Alzheimer's disease

**DOI:** 10.1002/alz.71103

**Published:** 2026-01-18

**Authors:** James T. Kennedy, Julie K. Wisch, Anna H. Boerwinkle, Peter R. Millar, Nicole S. McKay, Adam M. Brickman, Jasmeer P. Chhatwal, Patricio Chrem Mendez, Bradley T. Christian, Anne Cohen, Carlos Cruchaga, Alisha Daniels, Shaney Flores, Benjamin L. Handen, Sigan L. Hartley, Elizabeth Head, Laura Ibanez, Sharon J. Krisnsky‐McHale, Christian la Fougere, Florence Lai, Charles M. Laymon, Joseph H. Lee, Jae‐Hong Lee, Johannes Levin, Jorge Llibre‐Guerra, David F. Aguillon, Ira T. Lott, Mark Mapstone, Eric McDade, John Morris, Sid E. O'Bryant, Julie C. Price, Michael S. Rafii, Jee Hoon Roh, H. Diana Rosas, Nicole Schupf, Charlene Supnet‐Bell, Chengjie Xiong, Shahid Zaman, Tammie L. S. Benzinger, Brian A. Gordon, Beau M. Ances, the Alzheimer's Brain Consortium – Down Syndrome, and the Dominantly Inherited Alzheimer's Network

**Affiliations:** ^1^ Department of Neurology Washington University School of Medicine in St. Louis St. Louis Missouri USA; ^2^ McGovern Medical School University of Texas in Houston Houston Texas USA; ^3^ Department of Radiology Washington University School of Medicine in St. Louis St. Louis Missouri USA; ^4^ Department of Neurology Columbia University New York New York USA; ^5^ Gertrude H. Sergievsky Center and Taub Institute for Research on Alzheimer's Disease and the Aging Brain Columbia University New York New York USA; ^6^ Department of Neurology Harvard Medical School Massachusetts General Hospital Charlestown Massachusetts USA; ^7^ Centro de Memoria y Envejecimiento Buenos Aires Argentina; ^8^ Waisman Center University of Wisconsin‐Madison Madison Wisconsin USA; ^9^ Department of Medical Physics University of Wisconsin‐Madison Madison Wisconsin USA; ^10^ Department of Psychiatry University of Pittsburgh Pittsburgh Pennsylvania USA; ^11^ Department of Psychiatry Washington University in St Louis St Louis Missouri USA; ^12^ Hope Center for Neurological Disorders Washington University in St Louis St Louis Missouri USA; ^13^ Department of Human Development & Family Studies University of Wisconsin‐Madison Madison Wisconsin USA; ^14^ Department of Pathology and Laboratory Medicine University of California Irvine California USA; ^15^ Department of Neurobiology and Behavior University of California Irvine California USA; ^16^ Department of Psychology New York State Institute for Basic Research in Developmental Disabilities New York New York USA; ^17^ Department of Nuclear Medicine and Molecular Imaging Eberhard Karls University Hospital Tübingen Germany; ^18^ Department of Radiology University of Pittsburgh Pittsburgh Pennsylvania USA; ^19^ Department of Epidemiology Columbia University Irving Medical Center New York New York USA; ^20^ Department of Neurology University of Ulsan College of Medicine Asian Medical Center Seoul South Korea; ^21^ Department of Neurology LMU University Hospital LMU Munich Munich Germany; ^22^ German Center for Neurodegenerative Diseases, site Munich Munich Germany; ^23^ Munich Cluster for Systems Neurology Munich Germany; ^24^ Grupo de Neurociencias de Antioquia Facultad de Medicina Universidad de Antioquia Medellín Colombia; ^25^ Department of Pediatrics University of California Irvine School of Medicine Irvine California USA; ^26^ Department of Neurology University of California Irvine School of Medicine Irvine California USA; ^27^ Institute for Translational Research Department of Pharmacology and Neuroscience University of North Texas Health Science Center Fort Worth Texas USA; ^28^ Department of Radiology Harvard Medical School Massachusetts General Hospital Charlestown Massachusetts USA; ^29^ Alzheimer's Therapeutic Research Institute Keck School of Medicine of USC Los Angeles California USA; ^30^ Departments of Physiology and Neurology Korea University College of Medicine Seoul South Korea; ^31^ Department of Radiology Harvard Medical School Massasschusetts General Hospital Charlestown Massachusetts USA; ^32^ Department of Biostatistics Washington University in St Louis St Louis Missouri USA; ^33^ Cambridge Intellectual and Developmental Disabilities Research Group University of Cambridge Cambridge UK; ^34^ Department of Psychological & Brain Sciences Washington University in St. Louis St. Louis Missouri USA

**Keywords:** amyloid, MRI, volume

## Abstract

**INTRODUCTION:**

It is unknown if neurodegeneration trajectories differ between Down syndrome (DS) and autosomal dominant Alzheimer's disease (ADAD), both of which are genetic forms of Alzheimer's disease (AD).

**METHODS:**

We compared brain volumes in DS, ADAD, and unaffected family members serving as controls. Participants underwent magnetic resonance imaging (MRI) and amyloid positron emission tomography (PET), deriving volumetric and amyloid burden, respectively. Nonlinear associations between regional volumes and estimated years to clinical symptom onset (EYO) were evaluated using generalized additive mixed‐models.

**RESULTS:**

Longitudinal data from 267 controls, 341 participants with DS, and 358 participants with ADAD were included, totaling 1908 scans. DS volumes were lower than ADAD and controls initially and dropped linearly. ADAD had similar volumes to controls until diverging, beginning at EYO ‐7. Amyloid was negatively associated with volume, with similar slopes in DS and ADAD.

**DISCUSSION:**

ADAD and DS demonstrate distinct patterns of brain volume decline prior to symptom onset despite being similarly affected by amyloid.

## Highlights

- Volume declines before Alzheimer's pathology in Down syndrome.
- Volume declines shortly before symptom onset in autosomal dominant Alzheimer's.
- Down syndrome and autosomal dominant Alzheimer's are similarly affected by amyloid.
- Estimated years to onset of decline outpredicts volume relative to Centiloid.

## 1 BACKGROUND

Alzheimer's disease (AD) is the leading cause of dementia worldwide, affecting more than 55 million people and costing more than $1.3 trillion per year.^1^ AD is marked by the presence of amyloid plaques and tau tangles, followed by neurodegeneration and cognitive impairment.^2^ Rare genetic forms of AD account for ∼1%–2% of AD prevalence with almost 100% penetrance, leading to cognitive impairment at younger ages than typically seen for late onset AD.^3^, ^4^, ^5^ Genetic forms of AD include Down syndrome AD (DSAD) and autosomal dominant AD (ADAD). In individuals with DS, triplication of chromosome 21 results in an additional copy of the amyloid precursor protein (APP) gene responsible for overproduction of amyloid‐β,^3^, ^4^ leading to cognitive impairment developing in their early 50s.^3^ Similarly, individuals possessing ADAD‐specific mutations, which occur in the APP, presenilin 1 (PSEN1), or presenilin 2 (PSEN2) genes, also exhibit excess amyloid. This leads to dementia at a younger, highly predictable age,^5^ that can be predicted based on mutation type and parental age of onset.^6^ Unlike ADAD, where affected individuals are similar to the unaffected until disease onset, people with DS have a distinct phenotype that includes accelerated aging related to early cell senescence.^7^ Given that DSAD and ADAD are associated with highly reliable age of symptom onsets specific to their underlying mutation, pathological changes in both forms of AD can be staged as a function of estimated years to onset (EYO) of clinical symptoms.

Neuroimaging studies have previously compared DS and ADAD using amyloid^8^, ^9^, ^10^ and tau positron emission tomography (PET).^11^, ^12^ Overall, both groups have a similar sequence of pathological events with initial amyloid deposition, followed by tau accumulation, then cognitive impairment. However, the timing of these events differs between ADAD and DS.^3^, ^8^ Amyloid accumulates earlier relative to cognitive impairment in ADAD compared to DS, though the rate of change in amyloid is greater for DS than ADAD. By the onset of dementia, amyloid levels are similar for both groups.^8^ Tau has been shown to accumulate soon after amyloid onset and to be higher for a given amount of amyloid in DS compared to ADAD.^11^, ^12^

In general, brain volumes decrease with increasing AD pathology,^13^ serving as a biomarker of neurodegeneration, and predicting future cognitive impairment.^14^ The temporal and spatial changes in volumetric with regards to EYO have not been well studied in DS; subsequently, its usefulness as a biomarker remains uncertain. Changes in brain volumes in DS are complicated by developmental differences. Brain structure is different from birth in DS^15^ with smaller intracranial volumes,^16^ thicker cortices due to reduced gyrification,^17^ smaller hippocampi, and larger putamen observed.^18^ Brain volumes continue to decline with aging,^19^, ^20^, ^21^, ^22^, ^23^ though a recent paper found inflections in medial temporal volumes between the ages of 38 and 45 years old.^24^ In contrast, brain structure for ADAD is similar to healthy controls until later in the disease course.^25^

**RESEARCH IN CONTEXT**

1. **Systematic review**: The authors reviewed the literature using PubMed. Previous research has explored autosomal dominant Alzheimer's disease (ADAD) volume changes, typically using a single inflection changepoint model in cross‐sectional analyses. Down syndrome (DS) volume has also been investigated, typically as a linear model or in a limited set of regions of interest (ROIs). Differences in volume changes in both groups have not been examined.
2. **Interpretation**: Our findings point to independent neurodegenerative patterns in DS and ADAD despite both groups being similarly affected by amyloid. DS volumes are lower initially than controls and decrease prior to Alzheimer's disease (AD) pathology. Volumetric decline does not accelerate following amyloid accumulation. ADAD volumes are similar to controls until rapidly decreasing, beginning up to 7 years prior to symptom onset.
3. **Future directions**: Future direction include applying this approach to other neurodegenerative biomarkers (e.g., plasma neurofilament light chain [NfL], cortical thickness). AD drug trials may use these normative volume estimates to evaluate the effects of anti‐amyloid therapies on brain volumes.

Due to their predictable symptom onset, ADAD and DS can help identify early AD‐related changes in a reliable manner over a shorter period of time. This differs from sporadic AD, where individuals may have decades of pathology before the onset of symptoms.^2^ Understanding the expected trajectory of volumetric change over AD progression may be particularly important as new anti‐amyloid therapies may exacerbate volume loss.^26^ Insights into the timing and regional pattern of brain volumetric changes due to AD is necessary to evaluate the impact of new treatments in these two genetic forms of AD. The goal of these analyses is to identify the timing and location of brain volume changes in ADAD and DS, both before and after the onset of cognitive impairment, and to determine whether these changes are similar or different in these conditions.

## 2 METHODS

### 2.1 Participants

Participants were from two ongoing multisite longitudinal studies: the Alzheimer Biomarker Consortium—Down Syndrome (ABC‐DS) study and the Dominantly Inherited Alzheimer Network (DIAN). ABC‐DS enrolls participants with DS and sibling controls 25 years and older. DIAN recruits ADAD mutation carriers and familial controls who are at least 18 years old. See Supplemental Methods for how genetic status was determined.

### 2.2 Standard protocol approvals, registrations, and patient consents

Written informed consent or assent was obtained from all participants, or if individuals are incapable of providing consent, from their legally authorized representatives. All protocols were approved by the institutional review boards at the ABC‐DS and DIAN sites and conform to the ethical standards of the 1964 Declaration of Helsinki and its later amendments.

### 2.3 Genetics

All DIAN participants underwent genetic testing for an ADAD‐causing mutation in the APP, PSEN1, or PSEN2 genes. ABC‐DS participants had their medical records checked for karyotype or underwent genetic testing to confirm DS. Apolipoprotein E (APOE) ε4 status was established using a TaqMan genotyping assay (Applied Biosystems [Waltham, MA, USA]) for DIAN participants and a KASP assay (LGC Genomics [Beverly, MA, USA]) for ABC‐DS. The presence of at least one APOE ε4 allele was deemed as APOE ε4 positive.

### 2.4 EYO determination

For the ADAD group, EYO was calculated individually based on mutation‐specific age of onset and actual age of onset of dementia.^6^ For ABC‐DS the estimated age of onset was defined as 52.5 years old based on previous literature.^3^, ^8^ By using EYO we are able to directly compare DS and ADAD. EYO for familial controls was identical to their affected relatives. Our approach to EYO was consistent with previous analyses comparing ADAD and DSAD.^8^, ^11^, ^27^, ^28^, ^29^

### 2.5 Imaging

All participants had a T1‐weighted scan performed on a 3T magnetic resonance imaging (MRI) scanner. Scans were processed using FreeSurfer^30^ v5.3 and v7.3 for a subset of the ABC‐DS participants (*N* = 143; a comparison of versions is included as Table S1). Brain volumes were summed across hemispheres for each individual region, generating 34 unique cortical and 10 unique subcortical gray matter regions. Regional volumes were corrected for intracranial volume using a linear mixed effect‐based residual approach^31^ and converted to a *z*‐score to simplify comparisons across regions using two approaches: normalizing using all participants so that change across regions is on the same scale while preserving group differences or normalizing groups separately using the amyloid negative members of each cohort. The first approach allows for comparison of the actual volume of each group while the second removes the developmental offset observed in DS. All cohorts had the same processing and quality control to allow for direct comparison between groups.

### 2.6 Amyloid PET imaging

The majority of participants underwent amyloid PET scans using [11C]‐Pittsburgh compound B (PiB) for DIAN and PiB or [18F]‐AV45 (florbetapir) for ABC‐DS. PET data from 50–70 min after bolus injection was used for ABC‐DS for both tracers and 40‐70 min for DIAN. PET scans were processed using a PET Unified Pipeline^32^ that aligns the PET scan with the FreeSurfer segmentations and applies partial volume correction using a regional spread function.^33^ Regional standardized uptake value ratios (SUVRs) were calculated using the cerebellum as the reference region. To harmonize across PET tracers, Centiloid values^34^ were calculated from the SUVRs based on a cortical summary measure.^32^ All PET processing was identical for ABC‐DS and DIAN data. Amyloid positive (Centiloid ≥ 16.4)^34^ controls were omitted from analyses.

### 2.7 Clinical assessment

All participants were evaluated for dementia. In ABC‐DS, this was accomplished by a committee with experience in evaluating dementia in DS.^35^ The committee used neuropsychological evaluations designed for people with DS, medical and psychiatric histories, and interviews with caregivers or informants to derive a consensus diagnosis of cognitively unimpaired, mild cognitive impairment (MCI), or dementia due to AD. For some participants with DS, a consensus was not reached, and they were designated as uncertain. Cognitive status in DIAN participants was established using the Clinical Dementia Rating (CDR) scale, which rates participants as either cognitively unimpaired (0), very mild dementia (0.5), mild dementia (1), moderate dementia (2), or severe dementia (3). Cognitive status in both groups was binarized, either cognitively unimpaired or showing signs of cognitive impairment (MCI or dementia) for ABC‐DS or CDR > 0 for DIAN. While there is some heterogeneity in clinical presentation in MCI,^36^ the early onset, high penetrance, and symptom homogeneity of AD in DS and ADAD meant any cognitive impairment was interpreted as indicative of AD.

### 2.8 Statistics

All analyses were completed in R v4.41.^37^ Group differences in demographics were analyzed using chi‐squared tests for categorical variables and independent sample t‐tests for continuous variables. Results are reported as uncorrected for multiple comparisons for demographics only. The effects of amyloid accumulation and impairment on regional brain volumes were calculated in linear mixed effect (LME) models, comparing volumes of controls against cognitively unimpaired amyloid negative (CU‐), cognitively unimpaired amyloid positive (CU+), and cognitively impaired amyloid positive (IMP+) participants with DS or ADAD mutations. All models controlled for the effects of sex, APOE e4 status, and FreeSurfer version and treated individual nested under family as random effects. Group difference effects were considered significant if they survived a false discovery rate (FDR) corrected *p* ≤0.05 applied to a Tukey correction for number of group comparisons in each analysis (21).

We also compared brain volumes trajectories between groups through generalized additive mixed‐models (GAMMs) in the R mgcv package.^38^ GAMMs do not assume a consistent relationship between two measures, instead allowing the direction and shape (linear or nonlinear) of the relationship to vary over segments of the slope, or splines. GAMMs find the model that accounts for the most variance while penalizing for model complexity to avoid overfitting. Slope shape was summarized with the estimated degrees of freedom (EDF), where 1 indicates a linear relationship and values greater than 1 indicate increasingly nonlinear relationships. The shape of a relationship can significantly differ between groups that have linear relationships due to differences in slope steepness.

Group‐specific relationships between brain volume and EYO were evaluated using GAMMs. GAMMs allow for slopes to vary by group and included sex, APOE e4 status, and FreeSurfer version as covariates, and treated individual nested under family as a random effect. Differences between DS, ADAD, and controls were examined with regards to main effect differences in brain volumes between groups (offset), differences in the shape of the relationship with EYO between groups, and the EYO ranges where groups significantly diverged. A group‐specific amyloid negative normalization was performed to allow for divergence analyses for DS as significant developmental effects are present.^16^, ^17^, ^18^ Differences in group offset and shape were extracted from the model summary. EYO‐specific group divergences were calculated by examining when the estimate for one group no longer fell within the spline‐based confidence interval for another and vice‐versa. Correction for multiple comparisons was performed by applying an FDR correction for tests of group main effects and interactive effects with EYO and a Bonferroni correction for ranges of group divergences. Multiple comparison corrections were applied separately for the analyses using group‐specific normalized data. Analyses were repeated using amyloid in Centiloids,^34^ excluding controls due to limited range.

To examine whether brain volumes were better predicted by EYO or Centiloid, analyses compared the Akaike Information Criterion (AIC) for volumes predicted by EYO or by Centiloid. AIC is a measure of model fit, where lower values indicate better model performance. As AICs depend on sample size and number of parameters, they cannot be compared across groups. These analyses examined groups separately, using only participants with both an EYO and Centiloid. As the EYO and Centiloid models have the same degrees of freedom, region‐specific comparisons in an analysis of variance (ANOVA) were not feasible. Instead, a paired t‐test was performed on the AICs of the EYO and Centiloid models.

Analyses were conducted to test for group‐specific inflection points in the linear relationship between intracranial volume‐corrected volume and EYO. A group‐specific piecewise LME model was calculated, allowing for a single inflection point for each region, and compared against an inflectionless model. Inflection methods and results are detailed in the supplementary materials.

Volumetric results are described at a summary level (e.g., cortical or subcortical) in the main text. When demonstrating group slopes we used three regions implicated in the pathogenesis of genetic AD pathology: the hippocampus, precuneus, and nucleus accumbens.^3^, ^39^, ^40^, ^41^, ^42^ A more detailed regional description of trajectory differences by group can be found in the supplemental results. Videos of volume estimates and group differences between EYOs ‐20 to 10 and Centiloids 0 to 150 can be found in the supplement, with and without correcting for developmental offset (Supplemental Videos 1–6). Individual region volume‐EYO/Centiloid plots can also be found in the supplemental materials (Supplemental Plots 1‐8).

Analyses examining gene‐specific effects in ADAD are detailed in the supplement.

## 3 RESULTS

### 3.1 Participants

Participants consisted of 267 controls combined over both cohorts, 341 adults with DS, and 358 individuals with an ADAD mutation, with 576, 472, and 860 longitudinal MRIs, respectively, for a total of 1908 scans. There were 106 unique mutations in the ADAD sample. Participants with DS (mean age[SD] = 42.8[9.4] at baseline) were significantly older than ADAD (37.4[10.8]; *p <* 0.001) and controls (37.6[11.2]; *p <* 0.001). Age was not included as a covariate in the model to control for this group difference due to its high collinearity with EYO, especially for DS. EYO was only significantly different between ADAD (−8.7[9.4]) and controls (−11.2[11.5]; DS ‐9.7[9.4]). A higher proportion of DS participants were male (55.1%) compared to controls (39.0%; *p <* 0.001) or ADAD (43.3%; *p = *0.002). Participants with ADAD were significantly more likely to be impaired relative to DS (33.2% vs 19.1%, *p <* 0.001). The presence of an APOE e4 allele did not significantly differ between groups. Amyloid PET (in Centiloids) was significantly different between all groups at baseline—ADAD (40.7[48.8]), DS (28.0[37.3]), and controls (−0.7[3.4]), all *p's *< 0.001. However, for those individuals with cognitive impairment, Centiloid did not differ between ADAD and DS (81.3[54.5] and 79.6[41.4], respectively; *p = *0.85). See Table 1 for a description of groups by sex, mutation type, APOE e4, age, EYO, and Centiloid. See Supplemental Results and Table S2 for ADAD gene‐specific demographics.

**TABLE 1:** Demographics.

Parameter	Baseline			All		
	Control	DS	ADAD	Control	DS	ADAD
N (male)	267 (104)	341 (188)^*^	358 (155)	576 (231)	472 (258)	860 (373)
No. Impaired	–	65	119^*^	–	100	294
No. of amyloid scans	246	238	314	493	318	688
APOE E4 prevalence	28%	23%	28%	29%	24%	30%
Age (SD)	37.6 (11.2)	42.8 (9.4)^*^	37.4 (10.8)	39.0 (10.3)	43.8 (9.4)	39.4 (10.8)
EYO (SD)	−11.2 (11.5)	−9.7 (9.4)	−8.7 (11.4)^*^	−9.5 (10.4)	−8.7 (9.4)	−7.0 (10.7)
Centiloid (SD)	−0.7 (3.4)	28.0 (37.3)^*^	40.7 (48.8)^*^	−1.0 (3.4)	30.3 (38.8)	44.7 (50.7)
Impaired Centiloid (SD)	–	79.6 (41.4)	81.3 (55.4)	–	79.6 (43.7)	85.0 (54.5)
N Tri/Mos/Trns/Unk	–	308/12/14/7	–	–	428/16/19/9	–
N APP/PSEN1/PSEN2	–	–	65/266/27	–	–	191/603/66

*Note*: Characteristics of each genetic Alzheimer group and controls.

* : Group was significantly different from another for this metric, see Results for more information.

Abbreviations: ADAD, participants with an autosomal dominant mutation; APP, amyloid precursor protein; APOE, apolipoprotein E; DS, participants with Down syndrome; EYO, estimated years until onset of cognitive impairment; Mos, mosaicism; PSEN, presenilin; SD, standard deviation; Tri, trisomy 21; Trns, translocation; Unk, unknown.

### 3.2 Effects of amyloid and cognitive impairment at the group level

**FIGURE 1:**
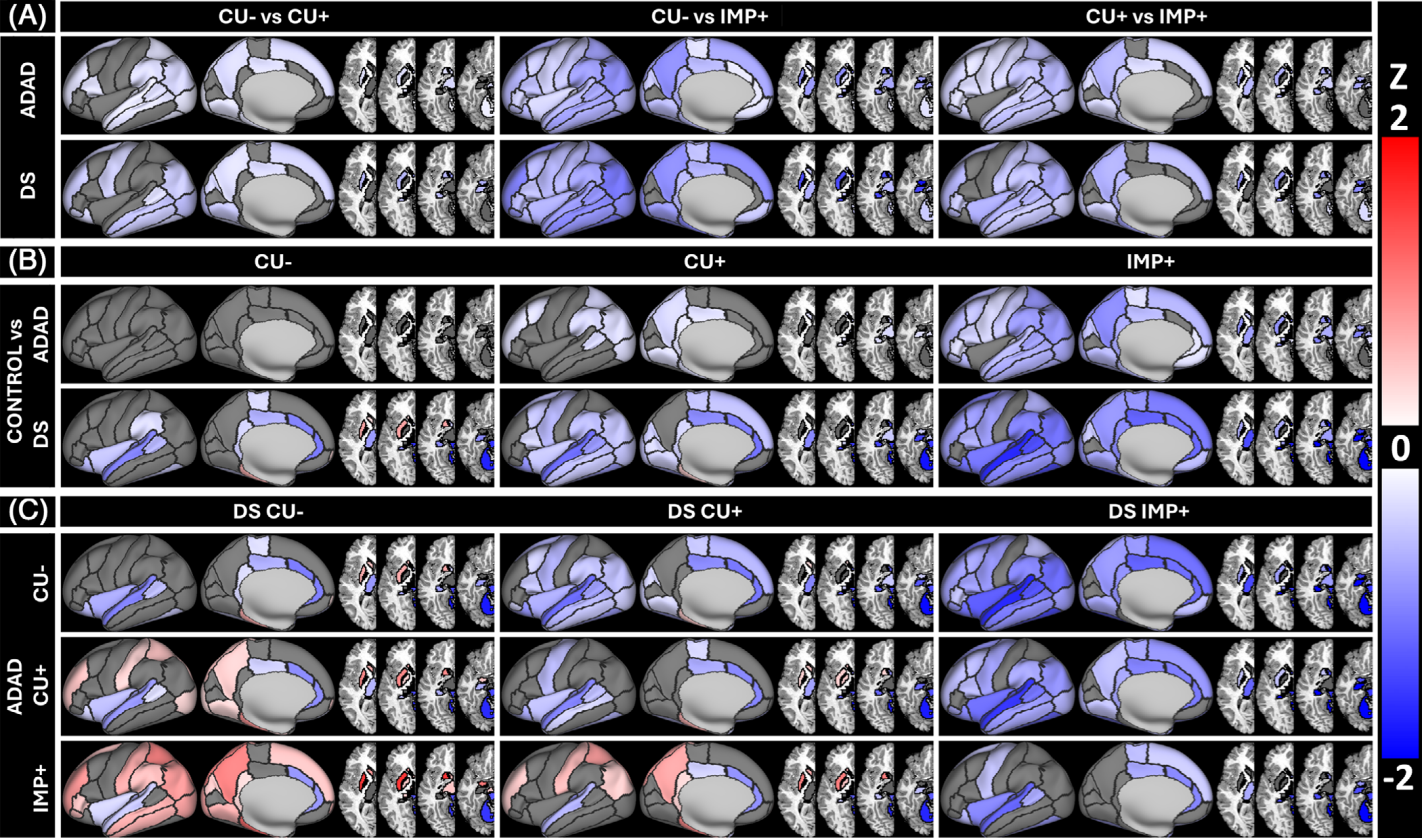
Group‐specific volume differences. Volumes presented as differences in *z*‐scores calculated across all groups after an intracranial volume‐correction. *z*‐scores thresholded at ± 2. Groups were divided based on stage of AD pathology. (A) Comparisons between controls and all groups. (B) Within‐group ADAD comparisons. (C) Within‐group DS comparisons. (D) ADAD and DS comparisons. Red indicates the first group listed was significantly smaller than the second, blue indicates the second group was significantly smaller than the first. Gray regions not significantly different. All significant values survived an FDR for the number of regions analyzed (44) applied to a Tukey corrected group difference for number of comparisons (21). AD, Alzheimer's disease; ADAD, autosomal‐dominant Alzheimer's disease; CU−, cognitively unimpaired amyloid negative; CU+, cognitively unimpaired amyloid positive; DS, Down syndrome; FDR, false discovery rate; IMP+, cognitively impaired amyloid positive

LME analyses of the influence of amyloid and cognitive impairment on brain volume found that escalating AD pathology was associated with significantly smaller brain volumes in most regions. Amyloid accumulation in ADAD was associated with smaller volumes in primarily striatal subcortical, and inferior temporal, parietal, and occipital cortical regions. Progression to cognitive impairment in ADAD resulted in smaller brain volumes in almost all regions. In DS, amyloid accumulation was associated with smaller volumes in frontal, inferior/medial parietotemporal, and subcortical regions. Cognitive impairment was linked to greater brain volume loss in these regions, which was more significant than the loss caused by amyloid accumulation in ADAD. Between group analyses found that brain volumes in controls were similar to CU‐ ADAD carriers while CU‐ DS were typically smaller than either group. Figure 1 shows significant between and within group (control/ADAD/DS) and stage (CU‐/CU+/IMP+) differences in volume across all group and stage comparisons. Prior to amyloid accumulation, volumes were similar for ADAD and controls but were already lower for DS. Both DS and ADAD volumes decrease with progressive AD stage. Sex effects were seen for most regions for all groups, with smaller volumes observed for females relative to males. APOE e4 did not have a significant effect on brain volumes. See Supplemental Table 3 for the full LME results.

### 3.3 GAMM Analyses

#### 3.3.1 Volume loss with increasing EYO

**FIGURE 2:**
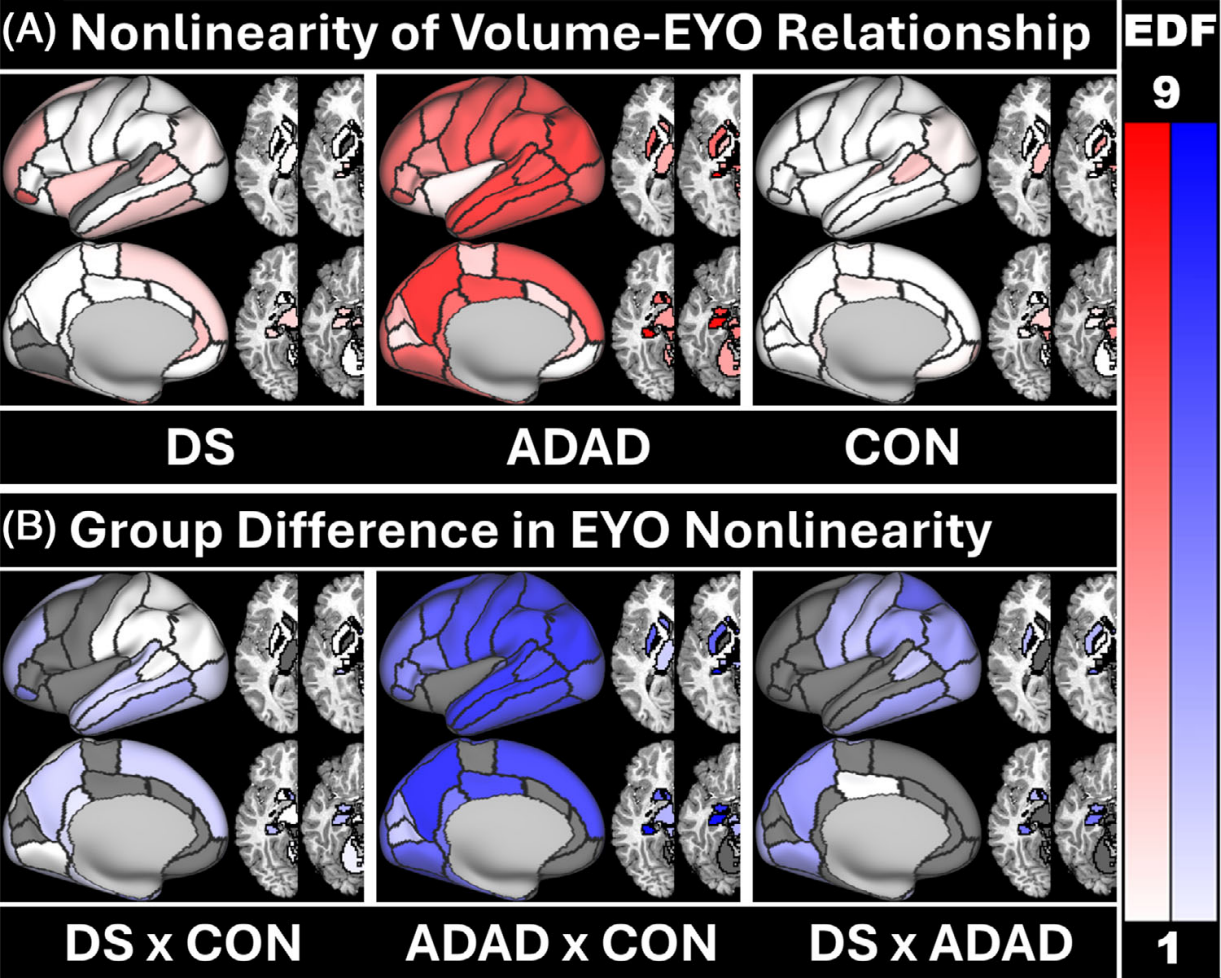
Nonlinearity of the relationship between volume and EYO. Nonlinearity, measured by EDF, of the relationship between volume and EYO (A) for DS, ADAD, and sibling CON and (B) group differences in linearity for the volume relationship with regards to EYO. An EDF = 1 (white regions) represents a linear relationship while an EDF > 1 indicated a nonlinear relationship (red/blue regions). A max EDF of 9 was chosen to capture the full range of EDFs reported (1 to 8.42). Darker colors indicate a more nonlinear relationship, gray indicating no significant relationship with EYO (A) or no shape difference between groups (B). (B) A white region indicates that, while both groups have the same slope complexity, one group's slope is significantly steeper than the other. ADAD, autosomal dominant Alzheimer disease; CON, controls; DS, Down syndrome; EDF, estimated degrees of freedom; EYO, estimated years to onset of cognitive impairment

Volumes decreased with increasing EYO for all groups. Parametric effects resemble those described in the LME analyses. Slope trajectory and group differences in slope are demonstrated in Figure 2. Figure 2A shows that the EYO‐volume relationship is linear (EDF ∼1) for both DS and controls but is nonlinear for ADAD (EDF > 1) in most regions, rapidly declining around EYO ‐7. This was consistent with inflection point analyses which observed significant inflections in almost all brain regions in ADAD (see Table S4). An inflection was seen in relatively few regions for DS but was observed for a few regions relevant to AD including the amygdala and hippocampus. For these two regions inflections were seen at EYO −18 to −19, respectively, with negative slopes seen before the inflection. Figure 2B shows that slope shape is significantly different between ADAD compared to DS or controls (white‐blue regions indicating significant differences in slope between groups, darker colors indicating greater differences in slope complexity). While DS and controls had linear slopes, negative slopes were steeper in DS (light colors indicating steepness differences in similar slopes) in subcortical, parietal, and temporal regions implicated in AD pathology.^40^, ^41^, ^42^ Full statistics can be found in Table S5.

**FIGURE 3:**
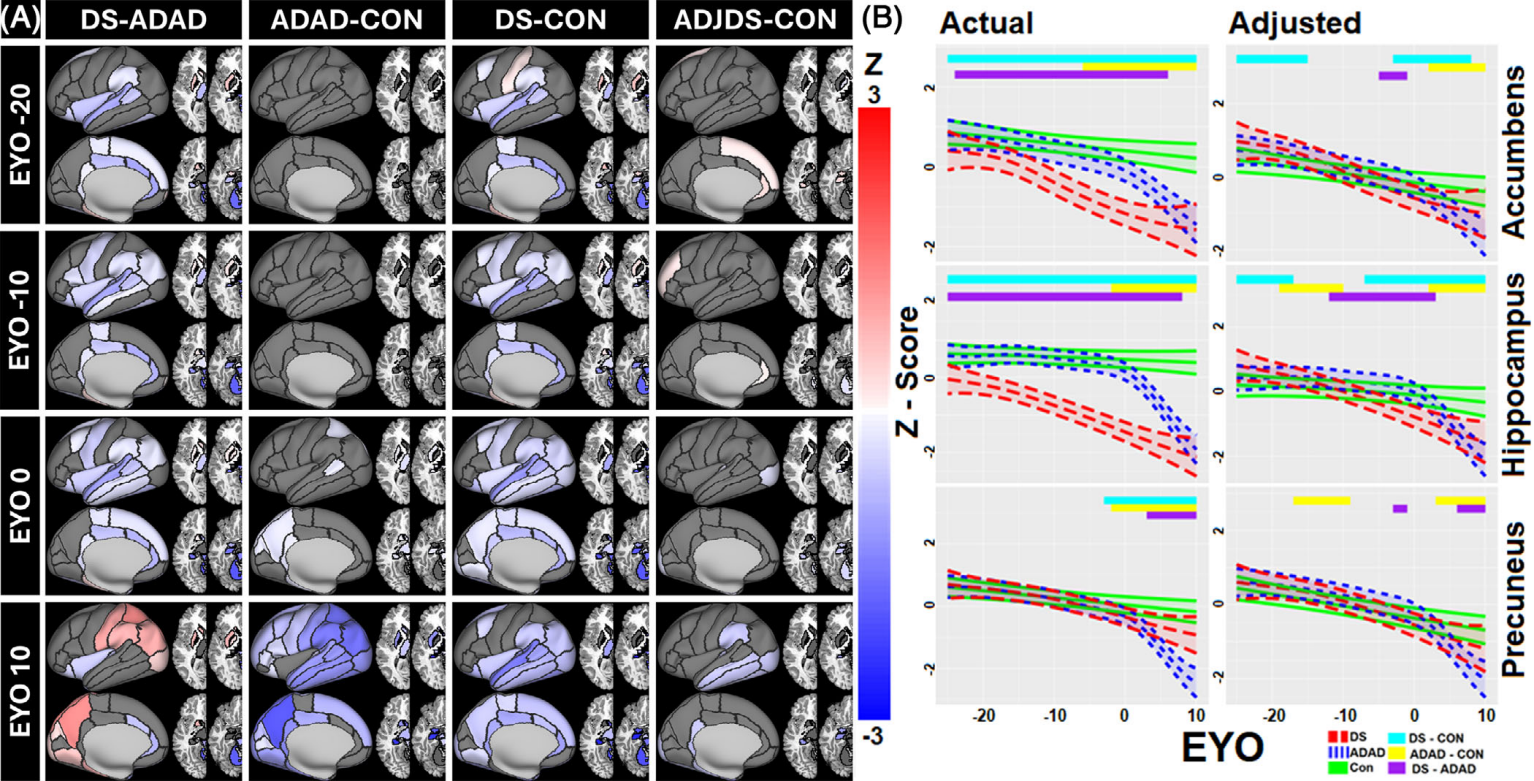
Group differences in volume over time. (A) Z value of time‐specific significant group differences in regional volume at different EYOs from ‐20 to +10. Red indicates the first group listed has a significantly higher volume than the second, blue the opposite. Darker colors indicate a greater difference. Gray indicates no significant difference between groups at a particular time interval. ADJDS‐CON compares volumes between DS and controls after adjusting for developmental offset in DS. Plots show early volume deficits in DS expanding over time while ADAD volume does not begin to diverge until near EYO 0, affecting most of the brain by EYO +10 and with smaller volumes than in DS after EYO 0. Adjusting for developmental differences limits DS volume deficits to primarily temporal and subcortical regions. (B) Standardized volumetric change over time (with regards to EYO) for certain regions of interest in each group with ranges of significant group divergences noted at the top of the plots. EYO‐specific estimate patterns show the relatively linear DS slopes and the inflected ADAD trajectories. Actual: *z*‐score calculated across all participants, Adjusted: *z*‐scores adjusted to account for the developmental effect in DS. ADAD, autosomal dominant mutation carriers; CON, controls; DS, Down syndrome; EYO, estimated years until onset of cognitive impairment

Group differences in volumes at 10‐year intervals in EYO are shown in Figure 3A. DS volumes were typically smaller than controls initially, with the number of regions and the difference between group volumes increasing over time. Adjusting for developmental effects removed most differences between DS and controls. Significantly smaller volumes in DS (outside of the cerebellum) appeared only at EYO 0 in subcortical and occipital regions, spreading temporally and parietally by EYO +10. Volume differences between ADAD and controls were not observed at EYO ‐10 but were apparent at EYO 0 and widespread by EYO +10. By EYO +10 ADAD volumes were generally smaller than DS despite ADAD volumes declining later. Figure 3B shows estimated volume slopes in ROIs for each group, before and after accounting for developmental differences with DS. Due to the linear decline present before amyloid accumulation, normalizing the DS cohort to the amyloid negative subset leads to the youngest participants being shifted above the mean, while the oldest participants fall below the line. The results for ADAD based on specific genetic mutations can be found in the Supplemental Results and Table S6. The slopes in regions of interest for specific ADAD mutations can be visualized in Figure S1. Videos showing volume estimates and group divergences for DS, ADAD, and controls between EYOs ‐20 to +10, with and without adjusting for developmental effects, can be found in Supplemental Videos 1–4 (unadjusted group estimates SV1, unadjusted group differences SV2, adjusted group estimates SV3, and adjusted group differences SV4). Plots showing the volume‐EYO relationship for each region can be found in Supplemental Files 1–4.

#### 3.3.2 Amyloid models and fit relative to EYO

**FIGURE 4:**
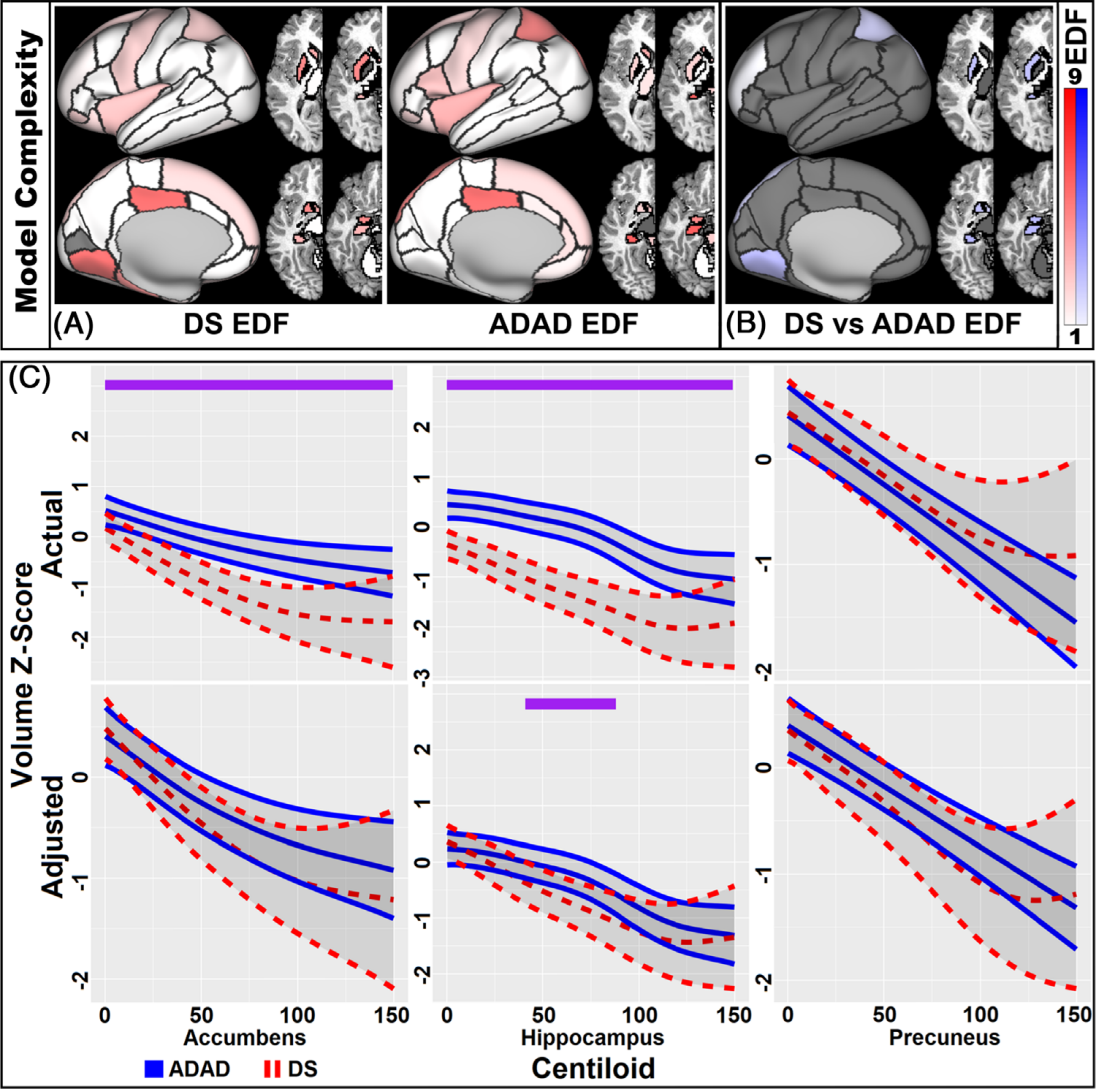
Relationship between Centiloid and volume. (A) Nonlinearity, measured by EDF, of the relationship between volume and Centiloid for DS and ADAD and (B) group differences in linearity for the volume–Centiloid relationship. EDF of 1 = linear, > 1 more nonlinear. Darker colors indicate a more nonlinear relationship, gray indicating no significant relationship with Centiloid (A) or no shape difference between groups (B). Volume–Centiloid slopes are typically linear and similar in both groups, outside subcortical regions. (C) Volumetric estimates across Centiloids in regions of interest in DS (red) and ADAD (blue), ranges of significant group divergences are noted in purple. Plots show similar relationship between volume and Centiloid in both groups, though DS is more affected in the hippocampus between Centiloids of 40 and 90. Actual: volume *z*‐score after normalizing across all participants. Adjusted: volume *z*‐score adjusted to account for developmental offset in DS. ADAD, autosomal dominant mutation carriers; EDF, estimated degrees of freedom; DS, Down syndrome

Analyses examining the relationship between Centiloid and volume found similar negative relationships in DS and ADAD in almost all brain regions. Relationships were nonlinear in subcortical and frontoparietal regions, including key regions like the hippocampus. Slope differences were mainly seen in subcortical regions, where DS showed greater volume loss than ADAD for the same Centiloid levels. The similarity in slopes can be visualized in Figure 4 for DS and ADAD and Figure S1 for DS, APP, PSEN1, and PSEN2. Full GAMM model results can be found in Supplemental Table 7 while results from models that allow slopes to vary according to affected ADAD gene can be found in Table S8, and inflection points for group‐specific relationships between Centiloid and volume can be found in Table S9. Videos showing the estimated volumes and group differences between Centiloids 0 and 150, with and without controlling for developmental differences, can be found in Supplemental Videos 5–6 (unadjusted SV5, adjusted SV6) and region‐specific volume–Centiloid plots found in Supplemental Files 5–8.

**FIGURE 5:**
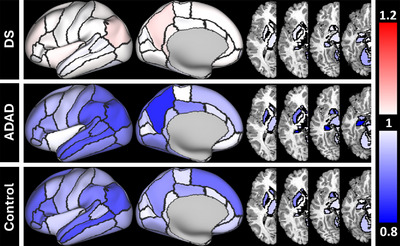
Ratio of EYO to Centiloid AIC. The model fit by a group and region‐specific EYO GAMM model divided by the fit of the Centiloid model. Lower values (blue) indicate the EYO model accounted for more variance than Centiloid, higher values (red) indicate Centiloid accounted for more variance than EYO. White indicates both models performed similarly. AIC, Akaike Information Criterion; EYO, estimated years to onset; GAMM, generalized additive mixed‐models

Comparing model fits, EYO better explained brain volume changes in ADAD and controls than Centiloid levels (paired t‐tests of EYO and Centiloid AICs: *p* < 0.001 for controls and ADAD, *p* = 0.321 for DS). Parietal and hippocampal regions strongly favored an EYO model in ADAD, and parietal regions slightly favored a Centiloid model in DS. Detailed results for the model fit comparisons can be visualized in Figure 5 and found in Table S10.

## 4 DISCUSSION

Our analyses found an inverse relationship between EYO and amyloid with brain volume. DS typically exhibited smaller brain volumes than controls or ADAD. EYO‐volume relationships were nonlinear in ADAD and linear in DS and controls. Volumetric declines were more pronounced in DS compared to controls, especially in posterior and subcortical regions. ADAD volumes followed a similar trajectory to controls until decreasing significantly about 7 years before cognitive impairment onset. Differences in EYO‐volume were earliest in subcortical regions for both groups. In ADAD, cortical divergence appeared first in parietal regions, while in DS, it began in the lateral occipital regions and spread to temporal areas near impairment onset. Volume‐Centiloid slopes were generally linear and similar in DS and ADAD, but associations occurred at lower Centiloid levels in DS. Model comparisons indicated that EYO is a better predictor of volume changes than amyloid in ADAD.

Non‐linear relationships were observed between volume and EYO in ADAD. Divergences began in regions associated with ADAD (e.g., precuneus, hippocampus)^25^, ^41^ and expanded to most of the brain by an EYO +10 (Figure 3). Changes were seen within areas typically spared in LOAD (e.g., occipital),^41^ with only the insula and medial prefrontal unaffected. Since volume loss occurred shortly before symptom onset, tracking these changes may provide a marker of incipient impairment in ADAD. A particular area of interest is the striatum, where volume first diverged and which has been implicated as an area of early AD pathology in ADAD and DSAD.^40^ This is in contrast to more cortical regions like the precuneus that have been implicated in LOAD.^3^, ^43^, ^44^ Our divergence timings differ from previous studies, which reported divergence more than a decade before impairment. This discrepancy may reflect differences in the modality (thickness) and EYO approach.^25^, ^39^, ^45^ Our changepoint analyses place hippocampal inflections at the same EYO as previous research (see supplement).^46^

The EYO–volume relationship was primarily linear for DS and controls, with developmental effects typically resulting in smaller volumes in DS. Decline was faster in DS in subcortical, parietal, and temporal regions compared to controls^41^ and preceded AD pathology.^8^, ^11^ This suggests volume loss independent of AD, as slopes largely did not change with amyloid conversion.^8^ Inflections were observed in a few subcortical regions, consistent with previous work.^24^ We detected inflections in DS in the hippocampus and amygdala around the time of amyloid accumulation, though slopes pre‐inflection were still negative. This suggests that, while the onset of AD pathology may be associated with accelerated volume loss in key regions, loss is already occurring in DS.

Interestingly, precuneus volumes did not conform to this overall pattern. Instead, volumetric decline in the precuneus of individuals with DS more closely resembled the pattern seen in ADAD. There were no early differences in precuneus volume between controls and individuals with DS. Individuals with DS exhibited a nonlinear decline in precuneus volume, initially matching controls and ADAD before diverging from controls at EYO ‐3, 1 year before the ADAD‐control divergence. Although these patterns were similar, the post‐divergence slope was greater in ADAD. Previous research in DS has identified precuneus thickness as an excellent classifier of AD stage.^42^ Other work has identified precuneus atrophy as an early marker of neurodegeneration in both ADAD^39^ and APOE ε44 carriers.^47^ Our findings in conjunction with existing evidence suggest that the precuneus may display unique vulnerability to genetically‐associated AD.

The non‐AD volume loss observed in DS may reflect two factors: accelerated aging and DYRK1A overexpression. Brain‐cell derived epigenetic age has been shown to be 11.5 years older than chronological age in DS.^48^ MRI‐based estimates of brain age found a 5.3‐year brain–age gap, indicating accelerated aging in DS detectable using neuroimaging.^49^ DYRK1A is a gene on the 21^st^ chromosome involved in neurogenesis and astrocyte reactivity.^50^ DYRK1A overexpression disrupts both factors, resulting in malformed dendrites and axons, reduced synaptogenesis, and impaired excitotoxic glutamate clearance by astrocytes. The linear decline observed in most brain regions may reflect accelerated aging or excitotoxic cell death, while the inflection point decline reported in certain regions may represent additional effects of AD. Why these effects are observed in several temporal‐parietal regions affected in AD is unclear.

The association between amyloid and volume was similar for both DS and ADAD. The largely linear and negative relationship observed in both groups suggest atrophy correlates with increasing disease severity. Although this association was present, EYO accounted for more variance in volumes in ADAD but not DS. The reasons for this are likely two‐fold: (1) as the rate of change for Centiloid is largely consistent once amyloid accumulation begins^8^ and volume is continuously decreasing and typically unaffected by amyloid in DS, then Centiloid is effectively another temporal marker in DS and, like EYO, exhibits a linear association with volume. (2) In contrast, in ADAD, EYO is a relatively precise temporal marker of disease stage, as it is anchored to the predictable age of symptom onset within families which, along with ADAD time course,^51^ can vary significantly by mutation.^5^ Because EYO in ADAD closely reflects proximity to symptom onset, it aligns more tightly with the period of rapid volumetric loss. This can be visualized in Figures 2 and 5, where the regions that have the greatest volume loss are also the regions where the difference in variance accounted for by the EYO and Centiloid models was greatest. The intrasubject variability is poorly accounted for by Centiloid in ADAD, resulting in weaker models and less accurate slopes where the inflection has been smoothed out. Thus, while both groups show similar patterns of amyloid‐related atrophy, differences in disease pacing and the precision of EYO as a staging metric likely explain the different EYO–volume association observed between DS and ADAD. It is worth noting that in DSAD an individualized measure of AD progression accounted for as much variance as a measure based on a groupwide impairment estimate subtracted from age.

Our current results align with our recent fluorodeoxyglucose (FDG) ‐PET research conducted on a subset of participants from ABC‐DS and DIAN.^27^ FDG‐PET, which measures glucose metabolism and serves as a proxy for neurodegeneration, showed a nonlinear relationship with EYO for ADAD, diverging from controls around EYO ‐7. DS FDG‐PET started lower than ADAD and controls and declined linearly. The observed decline for DS was more rapid than controls. For both DS and ADAD, FDG‐PET declined with increasing Centiloid. Overall, both FDG‐PET and MRI results in DS indicate a linear decline in neurodegeneration markers that precedes amyloid and tau changes, suggesting these changes are largely unaffected by AD pathology. These neurodegenerative changes may be linked to developmental alterations in key brain structures due to chromosomal triplication, starting at birth and progressing with age.^52^

Understanding regional volume trajectories in genetic forms of AD is important as new AD treatments that target amyloid and slow the course of the disease have also been shown to cause volume loss.^26^ Clinicians will need to be familiar with typical patterns of change due to aging compared to anti‐amyloid therapy. Providing regional volume‐EYO trajectories for both ADAD and DS gives group‐specific templates to compare volumetric change against when examining treatment efficacy and potential side effects. Several clinical trials have been performed in ADAD, with an upcoming study administering anti‐amyloid therapies before participants become amyloid positive.^53^ Only recently have anti‐amyloid therapies been considered in DS.^54^

### 4.1 Limitations and future directions

There are some limitations with this study. The use of different amyloid tracers in ABC‐DS meant that we had to use a summary Centiloid measure and could not examine the effect of regional amyloid on volume. Centiloid was based on regions involved in late onset AD^32^ rather than regions affected in DSAD or ADAD. The prevalence of cognitive decline was significantly lower in DS compared to ADAD. There is heterogeneity in DS dementia onset^55^ that may influence volume trajectories. However, our analyses and previous pathological studies have shown that amyloid and tau are stereotypically seen by age 40. The acquisition of more longitudinal amyloid and cognitive data will allow us to estimate individual conversion points using techniques like amyloid chronicity.^56^ Future directions include expanding to other imaging modalities (e.g., cortical thickness) and other biomarker modalities (e.g., plasma/cerebrospinal fluid [CSF]).

## 5 CONCLUSIONS

Brain volume was inversely related to EYO and PET amyloid in most regions for both DS and ADAD. In ADAD, volume changes were similar to controls until sharply decreasing around EYO ‐7. DS volumes showed a more linear decline, suggesting AD‐independent volume loss. Despite differing temporal patterns, the amyloid‐volume relationship was similar for both DS and ADAD. Initially, DS participants had lower brain volumes than controls, with both groups experiencing linear declines; however, DS showed faster declines in posterior and subcortical regions. Conversely, ADAD exhibited inflection points where volume declined faster than DS once it began. Divergence from controls occurred earliest in subcortical regions for both groups, while cortical effects appeared in ADAD before cognitive impairment onset, mainly in the parietal lobe, and were most prominent in DS in the temporal lobe after onset. These findings may shed light on aging effects compared to new anti‐amyloid therapies related to volumetric declines.

## CONFLICTS OF INTEREST STATEMENT

The authors report no conflicts.

## CONSENT STATEMENT

All participants and their legally authorized caregivers provided informed assent or consent.

## Supporting information



Supporting information

Supporting information

Supporting information

Supporting information

Supporting information

Supporting information

Supporting information

Supporting information

Supporting information

Supporting information

Supporting information

Supporting information

Supporting information

Supporting information

Supporting information

Supporting information

Supporting information

Supporting information

## APPENDIX 1

### 1.1

ABC‐DS and DIAN collaborators are indexed online at https://www.nia.nih.gov/research/abc‐ds#sites and https://dian.wustl.edu/our‐research/observational‐study/dian‐observational‐study‐sites/
