# Supplementary material for: Brain volume trajectories in Down syndrome and autosomal dominant Alzheimer's disease
Source: Alzheimers Dement. 2026 Jan 18;22(1):e71103. doi: 10.1002/alz.71103 (PMC12812856; doi:10.1002/alz.71103)
Supplement: Supplementary file 2 — Supporting information [file ALZ-22-e71103-s009.docx]

**Supplemental Methods**

**FreeSurfer Version Comparison**

A subset of the ABC-DS participants was processed in FreeSurfer v7.3 rather than 5.3. The effect of FreeSurfer version was tested by comparing three DS only GAMM models - 1) where volume was predicted by nonlinear EYO, sex, and APOE4 status, 2) where a group effect of FreeSurfer version was added to model 1, and 3) where the nonlinear EYO effect in model 2 was allowed to vary between groups. These three models were compared for each region in ANOVAs, L-Ratios significant after a false discovery rate multiple comparison correction of corrected p < 0.05 were considered significant.

**Inflection Point Analyses**

Analyses were conducted to test for group-specific inflection points in the linear relationship between corrected volume and EYO/centiloid. A piecewise linear mixed effect model was calculated that allowed for a single inflection point for each region. This was done separately for each group using the segmented R package.^1^ As these analyses were already group specific, analyses using the amyloid-negative group based normalized data that allowed comparison across groups were not performed. The piecewise model was compared against a linear model with no inflection point. Note that this package cannot detect more than one inflection point in mixed effect models. Sex, APOE ε4 status, and FreeSurfer version were included as covariates. Family was not included as a random effect as nested mixed models were not viable. Regions in which the model with the inflection point had a significantly better fit than the model without one were considered significant after an FDR correction was applied within group across the number of regions analyzed. Group specific slopes were extracted from the model without an inflection as were pre and post inflection slopes from the model with an inflection. Paired t-tests of noninflected, pre-inflection, and post-inflection EYO-volume beta values were performed to compare change rates.

**Supplemental Results**

**Amyloid and Volume Relationships were Similar in ADAD and DS**

An increase in centiloids was associated with a reduction in brain volume for both DS and ADAD participants. The relationship between centiloids and brain volume tended to be linear for most brain regions for both groups. Nonlinear relationships in both DS and ADAD were observed in frontoparietal and subcortical regions. Slope differences were evaluated for subcortical regions associated with AD (e.g. hippocampus) or cortical regions affected late in the disease process (e.g. rostral middle frontal) with DS having a steeper slope compared to ADAD. After accounting for developmental volume differences in DS, divergences in the volume-centiloid slopes between ADAD and DS were observed primarily in temporal-occipital cortical regions and subcortically in the amygdala and hippocampus, with divergences typically beginning around a centiloid of 50 and with volumes lower in DS. Slope differences and divergence estimates suggest that amyloid induced volume loss was greater for DS compared to ADAD for temporal regions of interest. This is consistent with previous work finding greater tau pathology at the same amount of amyloid in DS compared to ADAD.^2^ The linearity of the relationship between volume and centiloid and differences are plotted in Figure 2A and B. Estimates in key regions, some demonstrating slope differences (DS steeper than ADAD), are presented in Figure 2C. See Supplemental Table 7 for the full GAMM PET centiloid results, and the supplemental materials for region specific volume-centiloid plots for all regions.

**EYO Outperforms Amyloid When Estimating Volume in ADAD**

While centiloid and EYO are related (*R^2^* of 0.47 in DS and 0.41 in ADAD using baseline data), centiloid reflects levels of AD pathology often early in the disease course while EYO reflects time to the onset of cognitive impairment and reflects changes later in the disease course.^3^ Comparison of model fits suggest that EYO outperforms centiloid when predicting brain volume for ADAD and controls but not DS (paired t-tests of EYO and centiloid AICs control and ADAD *p*’s < 0.001, DS *p* = 0.321). Centiloid models had a higher AIC than comparable EYO models except in DS, in which some cortical regions of interest did have slightly better fits in the centiloid model (e.g. inferior parietal, precuneus, see Supplemental Table 10). These results suggest that when examining the time course of volume loss in ADAD, research using amyloid PET may be less informative than a family history, consistent with previous research finding highly variable amyloid trajectories across mutations despite similar outcomes.^4^ Similar performance of subject specific amyloid PET and subject nonspecific EYO were observed in DS. It may be the case that the timing and rate of progression of AD pathology is highly consistent across individuals with DS, since individualized measures perform similarly to group-based estimates. That EYO outperformed centiloid in controls is unsurprising given that in an amyloid-negative cohort, centiloid is effectively meaningless, while EYO reflects changes seen with aging.

**FreeSurfer Version Comparison**

While significant group differences were observed between FreeSurfer versions, the direction of effect was inconsistent, making harmonization between versions using methods like ComBat impractical. The model that best accounted for variance included FreeSurfer version as a group effect in most regions. As the difference between versions may be safely accounted for by a parametric version offset, one was included in the main analyses. See the full results of the model comparison in Supplemental Table 1.

**Inflection Model Results**

The presence and timing of inflections differed strongly between groups and regions. A non-inflected model better described the EYO-volume relationship in DS and controls in most regions. Inflections were detected in a few key regions in DS, namely the amygdala (-19) and hippocampus (-18). Comparison of beta values pre and post inflection in these regions show volumes were declining pre-inflection and the decline accelerating post (see Supplemental Table 3). An inflection model better fit the ADAD data in all but 1 (non-converging in the medial orbitofrontal cortex), with the timing of the inflection point again varying by region (e.g. precuneus at EYO of -3 and nucleus accumbens at 1) but typically within a few years before or after EYO 0.

The slope of brain volume over disease progression differed between groups. The linear decline observed in the EYO-volume slopes in DS was steeper than the decline in controls (p < 0.001). The pre-inflection ADAD slopes were not significantly different from familial controls (p = 0.459) while post-inflection ADAD models were significantly steeper than the non-inflected DS models (p < 0.001; see Supplemental Table 4). These results again suggest that the EYO-volume relationship in DS is typically linear (outside of a few regions of interest) and that brain volumes in DS decrease consistently and with a steeper slope than in normal aging. ADAD slopes were similar to controls pre-inflection but steeper than both controls and DS post-inflection.

Inflection point analyses examining the relationship between centiloid and volume found few regions with significant inflections (see Supplemental Table 9). Significant inflection points were observed in DS in the subcortical and temporal regions (volume dropping quickly before plateauing at 73 centiloids) and precuneus (dropping and plateauing or rebounding at ~85 centiloids). ADAD inflections were observed in frontal and striatal regions, dropping until ~40 centiloids for striatal regions or 140 for frontal before rebounding.

**Gene Specific Analyses**

**Participants by Gene Group**

ADAD participants consisted of 65 people with an APP mutation, 266 people with a PSEN1 mutation, and 27 with a PSEN2 mutation, with a total number of 191, 603, and 66 scans, respectively. Demographic comparisons of groups at baseline found several significant differences. Participants with a PSEN1 mutation were significantly less likely to be male relative to participants with DS (p = 0.001) and people with a PSEN2 mutation were significantly more likely to be male than controls (p = 0.027). Participants with DS were significantly less likely than participants with APP (p = 0.007) or PSEN1 (p < 0.001) mutations to be impaired. Participants with PSEN2 mutations were significantly less likely than participants with PSEN1 mutations to be impaired at baseline (p = 0.018). Participants with DS were significantly older than participants with APP (p = 0.02), PSEN1 (p < 0.001), or PSEN2 (p = 0.004) mutations. Participants with PSEN2 mutations had lower EYOs than controls (p = 0.026), participants with DS (p = 0.005), and participants with APP (p = 0.003) or PSEN1 (p = 0.001) mutations. Controls also had lower EYOs than participants with PSEN1 mutations (p = 0.001). Centiloids were significantly lower in controls relative to all other groups (all ps ≤ 0.001) and were significantly higher in participants with PSEN1 mutations relative to participants with DS (p < 0.001) or participants with an APP mutation (p = 0.03). When limited to participants with AD-related cognitive decline, participants with PSEN2 mutations had higher centiloids than all other groups (p < 0.001). There was no significant difference in APOE4 prevalence among groups. See Supplemental Table 2 for the full demographics.

**GAMM Analyses**

[Insert Supplemental Figure 1]

**Parametric Effects for Gene Analyses**

Parametric differences in group offset were observed when comparing mutations in specific genes to DS or controls but not to one another. Understanding the significance of the effect of affected gene on offset is stymied by the wide range of sample sizes. Significant lower volumes in PSEN1 mutation carriers relative to controls were limited to posterior, primarily parieto-occipital and subcortical (amygdala, brainstem, hippocampus, accumbens, thalamus, and ventral diencephalon) regions, lower volume in controls was observed in the insula. PSEN2 mutation carriers had significantly smaller parietal, superior temporal, and nucleus accumbens volumes relative to controls. There were no significant differences in offset between APP mutation carriers and controls. Comparison of effect sizes rather than significance suggests that many regions of interest (e.g. precuneus, hippocampus, isthmus of the cingulate) were similarly affected in APP and PSEN1 mutation carriers, but a significant effect was not observed. The effect estimates in regions where a significant effect was observed in PSEN2 mutation carriers often double the offset effect observed in PSEN1, suggesting that PSEN2 pathology may be more severe than PSEN1. Significant group effects were observed between DS and each gene group, with mutation carriers generally having larger regional volumes than DS in a similar pattern to when all ADAD gene groups were combined. The number of significant regions in the DS vs gene group comparisons diminished with the sample size of the comparison group. See Supplemental Table 6 for the full parametric results.

**Linearity of the Relationship between Volume and EYO by Gene Group**

Significant associations between volume and EYO were observed in each gene group in almost all brain regions. A volume-EYO relationship was not observed in PSEN2 mutation carriers in multiple, primarily frontal regions, but also in the entorhinal cortex where AD pathology is typically observed. PSEN1 slopes were more nonlinear than APP or PSEN2 (paired t-test ps < 0.001) while PSEN2 were more linear than APP (p = 0.033). This may just reflect the sample sizes as a sparser sample would need fewer points fitted and may result in smoother slopes. Examination of trajectory plots in ROIs show a relatively flat trajectory leading to a clear inflection point for APP and PSEN1 groups while PSEN2 trajectories appear more quadratic. All slope plots are provided as supplementary figures.

Significant group differences in trajectories were observed between PSEN1 and APP/PSEN2 in almost all regions but not between APP and PSEN2, with sample size differences again obfuscating the potential reasons. Relative to APP mutation carriers, the PSEN1 group trajectories in ROIs like the hippocampus and precuneus appear to begin to decline slightly earlier and plateau late in the disease process, though this plateauing may simply be due to overfitting to an outlier resulting from a lack of participants with high EYOs. PSEN2 trajectories appear to begin to decline even earlier in most ROIs (though not the hippocampus), though slopes generally aren’t as steep. EYO specific significant group divergences between controls and gene groups were largely limited to PSEN1 carriers and largely followed the timing of the combined ADAD group. This is unsurprising as the confidence intervals used to estimate divergence are heavily influenced by sample size and the PSEN1 group was far larger than the others. Significant APP divergences were observed primarily in ROIs but not until after EYO 0. Divergences for PSEN2 mutation carriers tended to also happen later, though a volume drop at EYO -4 was observed in the supramarginal and in the hippocampus at EYO 0, possibly reflecting an early cortical effect in this group. Regional linearity and group trajectory differences are available in Supplemental Table 6.

Inflection point analyses show significant EYO-volume inflections in almost all regions for all groups, though fewer significant inflections were observed for the small sample PSEN2 group. Estimated inflections in ROIs vary between groups in ways that both support and contradict the GAMM analyses (e.g. showing an earlier inflection in the hippocampus for PSEN1; -3.5; relative to APP; -0.9; but a later inflection in the precuneus; -2.5 for PSEN1 and -6.4 for APP; all inflection estimates lie outside the Bonferroni corrected 95% confidence interval of the other group). The full list of inflections can be found in Supplemental Table 9.

**Relationship between Volume and Centiloid by Gene Group**

Parametric effects of group mirrored the EYO analyses. Significant and predominantly linear relationships between volume and centiloid were observed for each gene group. Nonlinearity in one gene group was typically present in the others, with significant differences in trajectory limited to the inferior parietal for APP vs PSEN2 groups (PSEN2 declining later and faster) and for PSEN1 vs APP/PSEN2 in the insula (volume rebounding around centiloids of 100 in PSEN1 mutation carriers). Centiloid specific group effect analyses show the different gene groups never significantly diverge. Full centiloid results can be found in Supplemental Table 8.

**Gene Group Effects on the Comparison of EYO and Centiloid when Predicting Volume**

Analyses comparing the model fits of EYO and centiloid for each gene group found that EYO generally outperformed centiloid, though with some exceptions. Centiloid performed similarly to or outpredicted EYO in PSEN2 carriers in several ROIs, including the amygdala, precuneus, superior temporal, and supramarginal gyrus. Pairwise comparison of the ratio of EYO to centiloid AICs between groups found that this ratio was lowest (meaning EYO performed better than centiloid) in PSEN1 models (average ratio = 0.91) relative to APP (average ratio = 0.951, p < 0.001) and PSEN2 (average ratio = 0.982, p < 0.001) and APP ratios were significantly lower than PSEN2 (p = 0.002). The lowest ratio was observed in the PSEN1 group in the hippocampus (0.73), far lower than in APP or PSEN2 (both 0.98). These results suggest that there may be factors unique to each group and region that influence how tightly coupled amyloid burden is to volumetric change. See Supplemental Table 10 for regional results.

**Supplemental Discussion**

**Gene-Specific ADAD Differences**

Specific genetic mutation group comparisons within the ADAD cohort suggest similar trajectories across affected genes, but small APP and PSEN2 sample sizes hinder interpretation. Previous work examining gene and mutation effects on amyloid trajectories^4^ found significant differences in slopes that did not appear to influence cognitive impairment. It may be that volumetric change, as the last measure in the AT(N) framework before the onset of cognitive impairment, is more driven by tau than amyloid^3^. If volume reflects a relatively consistent final progression to dementia and amyloid a more variable trajectory, volume may serve as a more generalizable proxy for incipient cognitive impairment in ADAD relative to amyloid, where mutation type must be considered. Alternately, we may just be underpowered to identify group differences in volume.

**Supplemental References**

1. Muggeo VM, Atkins DC, Gallop RJ, Dimidjian S. Segmented mixed models with random changepoints: a maximum likelihood approach with application to treatment for depression study. *Statistical Modelling*. 2014;14(4):293-313. doi:10.1177/1471082X13504721

2. Wisch JK, McKay NS, Boerwinkle AH, et al. Comparison of tau spread in people with Down syndrome versus autosomal-dominant Alzheimer’s disease: a cross-sectional study. *Lancet Neurol*. 2024;23(5):500-510. doi:10.1016/S1474-4422(24)00084-X

3. Jack CR, Knopman DS, Jagust WJ, et al. Tracking pathophysiological processes in Alzheimer’s disease: an updated hypothetical model of dynamic biomarkers. *Lancet Neurol*. 2013;12(2):207-216. doi:10.1016/S1474-4422(12)70291-0

4. Chhatwal JP, Schultz SA, McDade E, et al. Variant-dependent heterogeneity in amyloid β burden in autosomal dominant Alzheimer’s disease: cross-sectional and longitudinal analyses of an observational study. *The Lancet Neurology*. 2022;21(2):140-152. doi:10.1016/S1474-4422(21)00375-6

**Supplemental Video Caption 1: Group and EYO specific volume estimates in Z scores.** Red indicates an above average volume at a specific EYO (shown in the bottom right), blue indicates below average volume. Gray regions were nonsignificant at that EYO. Darker colors indicate greater deviation from normal/divergence between groups. EYO: Estimated years until onset of cognitive impairment, DS: Down syndrome, ADAD: Autosomal dominant mutation carriers, CON: Controls.

**Supplemental Video Caption 2: EYO specific group differences between estimates in Z scores.** Red indicates the first group listed is greater than the second and the opposite for blue. Gray regions were nonsignificant at that EYO. Darker colors indicate greater divergence between groups. EYO: Estimated years until onset of cognitive impairment, DS: Down syndrome, ADAD: Autosomal dominant mutation carriers, CON: Controls.

**Supplemental Video Caption 3: Group and EYO specific volume estimates in Z scores after correcting for developmental effects.** Red indicates an above average volume at a specific EYO (shown in the bottom right), blue indicates below average volume. Gray regions were nonsignificant at that EYO. Darker colors indicate greater deviation from normal/divergence between groups. EYO: Estimated years until onset of cognitive impairment, DS: Down syndrome, ADAD: Autosomal dominant mutation carriers, CON: Controls.

**Supplemental Video Caption 4: EYO specific group differences between estimates in Z scores after correcting for developmental effects.** Red indicates the first group listed is greater than the second and the opposite for blue. Gray regions were nonsignificant at that EYO. Darker colors indicate greater divergence between groups. EYO: Estimated years until onset of cognitive impairment, DS: Down syndrome, ADAD: Autosomal dominant mutation carriers, CON: Controls.

**Supplemental Video Caption 5: Group and centiloid specific volume estimates (1st and 2nd columns) and group differences between estimates (3rd column) in Z scores.** In the first two columns red indicates an above average volume at a specific centiloid (shown in the bottom right), blue indicates below average volume. In the last column red indicates the DS group has greater volume than the ADAD group and the opposite for blue. Gray regions were nonsignificant at that centiloid. Lighter colors indicate greater deviation from normal/divergence between groups. DS: Down syndrome, ADAD: Autosomal dominant mutation carriers.

**Supplemental Video Caption 6: Group and centiloid specific volume estimates (1st and 2nd columns) and group differences between estimates (3rd column) in Z scores after correcting for developmental effects.** In the first two columns red indicates an above average volume at a specific centiloid (shown in the bottom right), blue indicates below average volume. In the last column red indicates the DS group has greater volume than the ADAD group and the opposite for blue. Gray regions were nonsignificant at that centiloid. Lighter colors indicate greater deviation from normal/divergence between groups. DS: Down syndrome, ADAD: Autosomal dominant mutation carriers.

**Supplemental File 1 – EYO.** Region specific plots demonstrating the relationship between volume and estimated years until symptom onset (EYO) in controls (Con, green), Down syndrome (DS, red), and autosomal dominant mutation carriers (ADAD, blue). Plots limited to EYOs -25 to 10. Cyan bars at the top of the plot indicate EYOs when volumes for DS and Con significantly differ, yellow indicates when ADAD and Con significantly differ, and purple when DS and ADAD significantly differ.

**Supplemental File 2 – EYO Adjusted.** Region specific plots demonstrating the relationship between volume and estimated years until symptom onset (EYO) in controls (Con, green), Down syndrome (DS, red), and autosomal dominant mutation carriers (ADAD, blue) after adjusting for developmental effects. Plots limited to EYOs -25 to 10. Cyan bars at the top of the plot indicate EYOs when volumes for DS and Con significantly differ, yellow indicates when ADAD and Con significantly differ, and purple when DS and ADAD significantly differ.

**Supplemental File 3 – EYO Gene.** Region specific plots demonstrating the relationship between volume and estimated years until symptom onset (EYO) in controls (Con, green), Down syndrome (DS, blue), APP mutation carriers (red), PSEN1 mutation carriers (PS1,cyan), and PSEN2 mutation carriers (PS2, yellow). Striped bars to the right of the line plot indicate the EYOs when groups significantly differed.

**Supplemental File 4 – EYO Gene Adjusted.** Region specific plots demonstrating the relationship between volume and estimated years until symptom onset (EYO) in controls (Con, green), Down syndrome (DS, blue), APP mutation carriers (red), PSEN1 mutation carriers (PS1, cyan), and PSEN2 mutation carriers (PS2, yellow) after adjusting for developmental effects. Striped bars to the right of the line plot indicate the EYOs when groups significantly differed.

**Supplemental File 5 – Centiloid.** Region specific plots demonstrating the relationship between volume and centiloid in Down syndrome (DS, red), and autosomal dominant mutation carriers (ADAD, blue). Plots limited to centiloids 0-150. Purple bars at the top of the plot indicate centiloids where volumes for DS and ADAD significantly differ.

**Supplemental File 6 – Centiloid Adjusted.** Region specific plots demonstrating the relationship between volume and centiloid in Down syndrome (DS, red), and autosomal dominant mutation carriers (ADAD, blue) after adjusting for developmental effects. Plots limited to centiloids 0-150. Purple bars at the top of the plot indicate centiloids where volumes for DS and ADAD significantly differ.

**Supplemental File 7 – Centiloid Gene.** Region specific plots demonstrating the relationship between volume and estimated years until symptom onset (EYO) in controls (Con, green), Down syndrome (DS, blue), APP mutation carriers (red), PSEN1 mutation carriers (PS1,cyan), and PSEN2 mutation carriers (PS2, yellow). Striped bars to the right of the line plot indicate the centiloids where groups significantly differed.

**Supplemental File 8 – Centiloid Adjusted.** Region specific plots demonstrating the relationship between volume and centiloid in Down syndrome (DS, blue), APP mutation carriers (red), PSEN1 mutation carriers (PS1, cyan), and PSEN2 mutation carriers (PS2, yellow) after adjusting for developmental effects. Striped bars to the right of the line plot indicate the centiloids where groups significantly differed.
