# Supplementary material for: Brain volume trajectories in Down syndrome and autosomal dominant Alzheimer's disease
Source: Alzheimers Dement. 2026 Jan 18;22(1):e71103. doi: 10.1002/alz.71103 (PMC12812856; doi:10.1002/alz.71103)
Supplement: Supplementary file 3 — Supporting information [file ALZ-22-e71103-s005.docx]

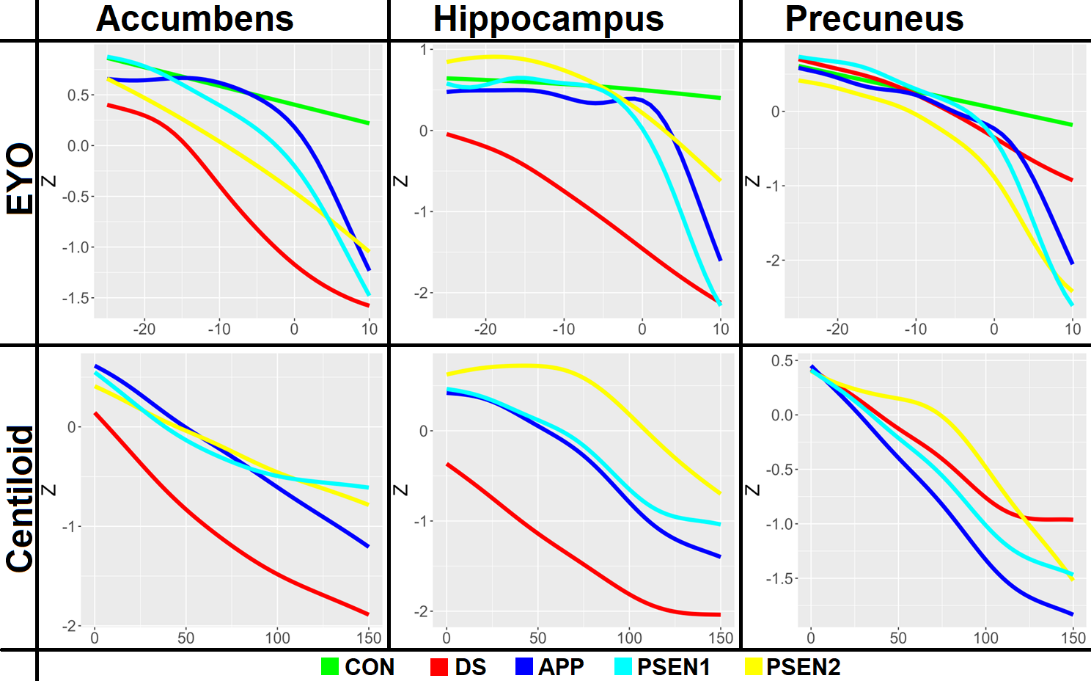


**Supplemental Figure 1: The effects of ADAD specific Gene and DS on the relationship between volume and EYO/centiloid.** Group specific volume estimates (Z-scored) in ROIs across EYOs and centiloids after splitting autosomal-dominant AD mutation carriers by affected gene. Gene specific slopes typically do not diverge from one another until after EYO 0 and not at all in centiloid analyses. Though appearing different, PSEN2 slopes are not significantly different, likely due to low power. CON: Controls, DS: Down syndrome, APP/PSEN1/PSEN2: Gene with early onset AD mutation, EYO: Estimated Years until Onset of cognitive impairment.
