# Supplementary material for: Brain volume trajectories in Down syndrome and autosomal dominant Alzheimer's disease
Source: Alzheimers Dement. 2026 Jan 18;22(1):e71103. doi: 10.1002/alz.71103 (PMC12812856; doi:10.1002/alz.71103)
Supplement: Supplementary file 4 — Supporting information [file ALZ-22-e71103-s012.pdf]

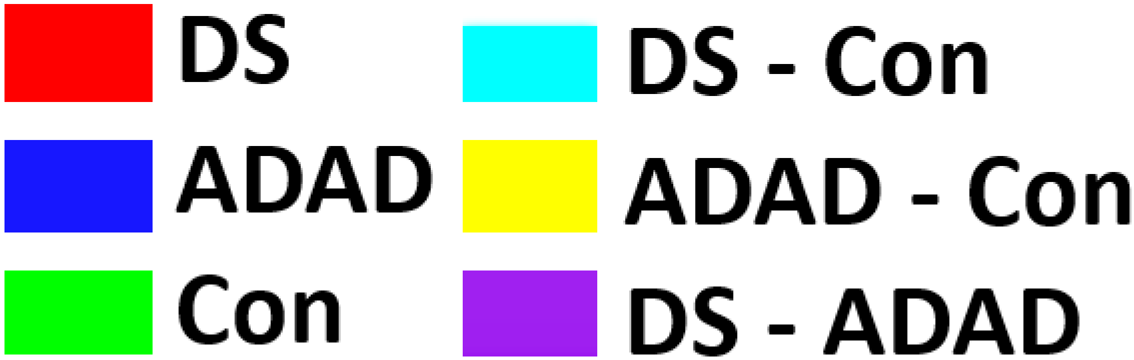

DS
ADAD
Con
DS - Con
ADAD - Con
DS - ADAD

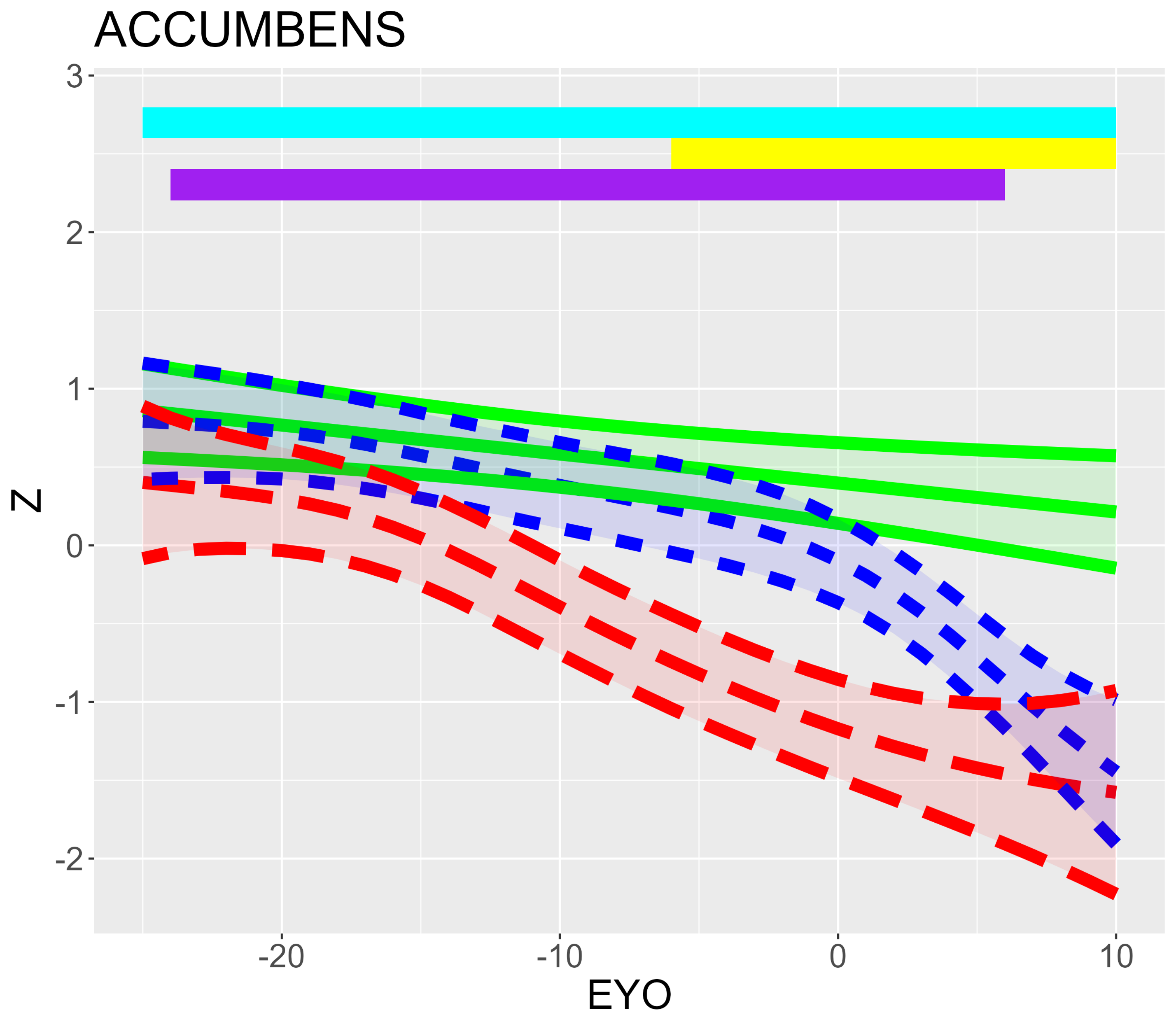

ACCUMBENS
3
2
1
0
-1
-2
Z
-20
-10
0
10
EYO

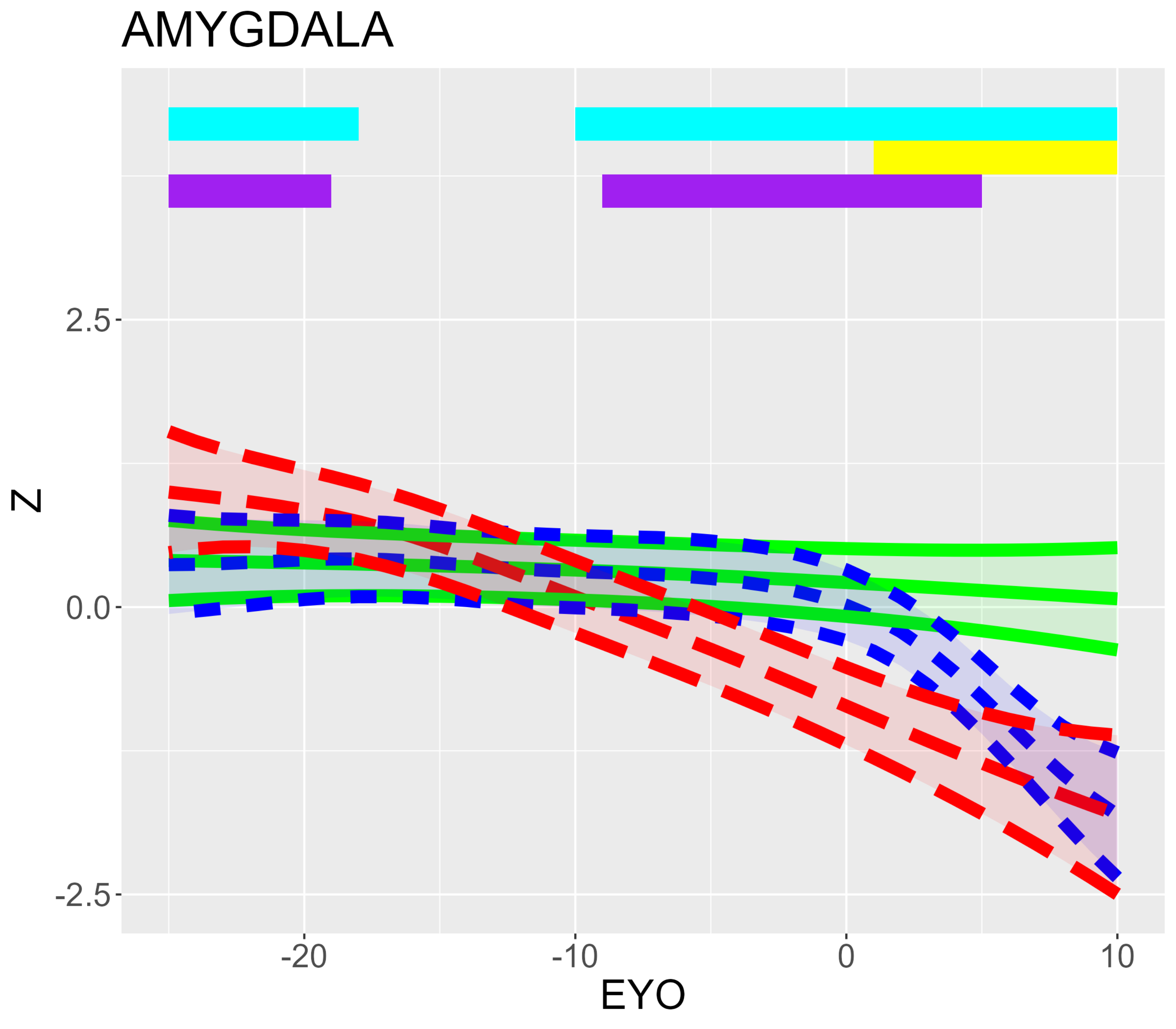

AMYGDALA
2.5
0.0
-2.5
Z
-20
-10
0
10
EYO

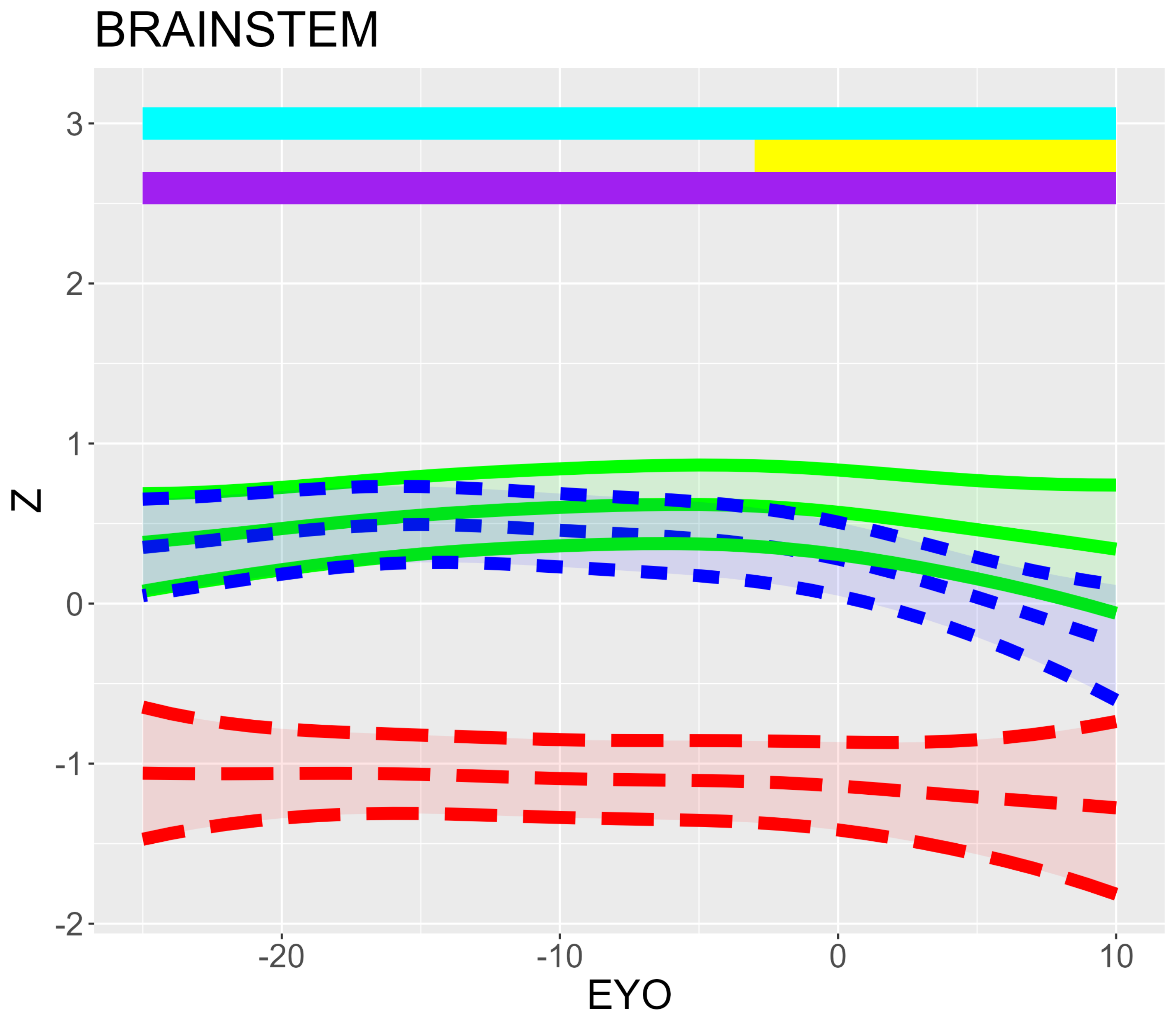
BRAINSTEM
3
2
1
0
-1
-2
Z
-20
-10
0
10
EYO

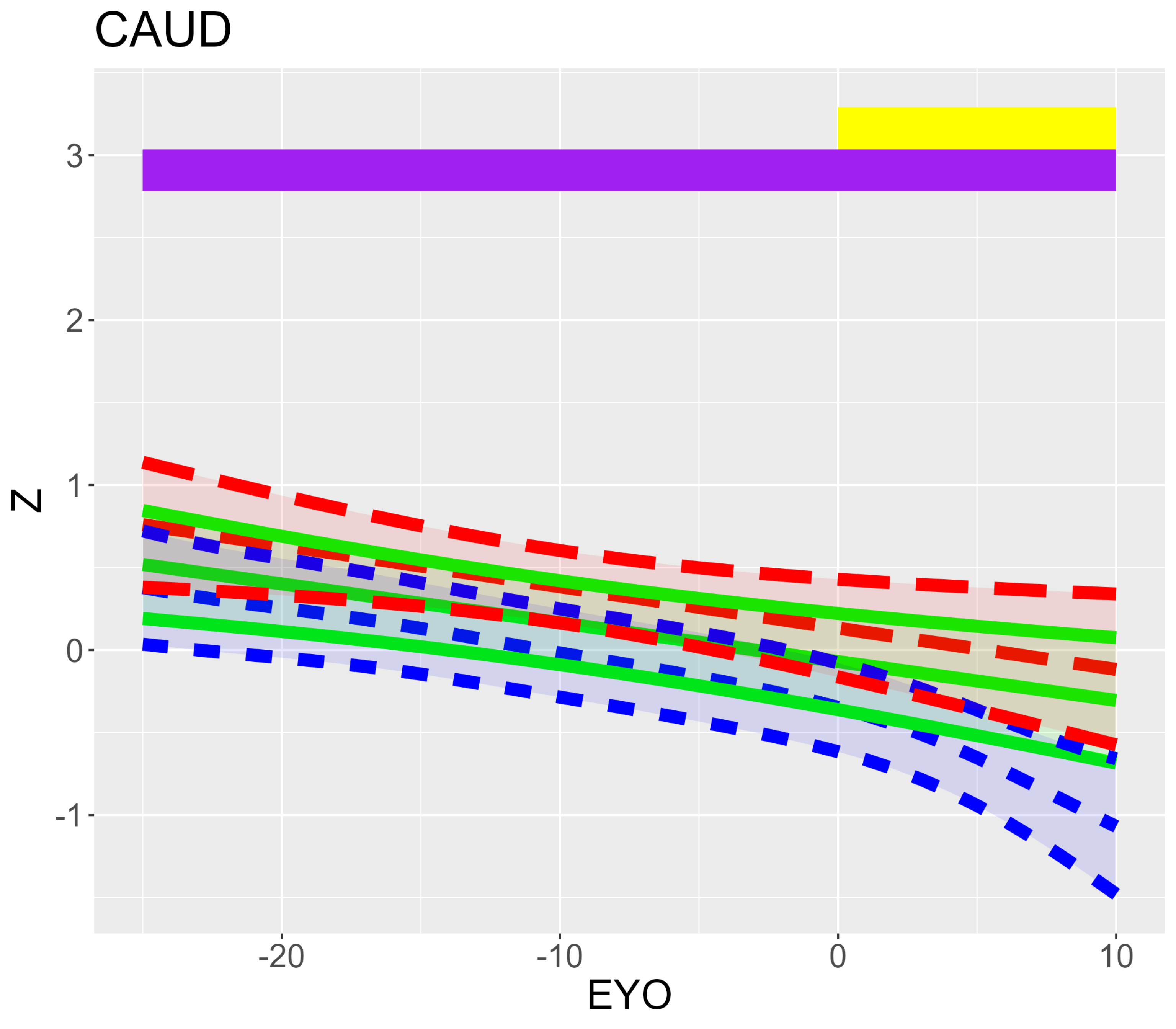
CAUD
3
2
1
0
-1
Z
-20
-10
0
10
EYO

CAUDANTCNG

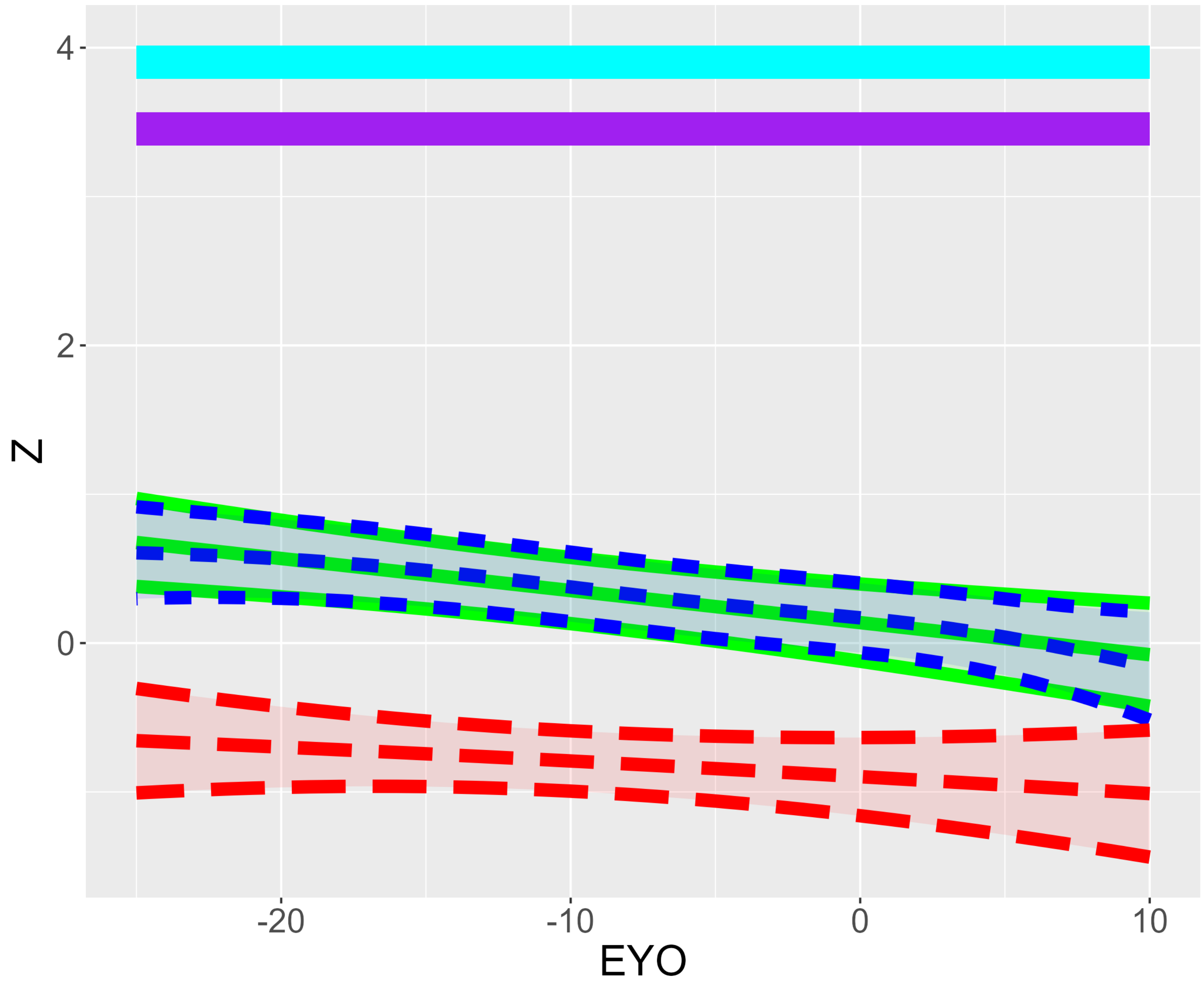

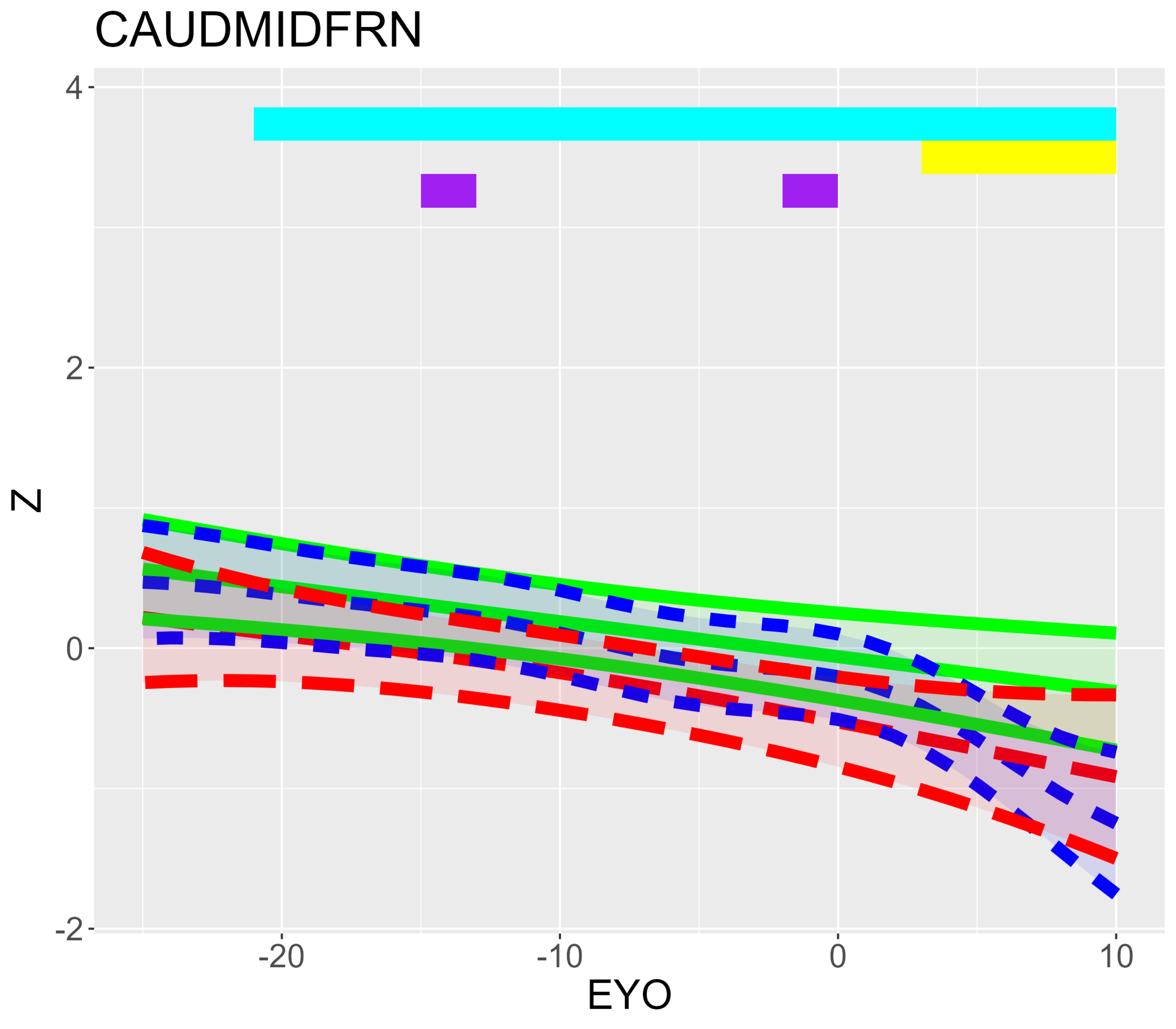

CAUDMIDFRN
4
2
0
-2
Z
-20
-10
0
10
EYO

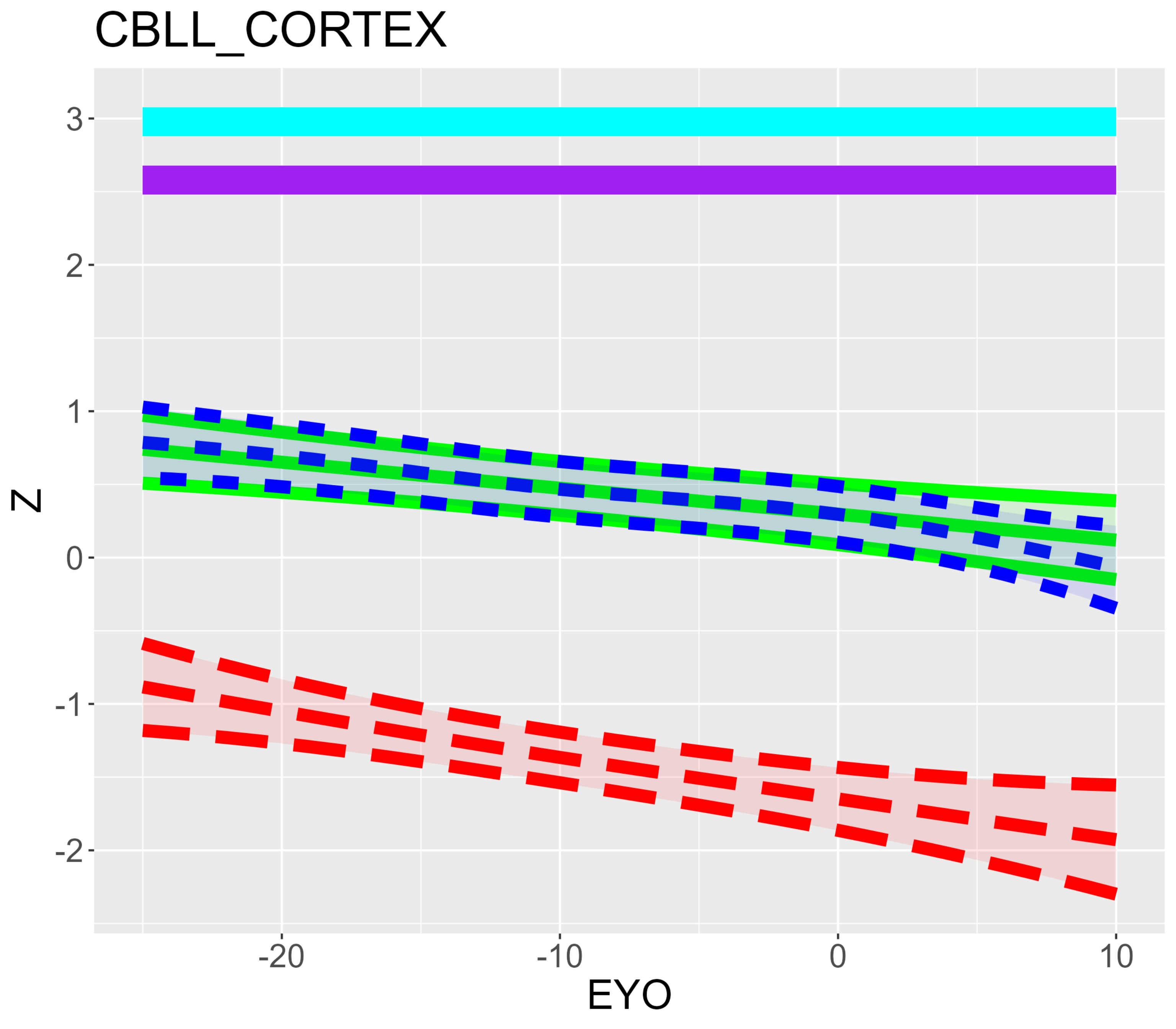
CBLL_CORTEX
3
2
1
0
-1
-2
Z
-20
-10
0
10
EYO

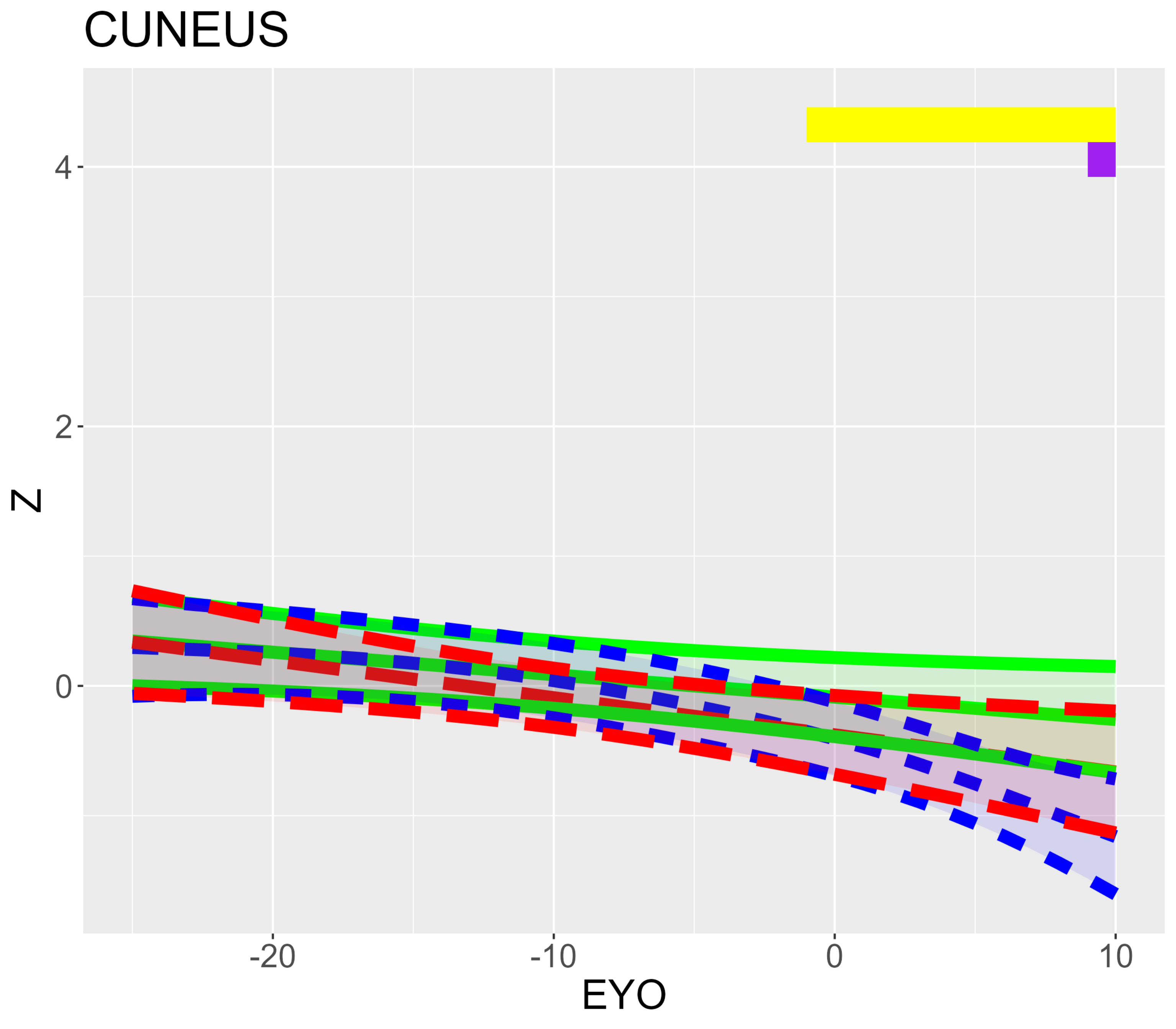
CUNEUS
4
2
0
Z
-20
-10
0
10
EYO

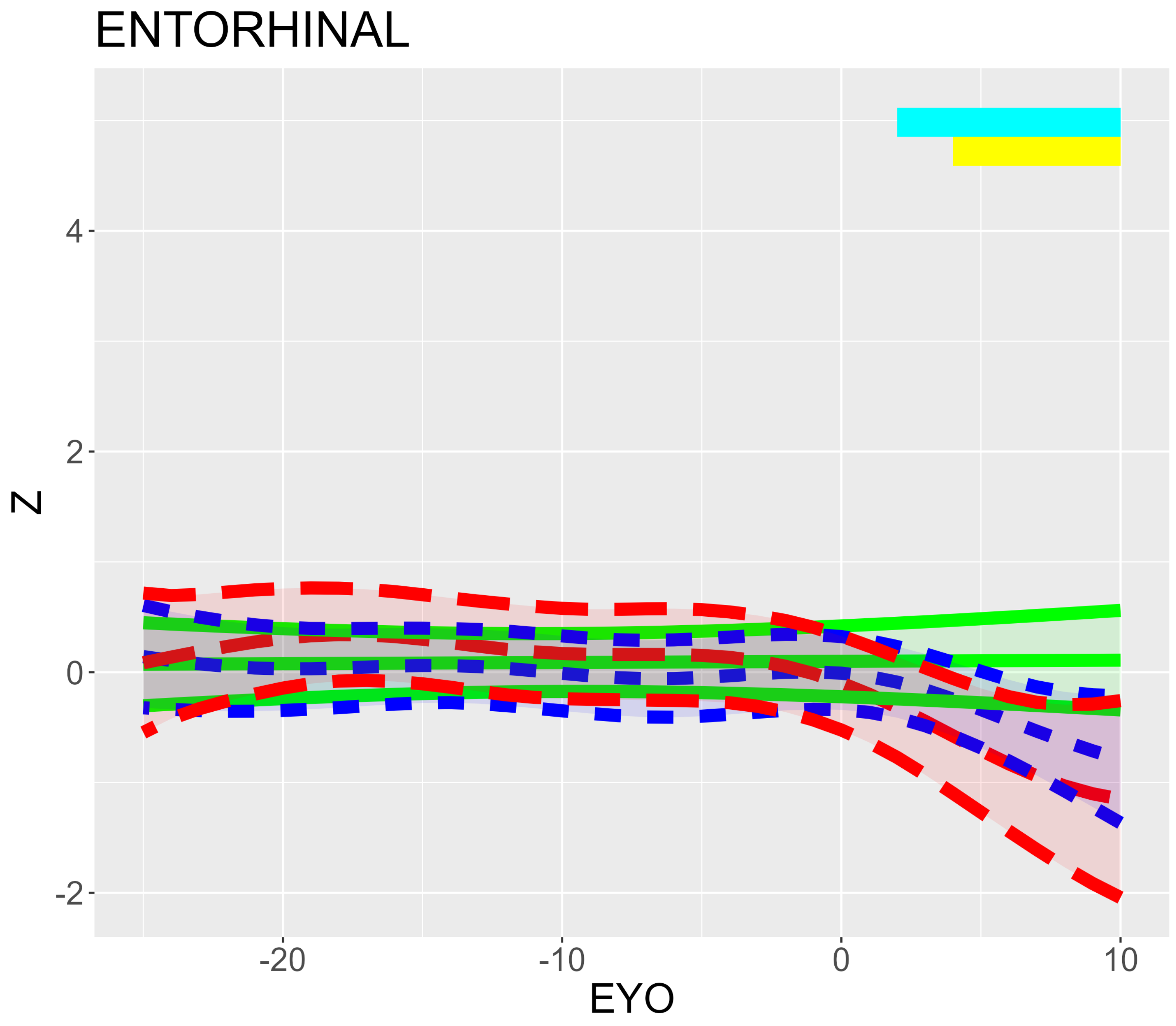

ENTORHINAL
Z
EYO
4
2
0
-2
-20
-10
0
10

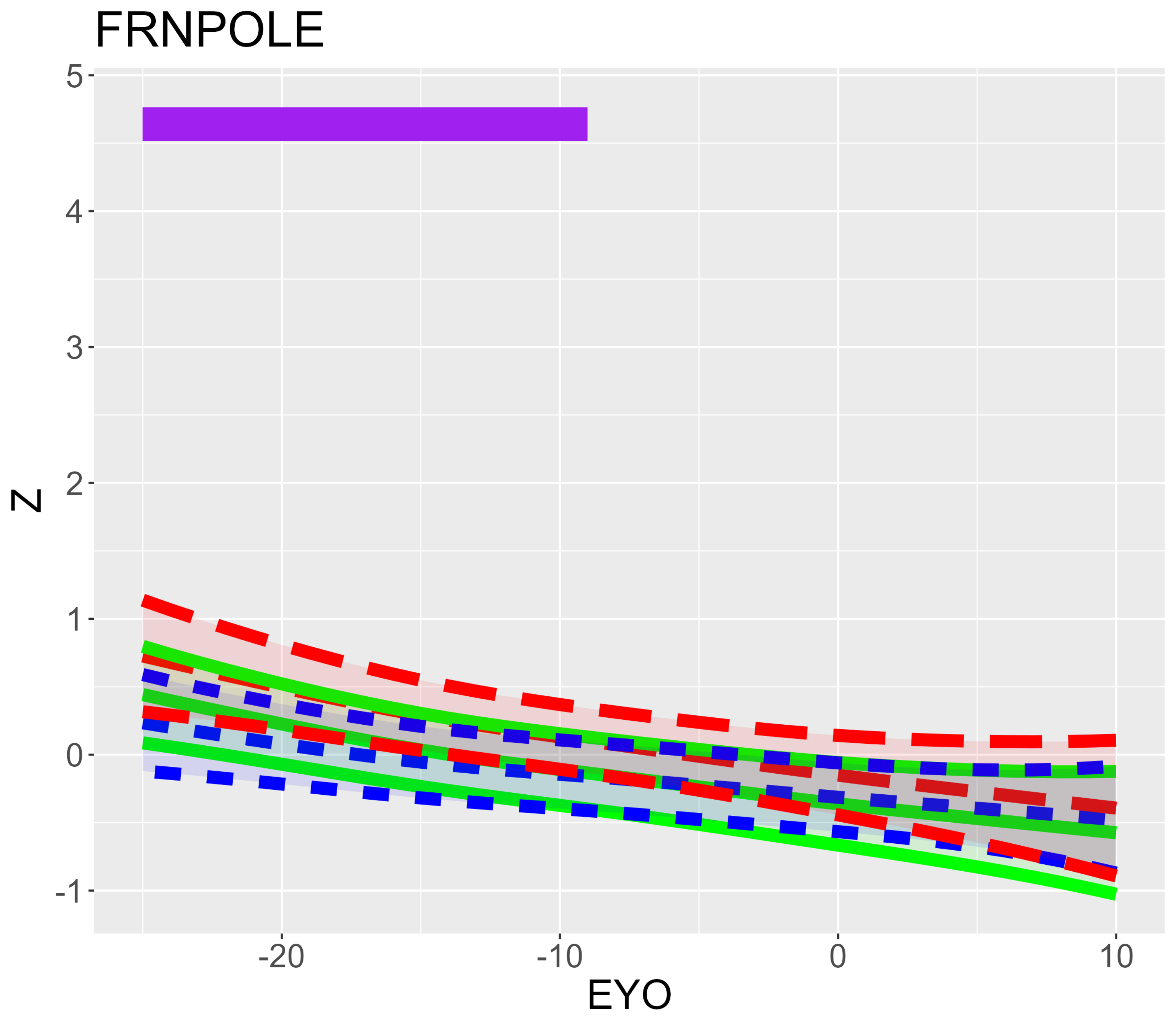
FRNPOLE
Z
5
4
3
2
1
0
-1
-20
-10
0
10
EYO

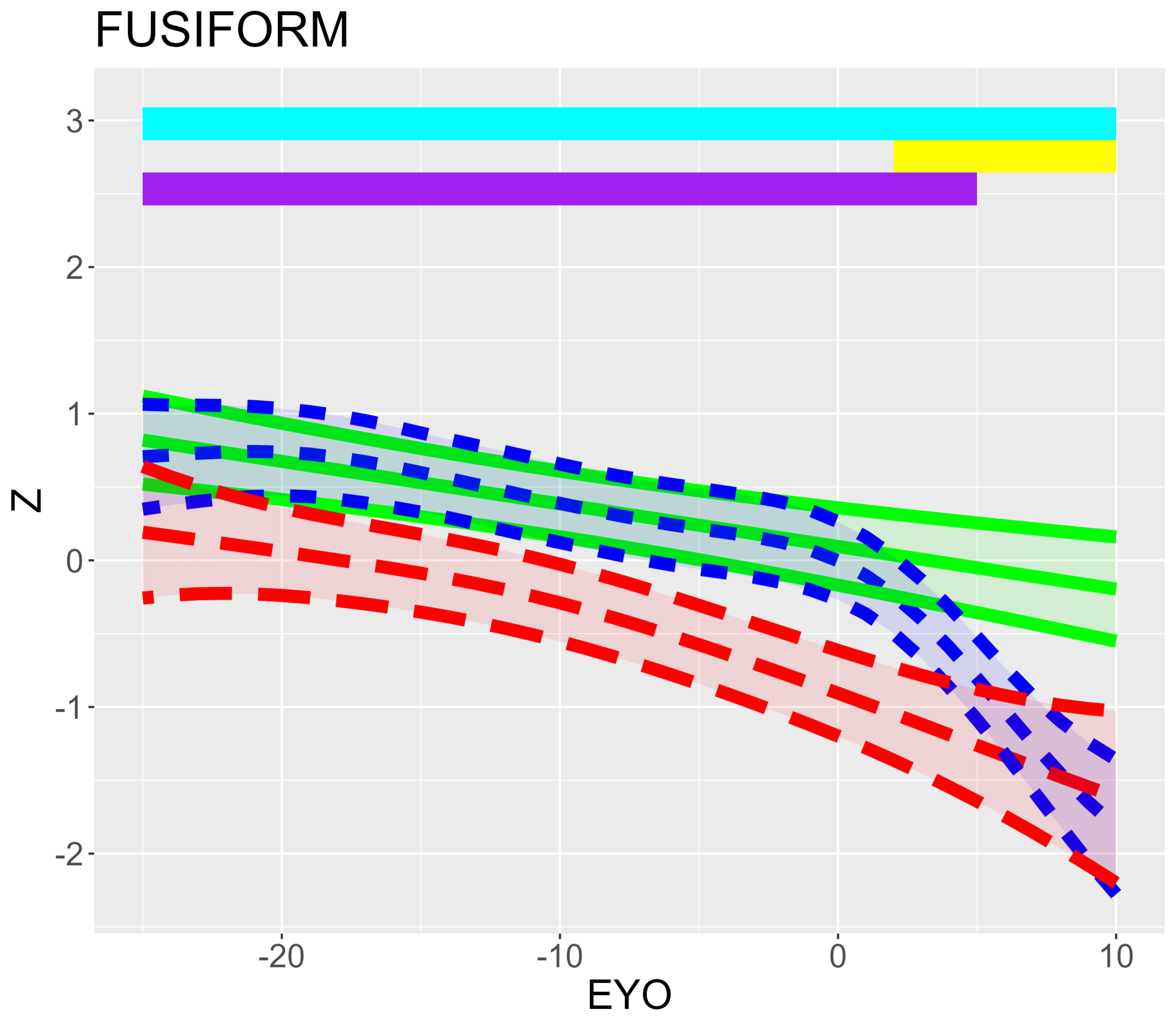

FUSIFORM
3
2
1
0
-1
-2
Z
-20
-10
0
10
EYO

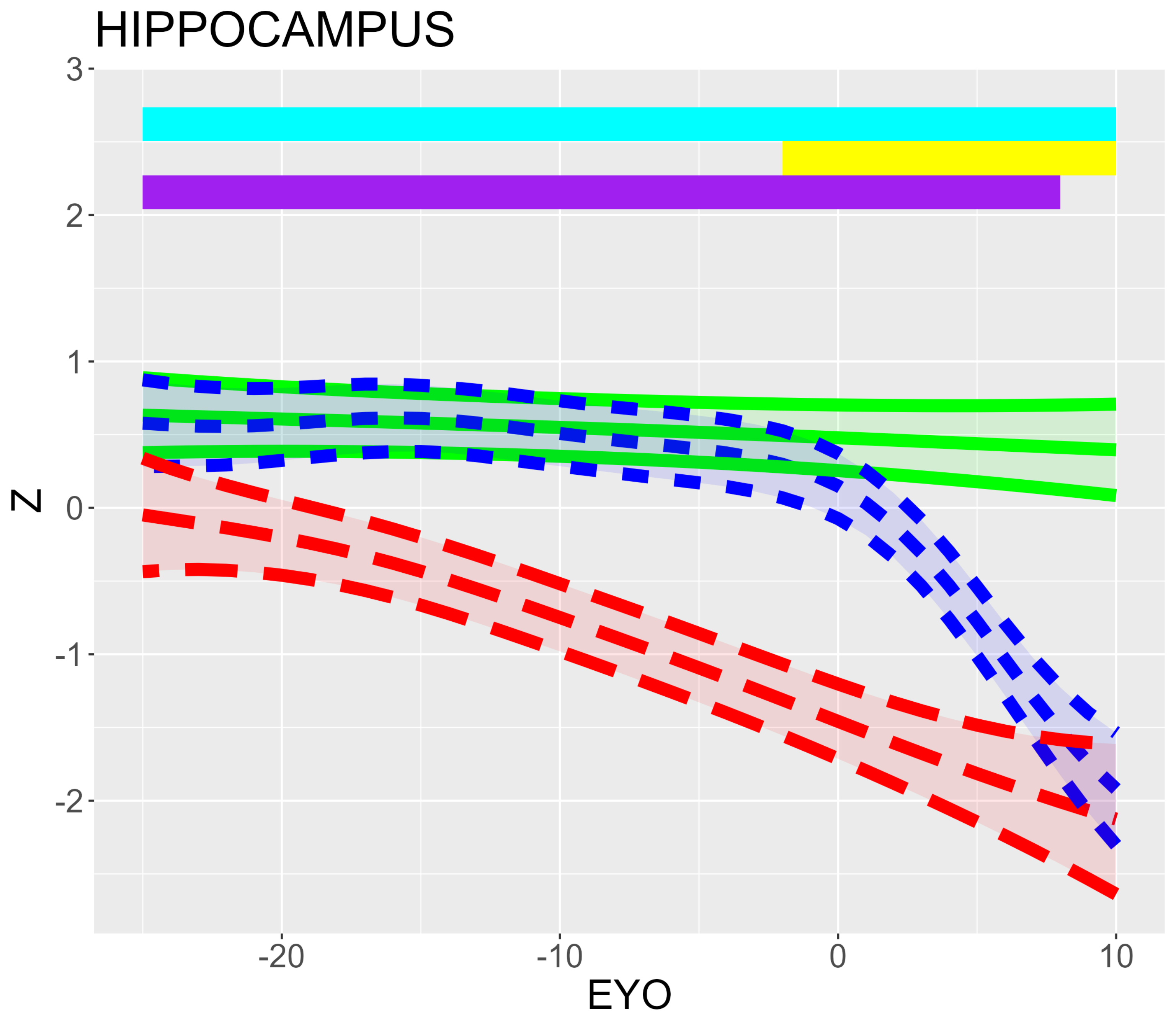
HIPPOCAMPUS
3
2
1
0
-1
-2
Z
-20
-10
0
10
EYO

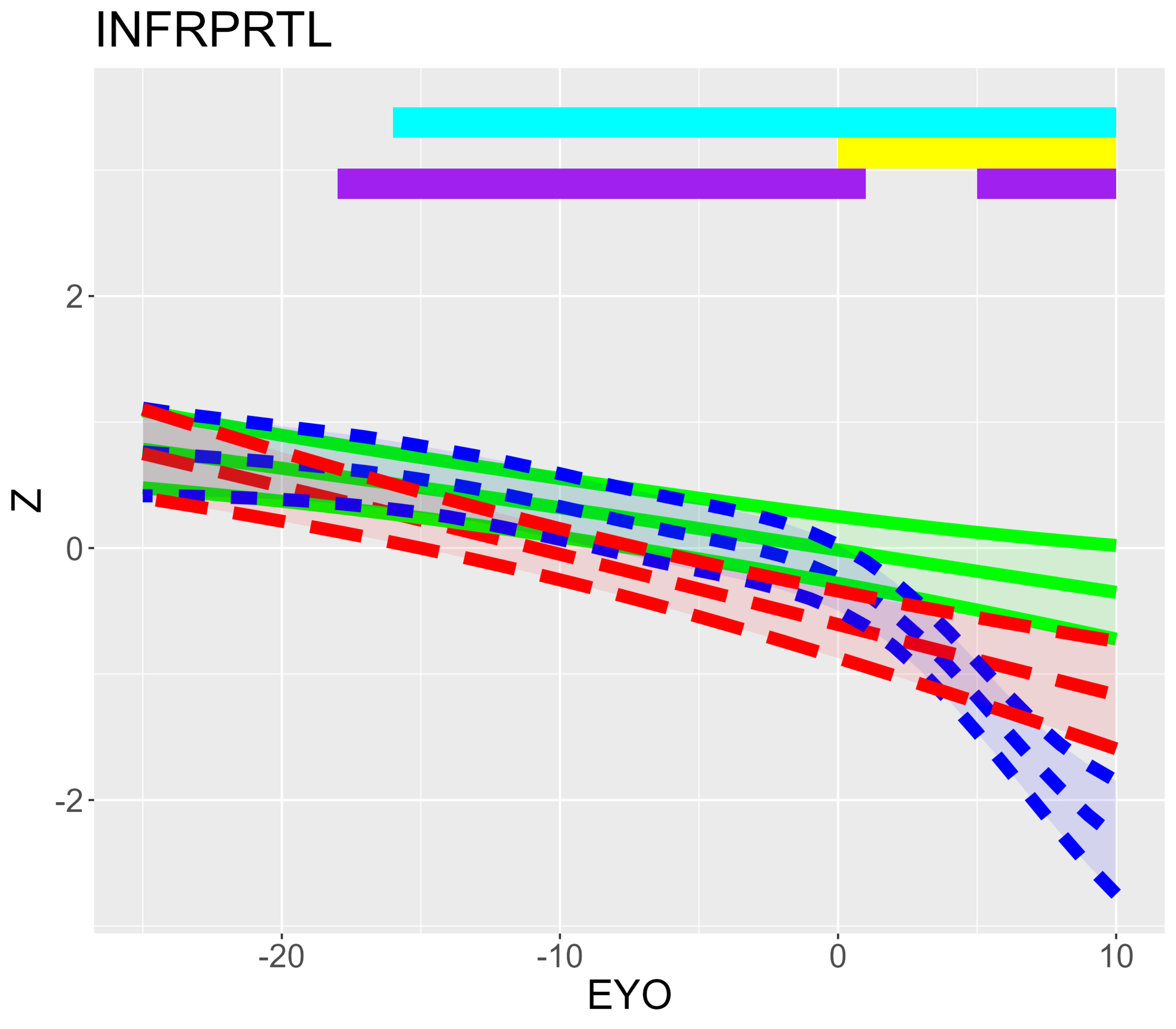

INFRPRTL
2
0
-2
Z
-20
-10
0
10
EYO

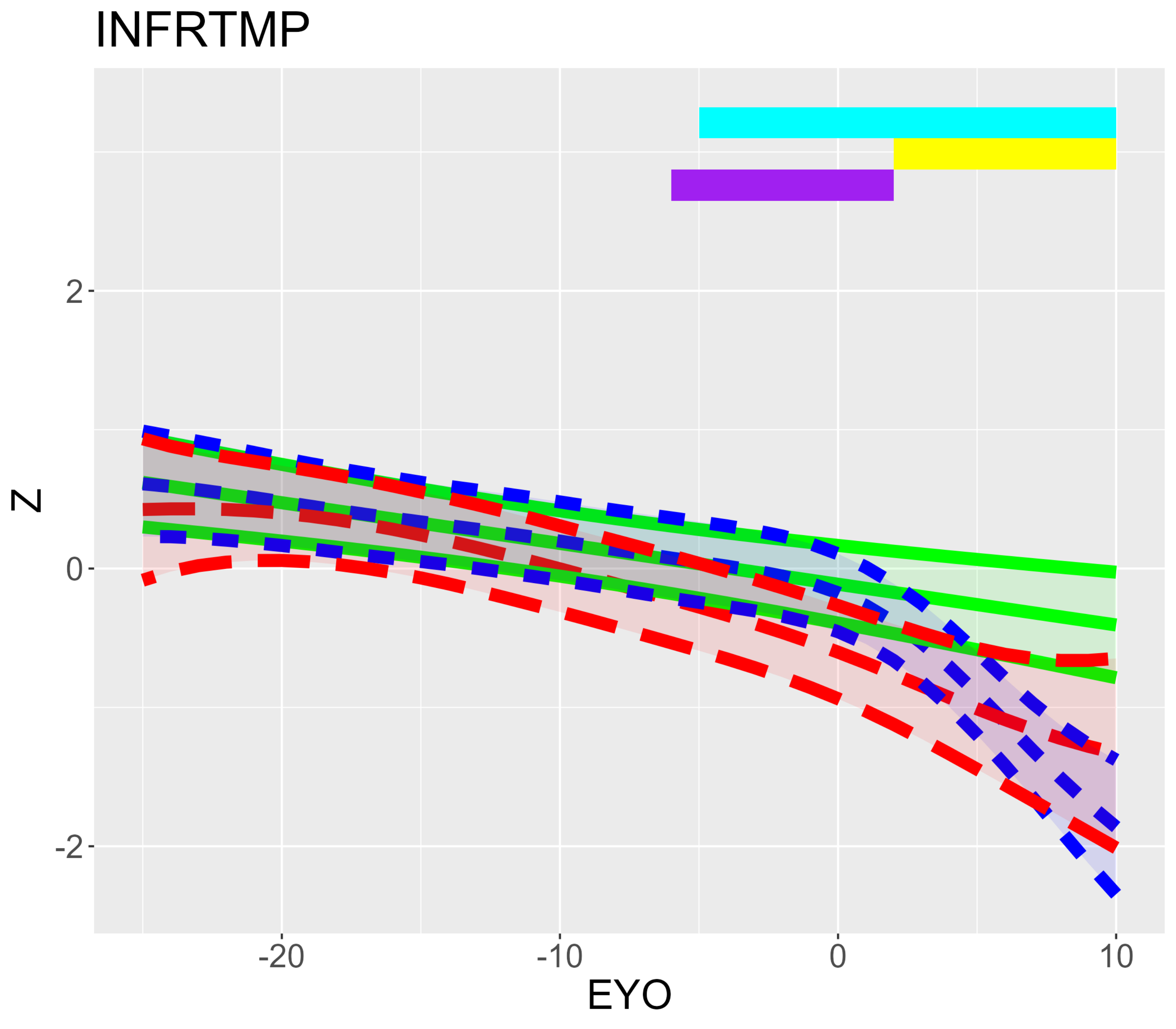
INFRTMP
2
0
-2
Z
-20
-10
0
10
EYO

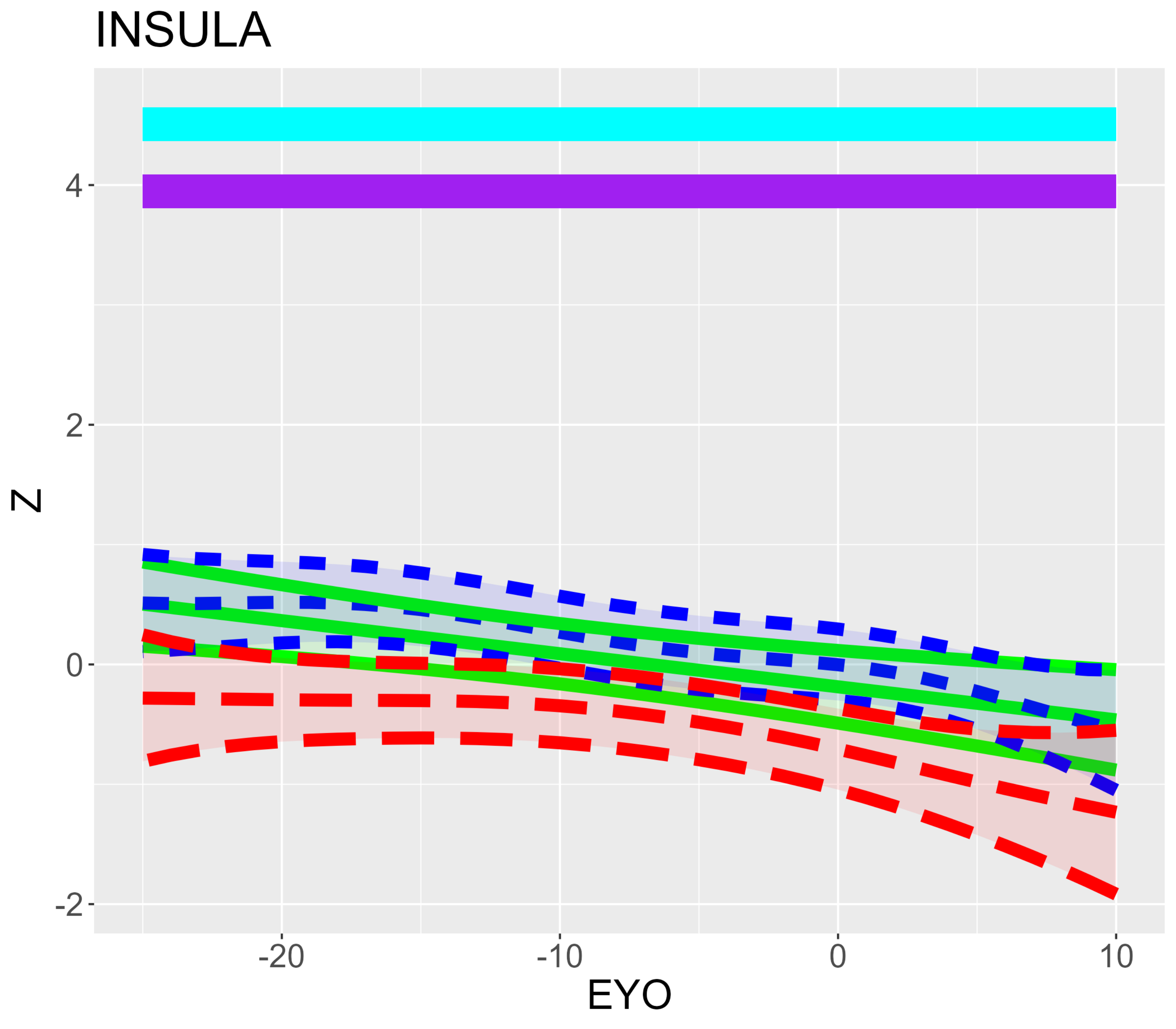
INSULA
4
2
0
-2
Z
-20
-10
0
10
EYO

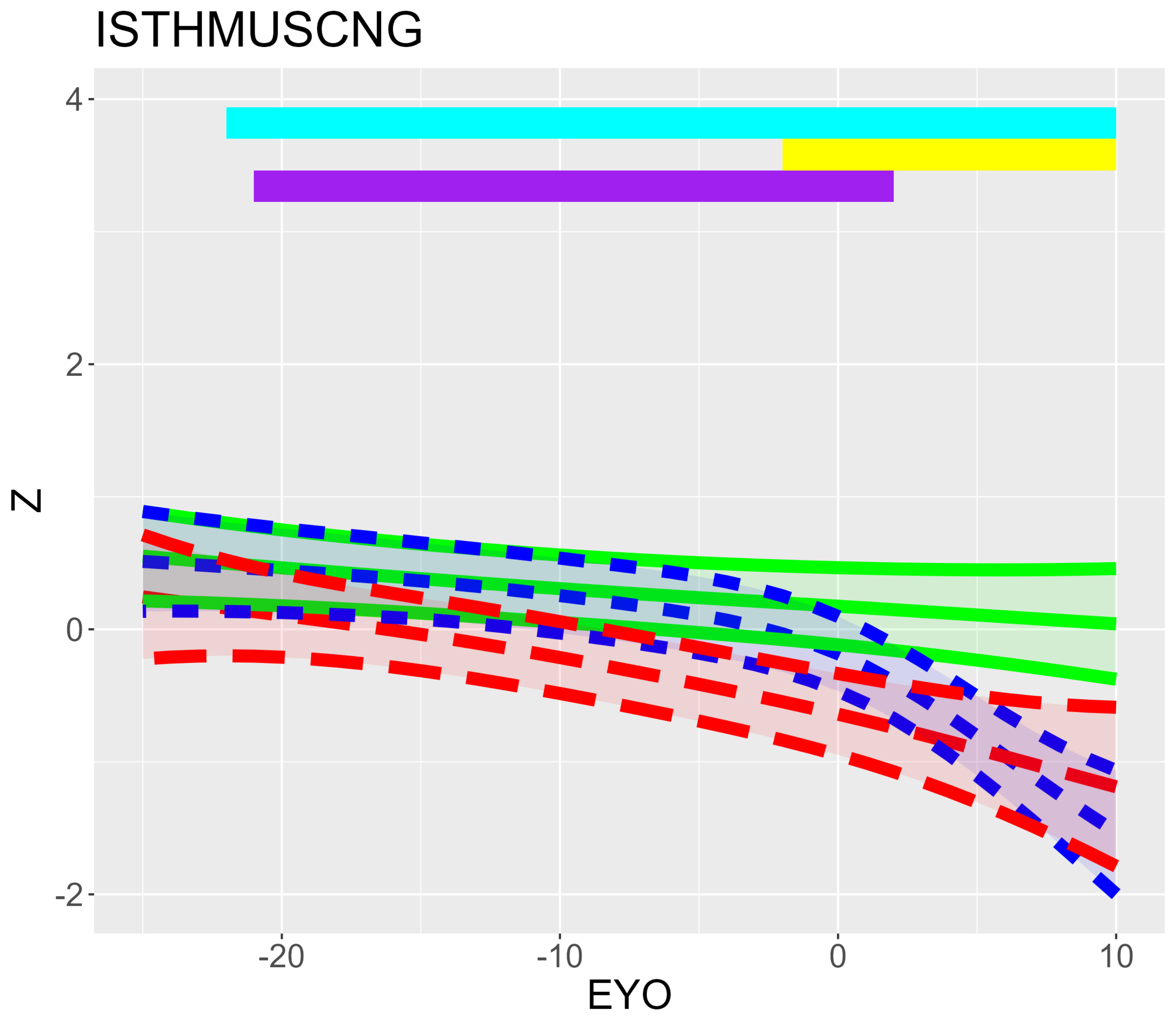
ISTHMUSCNG
4
2
0
-2
Z
-20
-10
0
10
EYO

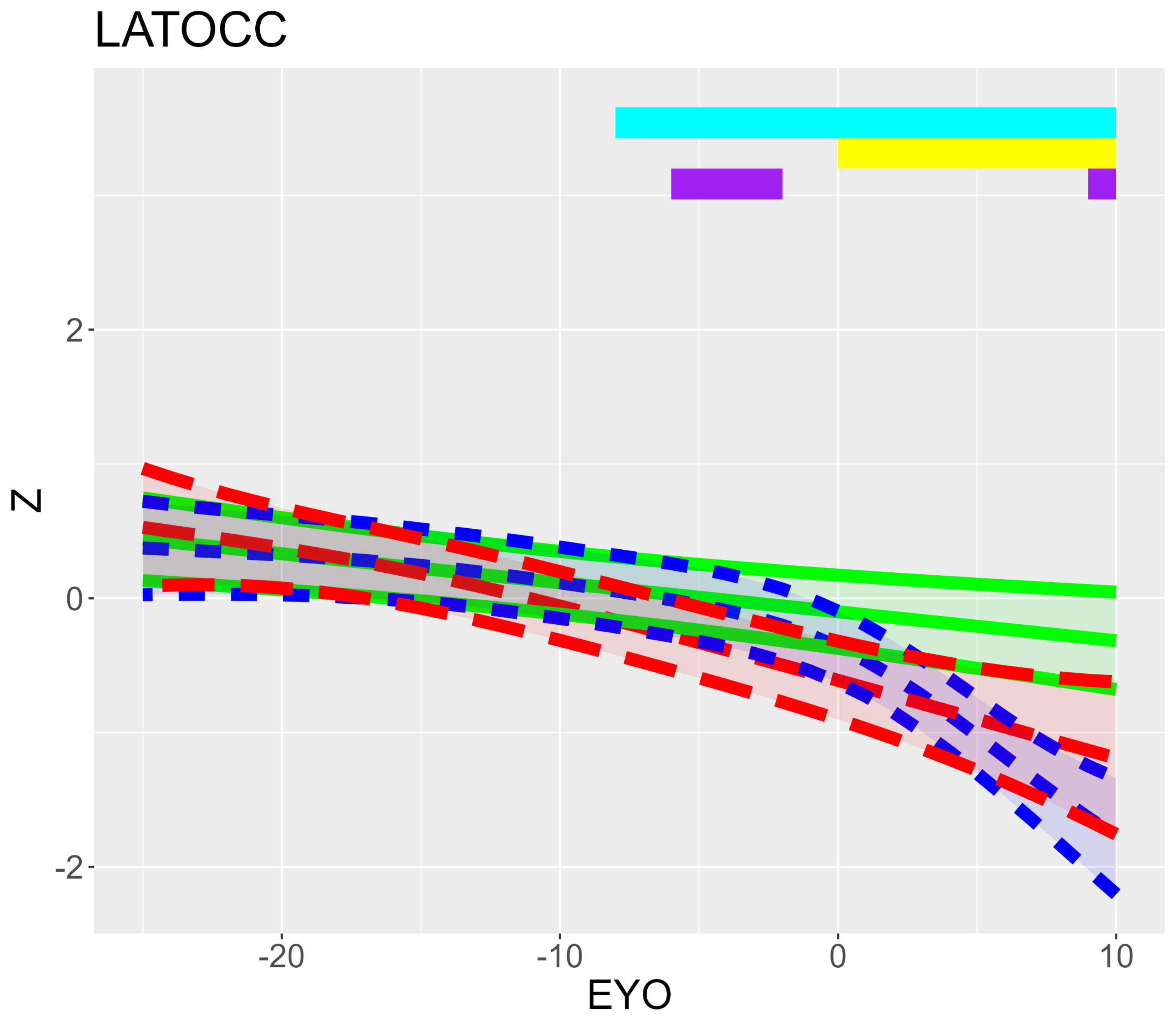
LATOCC
Z
2
0
-2
-20
-10
0
10
EYO

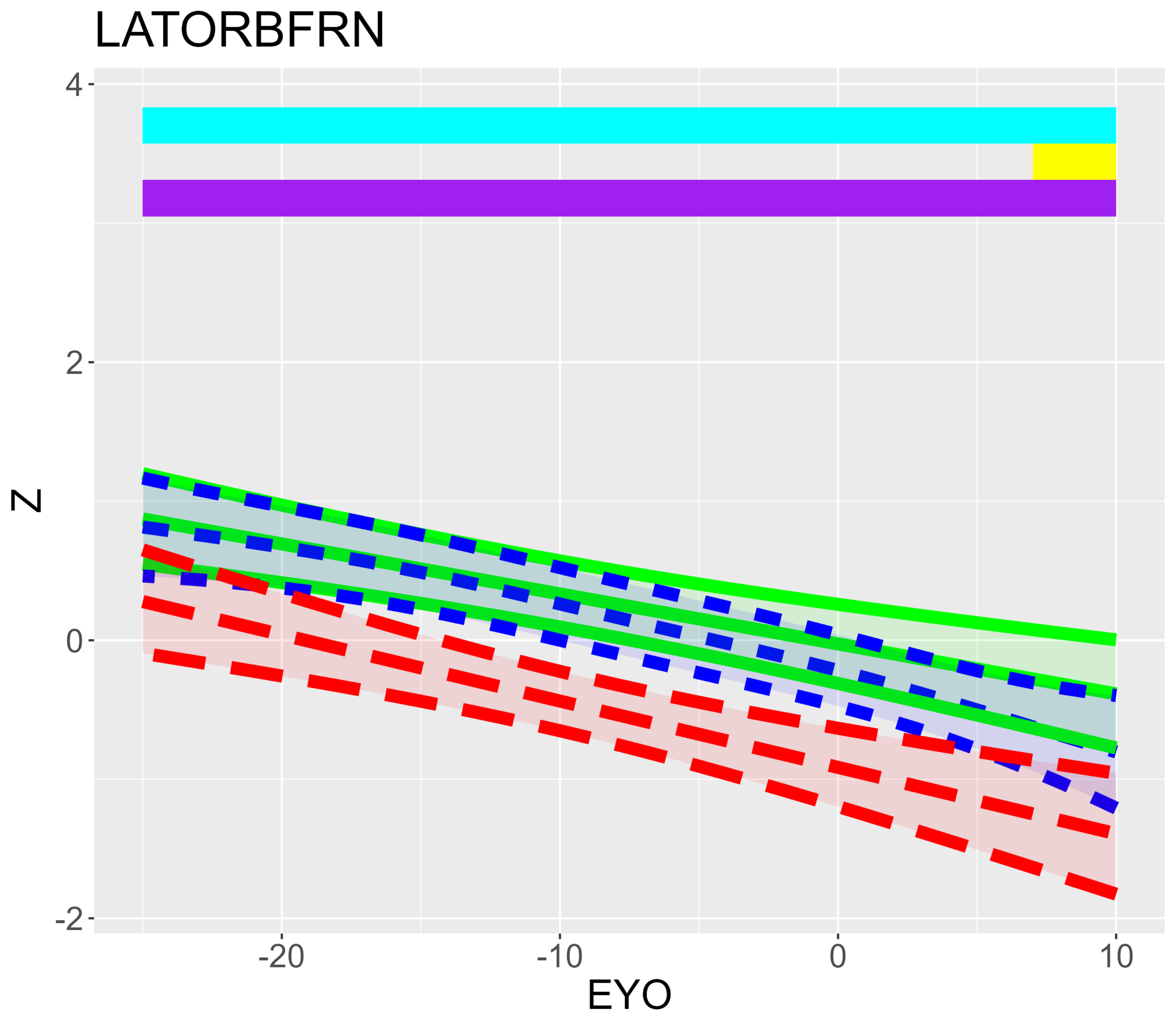
LATORBFRN
4
2
0
-2
Z
-20
-10
0
10
EYO

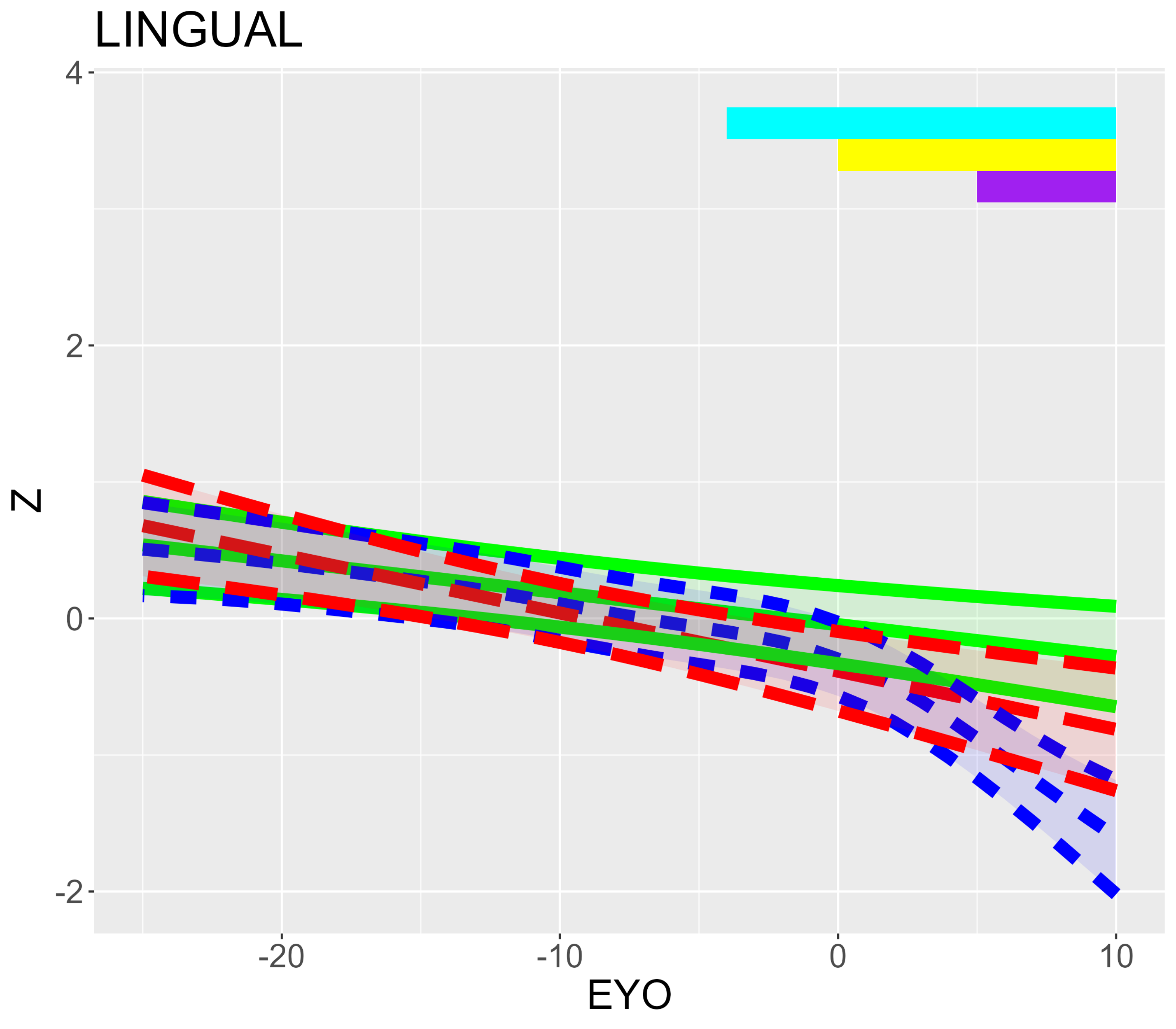
LINGUAL
4
2
0
-2
Z
-20
-10
0
10
EYO

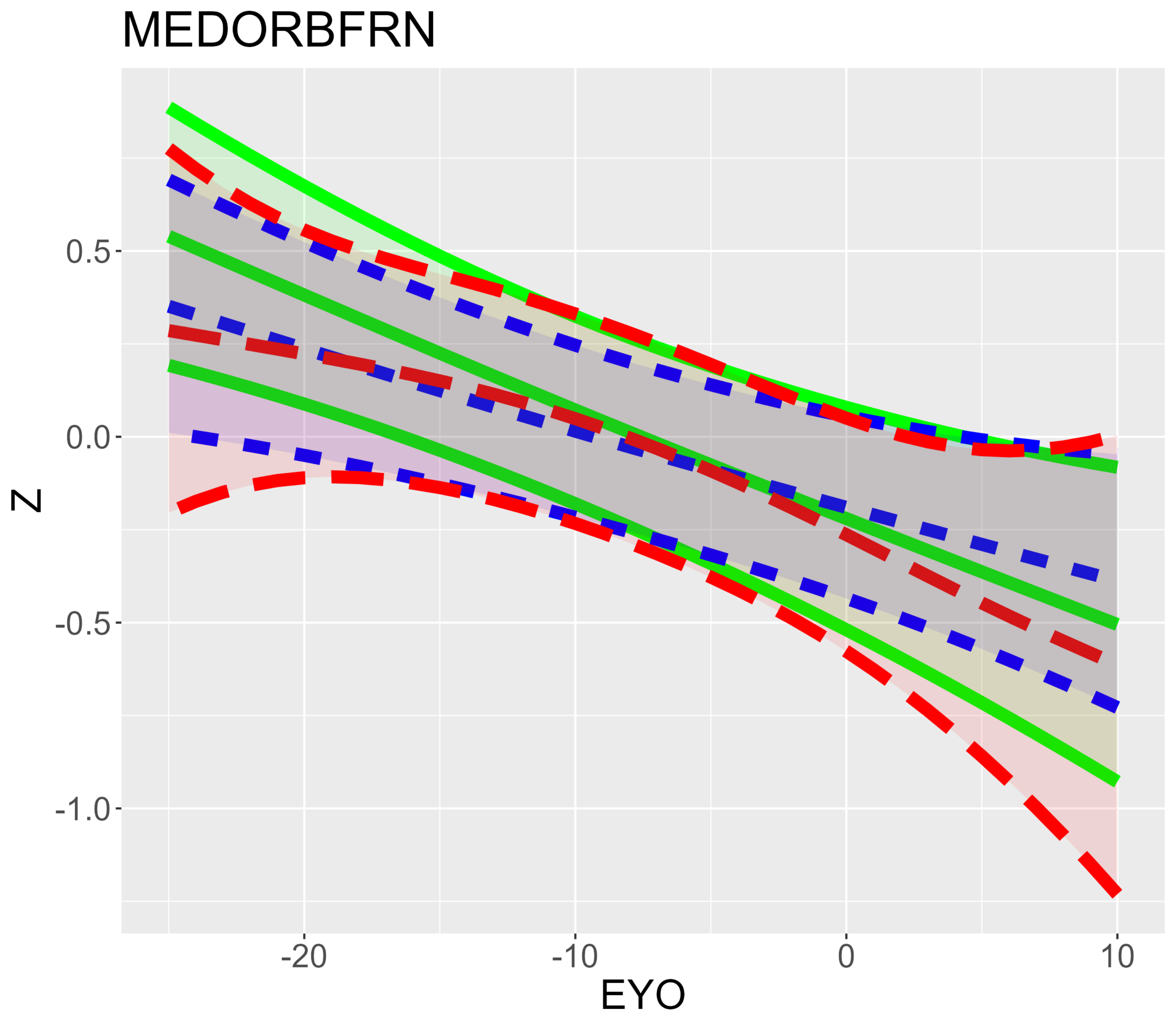
MEDORBFRN
0.5
0.0
-0.5
-1.0
Z
-20
-10
0
10
EYO

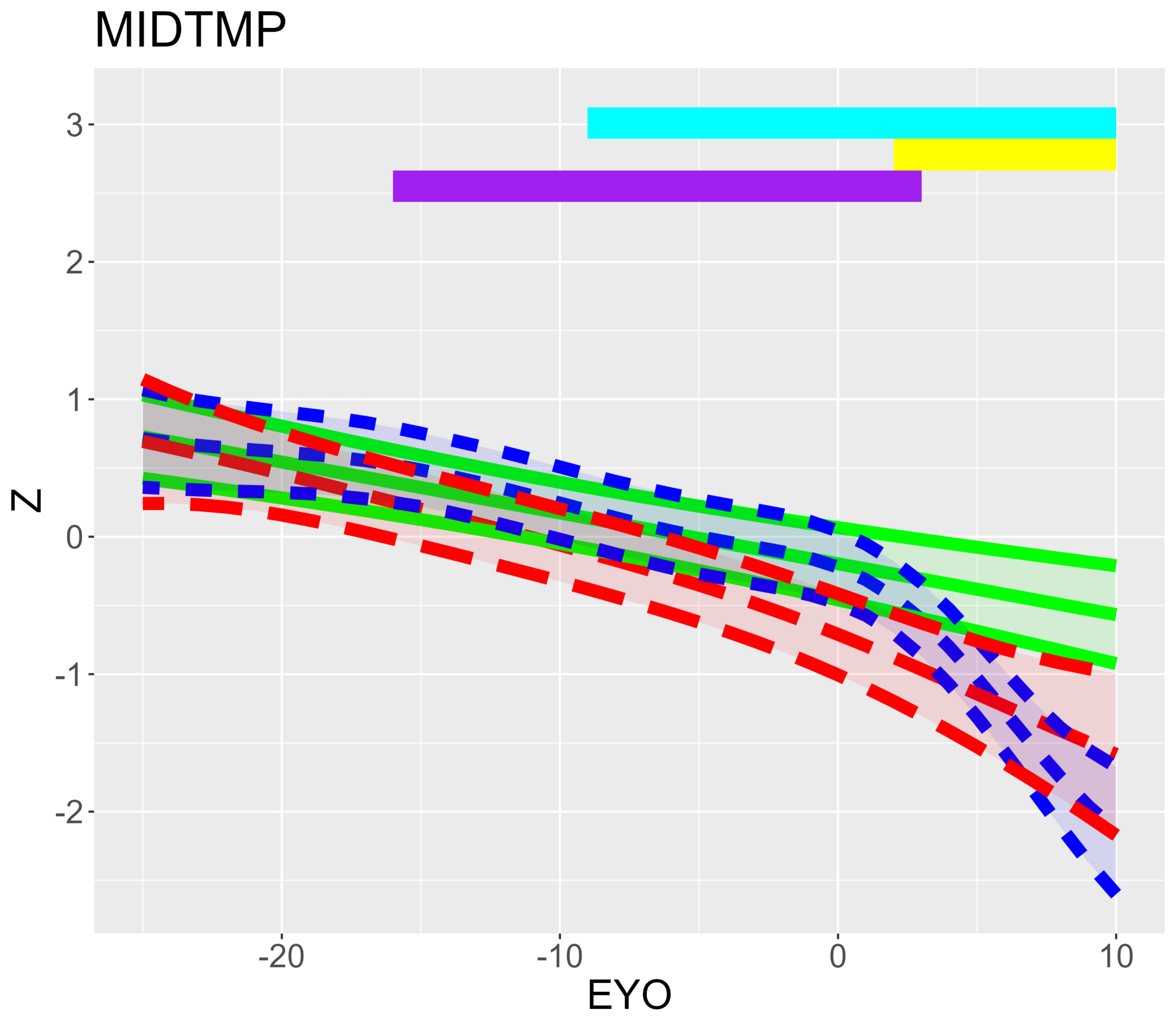
MIDTMP
Z
3
2
1
0
-1
-2
-20
-10
0
10
EYO

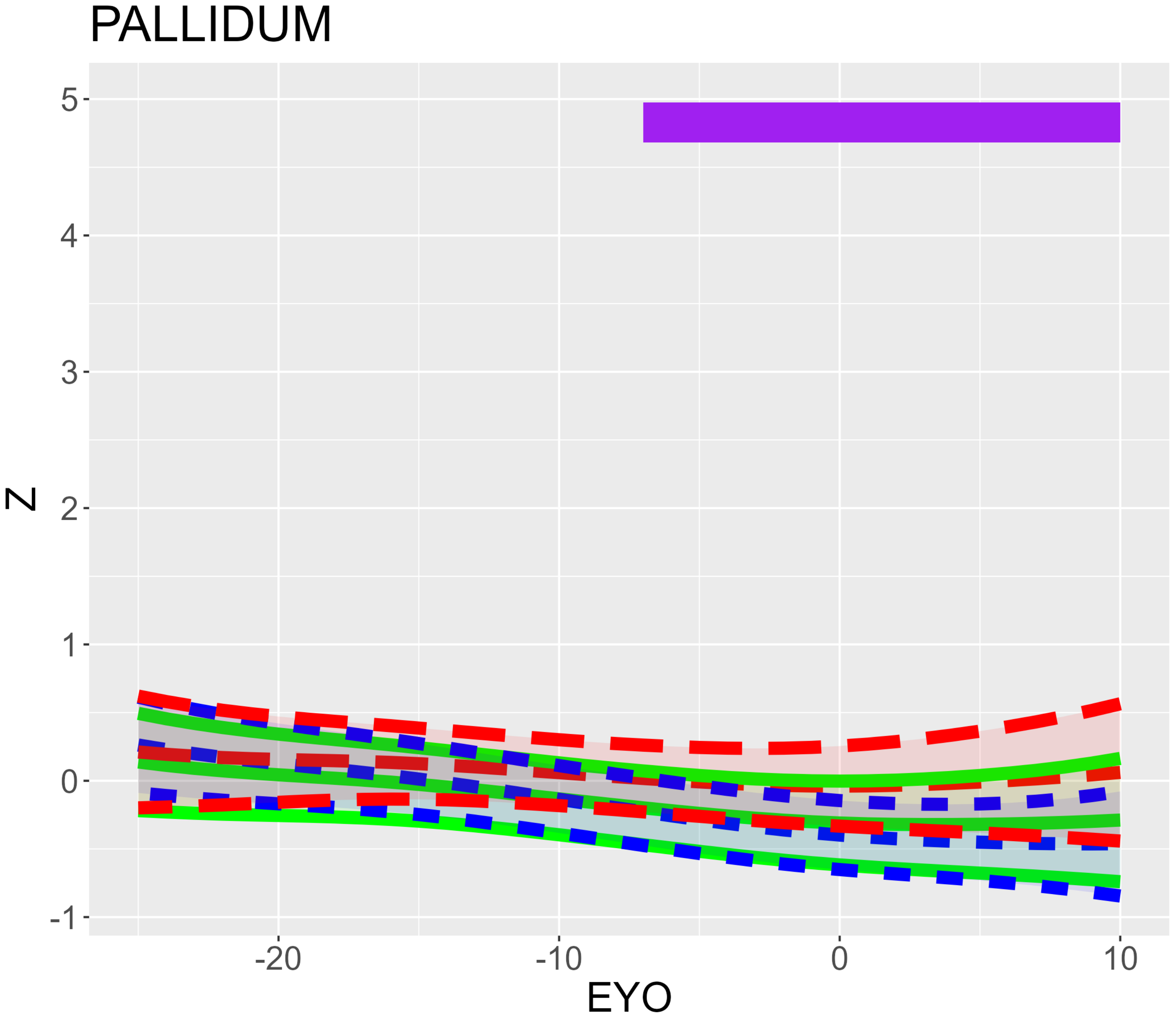

PALLIDUM
5
4
3
2
1
0
-1
Z
-20
-10
0
10
EYO

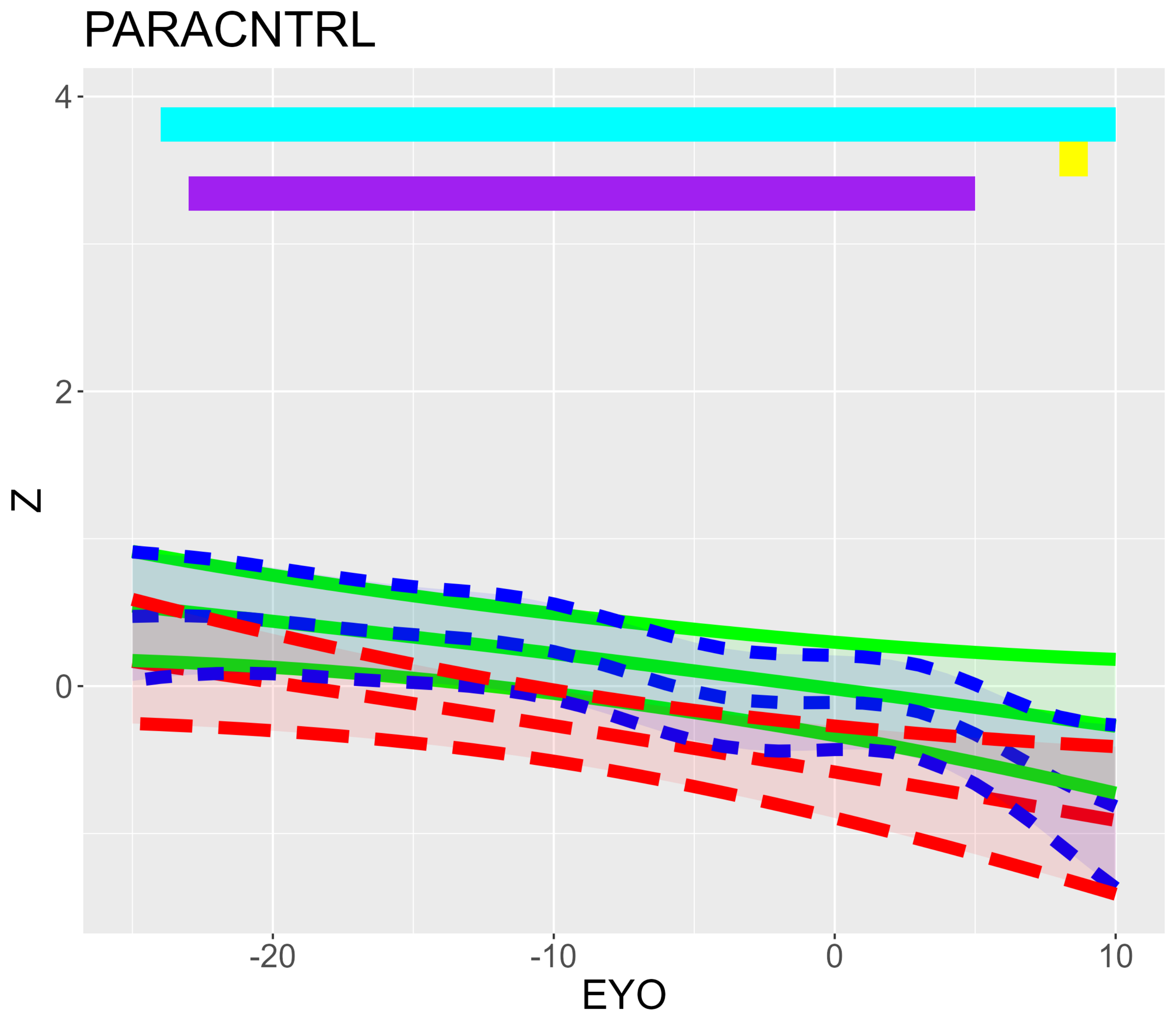
PARACNTRL
4
2
0
Z
-20
-10
0
10
EYO

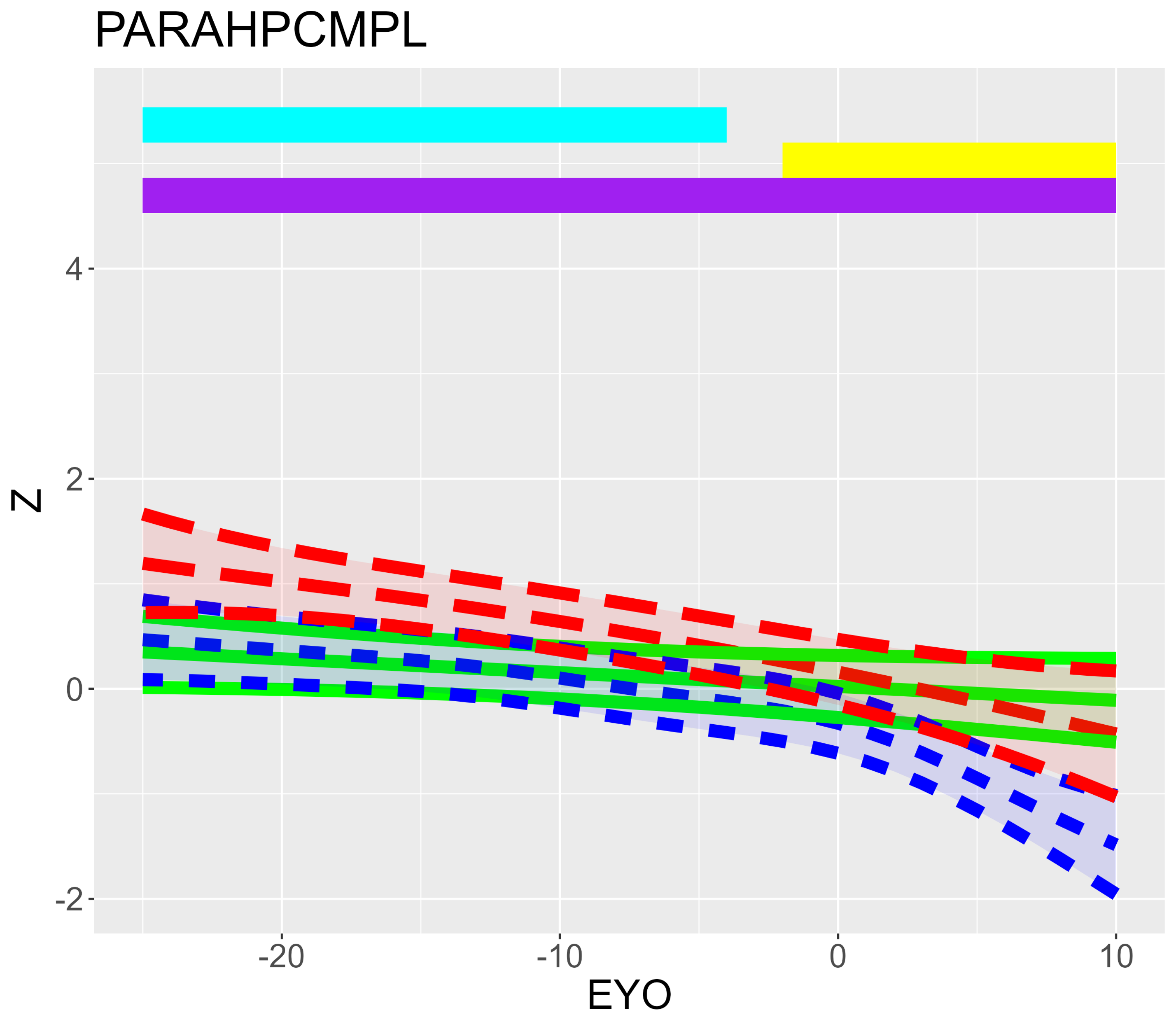
PARAHPCMPL
Z
4
2
0
-2
-20
-10
0
10
EYO

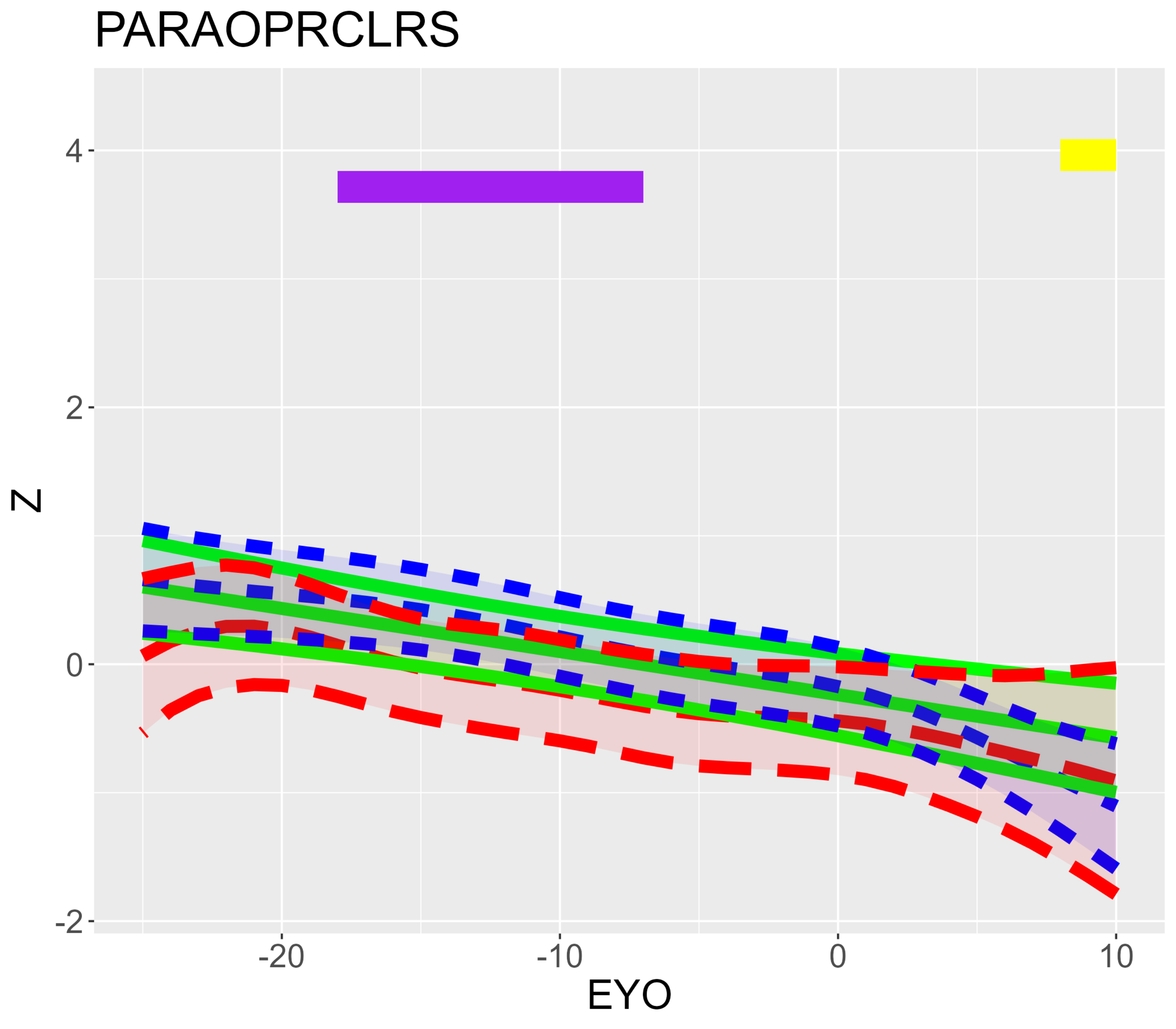
PARAOPRCLRS
4
2
0
-2
Z
-20
-10
0
10
EYO

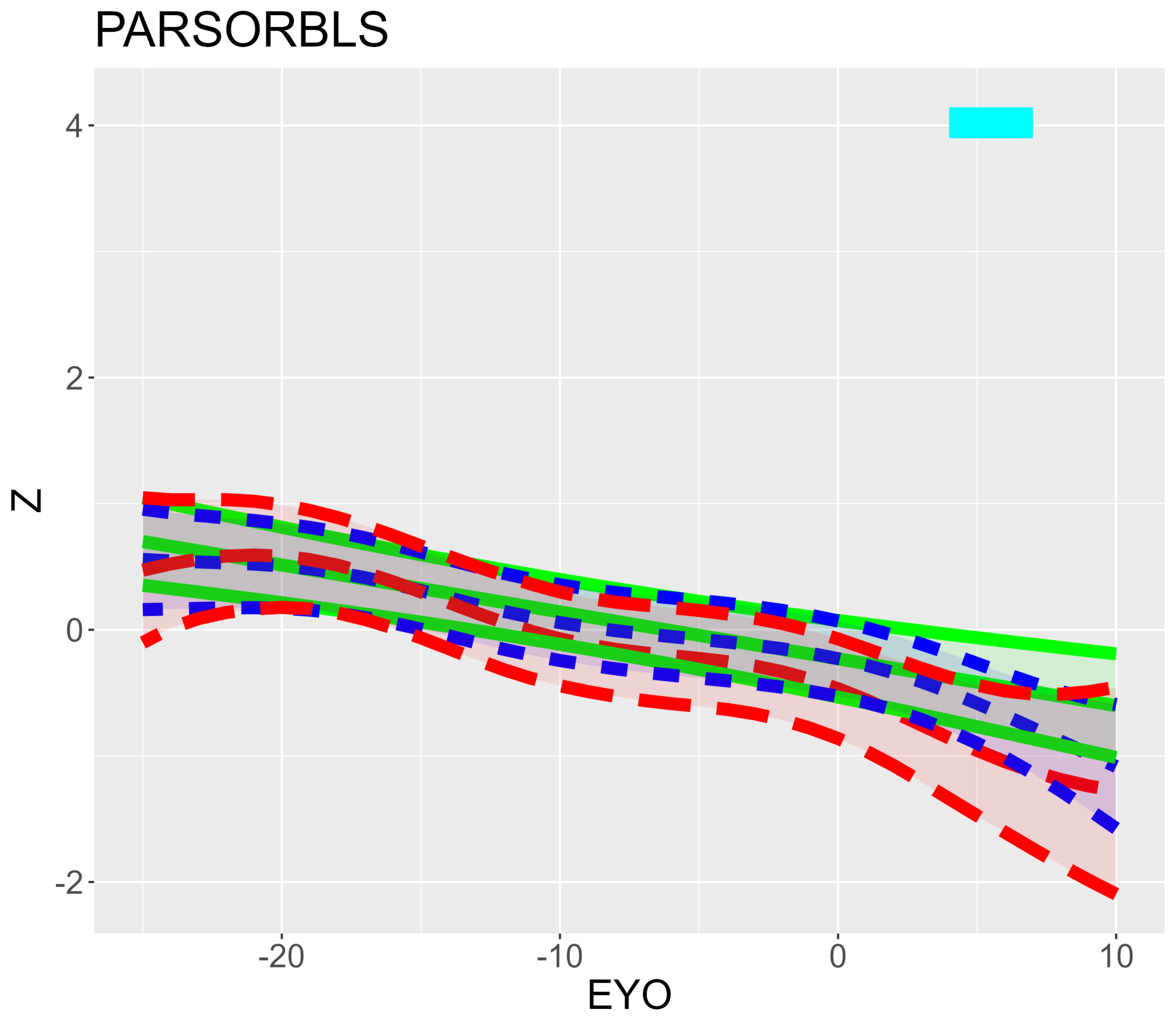
PARSORBLS
Z
4
2
0
-2
-20
-10
0
10
EYO

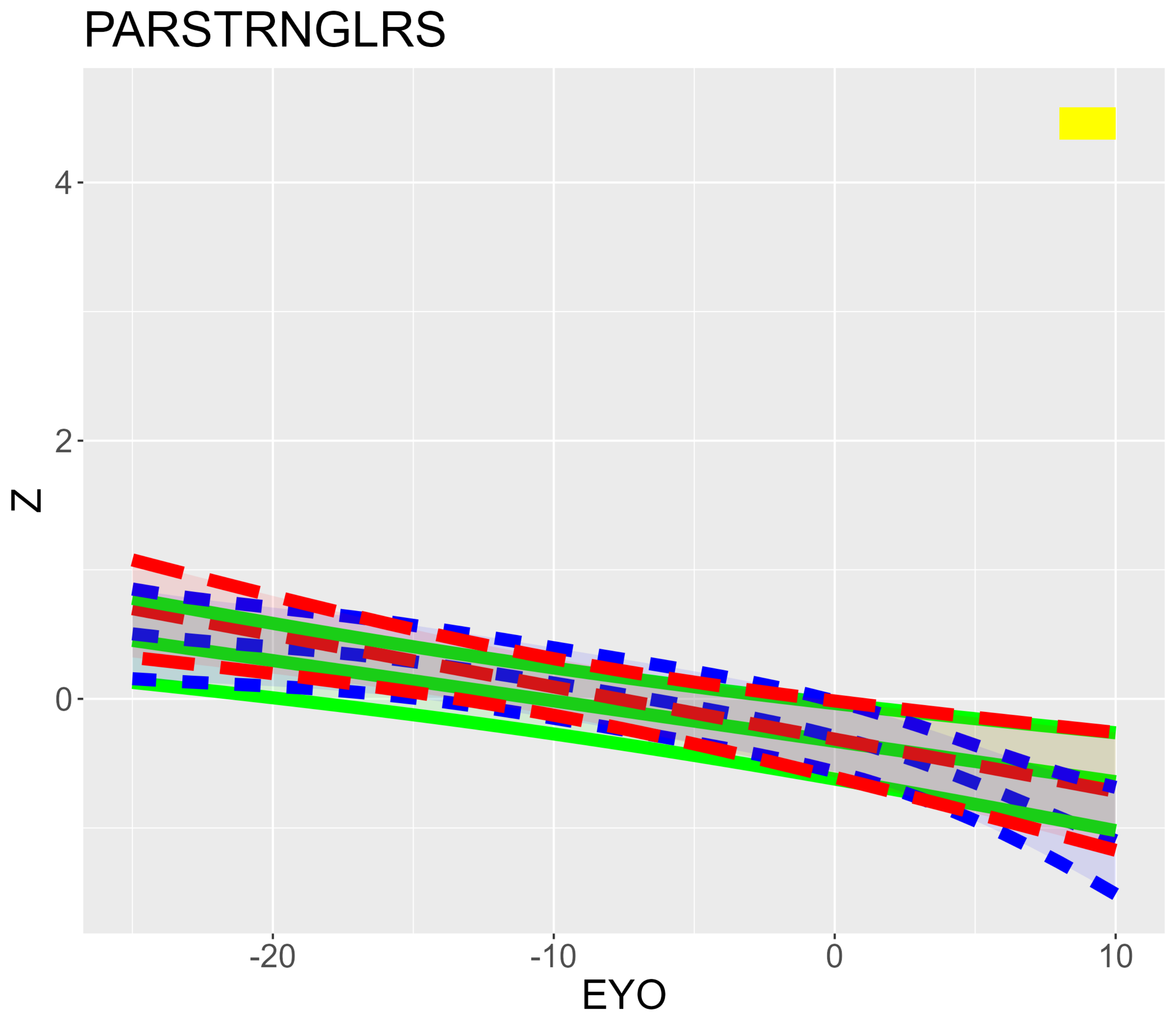
PARSTRNGLRS
4
2
0
Z
-20
-10
0
10
EYO

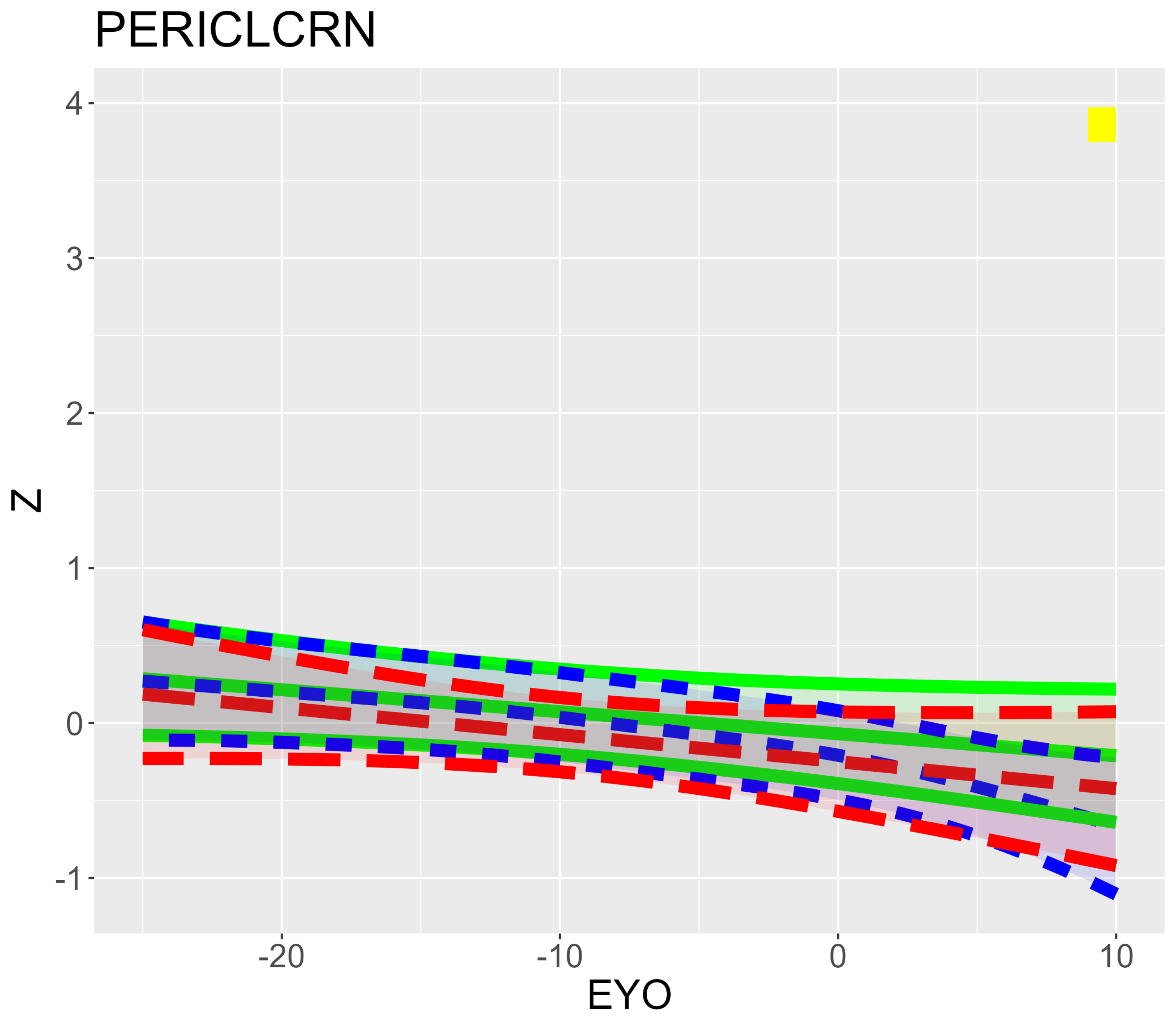
PERICLCRN
4
3
2
1
0
-1
Z
-20
-10
0
10
EYO

POSTCNG

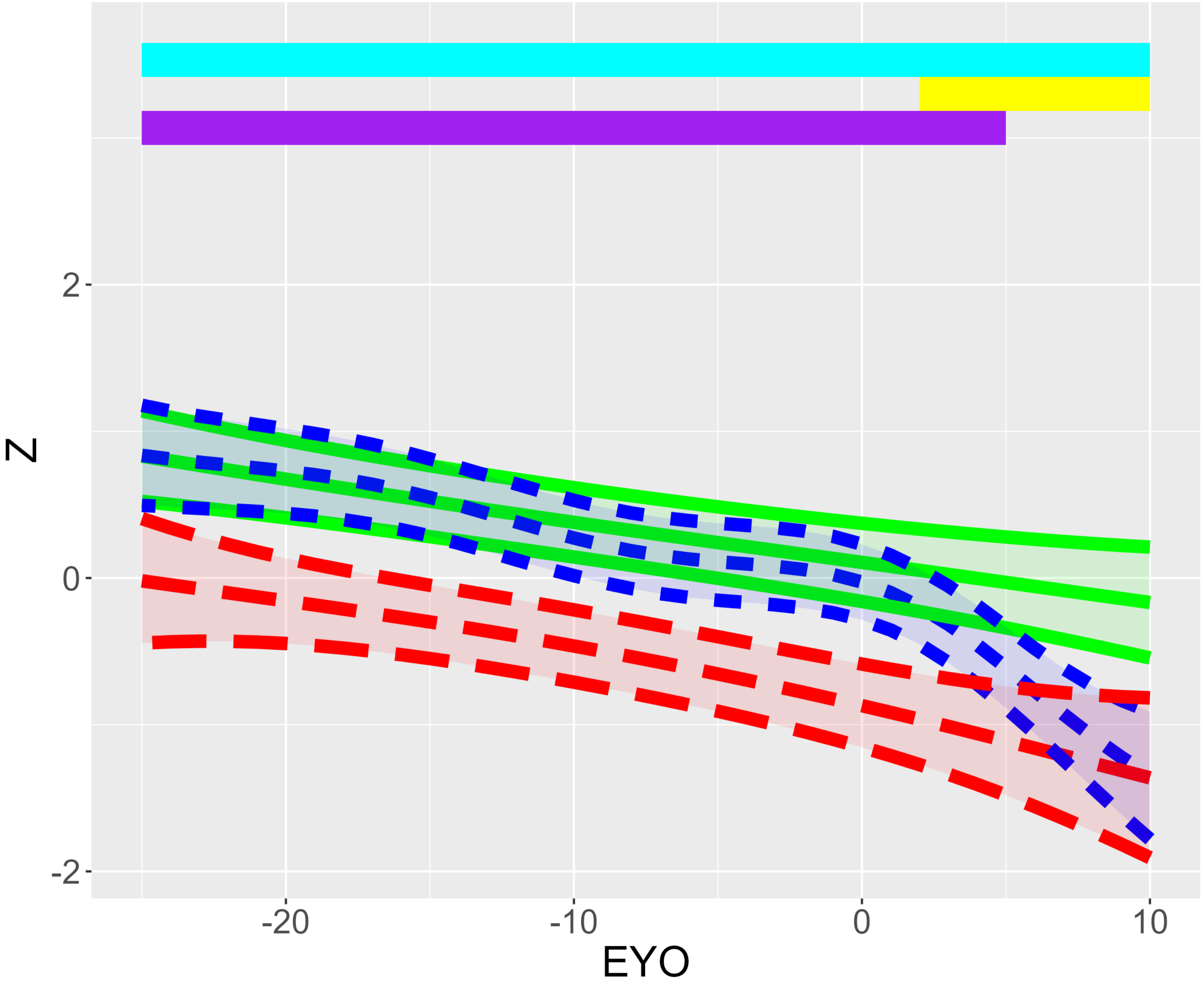

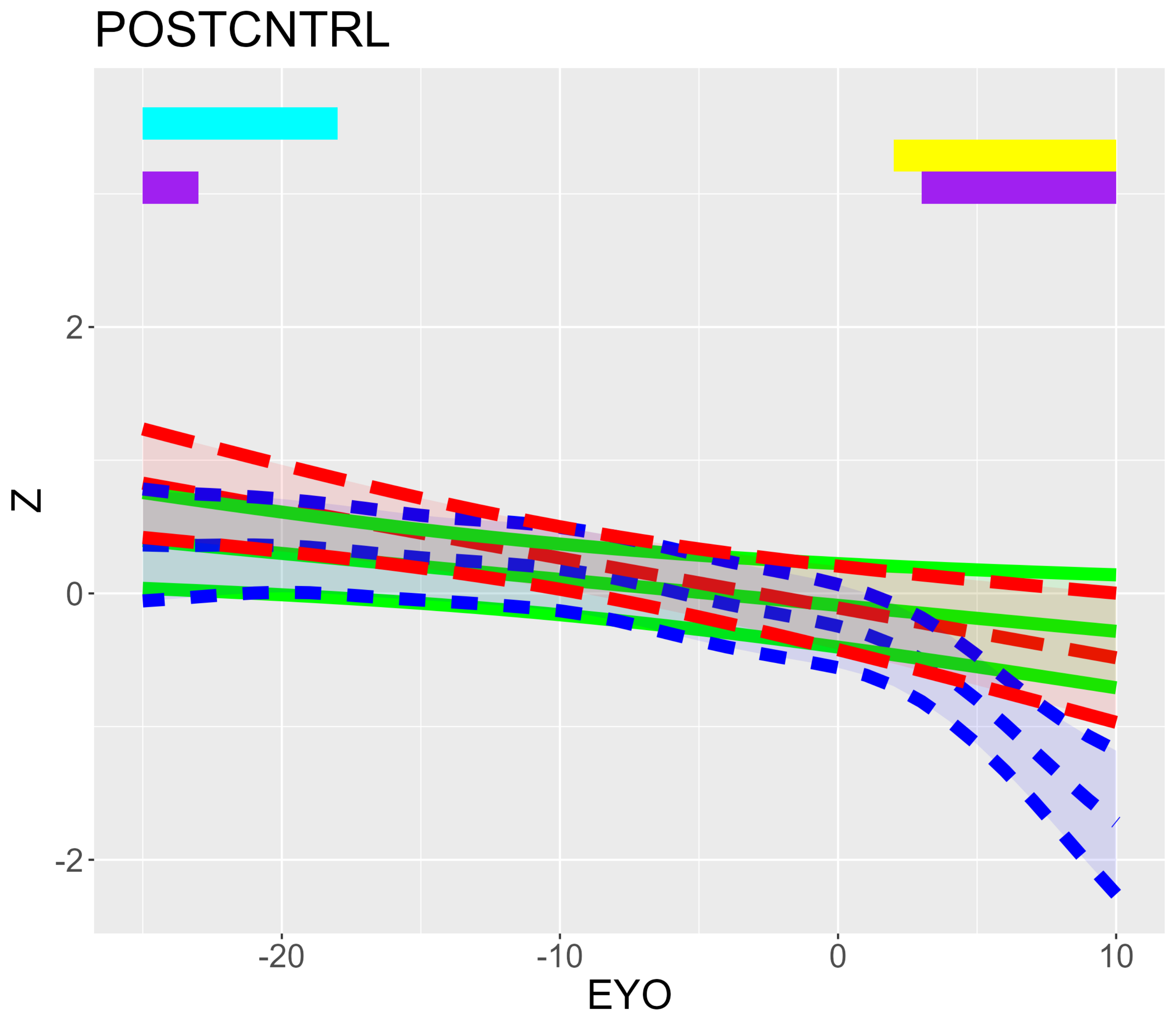
POSTCNTRL
2
0
-2
Z
-20
-10
0
10
EYO

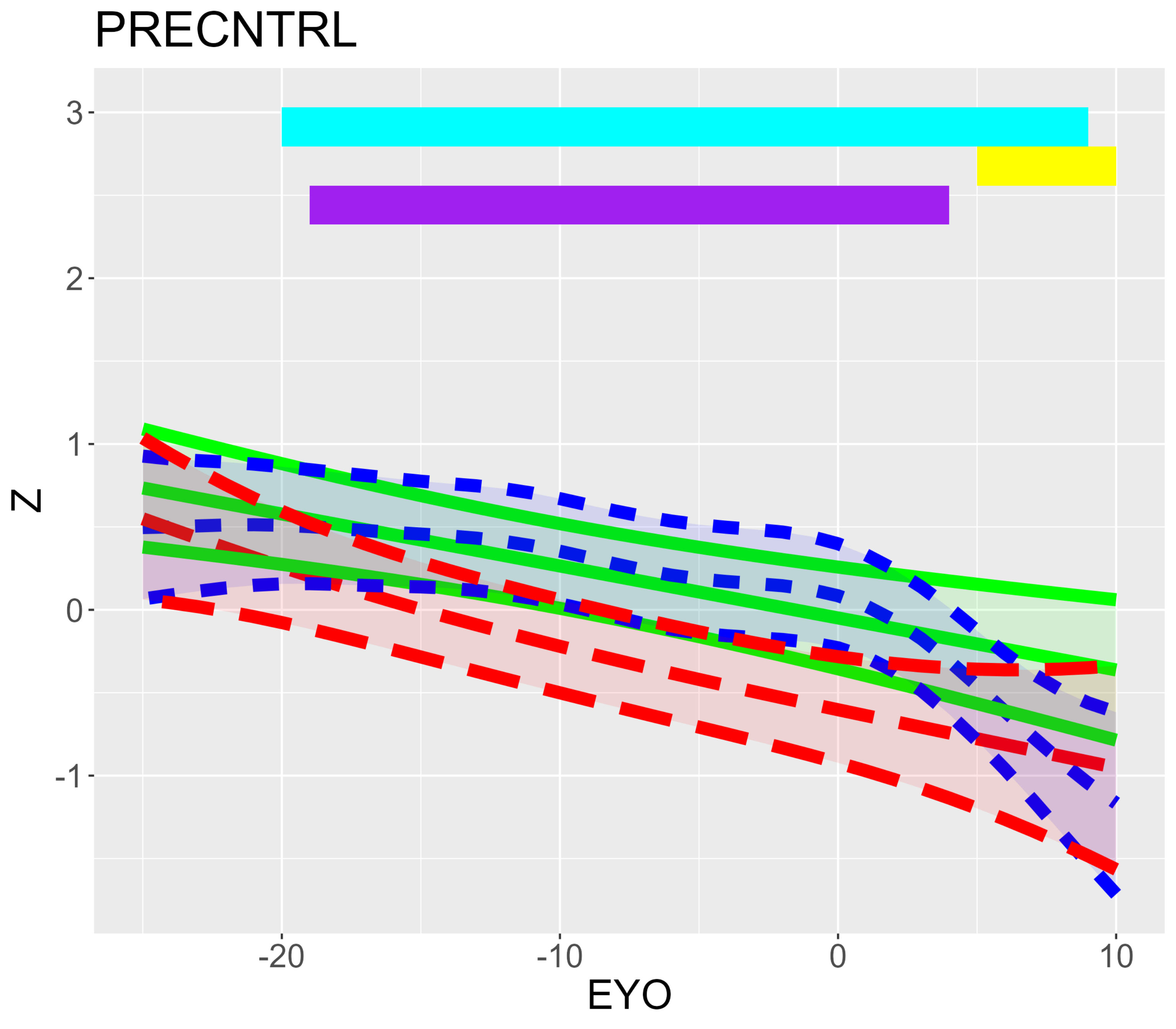
PRECNTRL
3
2
1
0
-1
Z
-20
-10
0
10
EYO

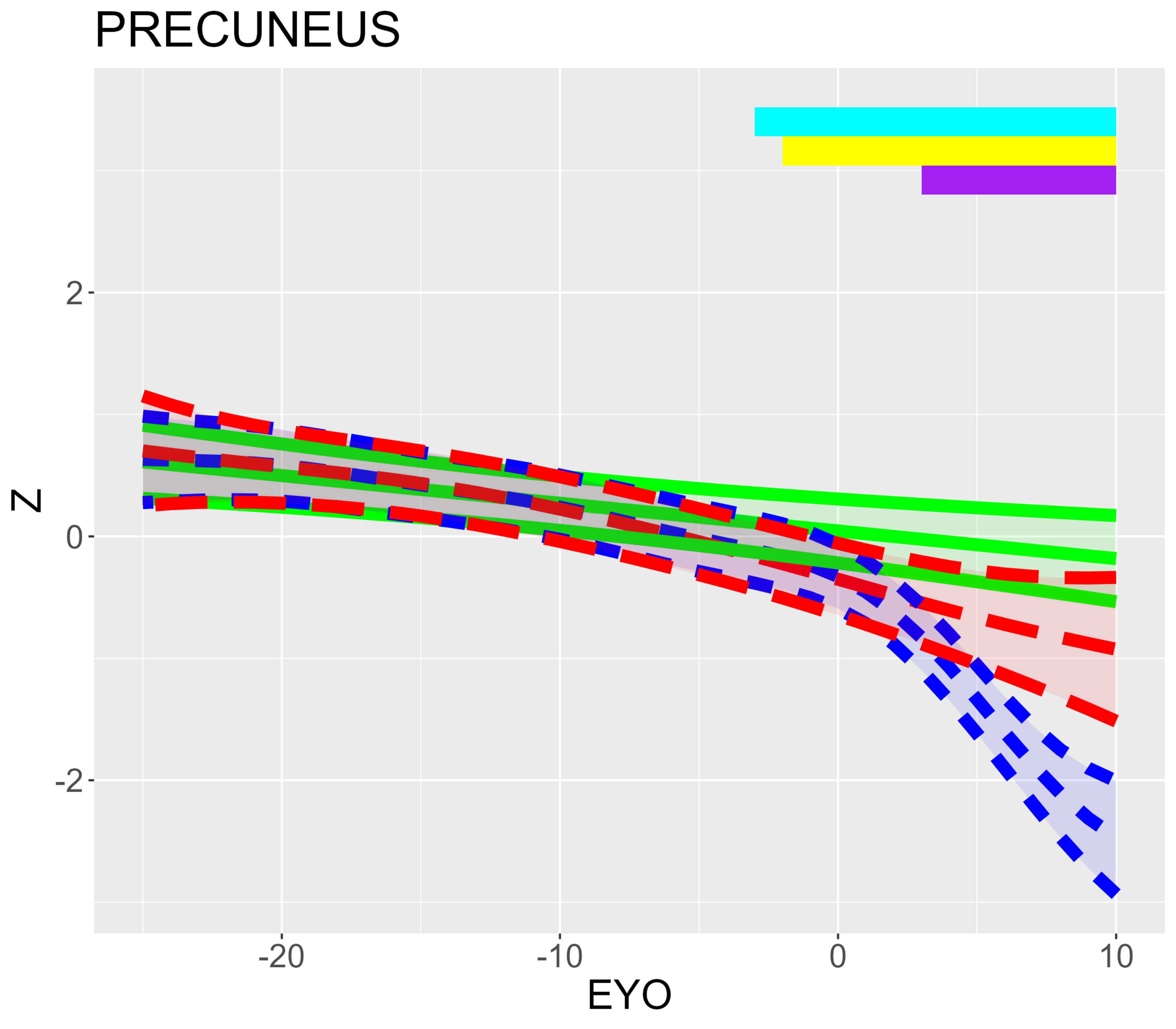
PRECUNEUS
2
0
-2
Z
-20
-10
0
10
EYO

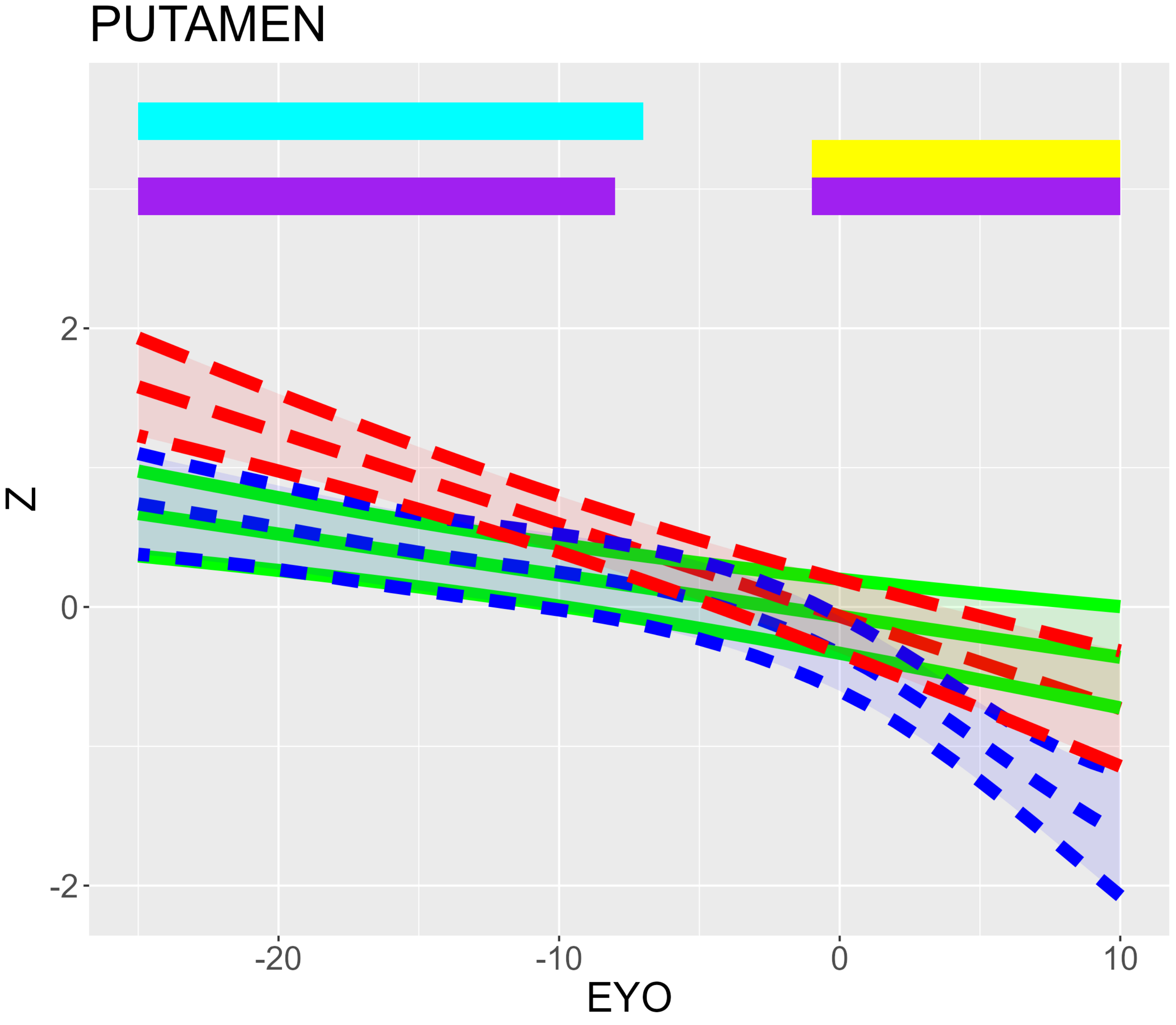

PUTAMEN
2
0
-2
Z
-20
-10
0
10
EYO

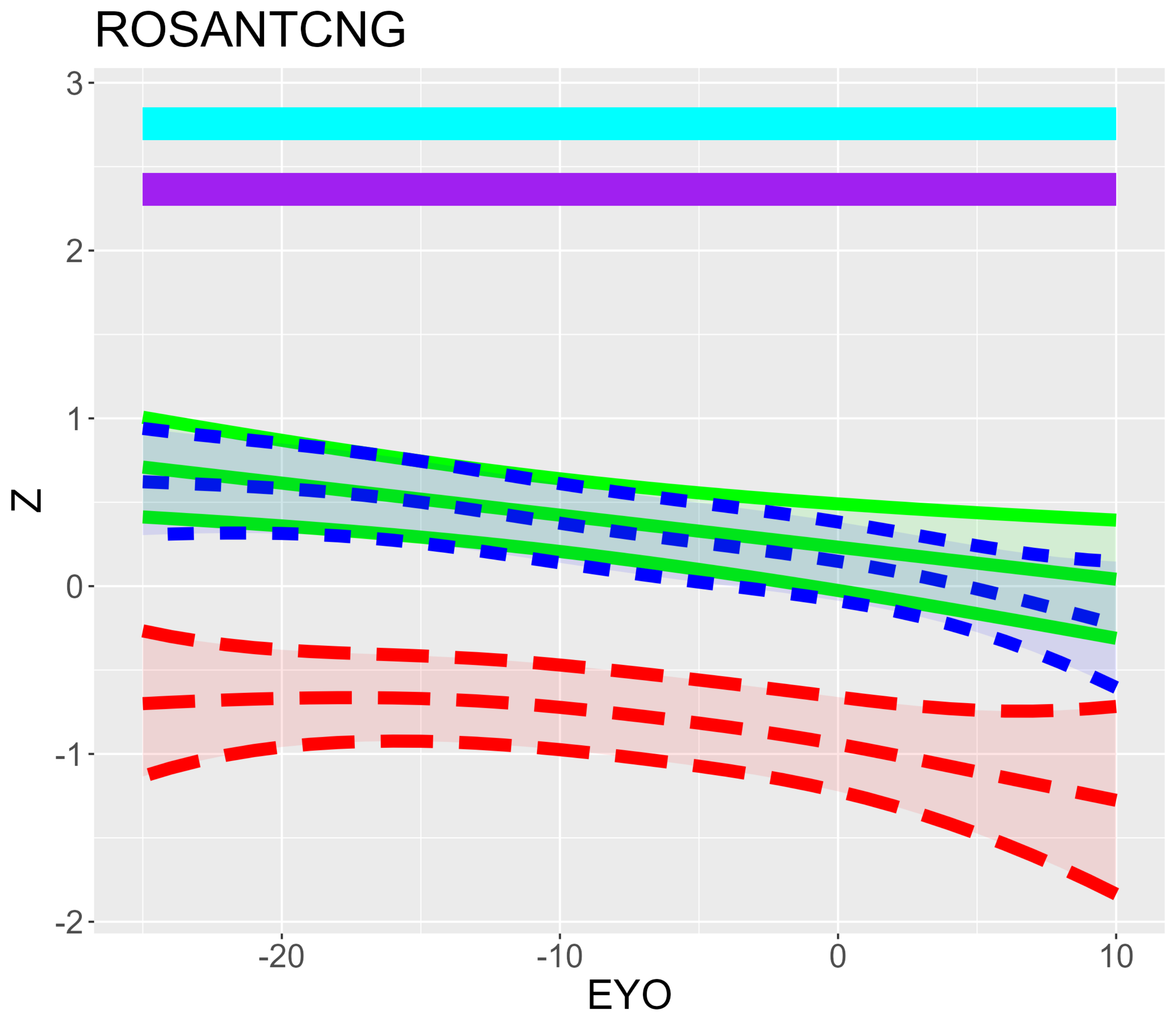
ROSANTCNG
3
2
1
0
-1
-2
Z
-20
-10
0
10
EYO

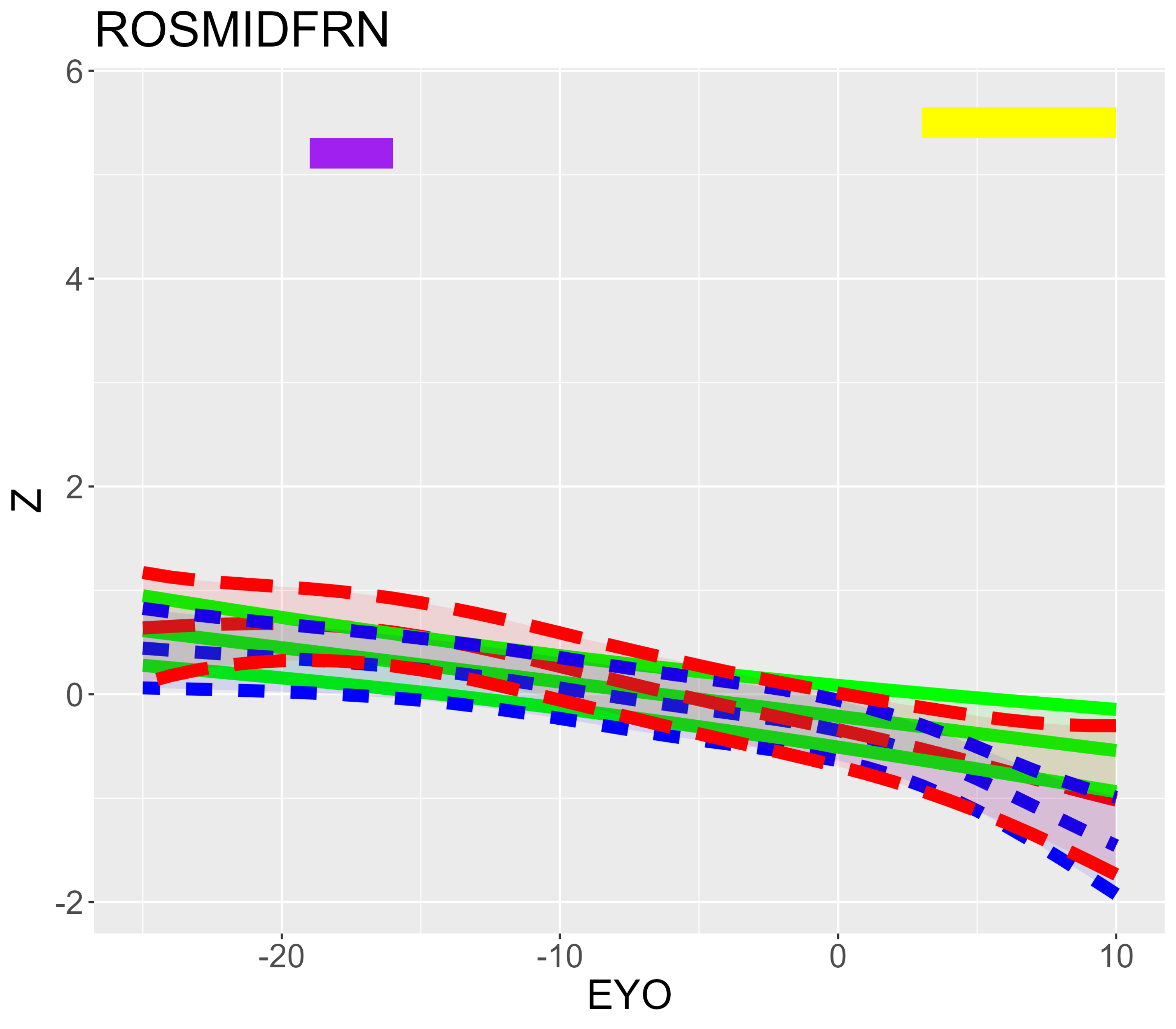
ROSMIDFRN
6
4
2
0
-2
Z
-20
-10
0
10
EYO

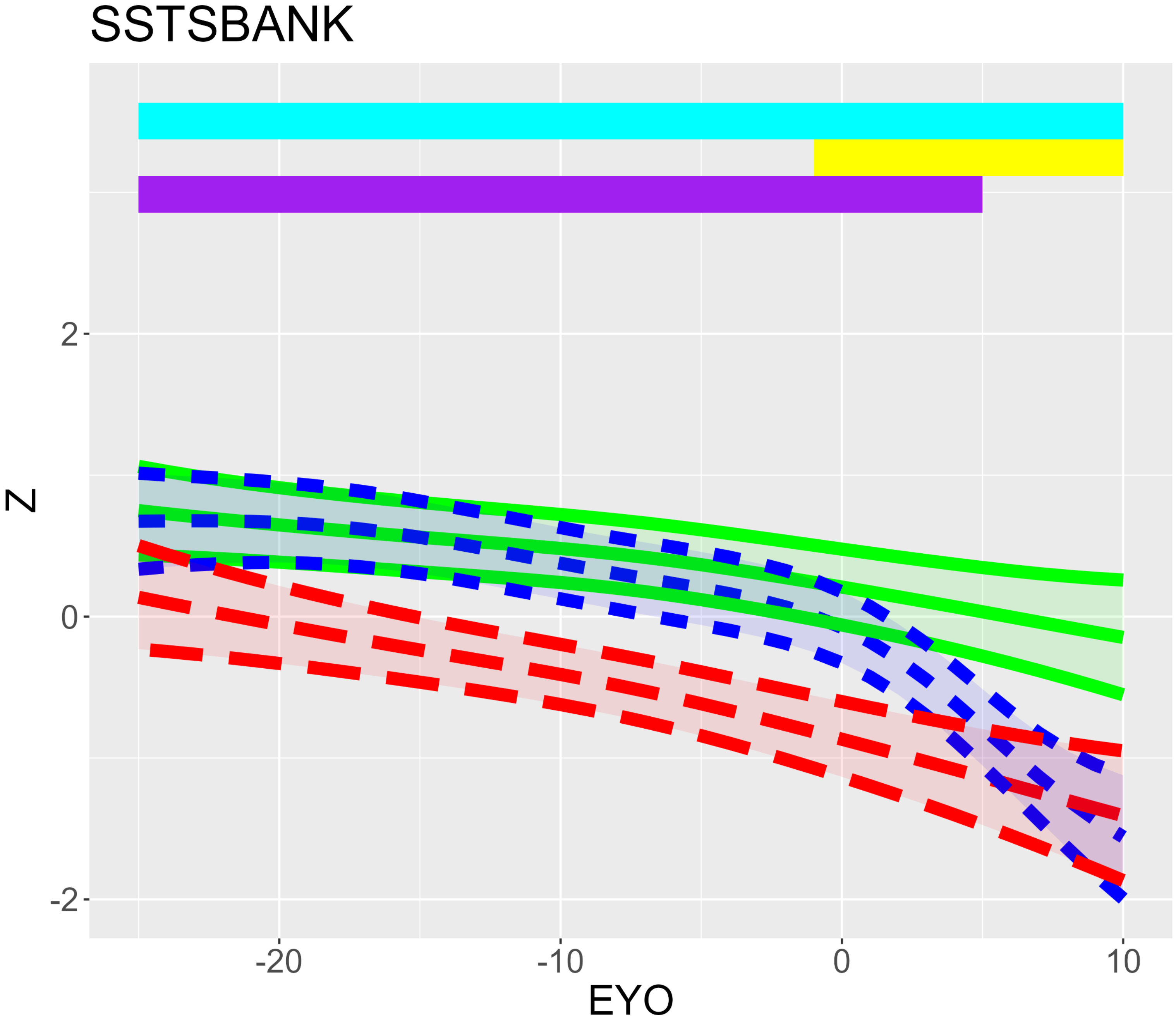
SSTSBANK
2
0
-2
Z
-20
-10
0
10
EYO

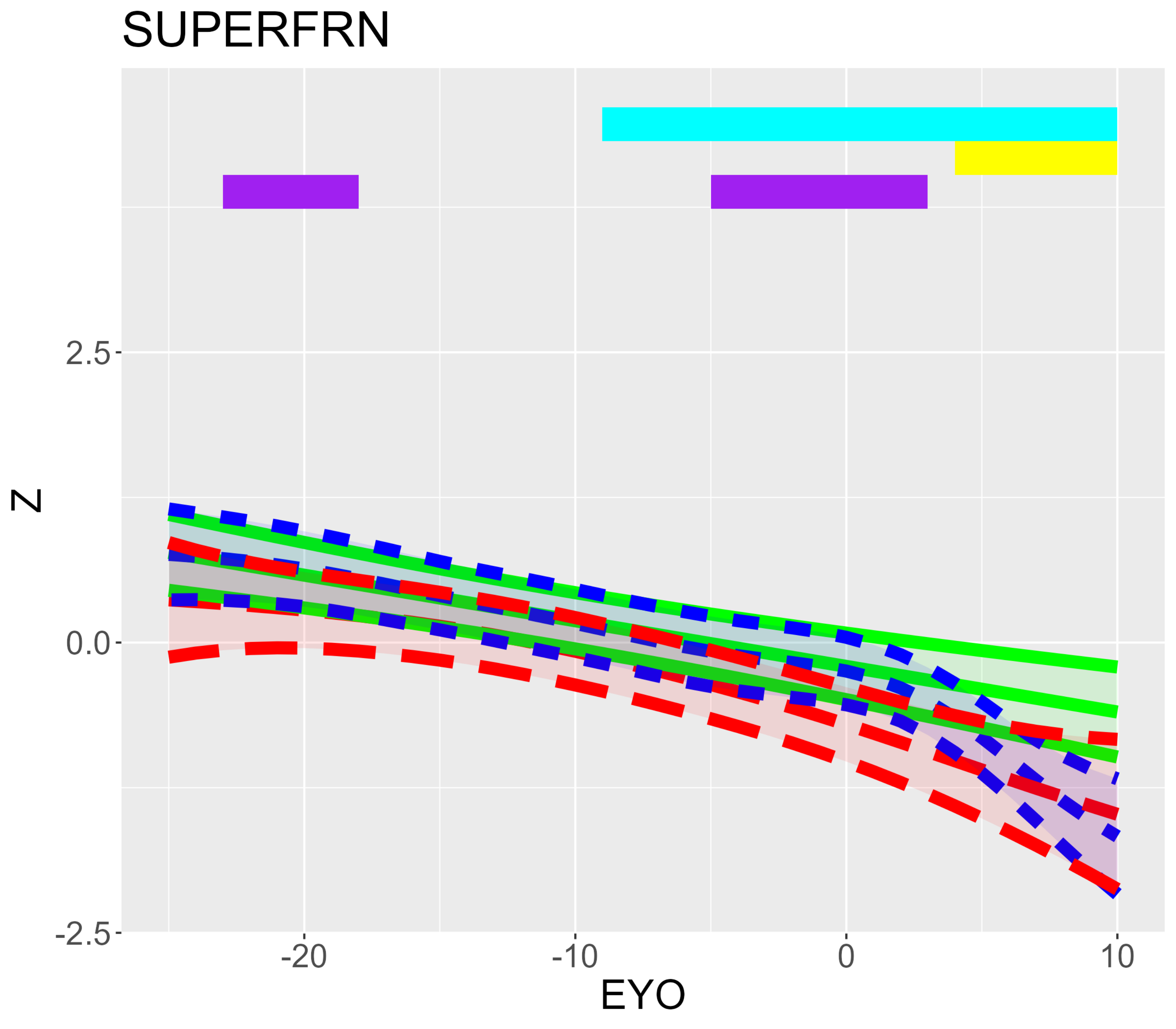
SUPERFRN
Z
2.5
0.0
-2.5
-20
-10
0
10
EYO

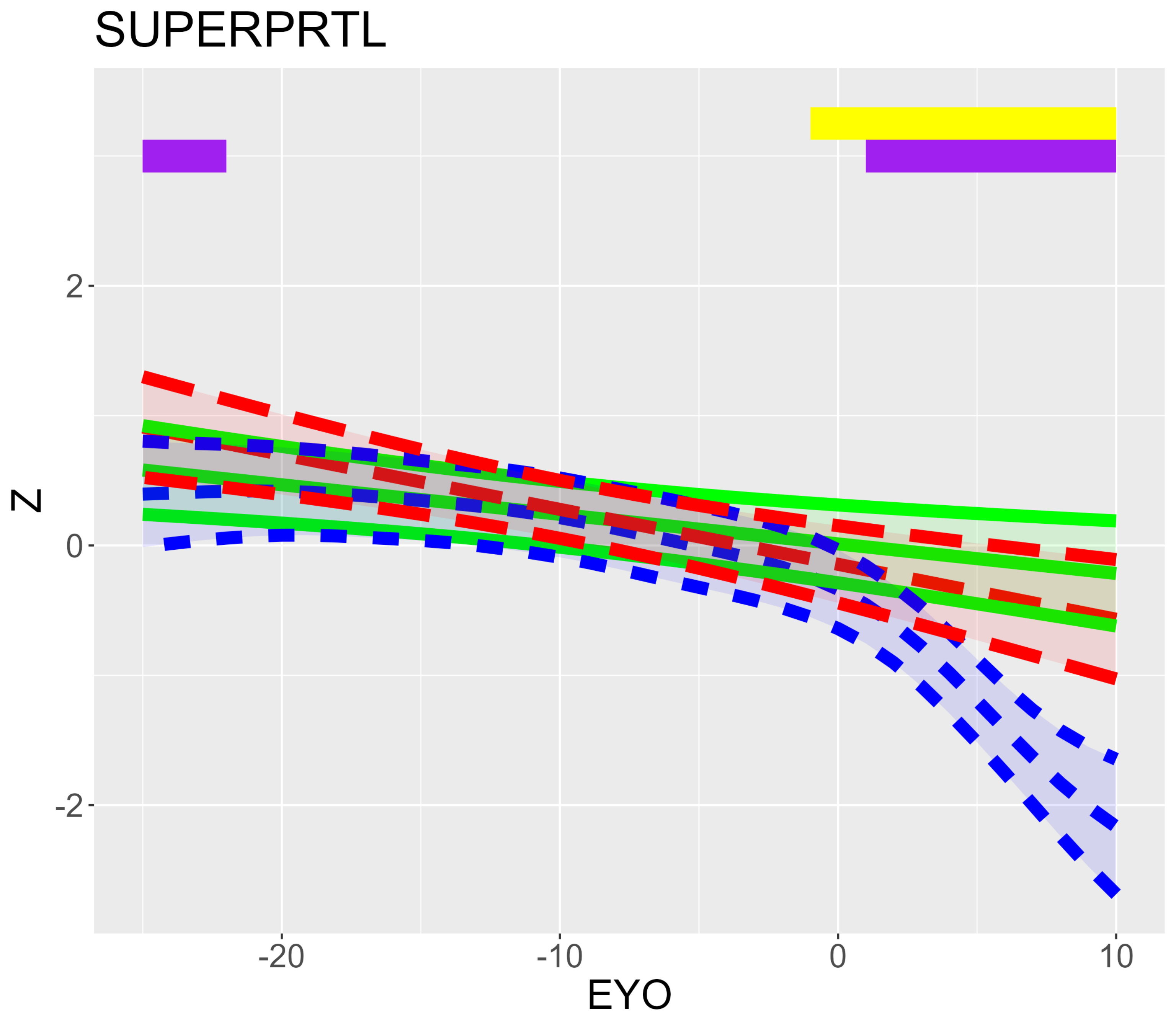
SUPERPRTL
Z
2
0
-2
-20
-10
0
10
EYO

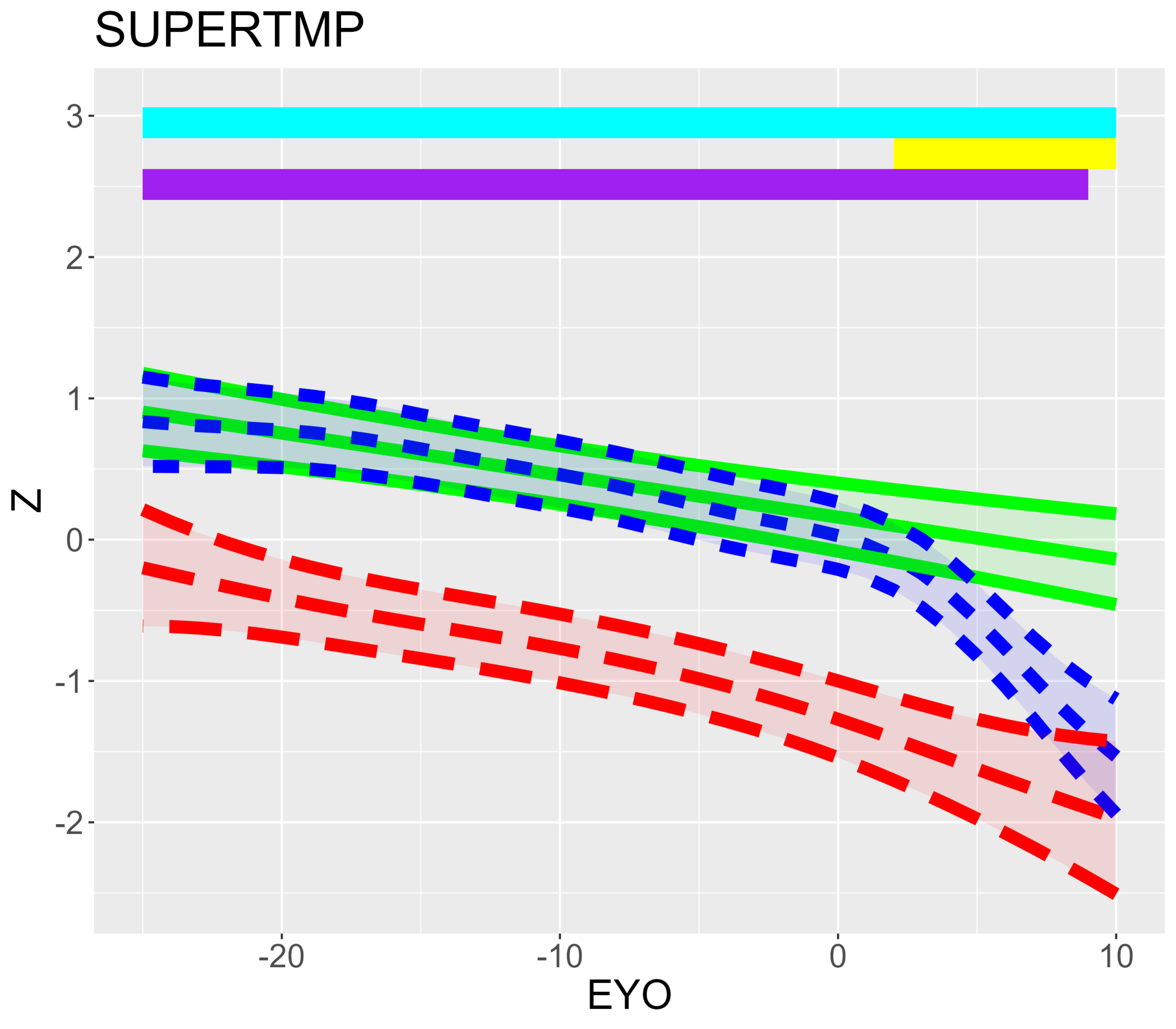
SUPERTMP
3
2
1
0
-1
-2
Z
-20
-10
0
10
EYO

SUPRAMRGNL

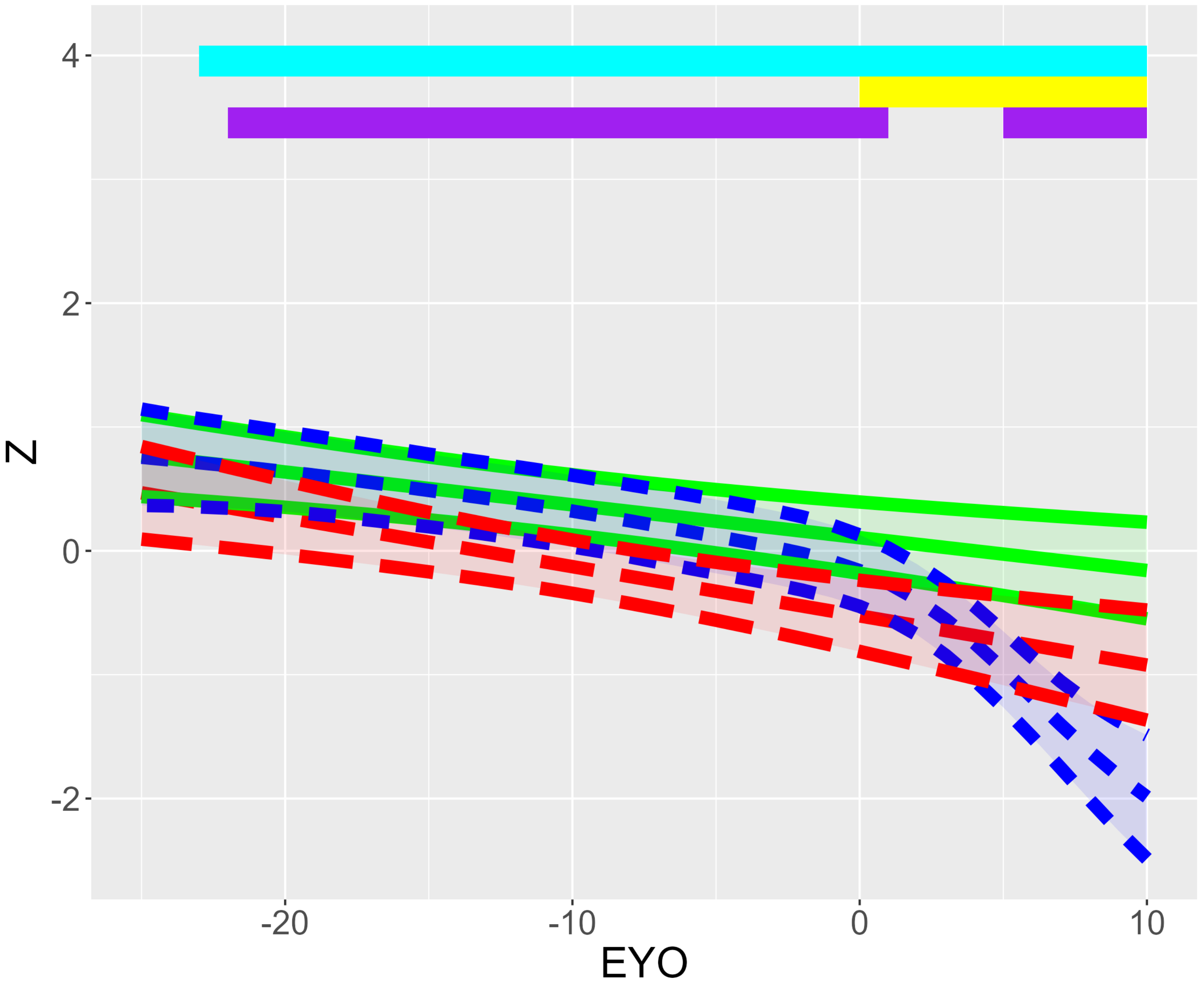

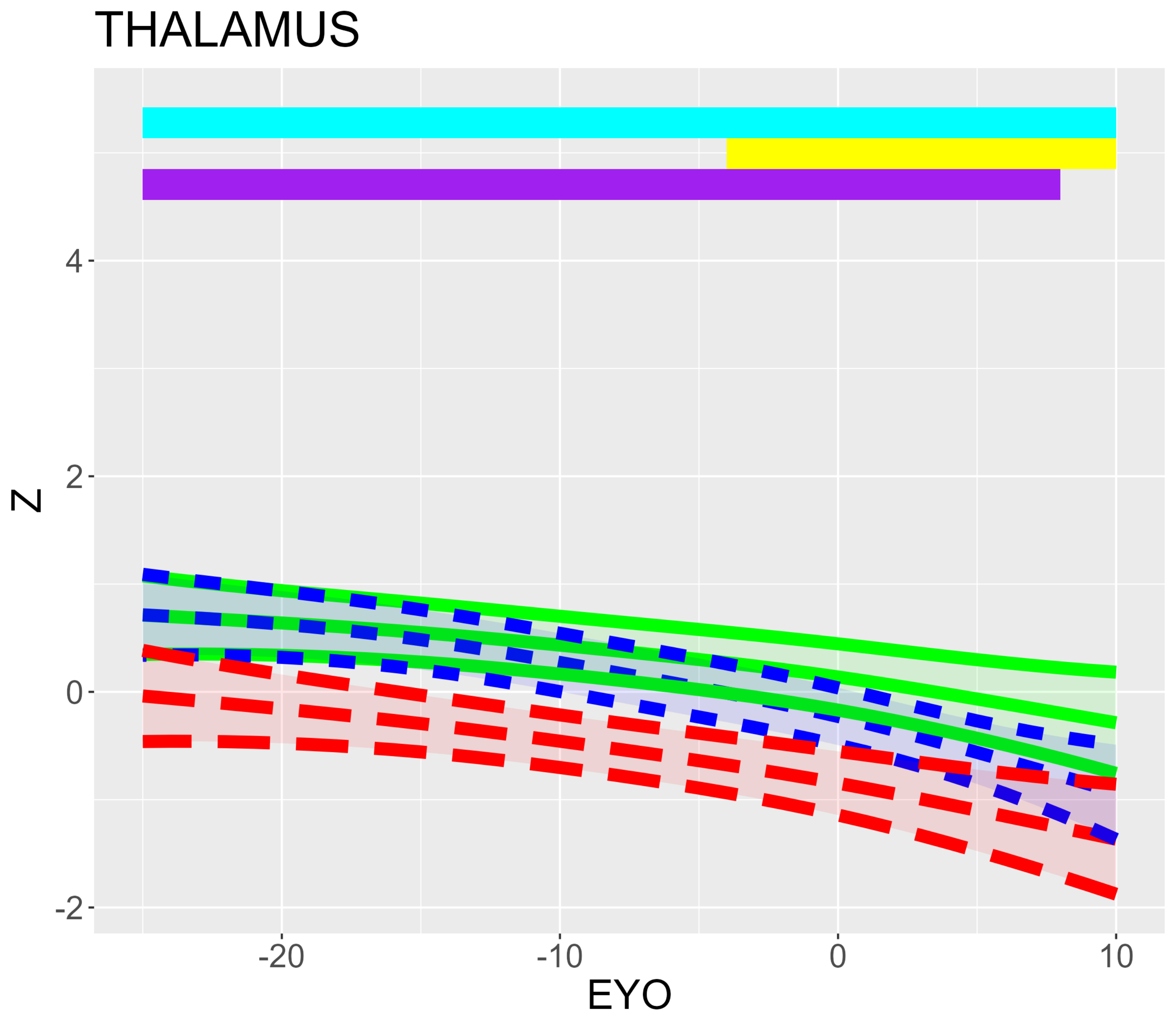
THALAMUS
4
2
0
-2
Z
-20
-10
0
10
EYO

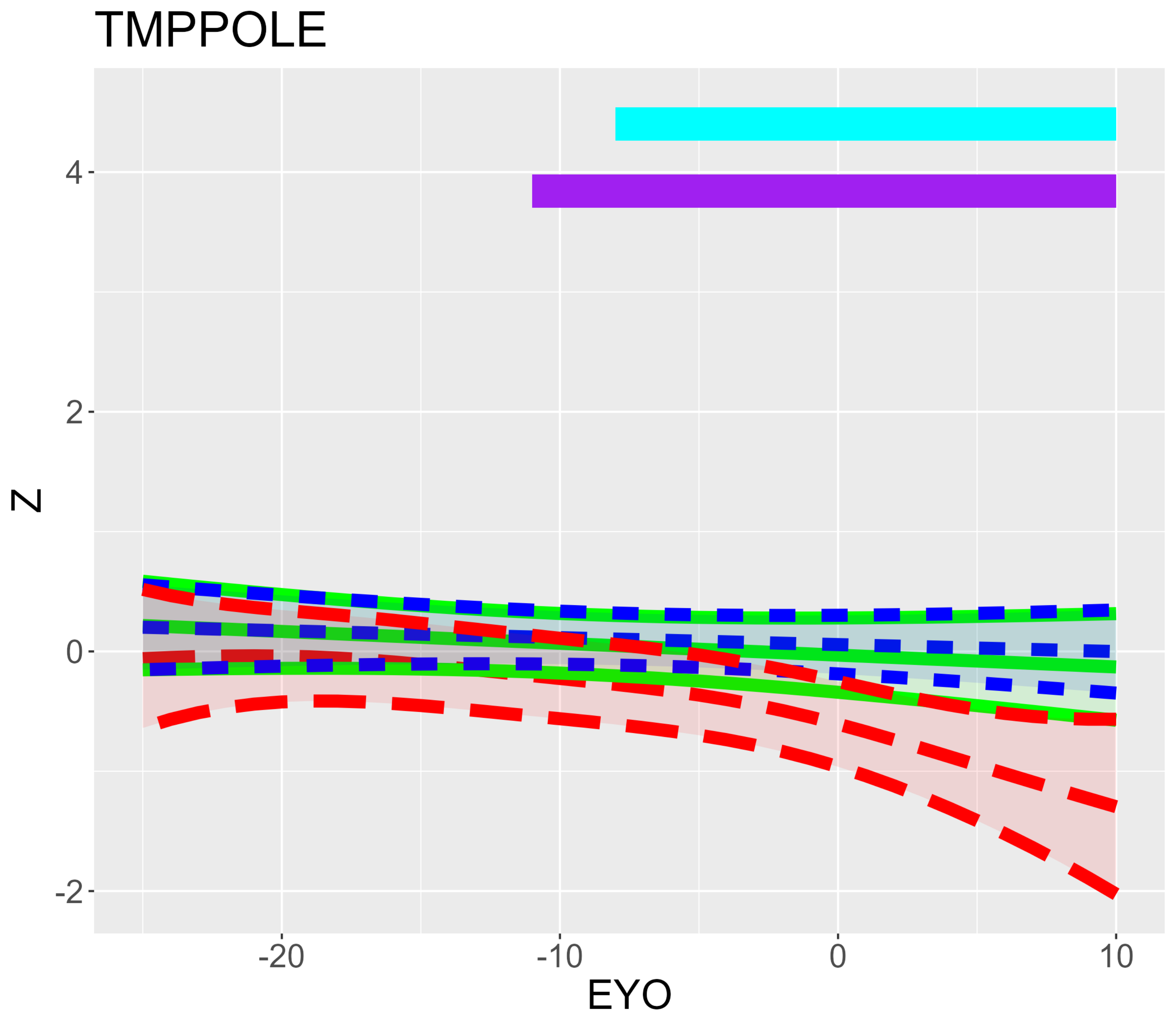
TMPPOLE
4
2
0
-2
Z
-20
-10
0
10
EYO

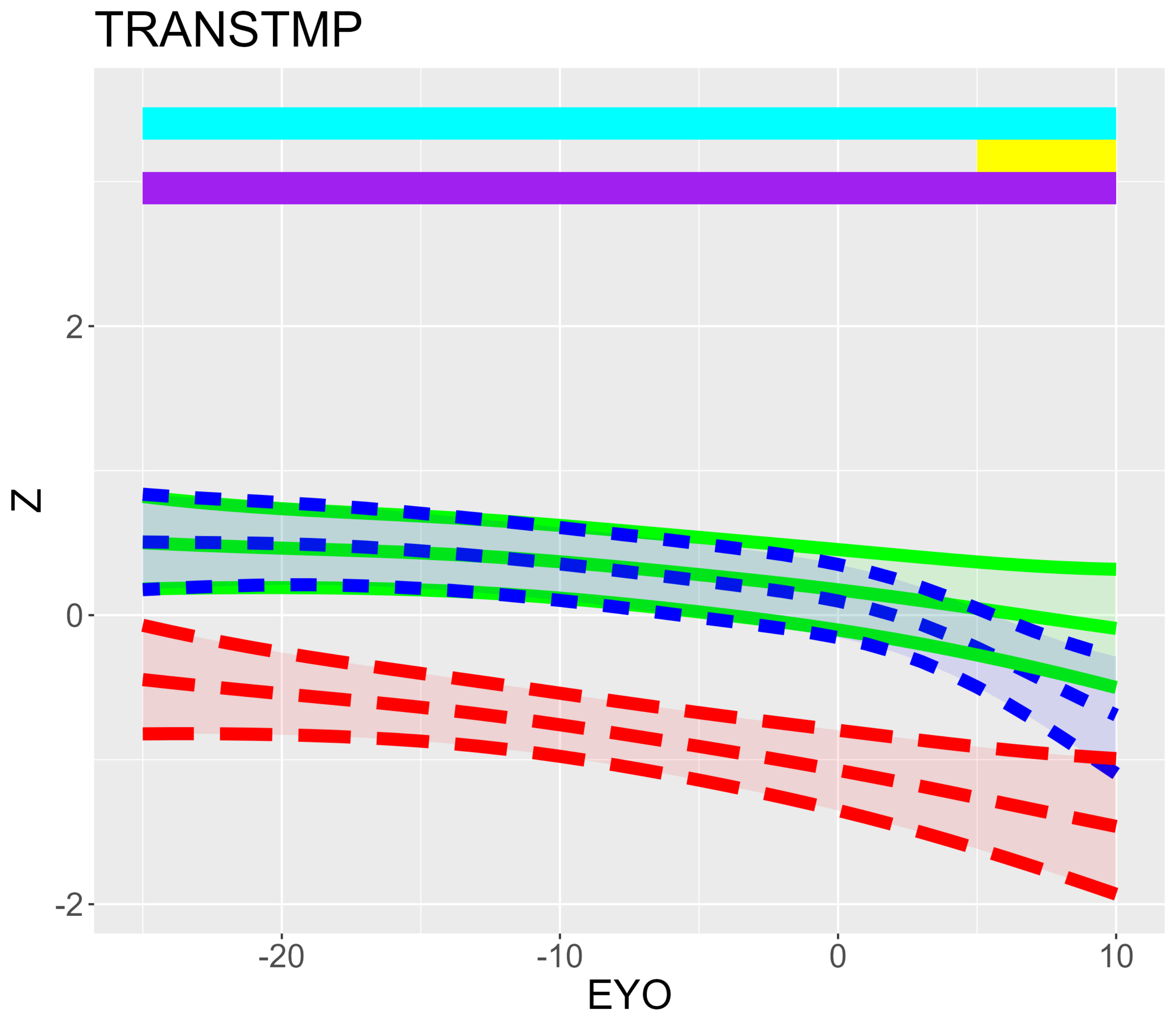

TRANSTMP
Z
2
0
-2
-20
-10
0
10
EYO

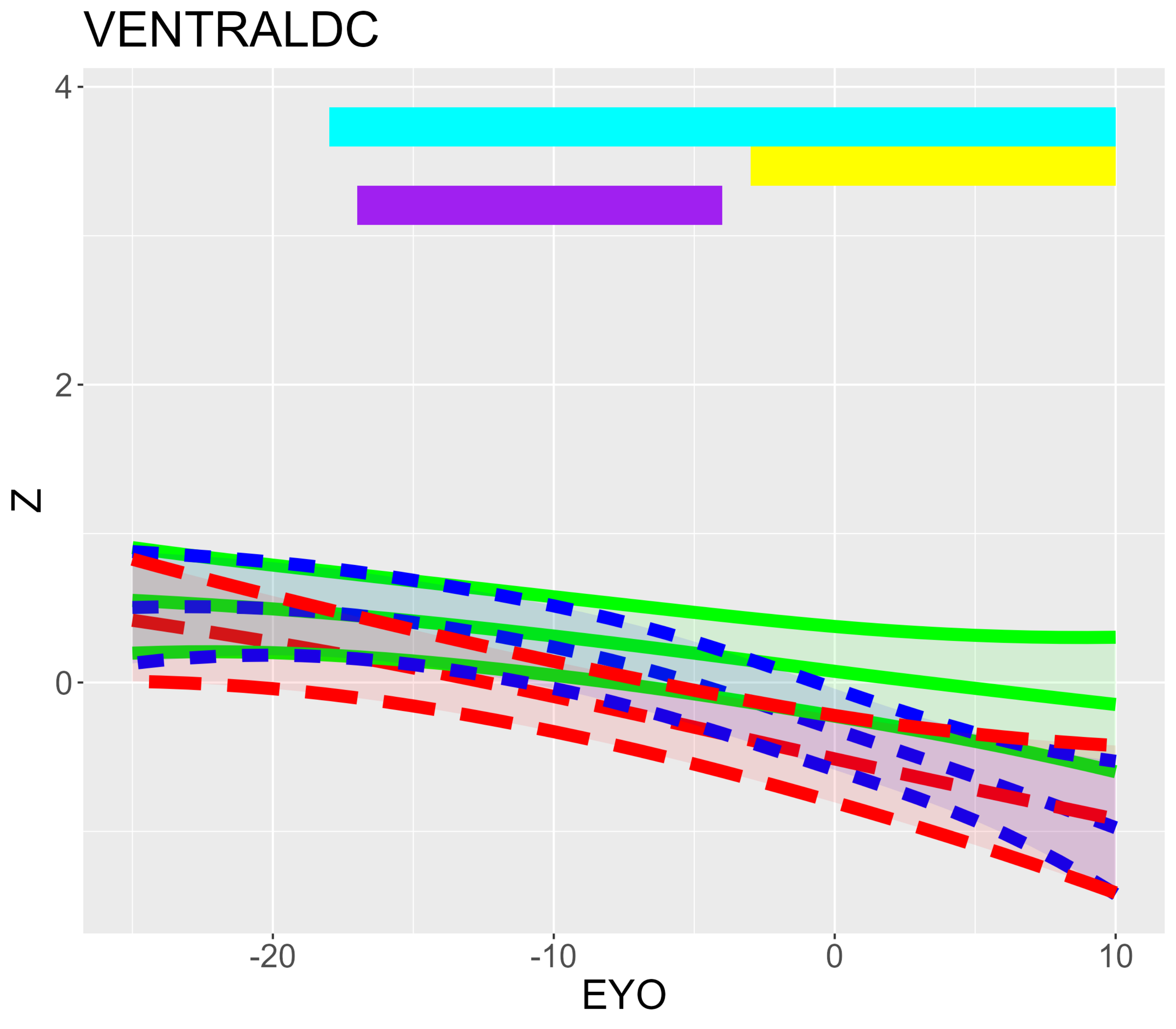

VENTRALDC
4
2
0
Z
-20
-10
0
10
EYO
