# Supplementary material for: Brain volume trajectories in Down syndrome and autosomal dominant Alzheimer's disease
Source: Alzheimers Dement. 2026 Jan 18;22(1):e71103. doi: 10.1002/alz.71103 (PMC12812856; doi:10.1002/alz.71103)
Supplement: Supplementary file 5 — Supporting information [file ALZ-22-e71103-s018.pdf]

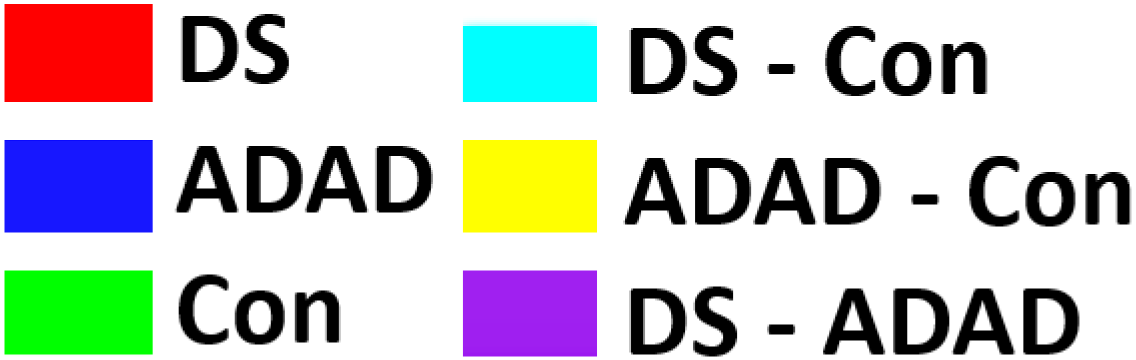
DS
ADAD
Con
DS - Con
ADAD - Con
DS - ADAD

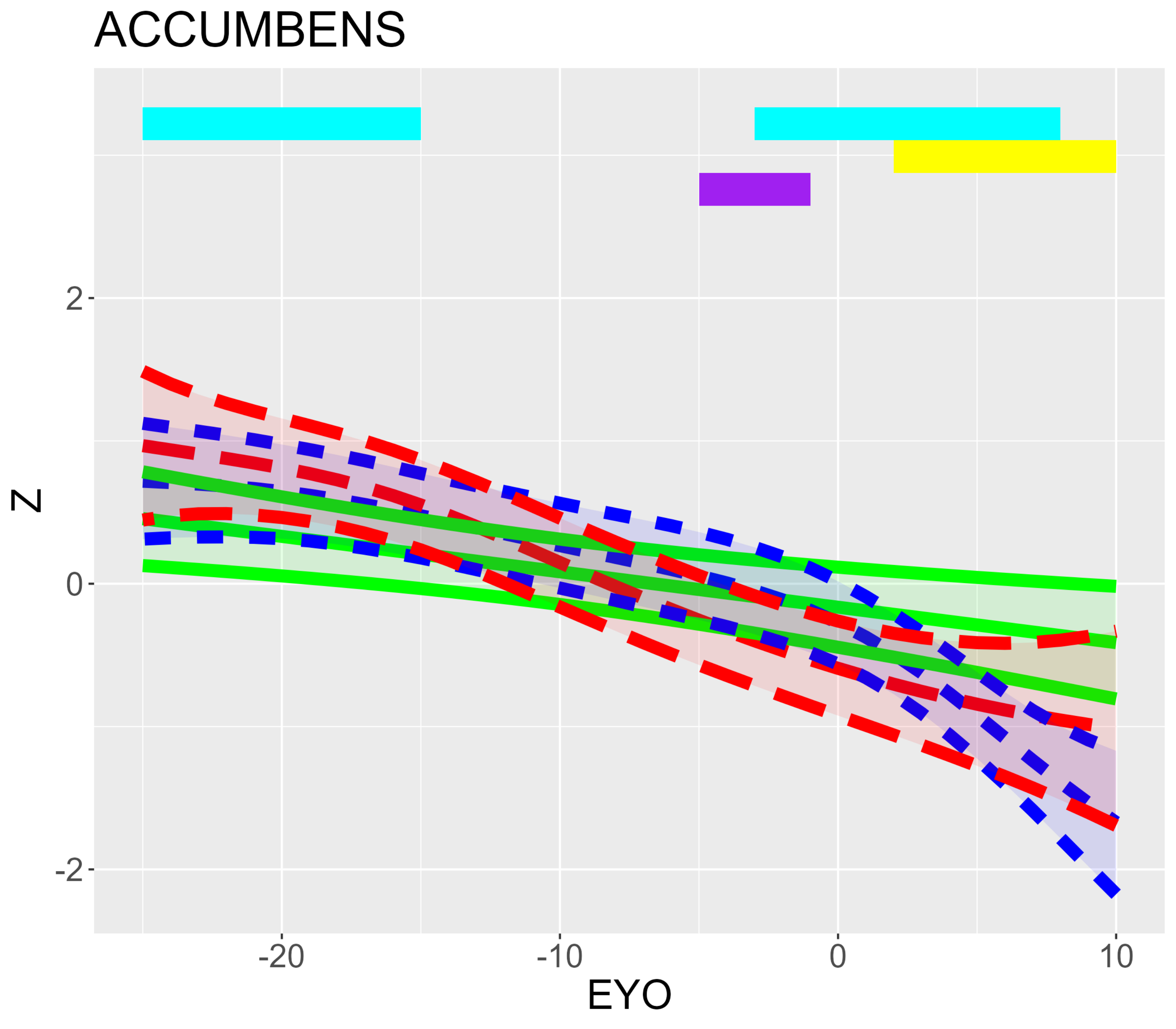

ACCUMBENS
2
0
-2
Z
-20
-10
0
10
EYO

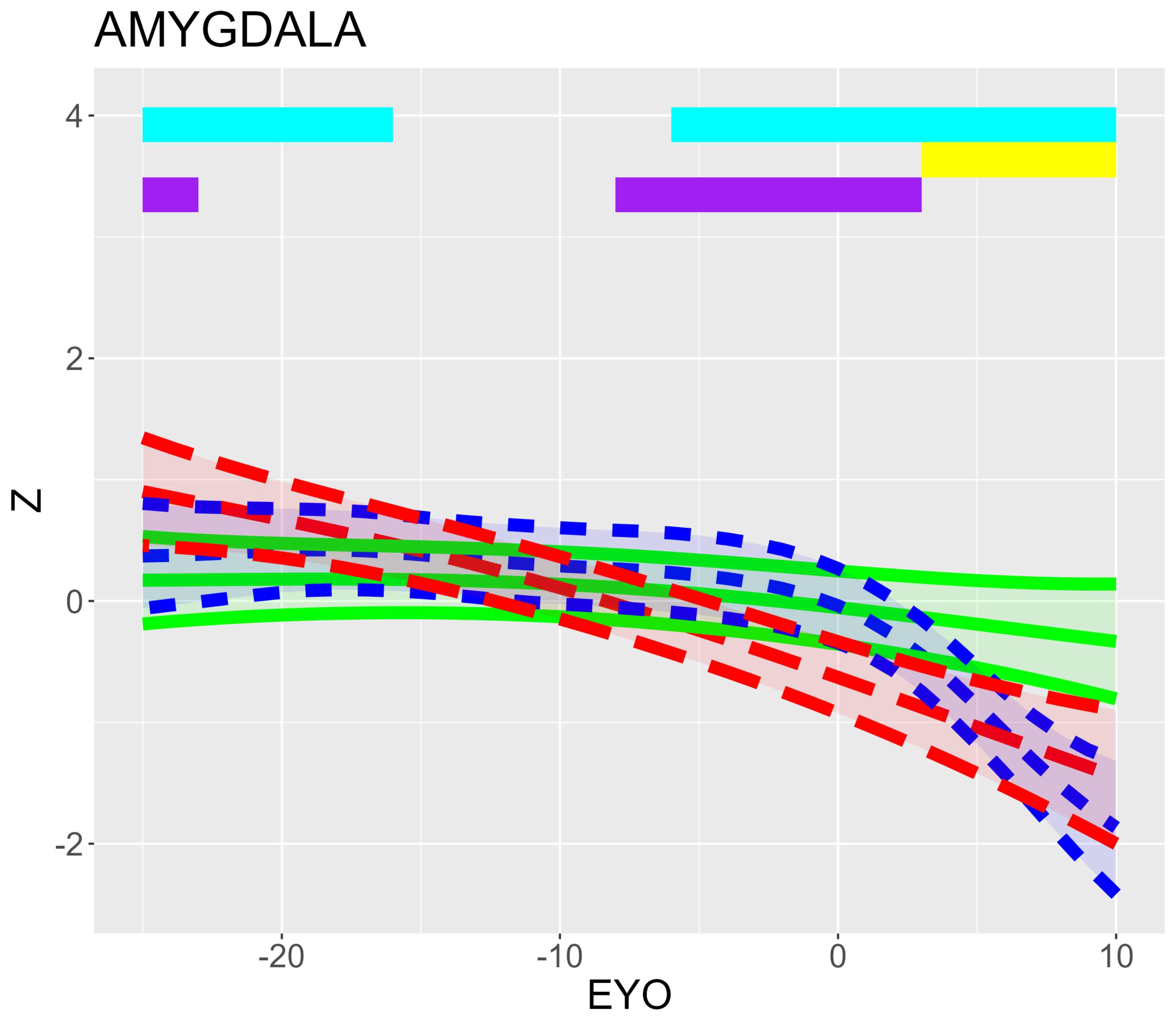
AMYGDALA
4
2
0
-2
Z
-20
-10
0
10
EYO

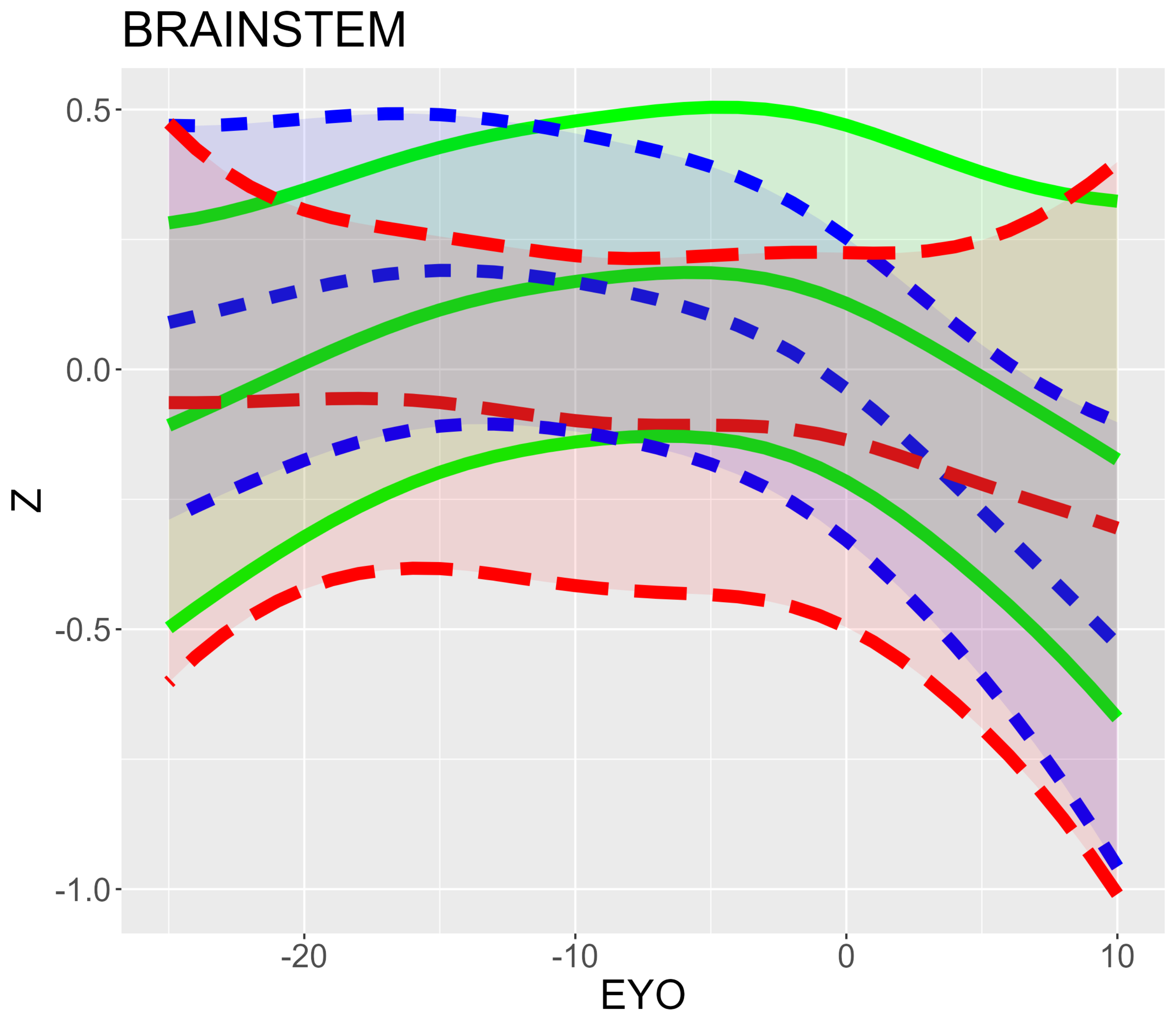
BRAINSTEM
0.5
0.0
-0.5
-1.0
Z
-20
-10
0
10
EYO

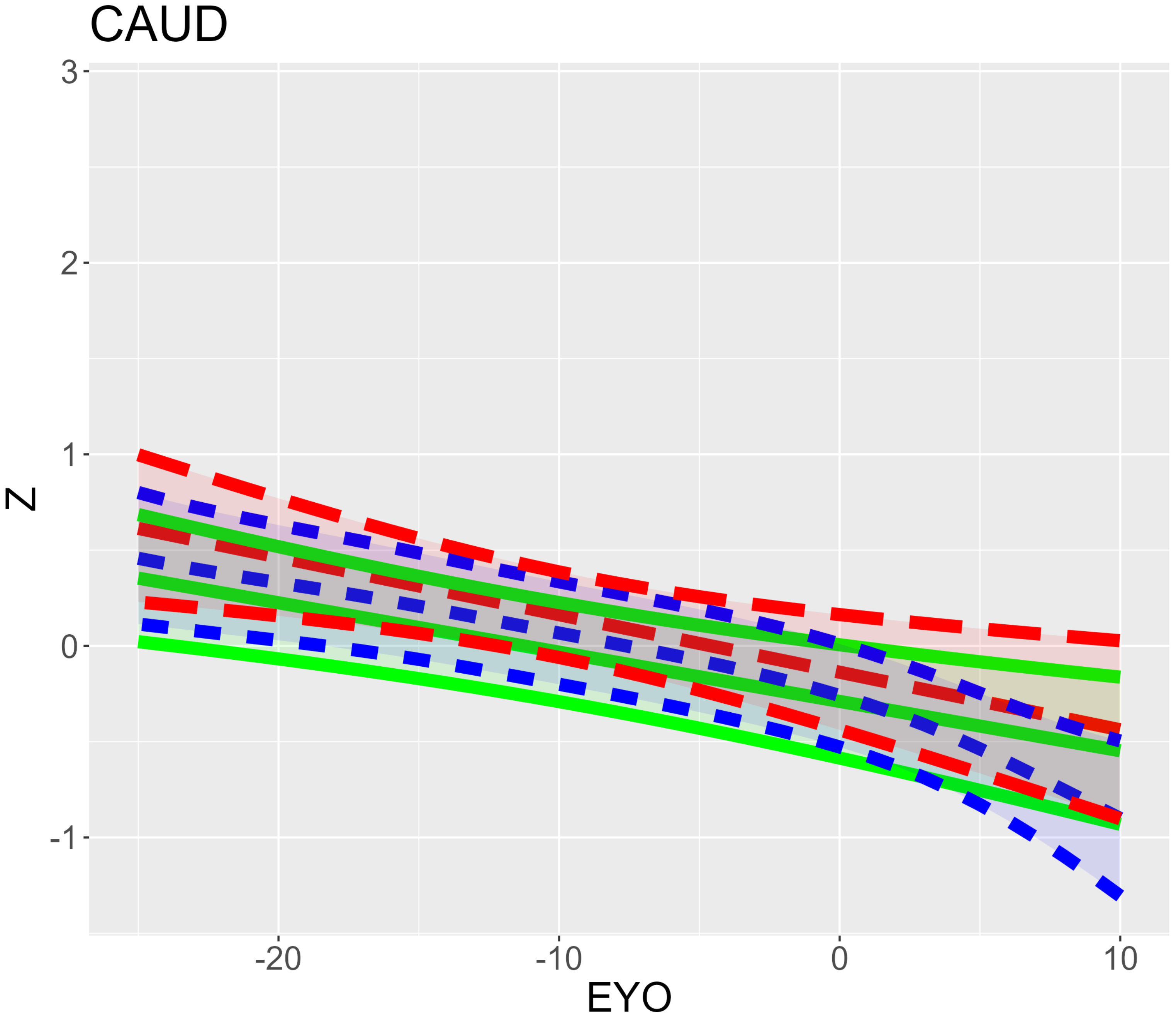
CAUD
3
2
1
0
-1
Z
-20
-10
0
10
EYO

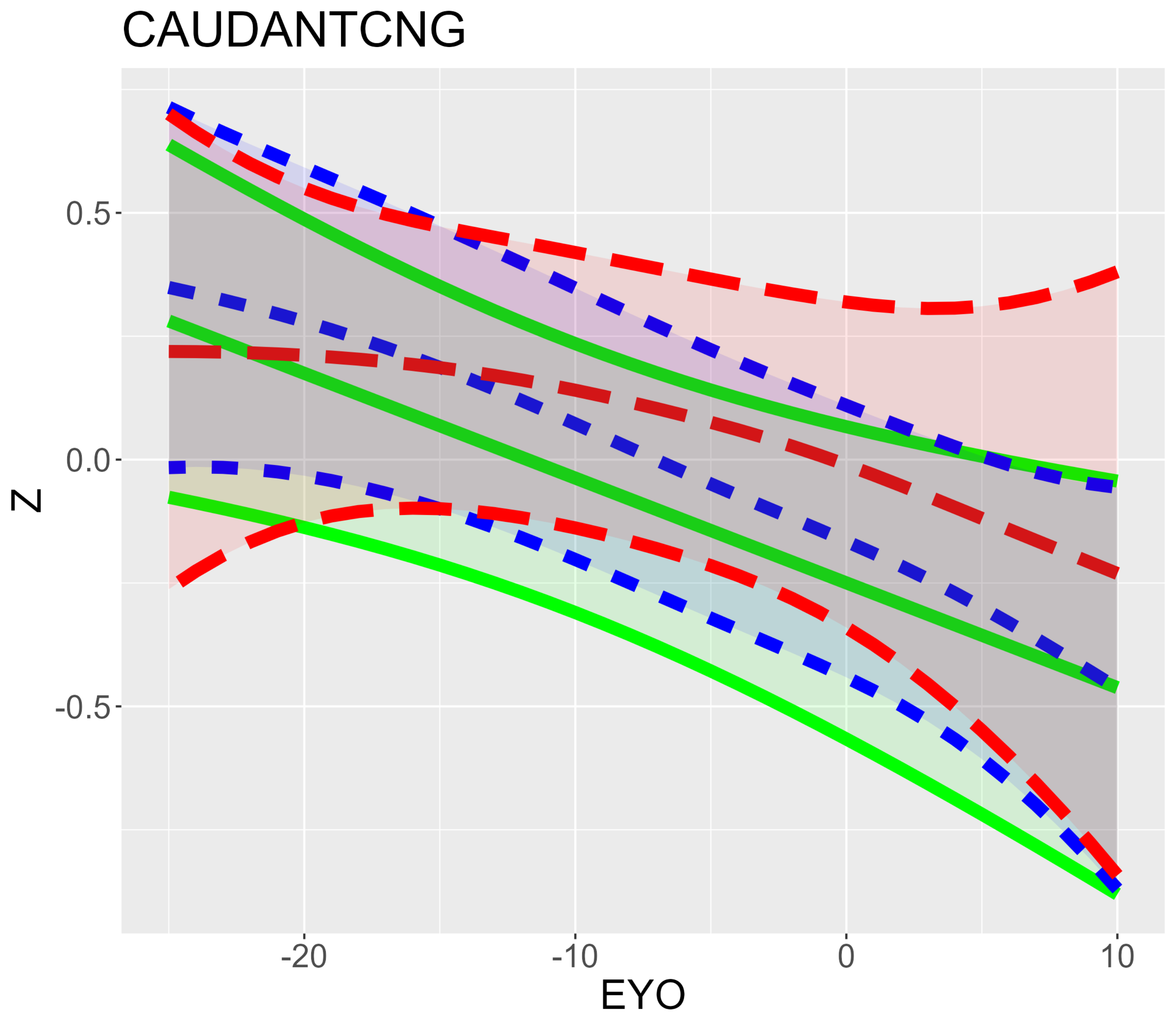
CAUDANTCNG
0.5
0.0
-0.5
Z
-20
-10
0
10
EYO

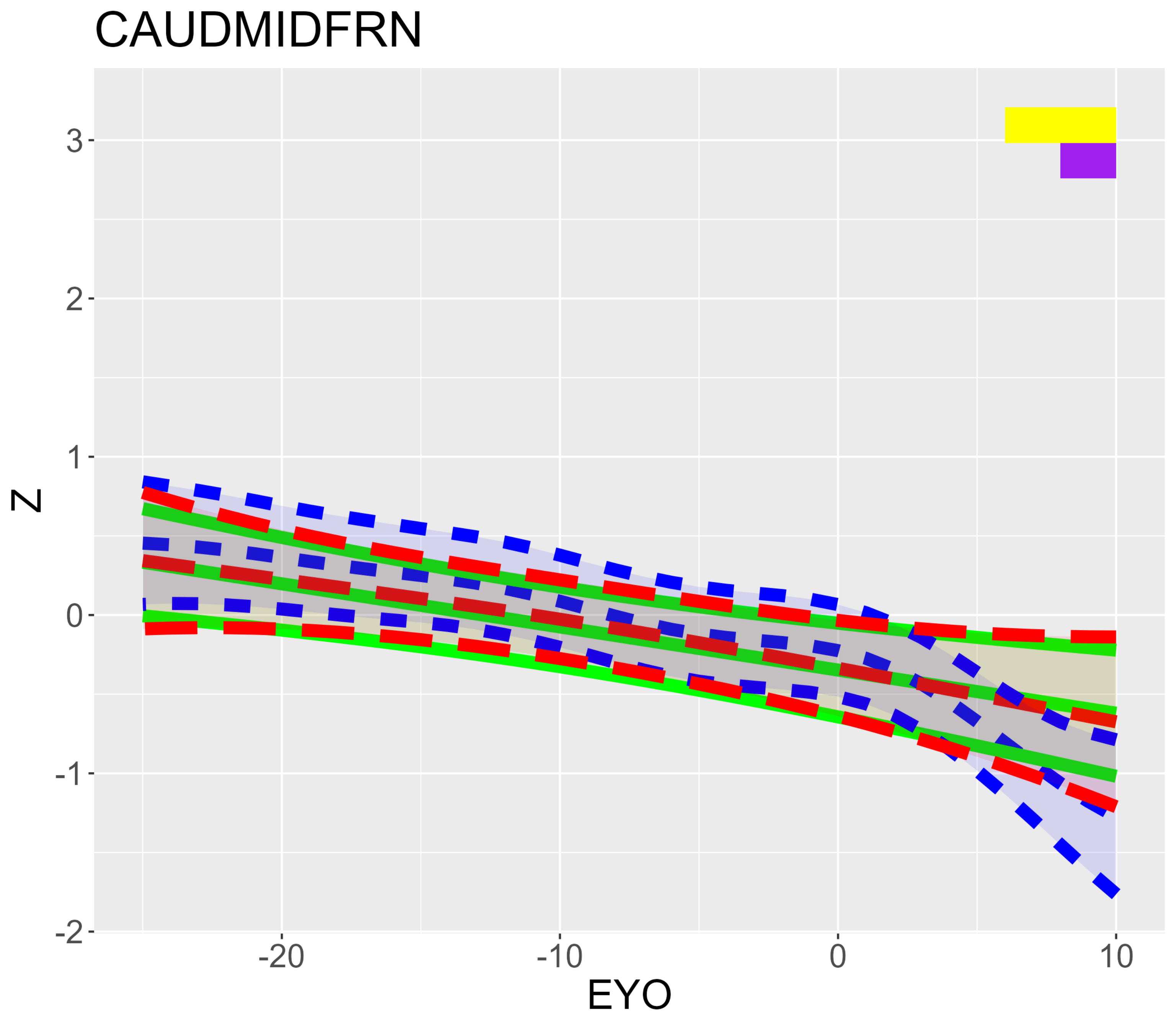
CAUDMIDFRN
Z
EYO
3
2
1
0
-1
-2
-20
-10
0
10

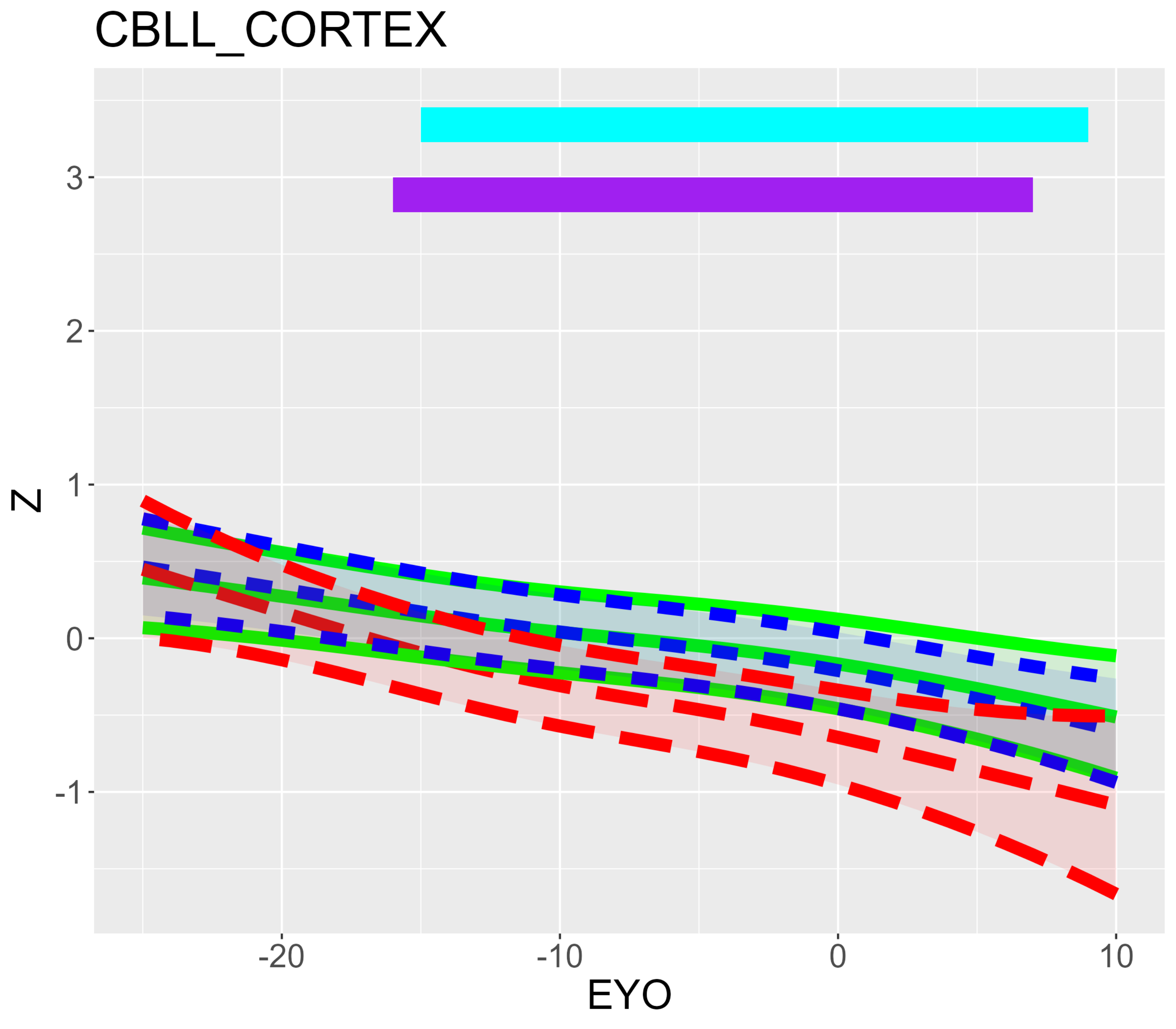
CBLL_CORTEX
3
2
1
0
-1
Z
-20
-10
0
10
EYO

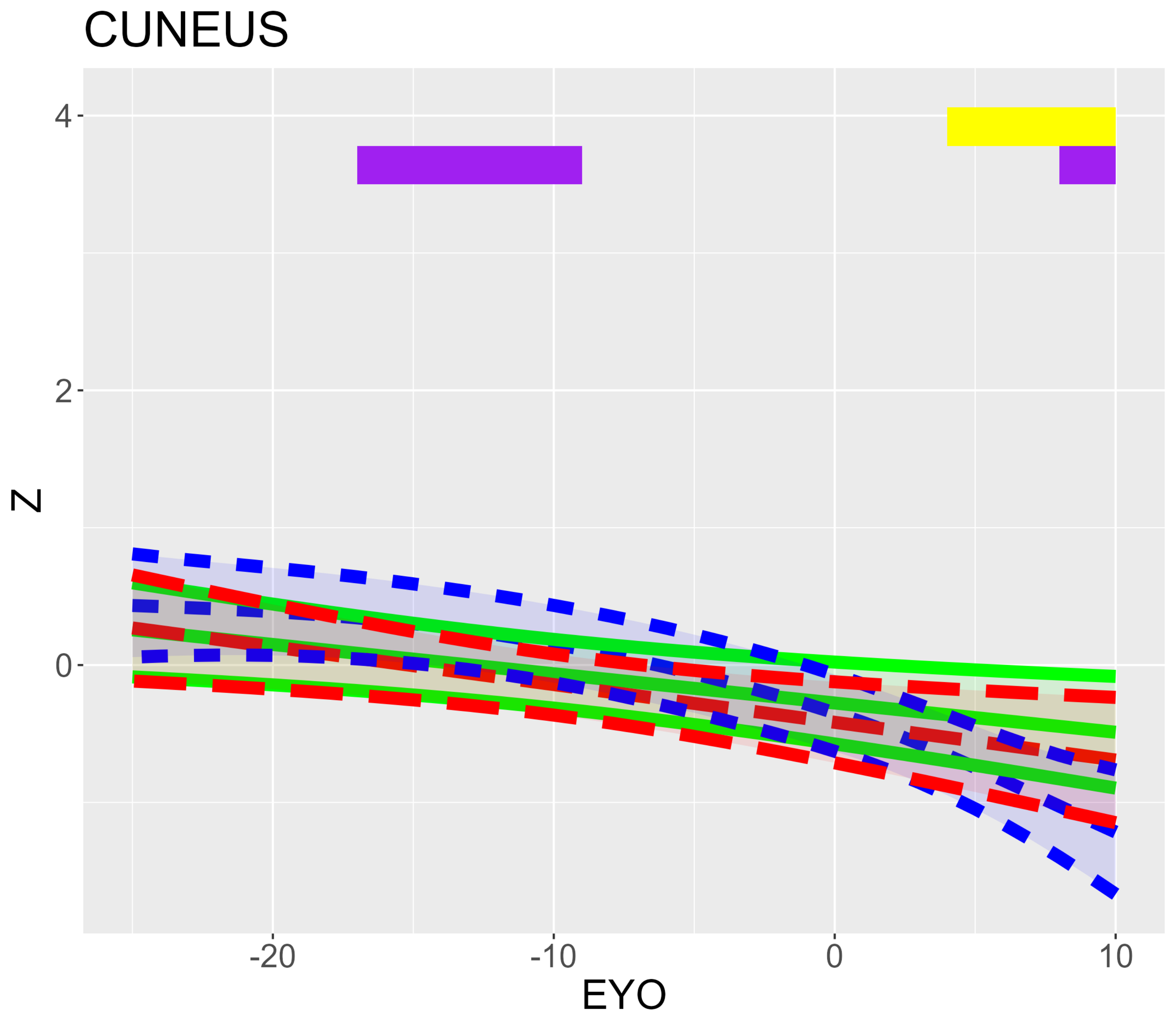

CUNEUS
4
2
0
-20
-10
0
10
Z
EYO

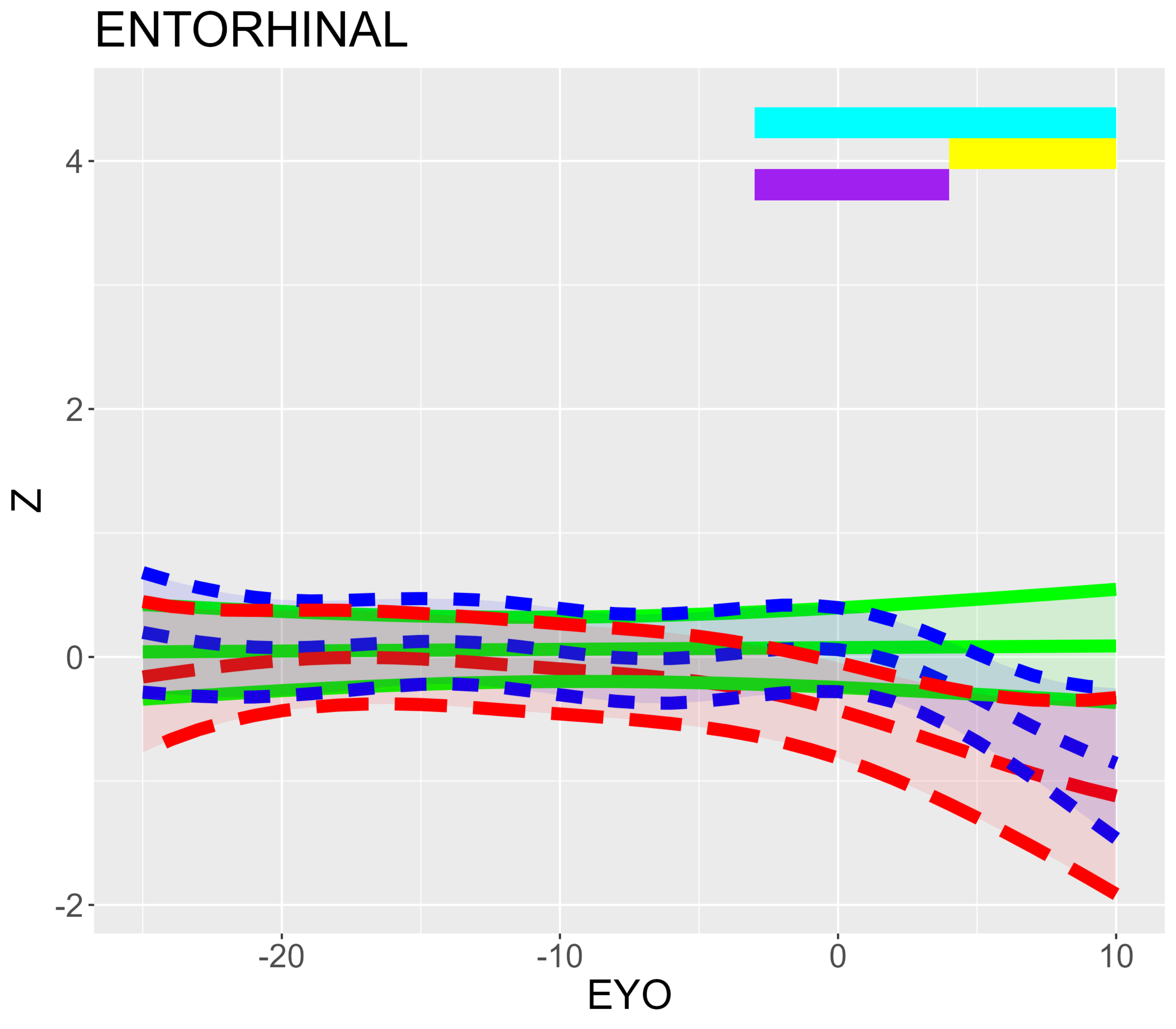
ENTORHINAL
4
2
0
-2
Z
-20
-10
0
10
EYO

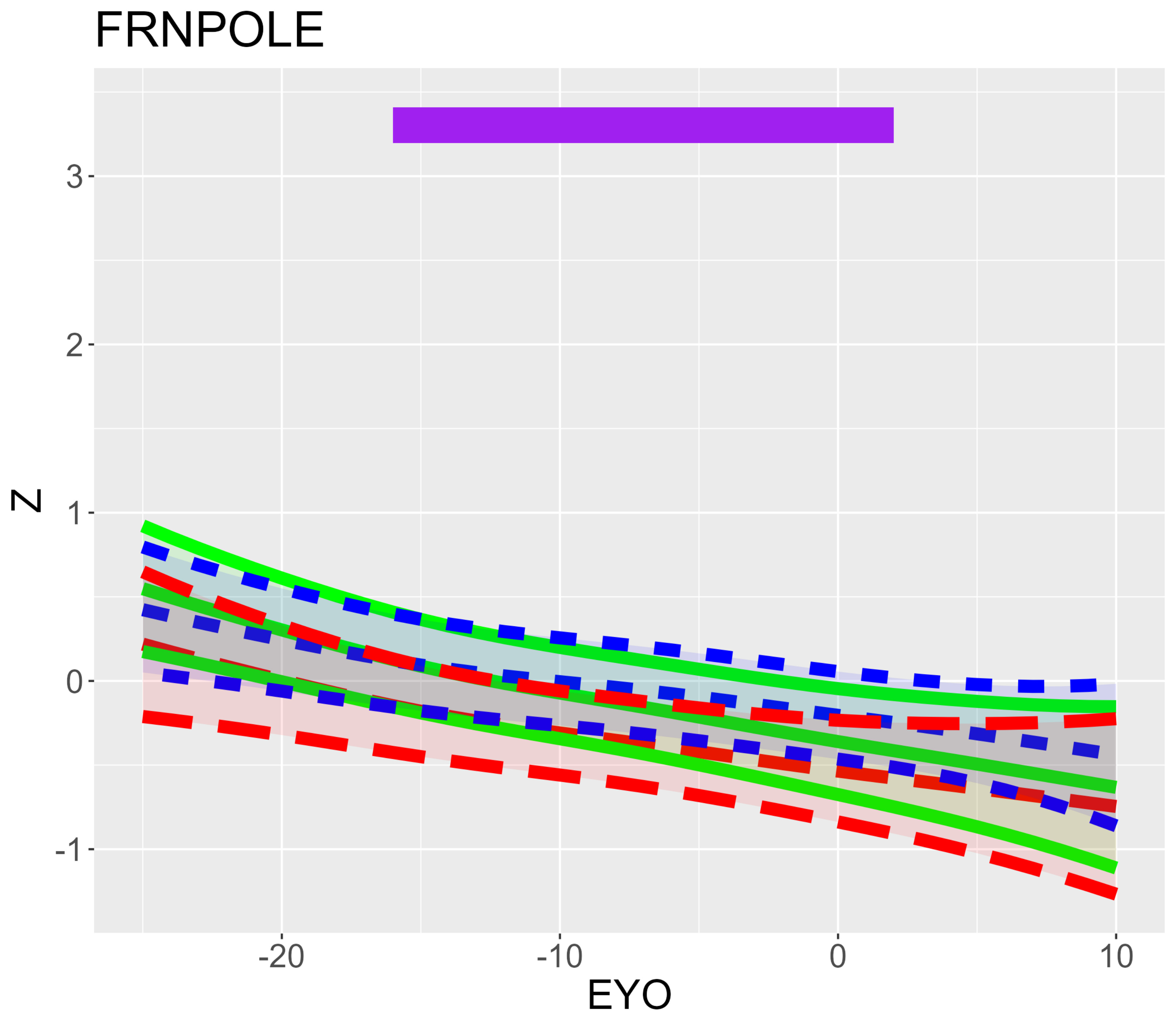
FRNPOLE
Z
EYO
3
2
1
0
-1
-20
-10
0
10

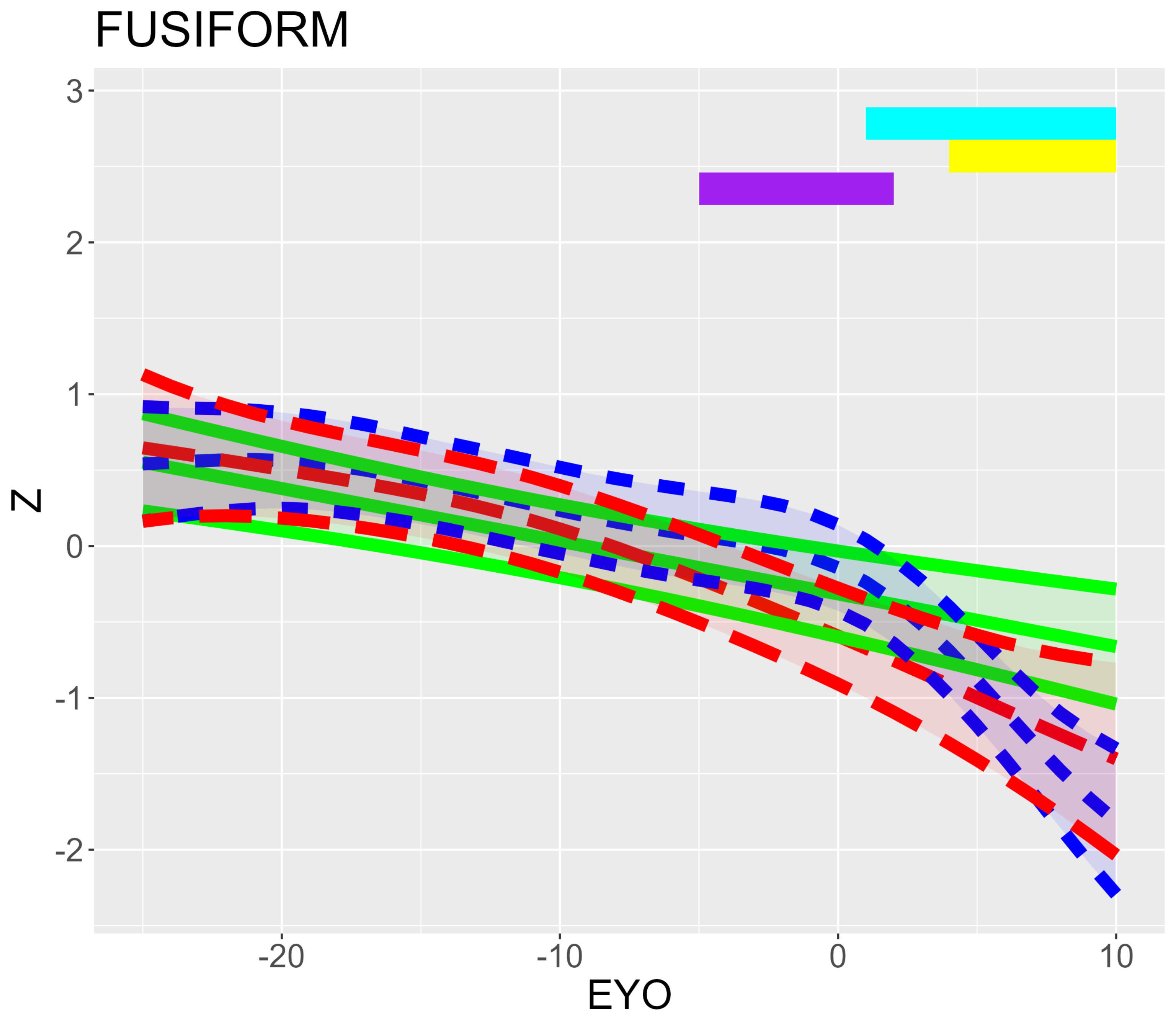

FUSIFORM
3
2
1
0
-1
-2
Z
-20
-10
0
10
EYO

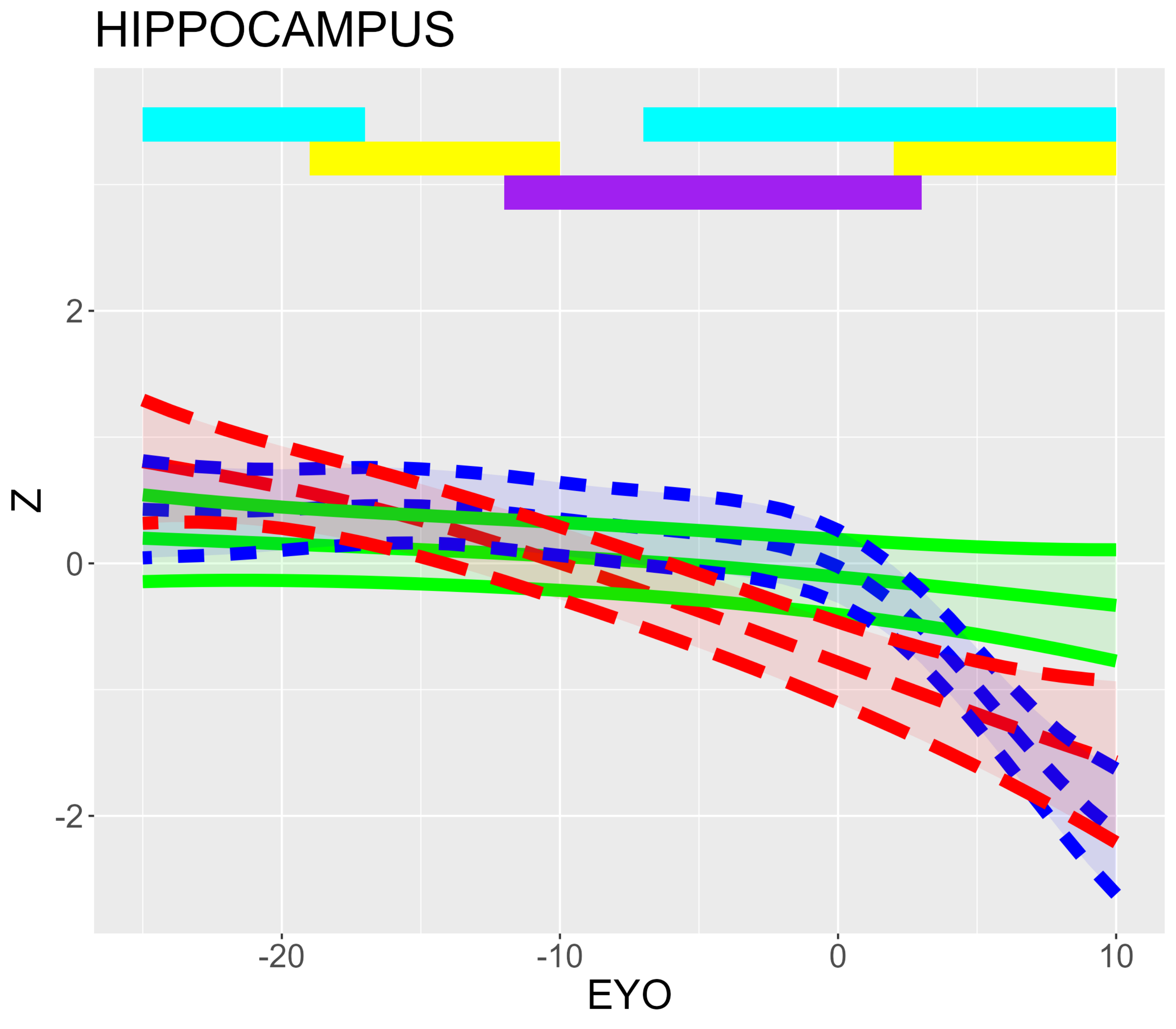
HIPPOCAMPUS
2
0
-2
Z
-20
-10
0
10
EYO

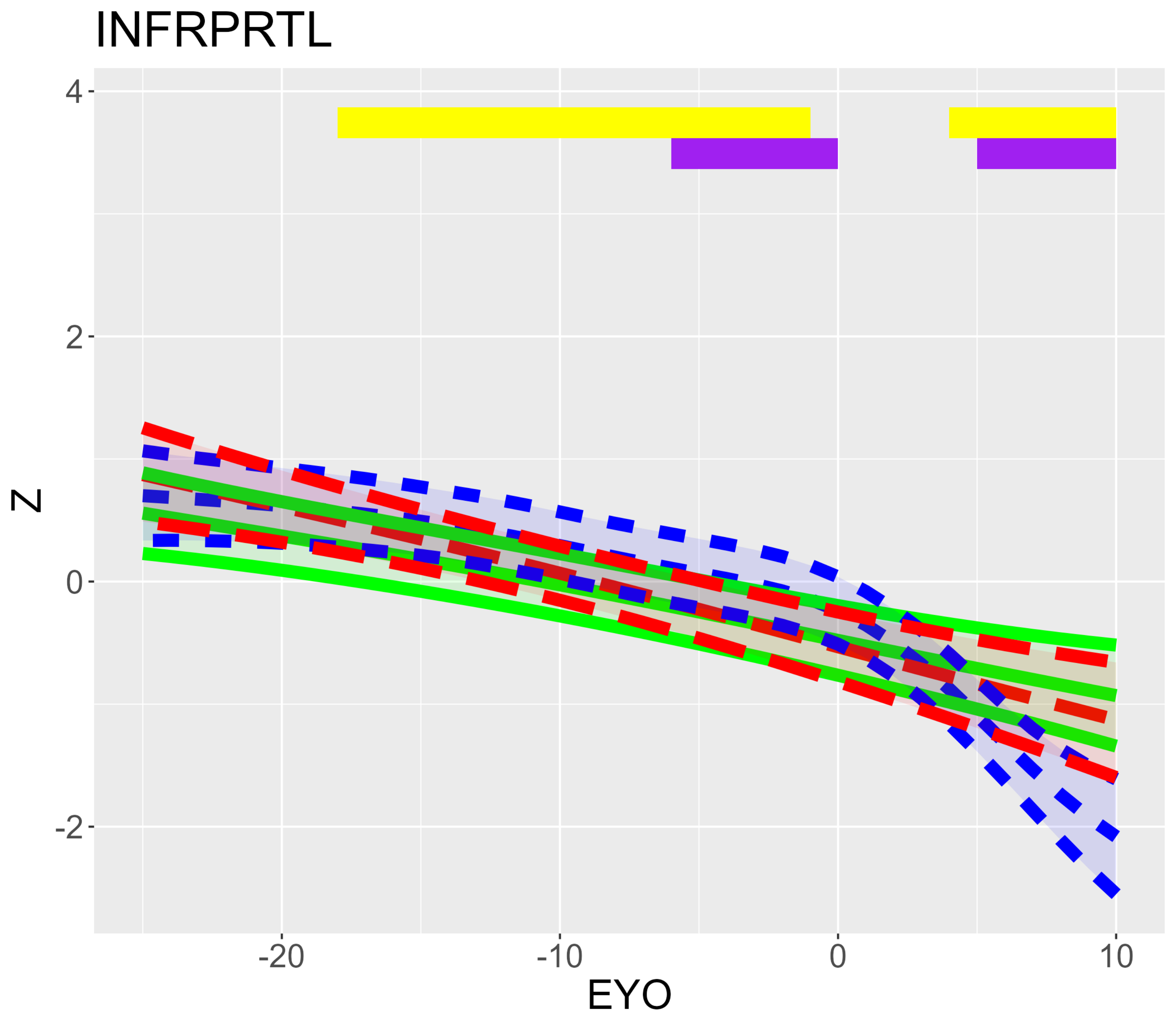
INFRPRTL
4
2
0
-2
Z
-20
-10
0
10
EYO

INFRTMP

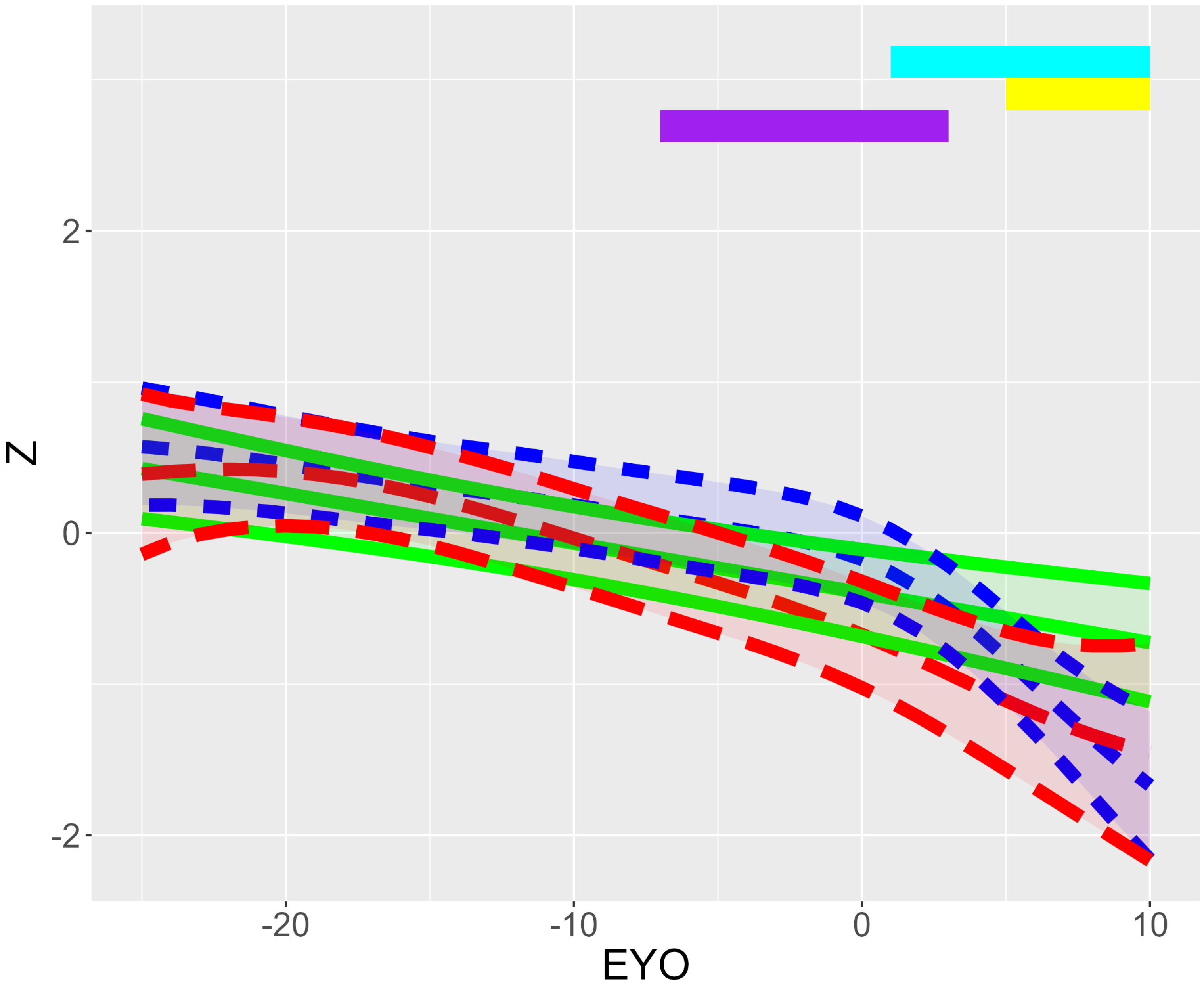

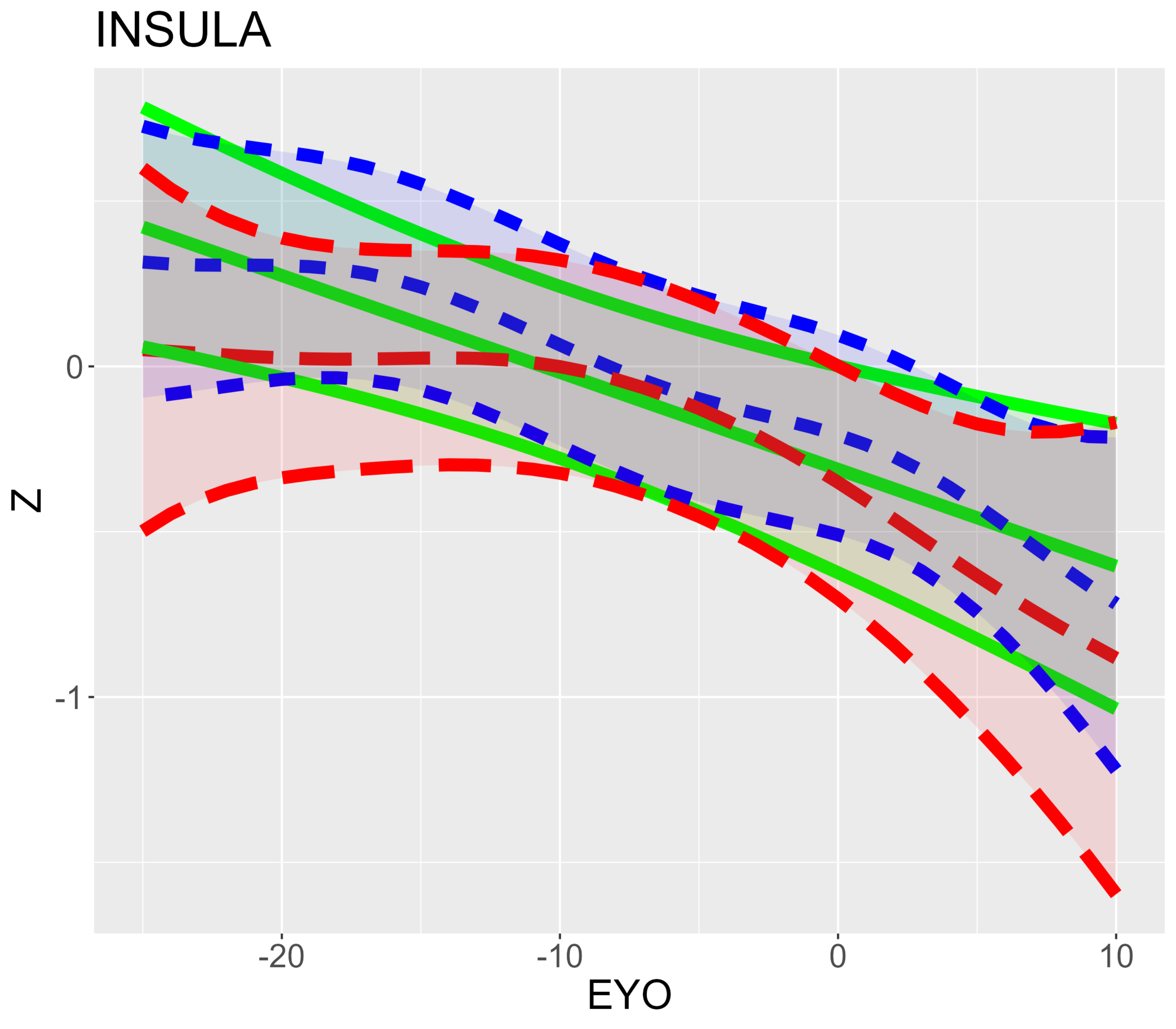

INSULA
0
-1
Z
-20
-10
0
10
EYO

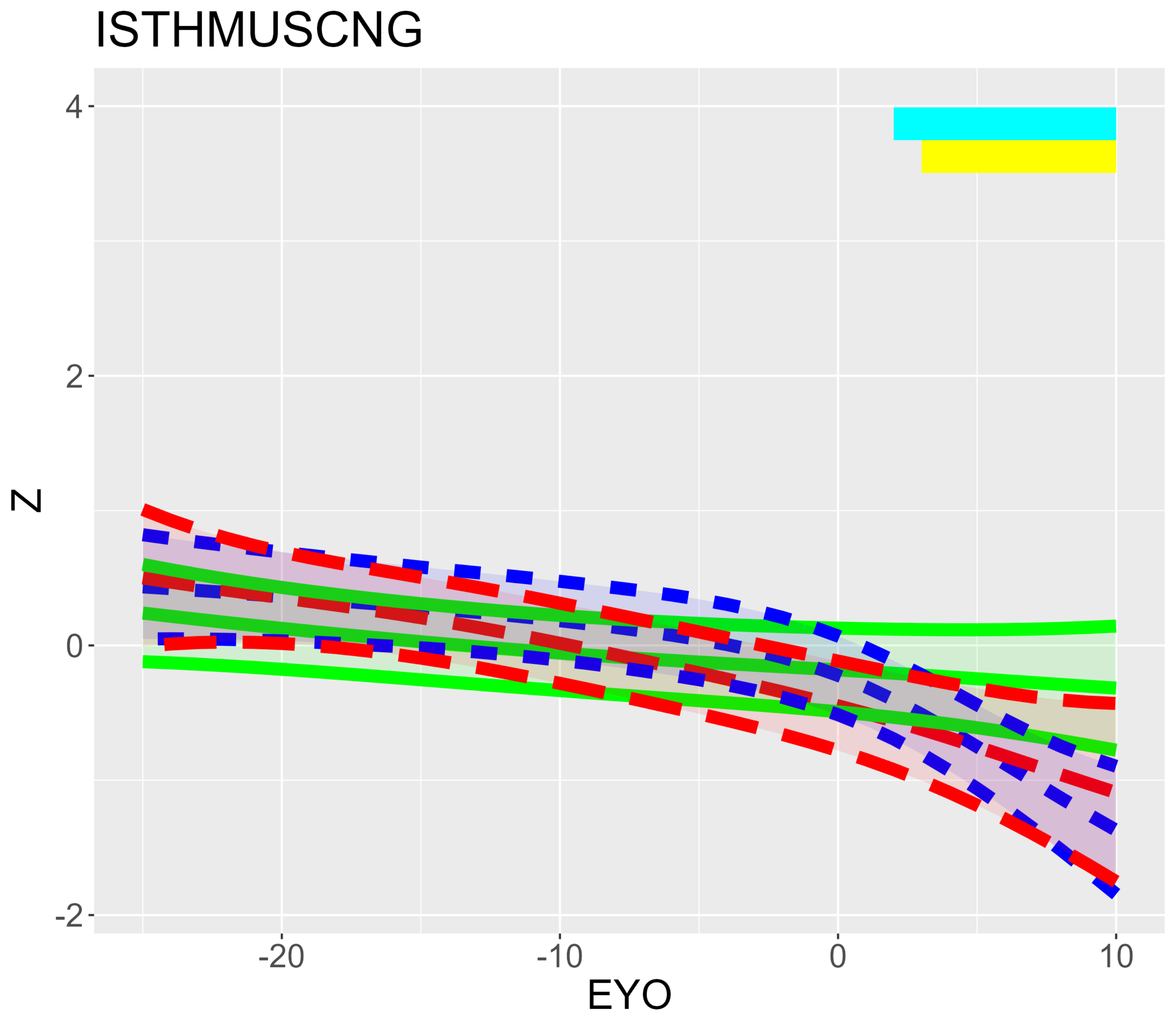

ISTHMUSCNG
4
2
0
-2
Z
-20
-10
0
10
EYO

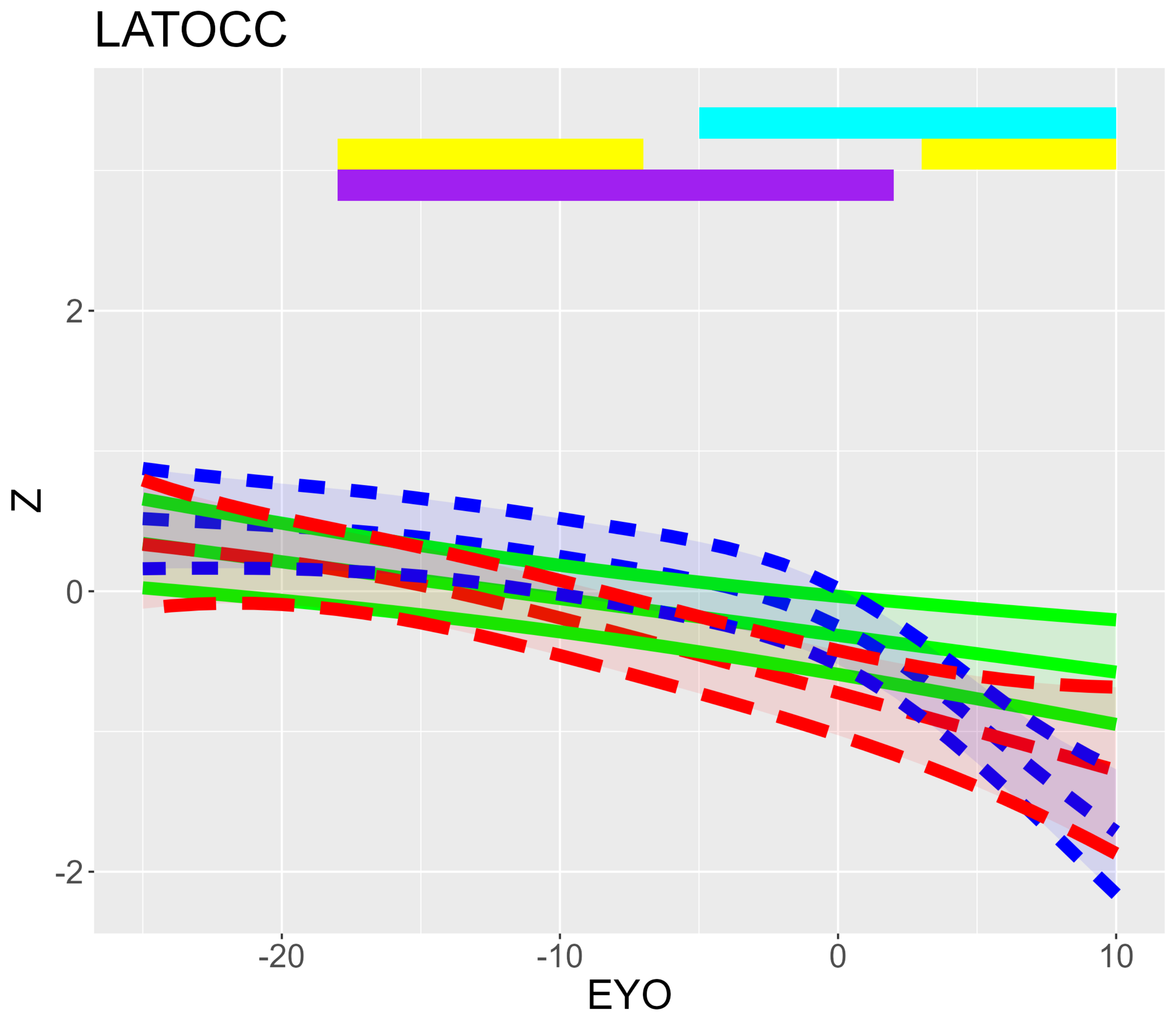
LATOCC
Z
2
0
-2
-20
-10
0
10
EYO

LATORBFRN

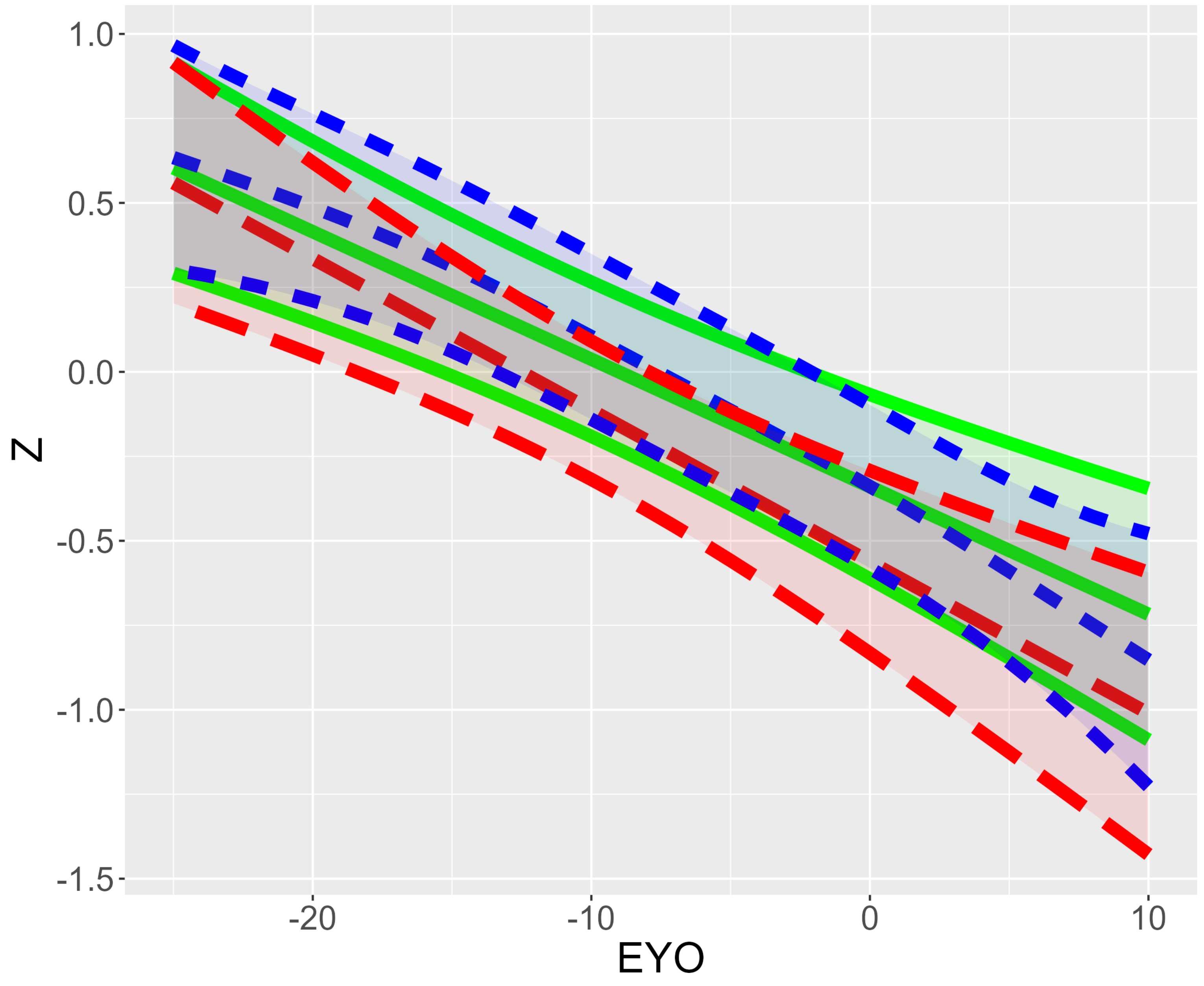

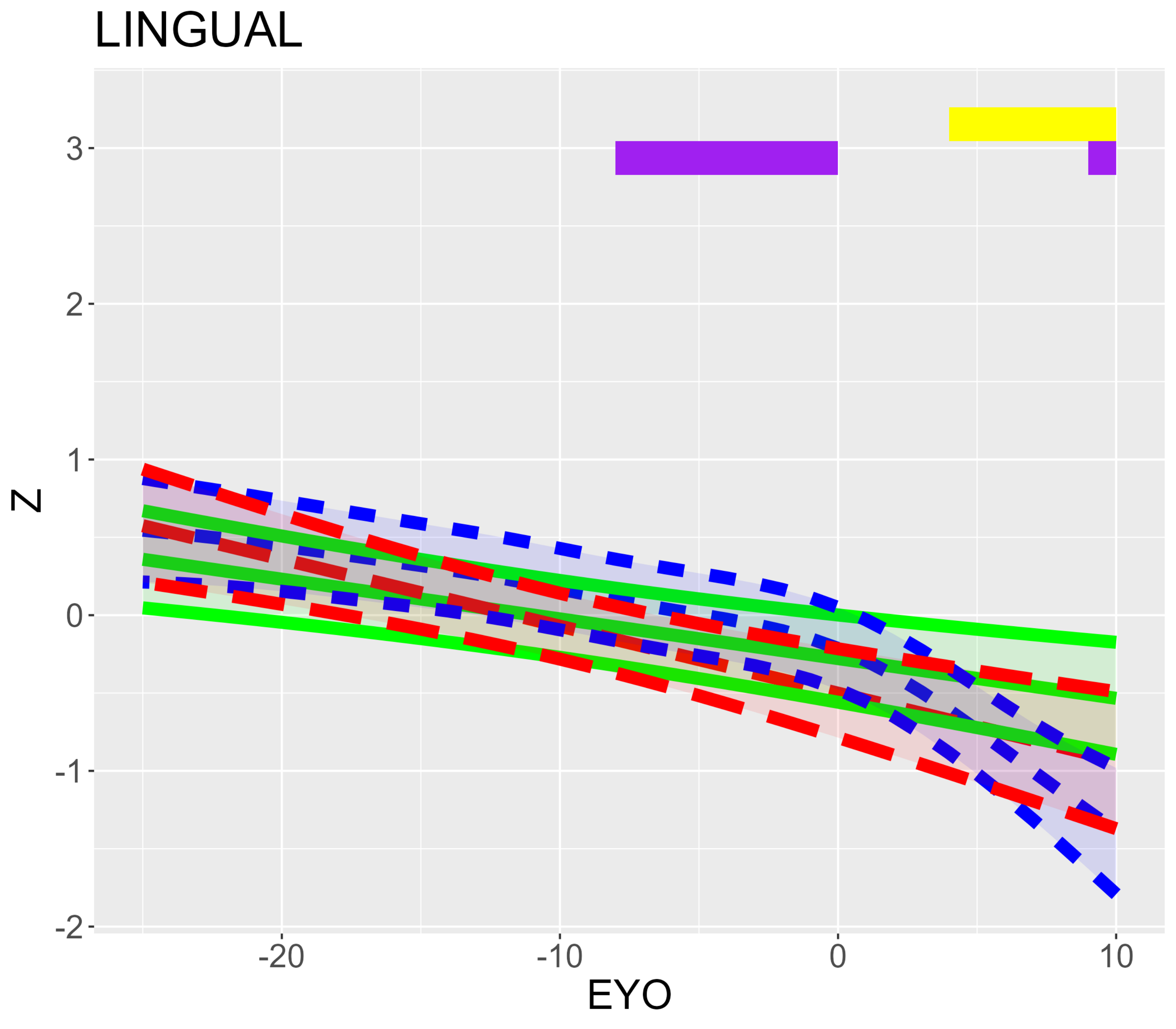
LINGUAL
Z
EYO
3
2
1
0
-1
-2
-20
-10
0
10

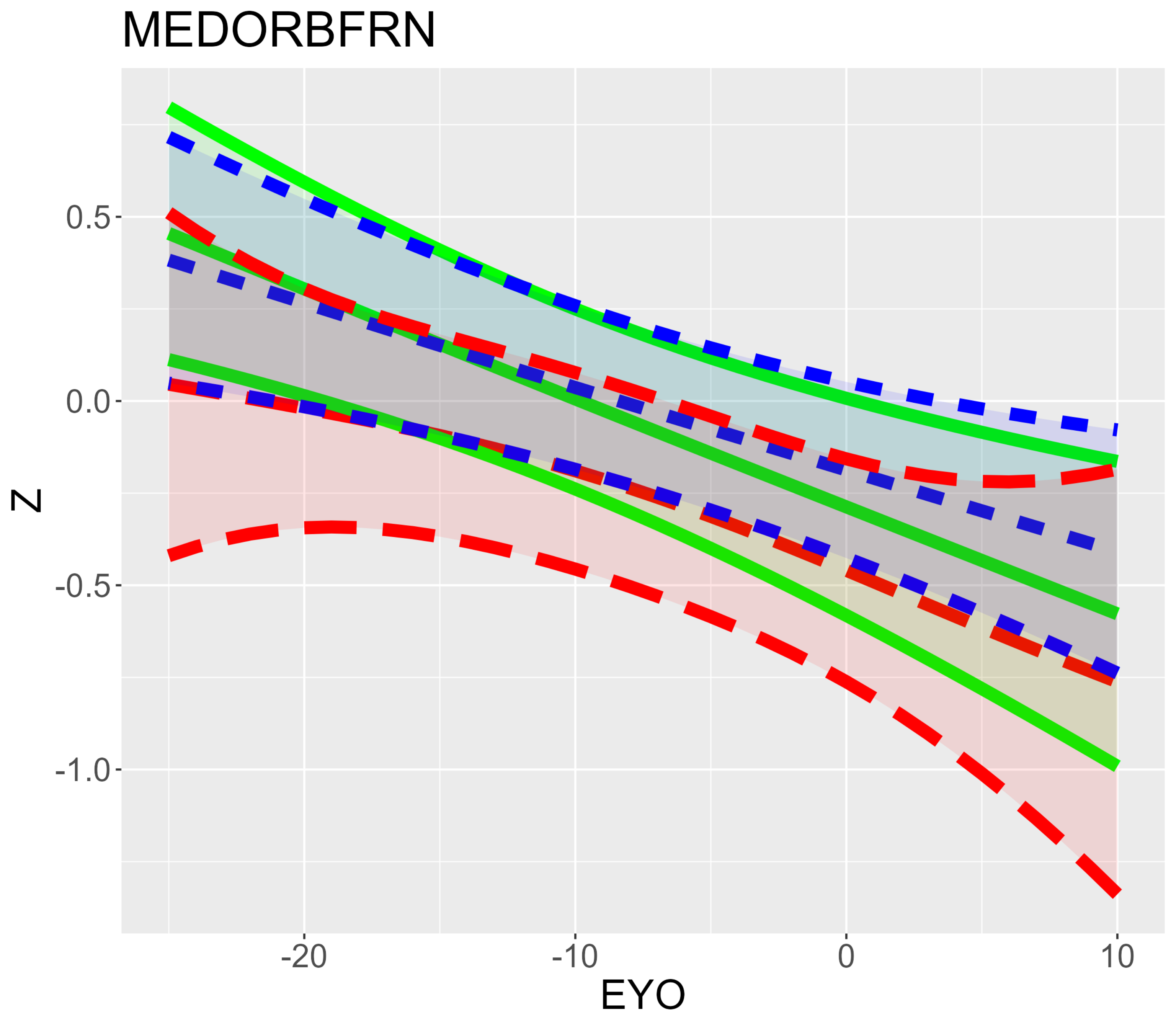
MEDORBFRN
0.5
0.0
-0.5
-1.0
Z
-20
-10
0
10
EYO

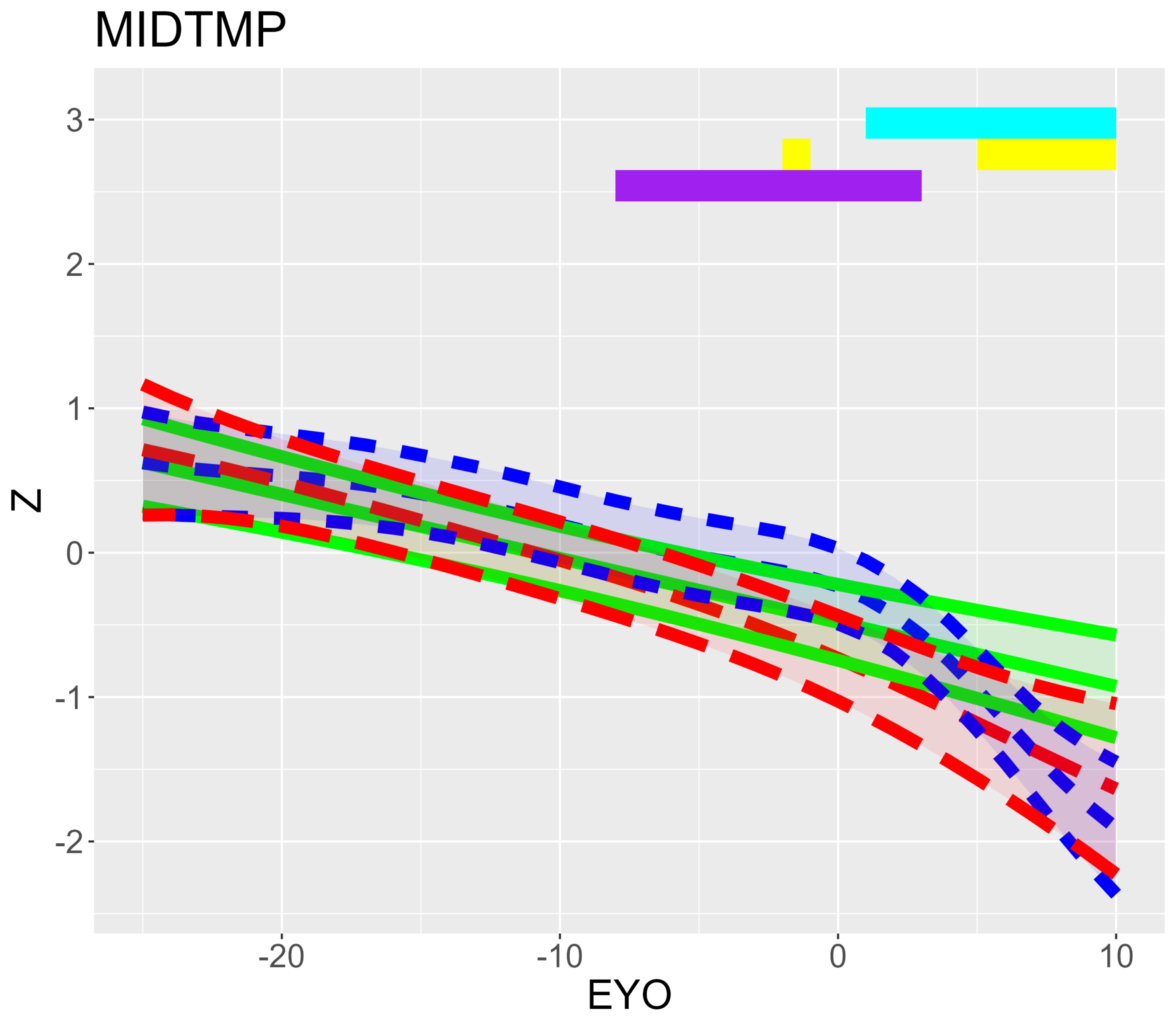
MIDTMP
Z
3
2
1
0
-1
-2
-20
-10
0
10
EYO

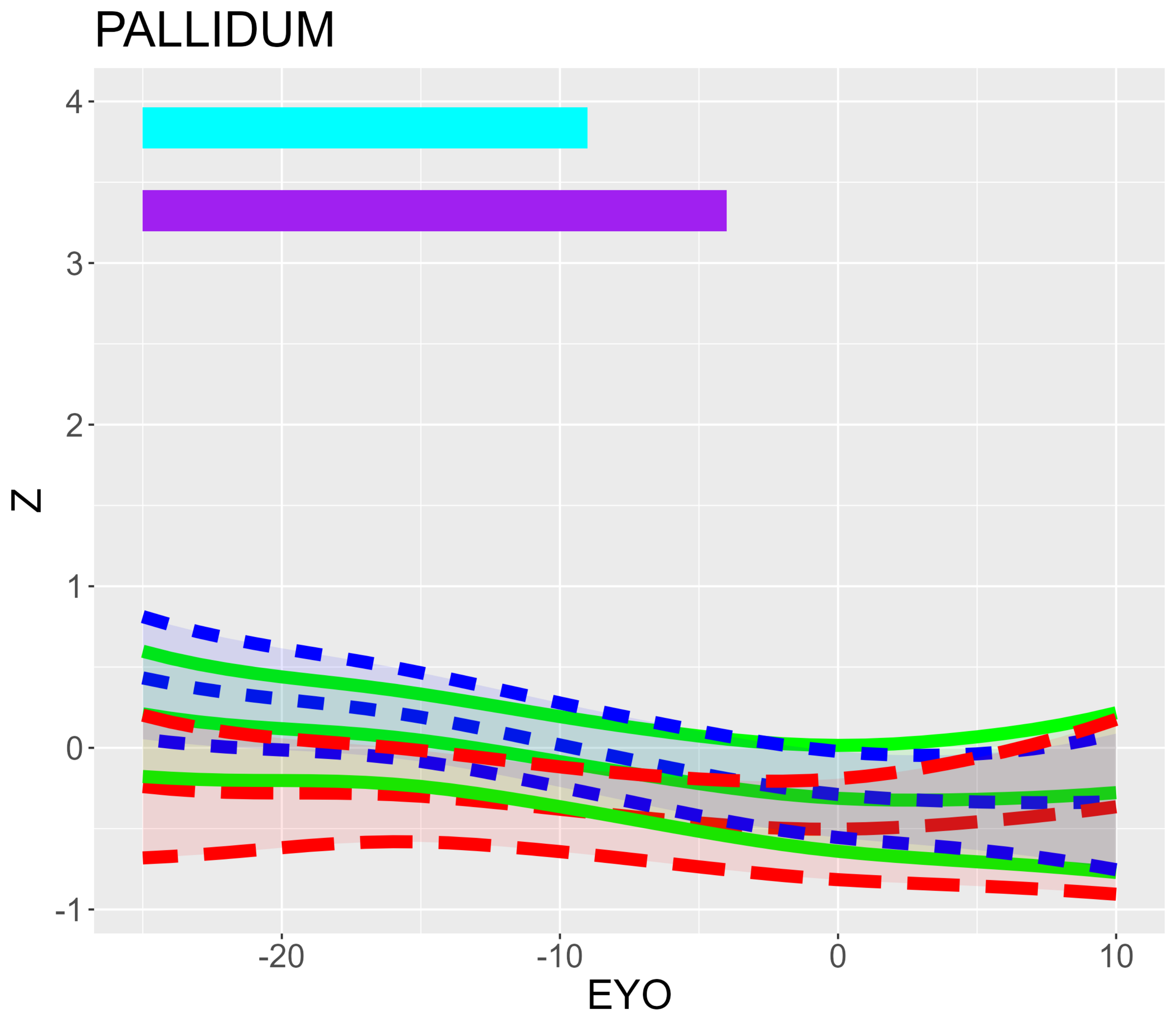
PALLIDUM
Z
EYO
4
3
2
1
0
-1
-20
-10
0
10

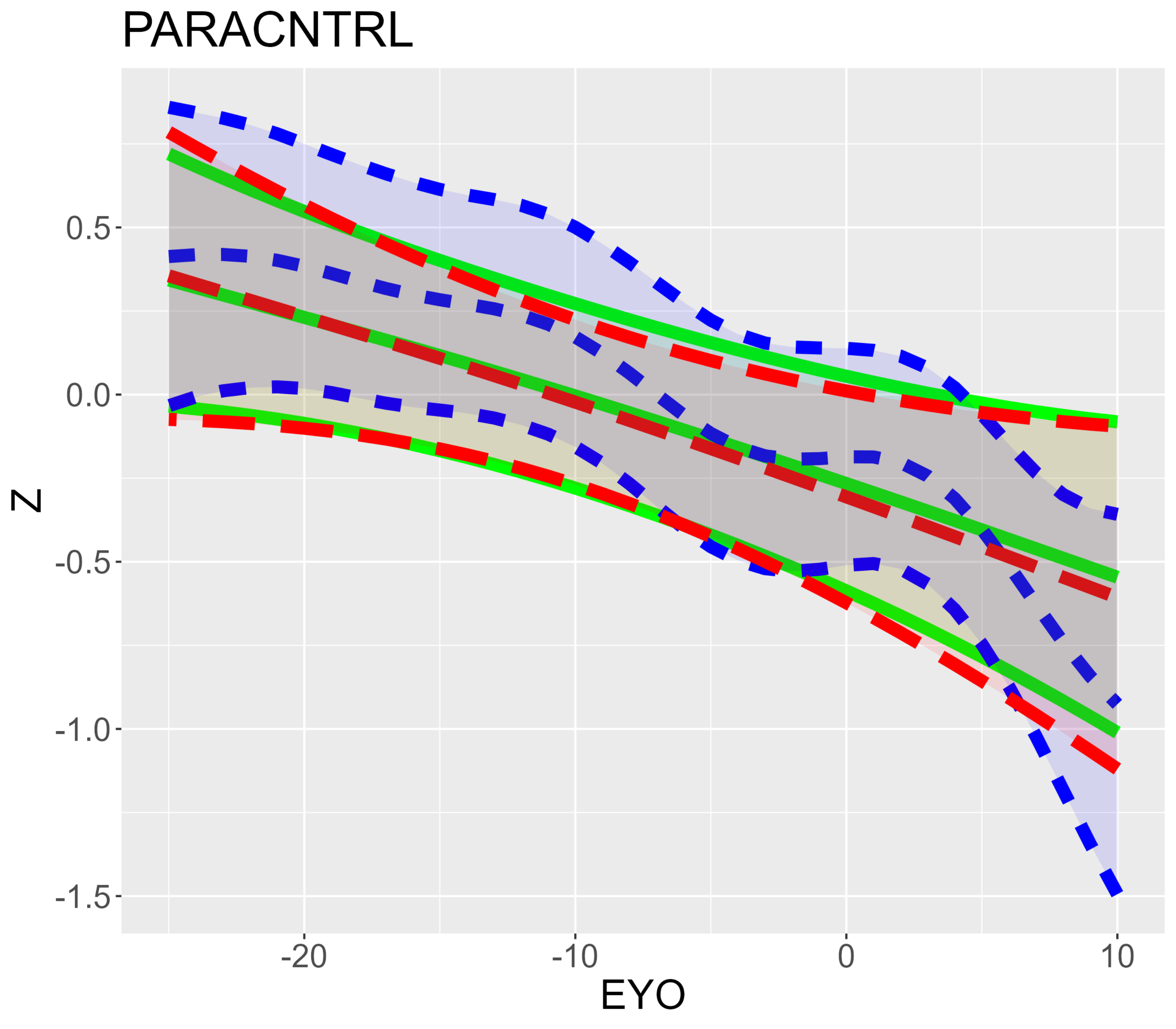
PARACNTRL
0.5
0.0
-0.5
-1.0
-1.5
Z
-20
-10
0
10
EYO

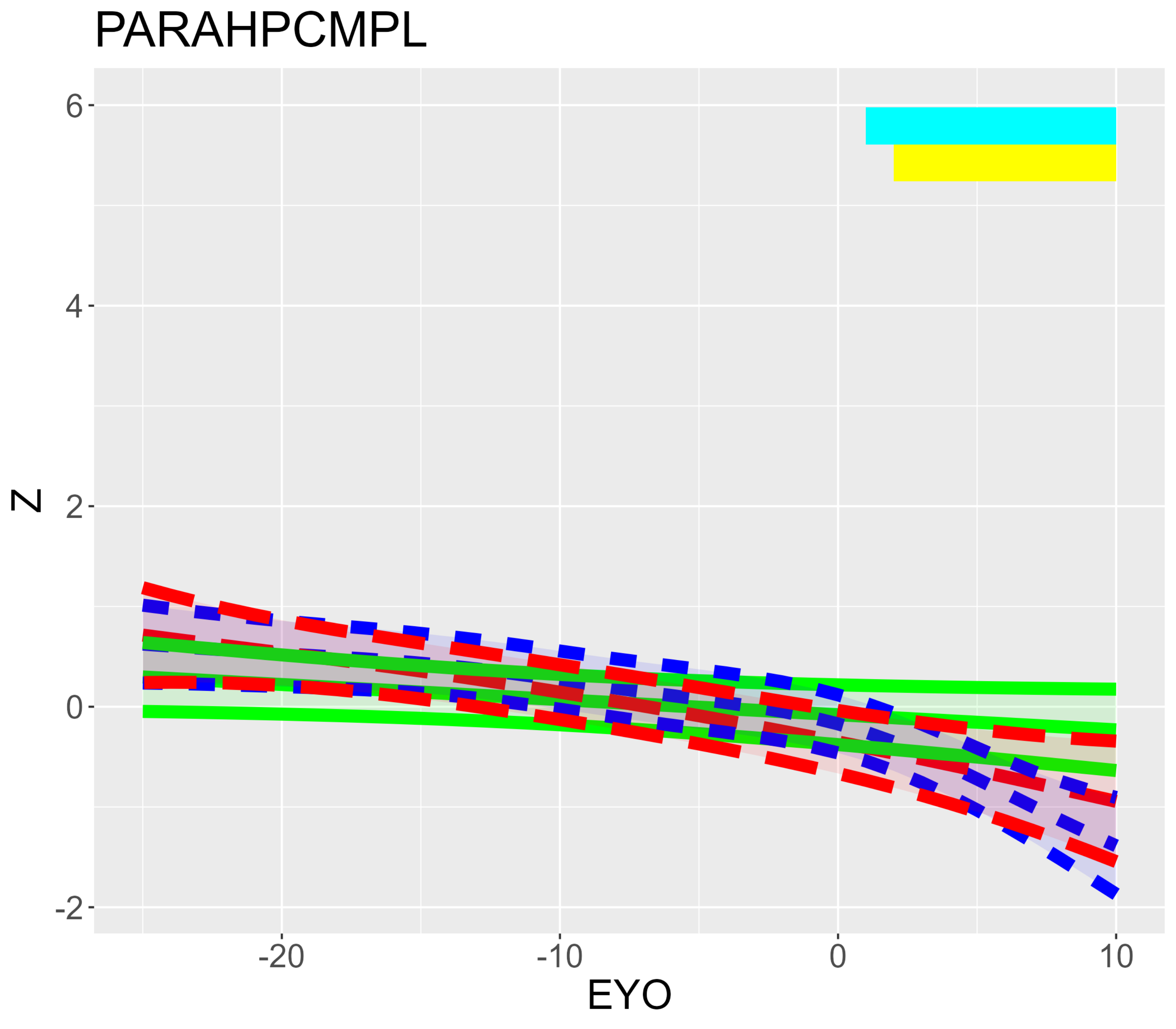
PARAHPCMPL
6
4
2
0
-2
Z
-20
-10
0
10
EYO

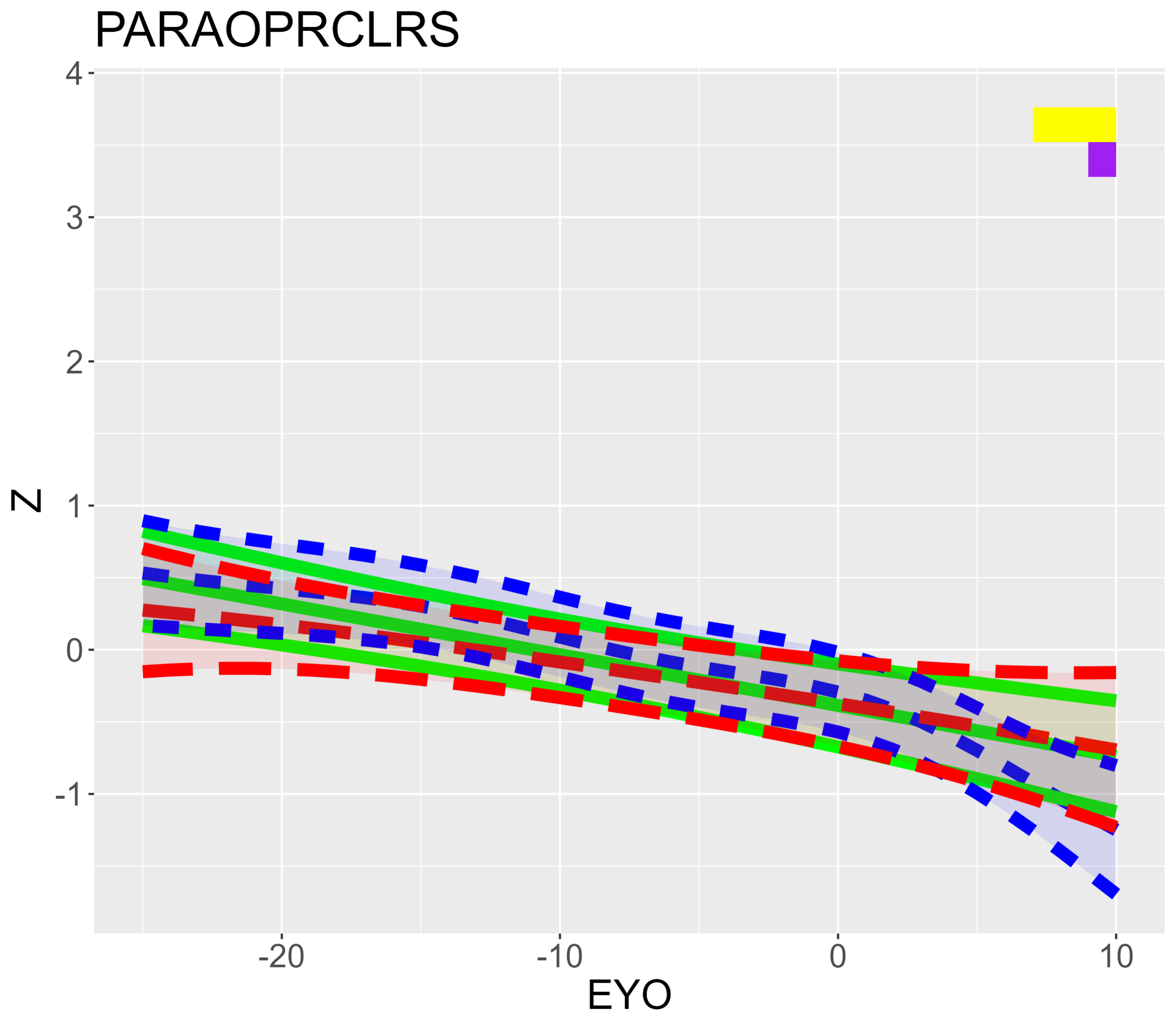
PARAOPRCLRS
4
3
2
1
0
-1
Z
-20
-10
0
10
EYO

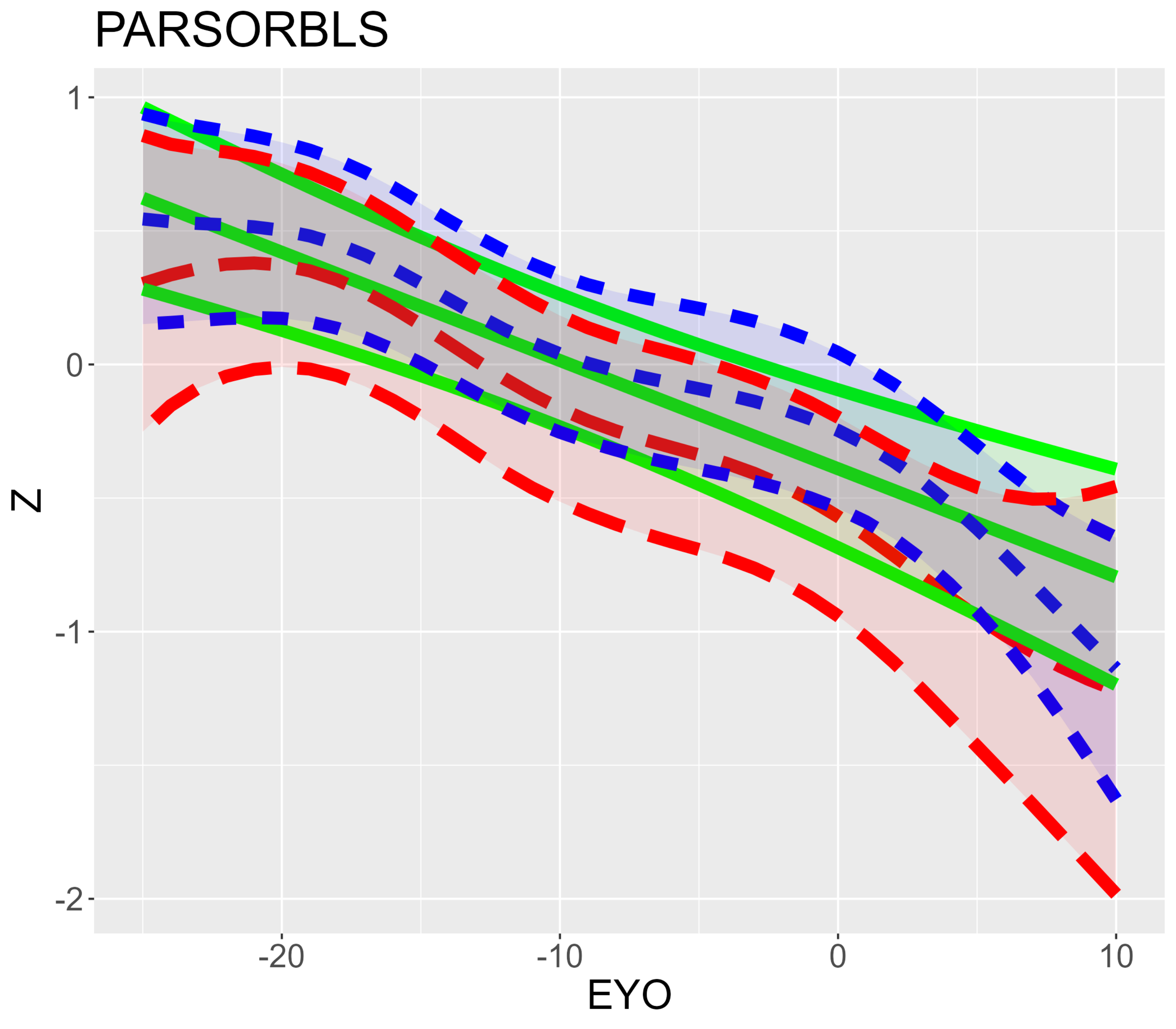

PARSORBLS
1
0
-1
-2
Z
-20
-10
0
10
EYO

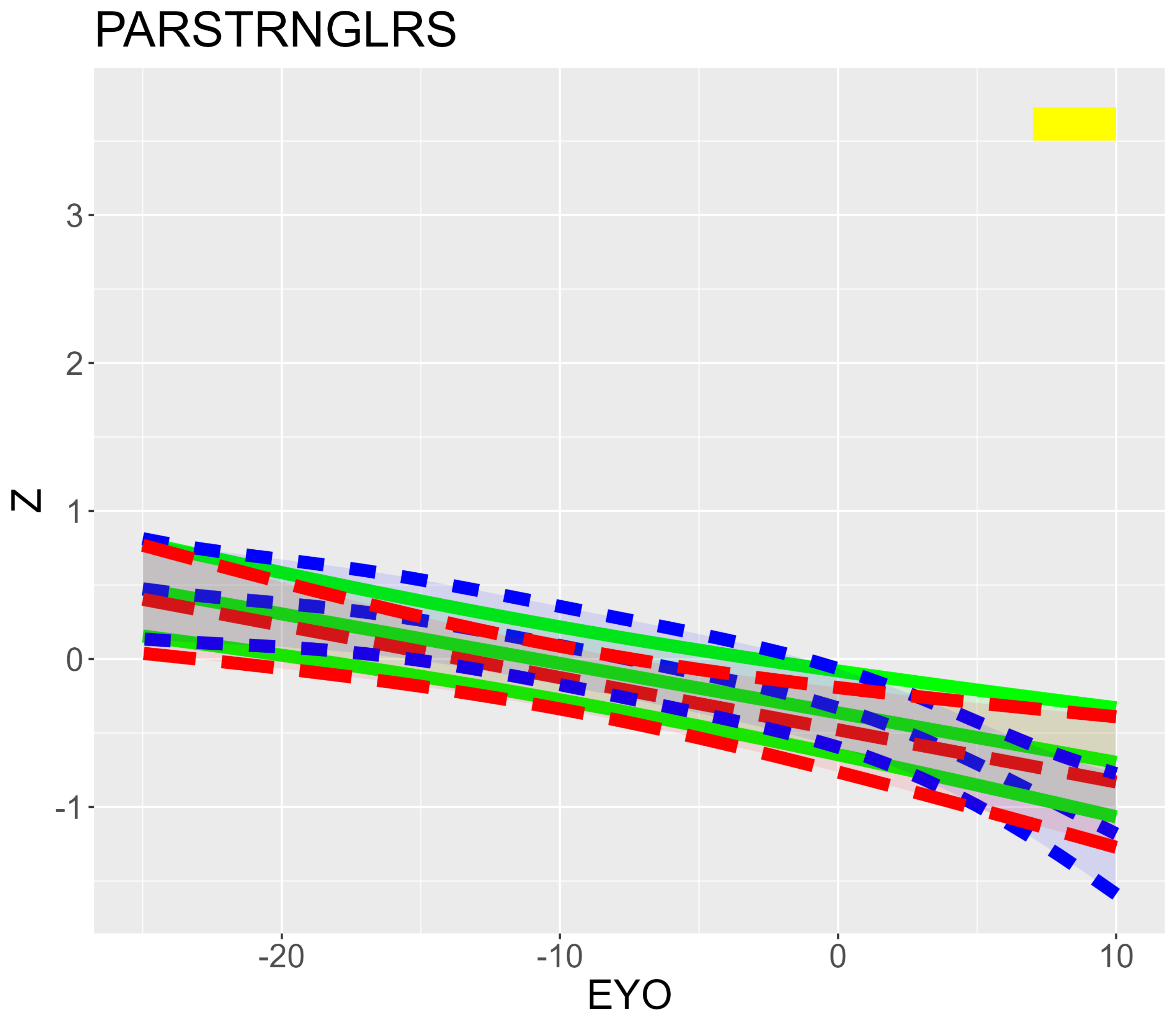

PARSTRNGLRS
3
2
1
0
-1
Z
-20
-10
0
10
EYO

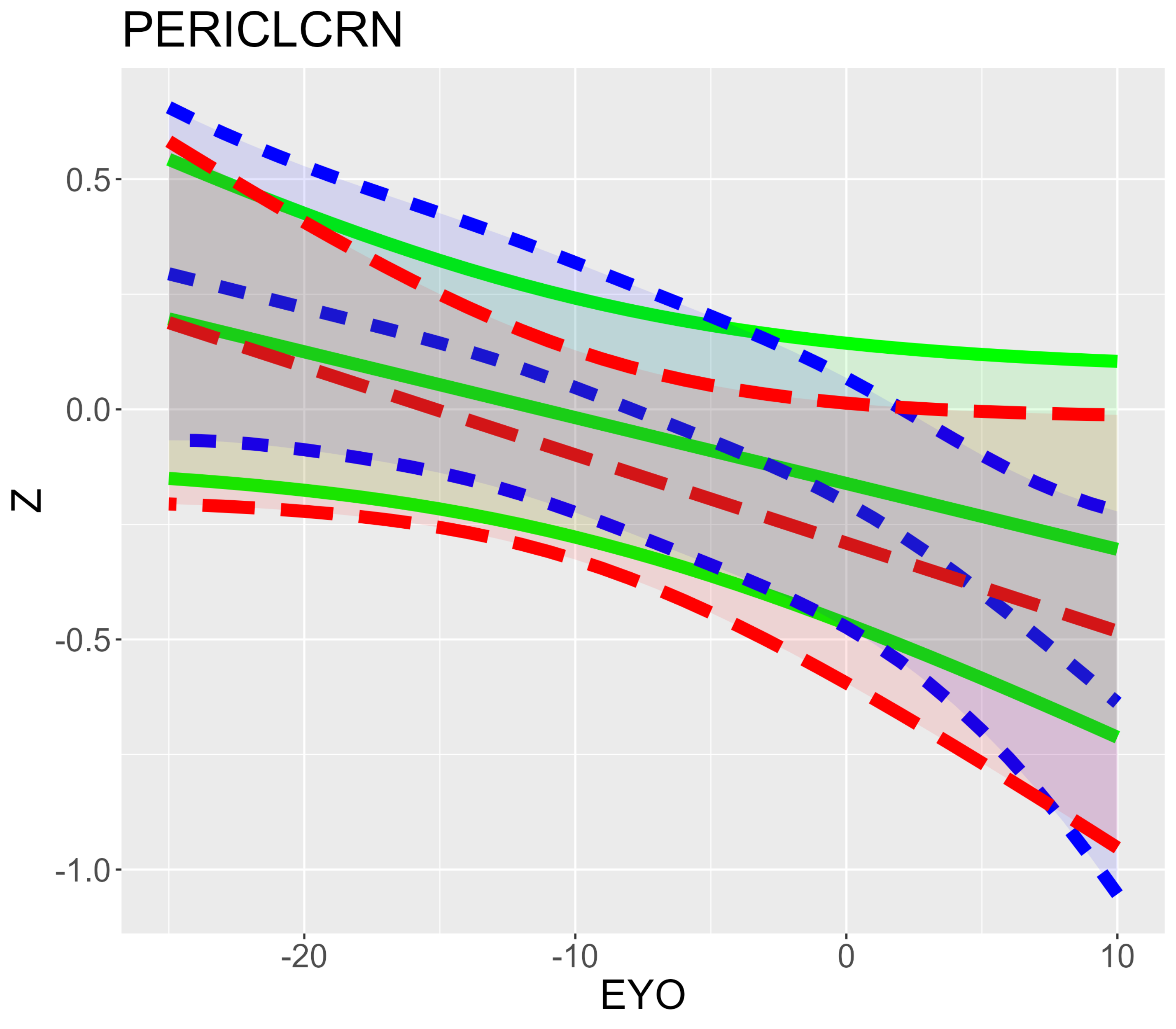
PERICLCRN
0.5
0.0
-0.5
-1.0
Z
-20
-10
0
10
EYO

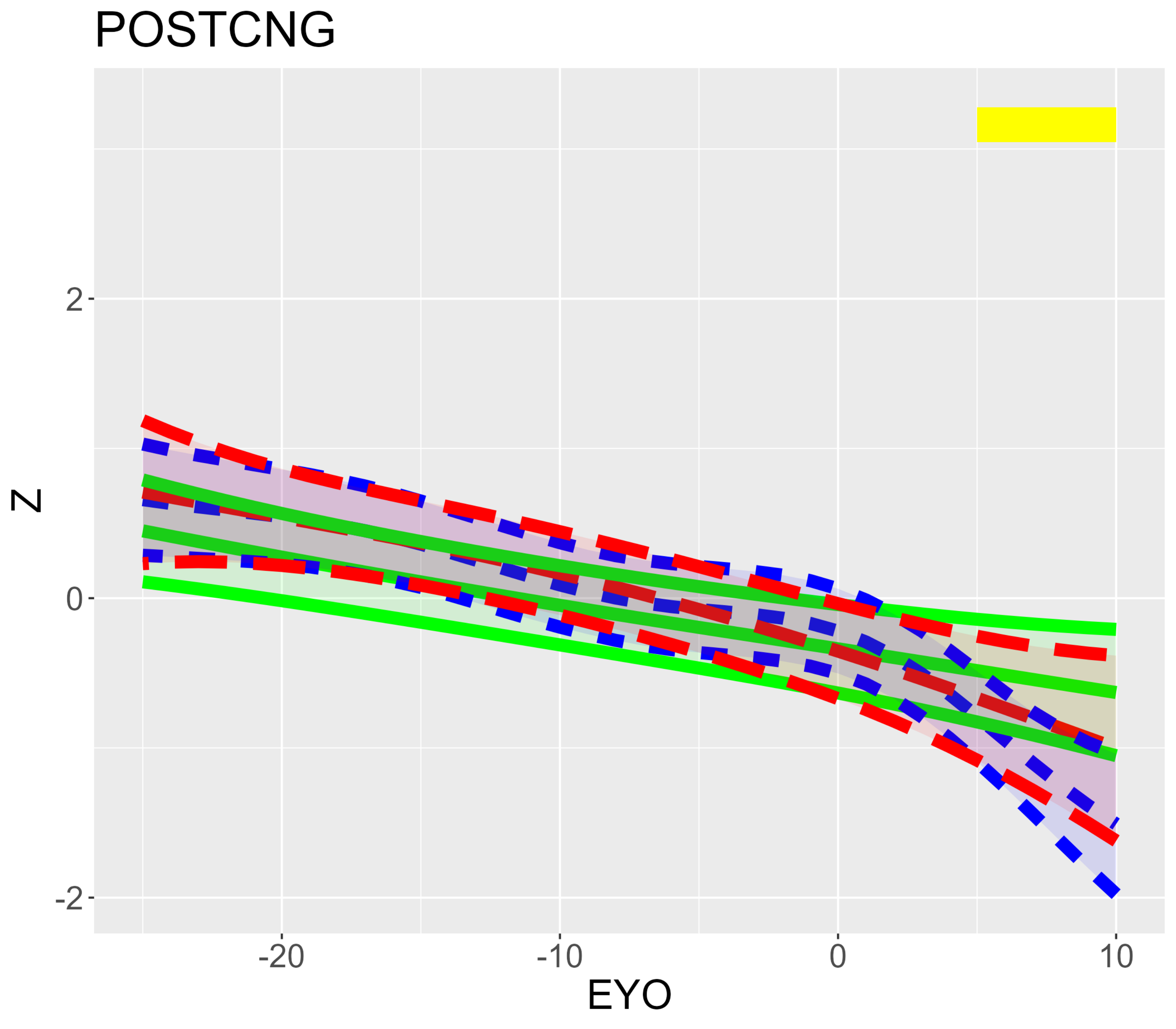
POSTCNG
Z
EYO
2
0
-2
-20
-10
0
10

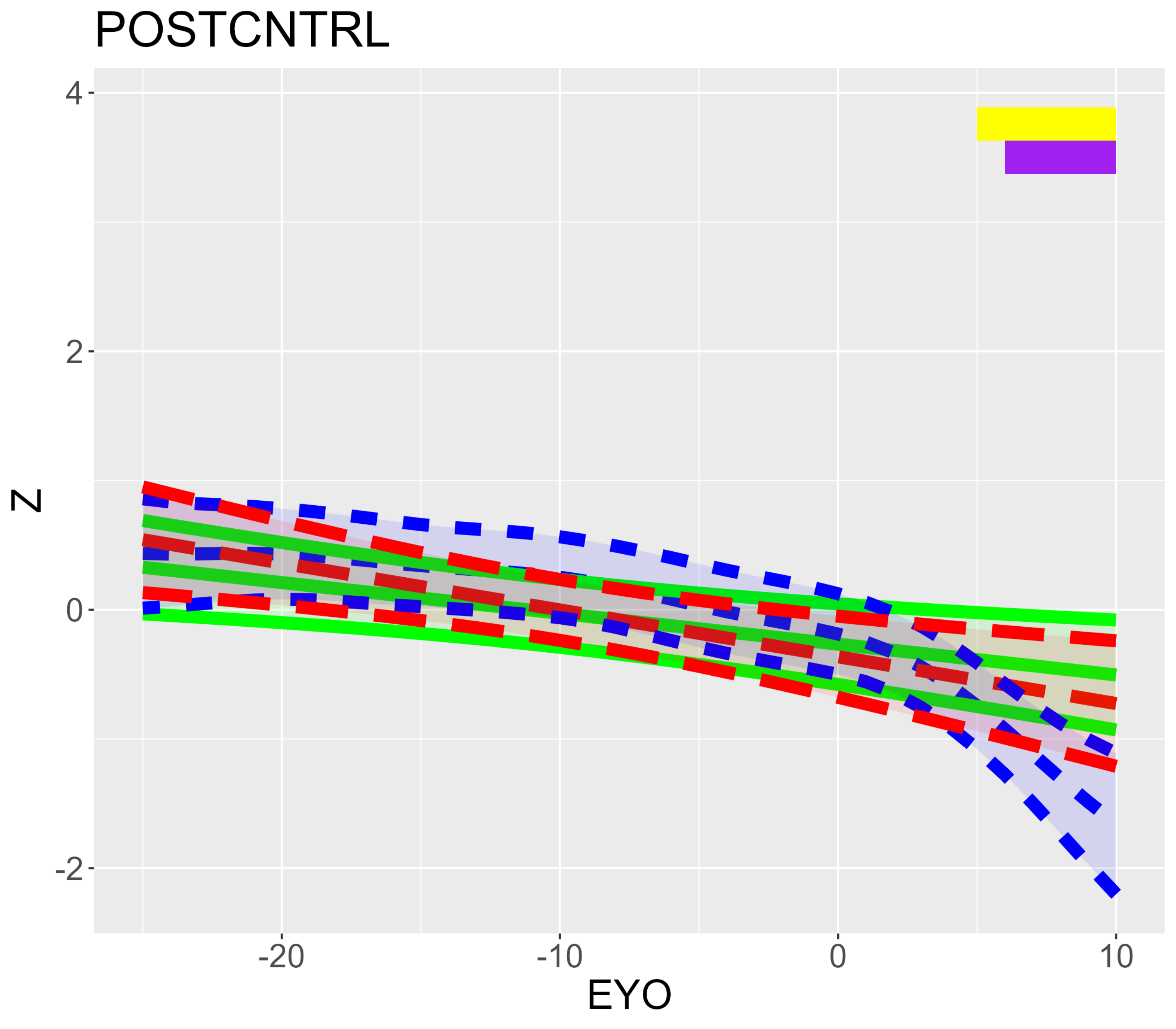
POSTCNTRL
4
2
0
-2
-20
-10
0
10
Z
EYO

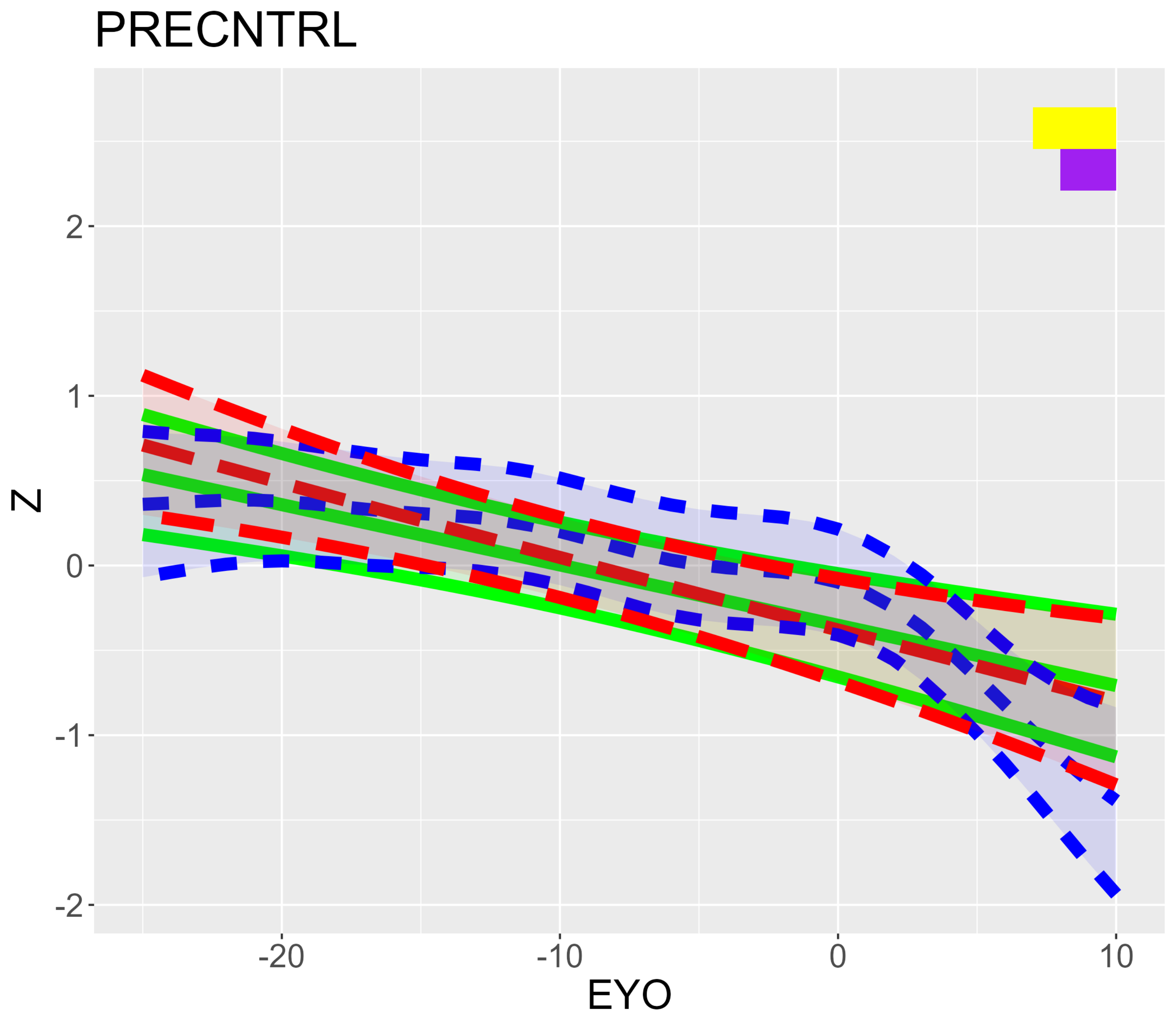

PRECNTRL
2
1
0
-1
-2
Z
-20
-10
0
10
EYO

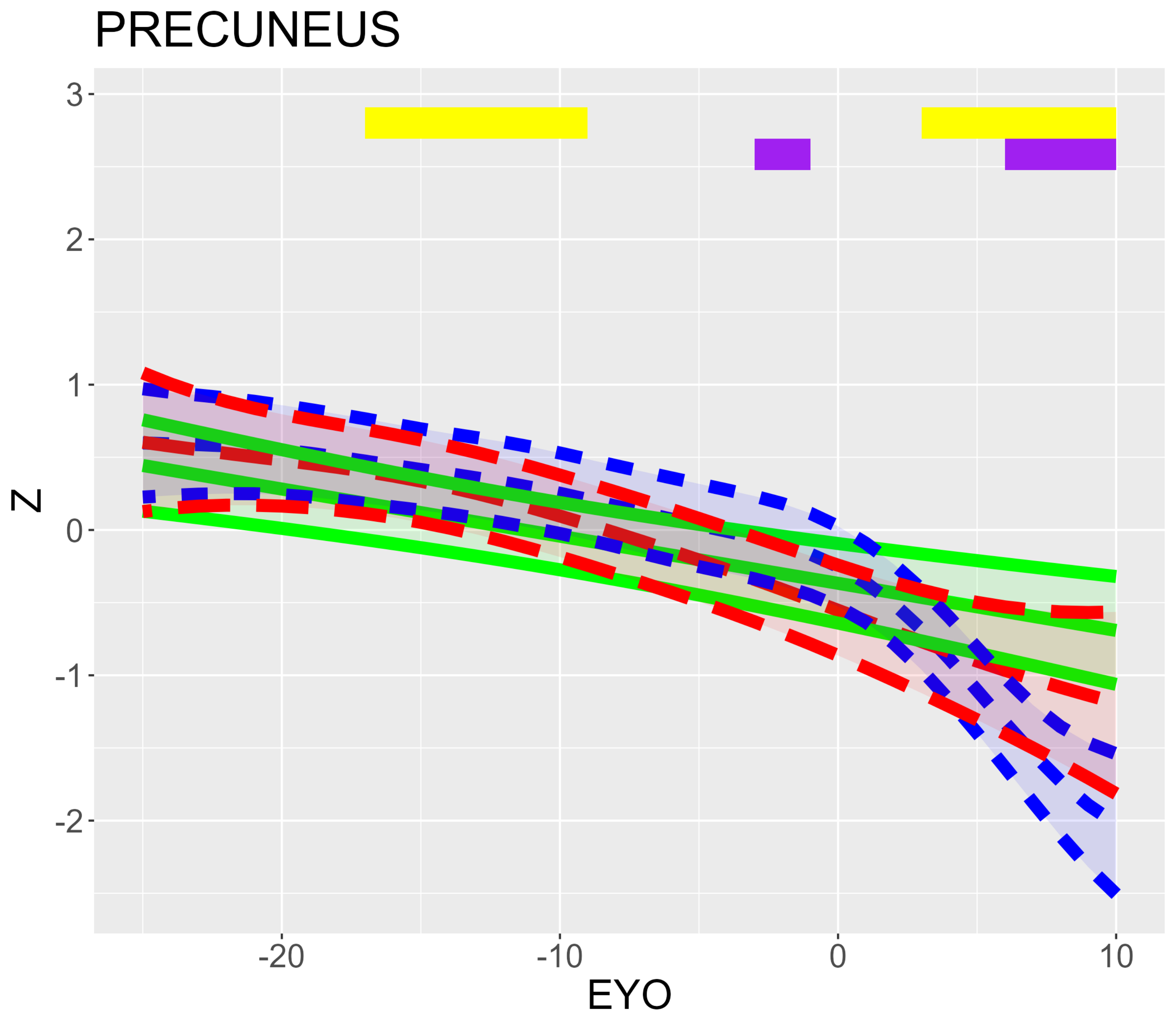
PRECUNEUS
Z
EYO
3
2
1
0
-1
-2
-20
-10
0
10

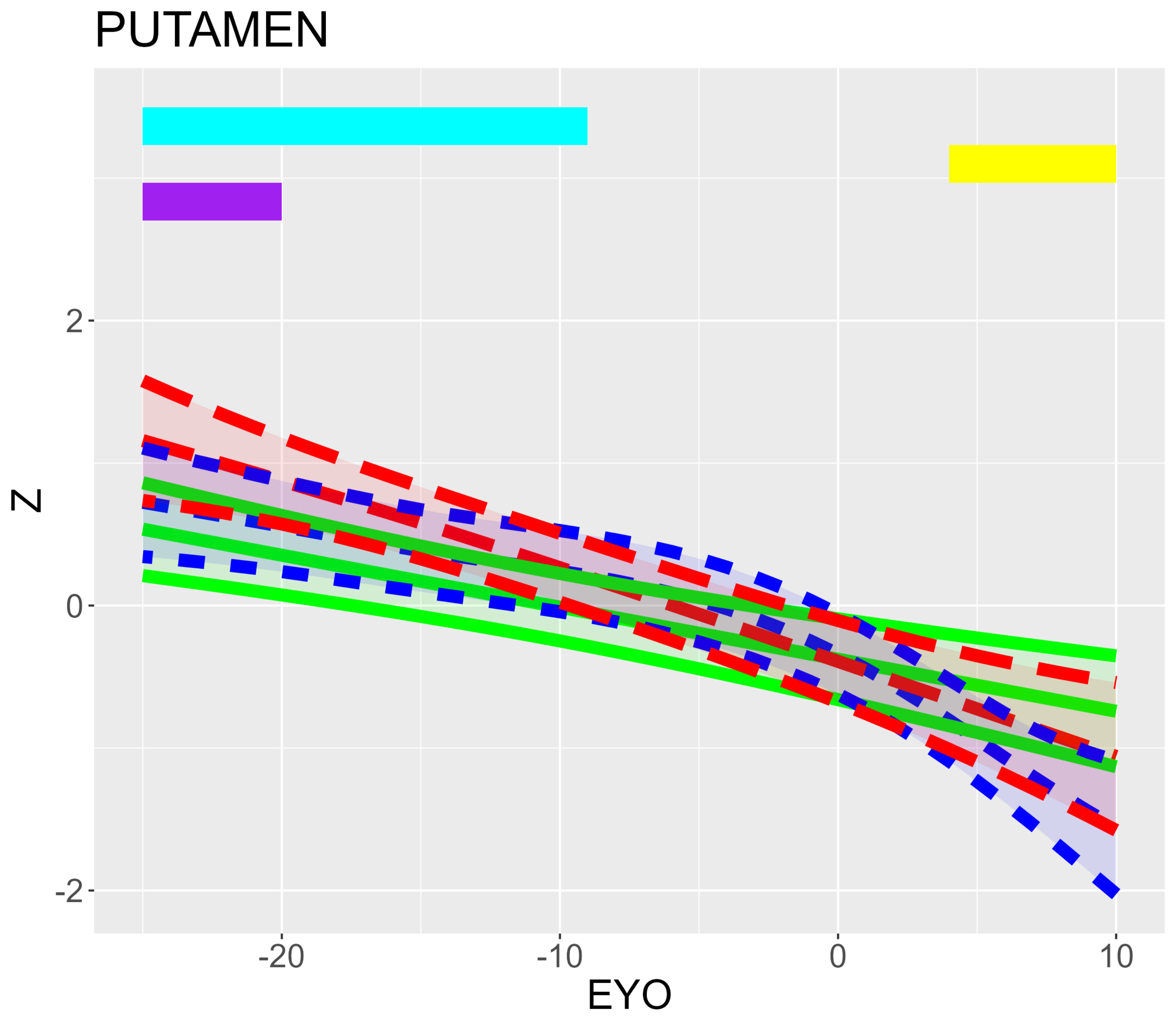

PUTAMEN
Z
2
0
-2
-20
-10
0
10
EYO

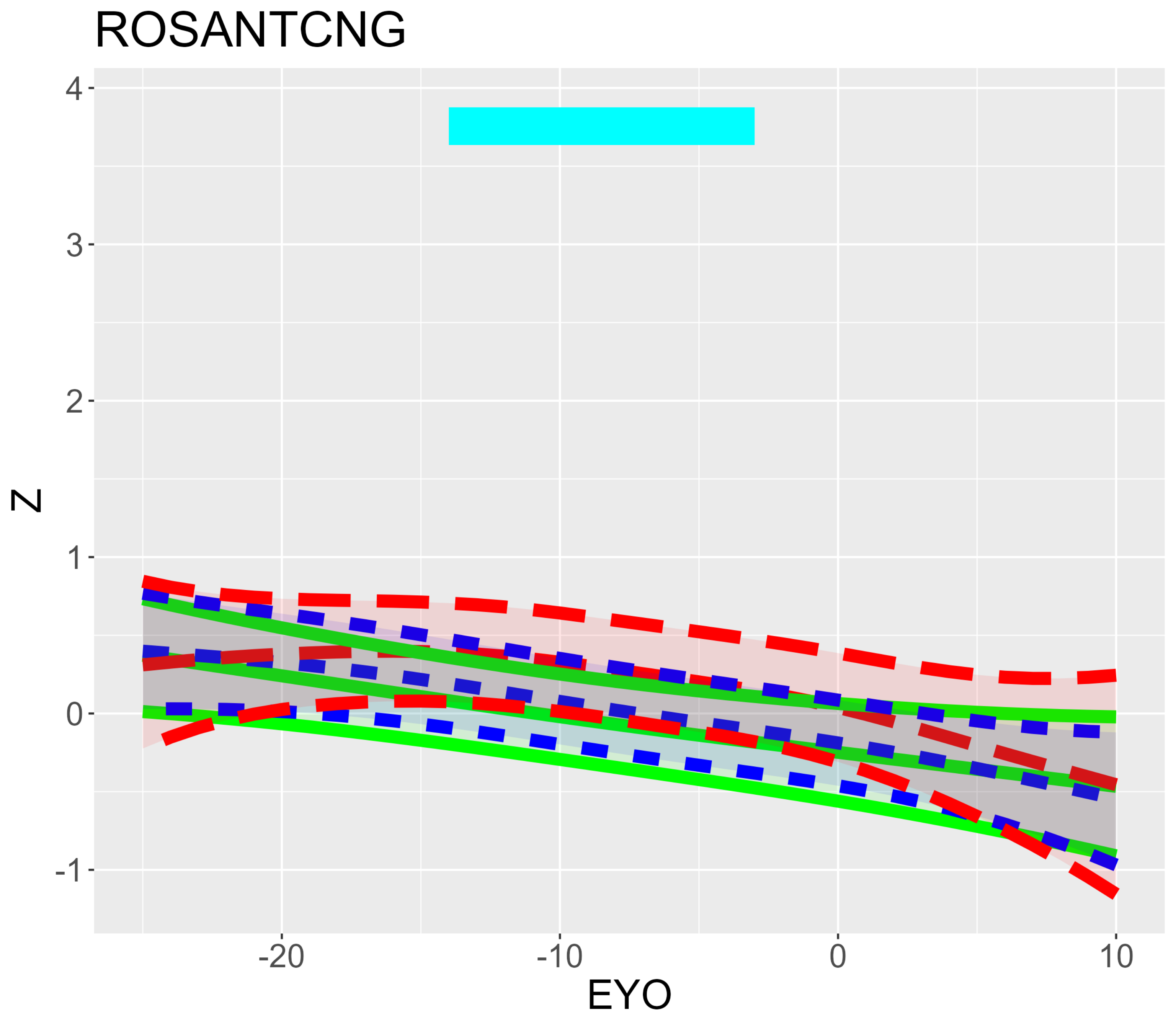

ROSANTCNG
4
3
2
1
0
-1
Z
-20
-10
0
10
EYO

ROSMIDFRN

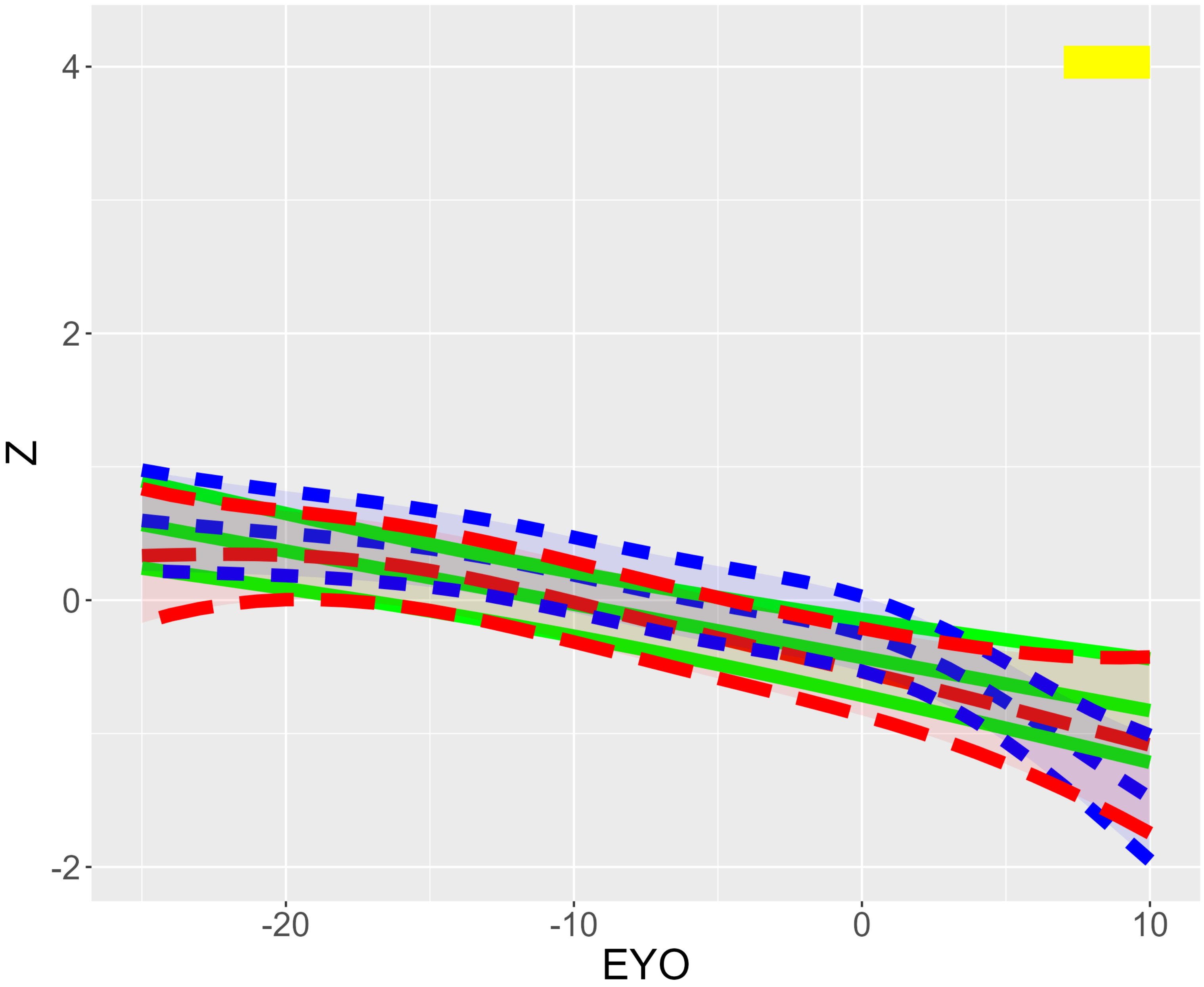

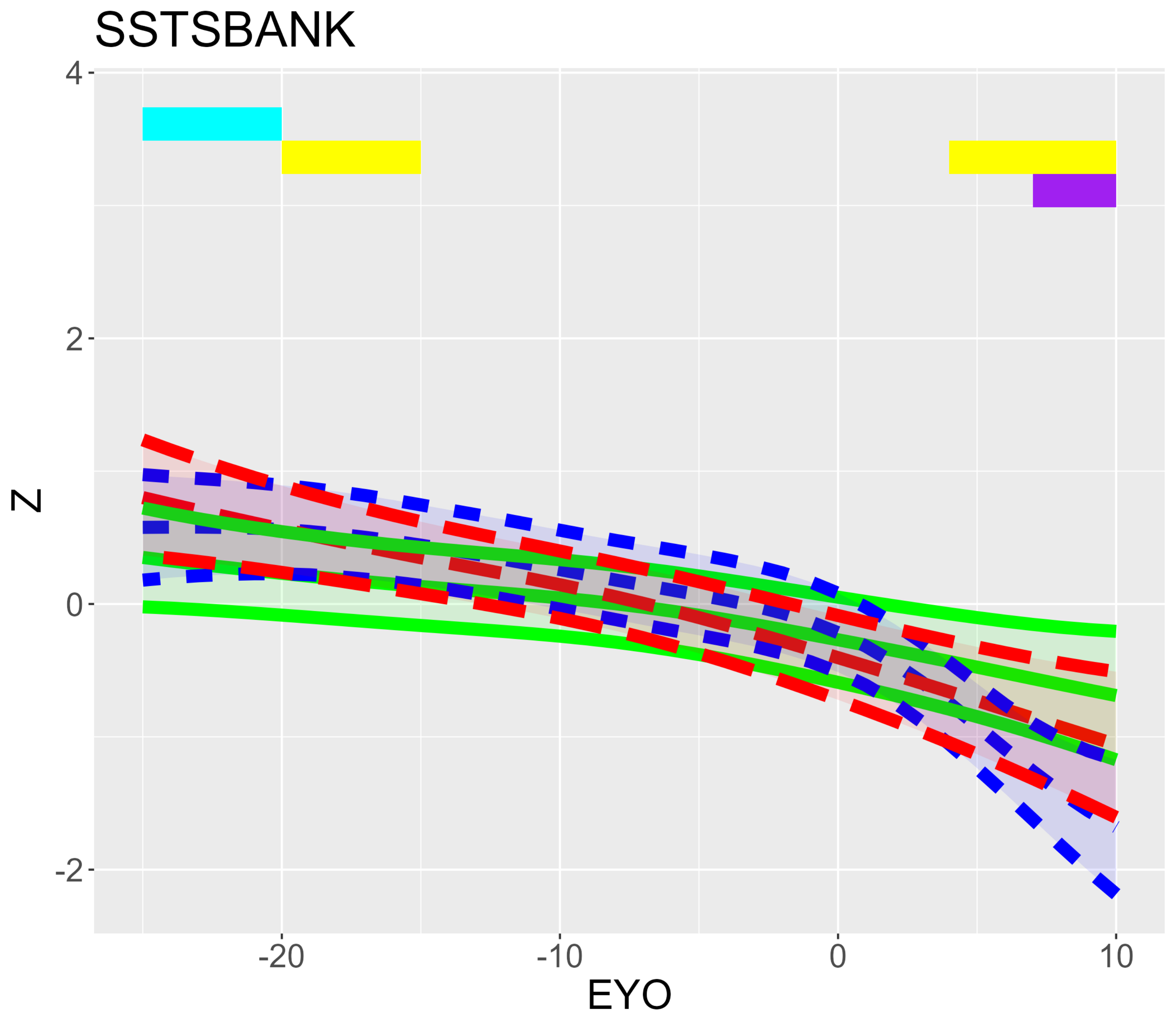

SSTSBANK
4
2
0
-2
Z
-20
-10
0
10
EYO

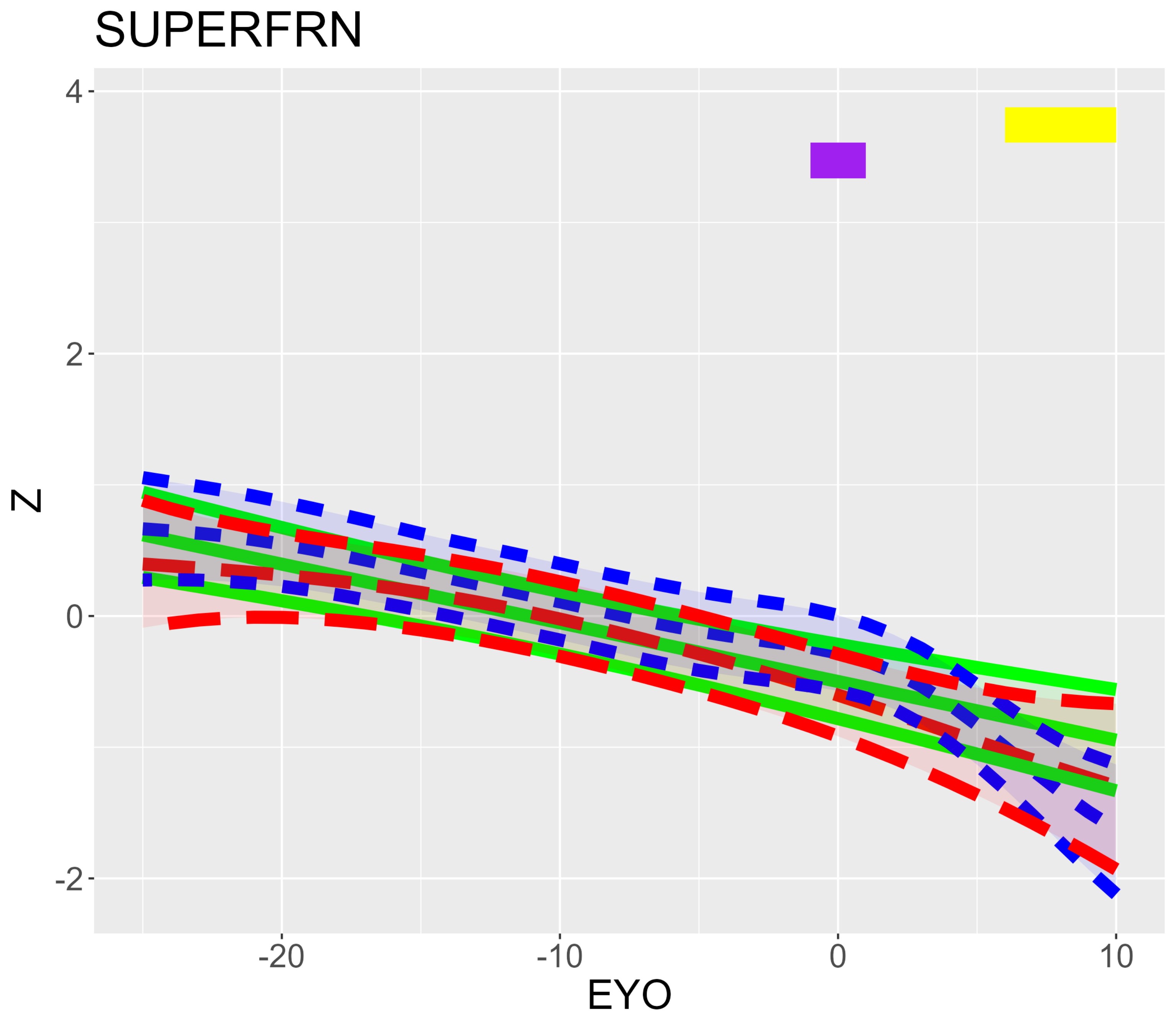
SUPERFRN
4
2
0
-2
Z
-20
-10
0
10
EYO

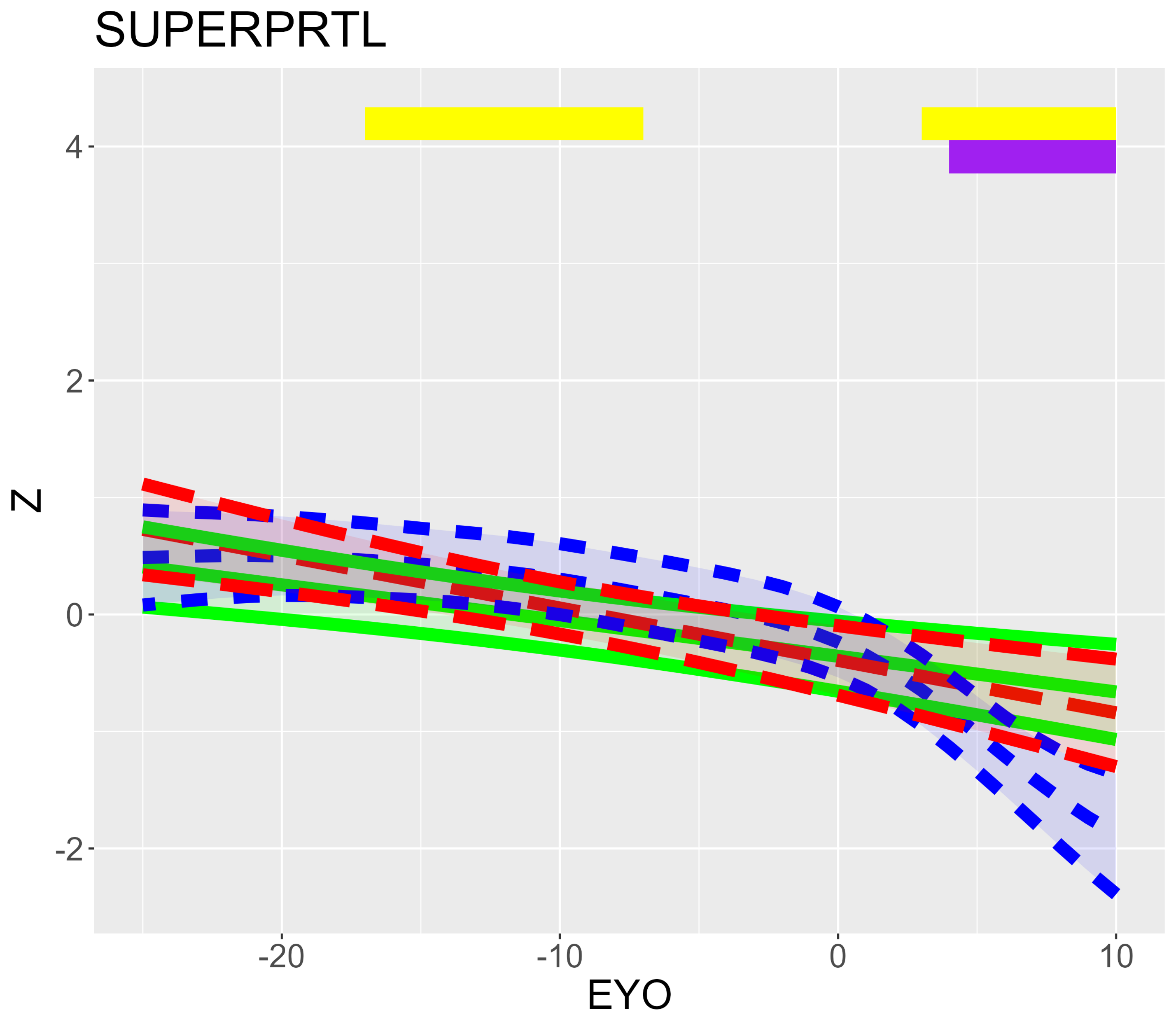
SUPERPRTL
Z
EYO
4
2
0
-2
-20
-10
0
10

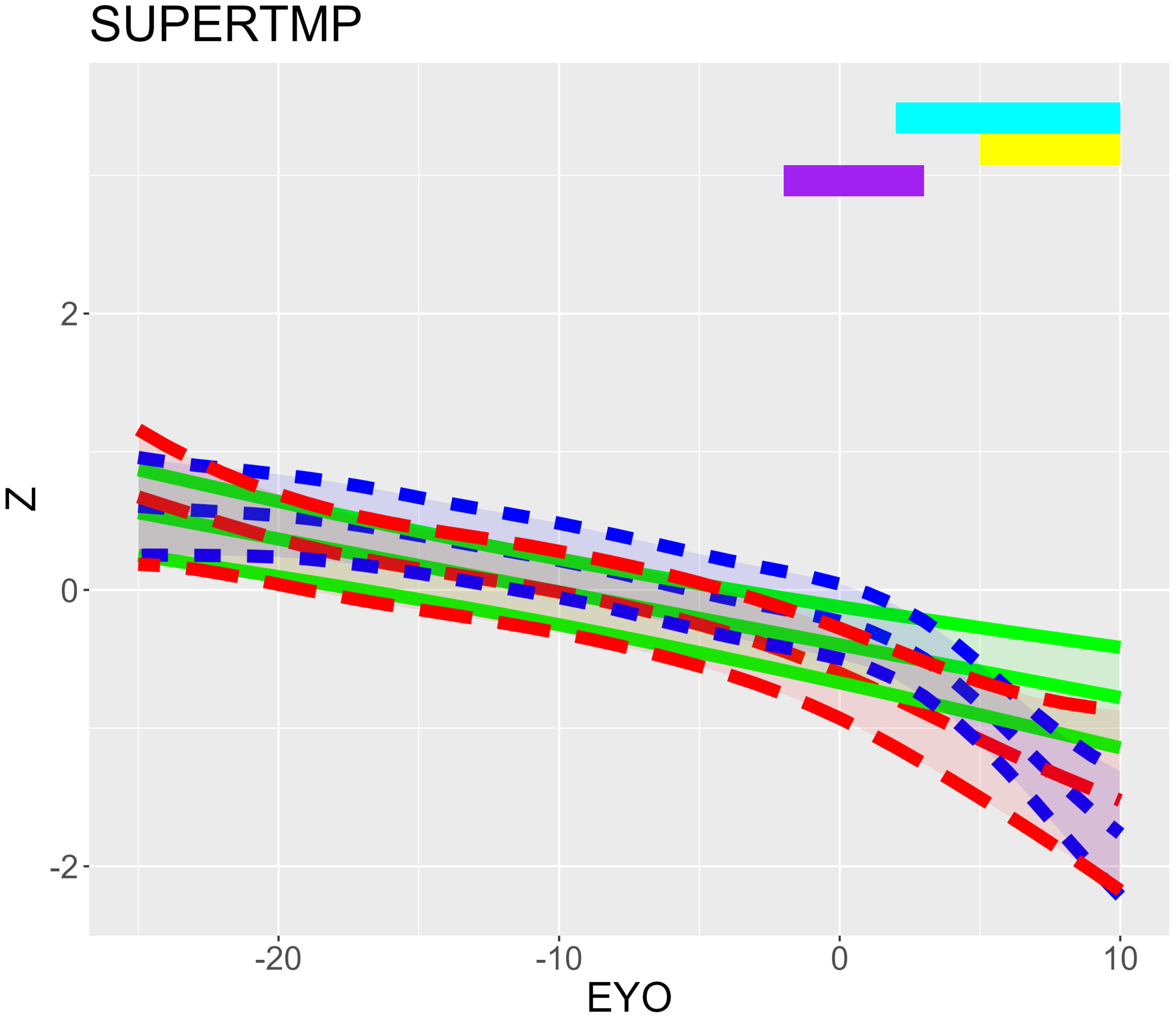

SUPERTMP
Z
2
0
-2
-20
-10
0
10
EYO

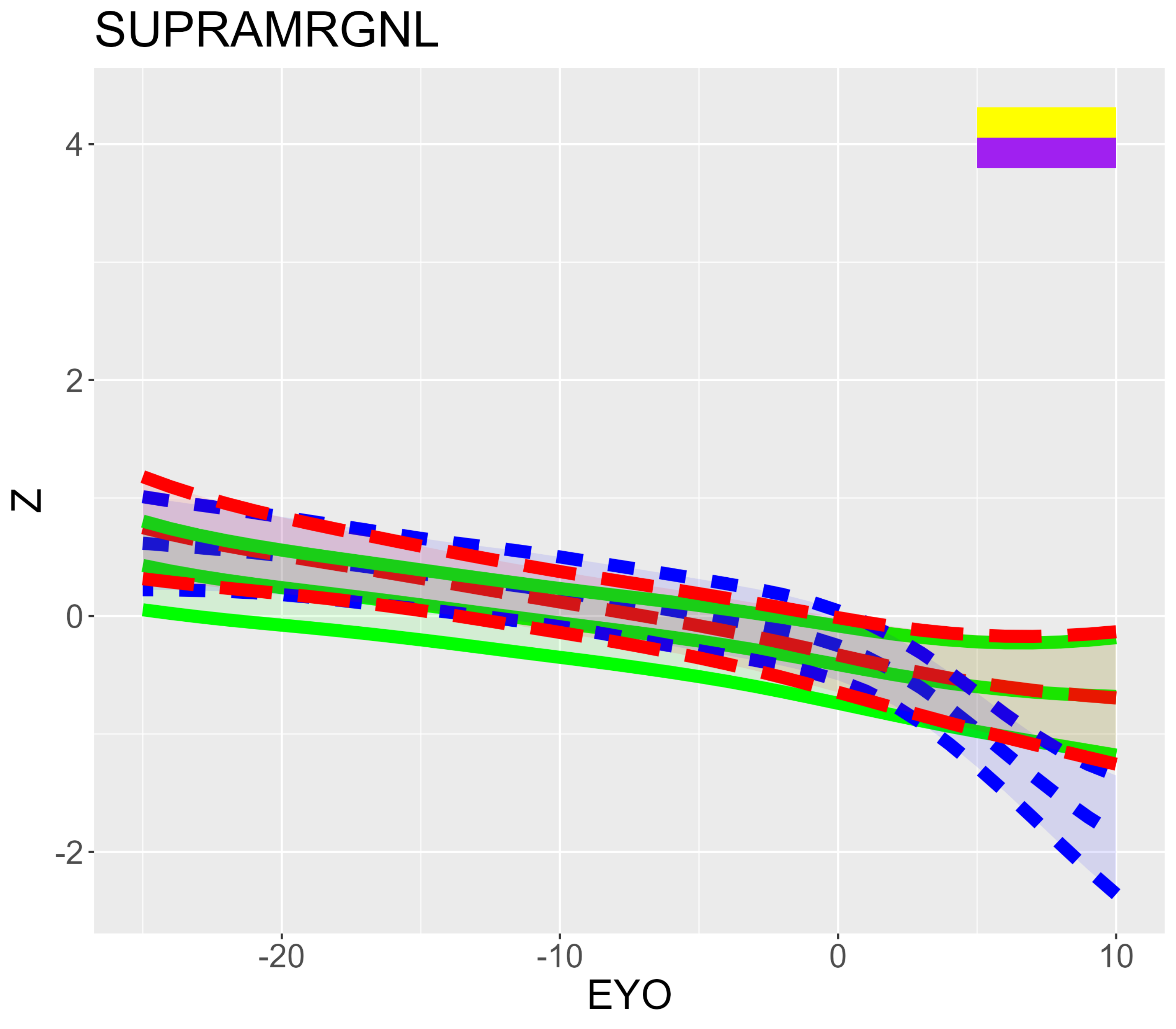
SUPRAMRGNL
4
2
0
-2
Z
-20
-10
0
10
EYO

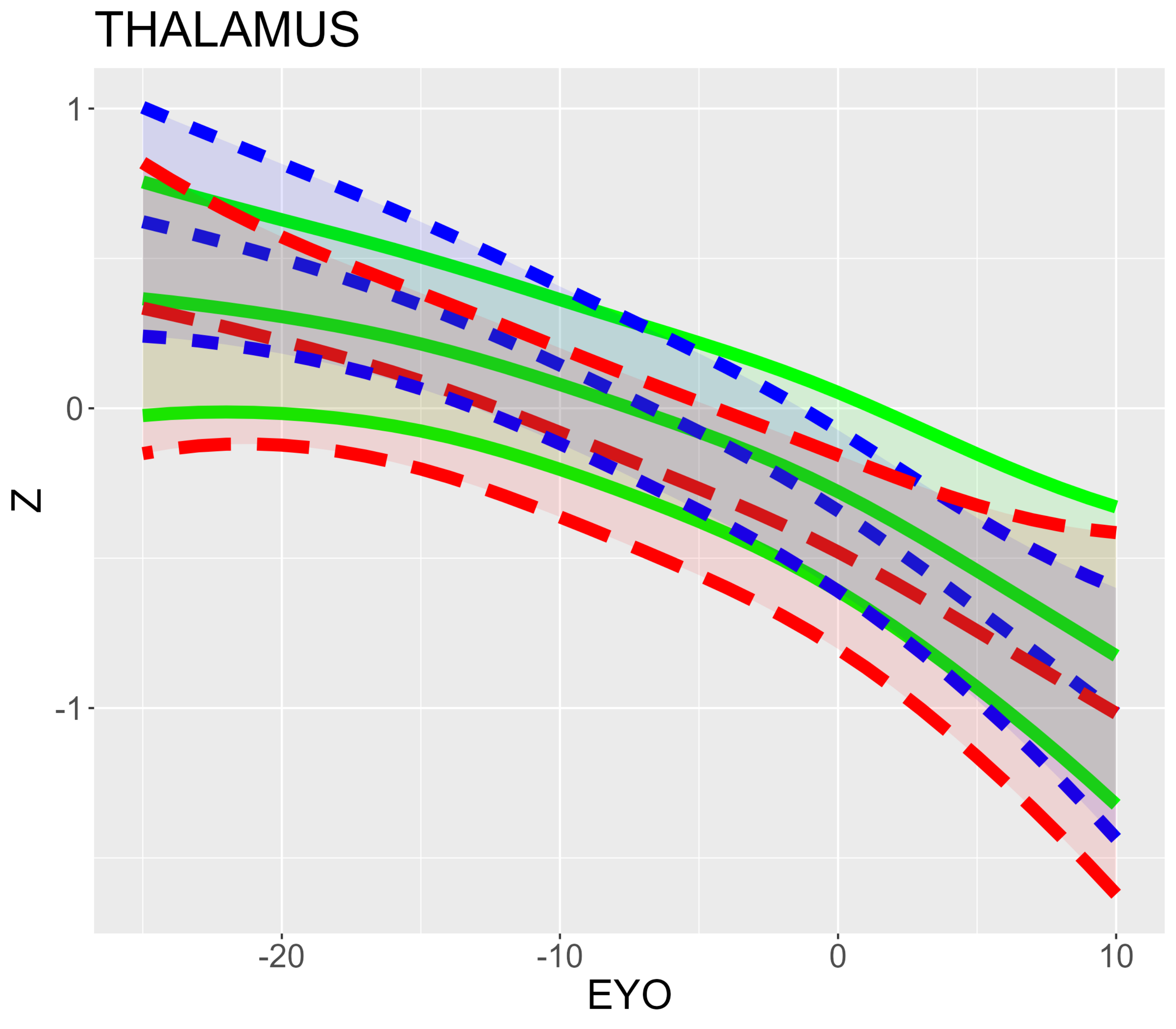
THALAMUS
1
0
-1
Z
-20
-10
0
10
EYO

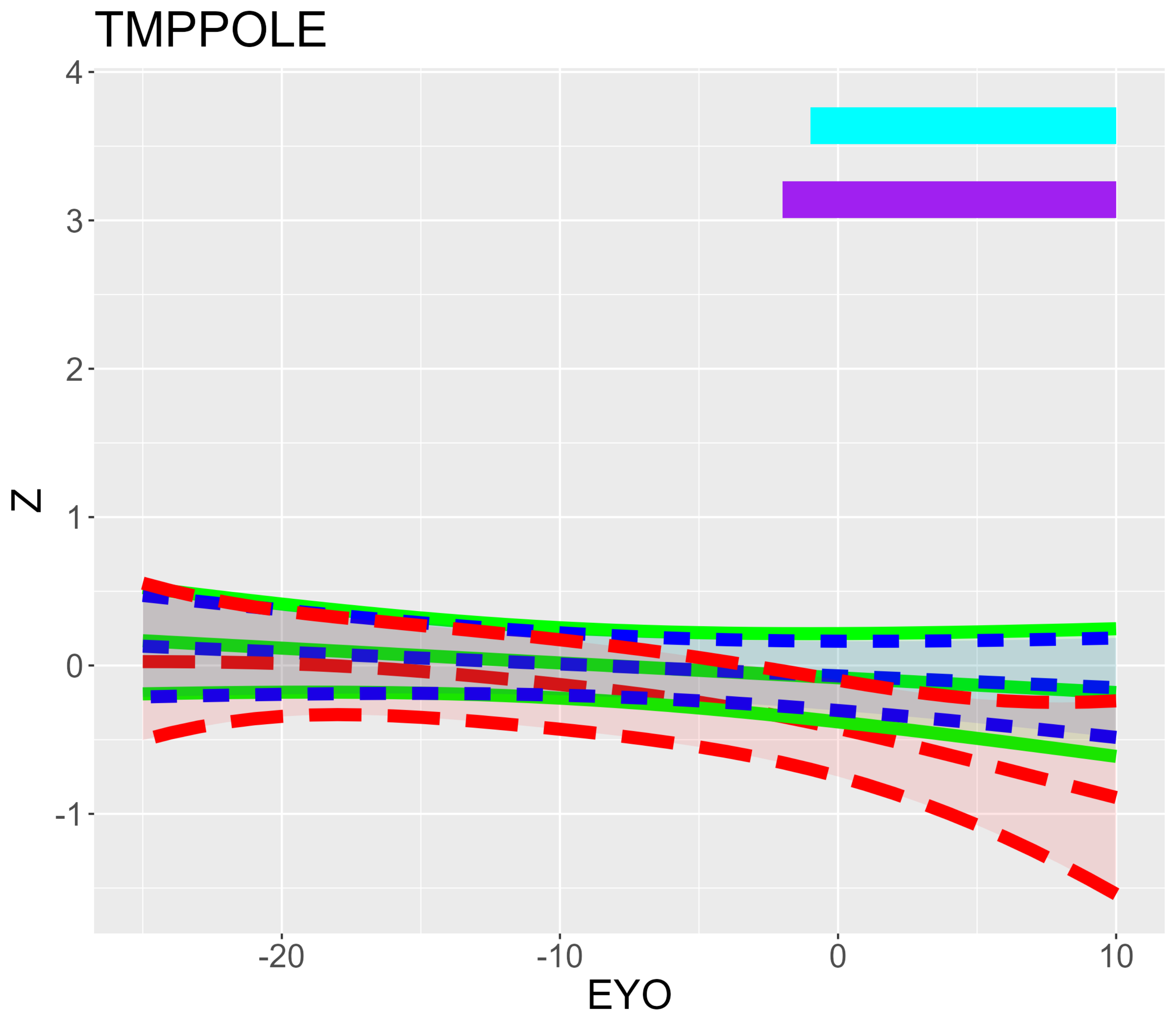
TMPPOLE
4
3
2
1
0
-1
Z
-20
-10
0
10
EYO

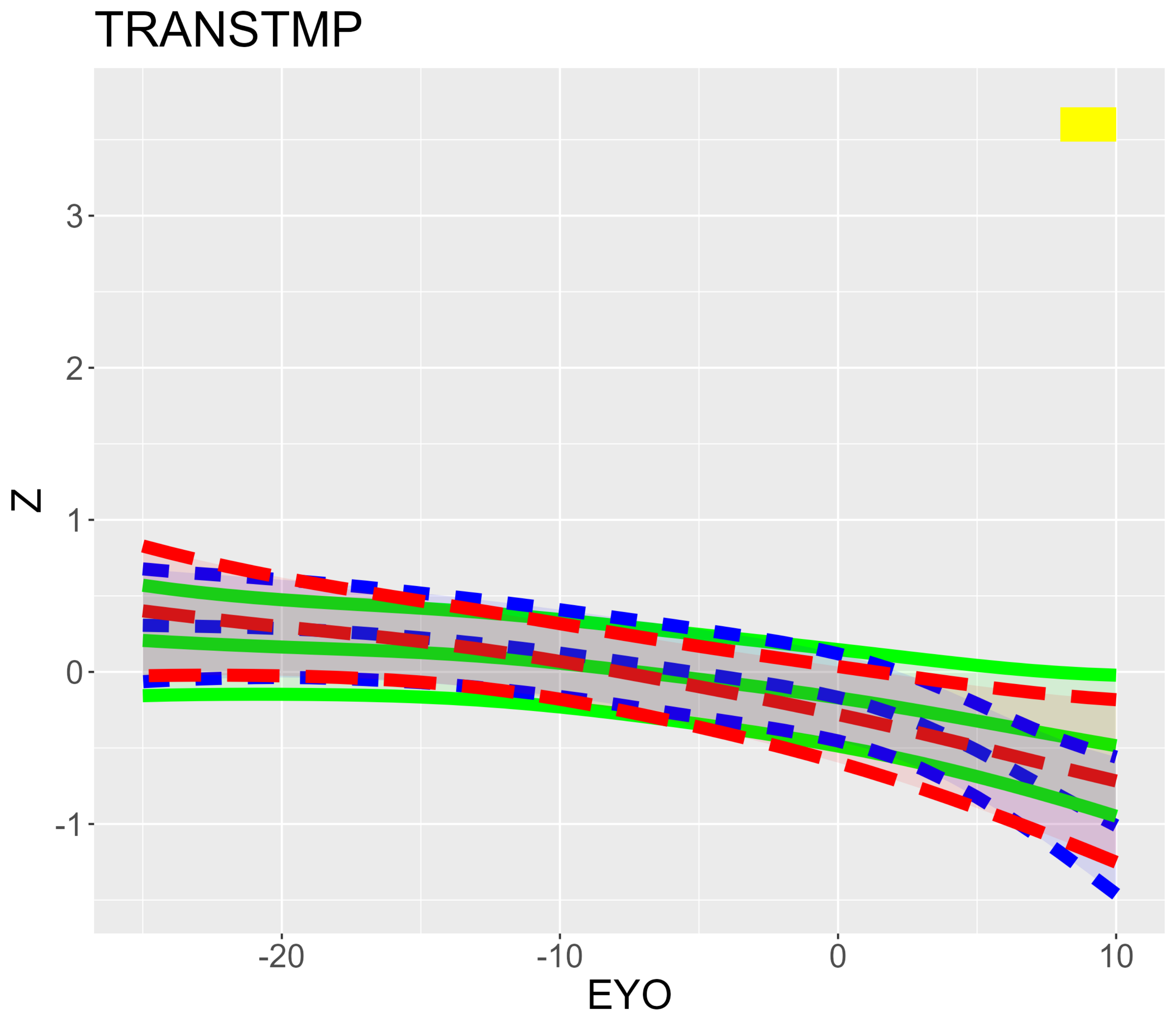

TRANSTMP
Z
EYO
3
2
1
0
-1
-20
-10
0
10

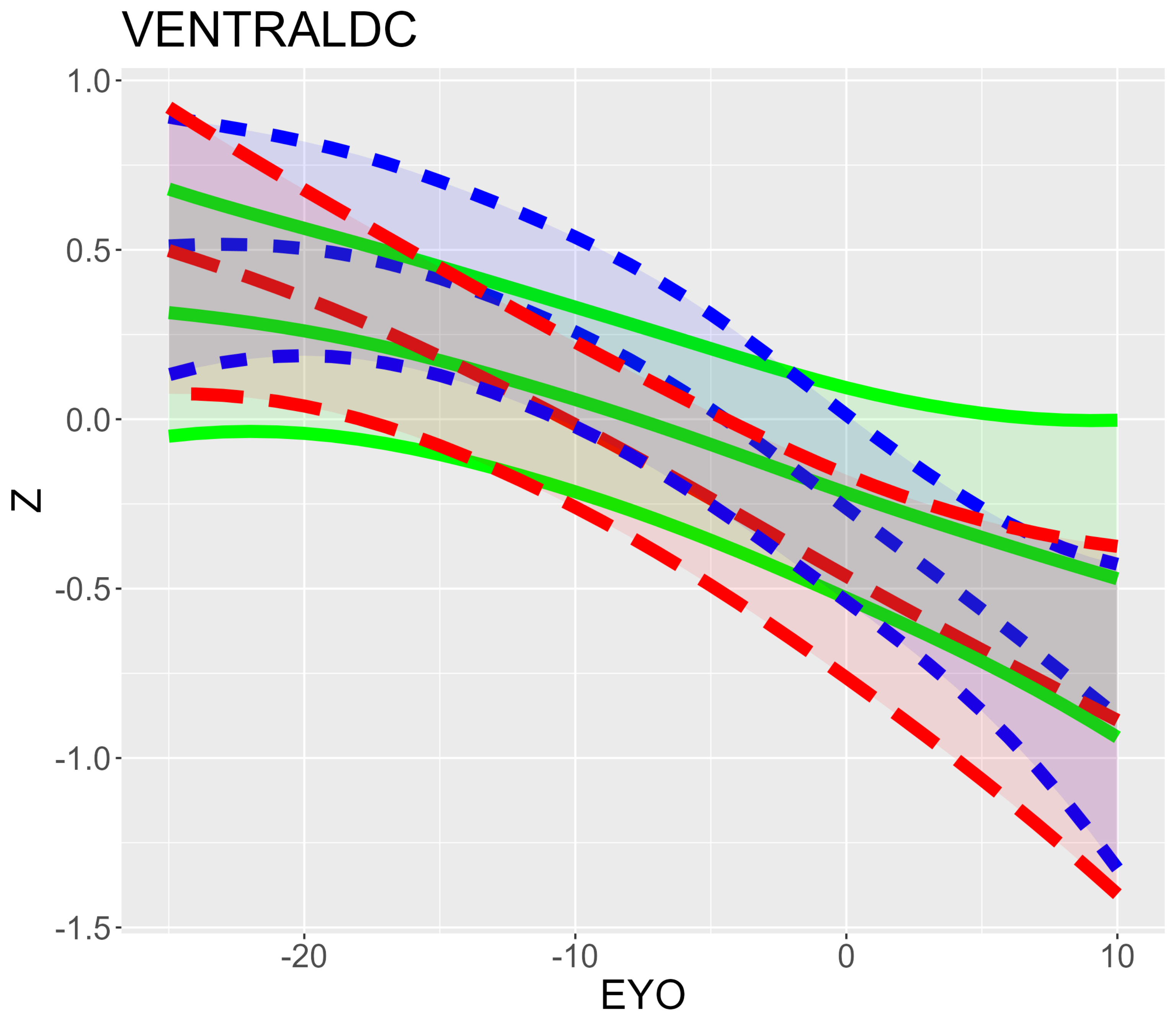
VENTRALDC
1.0
0.5
0.0
-0.5
-1.0
-1.5
Z
-20
-10
0
10
EYO
