# Supplementary material for: Brain volume trajectories in Down syndrome and autosomal dominant Alzheimer's disease
Source: Alzheimers Dement. 2026 Jan 18;22(1):e71103. doi: 10.1002/alz.71103 (PMC12812856; doi:10.1002/alz.71103)
Supplement: Supplementary file 6 — Supporting information [file ALZ-22-e71103-s002.pdf]

ACCUMBENS

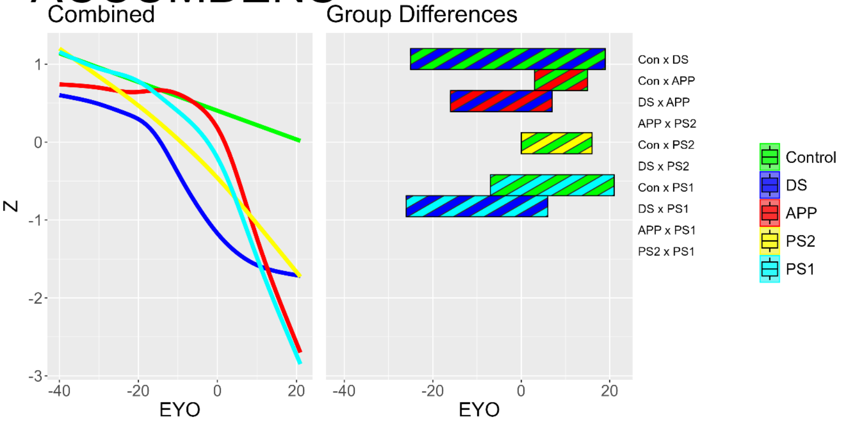

Combined
Group Differences
Z
1
0
-1
-2
-3
-40
-20
0
20
EYO
-40
-20
0
20
EYO
Con x DS
Con x APP
DS x APP
APP x PS2
Con x PS2
DS x PS2
Con x PS1
DS x PS1
APP x PS1
PS2 x PS1
Control
DS
APP
PS2
PS1

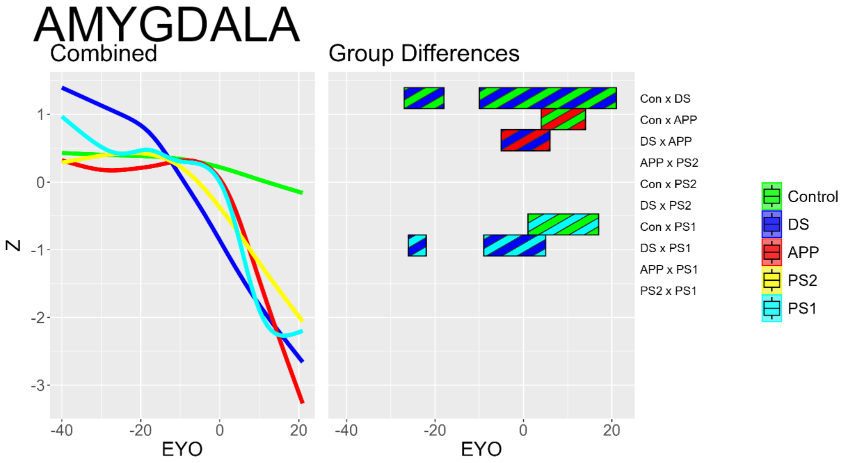
AMYGDALA
Combined
Group Differences
Z
EYO
EYO
1
0
-1
-2
-3
-40
-20
0
20
-40
-20
0
20
Con x DS
Con x APP
DS x APP
APP x PS2
Con x PS2
DS x PS2
Con x PS1
DS x PS1
APP x PS1
PS2 x PS1
Control
DS
APP
PS2
PS1

BRAINSTEM

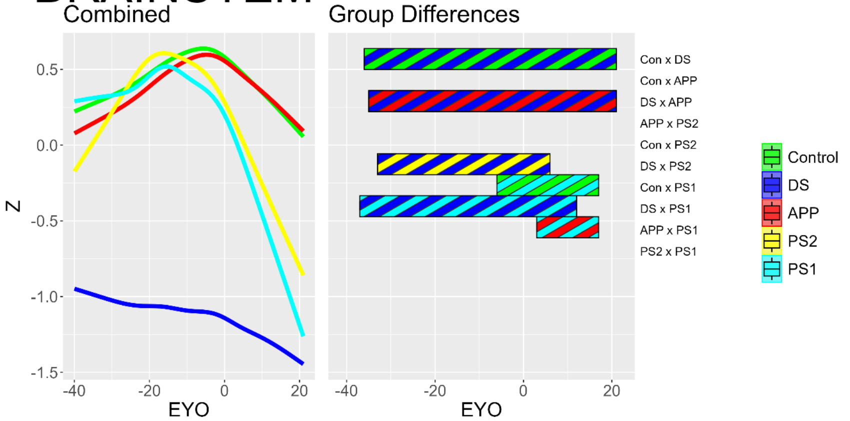

Combined
Group Differences
0.5
0.0
-0.5
-1.0
-1.5
Z
-40
-20
0
20
EYO
-40
-20
0
20
EYO
Con x DS
Con x APP
DS x APP
APP x PS2
Con x PS2
DS x PS2
Con x PS1
DS x PS1
APP x PS1
PS2 x PS1
Control
DS
APP
PS2
PS1

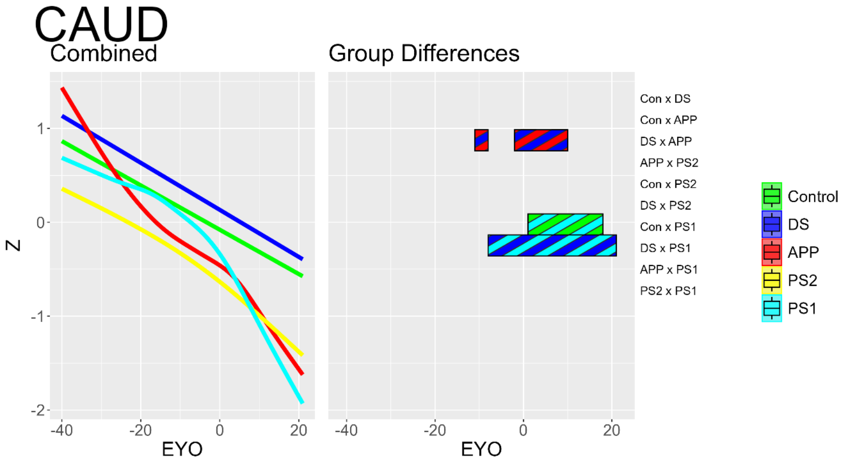

CAUD
Combined
Group Differences
Z
EYO
EYO
1
0
-1
-2
-40
-20
0
20
-40
-20
0
20
Con x DS
Con x APP
DS x APP
APP x PS2
Con x PS2
DS x PS2
Con x PS1
DS x PS1
APP x PS1
PS2 x PS1
Control
DS
APP
PS2
PS1

CAUDANTCNG

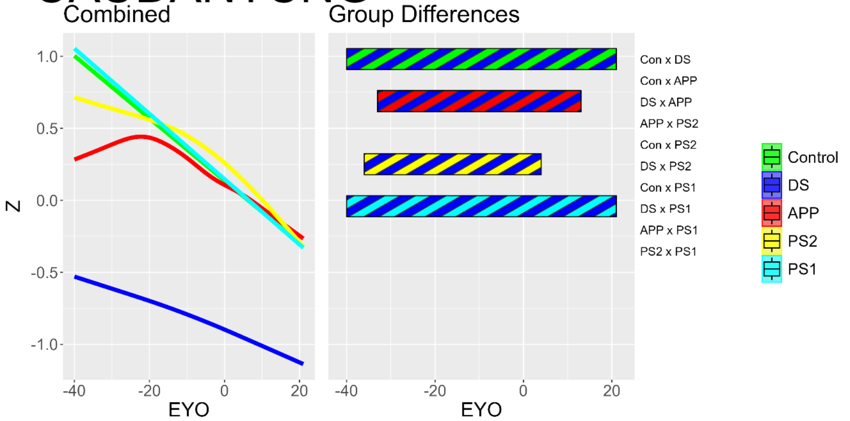
Combined
Group Differences
1.0
0.5
0.0
-0.5
-1.0
Z
-40
-20
0
20
EYO
-40
-20
0
20
EYO
Con x DS
Con x APP
DS x APP
APP x PS2
Con x PS2
DS x PS2
Con x PS1
DS x PS1
APP x PS1
PS2 x PS1
Control
DS
APP
PS2
PS1

CAUDMIDFRN

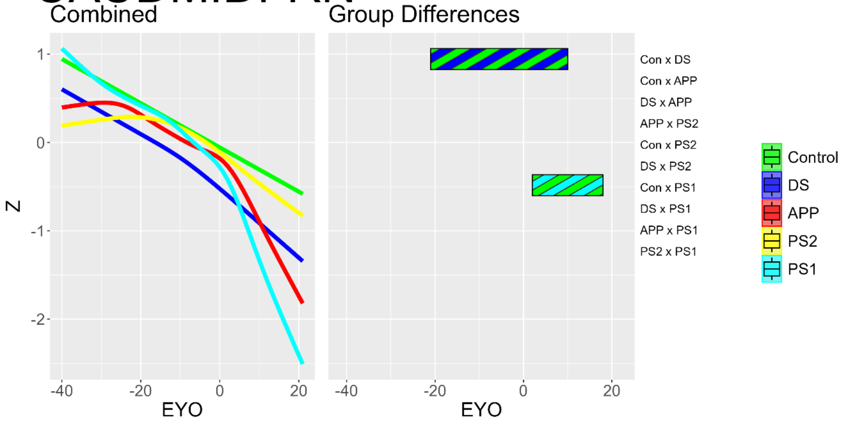

Combined
Group Differences
1
0
-1
-2
Z
-40
-20
0
20
EYO
-40
-20
0
20
EYO
Con x DS
Con x APP
DS x APP
APP x PS2
Con x PS2
DS x PS2
Con x PS1
DS x PS1
APP x PS1
PS2 x PS1
Control
DS
APP
PS2
PS1

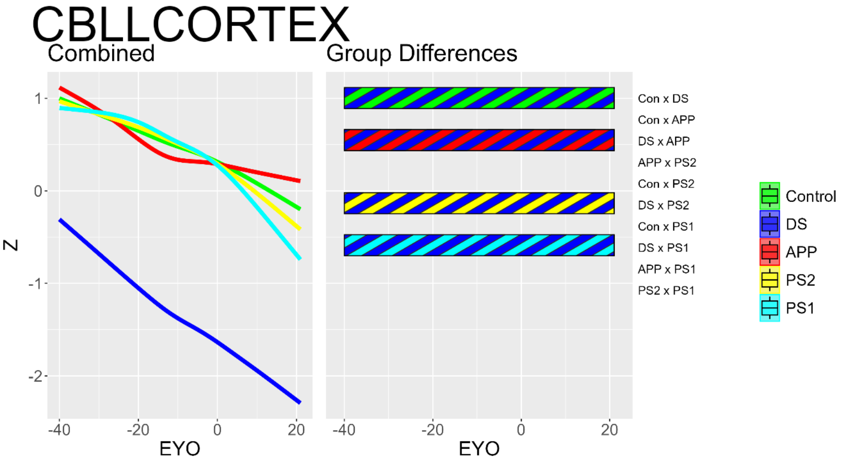

CBLLCORTEX
Combined
Group Differences
1
0
-1
-2
Z
-40
-20
0
20
EYO
-40
-20
0
20
EYO
Con x DS
Con x APP
DS x APP
APP x PS2
Con x PS2
DS x PS2
Con x PS1
DS x PS1
APP x PS1
PS2 x PS1
Control
DS
APP
PS2
PS1

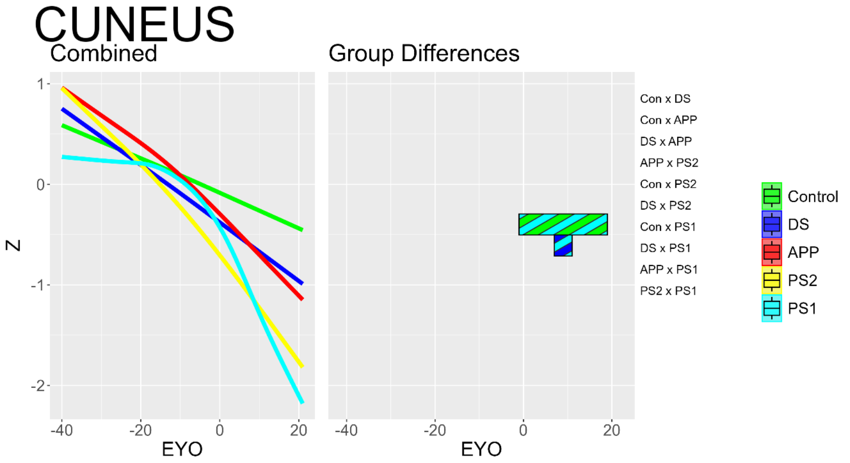
CUNEUS
Combined
Group Differences
1
0
-1
-2
Z
-40
-20
0
20
EYO
-40
-20
0
20
EYO
Con x DS
Con x APP
DS x APP
APP x PS2
Con x PS2
DS x PS2
Con x PS1
DS x PS1
APP x PS1
PS2 x PS1
Control
DS
APP
PS2
PS1

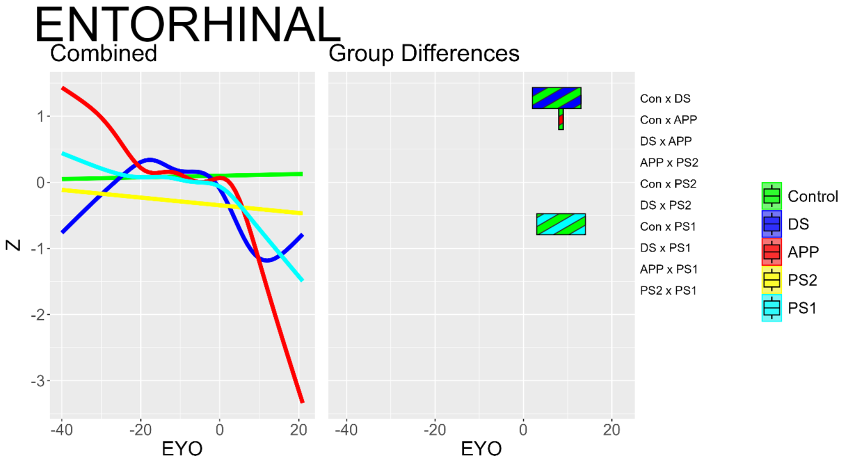
ENTORHINAL
Combined
Group Differences
Z
1
0
-1
-2
-3
-40
-20
0
20
EYO
-40
-20
0
20
EYO
Con x DS
Con x APP
DS x APP
APP x PS2
Con x PS2
DS x PS2
Con x PS1
DS x PS1
APP x PS1
PS2 x PS1
Control
DS
APP
PS2
PS1

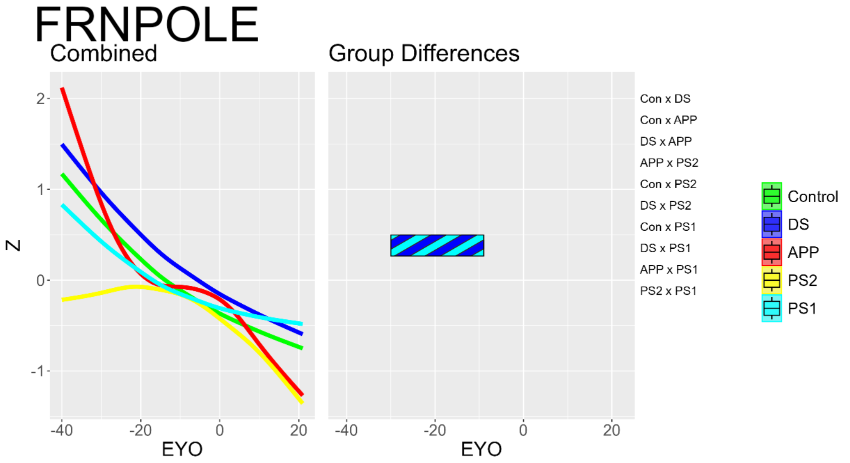

FRNPOLE
Combined
Group Differences
2
1
0
-1
Z
-40
-20
0
20
EYO
Con x DS
Con x APP
DS x APP
APP x PS2
Con x PS2
DS x PS2
Con x PS1
DS x PS1
APP x PS1
PS2 x PS1
Control
DS
APP
PS2
PS1

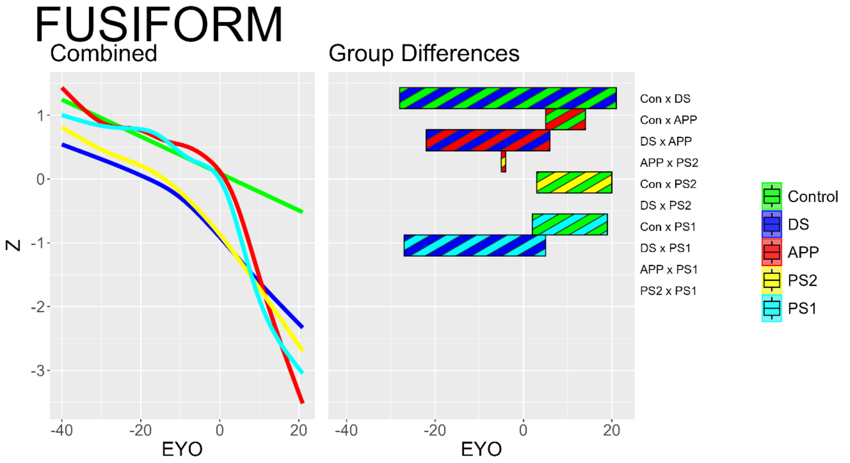

FUSIFORM
Combined
Group Differences
Z
EYO
EYO
1
0
-1
-2
-3
-40
-20
0
20
-40
-20
0
20
Con x DS
Con x APP
DS x APP
APP x PS2
Con x PS2
DS x PS2
Con x PS1
DS x PS1
APP x PS1
PS2 x PS1
Control
DS
APP
PS2
PS1

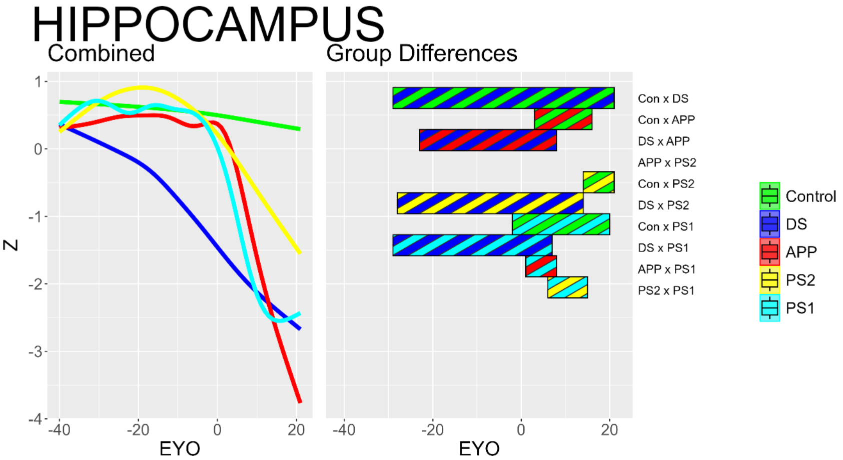
HIPPOCAMPUS
Combined
Group Differences
Z
EYO
EYO
1
0
-1
-2
-3
-4
-40
-20
0
20
-40
-20
0
20
Con x DS
Con x APP
DS x APP
APP x PS2
Con x PS2
DS x PS2
Con x PS1
DS x PS1
APP x PS1
PS2 x PS1
Control
DS
APP
PS2
PS1

INFRPRTL

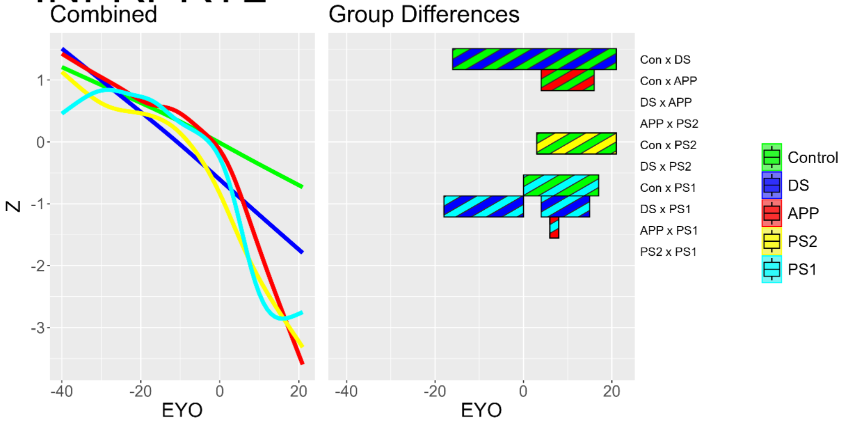

Combined
Group Differences
1
0
-1
-2
-3
Z
-40
-20
0
20
EYO
-40
-20
0
20
EYO
Con x DS
Con x APP
DS x APP
APP x PS2
Con x PS2
DS x PS2
Con x PS1
DS x PS1
APP x PS1
PS2 x PS1
Control
DS
APP
PS2
PS1

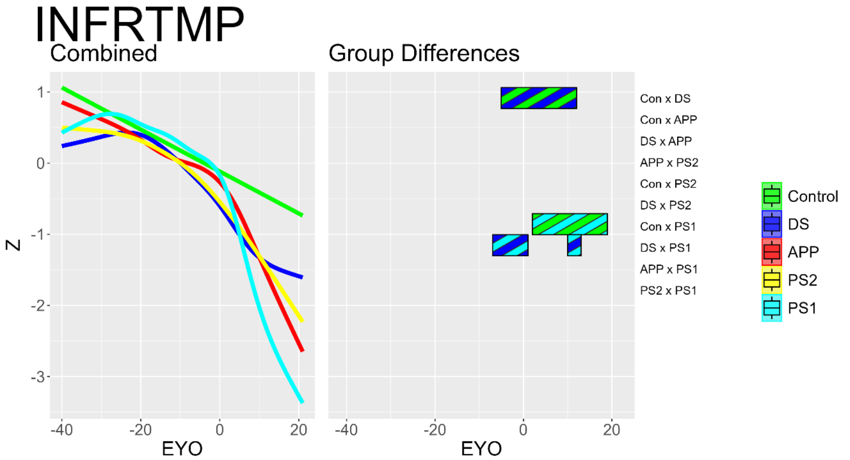

INFRTMP
Combined
Group Differences
Z
EYO
EYO
1
0
-1
-2
-3
-40
-20
0
20
-40
-20
0
20
Con x DS
Con x APP
DS x APP
APP x PS2
Con x PS2
DS x PS2
Con x PS1
DS x PS1
APP x PS1
PS2 x PS1
Control
DS
APP
PS2
PS1

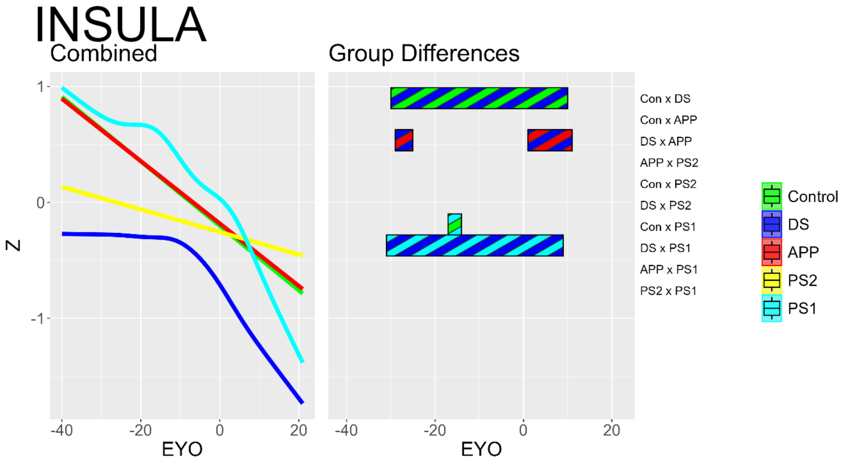
INSULA
Combined
Group Differences
1
0
-1
Z
-40
-20
0
20
EYO
-40
-20
0
20
EYO
Con x DS
Con x APP
DS x APP
APP x PS2
Con x PS2
DS x PS2
Con x PS1
DS x PS1
APP x PS1
PS2 x PS1
Control
DS
APP
PS2
PS1

ISTHMUSCNG

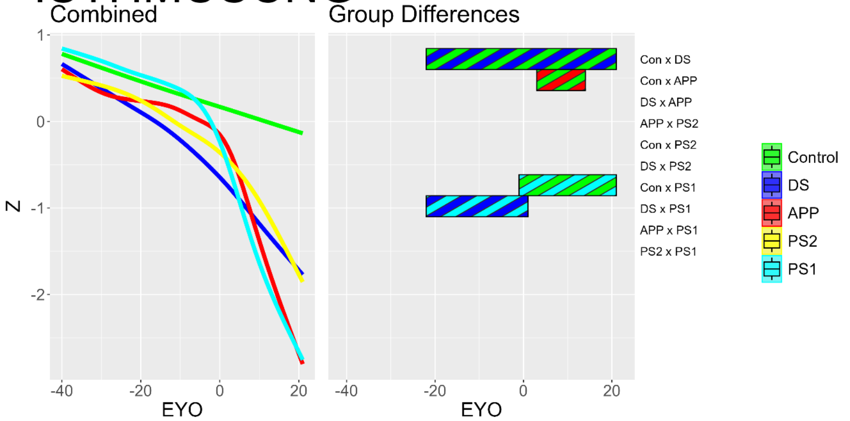
Combined
Group Differences
1
0
-1
-2
Z
-40
-20
0
20
EYO
-40
-20
0
20
EYO
Con x DS
Con x APP
DS x APP
APP x PS2
Con x PS2
DS x PS2
Con x PS1
DS x PS1
APP x PS1
PS2 x PS1
Control
DS
APP
PS2
PS1

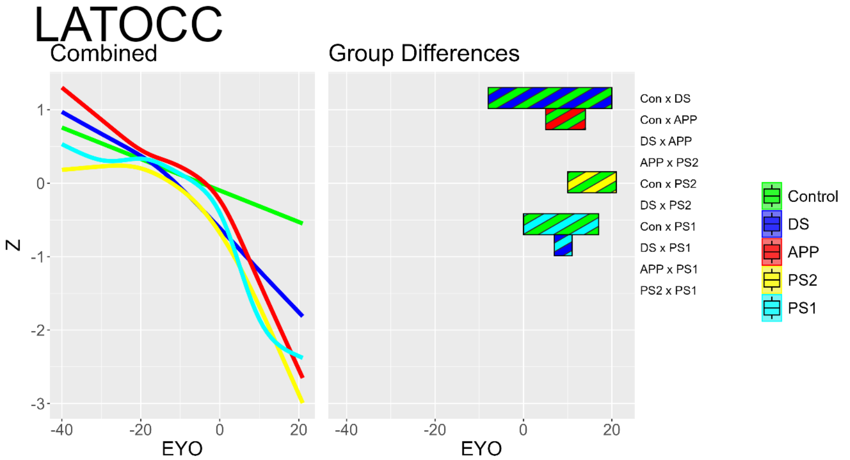

LATOCC
Combined
Group Differences
1
0
-1
-2
-3
Z
-40
-20
0
20
EYO
-40
-20
0
20
EYO
Con x DS
Con x APP
DS x APP
APP x PS2
Con x PS2
DS x PS2
Con x PS1
DS x PS1
APP x PS1
PS2 x PS1
Control
DS
APP
PS2
PS1

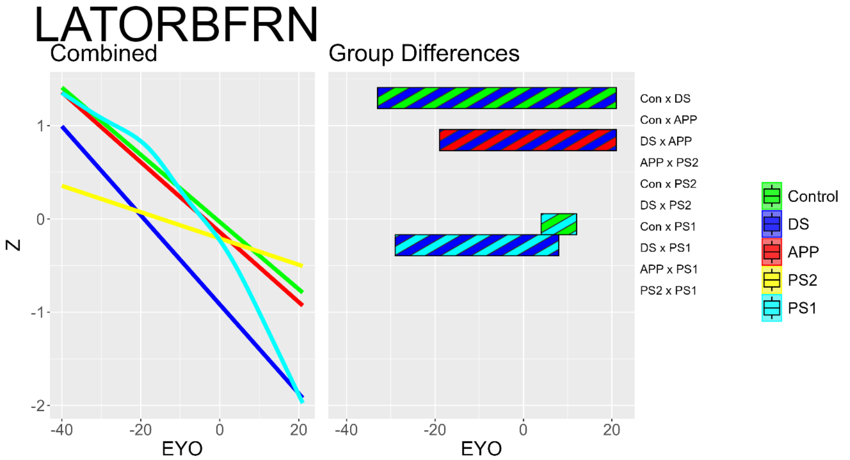

LATORBFRN
Combined
Group Differences
Z
EYO
EYO
1
0
-1
-2
-40
-20
0
20
-40
-20
0
20
Con x DS
Con x APP
DS x APP
APP x PS2
Con x PS2
DS x PS2
Con x PS1
DS x PS1
APP x PS1
PS2 x PS1
Control
DS
APP
PS2
PS1

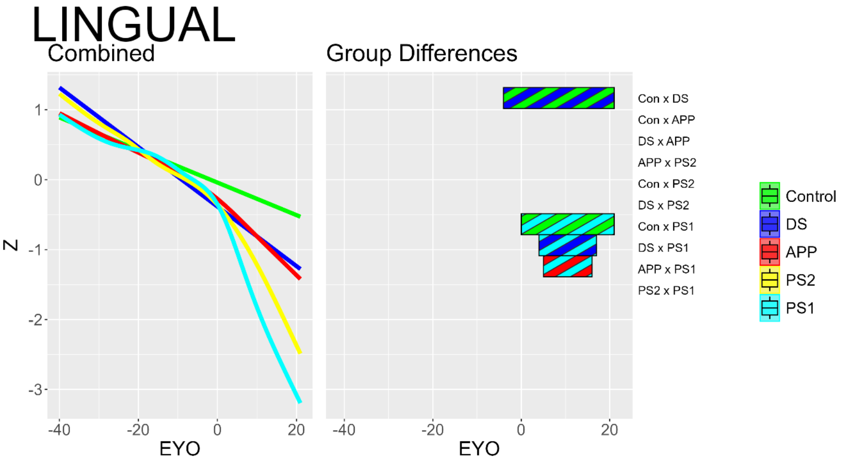
LINGUAL
Combined
Group Differences
Z
1
0
-1
-2
-3
-40
-20
0
20
EYO
-40
-20
0
20
EYO
Con x DS
Con x APP
DS x APP
APP x PS2
Con x PS2
DS x PS2
Con x PS1
DS x PS1
APP x PS1
PS2 x PS1
Control
DS
APP
PS2
PS1

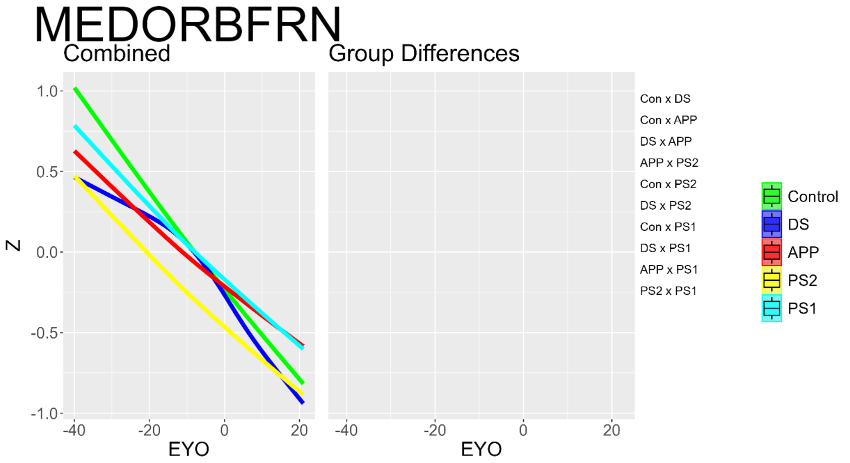
MEDORBFRN
Combined
Group Differences
1.0
0.5
0.0
-0.5
-1.0
Z
-40
-20
0
20
EYO
-40
-20
0
20
EYO
Con x DS
Con x APP
DS x APP
APP x PS2
Con x PS2
DS x PS2
Con x PS1
DS x PS1
APP x PS1
PS2 x PS1
Control
DS
APP
PS2
PS1

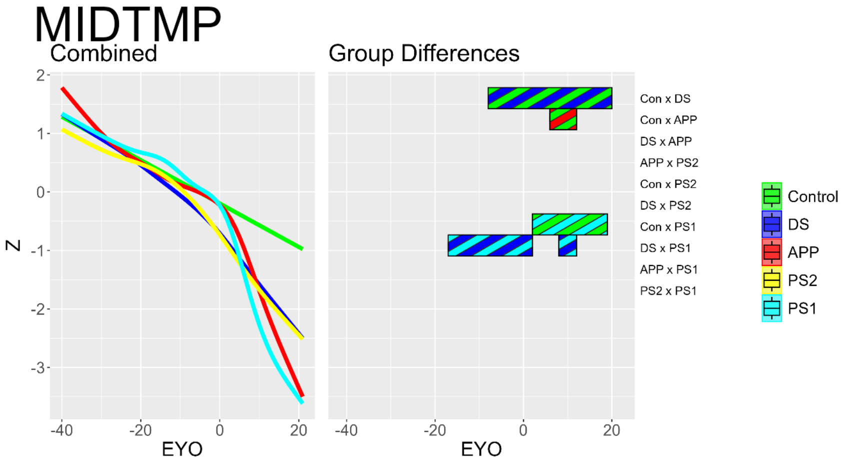
MIDTMP
Combined
Group Differences
Z
EYO
EYO
2
1
0
-1
-2
-3
-40
-20
0
20
-40
-20
0
20
Con x DS
Con x APP
DS x APP
APP x PS2
Con x PS2
DS x PS2
Con x PS1
DS x PS1
APP x PS1
PS2 x PS1
Control
DS
APP
PS2
PS1

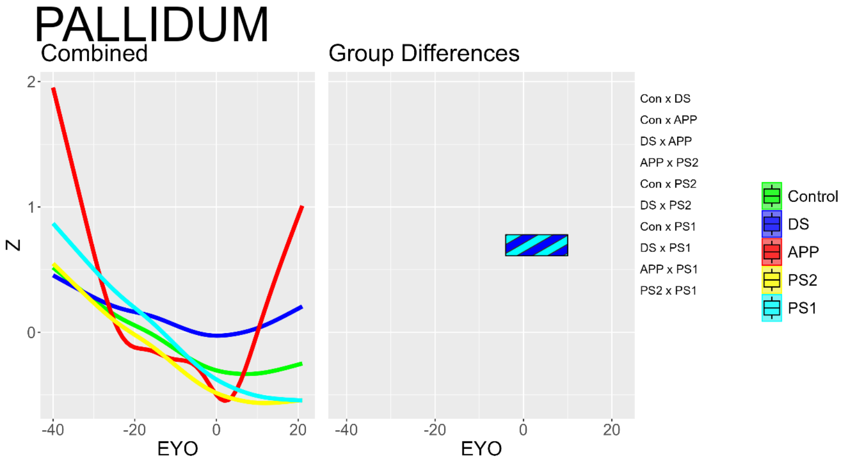
PALLIDUM
Combined
Group Differences
2
1
0
Z
-40
-20
0
20
EYO
-40
-20
0
20
EYO
Con x DS
Con x APP
DS x APP
APP x PS2
Con x PS2
DS x PS2
Con x PS1
DS x PS1
APP x PS1
PS2 x PS1
Control
DS
APP
PS2
PS1

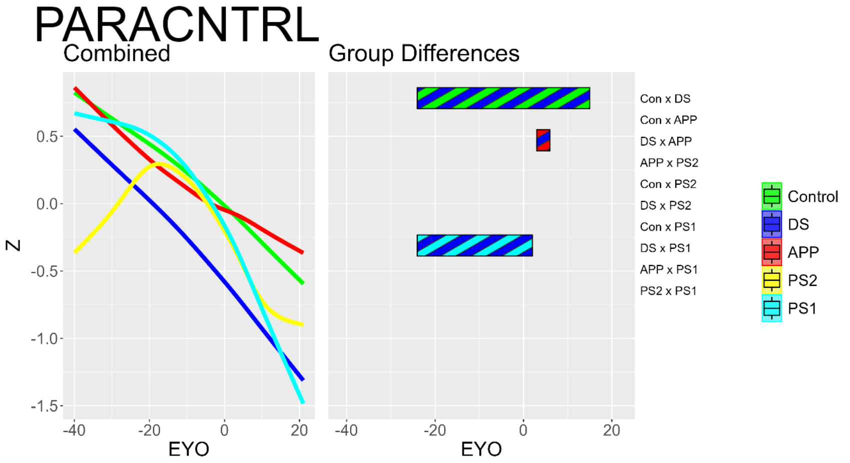
PARACNTRL
Combined
Group Differences
Z
EYO
EYO
0.5
0.0
-0.5
-1.0
-1.5
-40
-20
0
20
-40
-20
0
20
Con x DS
Con x APP
DS x APP
APP x PS2
Con x PS2
DS x PS2
Con x PS1
DS x PS1
APP x PS1
PS2 x PS1
Control
DS
APP
PS2
PS1

PARAHPCMPL

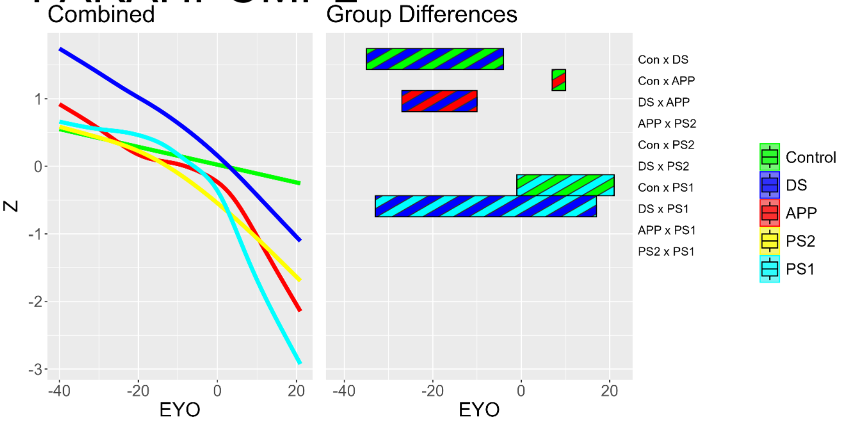

Combined
Group Differences
Z
1
0
-1
-2
-3
-40
-20
0
20
EYO
-40
-20
0
20
EYO
Con x DS
Con x APP
DS x APP
APP x PS2
Con x PS2
DS x PS2
Con x PS1
DS x PS1
APP x PS1
PS2 x PS1
Control
DS
APP
PS2
PS1

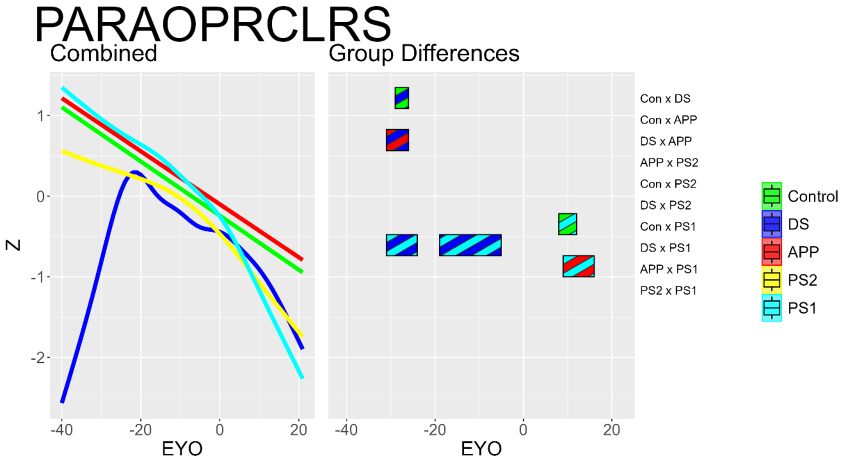
PARAOPRCLRS
Combined
Group Differences
Z
1
0
-1
-2
-40
-20
0
20
EYO
-40
-20
0
20
EYO
Con x DS
Con x APP
DS x APP
APP x PS2
Con x PS2
DS x PS2
Con x PS1
DS x PS1
APP x PS1
PS2 x PS1
Control
DS
APP
PS2
PS1

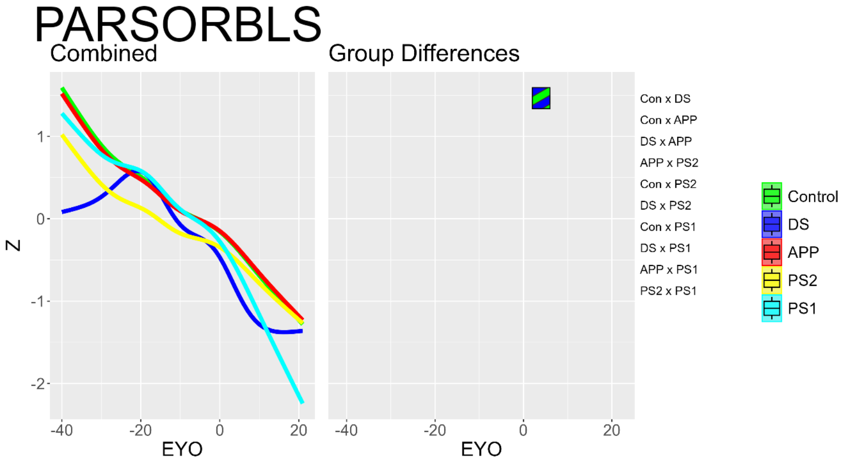
PARSORBLS
Combined
Group Differences
Z
1
0
-1
-2
-40
-20
0
20
EYO
-40
-20
0
20
EYO
Con x DS
Con x APP
DS x APP
APP x PS2
Con x PS2
DS x PS2
Con x PS1
DS x PS1
APP x PS1
PS2 x PS1
Control
DS
APP
PS2
PS1

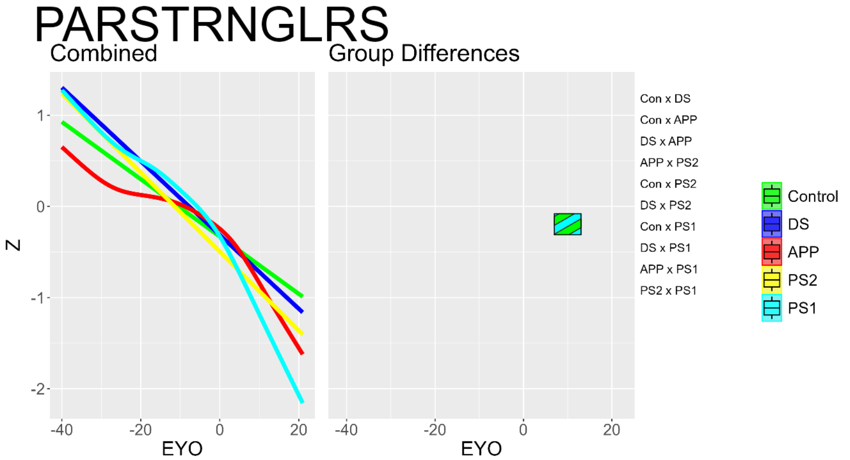
PARSTRNGLRS
Combined
Group Differences
Z
EYO
EYO
1
0
-1
-2
-40
-20
0
20
-40
-20
0
20
Con x DS
Con x APP
DS x APP
APP x PS2
Con x PS2
DS x PS2
Con x PS1
DS x PS1
APP x PS1
PS2 x PS1
Control
DS
APP
PS2
PS1

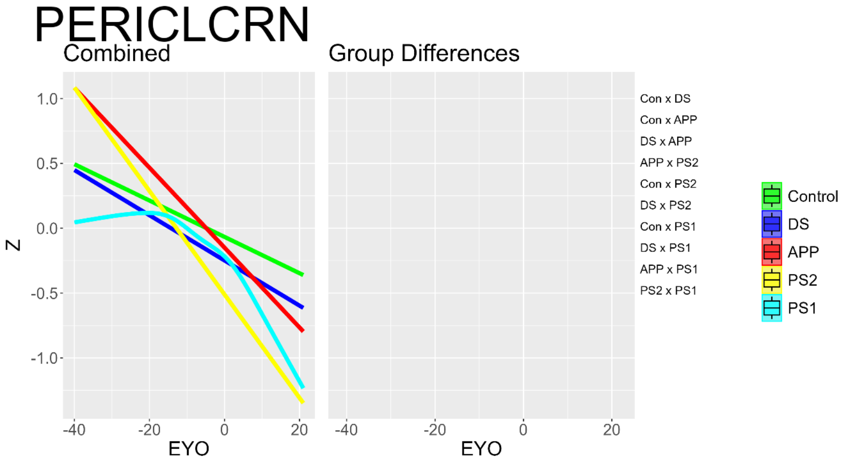

PERICLCRN
Combined
Group Differences
1.0
0.5
0.0
-0.5
-1.0
Z
-40
-20
0
20
EYO
Con x DS
Con x APP
DS x APP
APP x PS2
Con x PS2
DS x PS2
Con x PS1
DS x PS1
APP x PS1
PS2 x PS1
Control
DS
APP
PS2
PS1

POSTCNG

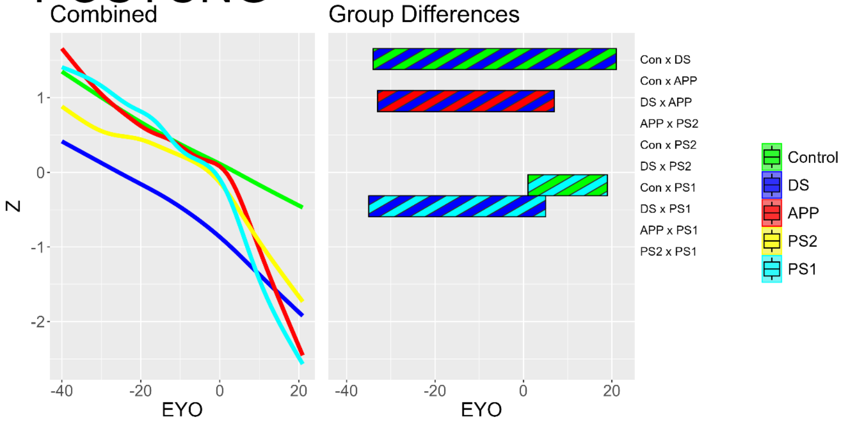
Combined
Group Differences
Z
1
0
-1
-2
-40
-20
0
20
EYO
Con x DS
Con x APP
DS x APP
APP x PS2
Con x PS2
DS x PS2
Con x PS1
DS x PS1
APP x PS1
PS2 x PS1
Control
DS
APP
PS2
PS1

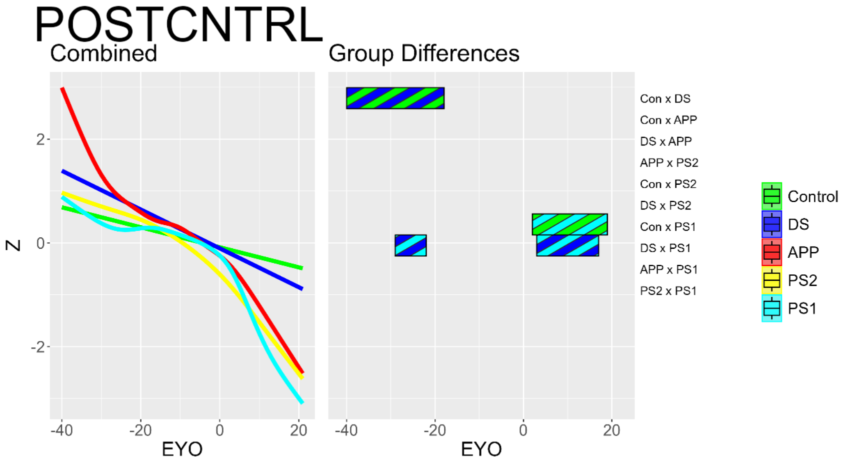

POSTCNTRL
Combined
Group Differences
Z
EYO
EYO
2
0
-2
-40
-20
0
20
-40
-20
0
20
Con x DS
Con x APP
DS x APP
APP x PS2
Con x PS2
DS x PS2
Con x PS1
DS x PS1
APP x PS1
PS2 x PS1
Control
DS
APP
PS2
PS1

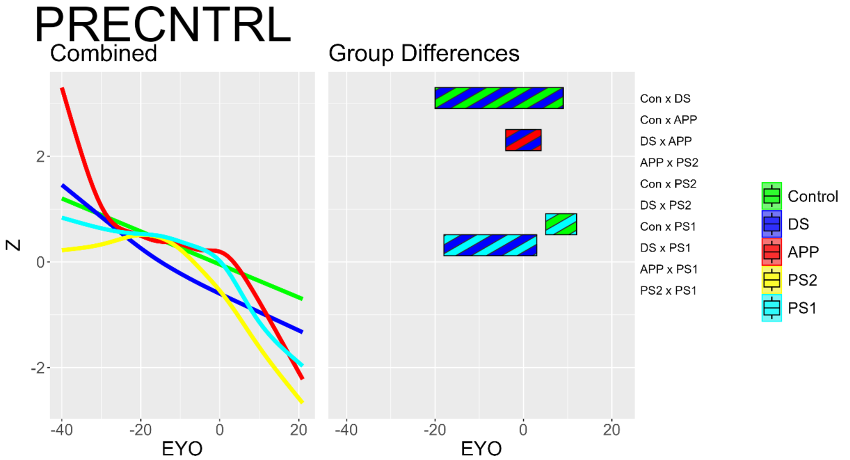
PRECNTRL
Combined
Group Differences
Z
2
0
-2
-40
-20
0
20
EYO
Con x DS
Con x APP
DS x APP
APP x PS2
Con x PS2
DS x PS2
Con x PS1
DS x PS1
APP x PS1
PS2 x PS1
Control
DS
APP
PS2
PS1

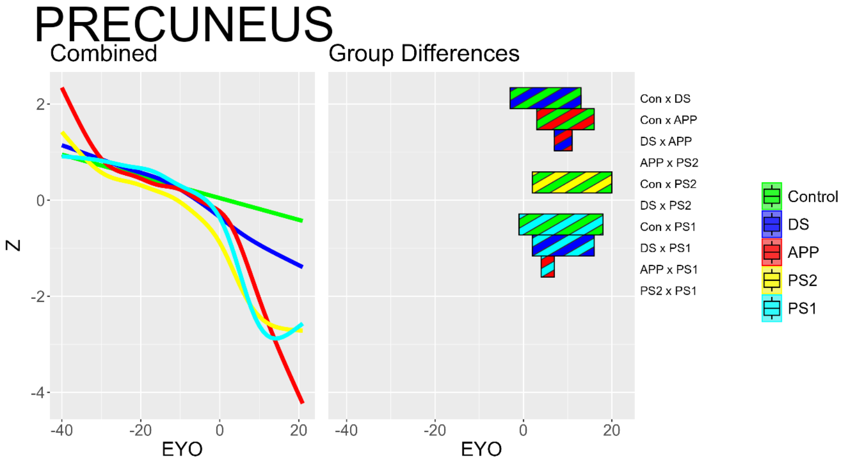
PRECUNEUS
Combined
Group Differences
2
0
-2
-4
Z
-40
-20
0
20
EYO
-40
-20
0
20
EYO
Con x DS
Con x APP
DS x APP
APP x PS2
Con x PS2
DS x PS2
Con x PS1
DS x PS1
APP x PS1
PS2 x PS1
Control
DS
APP
PS2
PS1

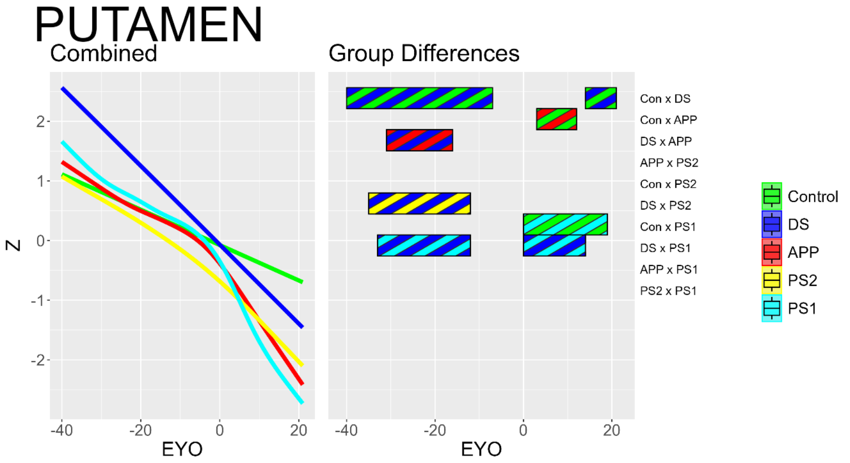
PUTAMEN
Combined
Group Differences
Z
2
1
0
-1
-2
-40
-20
0
20
EYO
-40
-20
0
20
EYO
Con x DS
Con x APP
DS x APP
APP x PS2
Con x PS2
DS x PS2
Con x PS1
DS x PS1
APP x PS1
PS2 x PS1
Control
DS
APP
PS2
PS1

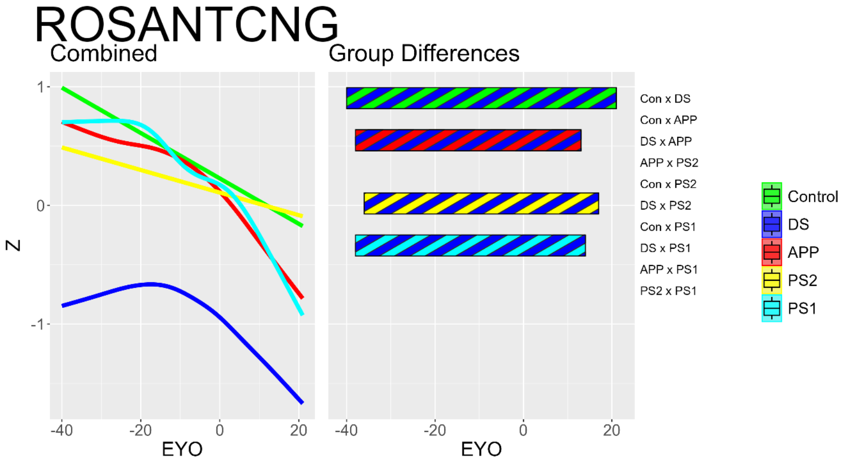
ROSANTCNG
Combined
Group Differences
1
0
-1
Z
-40
-20
0
20
EYO
-40
-20
0
20
EYO
Con x DS
Con x APP
DS x APP
APP x PS2
Con x PS2
DS x PS2
Con x PS1
DS x PS1
APP x PS1
PS2 x PS1
Control
DS
APP
PS2
PS1

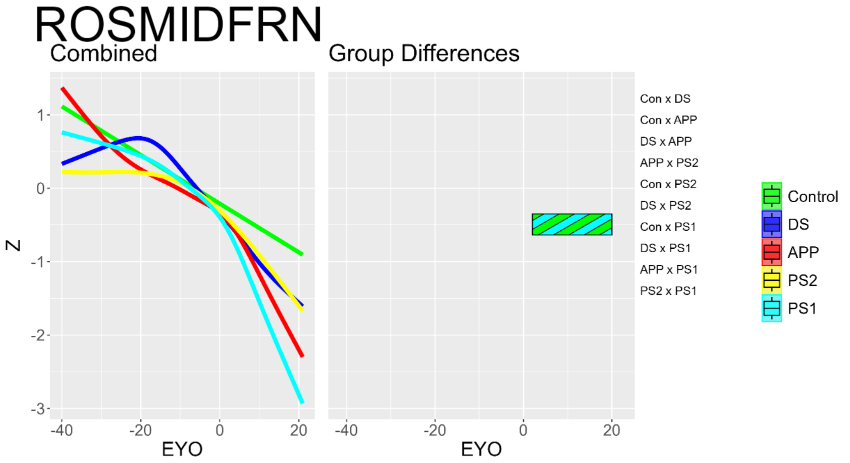
ROSMIDFRN
Combined
Group Differences
Z
EYO
EYO
1
0
-1
-2
-3
-40
-20
0
20
-40
-20
0
20
Con x DS
Con x APP
DS x APP
APP x PS2
Con x PS2
DS x PS2
Con x PS1
DS x PS1
APP x PS1
PS2 x PS1
Control
DS
APP
PS2
PS1

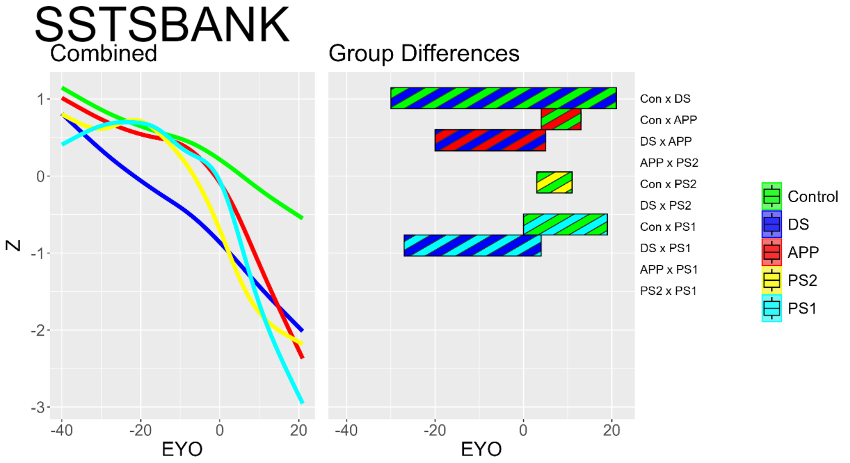
SSTSBANK
Combined
Group Differences
1
0
-1
-2
-3
Z
-40
-20
0
20
EYO
-40
-20
0
20
EYO
Con x DS
Con x APP
DS x APP
APP x PS2
Con x PS2
DS x PS2
Con x PS1
DS x PS1
APP x PS1
PS2 x PS1
Control
DS
APP
PS2
PS1

SUPERFRN

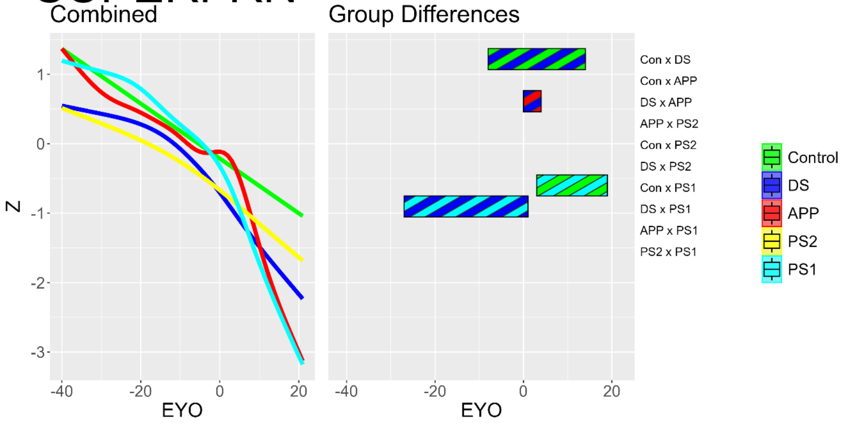

Combined
Group Differences
Z
EYO
EYO
1
0
-1
-2
-3
-40
-20
0
20
-40
-20
0
20
Con x DS
Con x APP
DS x APP
APP x PS2
Con x PS2
DS x PS2
Con x PS1
DS x PS1
APP x PS1
PS2 x PS1
Control
DS
APP
PS2
PS1

SUPERPRTL

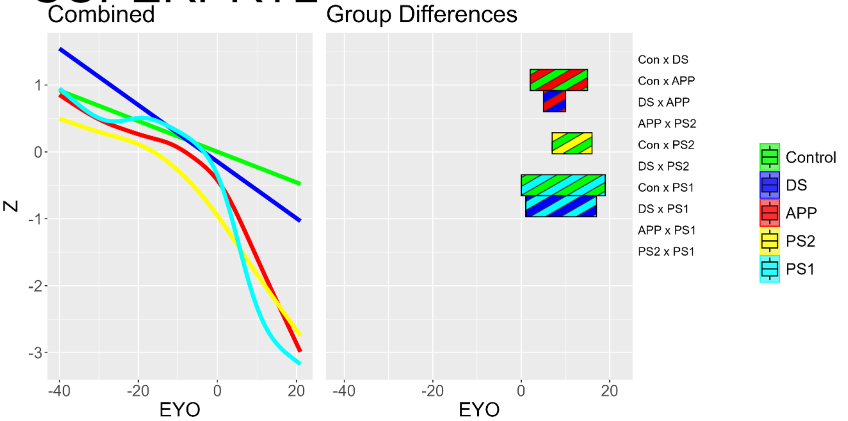

Combined
Group Differences
1
0
-1
-2
-3
Z
-40
-20
0
20
EYO
-40
-20
0
20
EYO
Con x DS
Con x APP
DS x APP
APP x PS2
Con x PS2
DS x PS2
Con x PS1
DS x PS1
APP x PS1
PS2 x PS1
Control
DS
APP
PS2
PS1

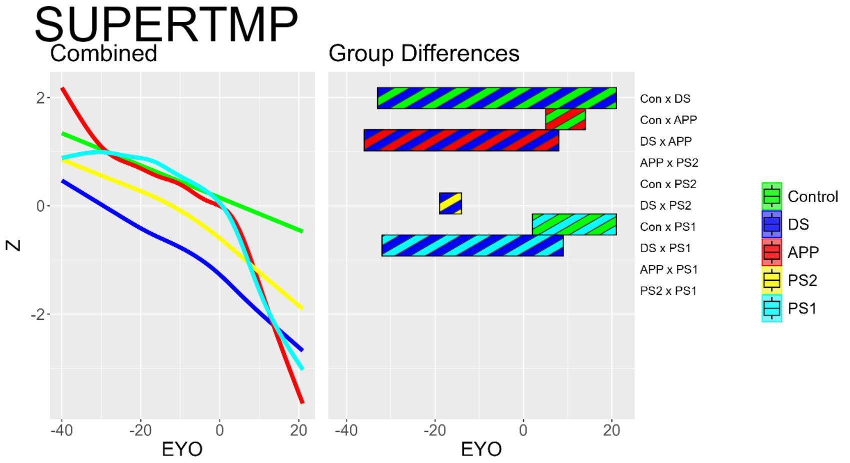
SUPERTMP
Combined
Group Differences
2
0
-2
Z
-40
-20
0
20
EYO
-40
-20
0
20
EYO
Con x DS
Con x APP
DS x APP
APP x PS2
Con x PS2
DS x PS2
Con x PS1
DS x PS1
APP x PS1
PS2 x PS1
Control
DS
APP
PS2
PS1

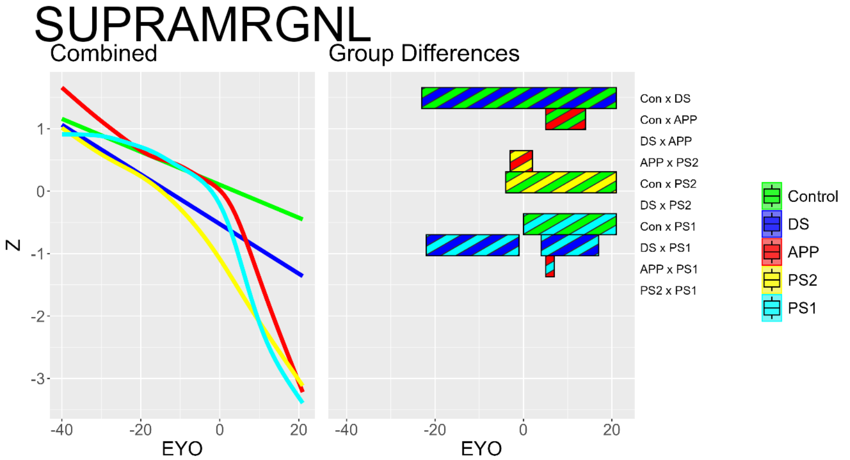

SUPRAMRGNL
Combined
Group Differences
Z
1
0
-1
-2
-3
-40
-20
0
20
EYO
-40
-20
0
20
EYO
Con x DS
Con x APP
DS x APP
APP x PS2
Con x PS2
DS x PS2
Con x PS1
DS x PS1
APP x PS1
PS2 x PS1
Control
DS
APP
PS2
PS1

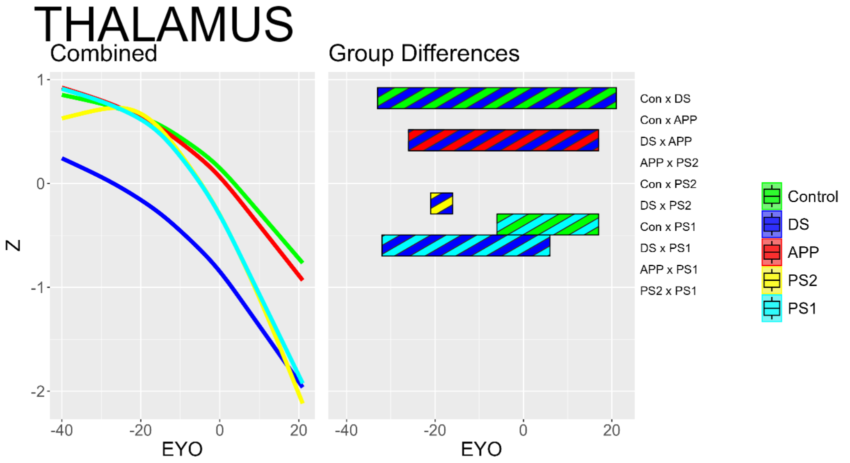
THALAMUS
Combined
Group Differences
1
0
-1
-2
Z
-40
-20
0
20
EYO
Con x DS
Con x APP
DS x APP
APP x PS2
Con x PS2
DS x PS2
Con x PS1
DS x PS1
APP x PS1
PS2 x PS1
Control
DS
APP
PS2
PS1

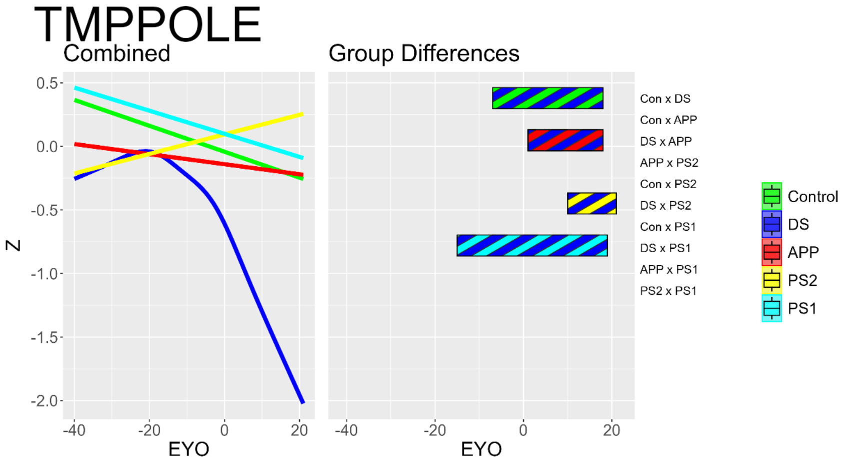

TMPPOLE
Combined
Group Differences
0.5
0.0
-0.5
-1.0
-1.5
-2.0
Z
-40
-20
0
20
EYO
-40
-20
0
20
EYO
Con x DS
Con x APP
DS x APP
APP x PS2
Con x PS2
DS x PS2
Con x PS1
DS x PS1
APP x PS1
PS2 x PS1
Control
DS
APP
PS2
PS1

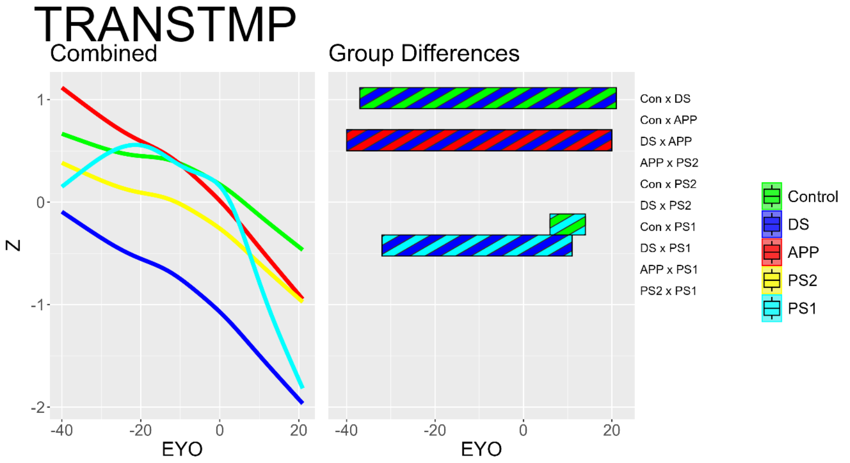

TRANSTMP
Combined
Group Differences
1
0
-1
-2
Z
-40
-20
0
20
EYO
Con x DS
Con x APP
DS x APP
APP x PS2
Con x PS2
DS x PS2
Con x PS1
DS x PS1
APP x PS1
PS2 x PS1
Control
DS
APP
PS2
PS1

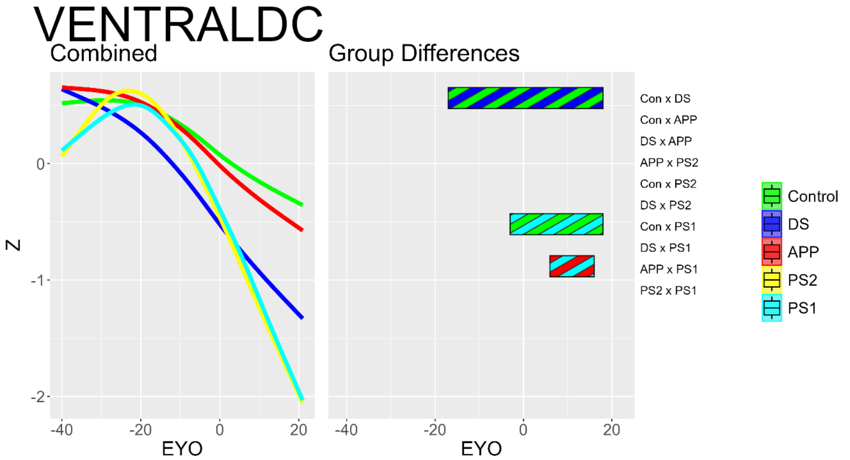

VENTRALDC
Combined
Group Differences
0
-1
-2
Z
-40
-20
0
20
EYO
-40
-20
0
20
EYO
Con x DS
Con x APP
DS x APP
APP x PS2
Con x PS2
DS x PS2
Con x PS1
DS x PS1
APP x PS1
PS2 x PS1
Control
DS
APP
PS2
PS1
