# Supplementary material for: Brain volume trajectories in Down syndrome and autosomal dominant Alzheimer's disease
Source: Alzheimers Dement. 2026 Jan 18;22(1):e71103. doi: 10.1002/alz.71103 (PMC12812856; doi:10.1002/alz.71103)
Supplement: Supplementary file 7 — Supporting information [file ALZ-22-e71103-s007.pdf]

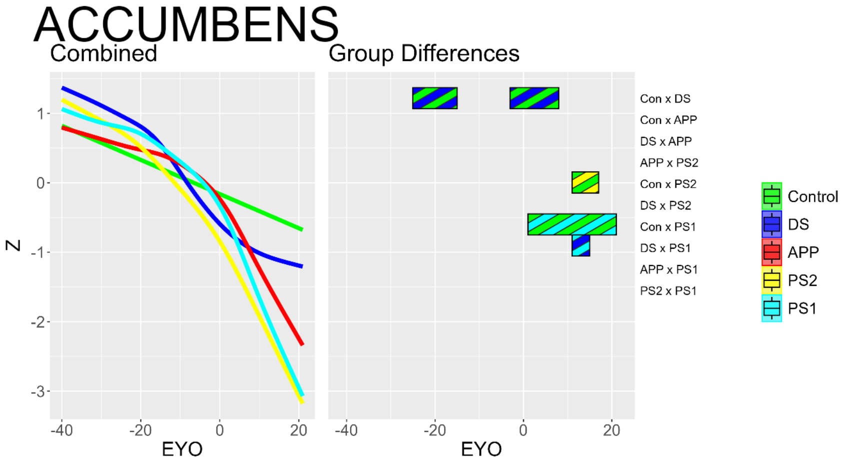

ACCUMBENS
Combined
Group Differences
Z
EYO
EYO
1
0
-1
-2
-3
-40
-20
0
20
-40
-20
0
20
Con x DS
Con x APP
DS x APP
APP x PS2
Con x PS2
DS x PS2
Con x PS1
DS x PS1
APP x PS1
PS2 x PS1
Control
DS
APP
PS2
PS1

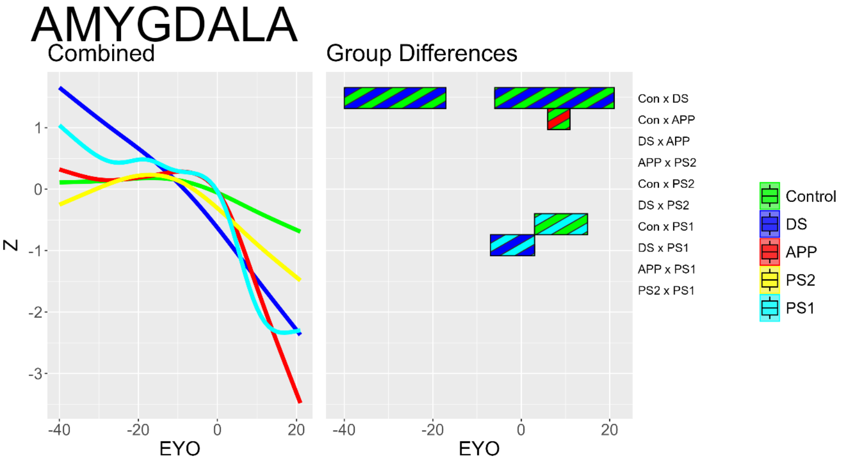
AMYGDALA
Combined
Group Differences
Z
EYO
-40
-20
0
20
1
0
-1
-2
-3
Con x DS
Con x APP
DS x APP
APP x PS2
Con x PS2
DS x PS2
Con x PS1
DS x PS1
APP x PS1
PS2 x PS1
Control
DS
APP
PS2
PS1

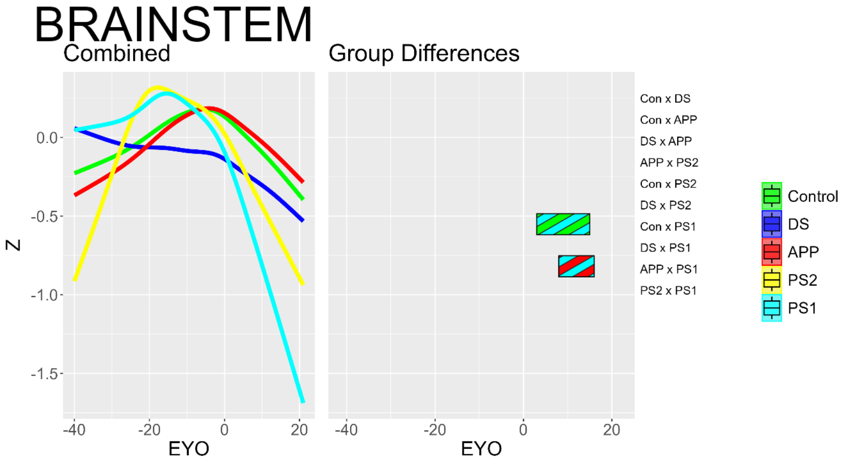

BRAINSTEM
Combined
Group Differences
0.0
-0.5
-1.0
-1.5
Z
-40
-20
0
20
EYO
Con x DS
Con x APP
DS x APP
APP x PS2
Con x PS2
DS x PS2
Con x PS1
DS x PS1
APP x PS1
PS2 x PS1
Control
DS
APP
PS2
PS1

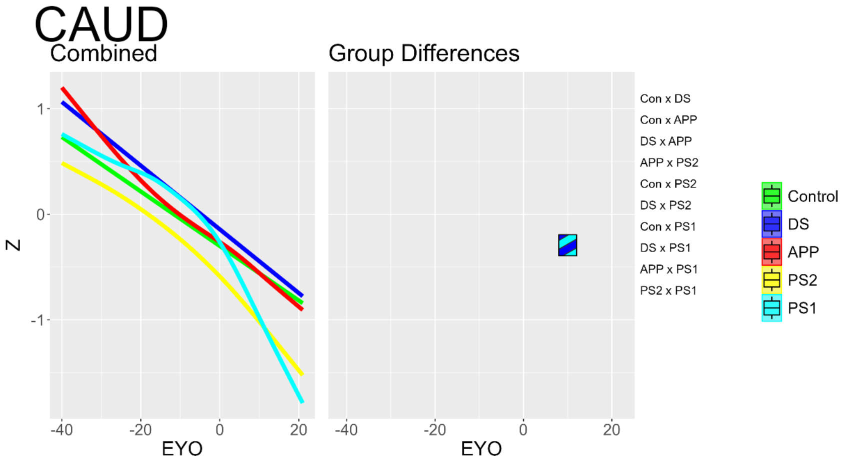
CAUD
Combined
Group Differences
1
0
-1
Z
-40
-20
0
20
EYO
Con x DS
Con x APP
DS x APP
APP x PS2
Con x PS2
DS x PS2
Con x PS1
DS x PS1
APP x PS1
PS2 x PS1
Control
DS
APP
PS2
PS1

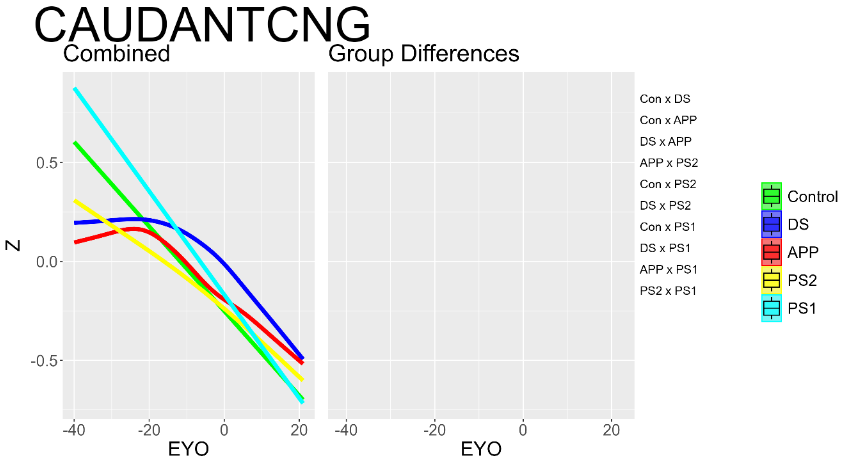
CAUDANTCNG
Combined
Group Differences
Z
0.5
0.0
-0.5
-40
-20
0
20
EYO
-40
-20
0
20
EYO
Con x DS
Con x APP
DS x APP
APP x PS2
Con x PS2
DS x PS2
Con x PS1
DS x PS1
APP x PS1
PS2 x PS1
Control
DS
APP
PS2
PS1

CAUDMIDFRN

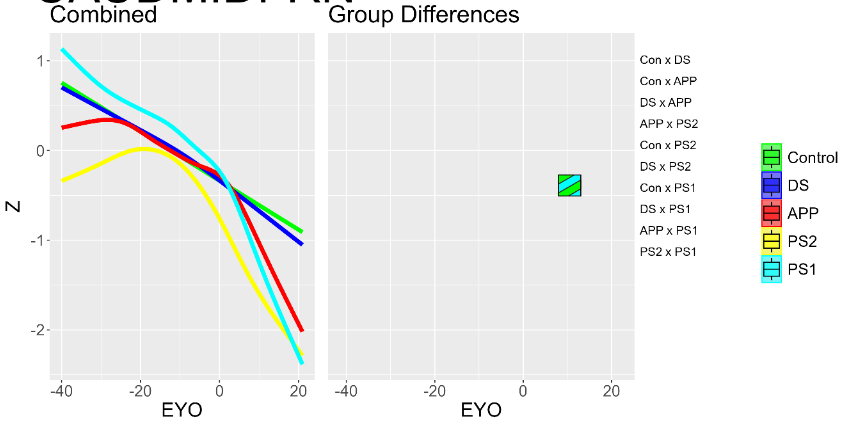

Combined
Group Differences
1
0
-1
-2
Z
-40
-20
0
20
EYO
-40
-20
0
20
EYO
Con x DS
Con x APP
DS x APP
APP x PS2
Con x PS2
DS x PS2
Con x PS1
DS x PS1
APP x PS1
PS2 x PS1
Control
DS
APP
PS2
PS1

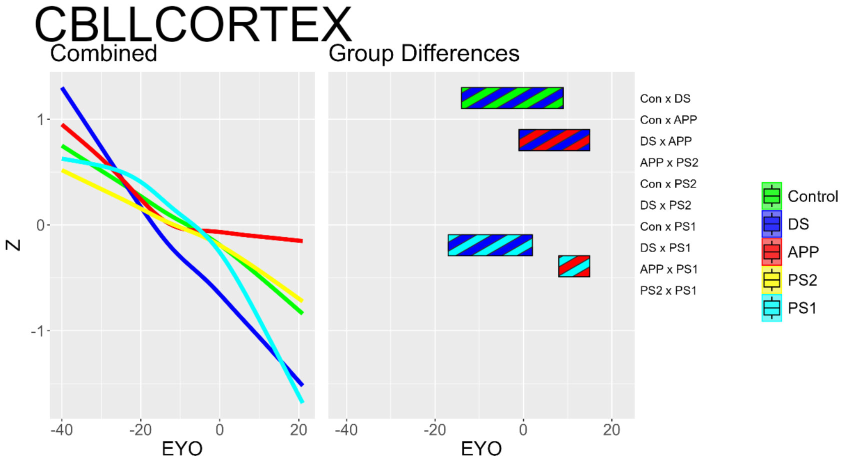
CBLLCORTEX
Combined
Group Differences
Z
1
0
-1
-40
-20
0
20
EYO
-40
-20
0
20
EYO
Con x DS
Con x APP
DS x APP
APP x PS2
Con x PS2
DS x PS2
Con x PS1
DS x PS1
APP x PS1
PS2 x PS1
Control
DS
APP
PS2
PS1

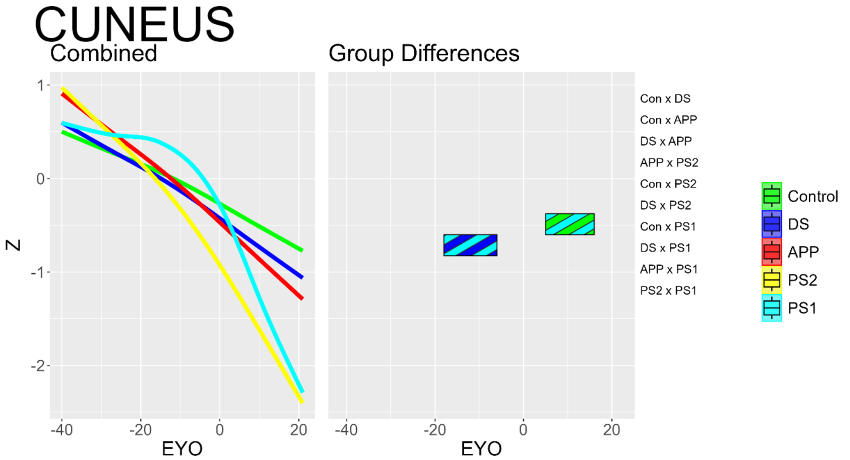
CUNEUS
Combined
Group Differences
1
0
-1
-2
Z
-40
-20
0
20
EYO
-40
-20
0
20
EYO
Con x DS
Con x APP
DS x APP
APP x PS2
Con x PS2
DS x PS2
Con x PS1
DS x PS1
APP x PS1
PS2 x PS1
Control
DS
APP
PS2
PS1

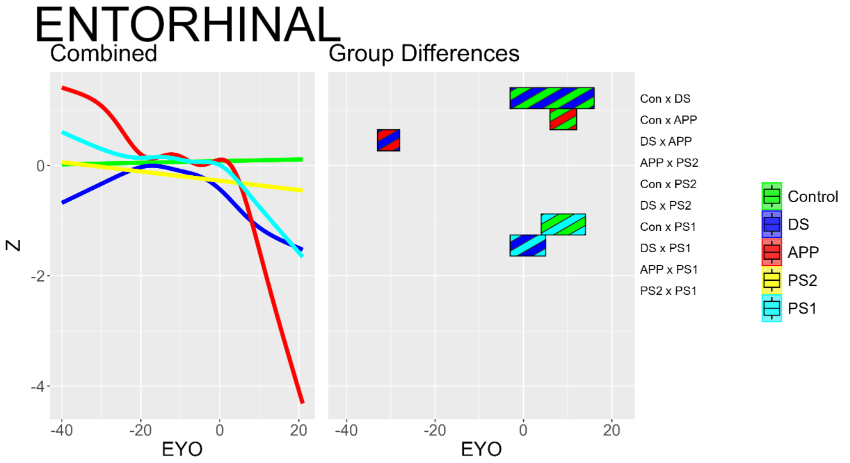
ENTORHINAL
Combined
Group Differences
Z
0
-2
-4
-40
-20
0
20
EYO
-40
-20
0
20
EYO
Con x DS
Con x APP
DS x APP
APP x PS2
Con x PS2
DS x PS2
Con x PS1
DS x PS1
APP x PS1
PS2 x PS1
Control
DS
APP
PS2
PS1

FRNPOLE

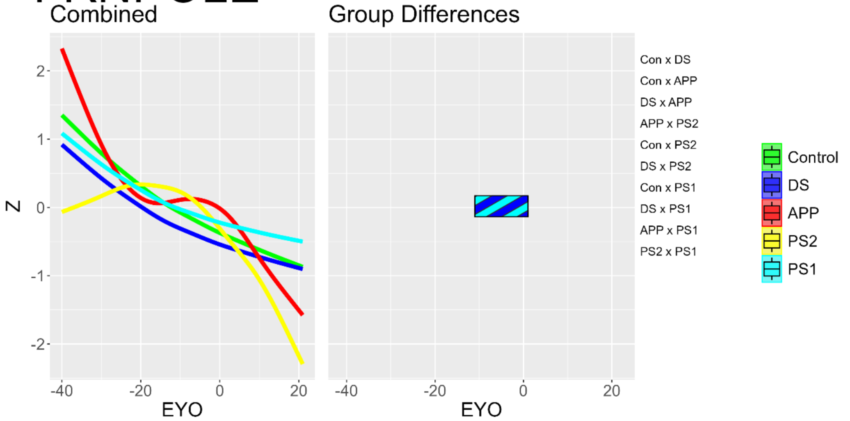

Combined
Group Differences
Z
EYO
EYO
2
1
0
-1
-2
-40
-20
0
20
Con x DS
Con x APP
DS x APP
APP x PS2
Con x PS2
DS x PS2
Con x PS1
DS x PS1
APP x PS1
PS2 x PS1
Control
DS
APP
PS2
PS1

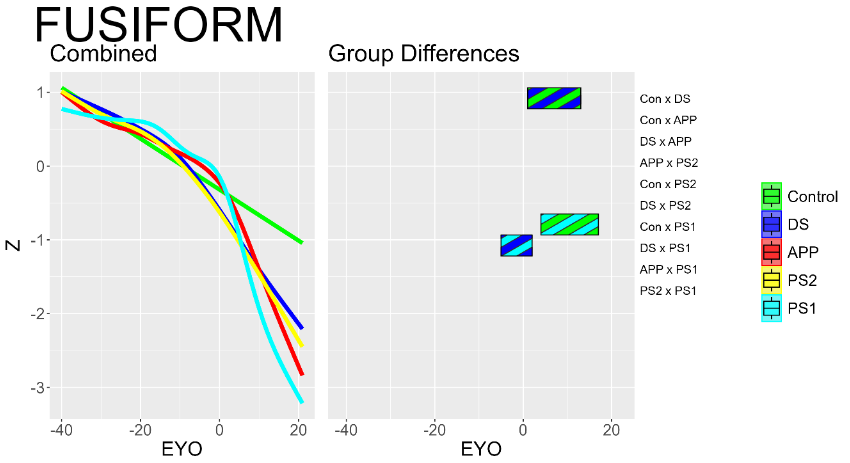
FUSIFORM
Combined
Group Differences
1
0
-1
-2
-3
Z
-40
-20
0
20
EYO
-40
-20
0
20
EYO
Con x DS
Con x APP
DS x APP
APP x PS2
Con x PS2
DS x PS2
Con x PS1
DS x PS1
APP x PS1
PS2 x PS1
Control
DS
APP
PS2
PS1

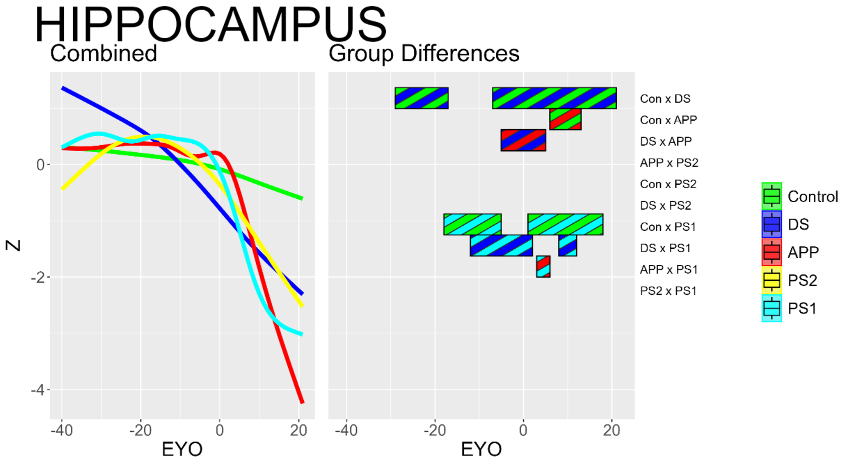
HIPPOCAMPUS
Combined
Group Differences
Z
0
-2
-4
-40
-20
0
20
EYO
Con x DS
Con x APP
DS x APP
APP x PS2
Con x PS2
DS x PS2
Con x PS1
DS x PS1
APP x PS1
PS2 x PS1
Control
DS
APP
PS2
PS1

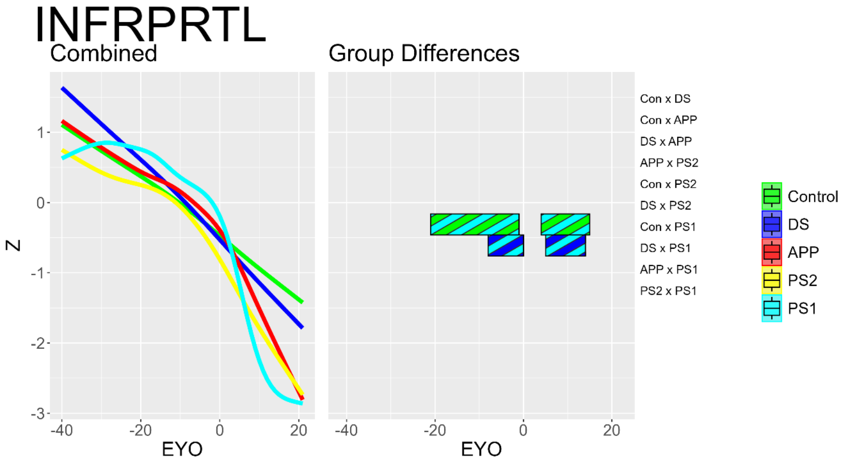

INFRPRTL
Combined
Group Differences
Z
EYO
EYO
1
0
-1
-2
-3
-40
-20
0
20
-40
-20
0
20
Con x DS
Con x APP
DS x APP
APP x PS2
Con x PS2
DS x PS2
Con x PS1
DS x PS1
APP x PS1
PS2 x PS1
Control
DS
APP
PS2
PS1

INFRTMP

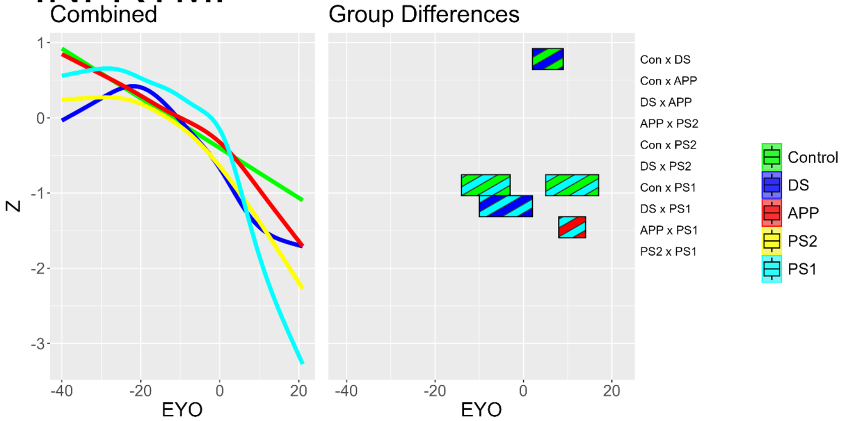

Combined
Group Differences
1
0
-1
-2
-3
Z
-40
-20
0
20
EYO
-40
-20
0
20
EYO
Con x DS
Con x APP
DS x APP
APP x PS2
Con x PS2
DS x PS2
Con x PS1
DS x PS1
APP x PS1
PS2 x PS1
Control
DS
APP
PS2
PS1

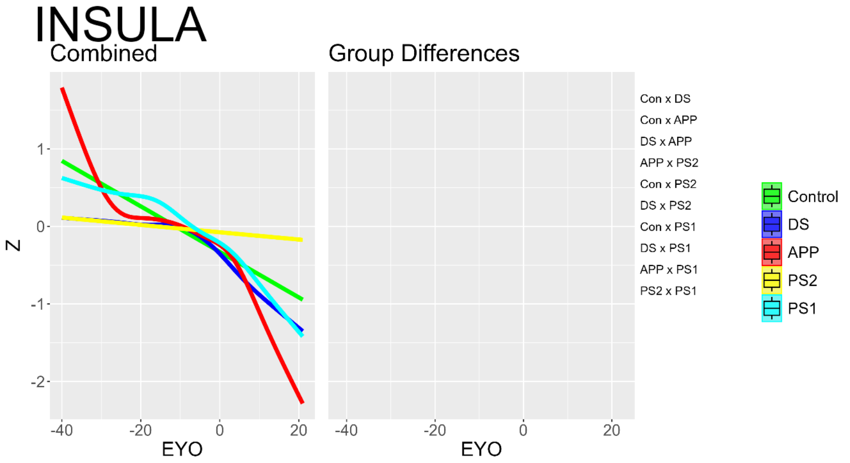

INSULA
Combined
Group Differences
1
0
-1
-2
Z
-40
-20
0
20
EYO
-40
-20
0
20
EYO
Con x DS
Con x APP
DS x APP
APP x PS2
Con x PS2
DS x PS2
Con x PS1
DS x PS1
APP x PS1
PS2 x PS1
Control
DS
APP
PS2
PS1

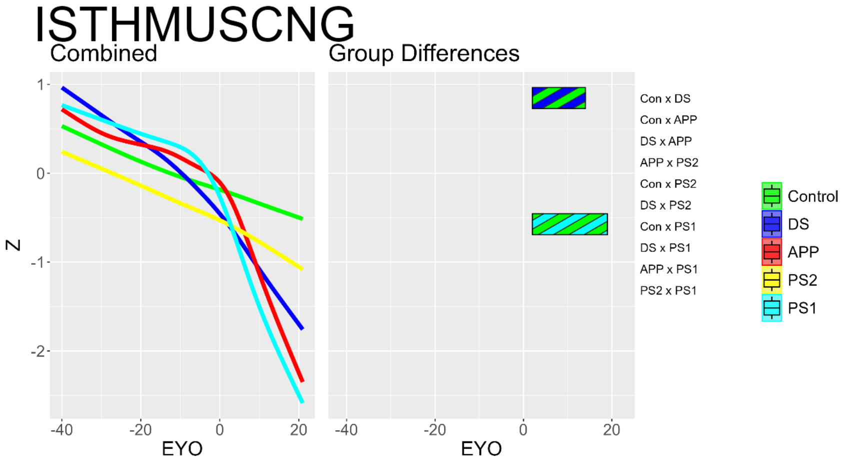
ISTHMUSCNG
Combined
Group Differences
1
0
-1
-2
Z
-40
-20
0
20
EYO
-40
-20
0
20
EYO
Con x DS
Con x APP
DS x APP
APP x PS2
Con x PS2
DS x PS2
Con x PS1
DS x PS1
APP x PS1
PS2 x PS1
Control
DS
APP
PS2
PS1

LATOCC

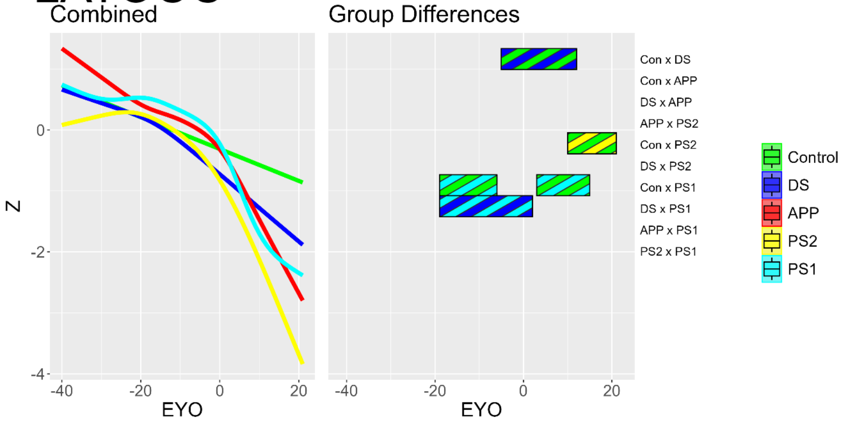

Combined
Group Differences
0
-2
-4
Z
-40
-20
0
20
EYO
-40
-20
0
20
EYO
Con x DS
Con x APP
DS x APP
APP x PS2
Con x PS2
DS x PS2
Con x PS1
DS x PS1
APP x PS1
PS2 x PS1
Control
DS
APP
PS2
PS1

LATORBFRN

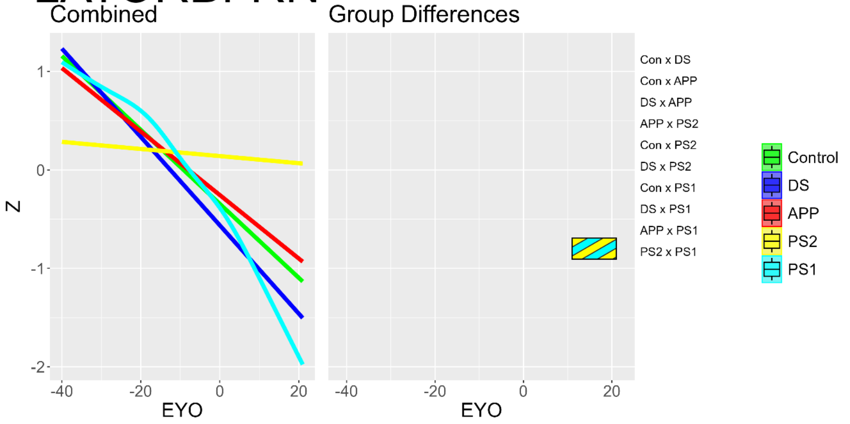
Combined
Group Differences
Z
EYO
EYO
1
0
-1
-2
-40
-20
0
20
-40
-20
0
20
Con x DS
Con x APP
DS x APP
APP x PS2
Con x PS2
DS x PS2
Con x PS1
DS x PS1
APP x PS1
PS2 x PS1
Control
DS
APP
PS2
PS1

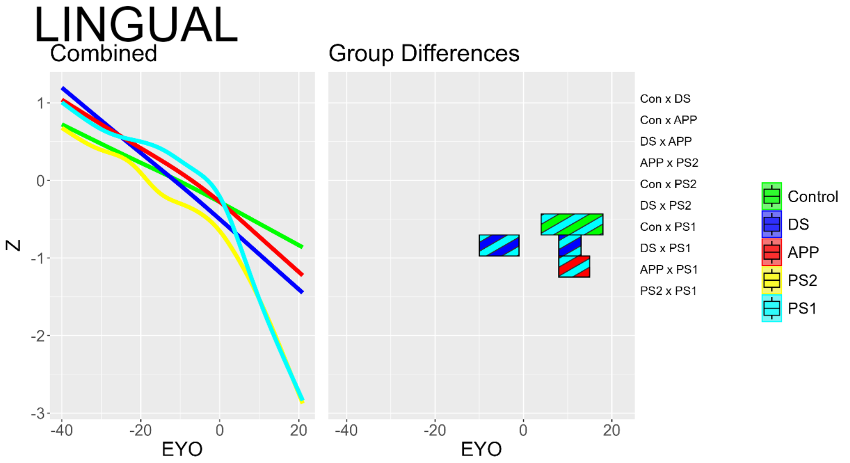
LINGUAL
Combined
Group Differences
1
0
-1
-2
-3
Z
-40
-20
0
20
EYO
-40
-20
0
20
EYO
Con x DS
Con x APP
DS x APP
APP x PS2
Con x PS2
DS x PS2
Con x PS1
DS x PS1
APP x PS1
PS2 x PS1
Control
DS
APP
PS2
PS1

MEDORBFRN

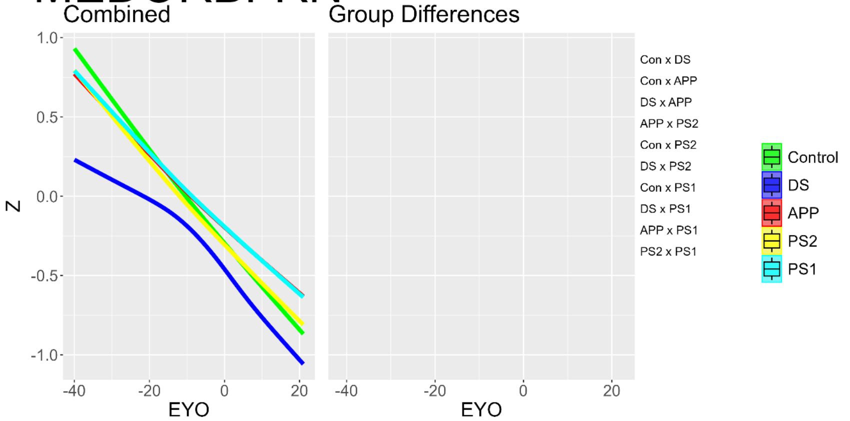

Combined
Group Differences
1.0
0.5
0.0
-0.5
-1.0
Z
-40
-20
0
20
EYO
-40
-20
0
20
EYO
Con x DS
Con x APP
DS x APP
APP x PS2
Con x PS2
DS x PS2
Con x PS1
DS x PS1
APP x PS1
PS2 x PS1
Control
DS
APP
PS2
PS1

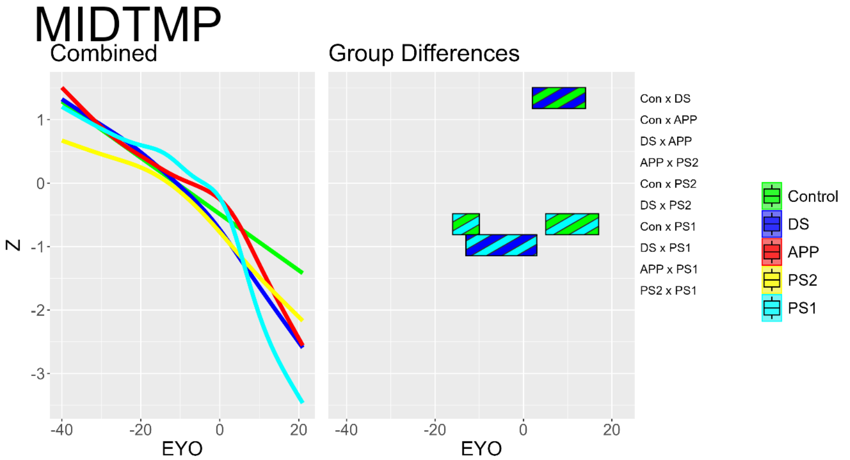
MIDTMP
Combined
Group Differences
Z
EYO
EYO
1
0
-1
-2
-3
-40
-20
0
20
-40
-20
0
20
Con x DS
Con x APP
DS x APP
APP x PS2
Con x PS2
DS x PS2
Con x PS1
DS x PS1
APP x PS1
PS2 x PS1
Control
DS
APP
PS2
PS1

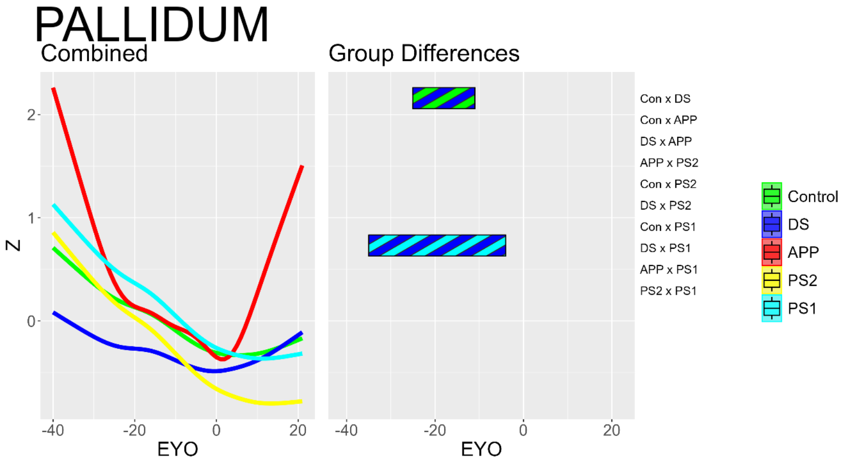

PALLIDUM
Combined
Group Differences
Z
EYO
EYO
-40
-20
0
20
-40
-20
0
20
0
1
2
Con x DS
Con x APP
DS x APP
APP x PS2
Con x PS2
DS x PS2
Con x PS1
DS x PS1
APP x PS1
PS2 x PS1
Control
DS
APP
PS2
PS1

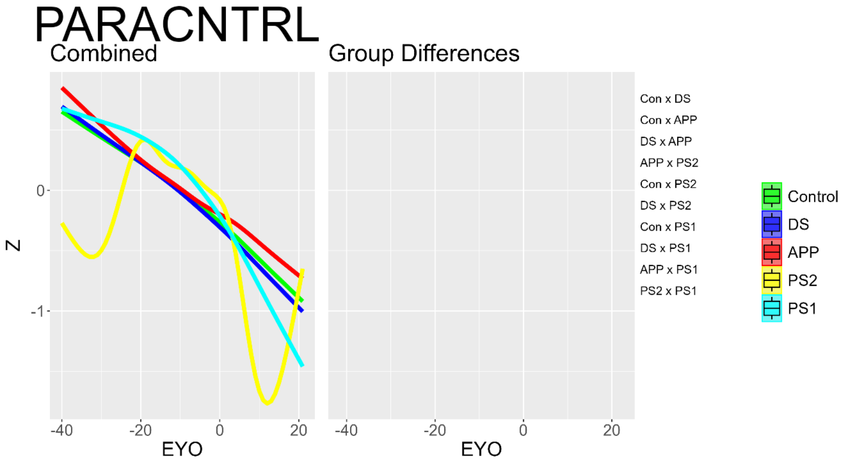
PARACNTRL
Combined
Group Differences
Z
0
-1
-40
-20
0
20
EYO
-40
-20
0
20
EYO
Con x DS
Con x APP
DS x APP
APP x PS2
Con x PS2
DS x PS2
Con x PS1
DS x PS1
APP x PS1
PS2 x PS1
Control
DS
APP
PS2
PS1

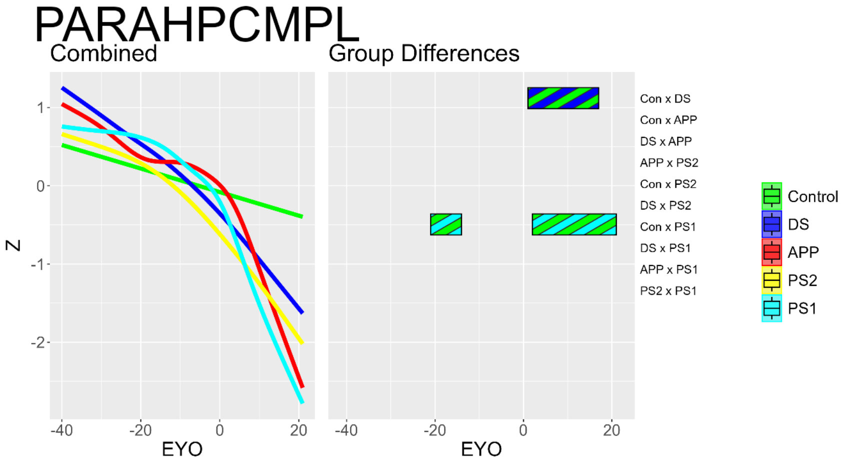

PARAHPCMPL
Combined
Group Differences
Z
1
0
-1
-2
-40
-20
0
20
EYO
-40
-20
0
20
EYO
Con x DS
Con x APP
DS x APP
APP x PS2
Con x PS2
DS x PS2
Con x PS1
DS x PS1
APP x PS1
PS2 x PS1
Control
DS
APP
PS2
PS1

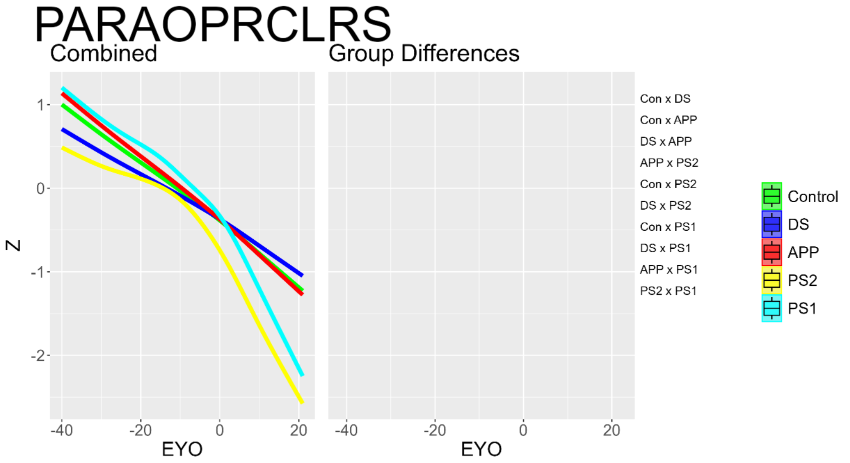
PARAOPRCLRS
Combined
Group Differences
Z
1
0
-1
-2
-40
-20
0
20
EYO
-40
-20
0
20
EYO
Con x DS
Con x APP
DS x APP
APP x PS2
Con x PS2
DS x PS2
Con x PS1
DS x PS1
APP x PS1
PS2 x PS1
Control
DS
APP
PS2
PS1

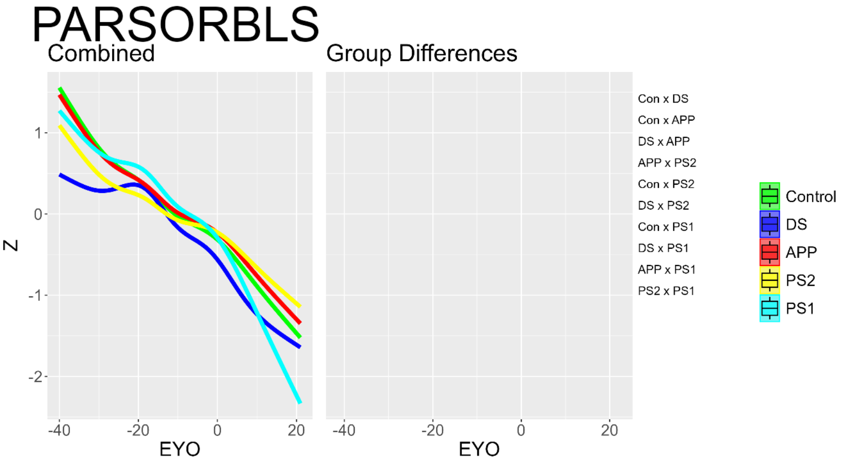
PARSORBLS
Combined
Group Differences
Z
1
0
-1
-2
-40
-20
0
20
EYO
-40
-20
0
20
EYO
Con x DS
Con x APP
DS x APP
APP x PS2
Con x PS2
DS x PS2
Con x PS1
DS x PS1
APP x PS1
PS2 x PS1
Control
DS
APP
PS2
PS1

PARSTRNGLRS

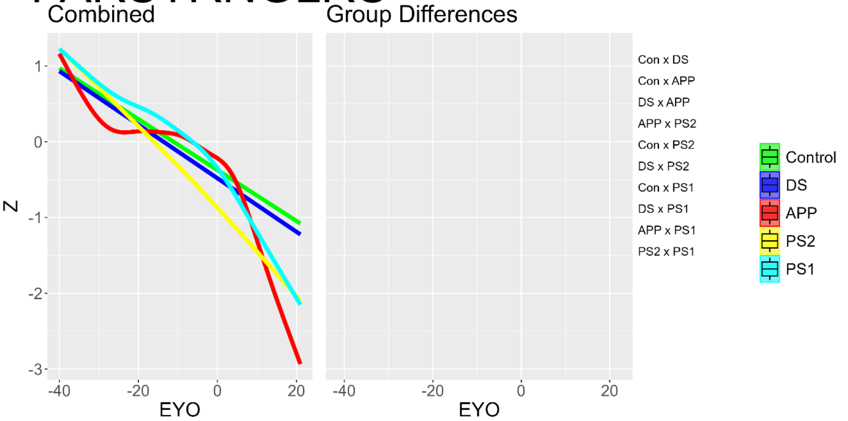

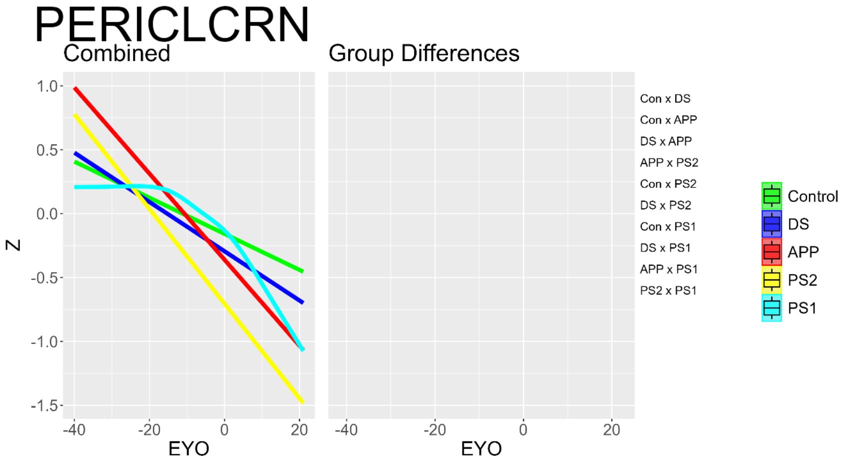
PERICLCRN
Combined
Group Differences
1.0
0.5
0.0
-0.5
-1.0
-1.5
Z
-40
-20
0
20
EYO
-40
-20
0
20
EYO
Con x DS
Con x APP
DS x APP
APP x PS2
Con x PS2
DS x PS2
Con x PS1
DS x PS1
APP x PS1
PS2 x PS1
Control
DS
APP
PS2
PS1

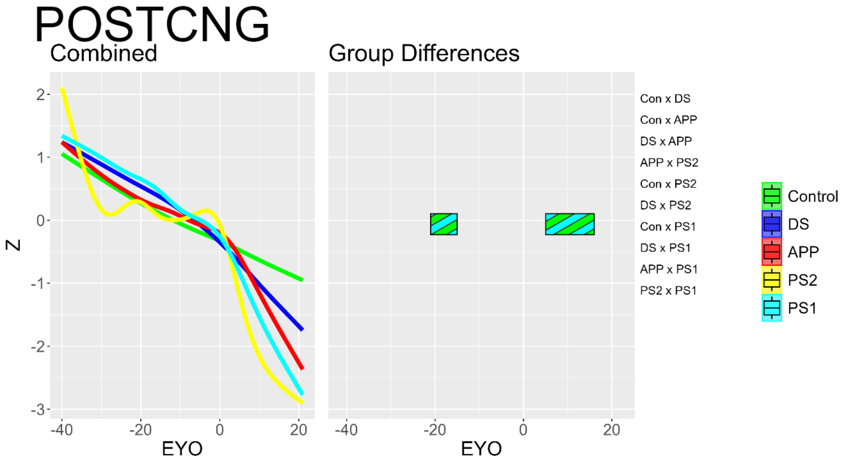
POSTCNG
Combined
Group Differences
Z
EYO
EYO
2
1
0
-1
-2
-3
-40
-20
0
20
-40
-20
0
20
Con x DS
Con x APP
DS x APP
APP x PS2
Con x PS2
DS x PS2
Con x PS1
DS x PS1
APP x PS1
PS2 x PS1
Control
DS
APP
PS2
PS1

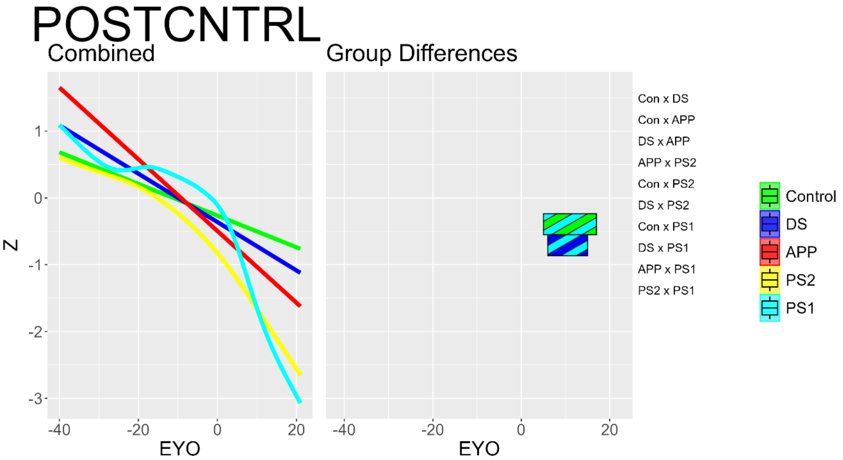
POSTCNTRL
Combined
Group Differences
1
0
-1
-2
-3
Z
-40
-20
0
20
EYO
-40
-20
0
20
EYO
Con x DS
Con x APP
DS x APP
APP x PS2
Con x PS2
DS x PS2
Con x PS1
DS x PS1
APP x PS1
PS2 x PS1
Control
DS
APP
PS2
PS1

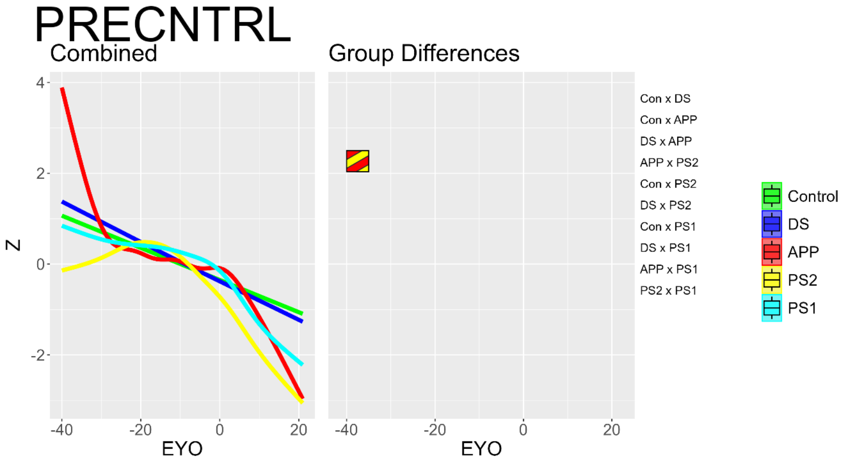
PRECNTRL
Combined
Group Differences
Z
EYO
EYO
4
2
0
-2
-40
-20
0
20
-40
-20
0
20
Con x DS
Con x APP
DS x APP
APP x PS2
Con x PS2
DS x PS2
Con x PS1
DS x PS1
APP x PS1
PS2 x PS1
Control
DS
APP
PS2
PS1

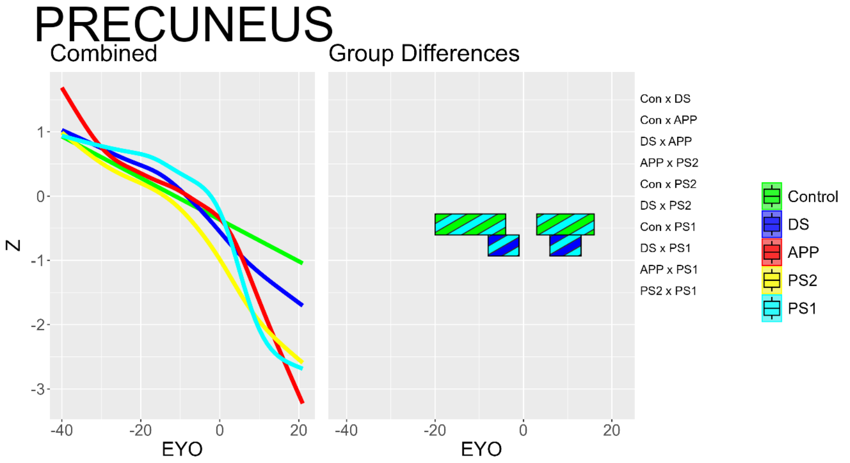
PRECUNEUS
Combined
Group Differences
Z
EYO
EYO
1
0
-1
-2
-3
-40
-20
0
20
-40
-20
0
20
Con x DS
Con x APP
DS x APP
APP x PS2
Con x PS2
DS x PS2
Con x PS1
DS x PS1
APP x PS1
PS2 x PS1
Control
DS
APP
PS2
PS1

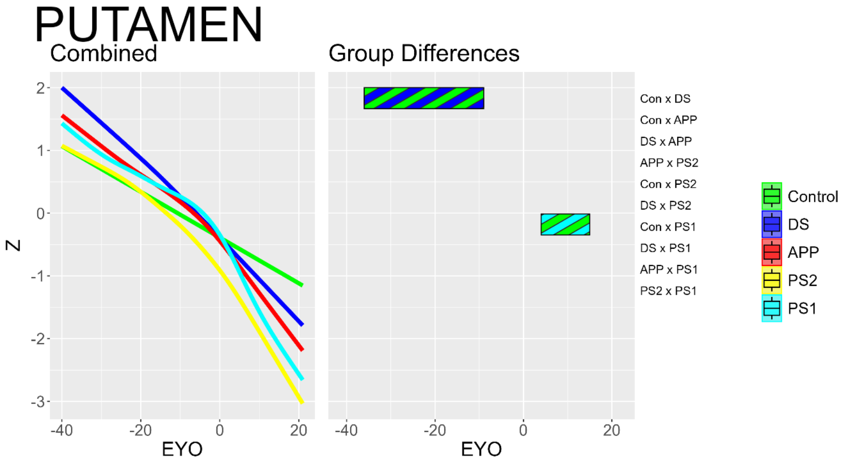

PUTAMEN
Combined
Group Differences
2
1
0
-1
-2
-3
Z
-40
-20
0
20
EYO
Con x DS
Con x APP
DS x APP
APP x PS2
Con x PS2
DS x PS2
Con x PS1
DS x PS1
APP x PS1
PS2 x PS1
Control
DS
APP
PS2
PS1

ROSANTCNG

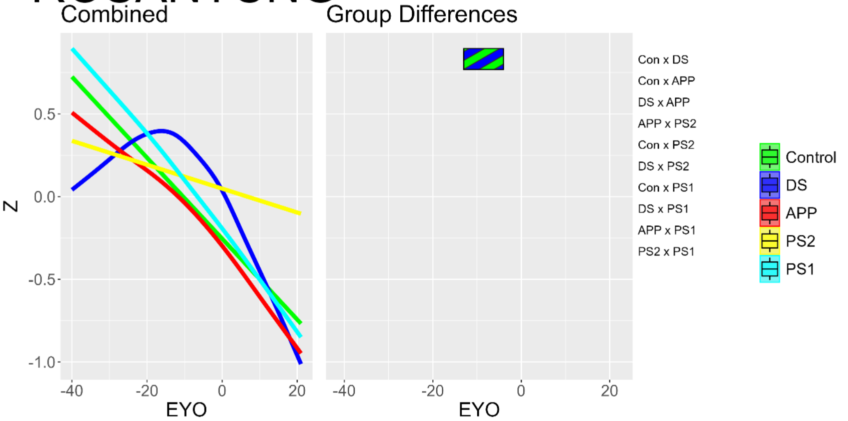

Combined
Group Differences
Z
0.5
0.0
-0.5
-1.0
-40
-20
0
20
EYO
-40
-20
0
20
EYO
Con x DS
Con x APP
DS x APP
APP x PS2
Con x PS2
DS x PS2
Con x PS1
DS x PS1
APP x PS1
PS2 x PS1
Control
DS
APP
PS2
PS1

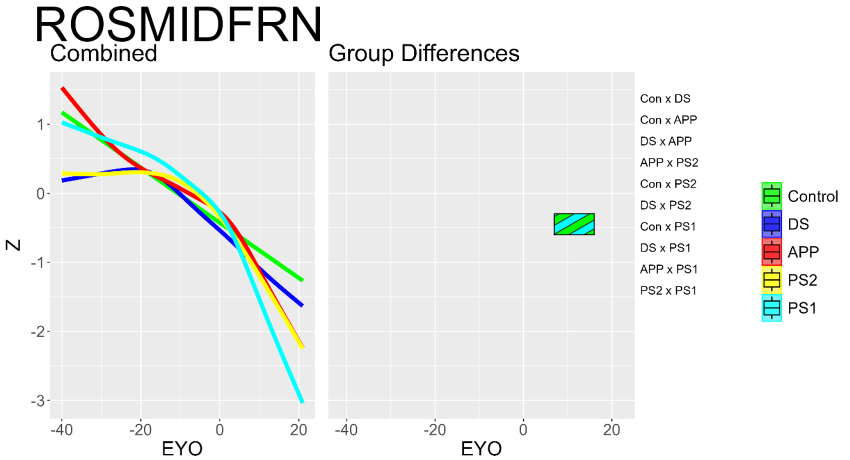
ROSMIDFRN
Combined
Group Differences
Z
EYO
EYO
1
0
-1
-2
-3
-40
-20
0
20
-40
-20
0
20
Con x DS
Con x APP
DS x APP
APP x PS2
Con x PS2
DS x PS2
Con x PS1
DS x PS1
APP x PS1
PS2 x PS1
Control
DS
APP
PS2
PS1

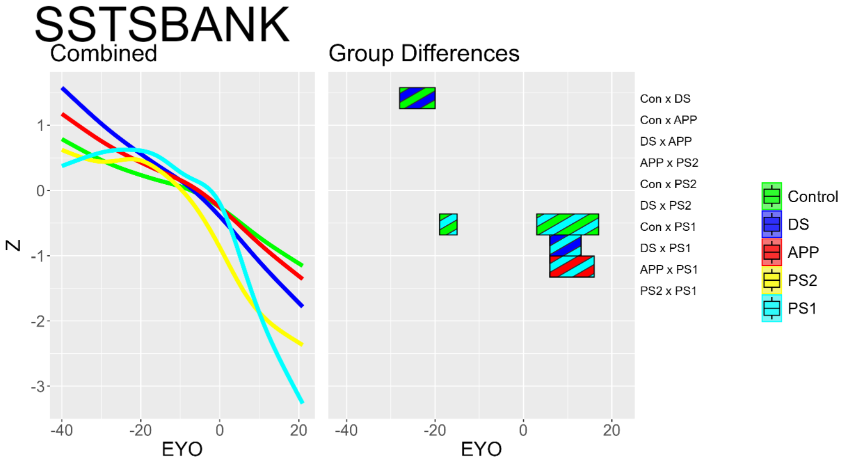
SSTSBANK
Combined
Group Differences
Z
1
0
-1
-2
-3
-40
-20
0
20
EYO
-40
-20
0
20
EYO
Con x DS
Con x APP
DS x APP
APP x PS2
Con x PS2
DS x PS2
Con x PS1
DS x PS1
APP x PS1
PS2 x PS1
Control
DS
APP
PS2
PS1

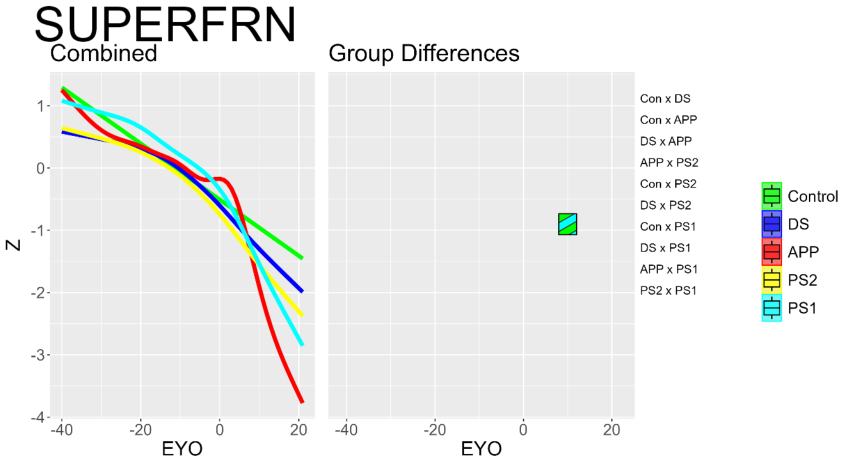
SUPERFRN
Combined
Group Differences
Z
1
0
-1
-2
-3
-4
-40
-20
0
20
EYO
-40
-20
0
20
EYO
Con x DS
Con x APP
DS x APP
APP x PS2
Con x PS2
DS x PS2
Con x PS1
DS x PS1
APP x PS1
PS2 x PS1
Control
DS
APP
PS2
PS1

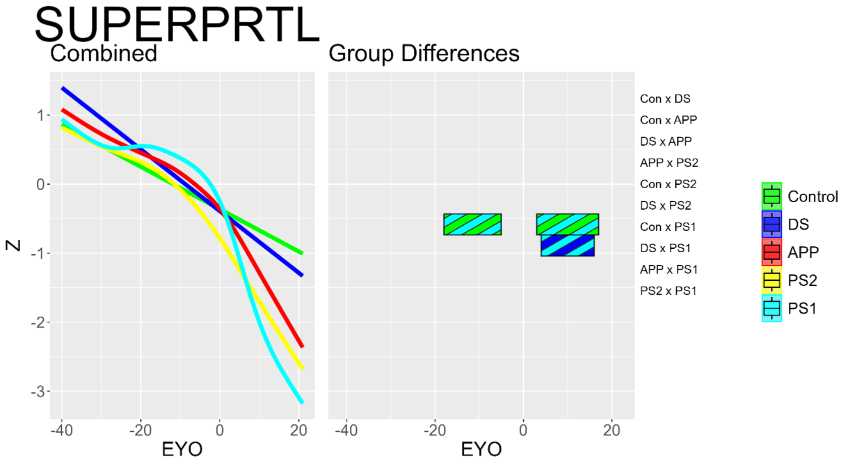
SUPERPRTL
Combined
Group Differences
Z
1
0
-1
-2
-3
-40
-20
0
20
EYO
-40
-20
0
20
EYO
Con x DS
Con x APP
DS x APP
APP x PS2
Con x PS2
DS x PS2
Con x PS1
DS x PS1
APP x PS1
PS2 x PS1
Control
DS
APP
PS2
PS1

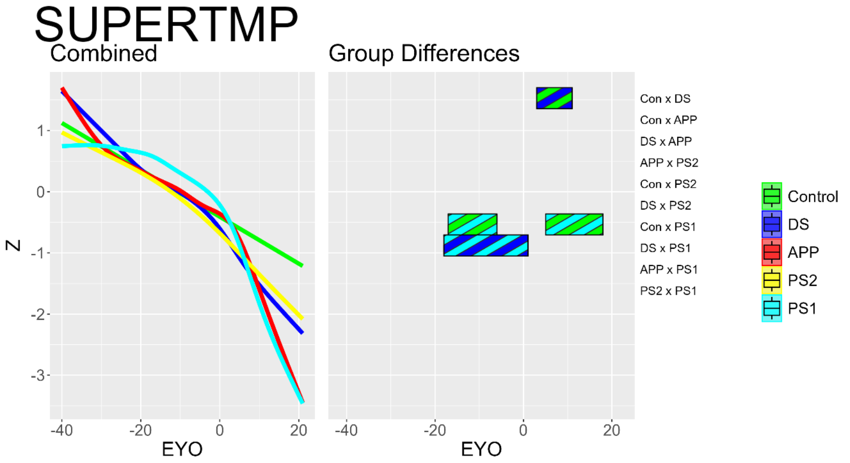
SUPERTMP
Combined
Group Differences
Z
EYO
EYO
1
0
-1
-2
-3
-40
-20
0
20
-40
-20
0
20
Con x DS
Con x APP
DS x APP
APP x PS2
Con x PS2
DS x PS2
Con x PS1
DS x PS1
APP x PS1
PS2 x PS1
Control
DS
APP
PS2
PS1

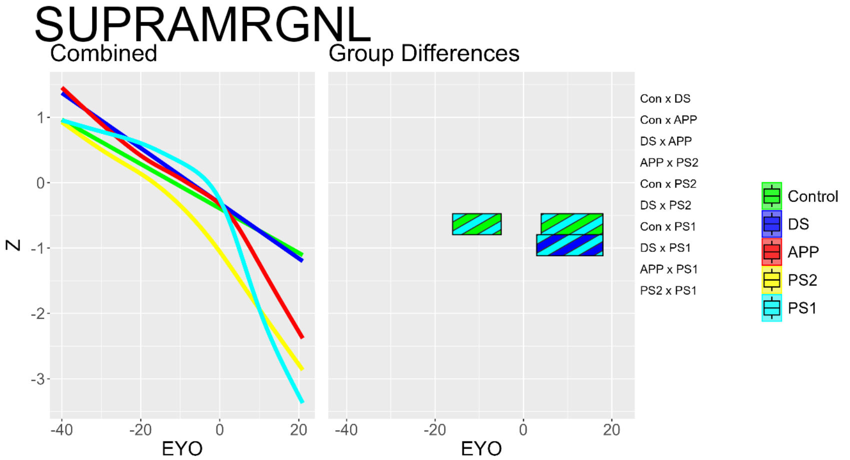
SUPRAMRGNL
Combined
Group Differences
Z
EYO
EYO
1
0
-1
-2
-3
-40
-20
0
20
-40
-20
0
20
Con x DS
Con x APP
DS x APP
APP x PS2
Con x PS2
DS x PS2
Con x PS1
DS x PS1
APP x PS1
PS2 x PS1
Control
DS
APP
PS2
PS1

THALAMUS

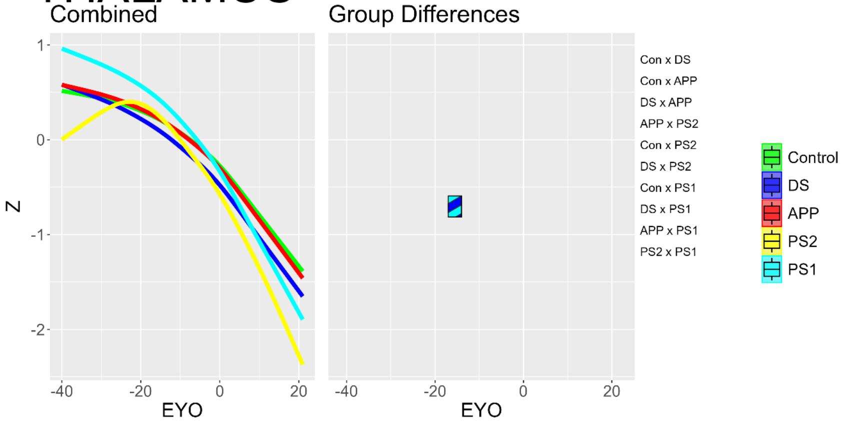

Combined
Group Differences
1
0
-1
-2
Z
-40
-20
0
20
EYO
-40
-20
0
20
EYO
Con x DS
Con x APP
DS x APP
APP x PS2
Con x PS2
DS x PS2
Con x PS1
DS x PS1
APP x PS1
PS2 x PS1
Control
DS
APP
PS2
PS1

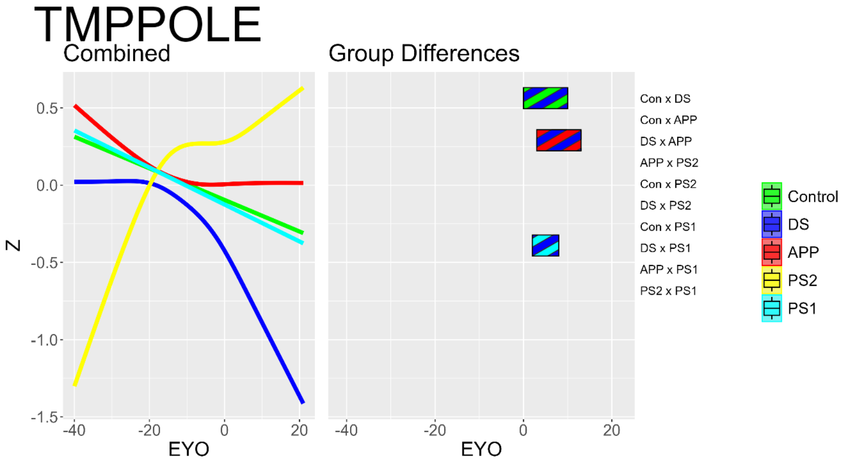
TMPPOLE
Combined
Group Differences
0.5
0.0
-0.5
-1.0
-1.5
Z
-40
-20
0
20
EYO
-40
-20
0
20
EYO
Con x DS
Con x APP
DS x APP
APP x PS2
Con x PS2
DS x PS2
Con x PS1
DS x PS1
APP x PS1
PS2 x PS1
Control
DS
APP
PS2
PS1

TRANSTMP

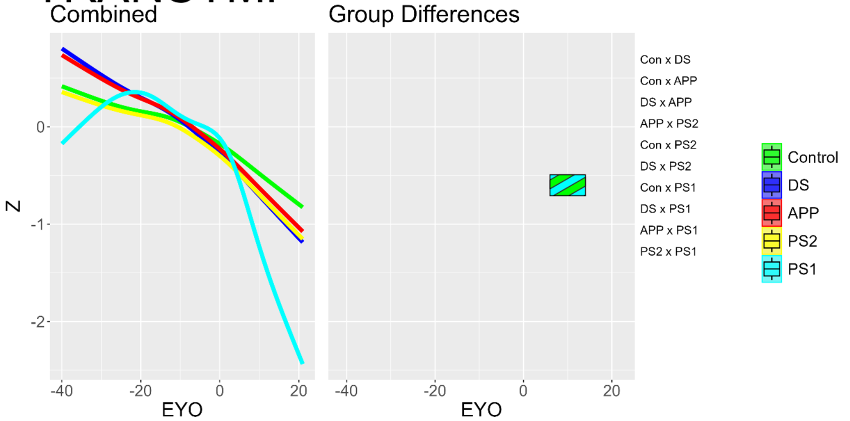

Combined
Group Differences
0
-1
-2
Z
-40
-20
0
20
EYO
Con x DS
Con x APP
DS x APP
APP x PS2
Con x PS2
DS x PS2
Con x PS1
DS x PS1
APP x PS1
PS2 x PS1
Control
DS
APP
PS2
PS1

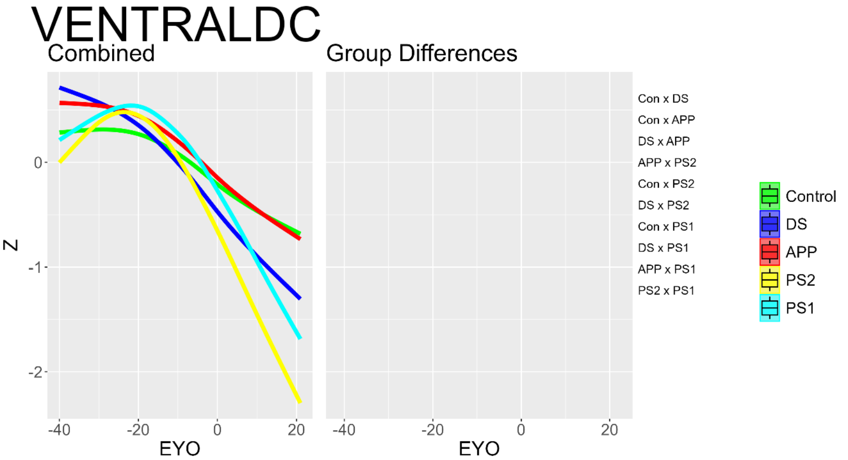

VENTRALDC
Combined
Group Differences
Z
0
-1
-2
-40
-20
0
20
EYO
-40
-20
0
20
EYO
Con x DS
Con x APP
DS x APP
APP x PS2
Con x PS2
DS x PS2
Con x PS1
DS x PS1
APP x PS1
PS2 x PS1
Control
DS
APP
PS2
PS1
