# Supplementary figures and images for: Brain volume trajectories in Down syndrome and autosomal dominant Alzheimer's disease
Source: Alzheimers Dement. 2026 Jan 18;22(1):e71103. doi: 10.1002/alz.71103 (PMC12812856; doi:10.1002/alz.71103)

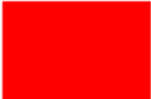 **DS**

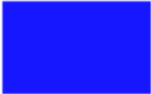 **ADAD**

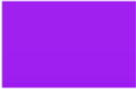 **DS - ADAD**

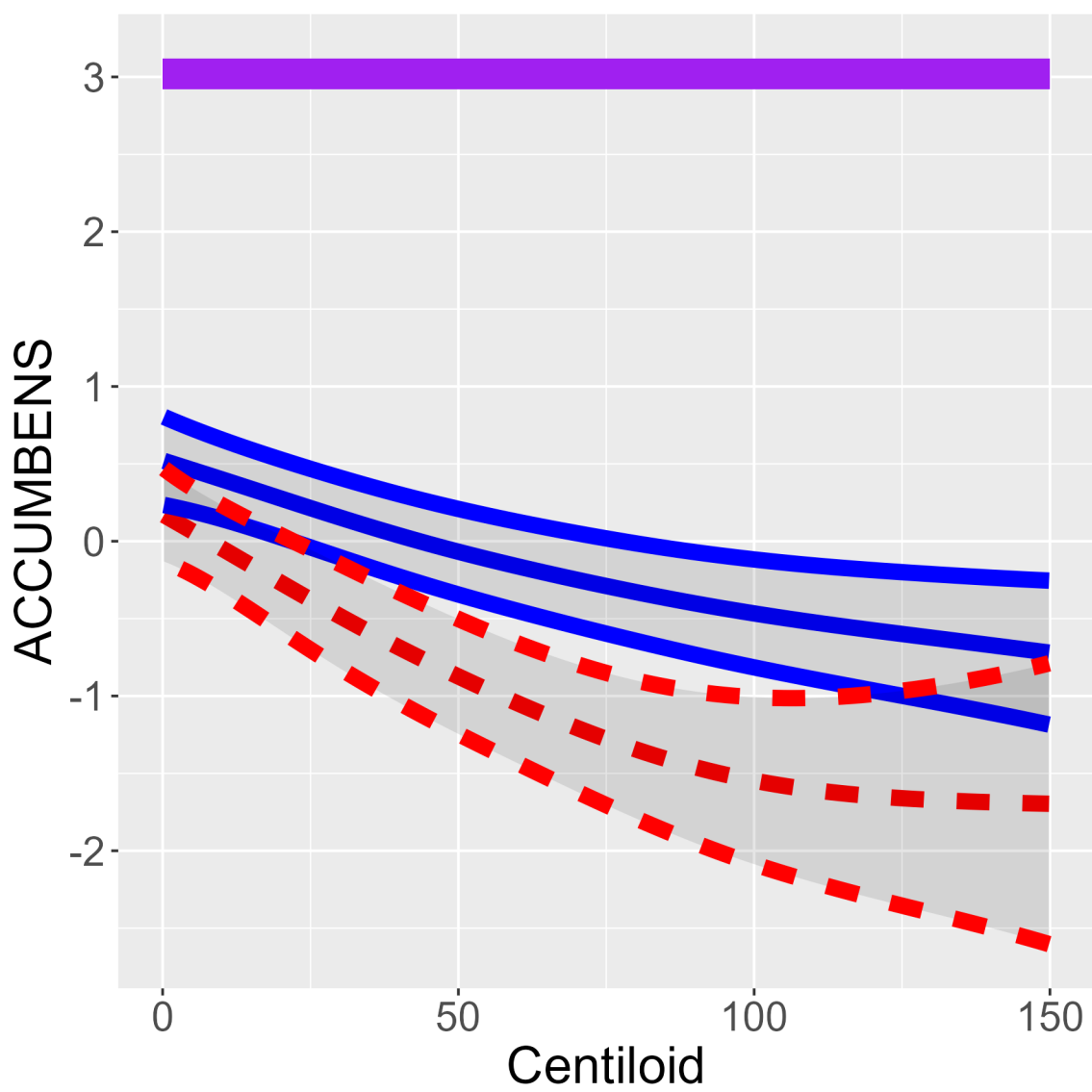

AMYGDALA

2.5

0.0

-2.5

0

50

100

150

Centiloid

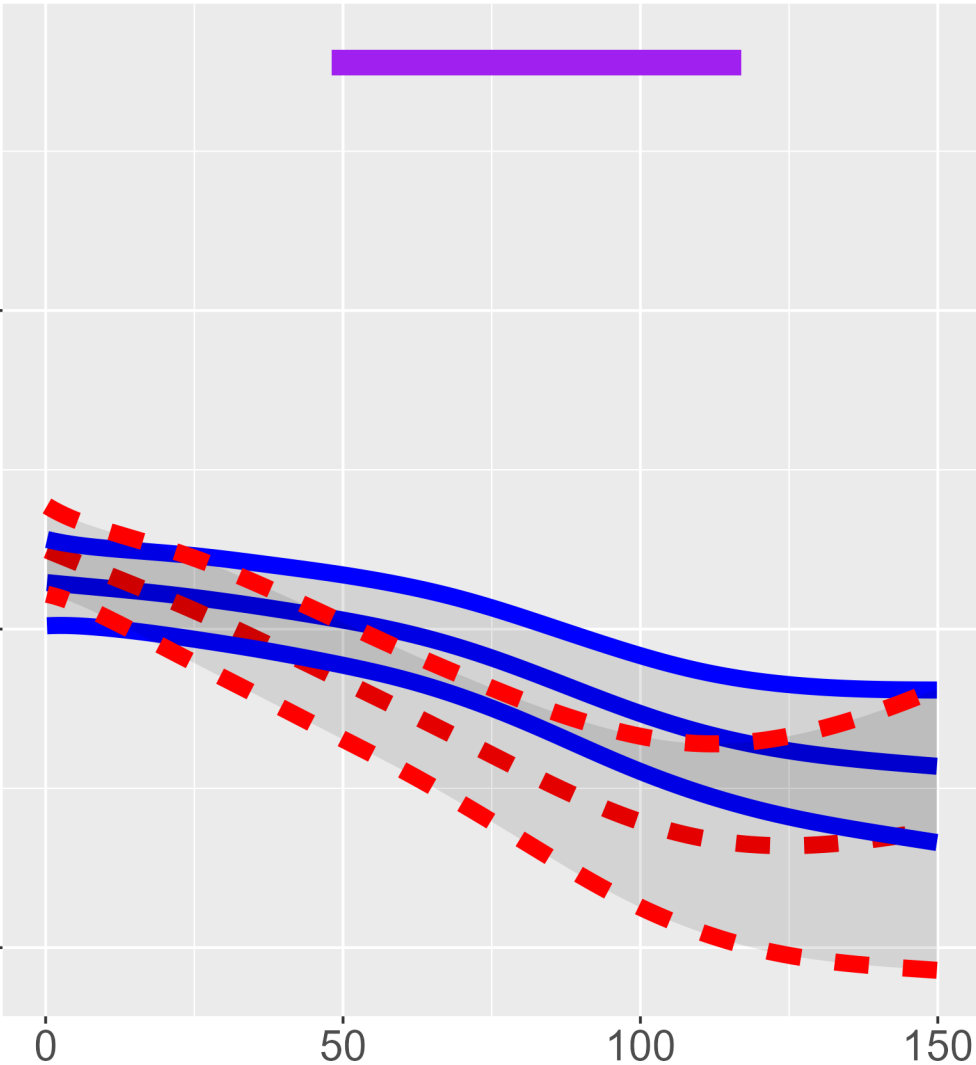

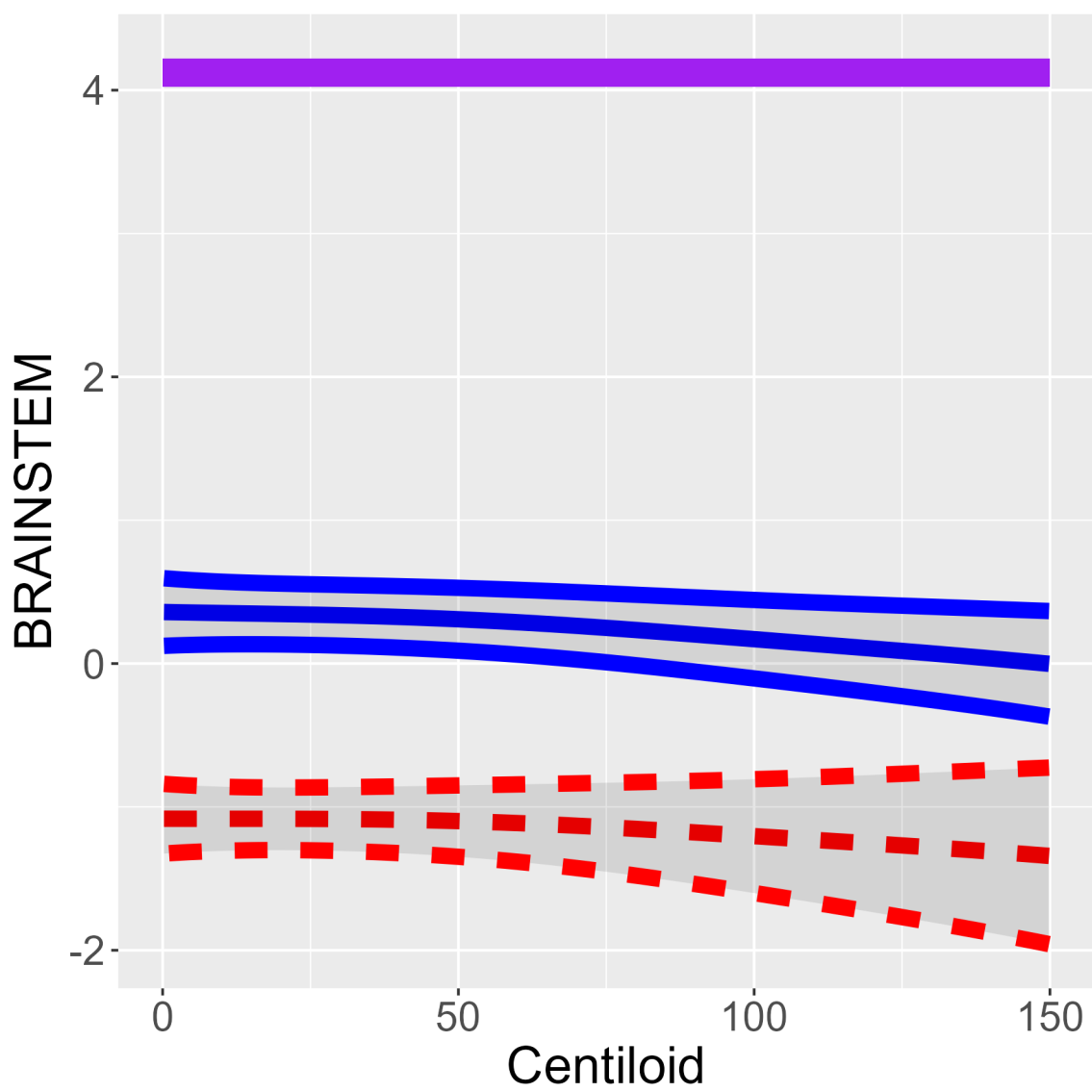

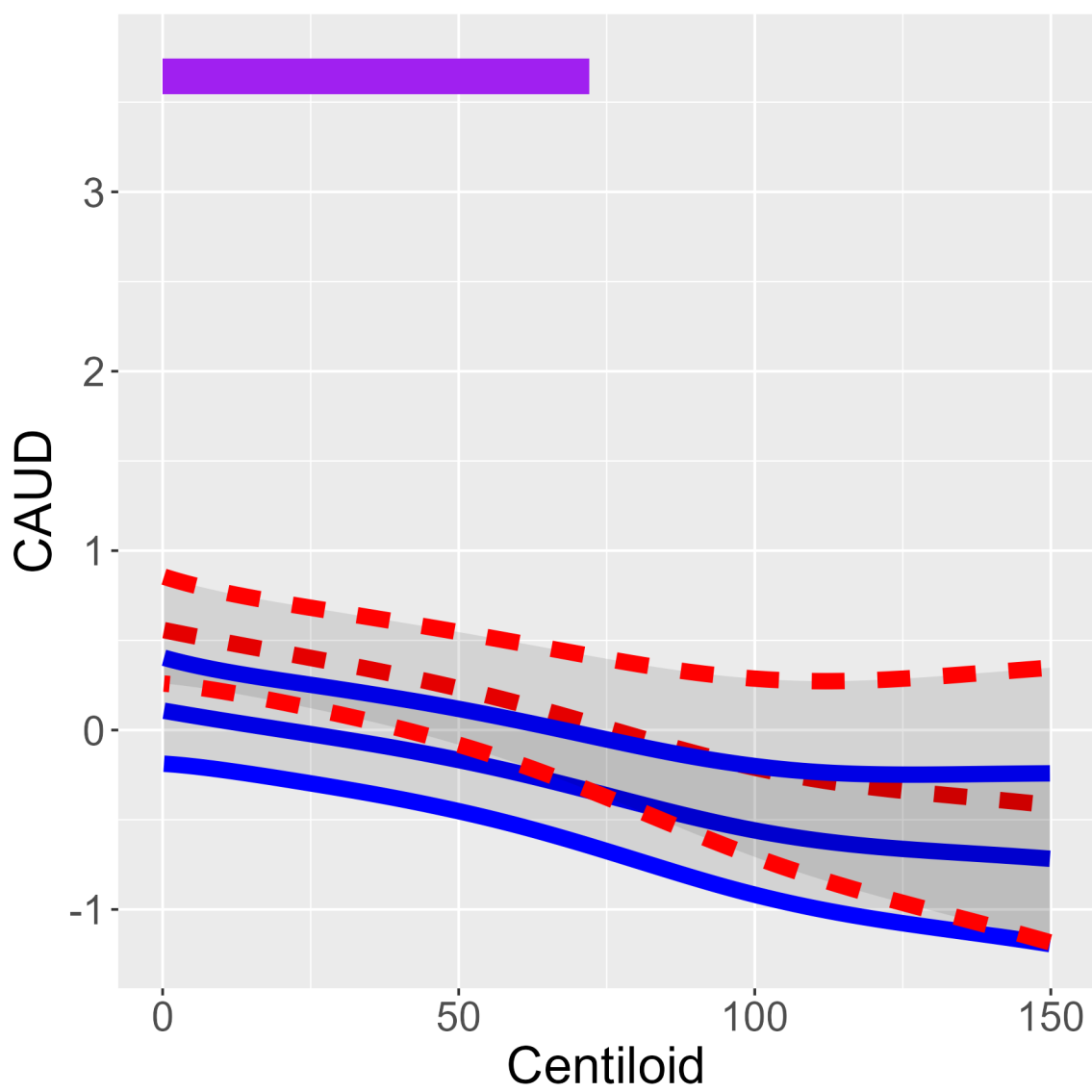

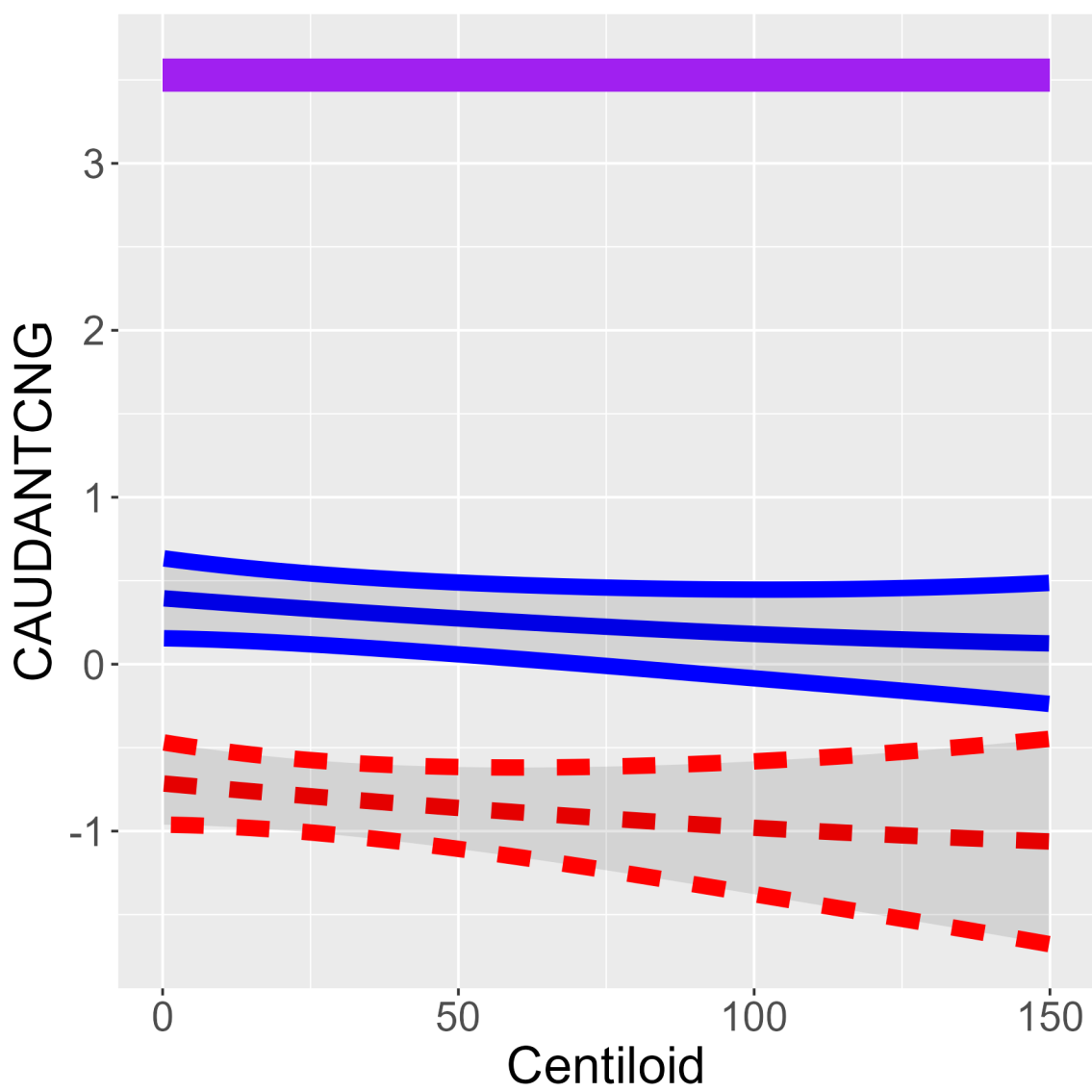

CAUDMIDFNR

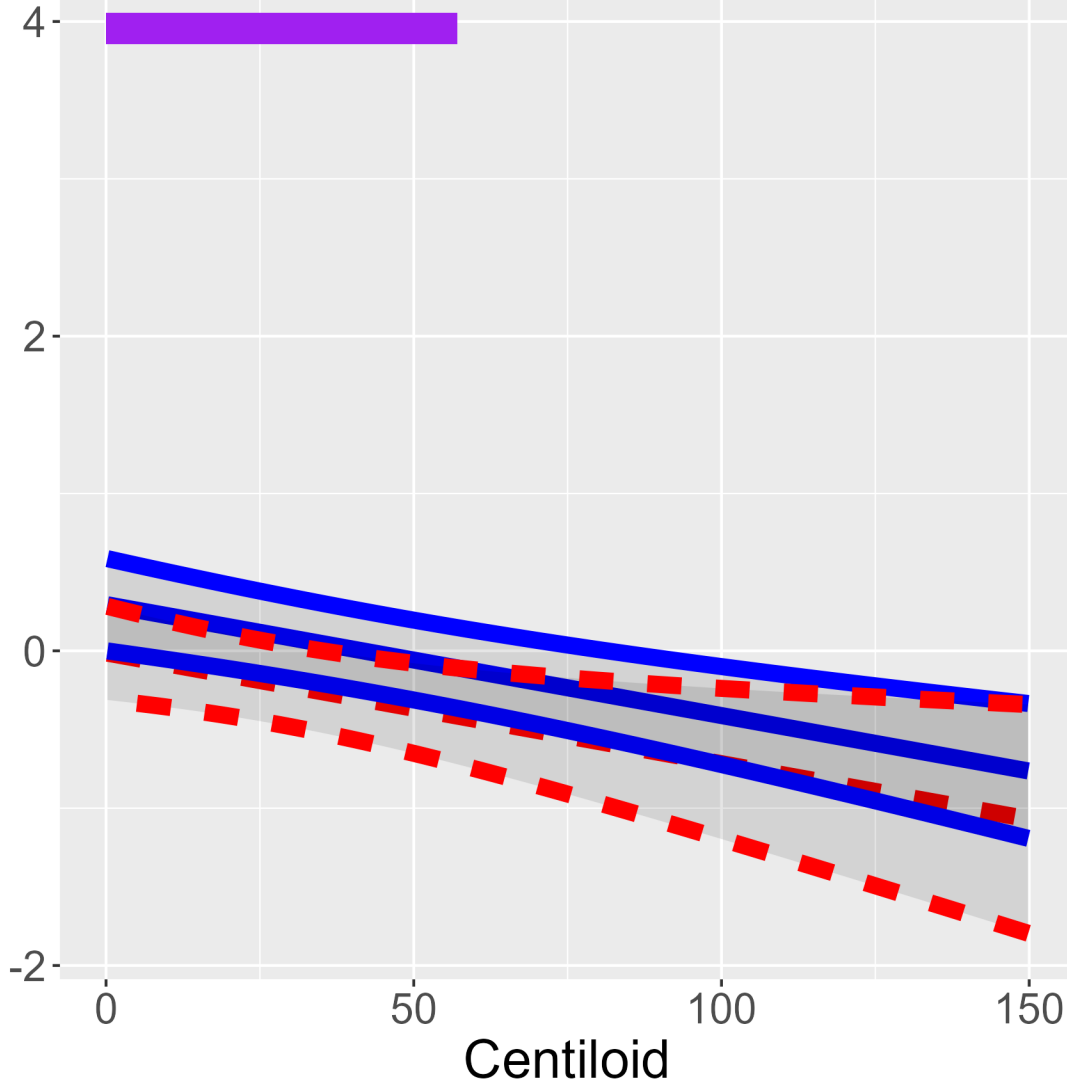

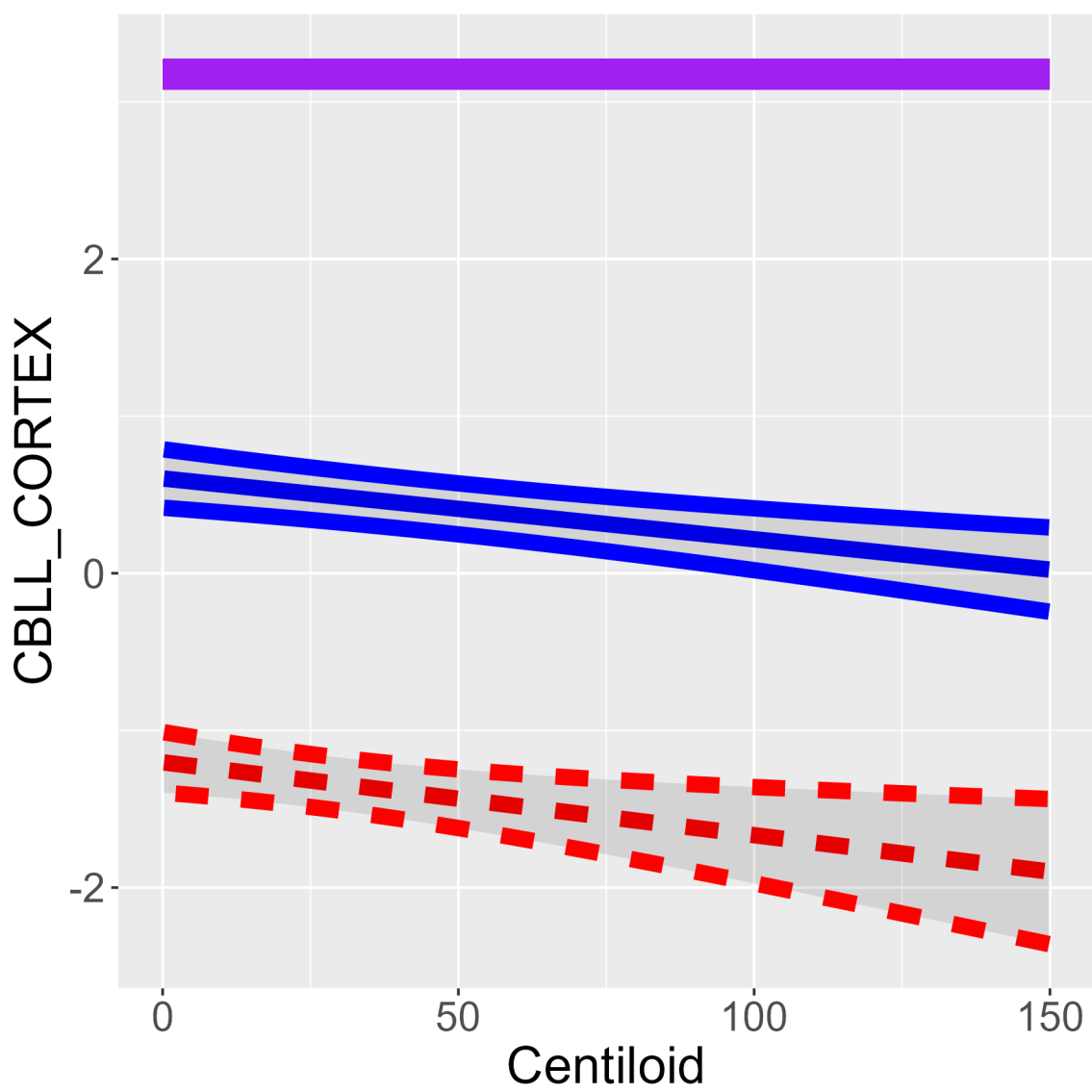

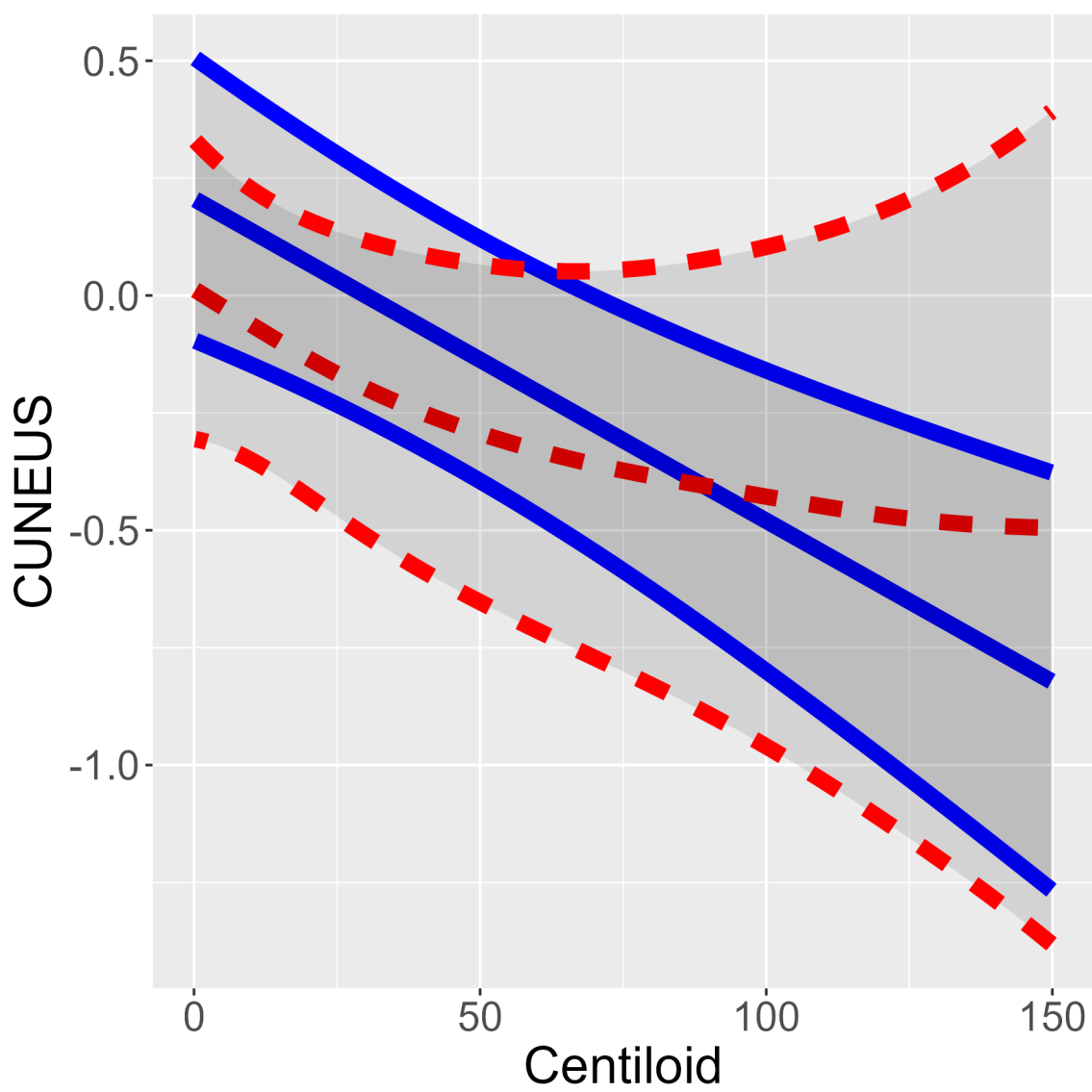

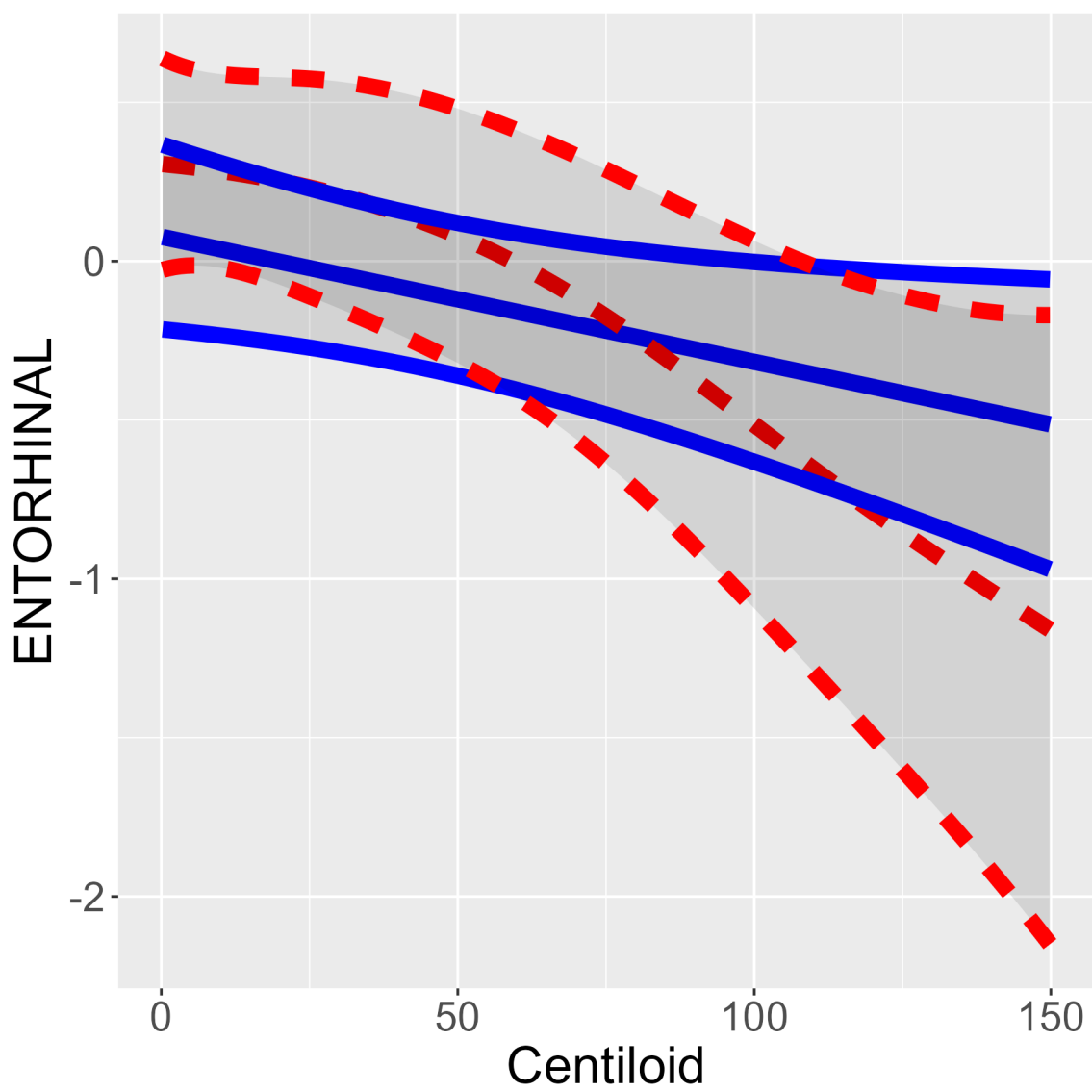

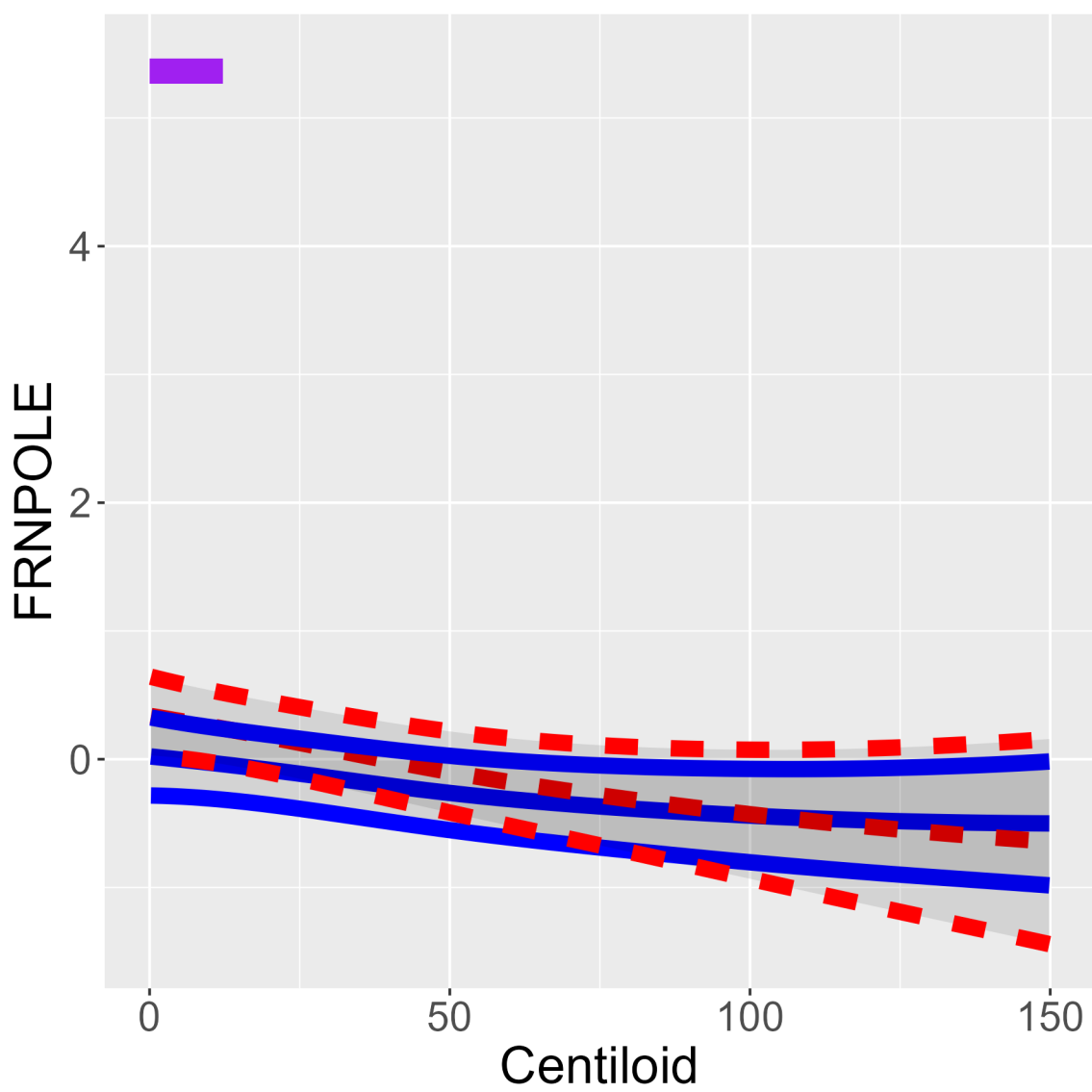

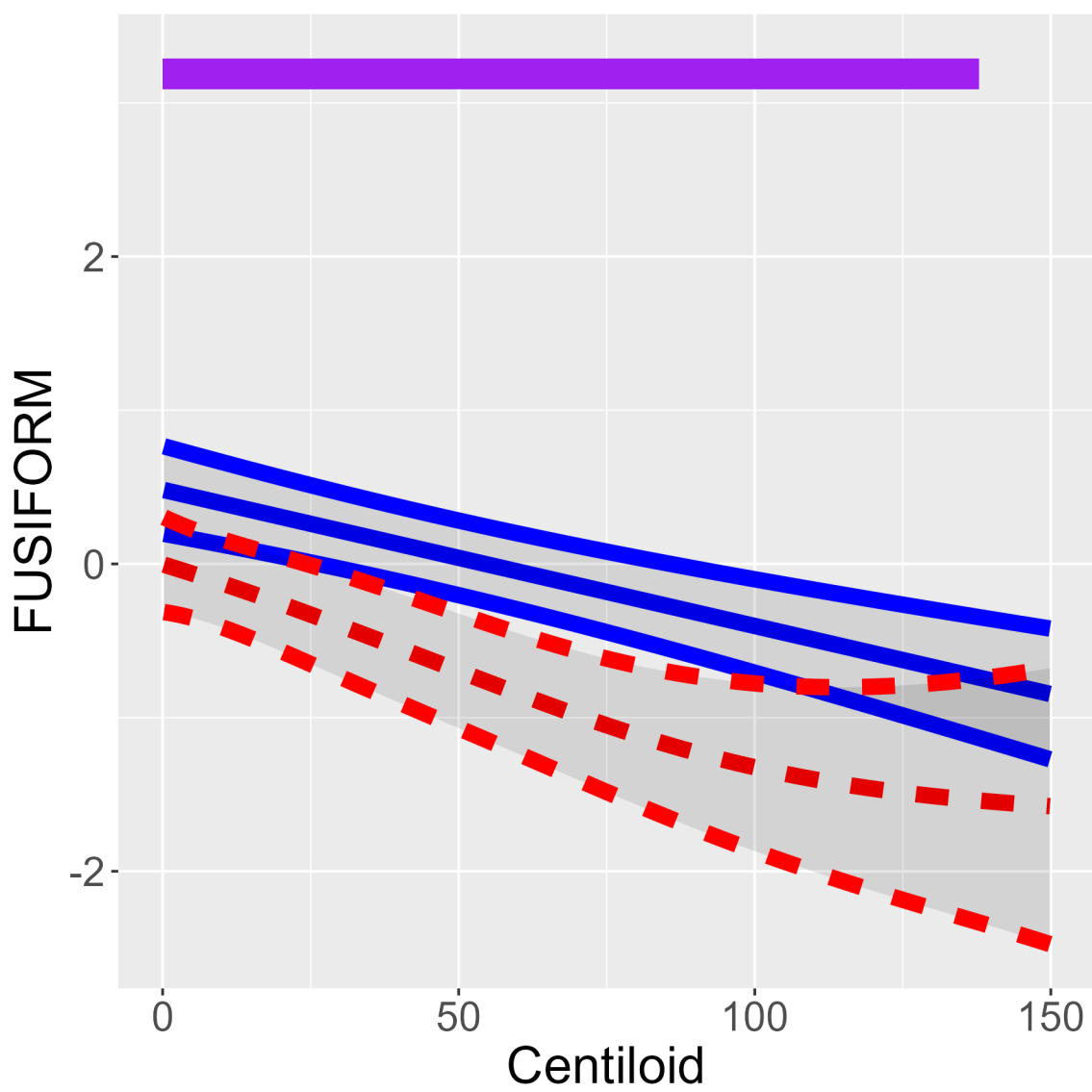

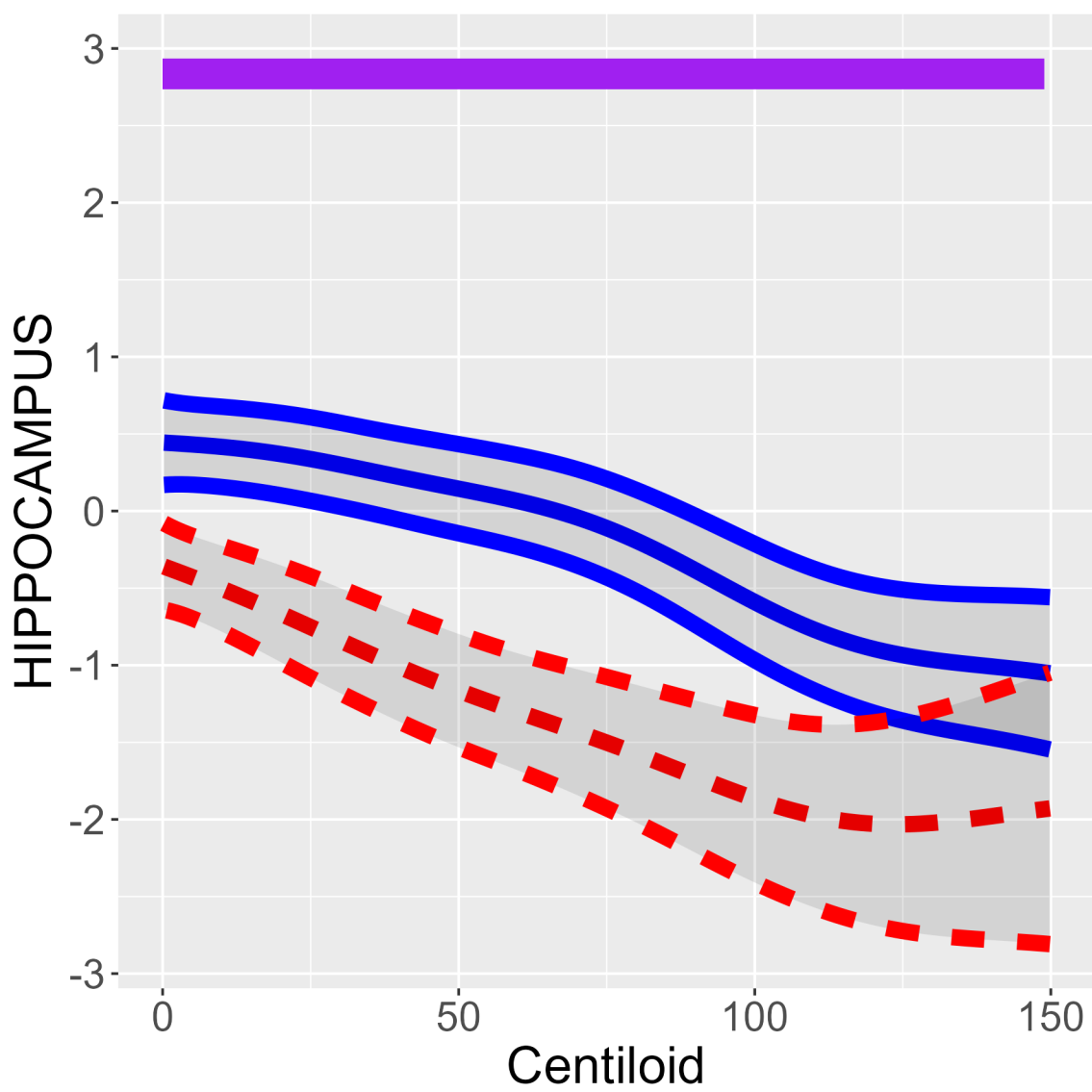

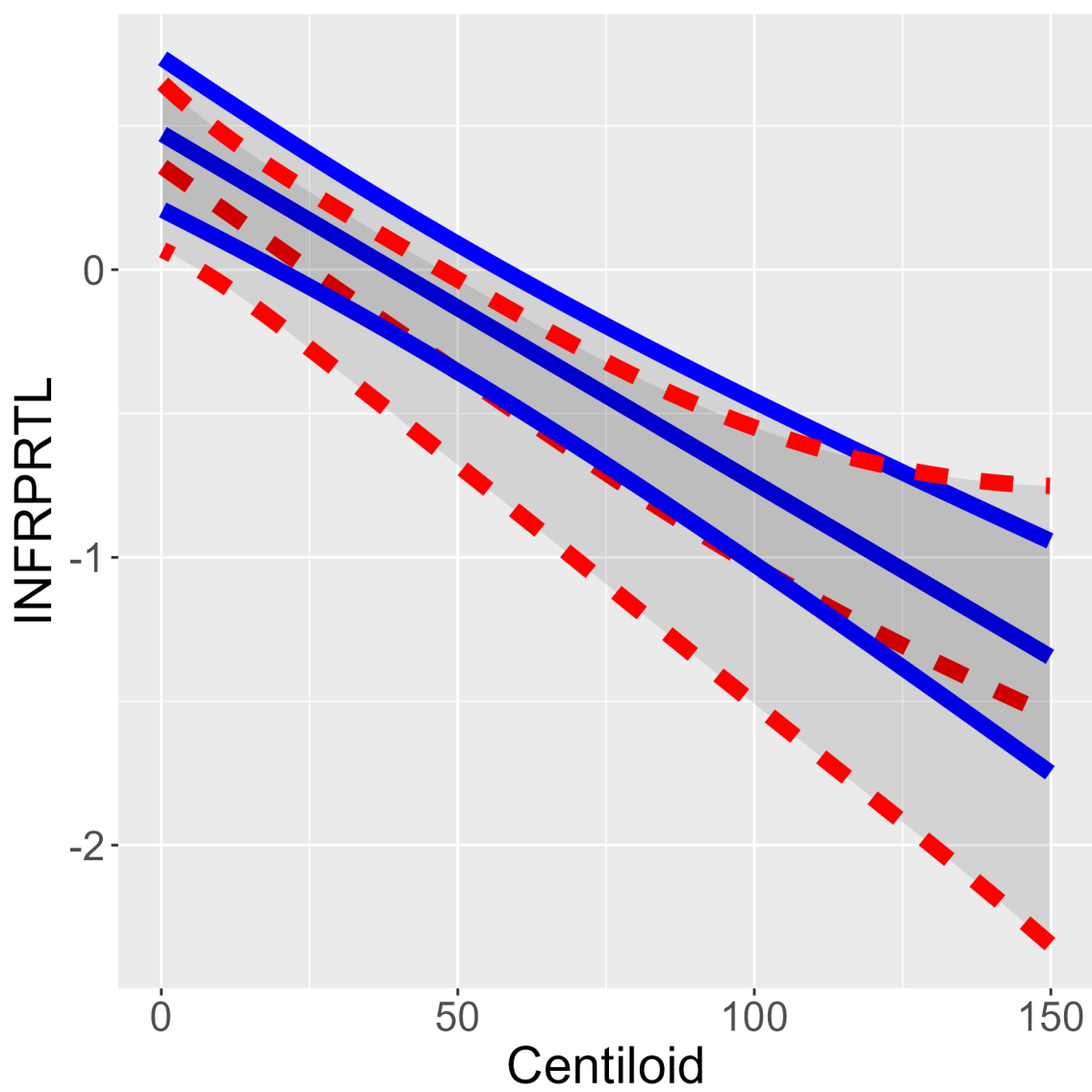

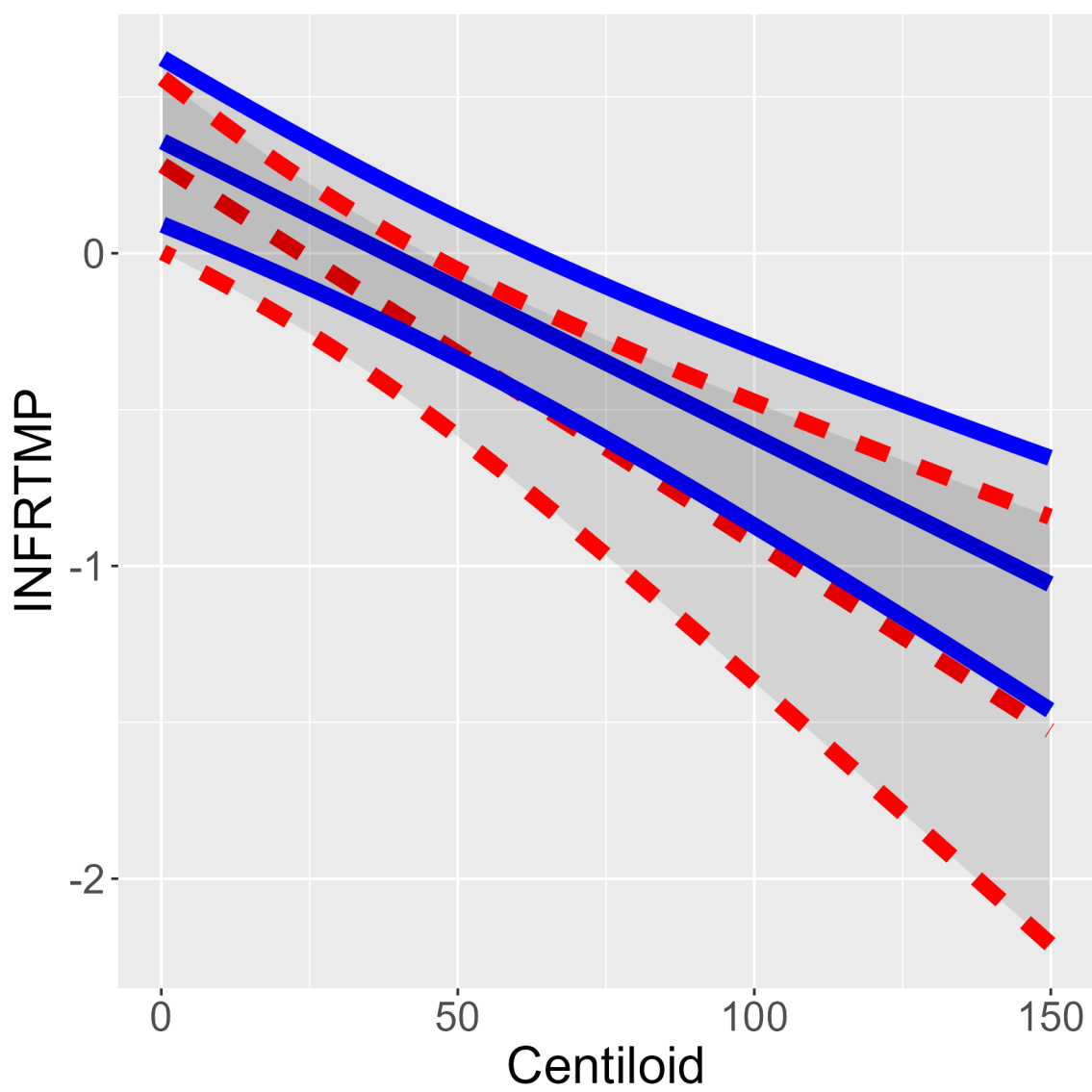

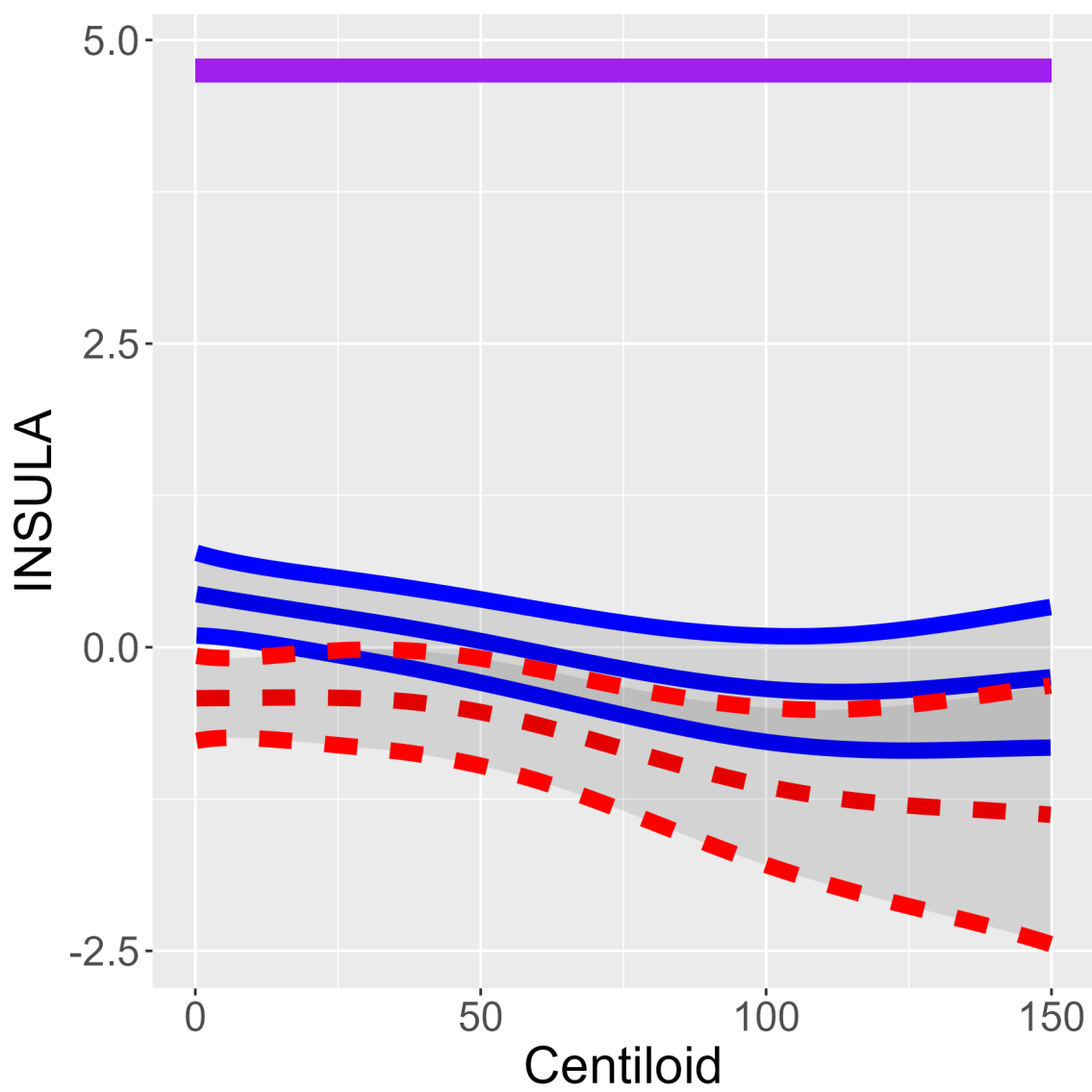

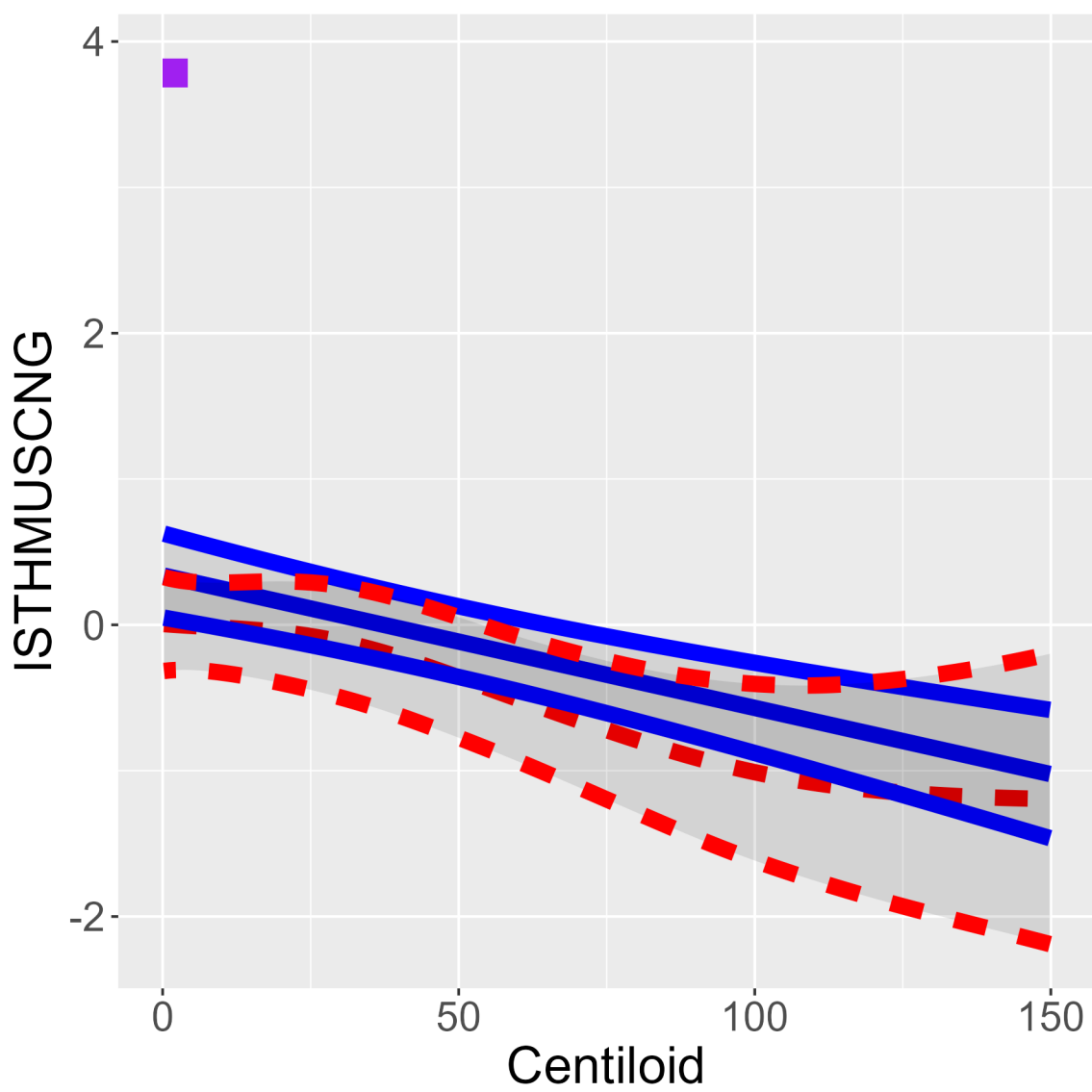

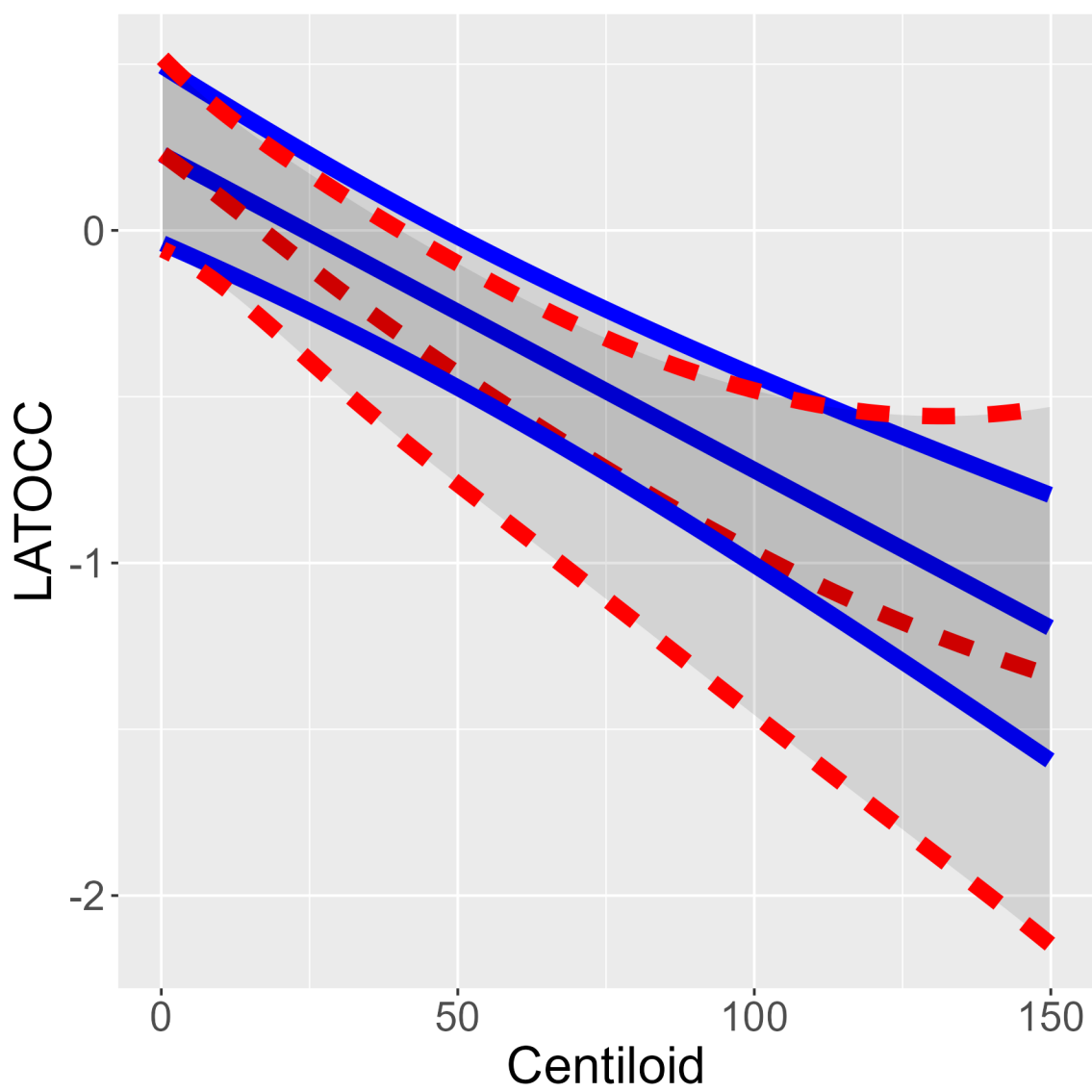

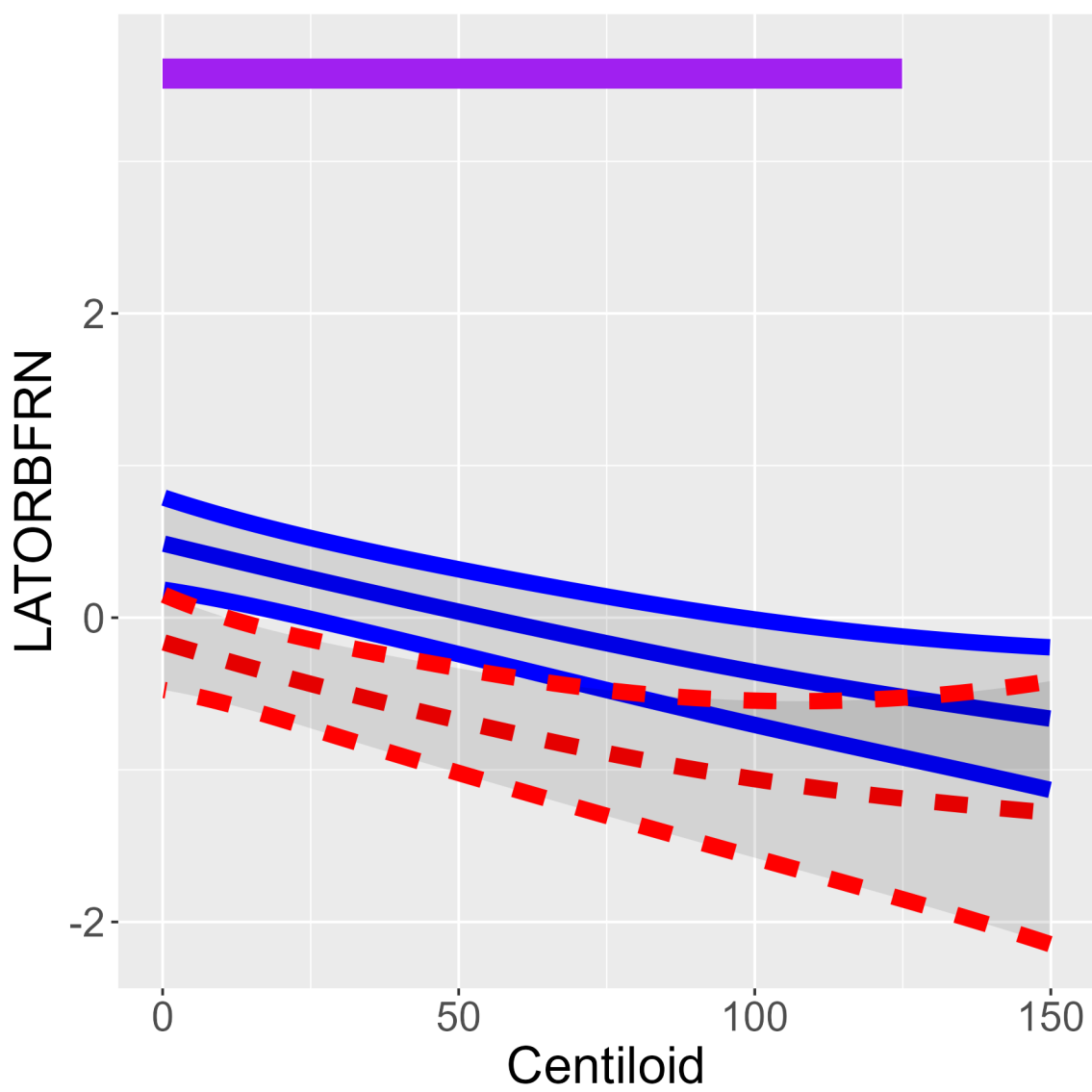

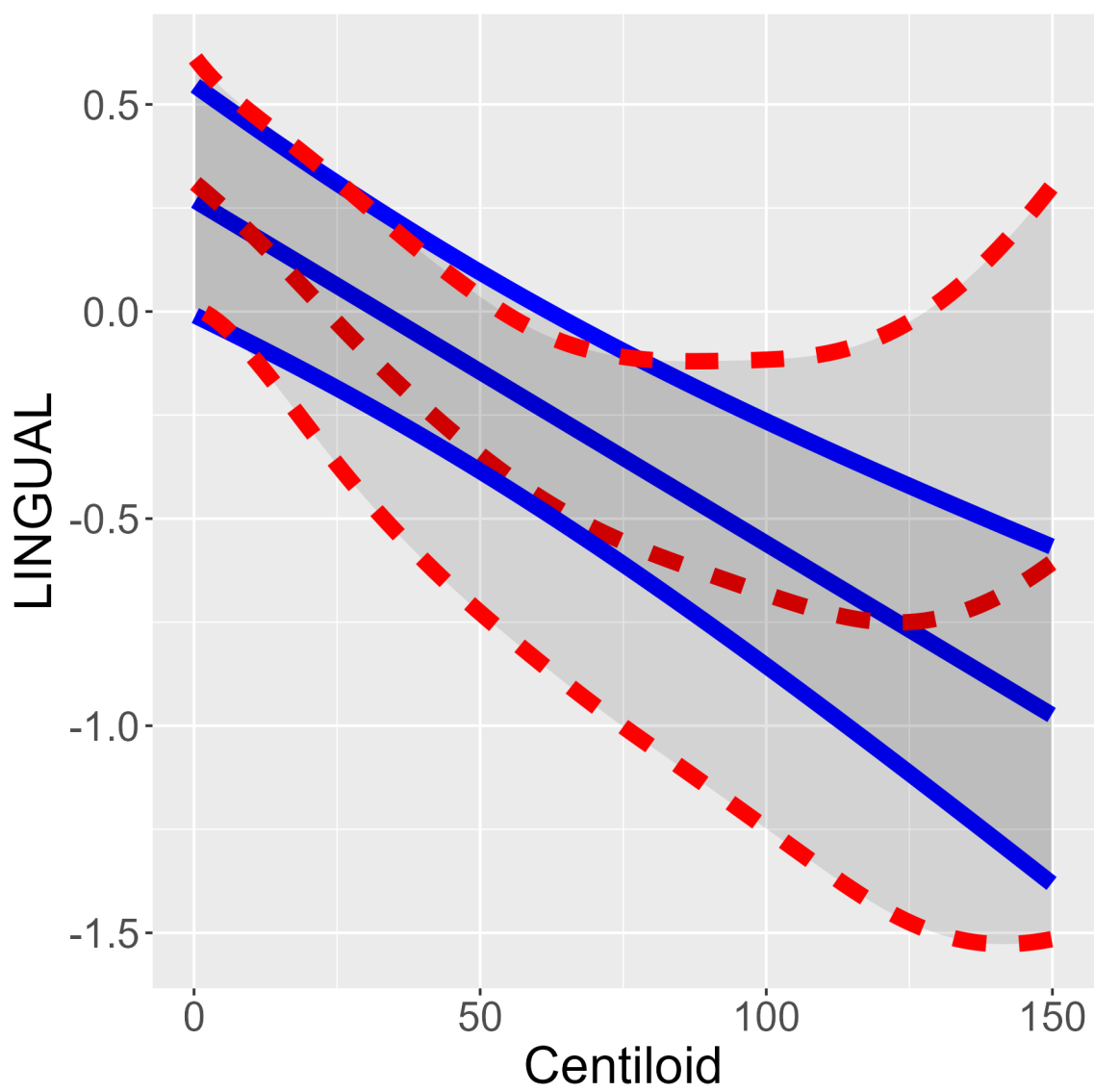

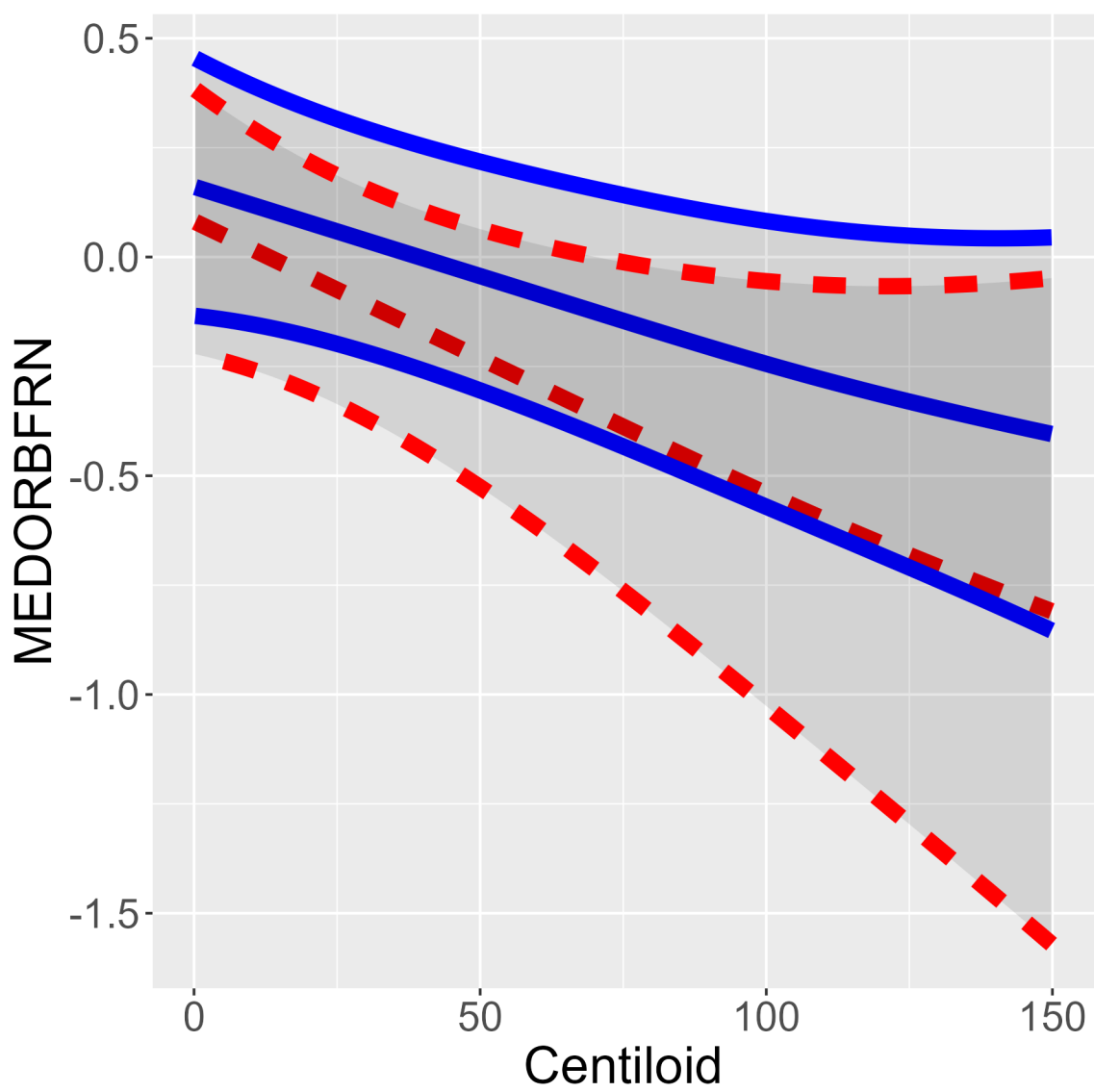

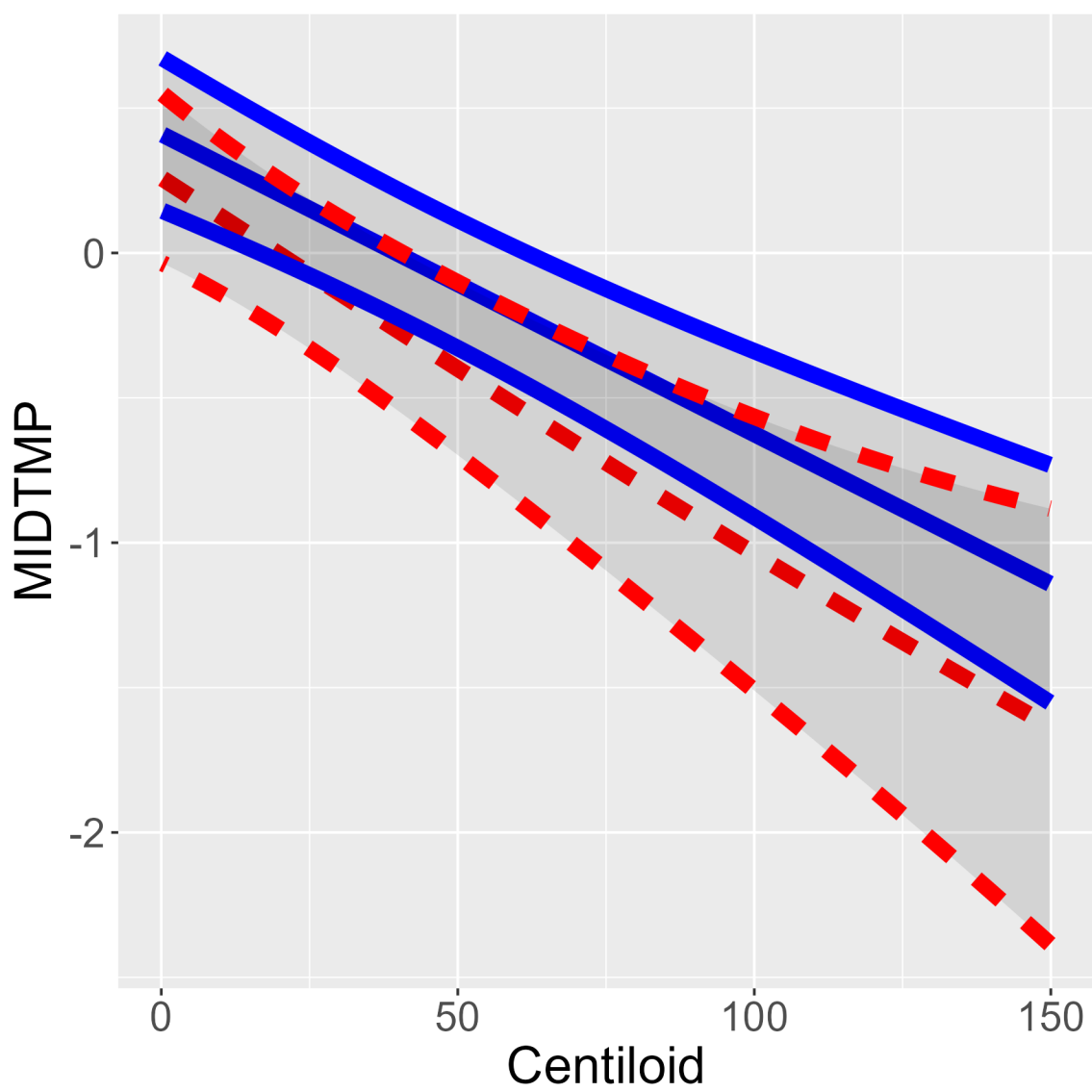

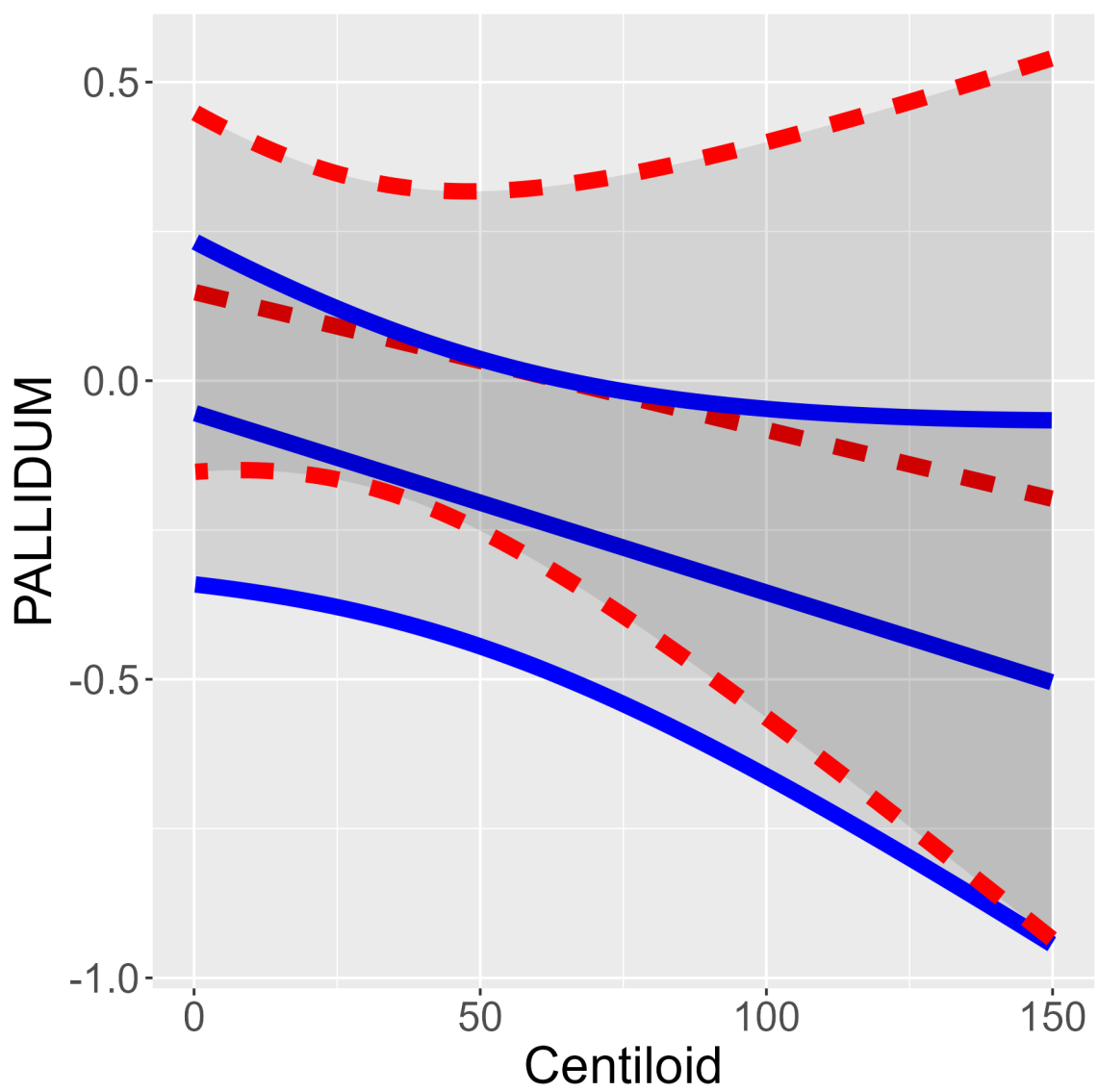

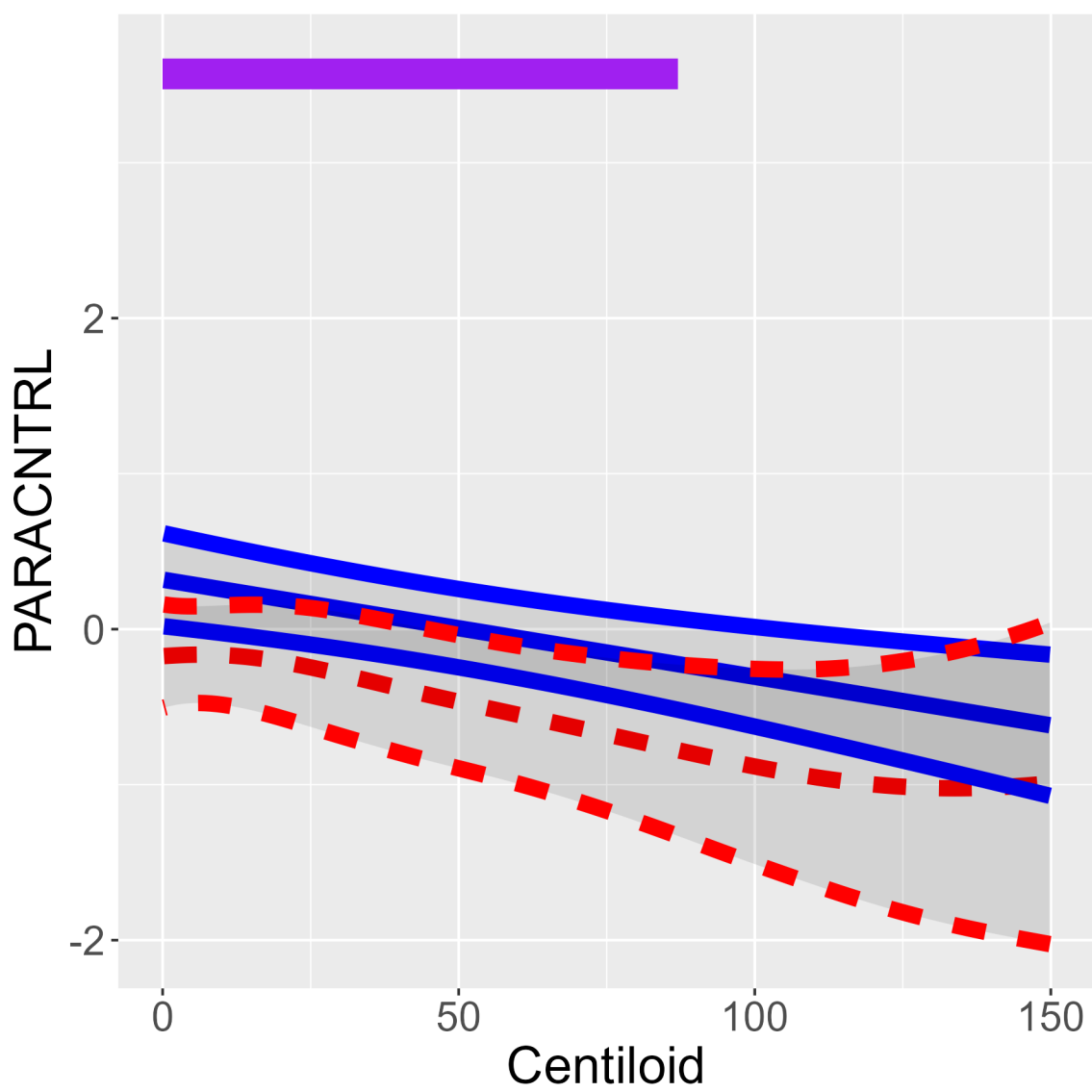

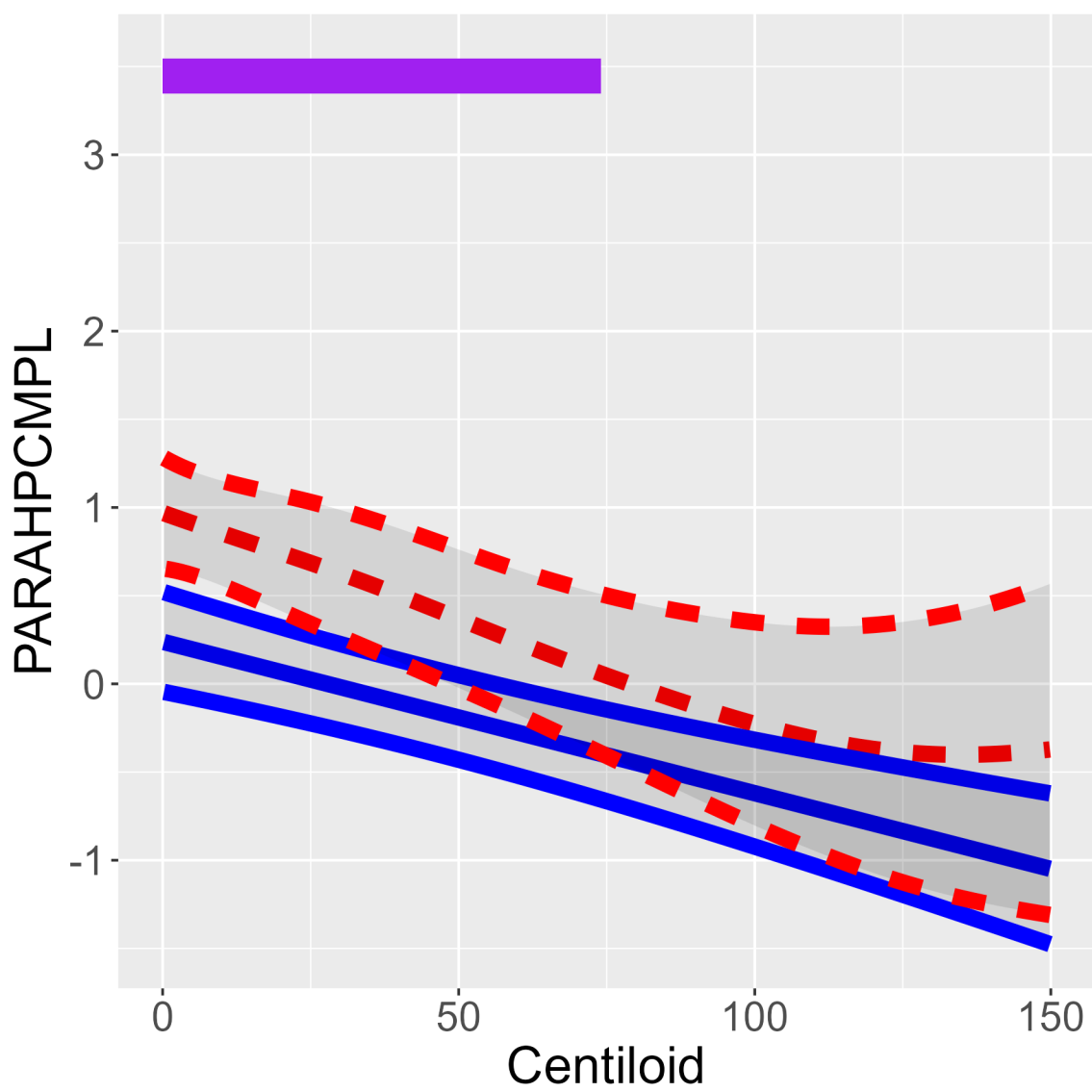

PARAOPRCLRS

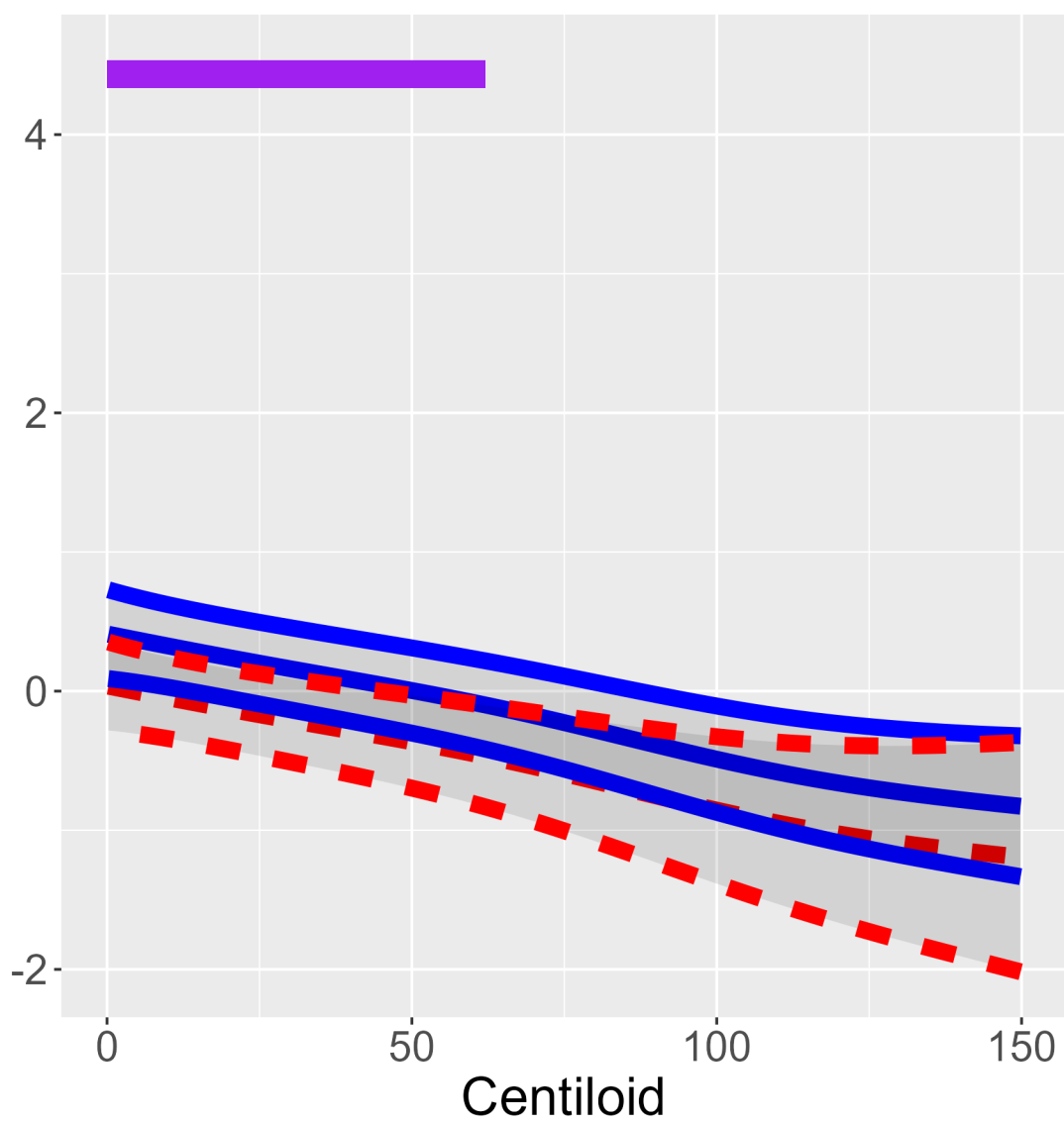

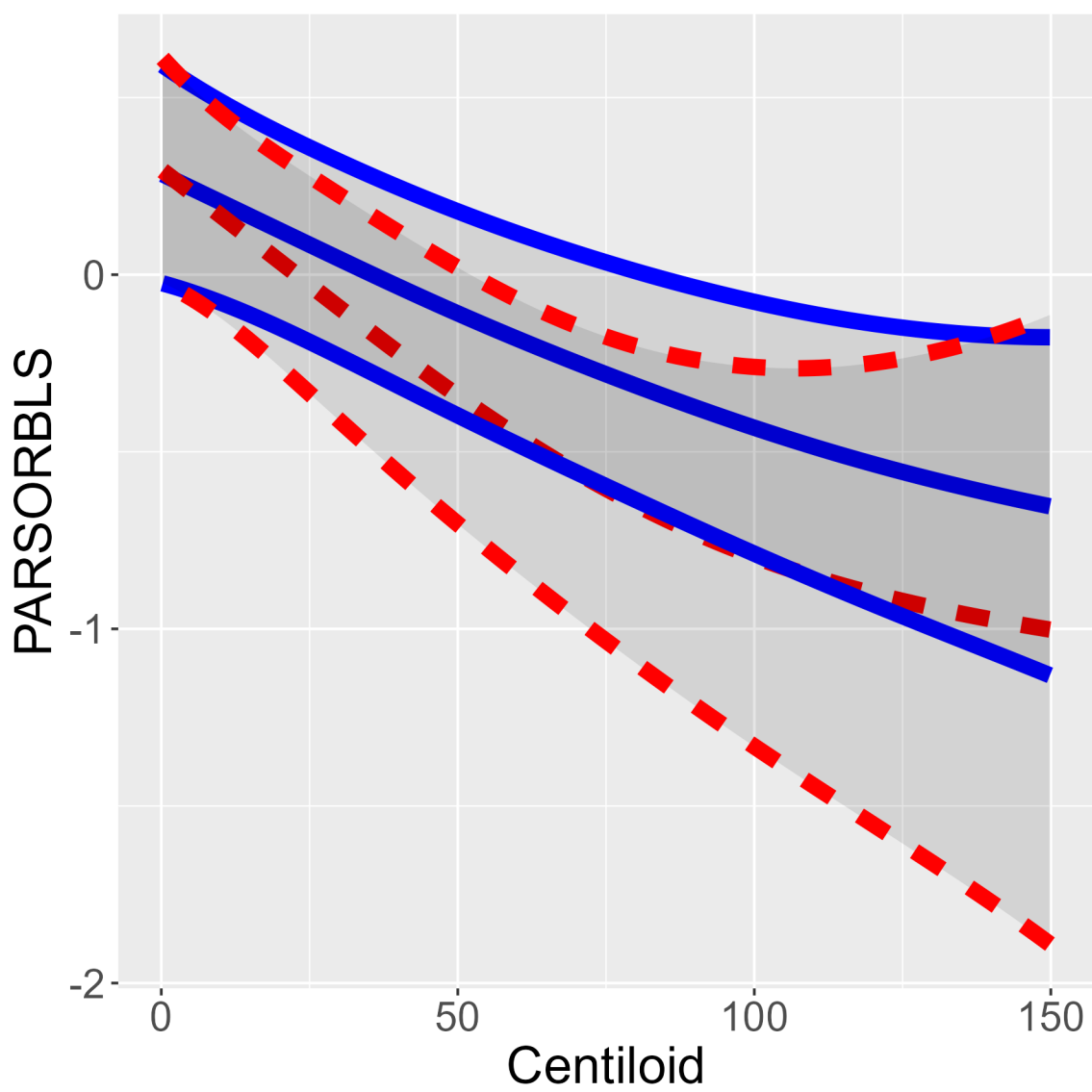

PARSTRNGLRS

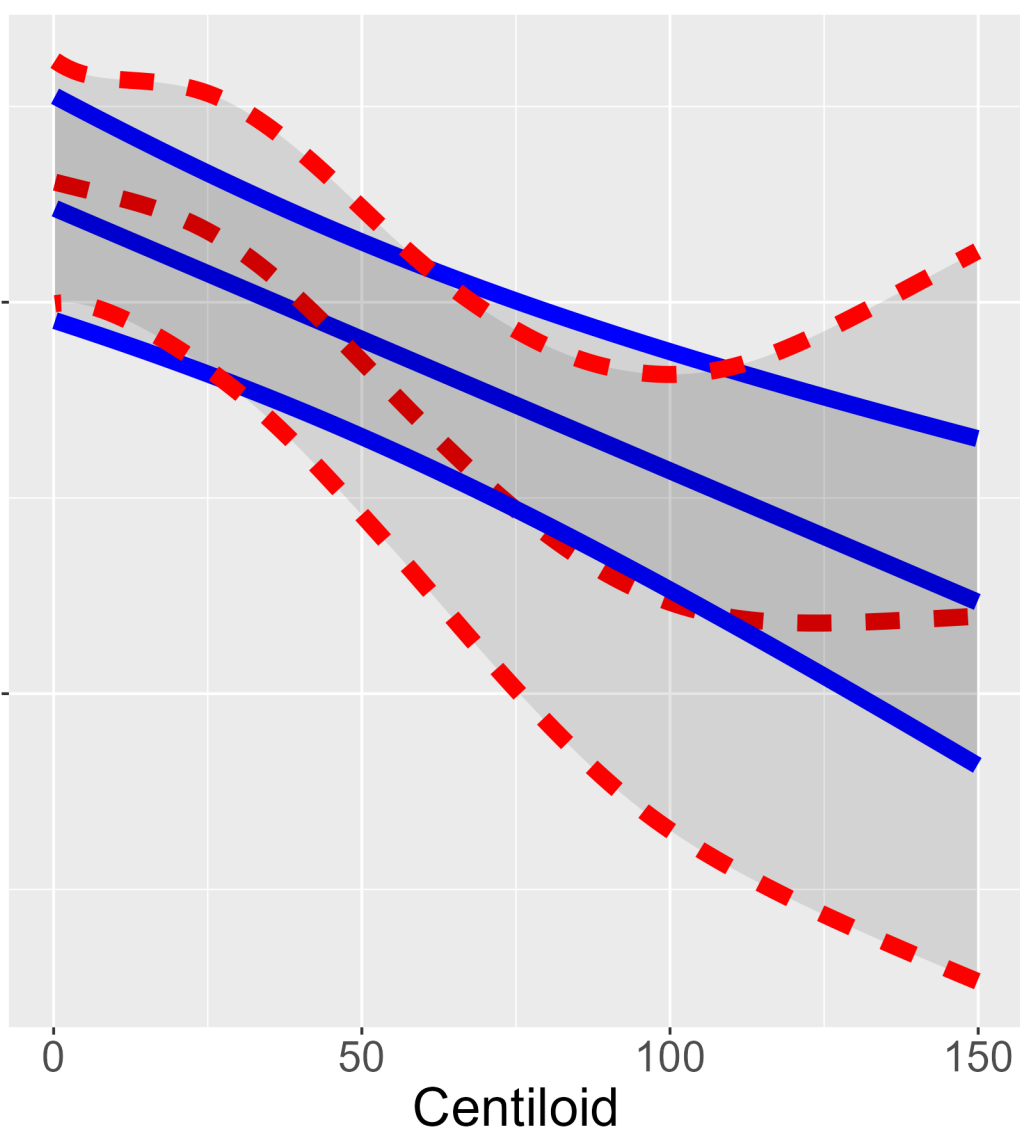

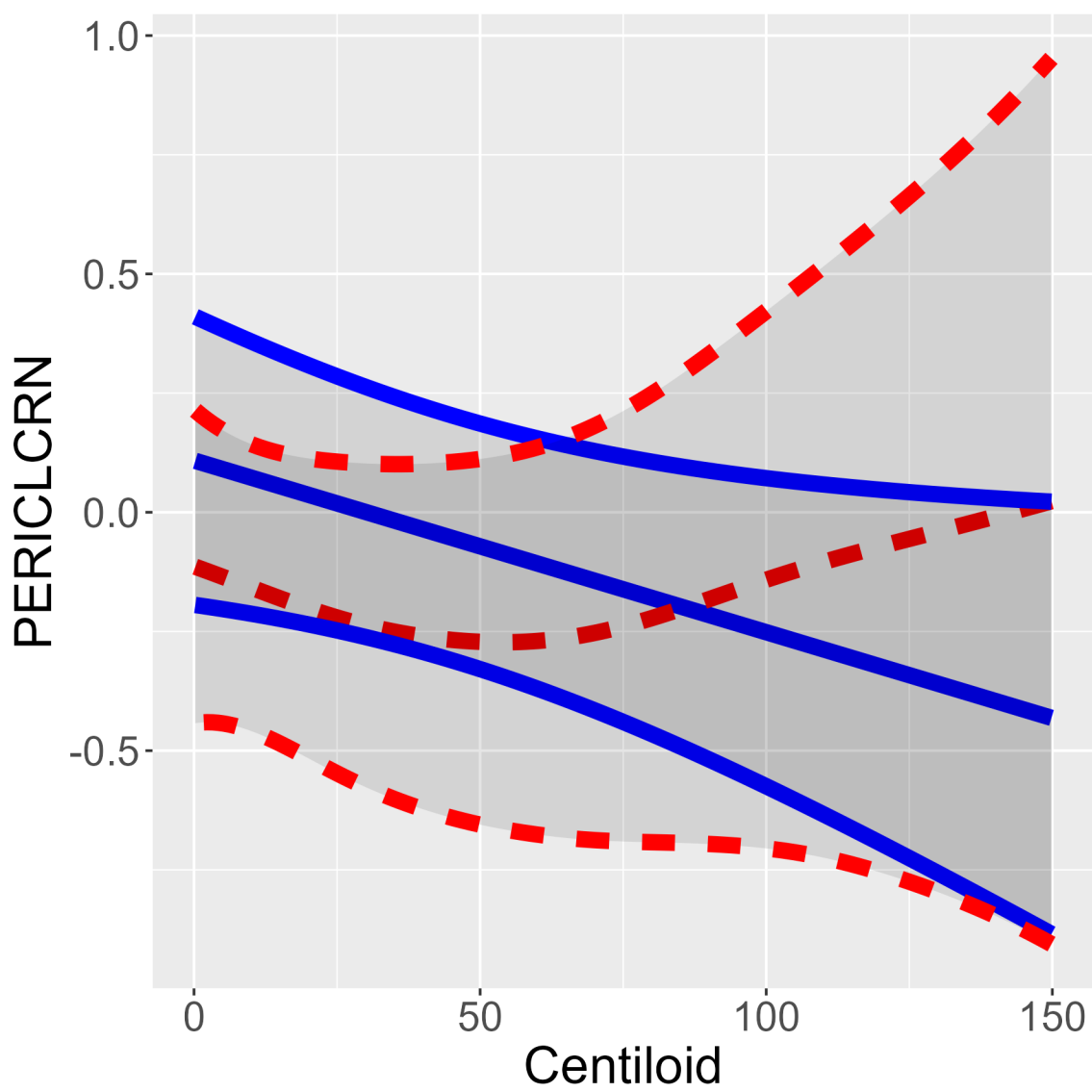

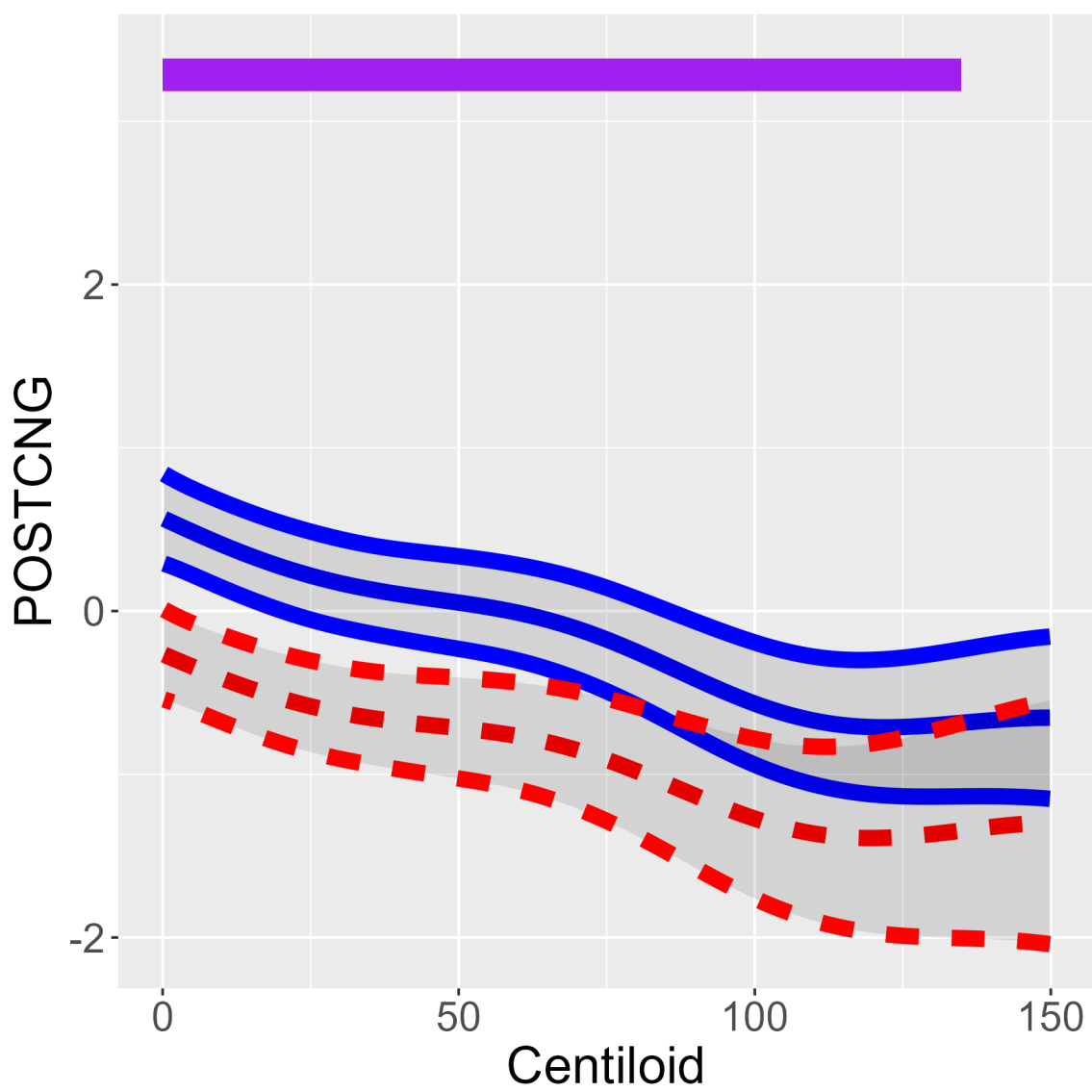

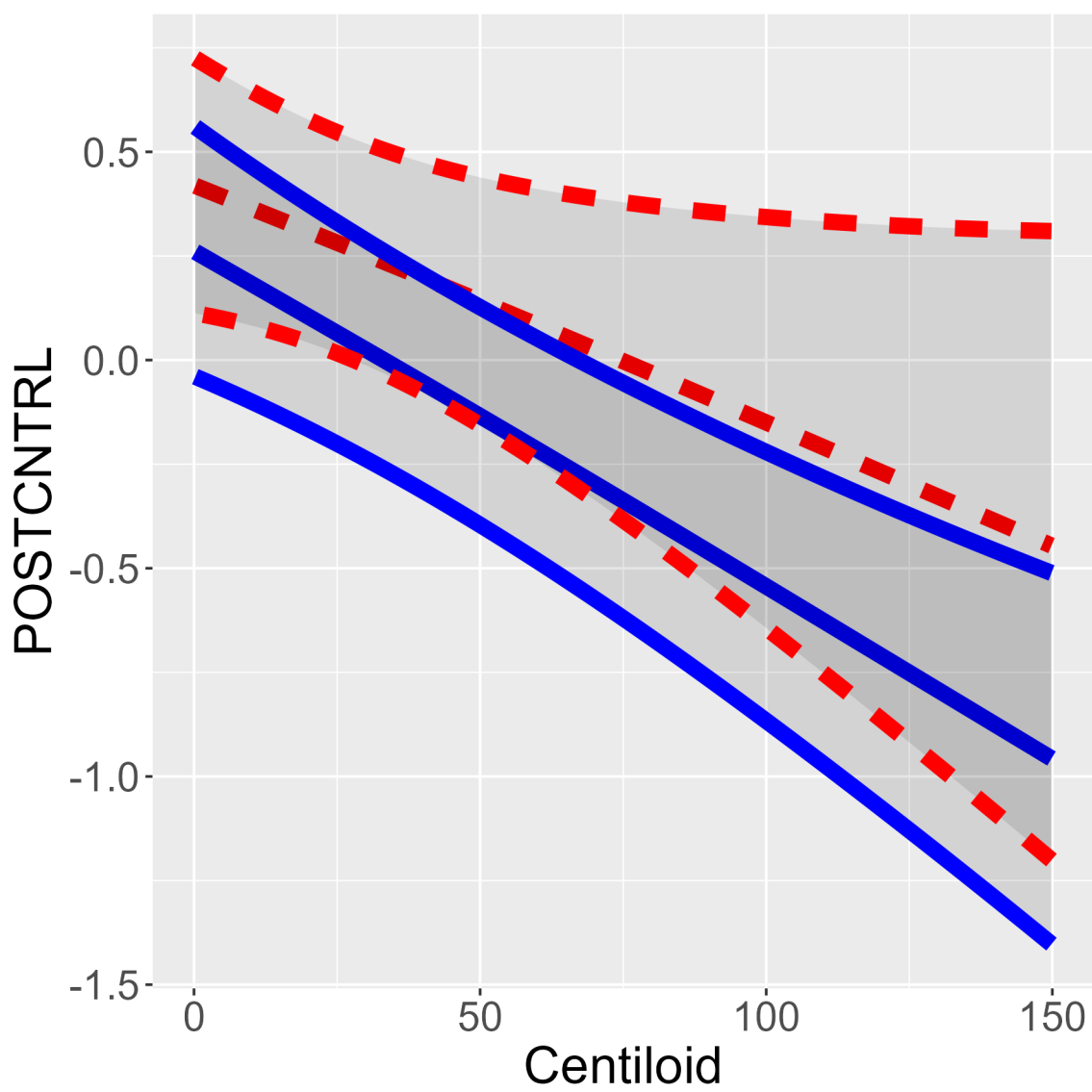

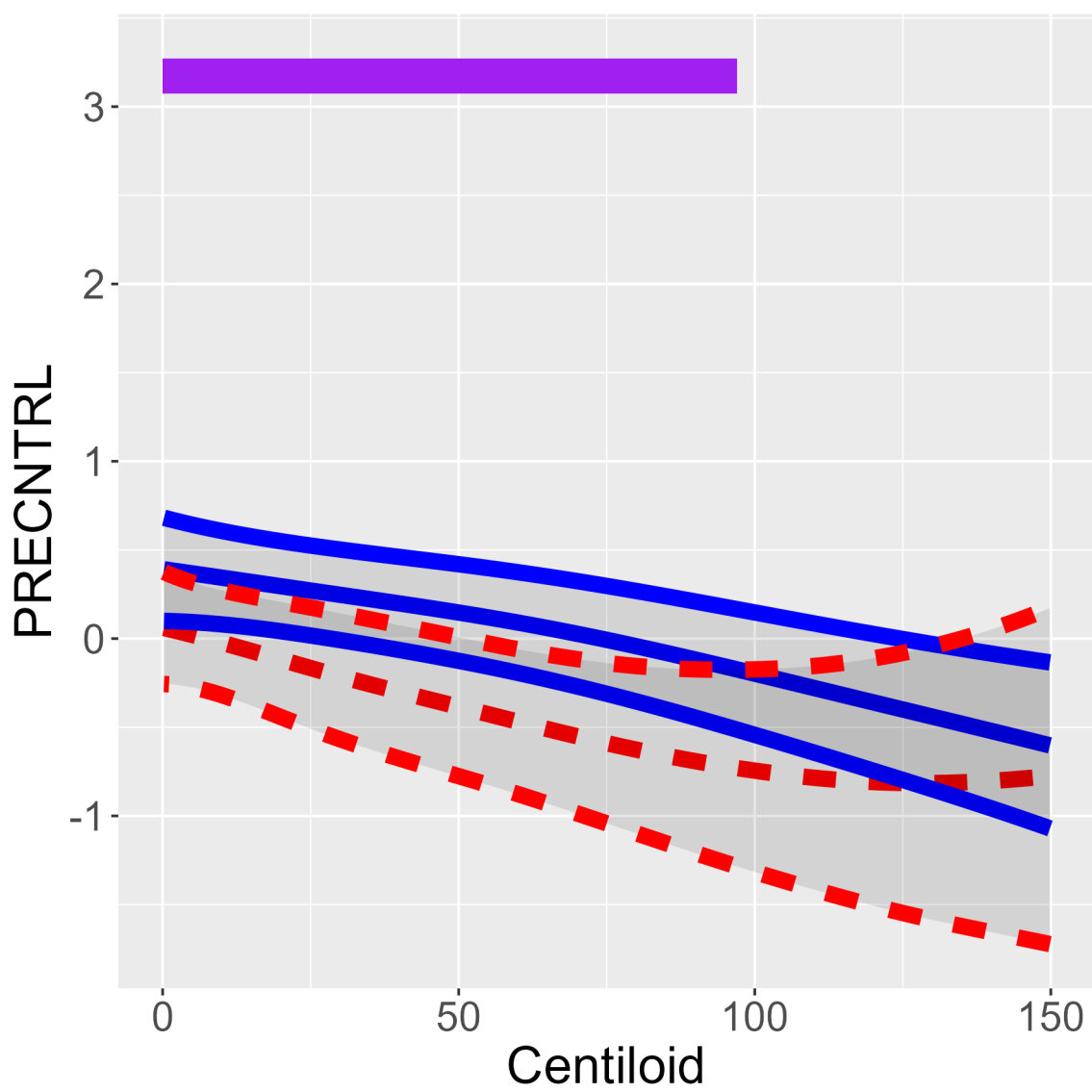

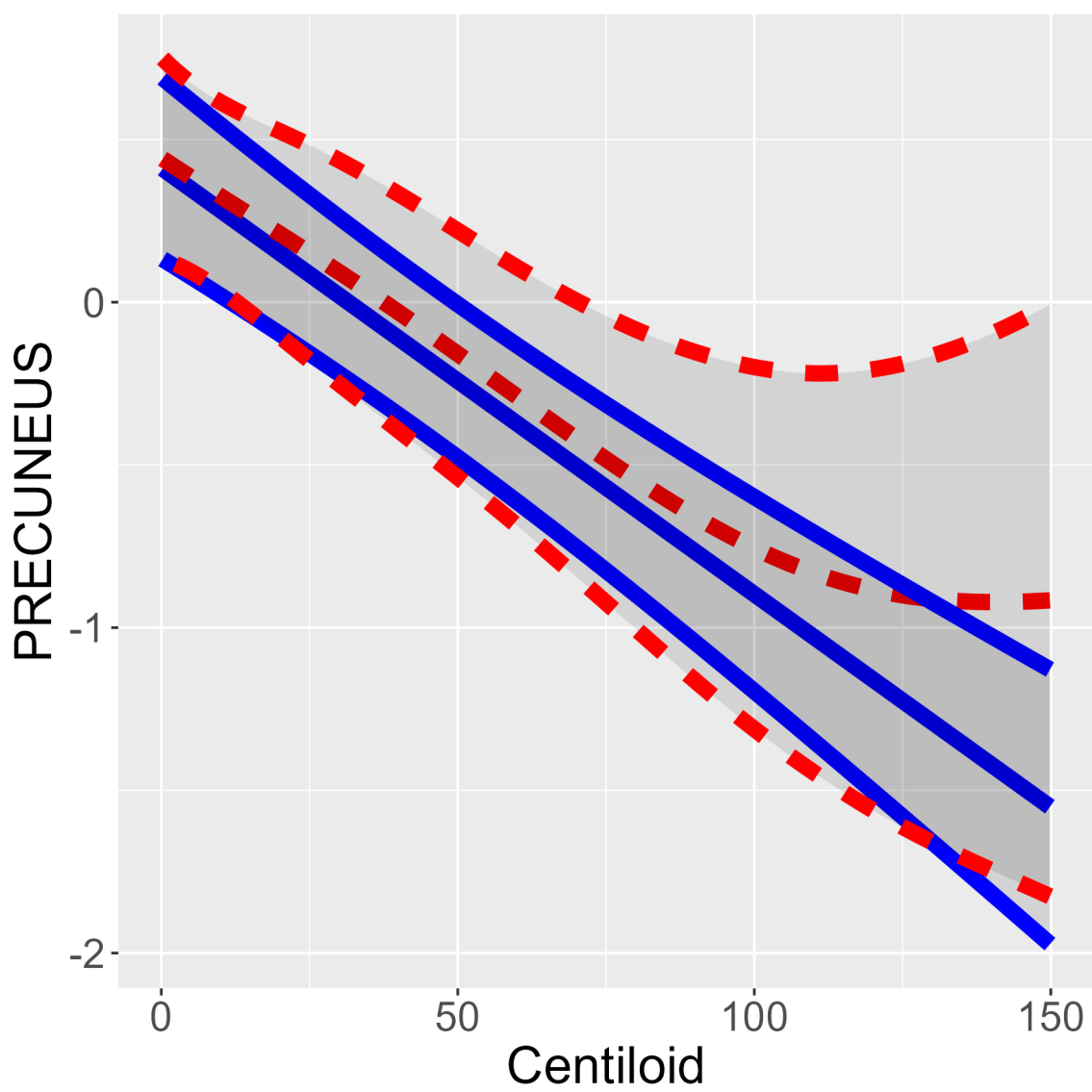

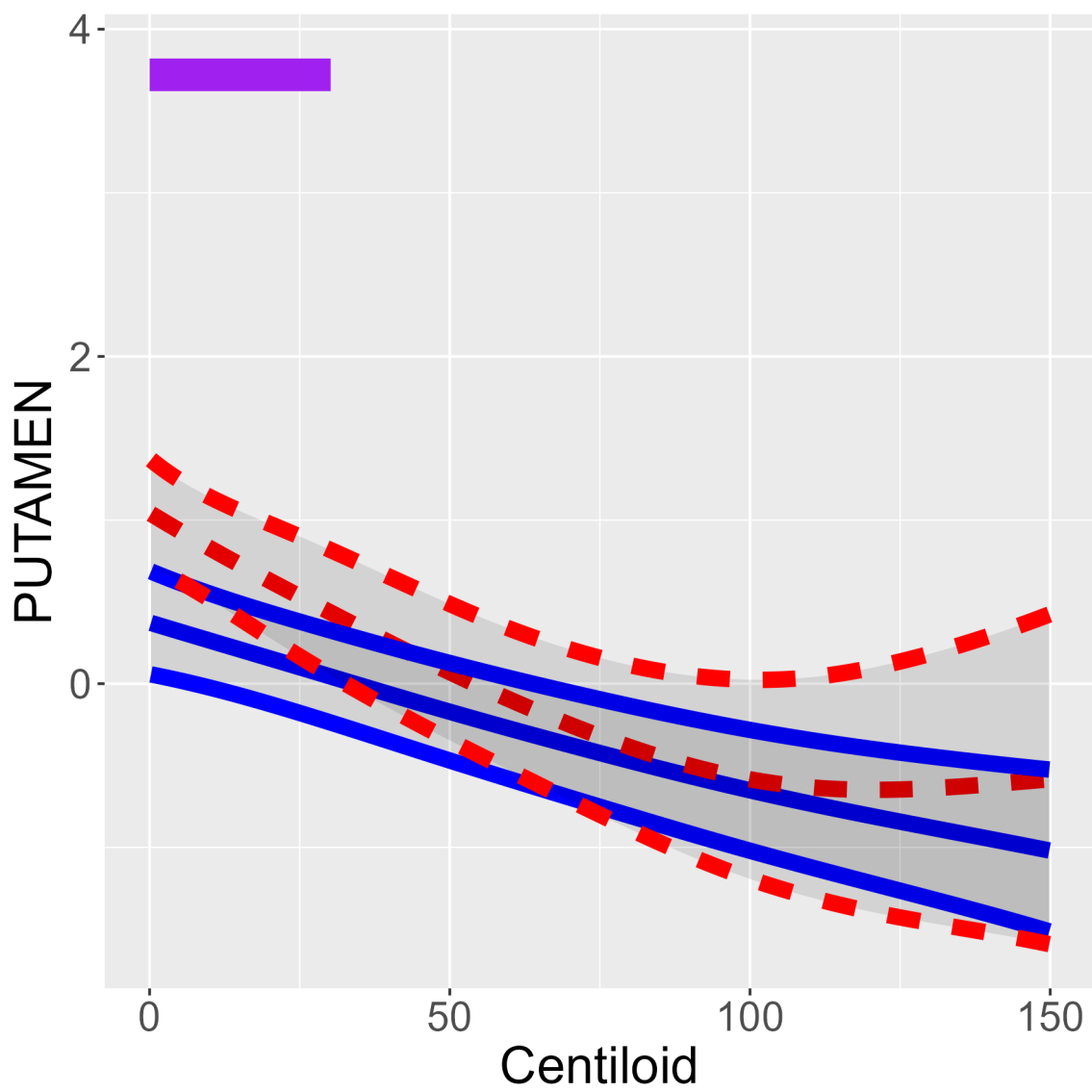

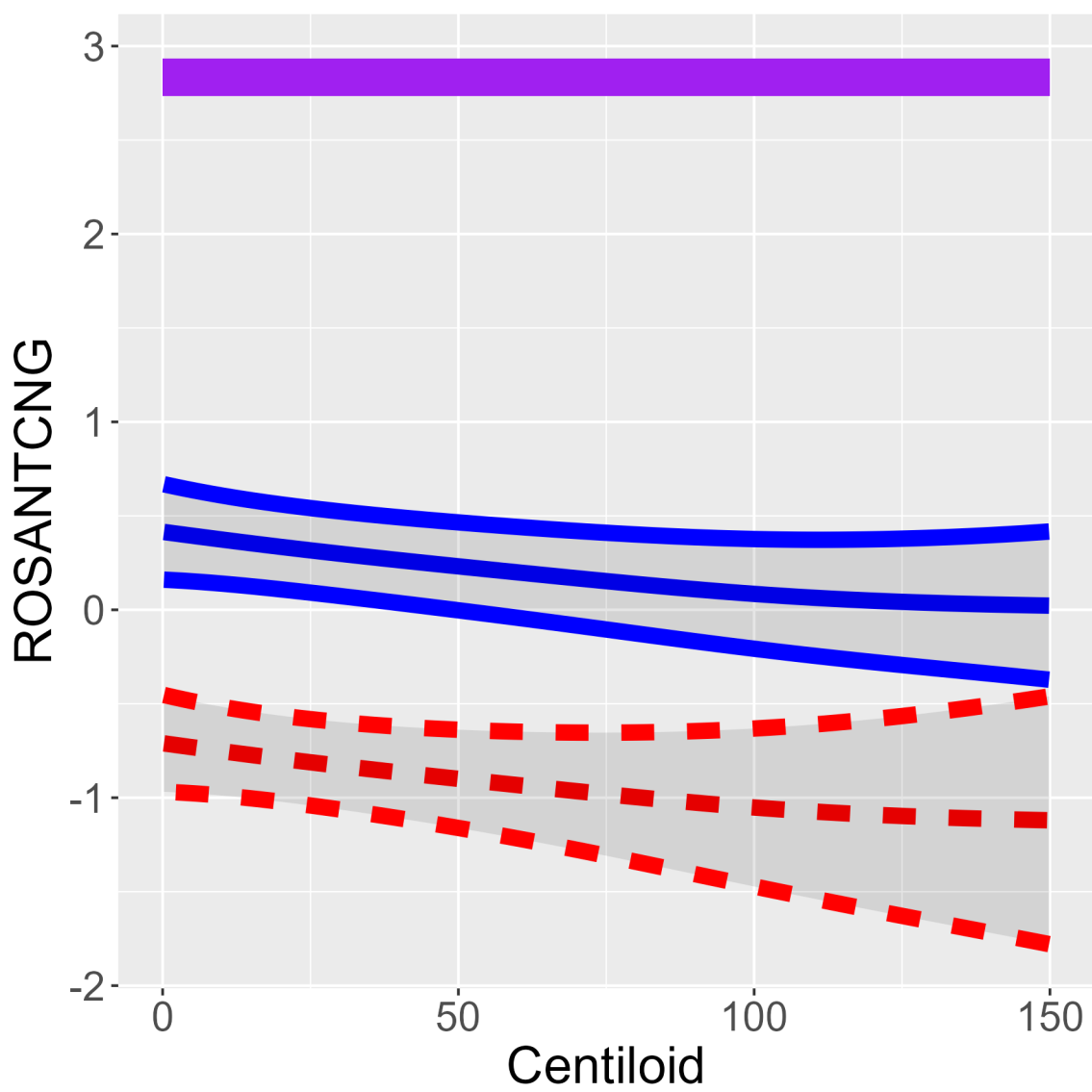

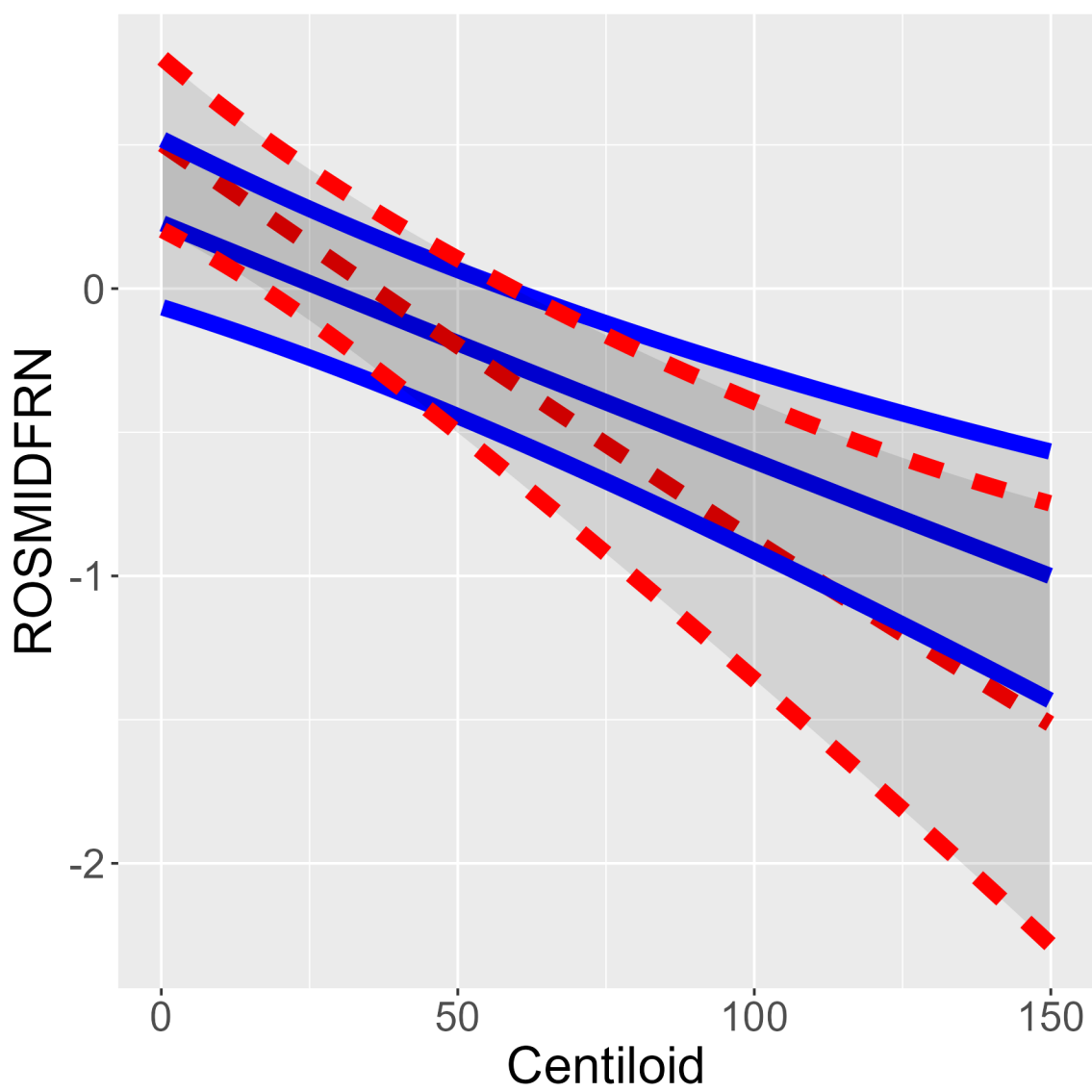

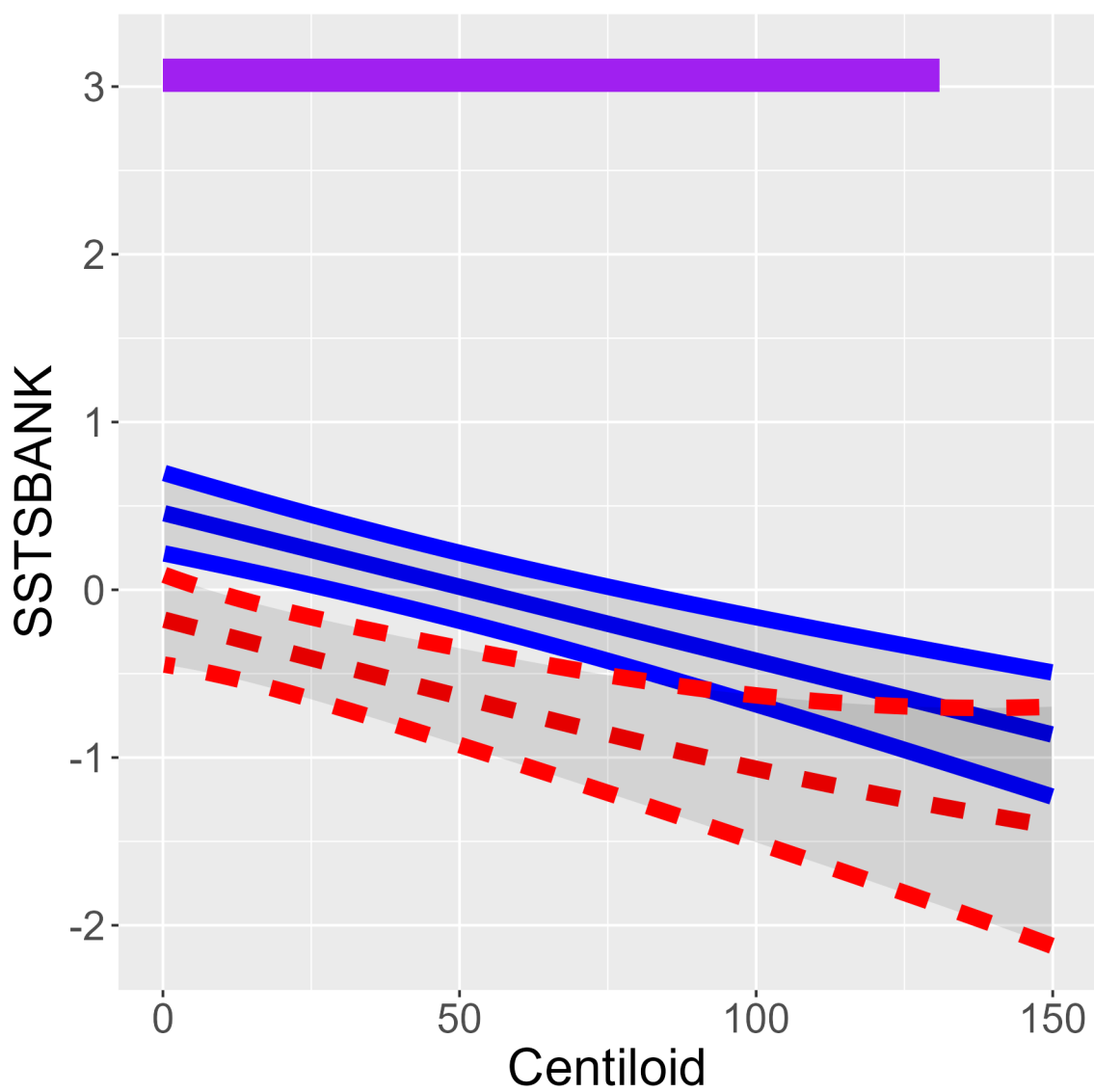

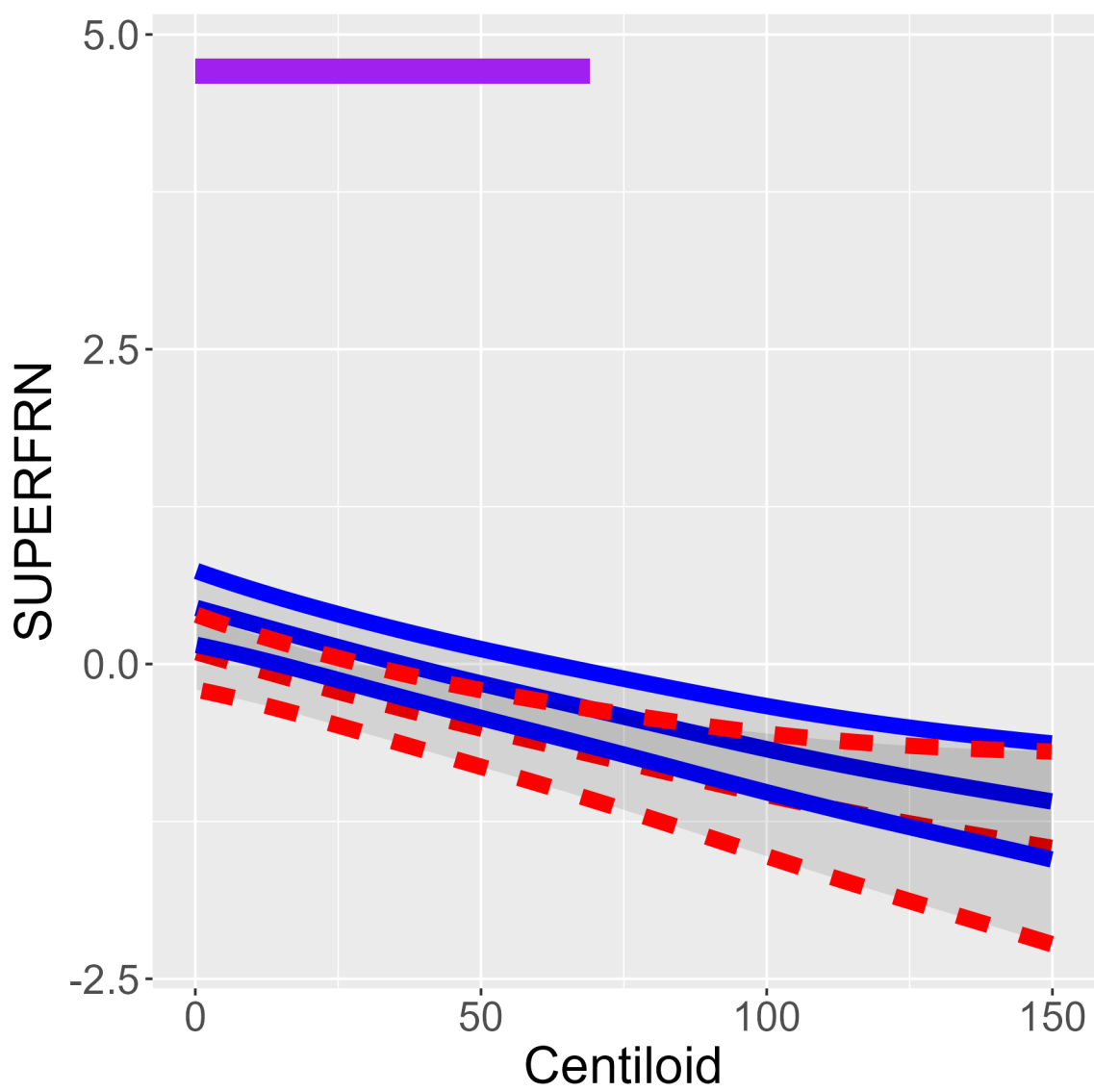

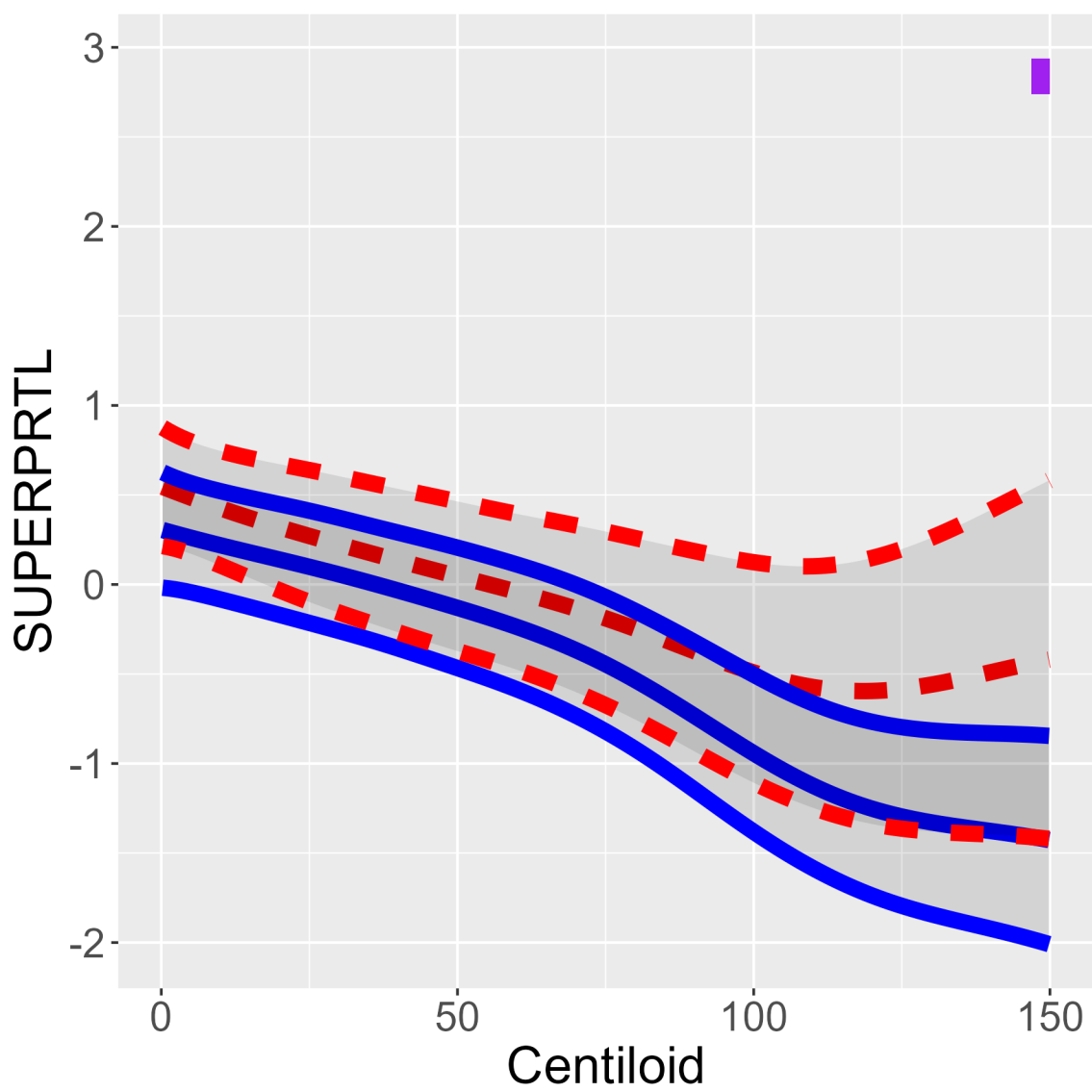

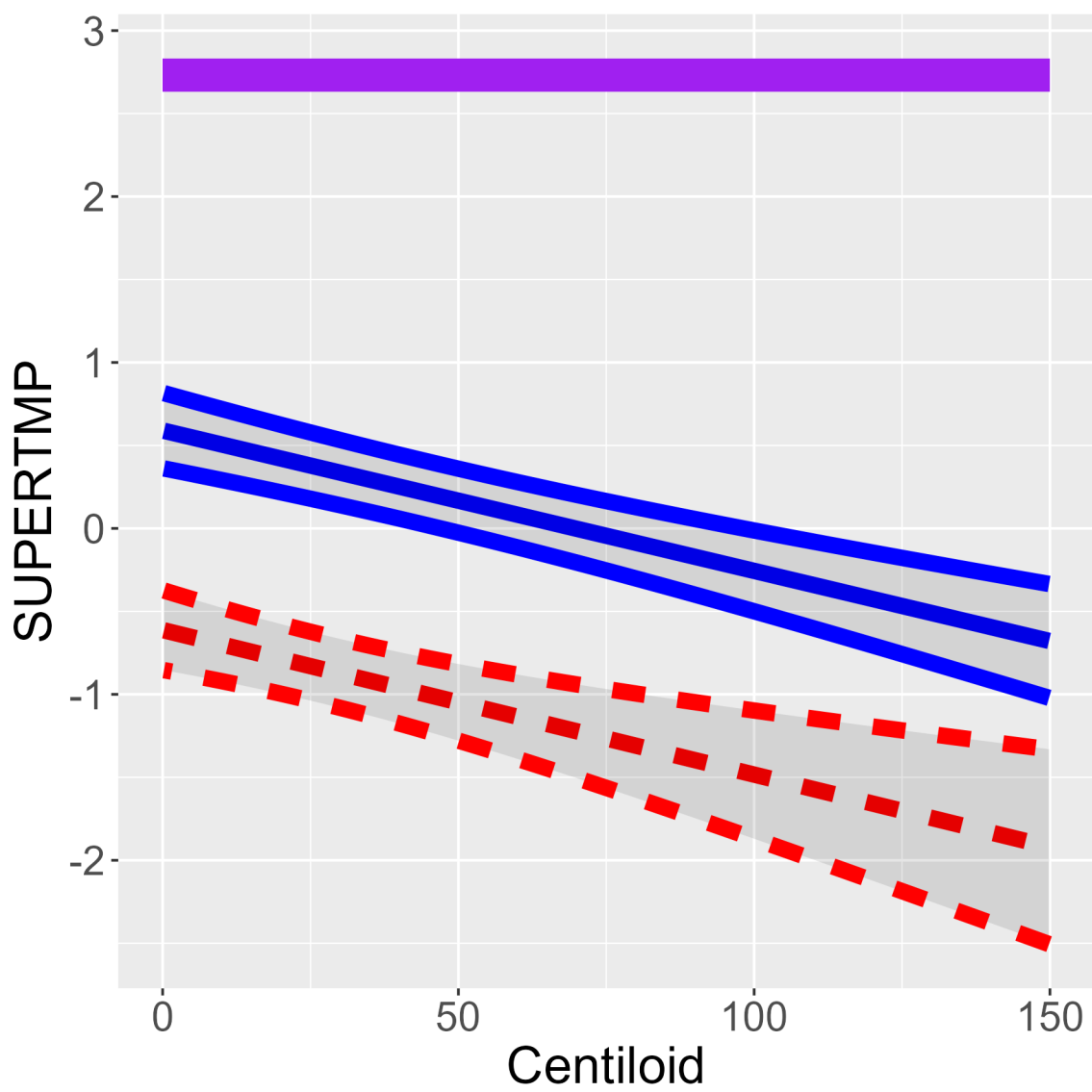

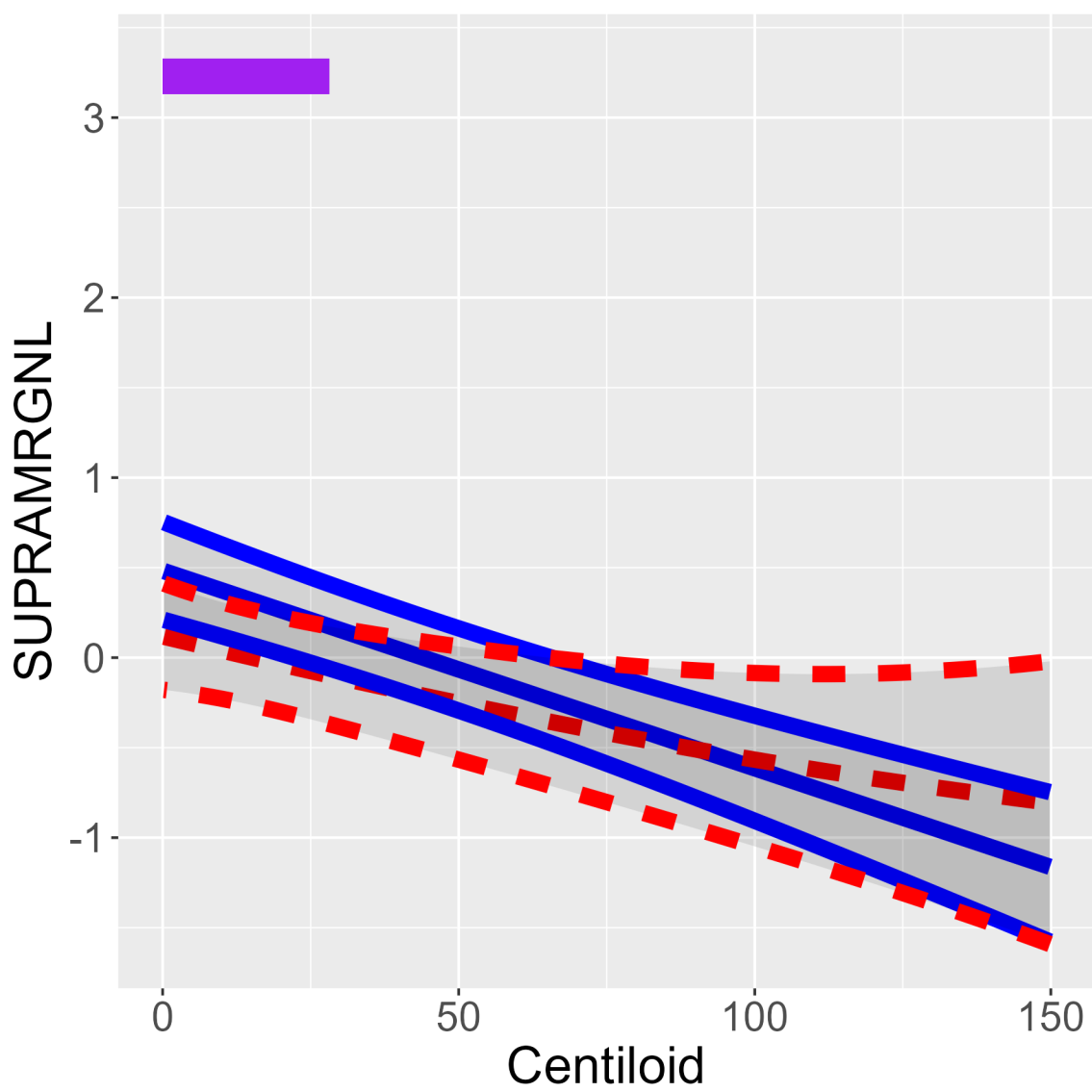

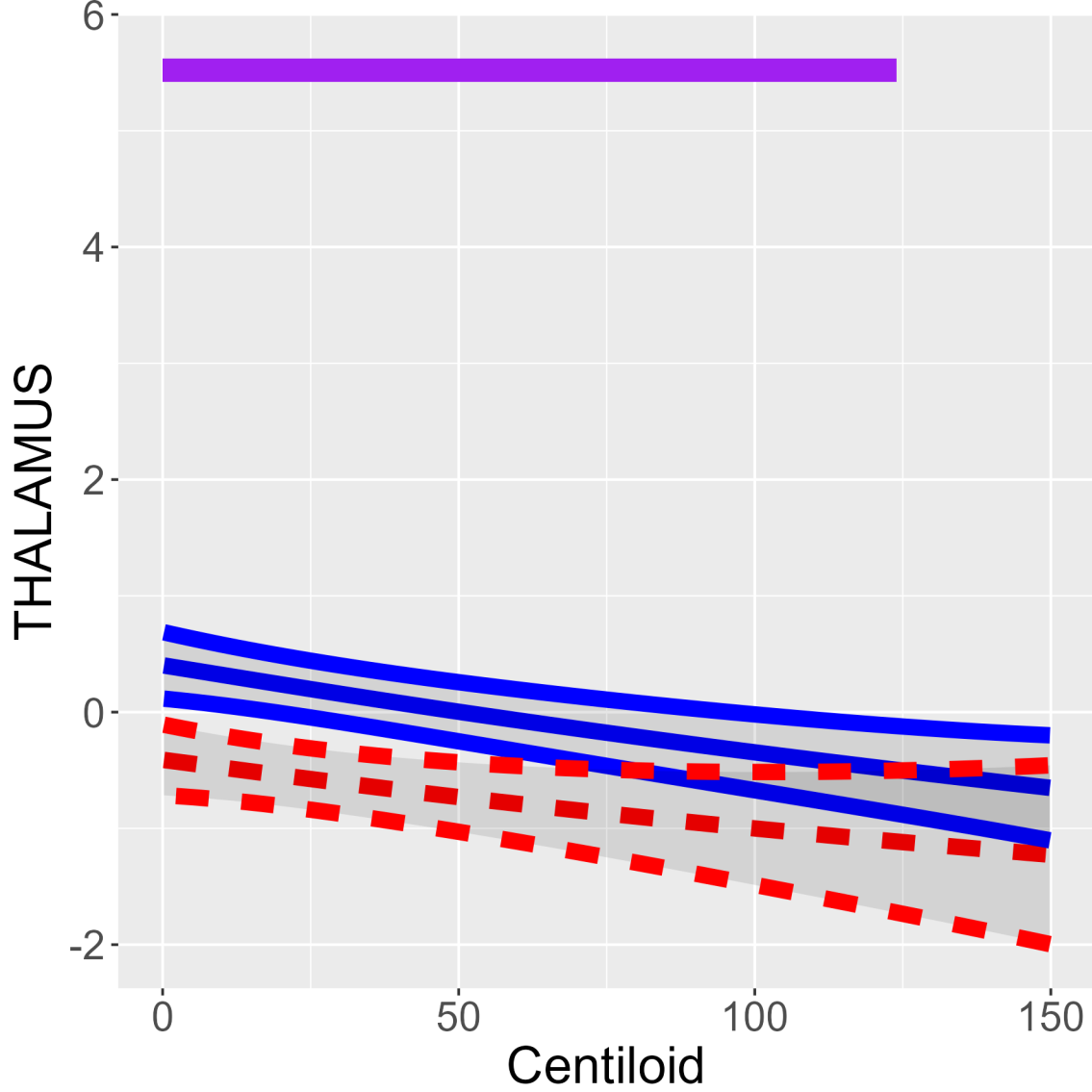

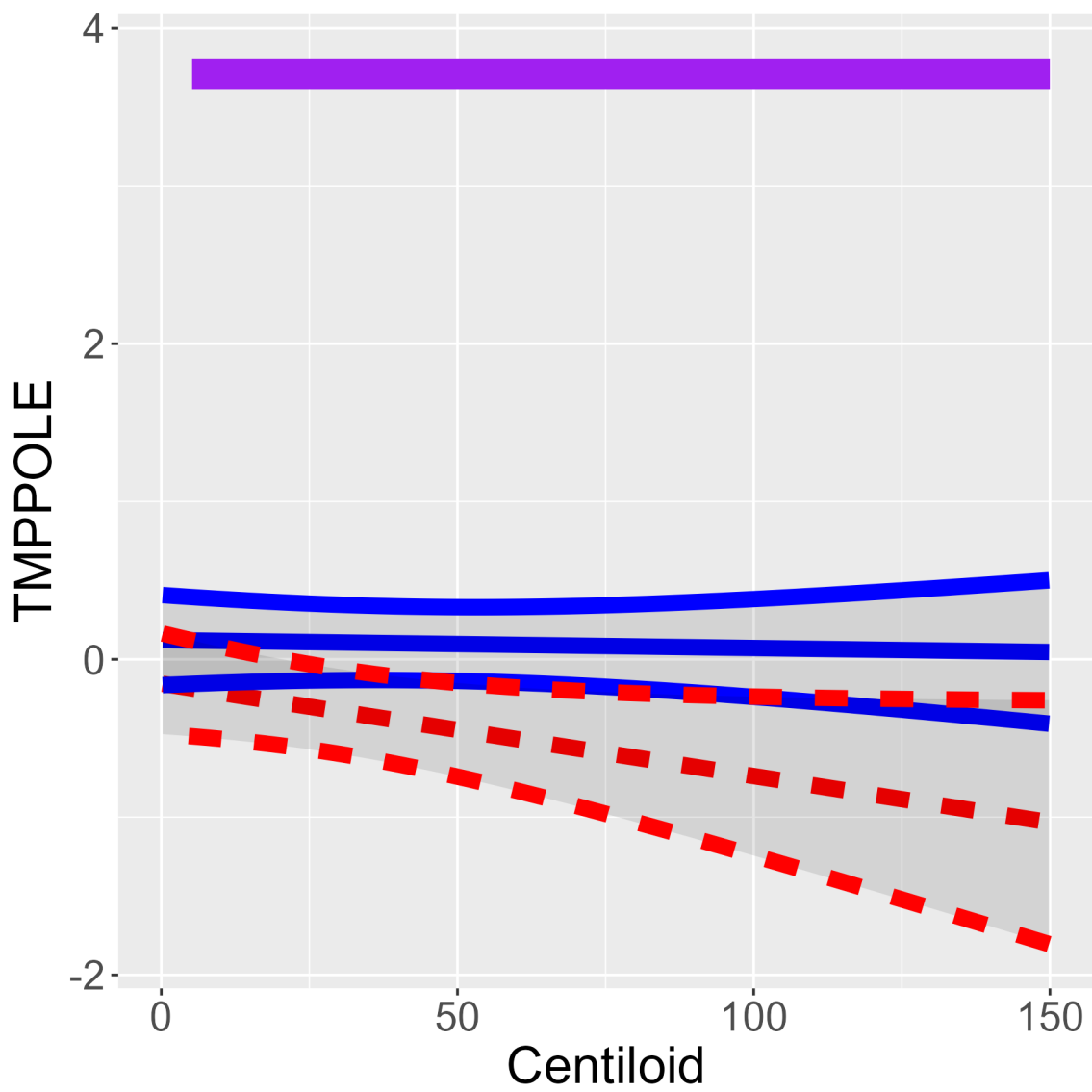

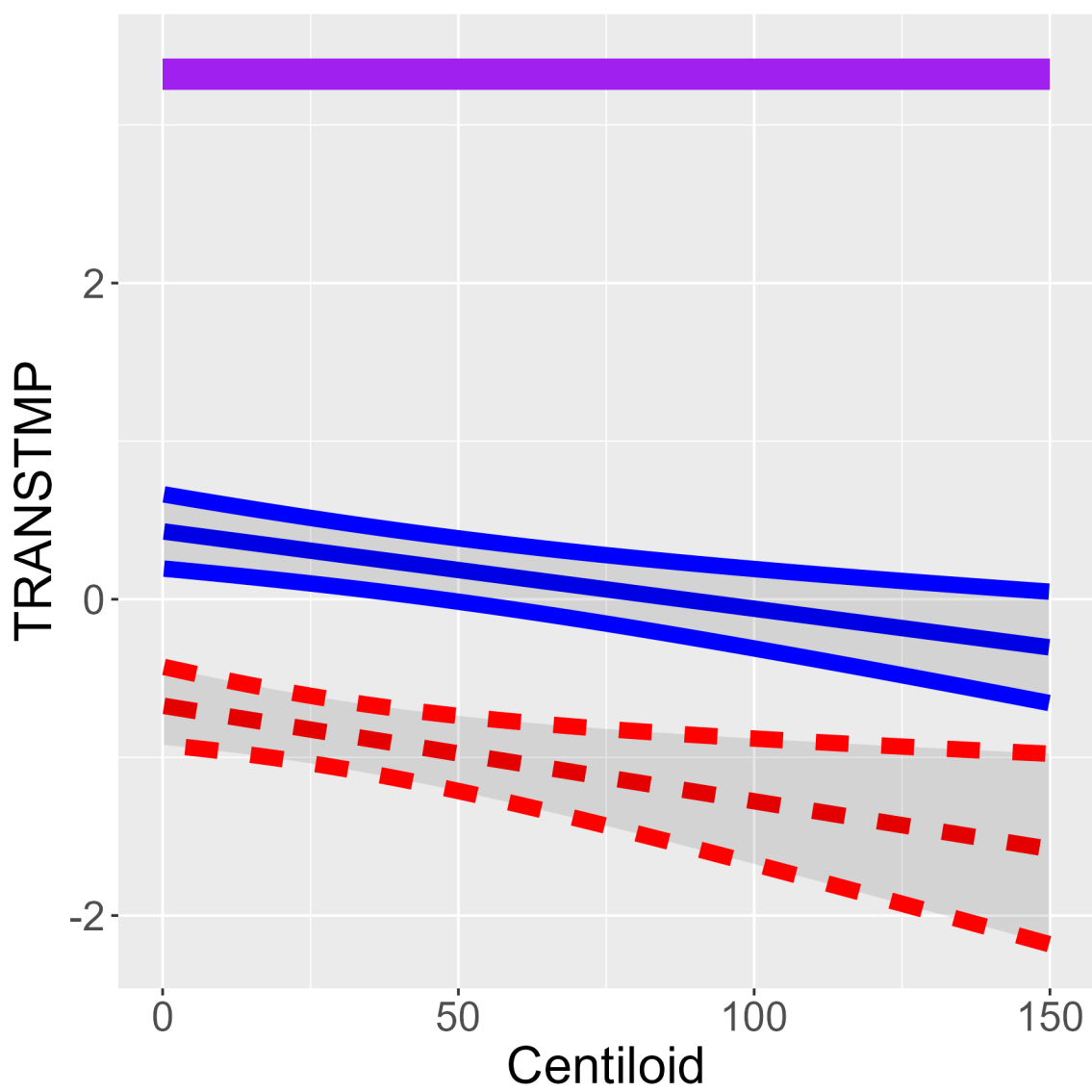

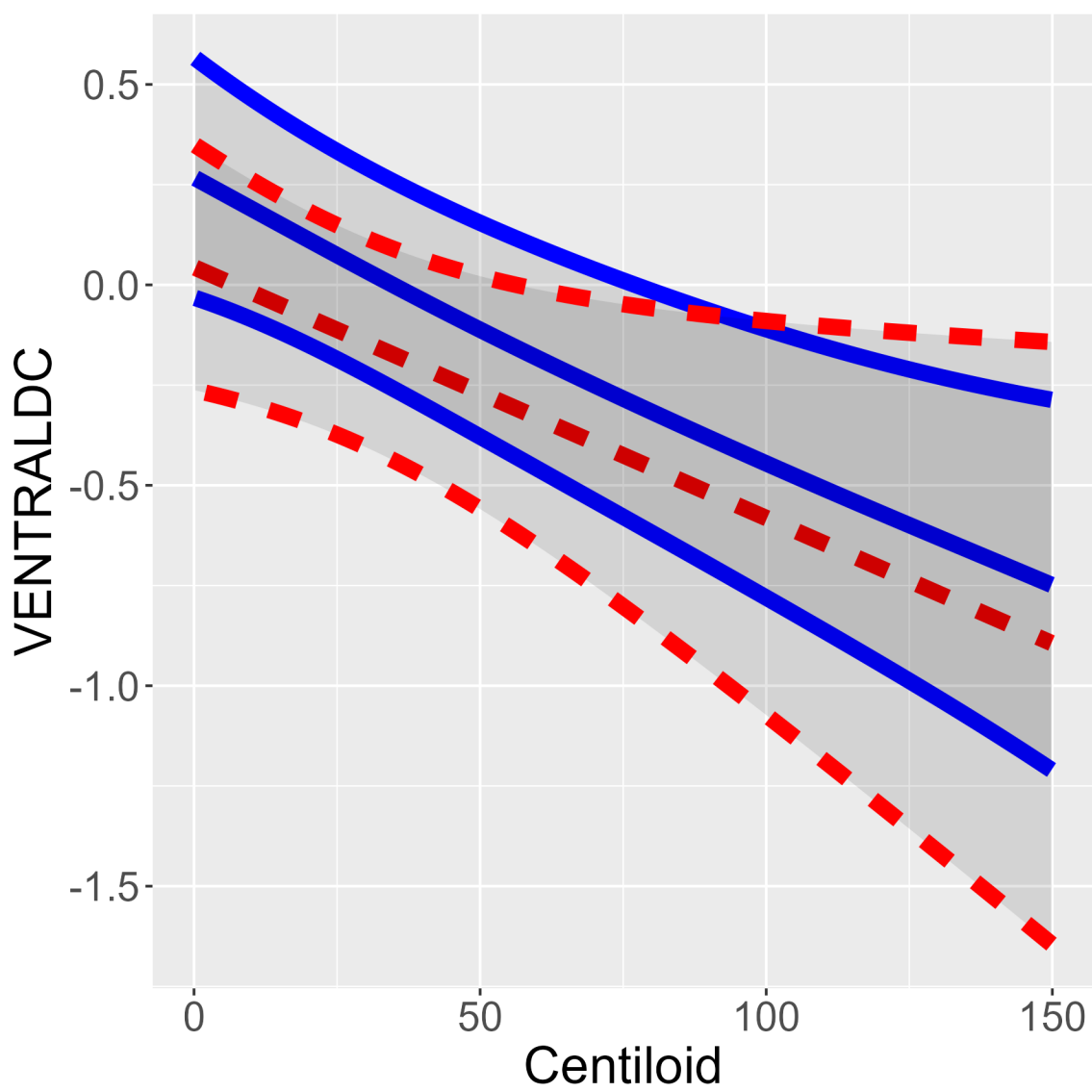

Supplement: Supplementary file 8 — Supporting information [file ALZ-22-e71103-s017.pdf]
