# Supplementary material for: Brain volume trajectories in Down syndrome and autosomal dominant Alzheimer's disease
Source: Alzheimers Dement. 2026 Jan 18;22(1):e71103. doi: 10.1002/alz.71103 (PMC12812856; doi:10.1002/alz.71103)
Supplement: Supplementary file 9 — Supporting information [file ALZ-22-e71103-s011.pdf]

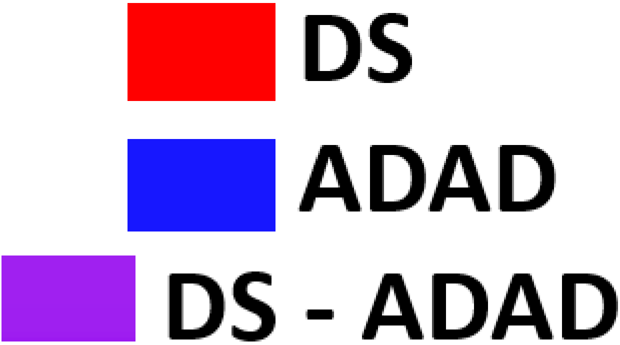
DS
ADAD
DS - ADAD

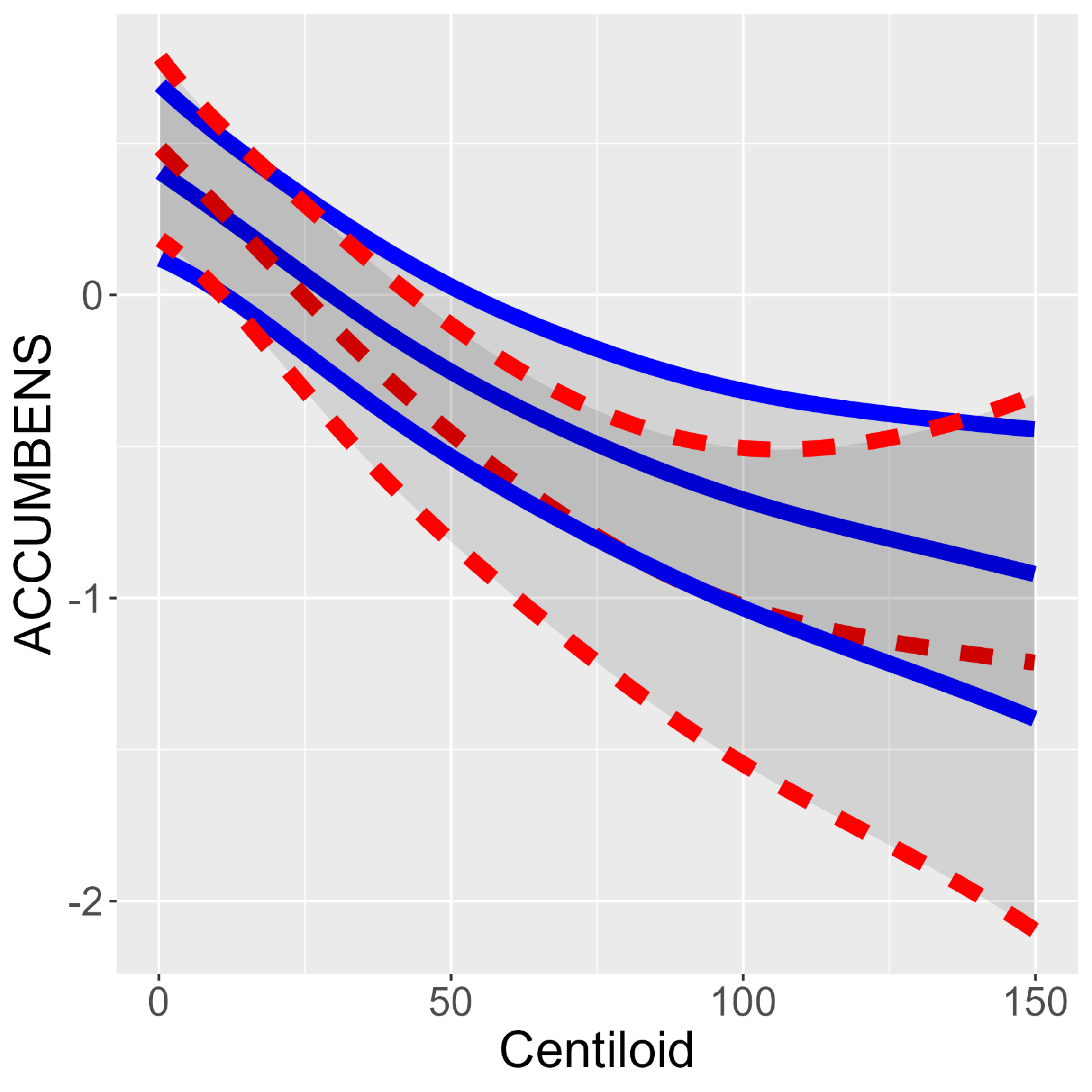

0
-1
-2
ACCUMBENS
0
50
100
150
Centiloid

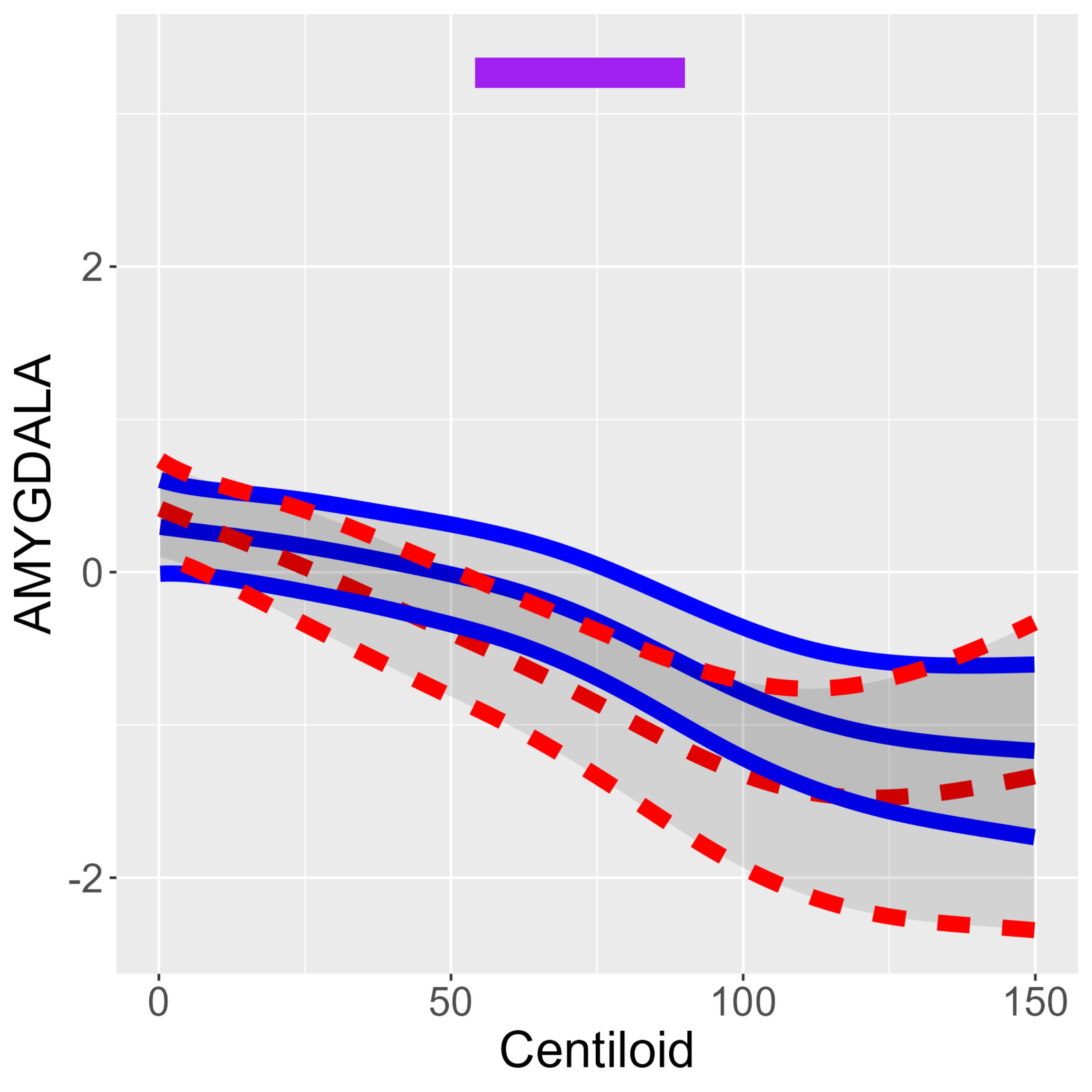

AMYGDALA
2
0
-2
0
50
100
150
Centiloid

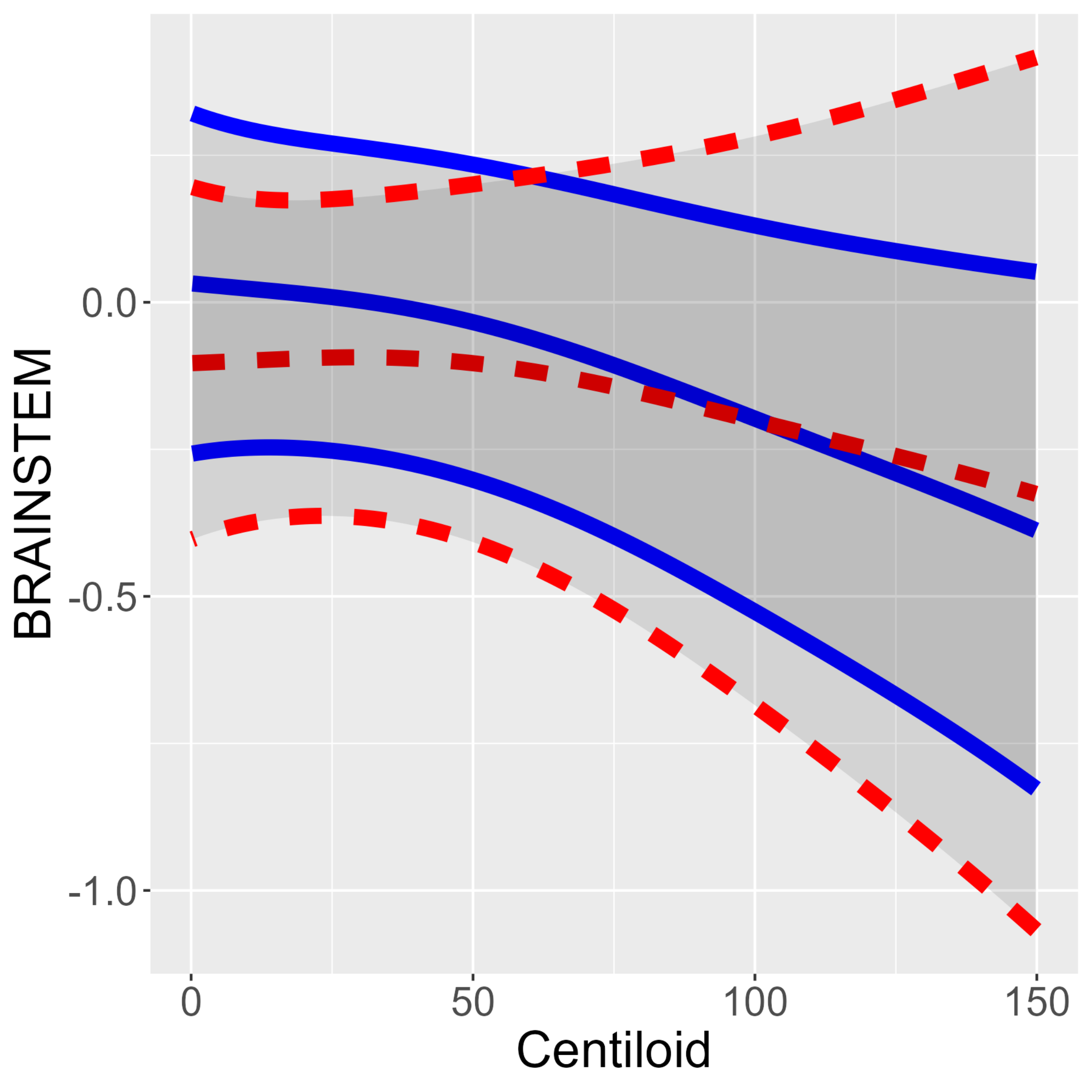

BRAINSTEM
0.0
-0.5
-1.0
0
50
100
150
Centiloid

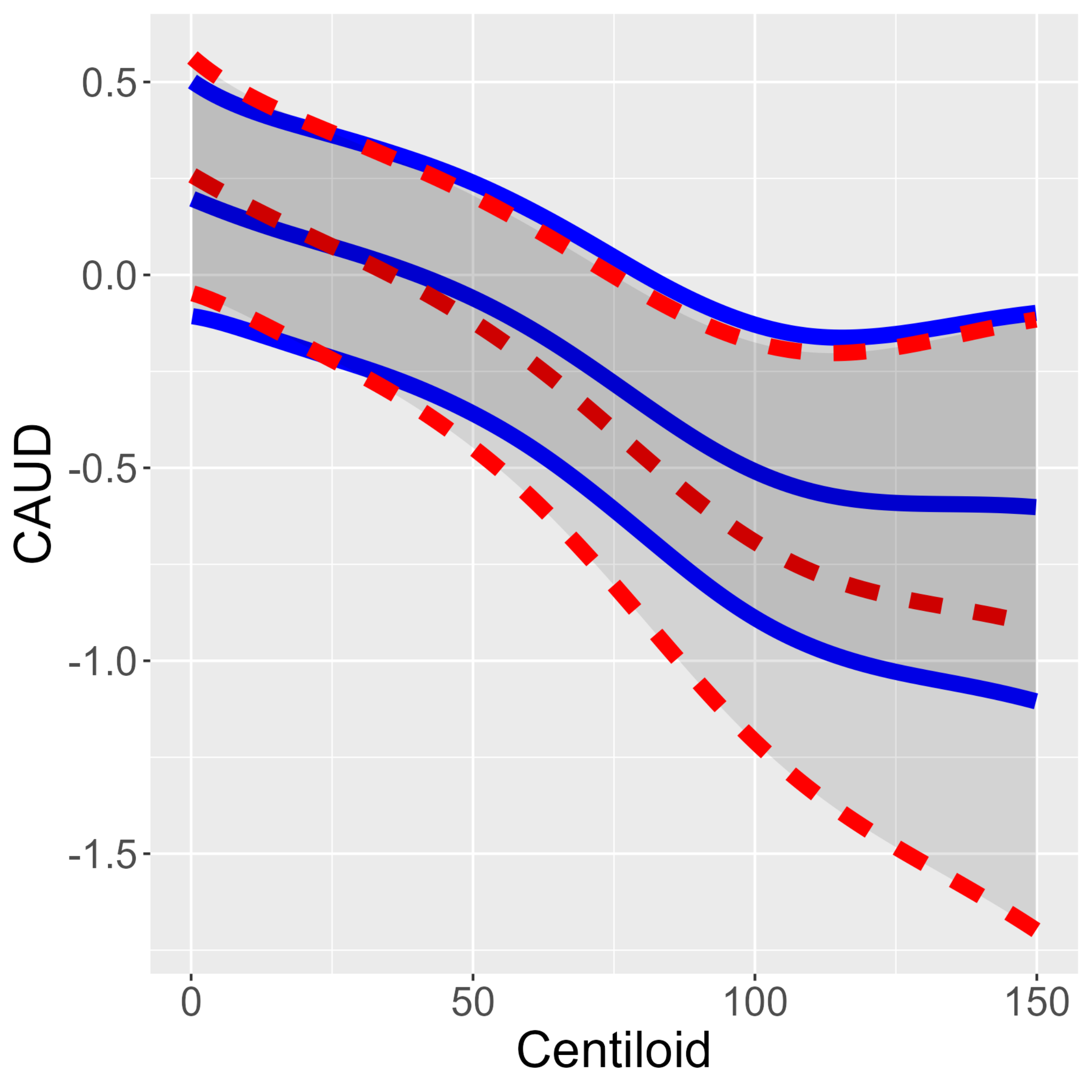

0.5
0.0
-0.5
-1.0
-1.5
CAUD
0
50
100
150
Centiloid

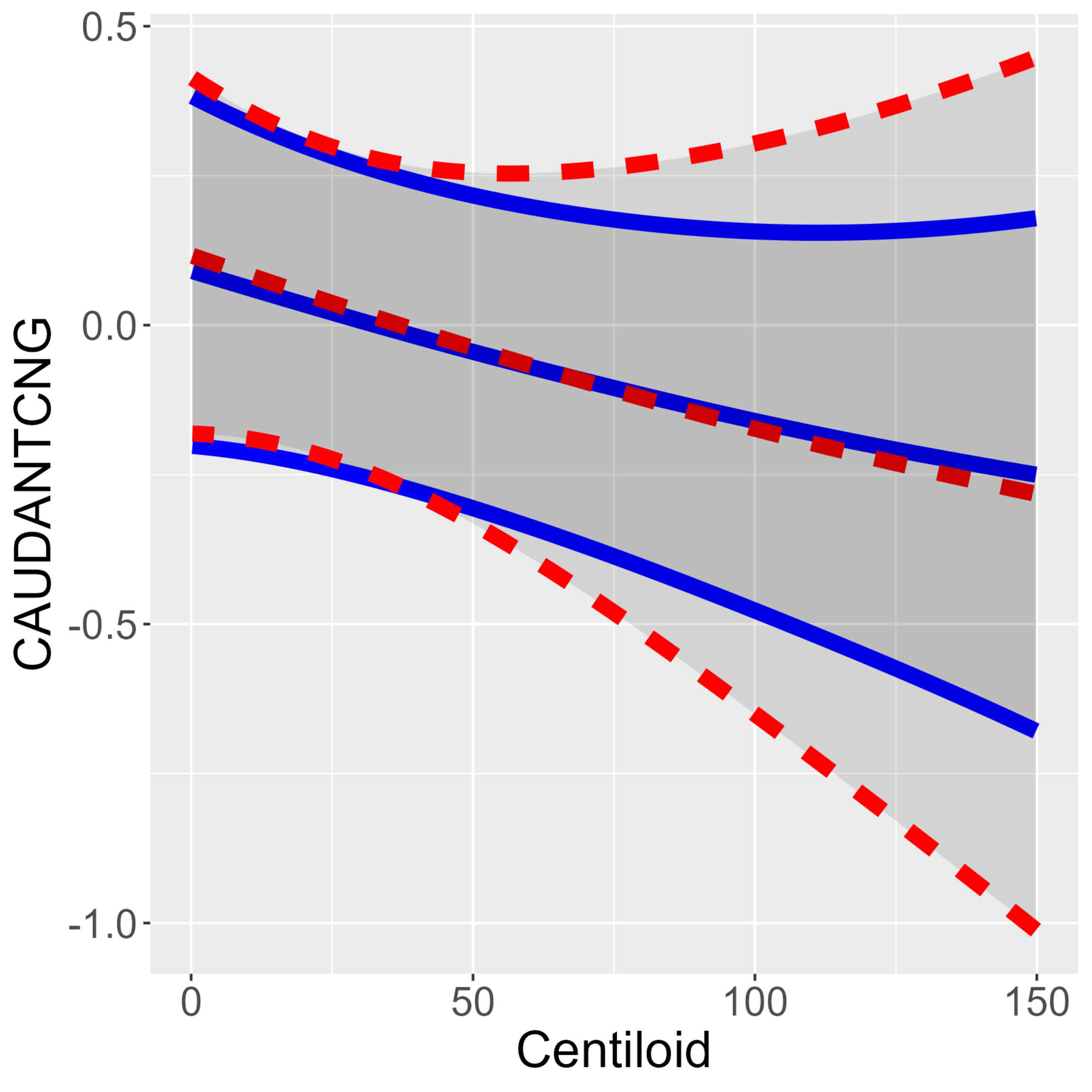

0.5
0.0
-0.5
-1.0
CAUDANTCNG
0
50
100
150
Centiloid

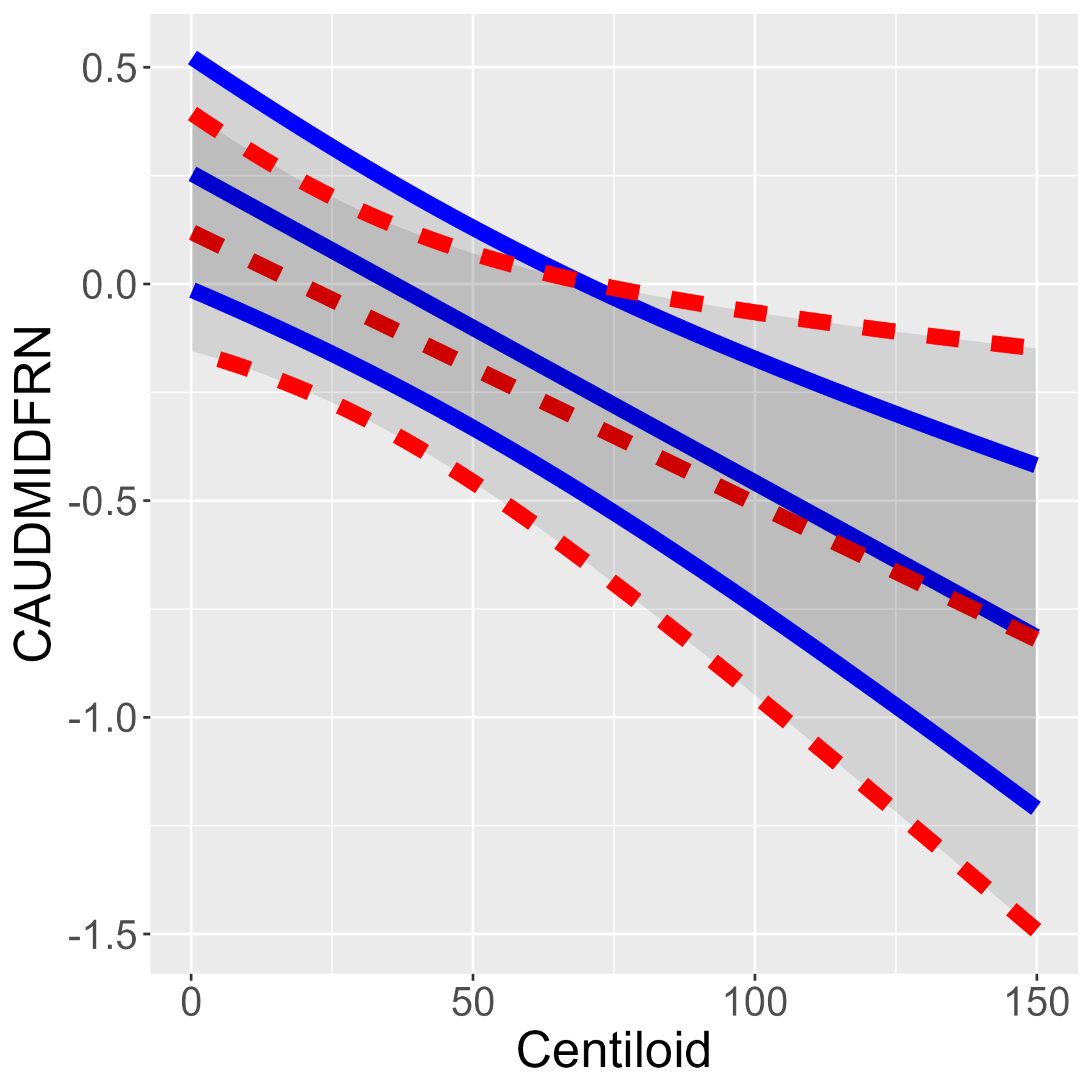

0.5
0.0
-0.5
-1.0
-1.5
CAUDMIDFRN
0
50
100
150
Centiloid

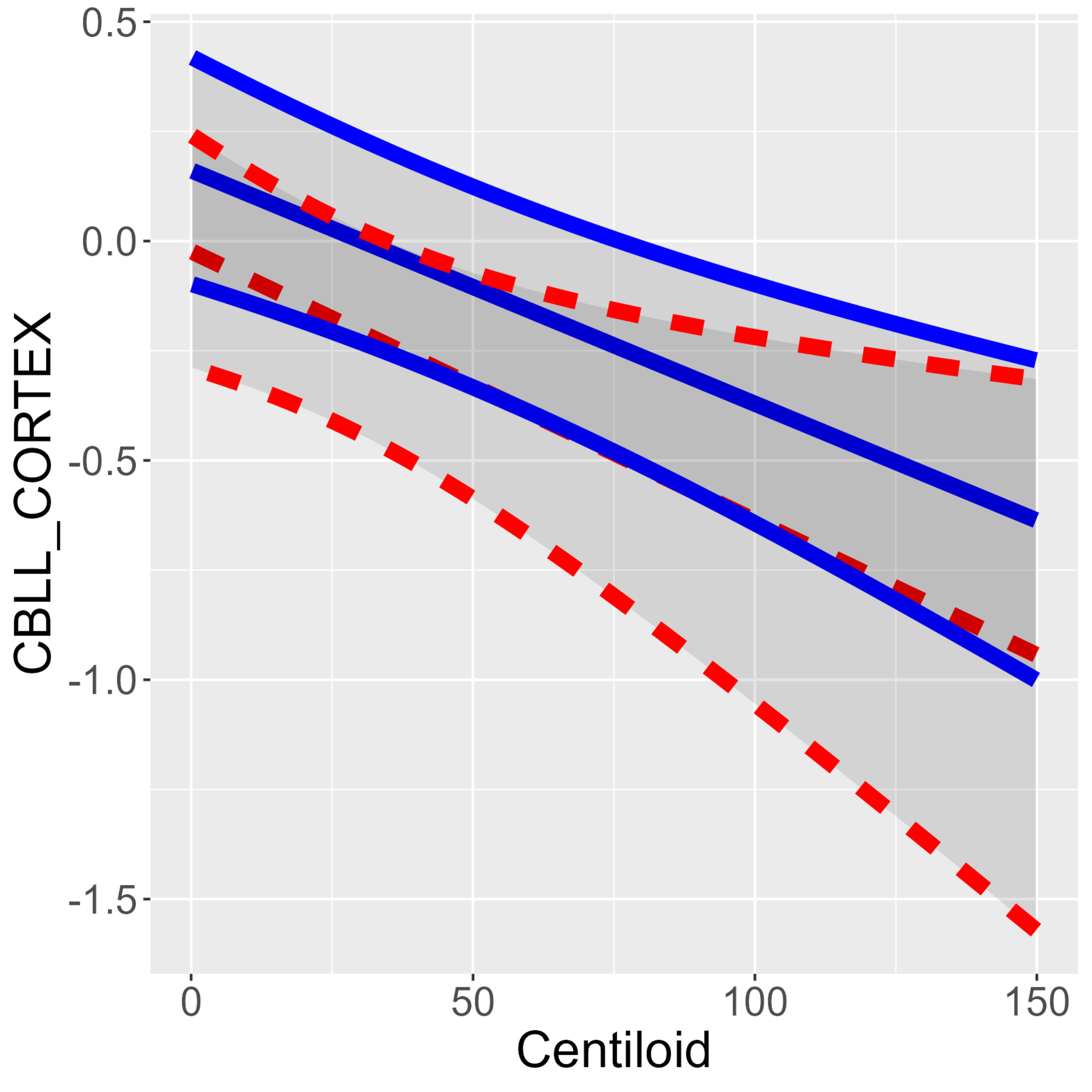

0.5
0.0
-0.5
-1.0
-1.5
CBLL_CORTEX
0
50
100
150
Centiloid

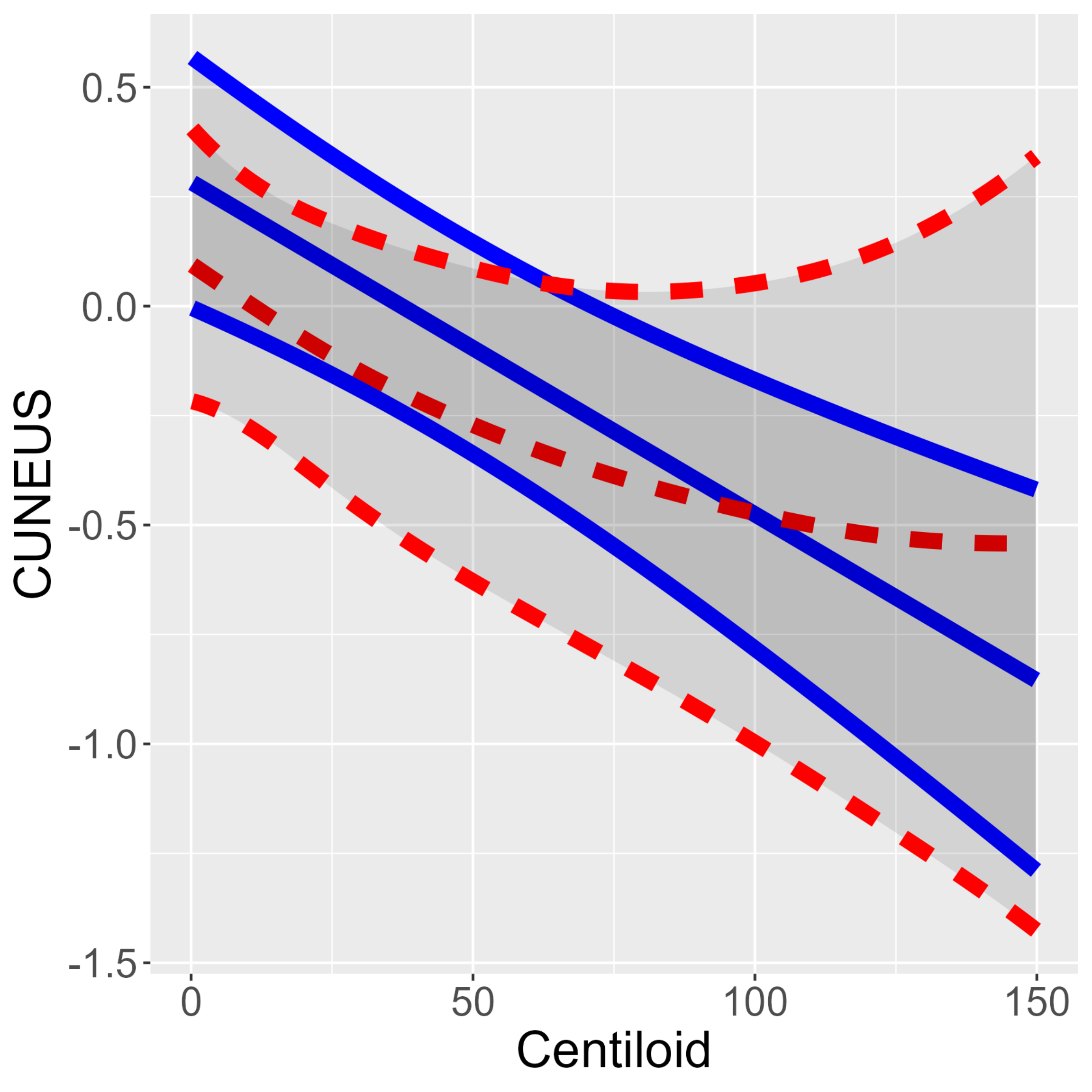

0.5
0.0
-0.5
-1.0
-1.5
CUNEUS
0
50
100
150
Centiloid

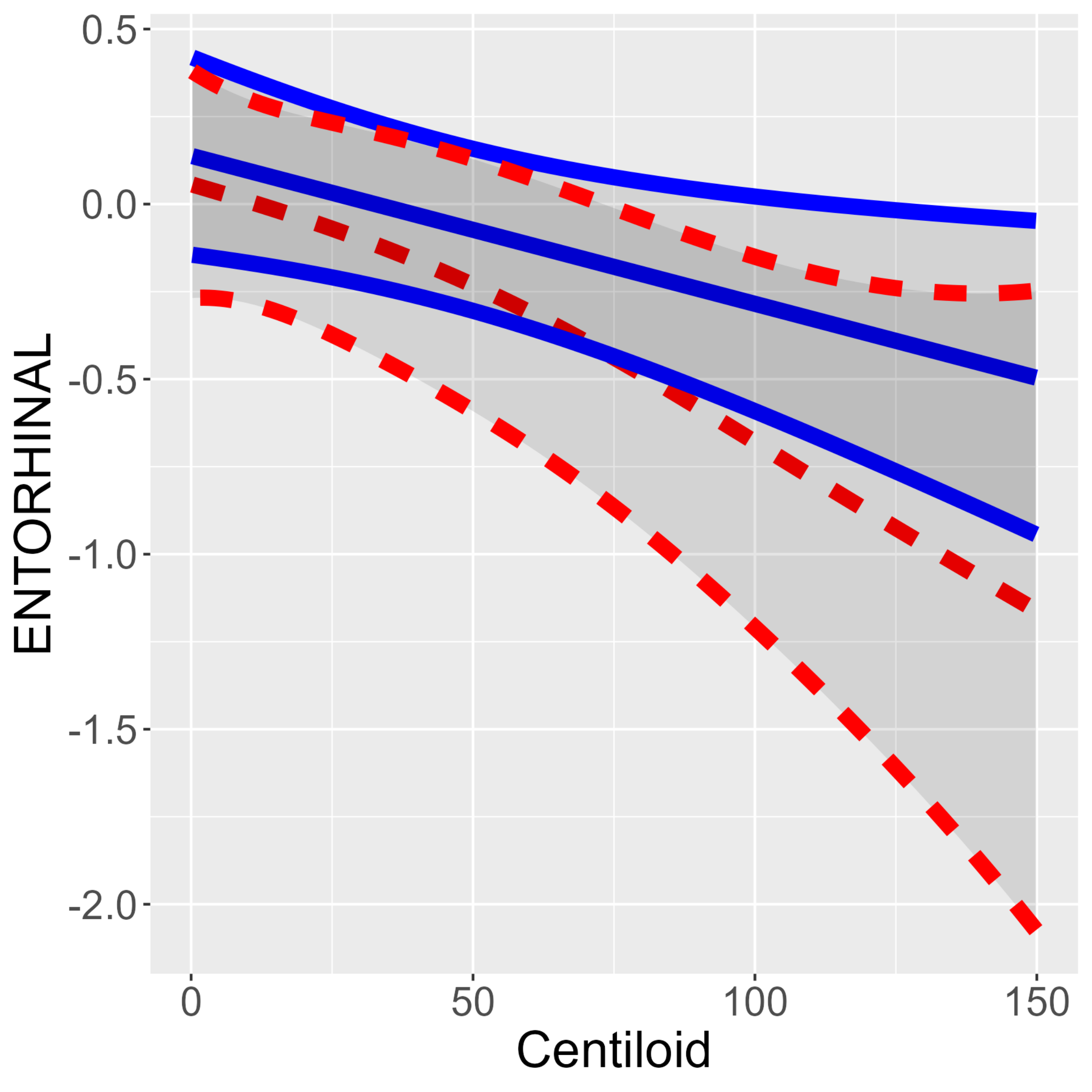

ENTORHINAL
Centiloid
0.5
0.0
-0.5
-1.0
-1.5
-2.0
0
50
100
150

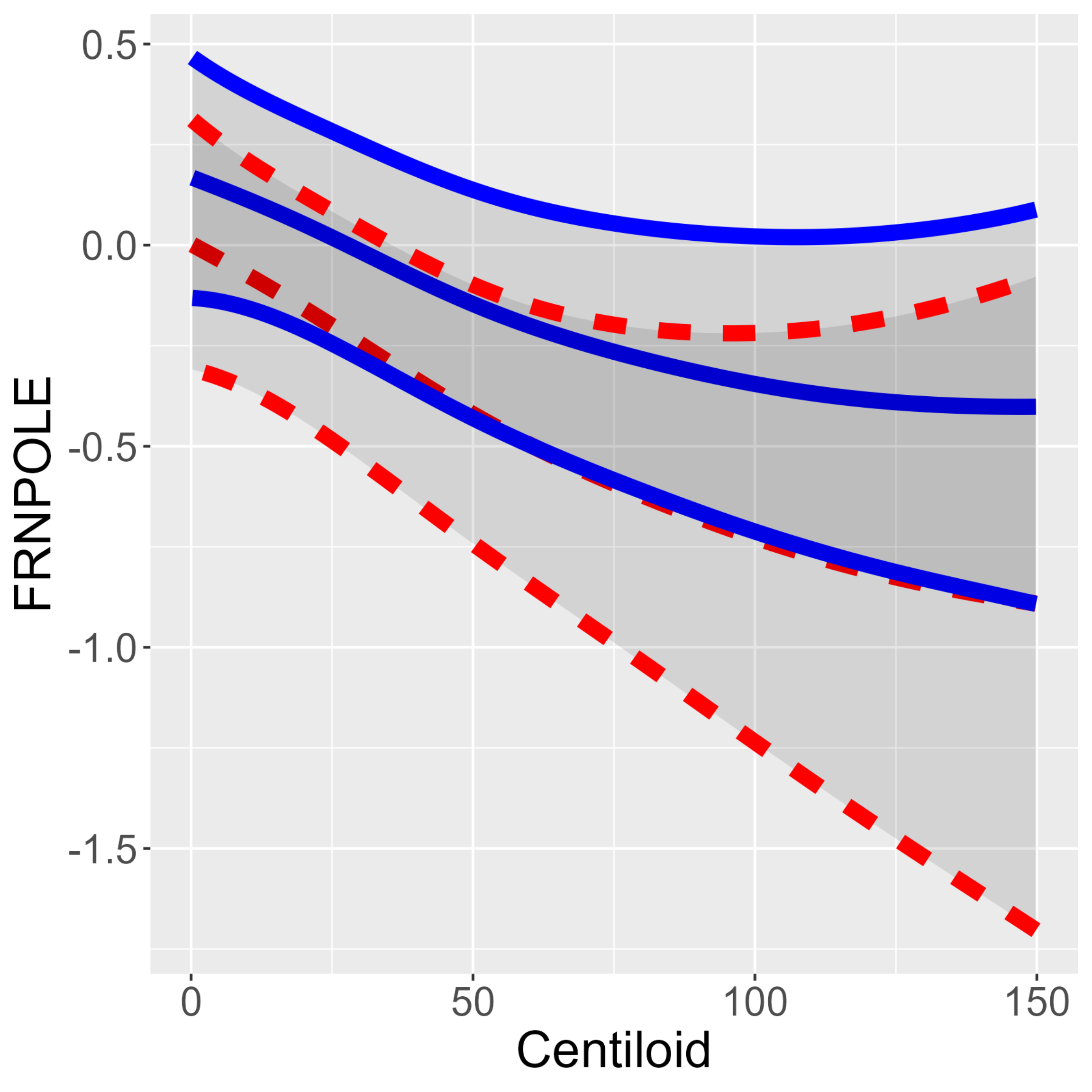

0.5
0.0
-0.5
-1.0
-1.5
FRNPOLE
0
50
100
150
Centiloid

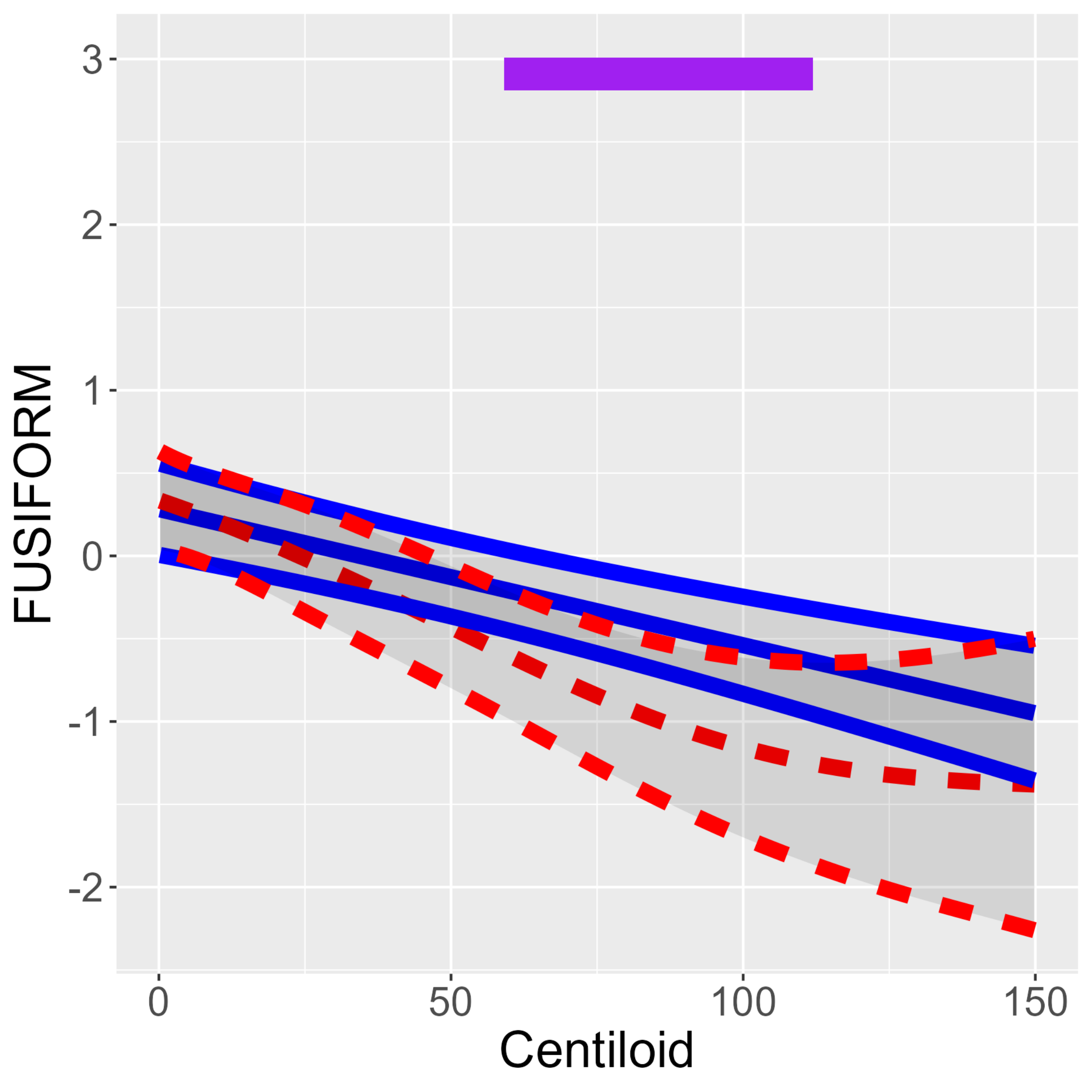

3
2
1
0
-1
-2
FUSIFORM
0
50
100
150
Centiloid

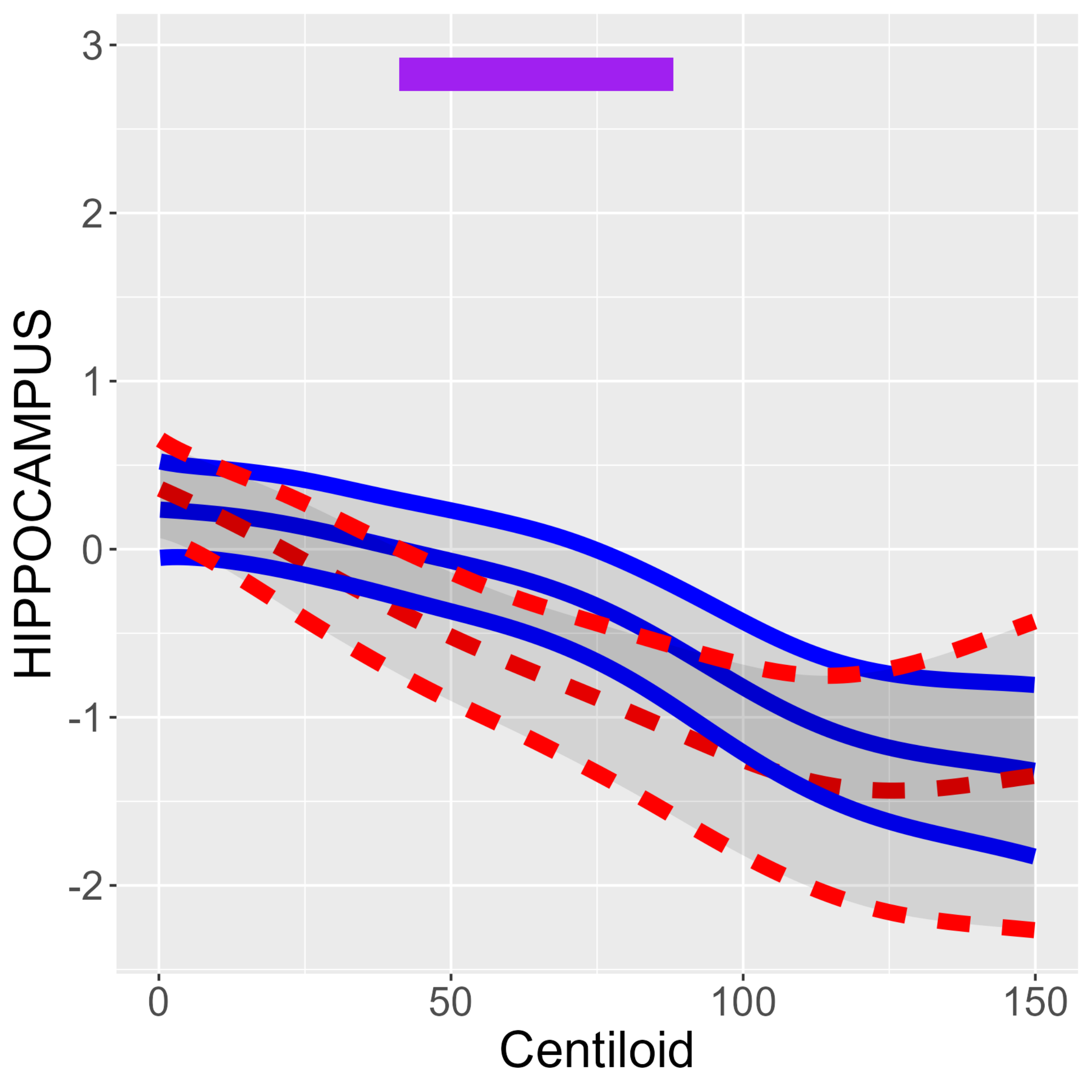

3
2
1
0
-1
-2
HIPPOCAMPUS
0
50
100
150
Centiloid

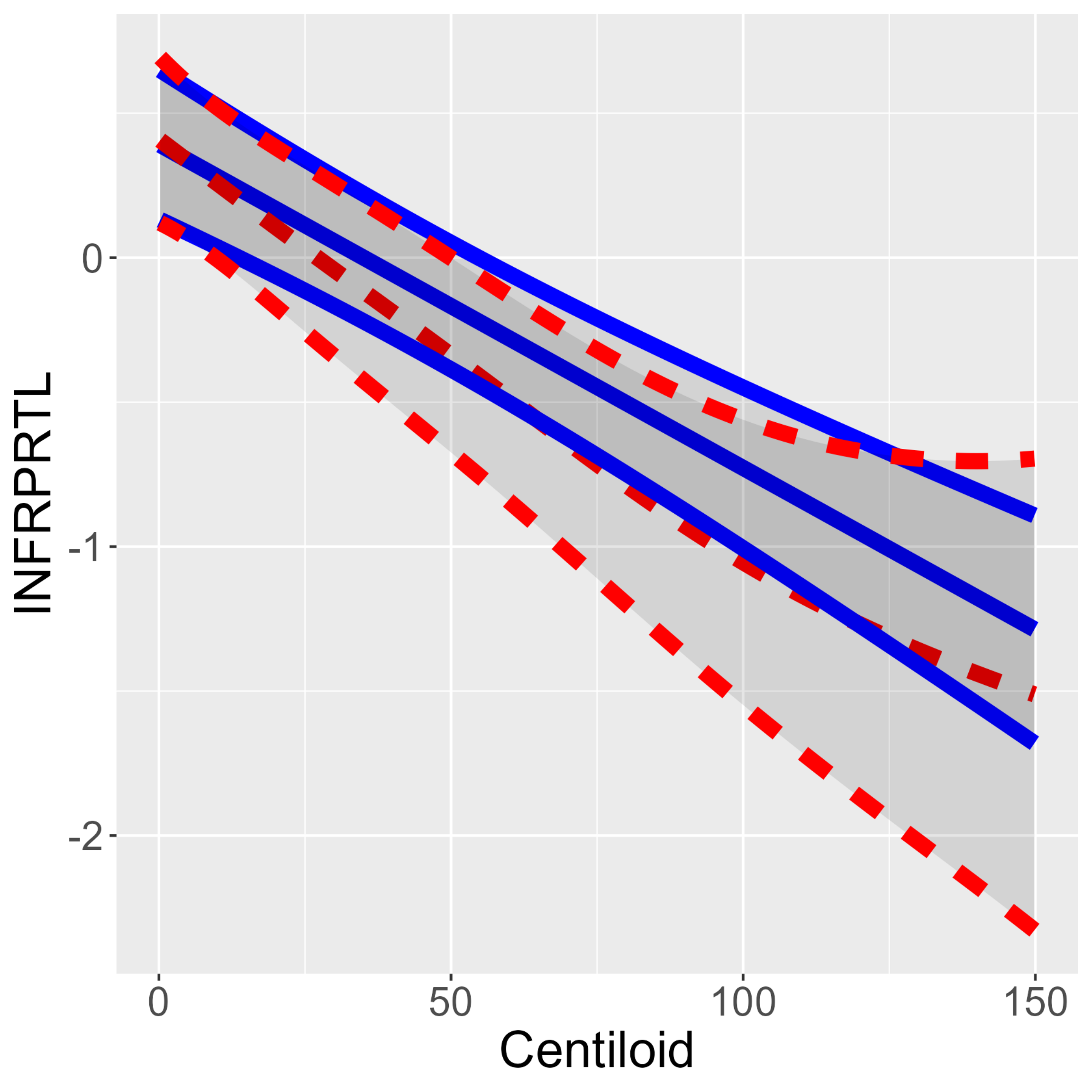

INFRPRTL
0
-1
-2
0
50
100
150
Centiloid

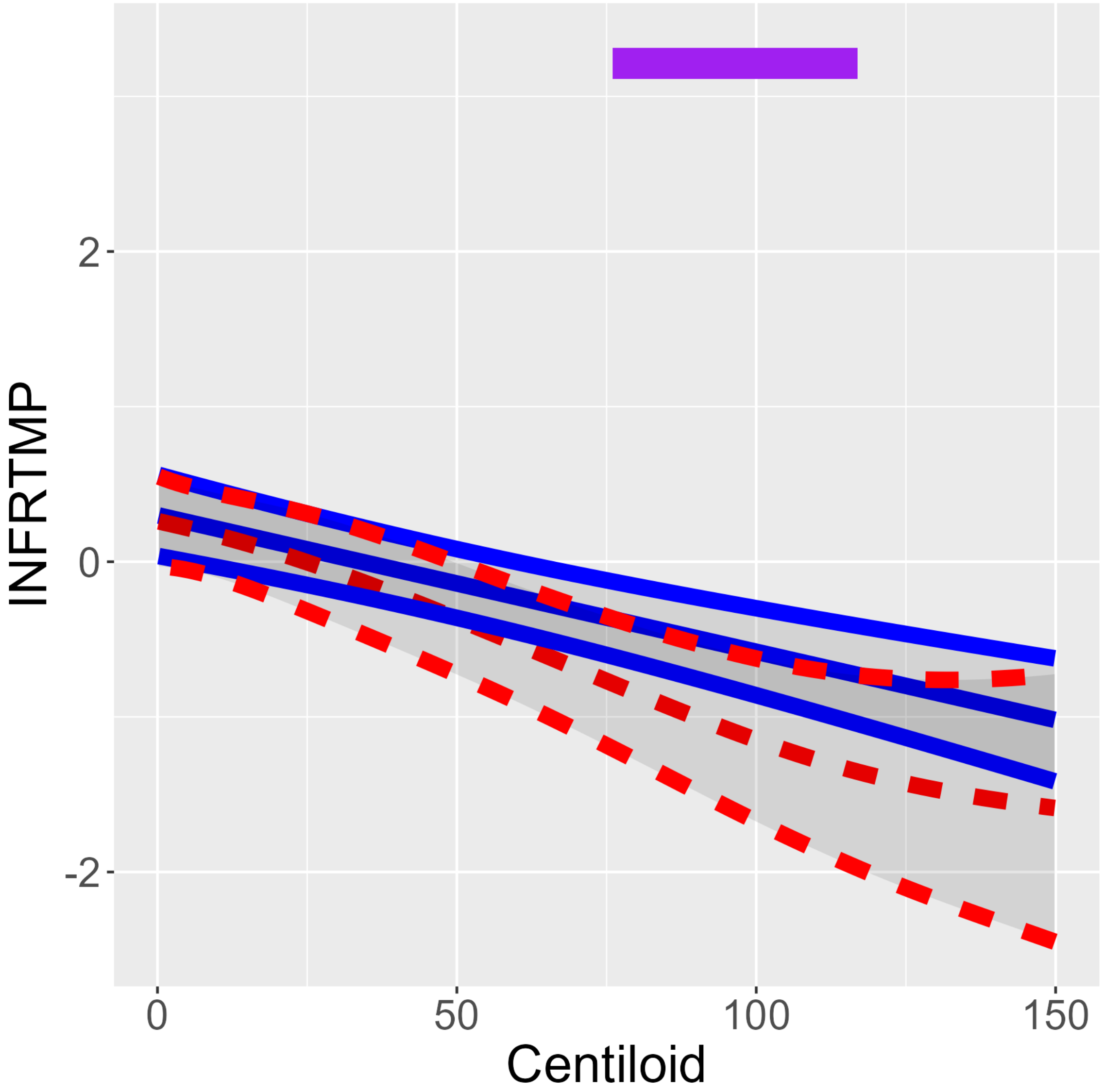
2
0
-2
INFRTMP
0
50
100
150
Centiloid

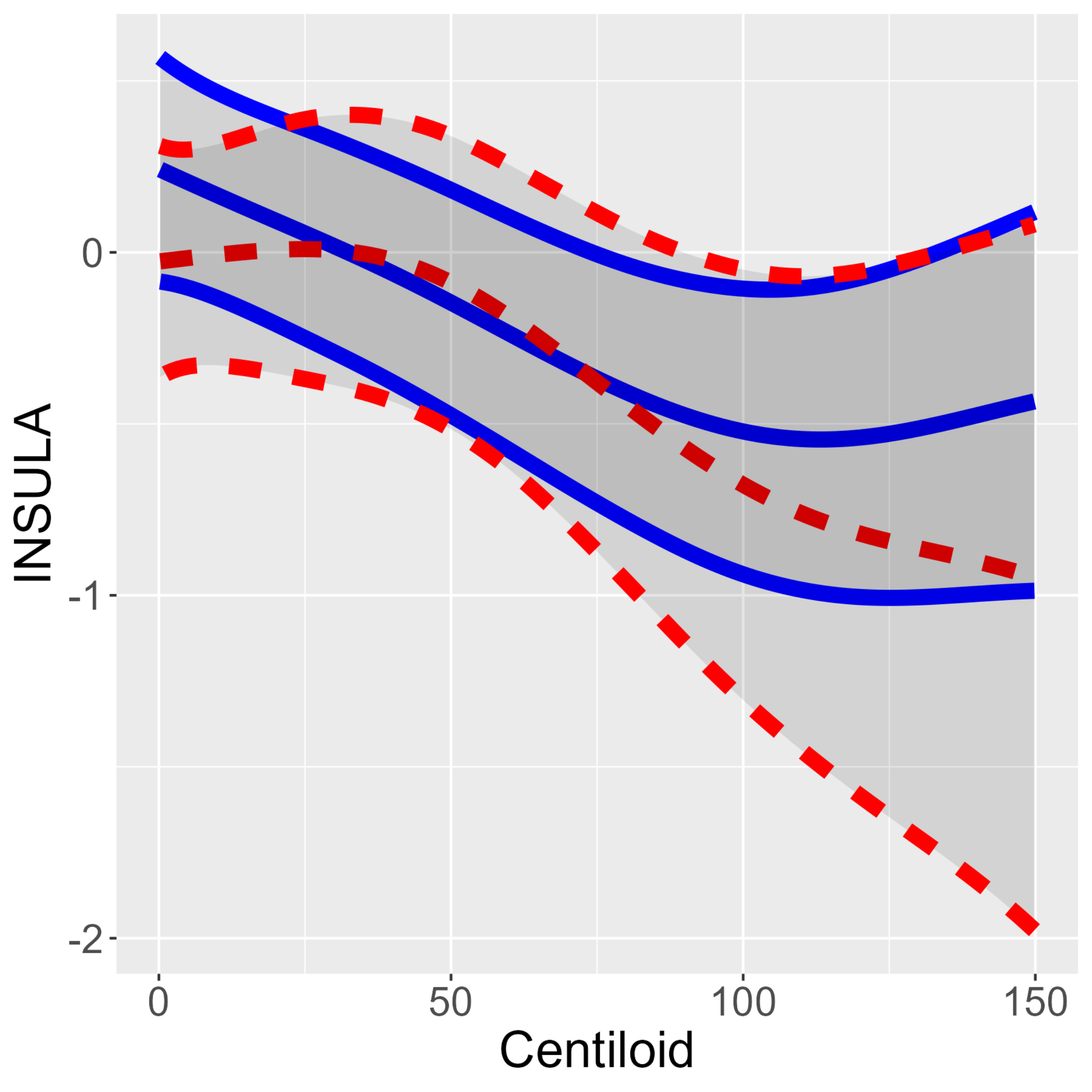

INSULA
0
-1
-2
0
50
100
150
Centiloid

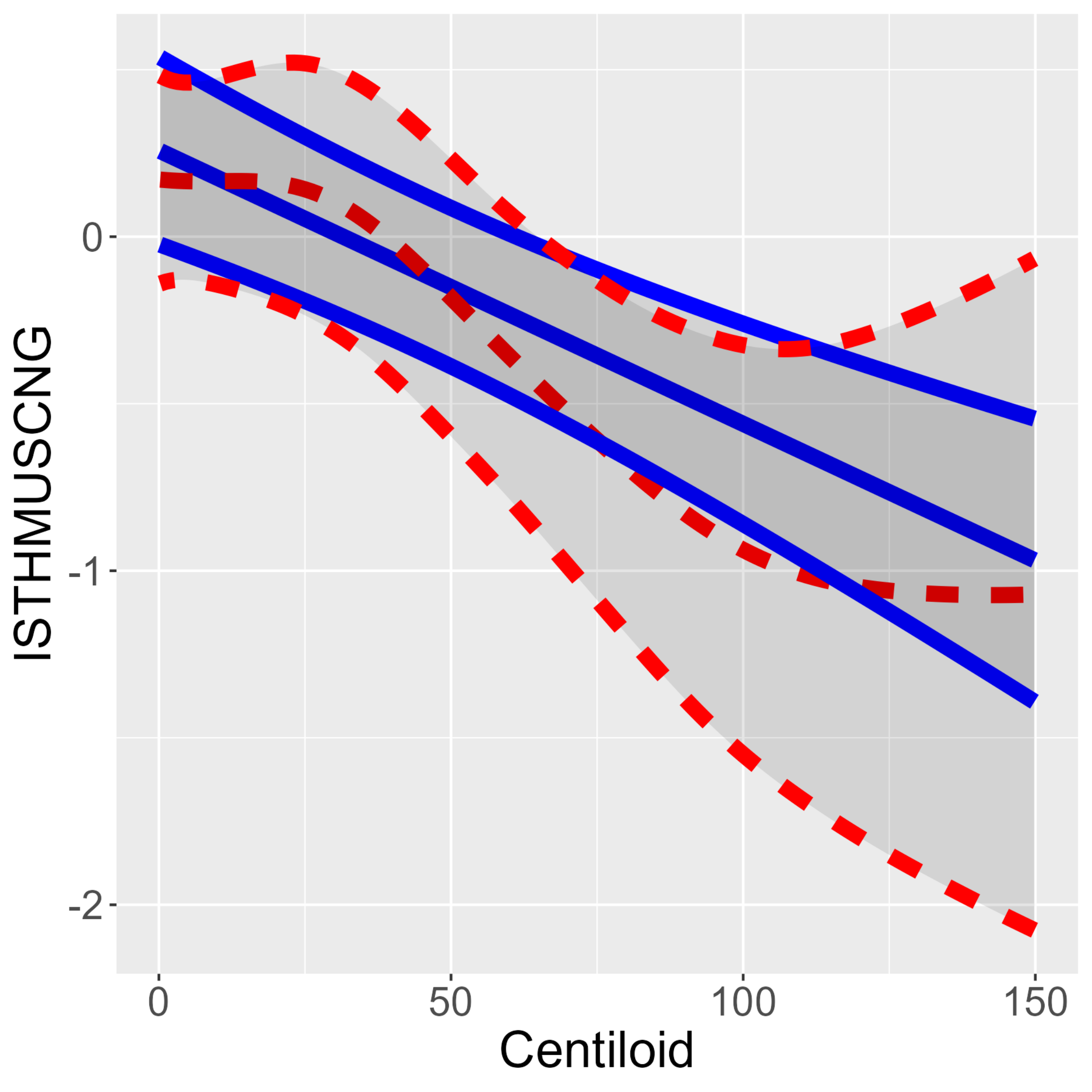

ISTHMUSCNG
0
-1
-2
0
50
100
150
Centiloid

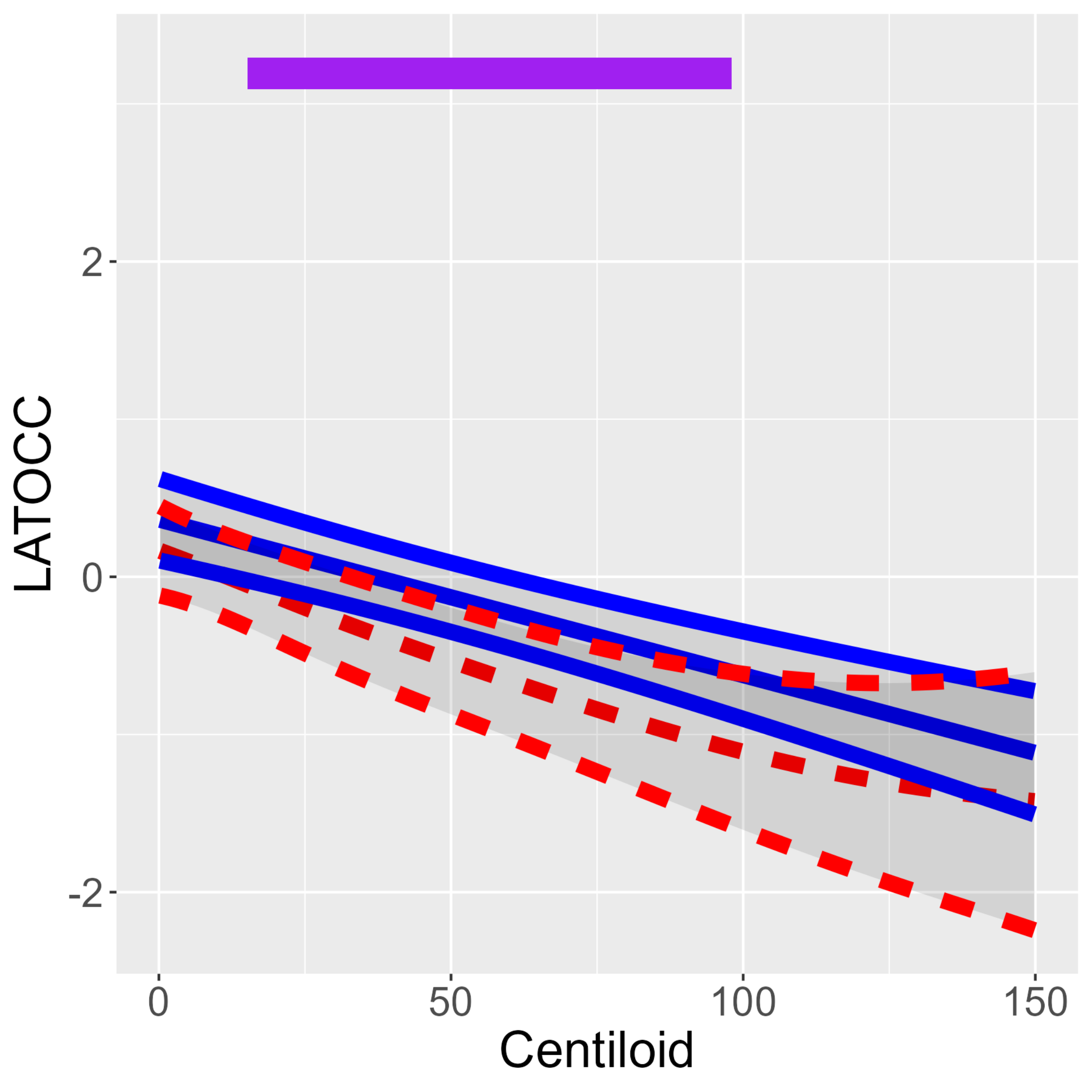

LATOCC
2
0
-2
0
50
100
150
Centiloid

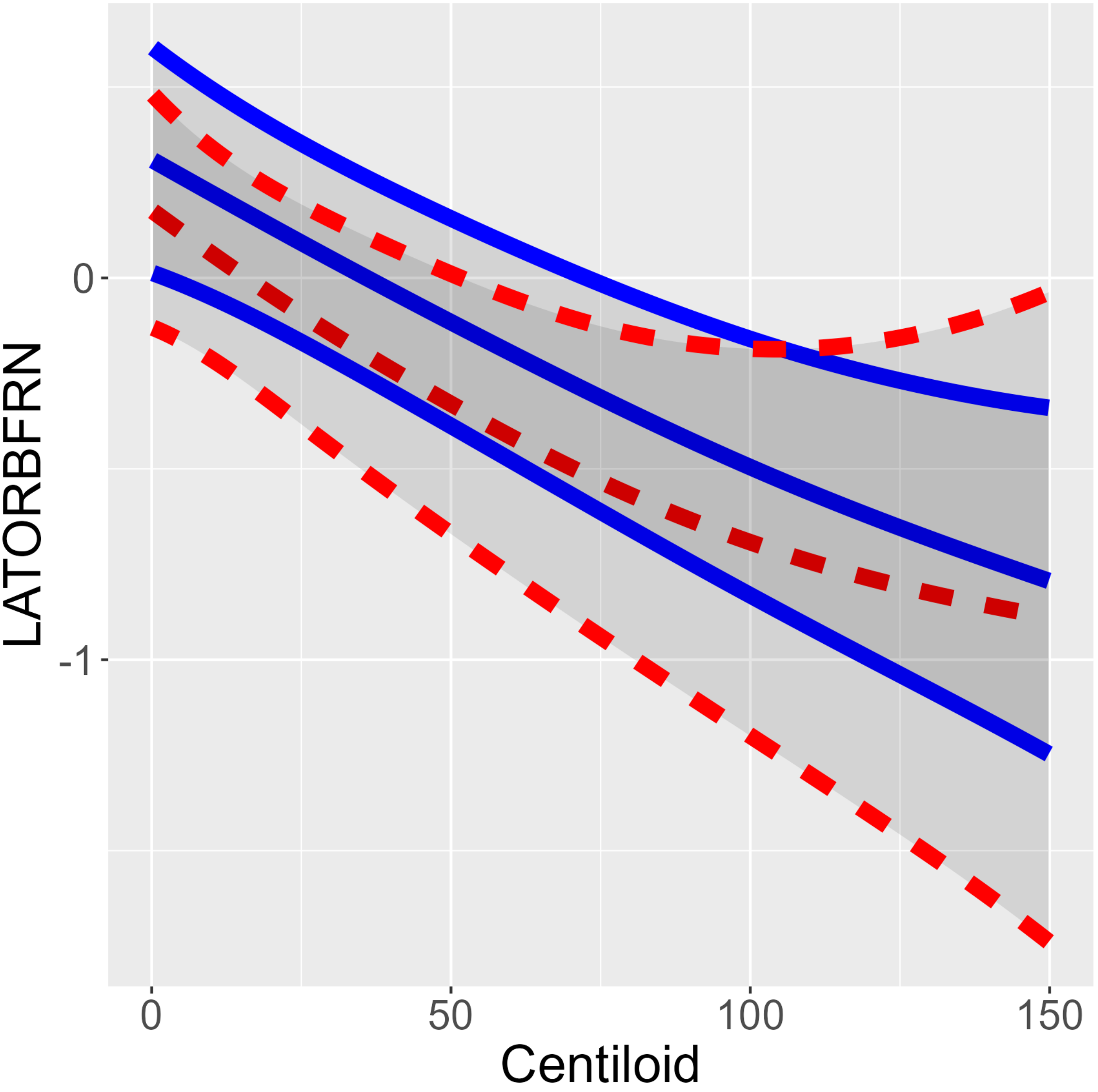

LATORBFRN
0
-1
0
50
100
150
Centiloid

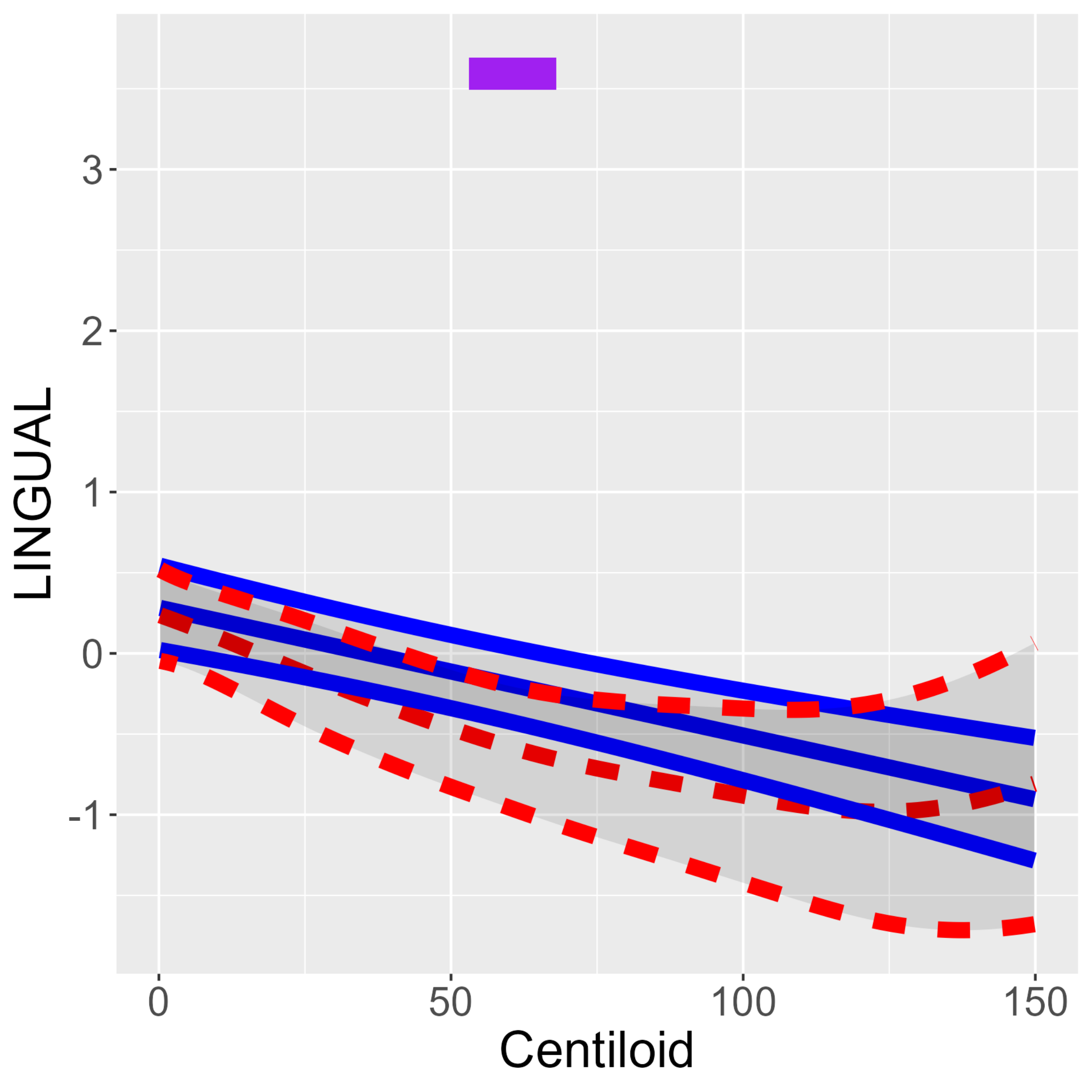

LINGUAL
3
2
1
0
-1
0
50
100
150
Centiloid

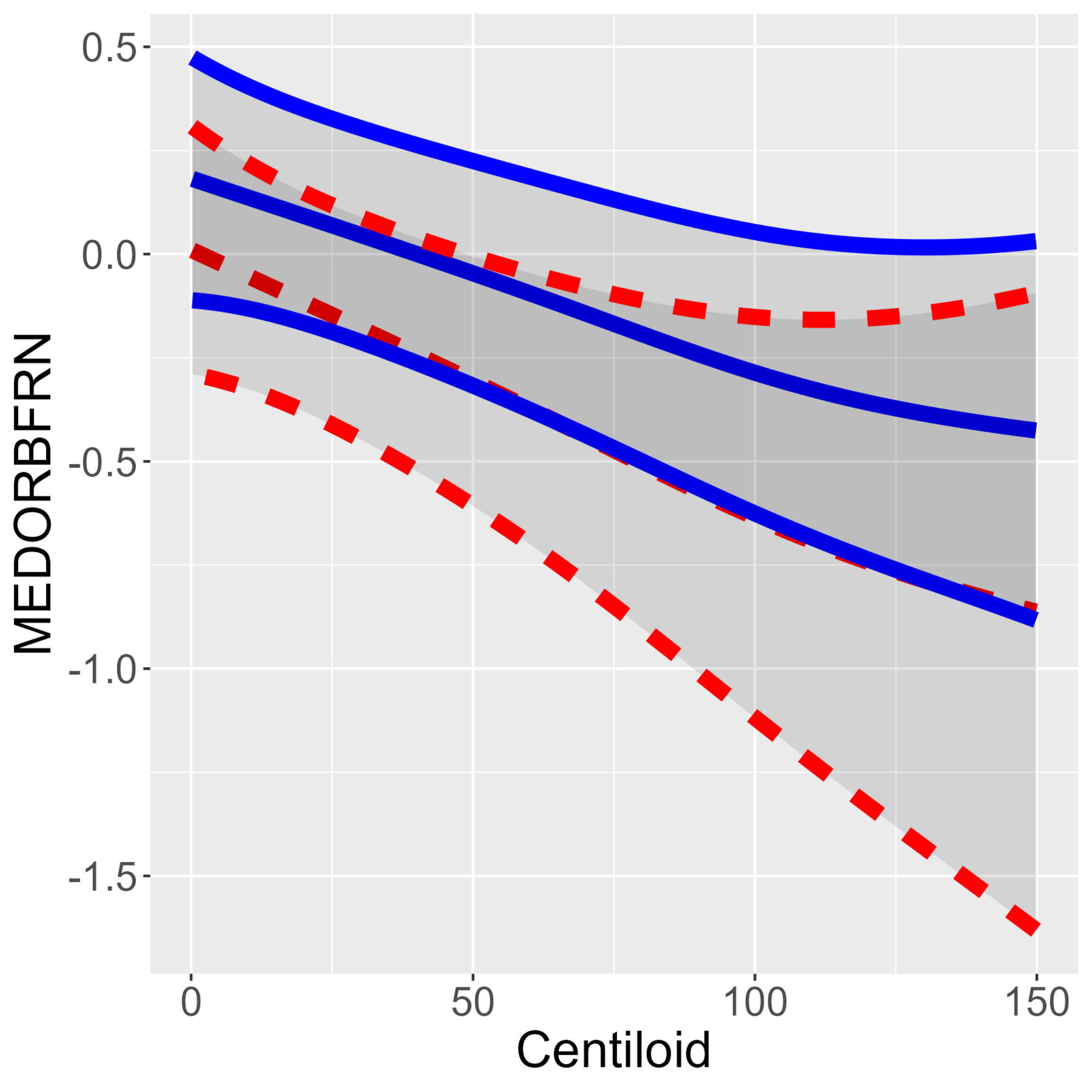

0.5
0.0
-0.5
-1.0
-1.5
MEDORBFRN
0
50
100
150
Centiloid

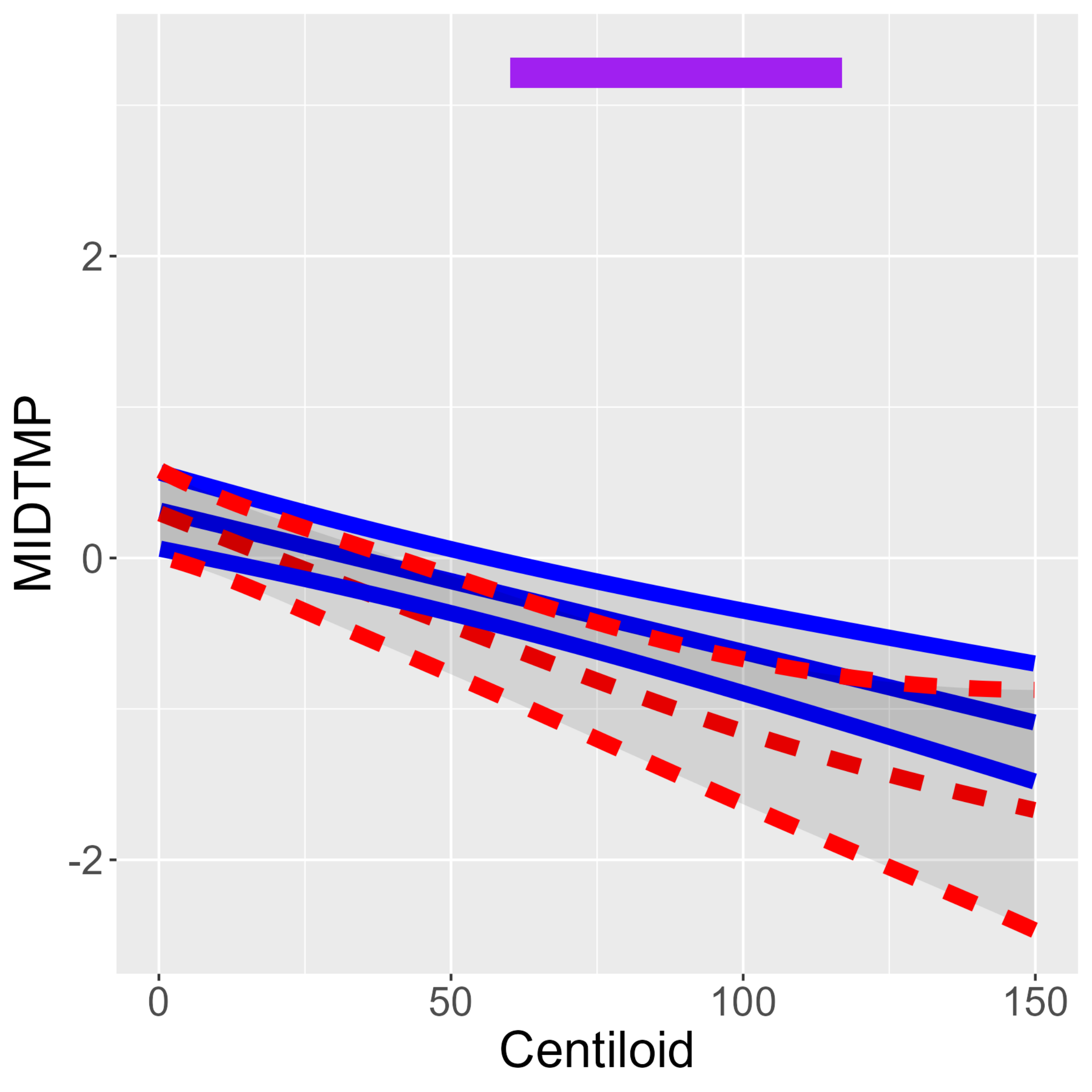

2
0
-2
MIDTMP
0
50
100
150
Centiloid

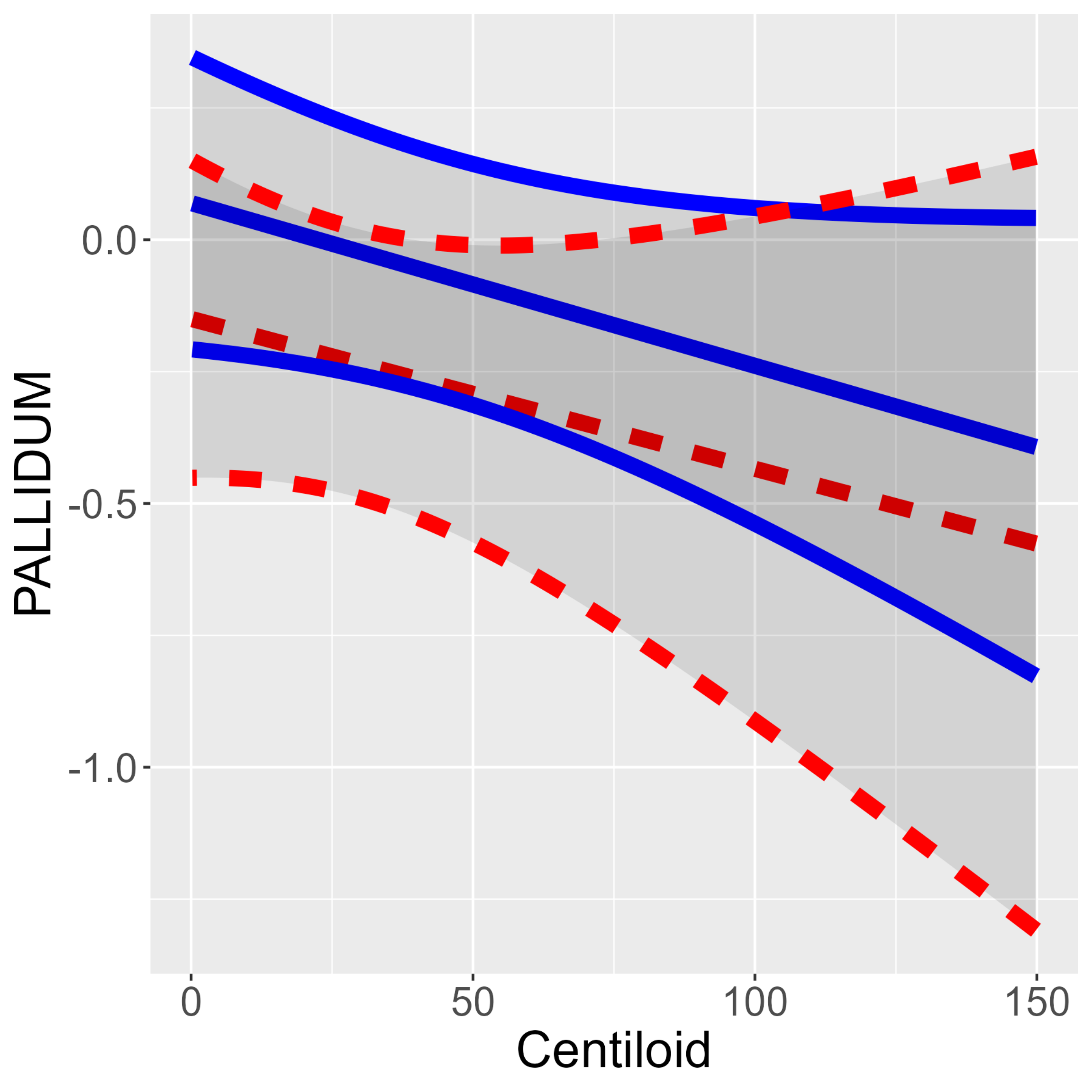

0.0
-0.5
-1.0
PALLIDUM
0
50
100
150
Centiloid

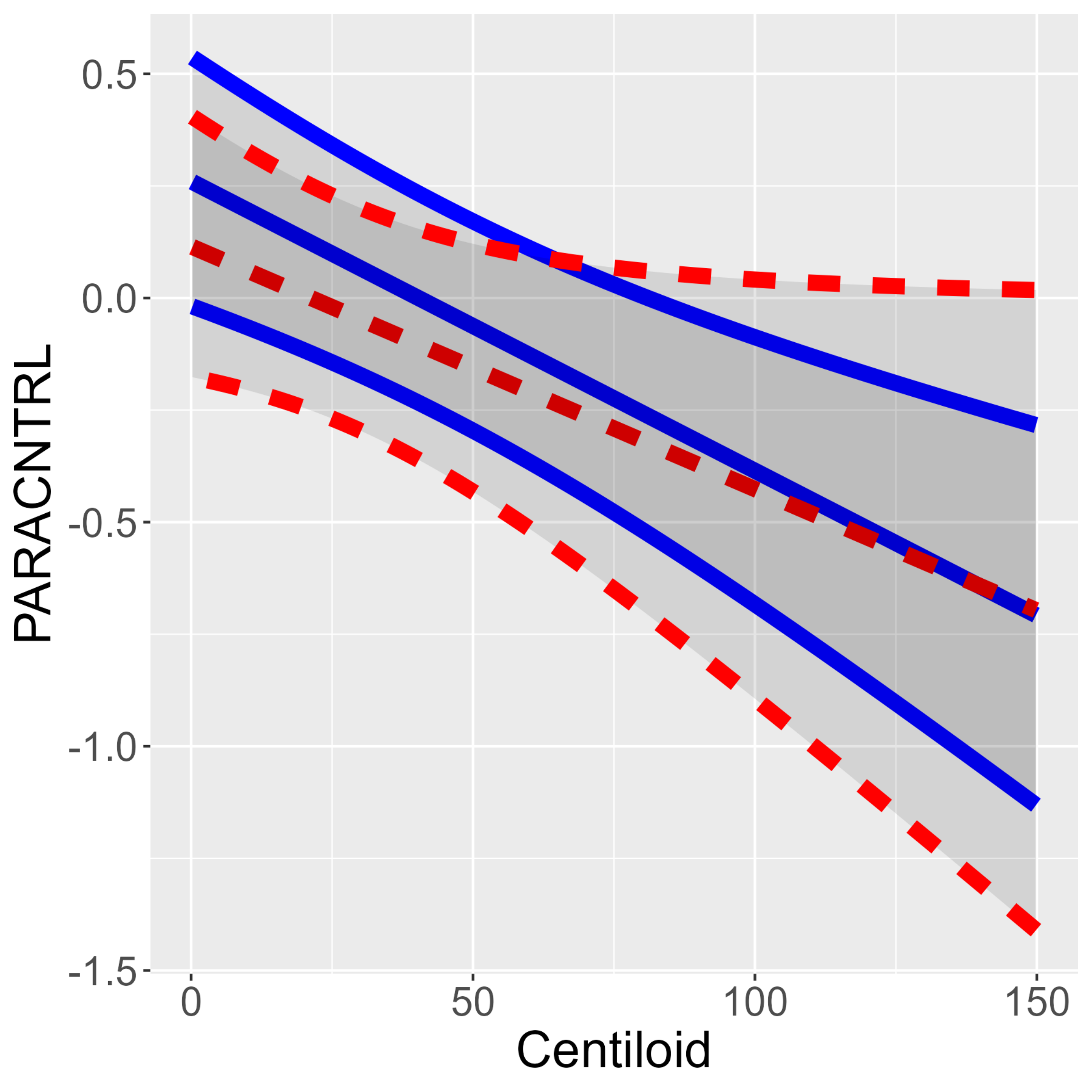

0.5
0.0
-0.5
-1.0
-1.5
PARACNTRL
0
50
100
150
Centiloid

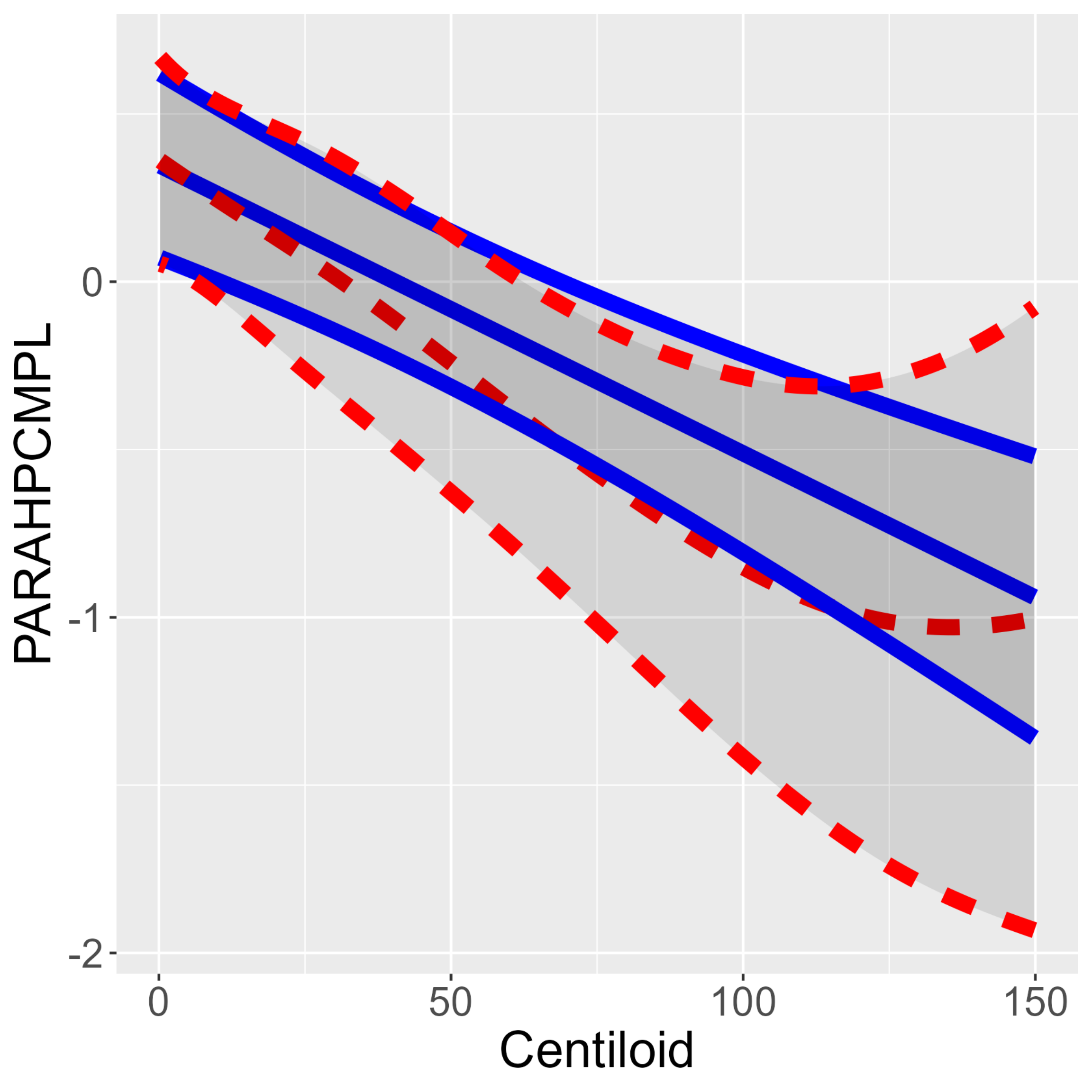

PARAHPCMPL
0
-1
-2
0
50
100
150
Centiloid

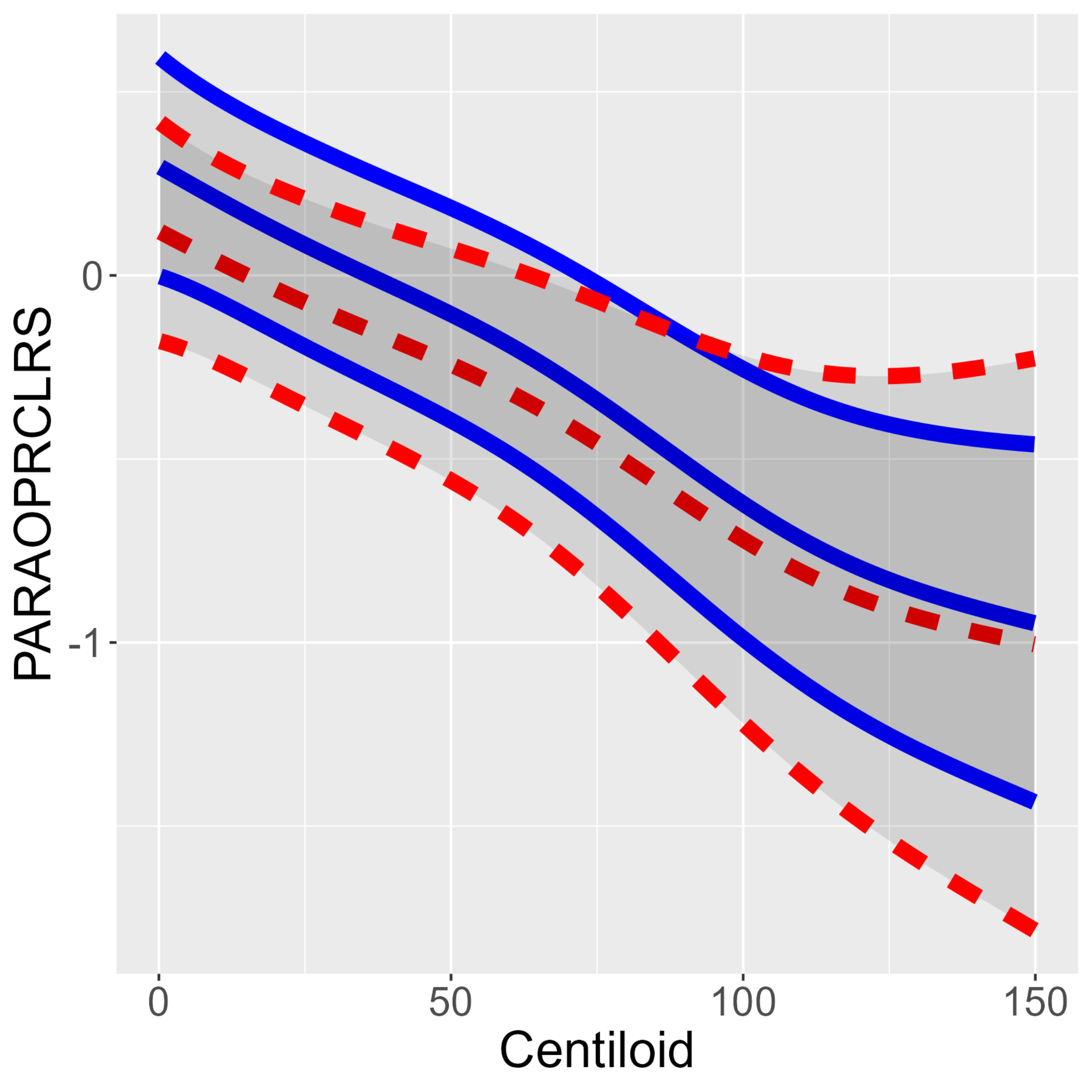

PARAOPRCLRS
0
-1
0
50
100
150
Centiloid

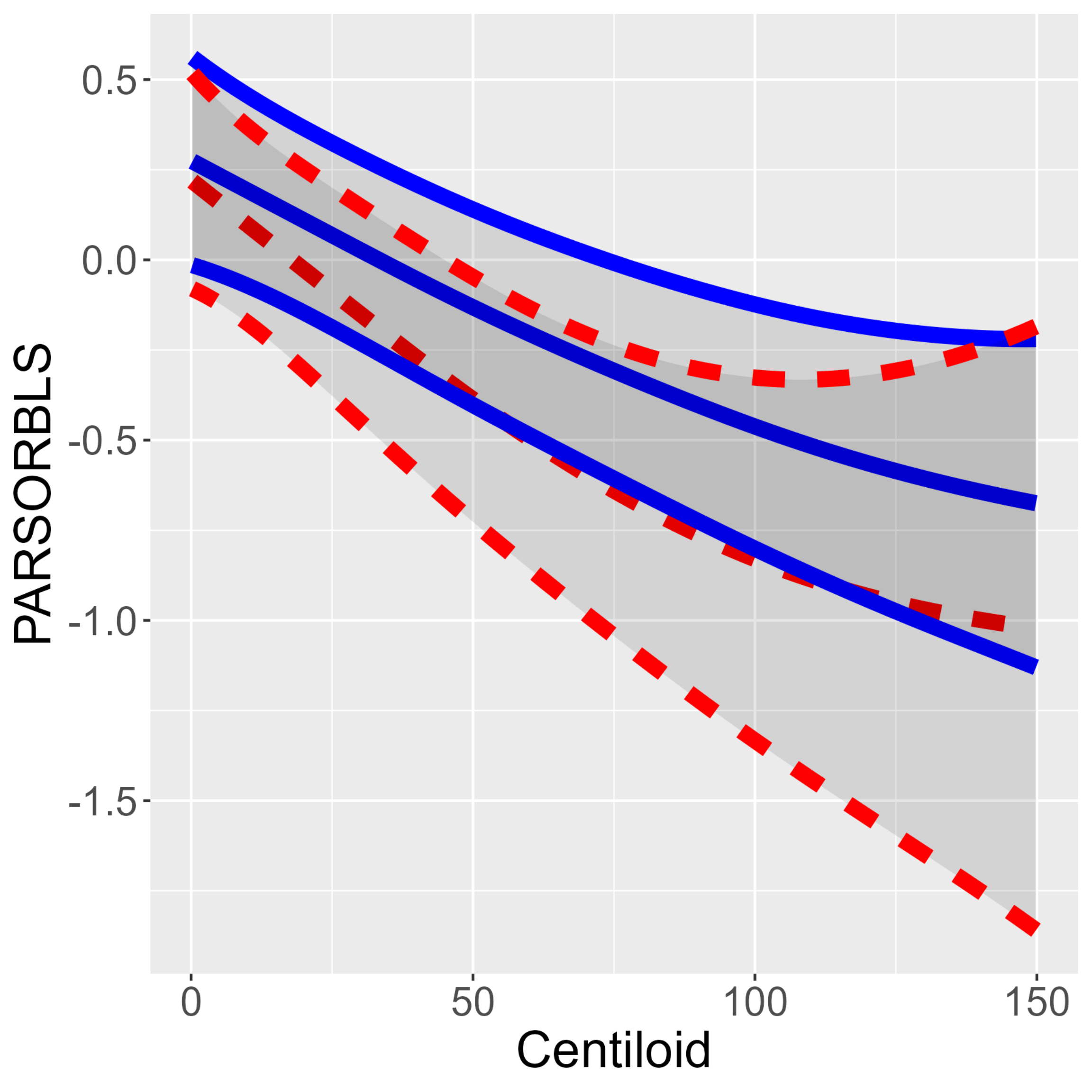

PARSORBLS
0.5
0.0
-0.5
-1.0
-1.5
0
50
100
150
Centiloid

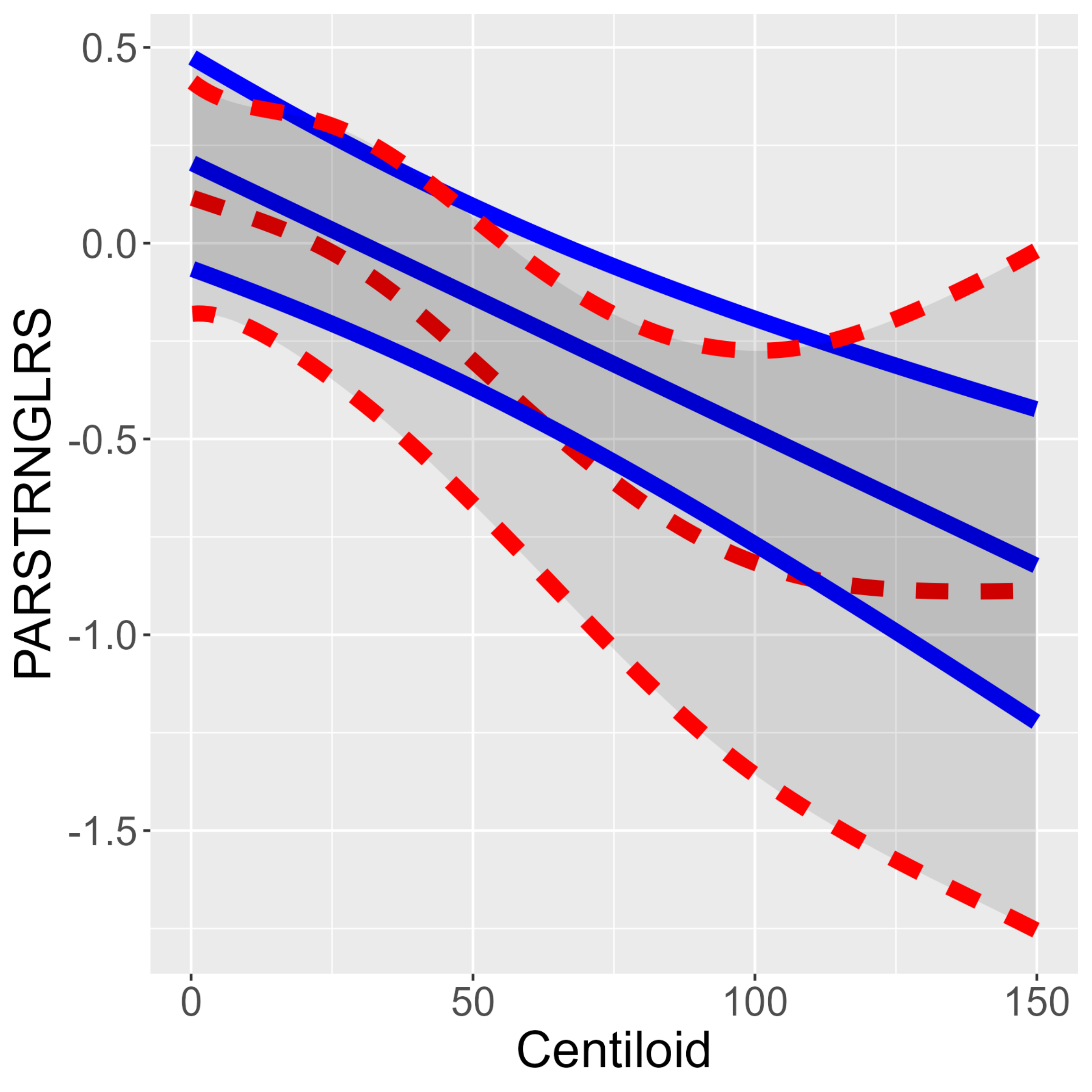

PARSTRNGLRS
0.5
0.0
-0.5
-1.0
-1.5
0
50
100
150
Centiloid

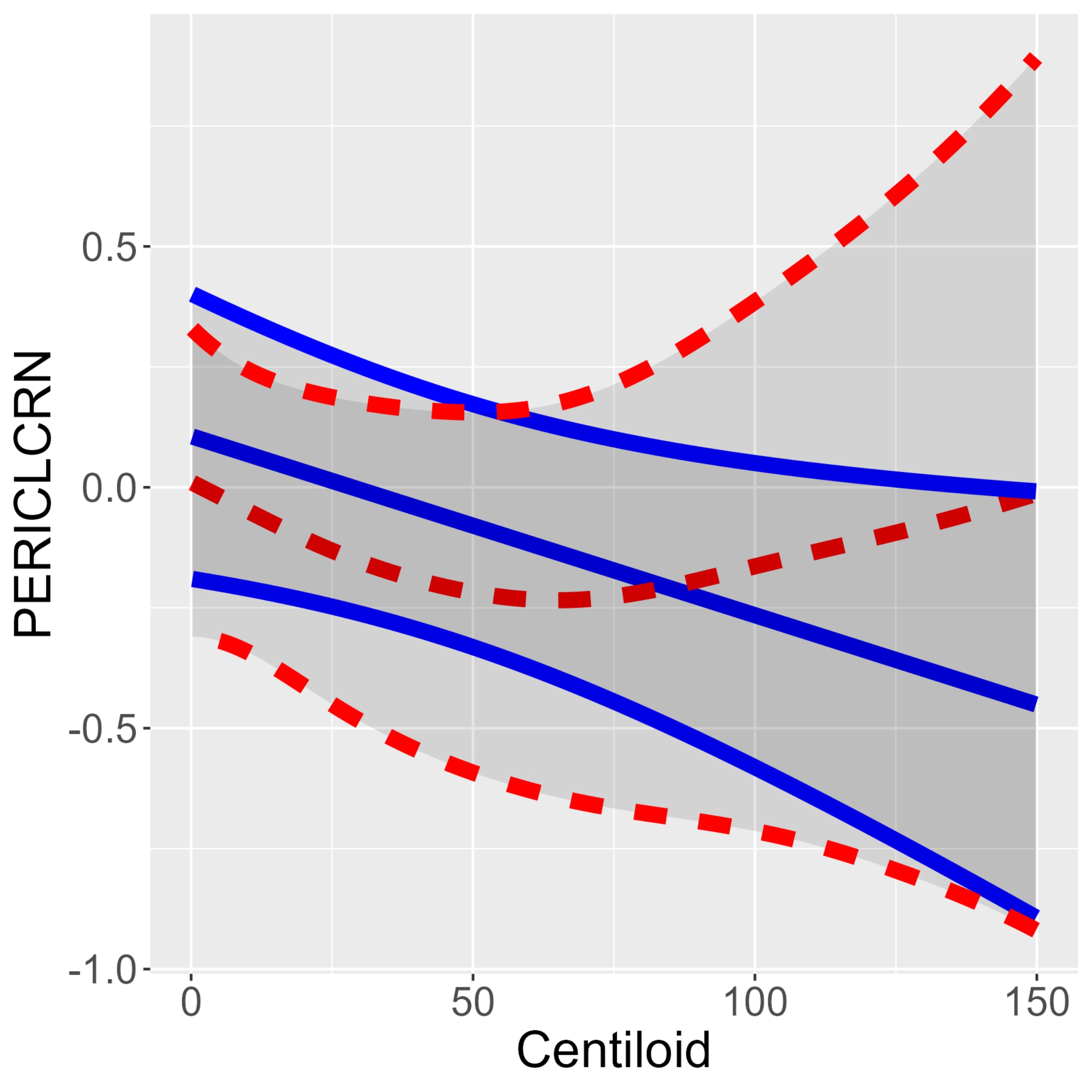

PERICLCRN
0.5
0.0
-0.5
-1.0
0
50
100
150
Centiloid

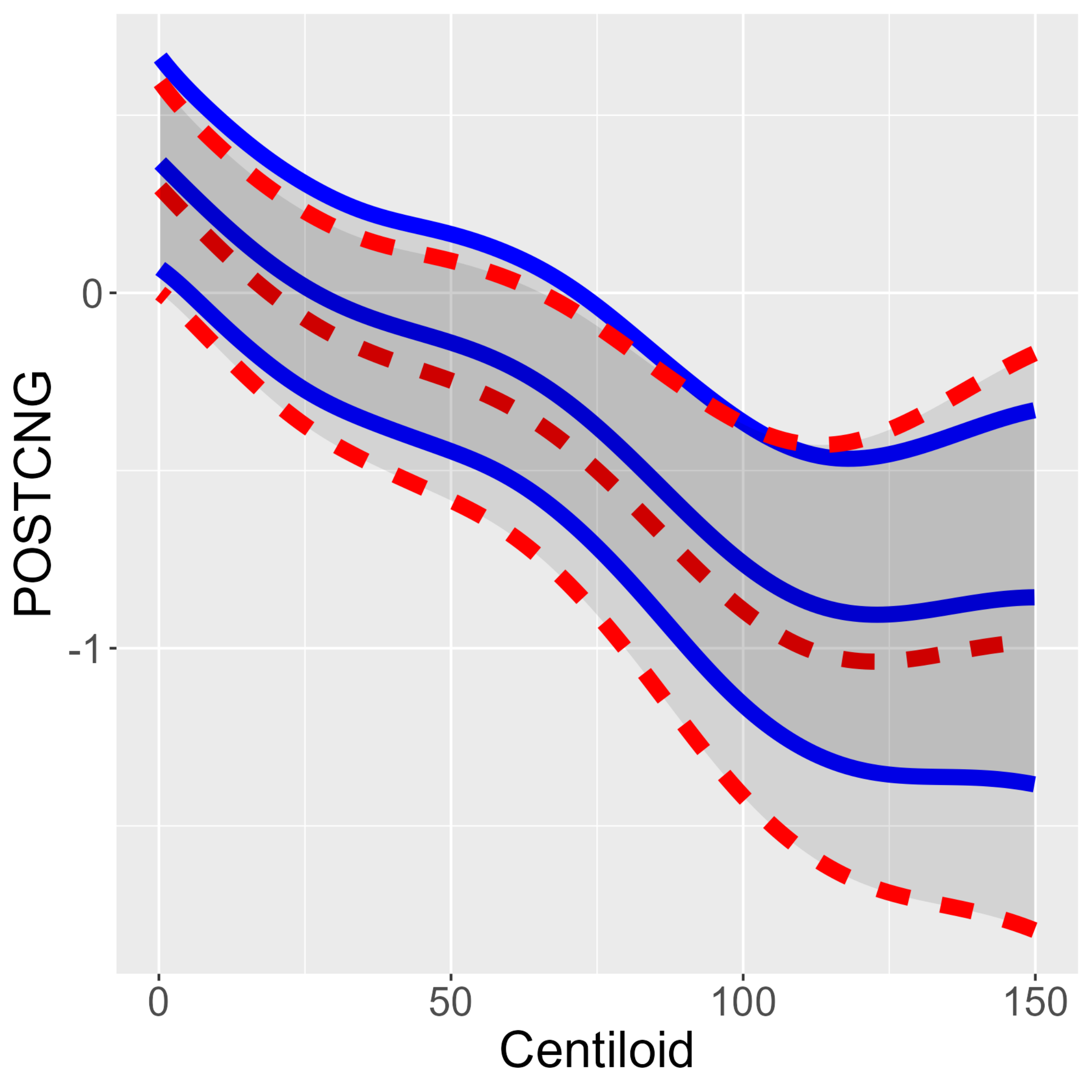

POSTCNG
0
-1
0
50
100
150
Centiloid

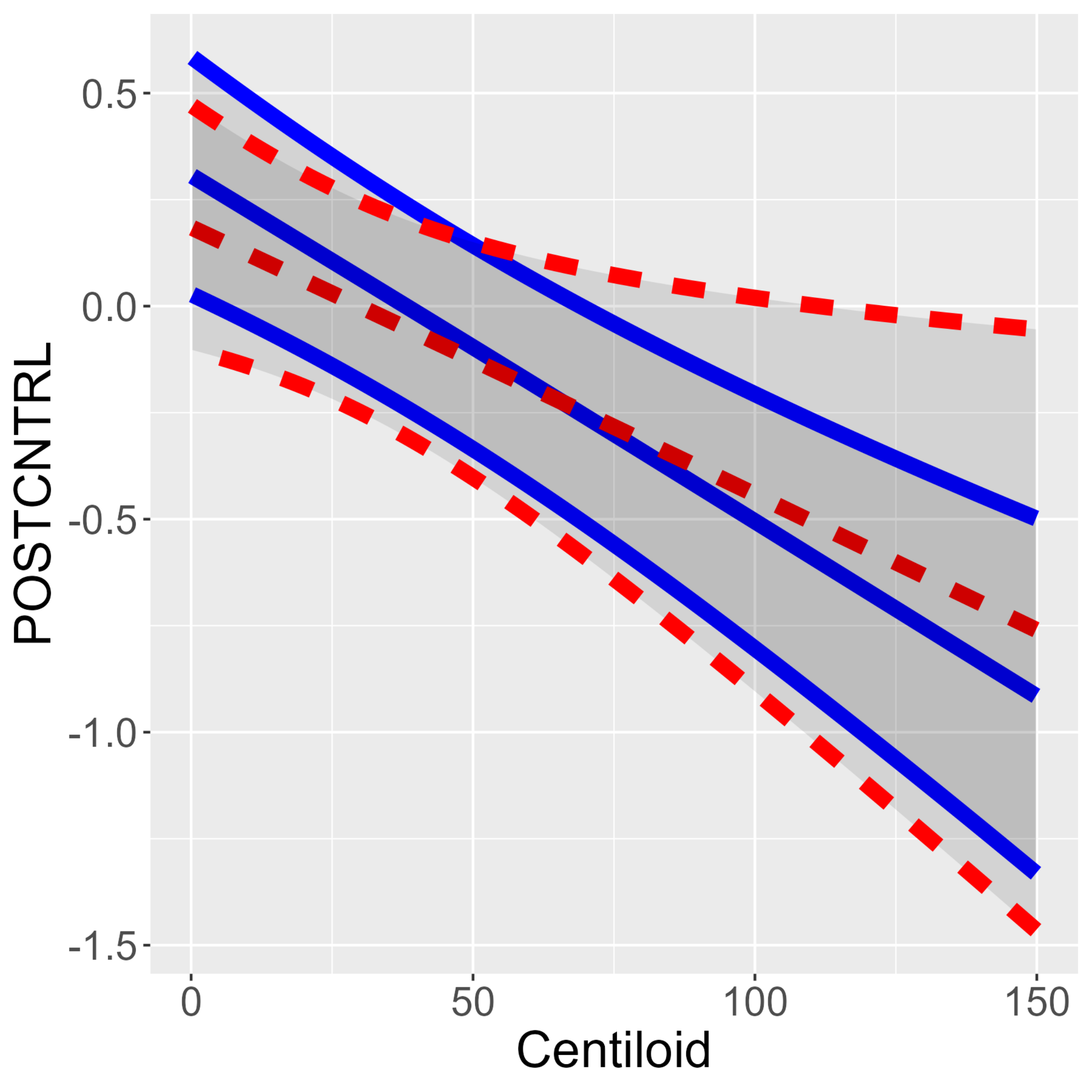

POSTCNTRL
0.5
0.0
-0.5
-1.0
-1.5
0
50
100
150
Centiloid

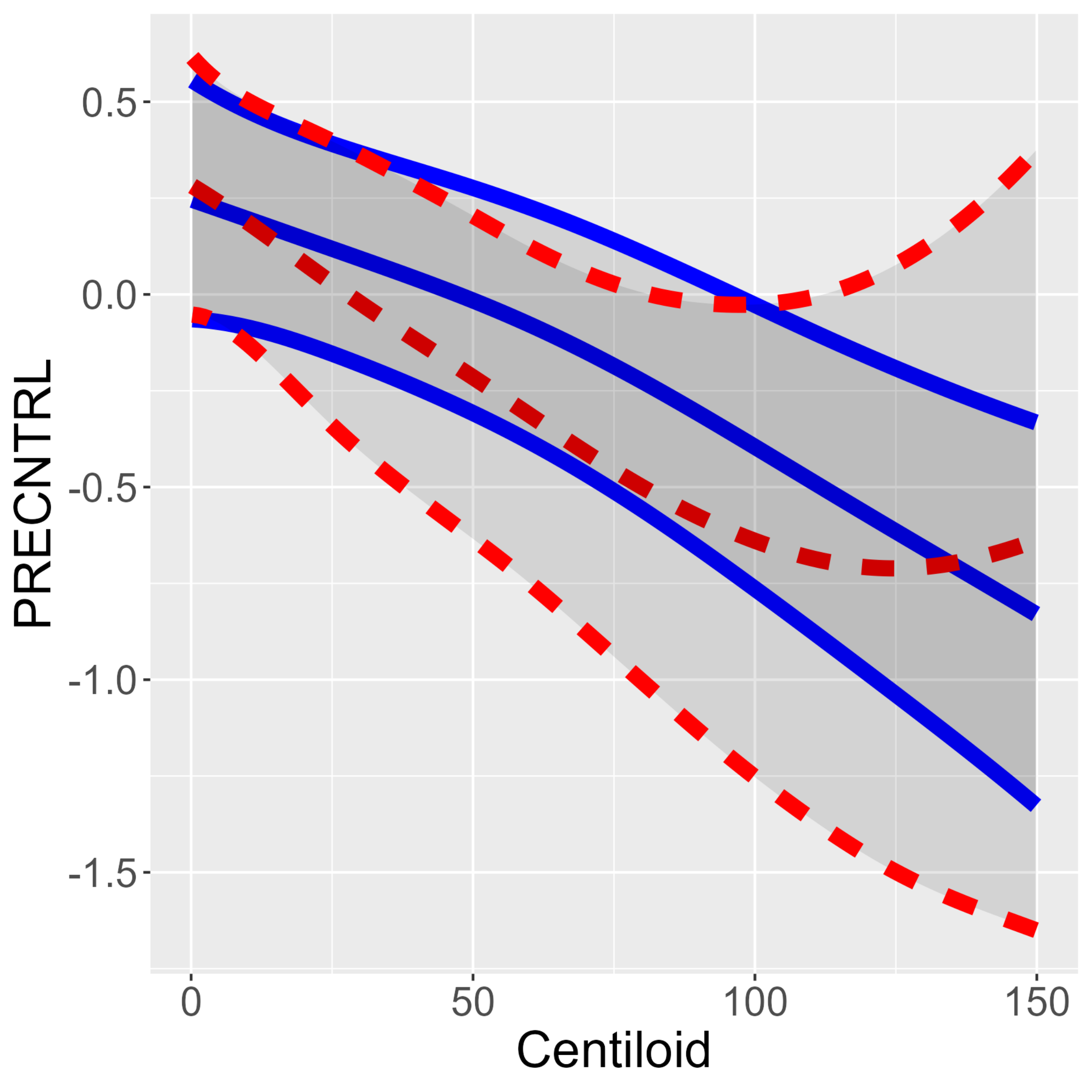

PRECNTRL
0.5
0.0
-0.5
-1.0
-1.5
0
50
100
150
Centiloid

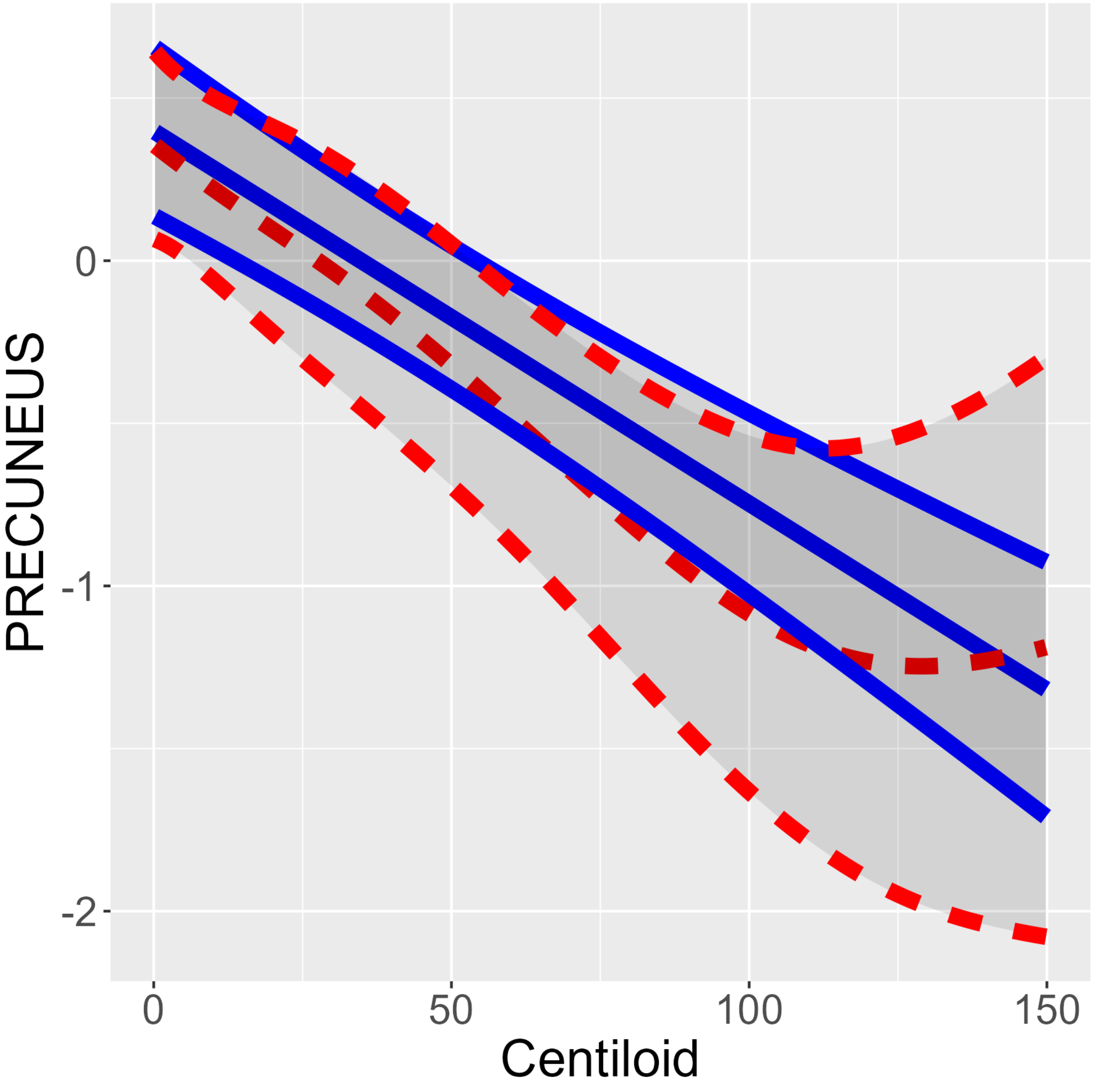
PRECUNEUS
0
-1
-2
0
50
100
150
Centiloid

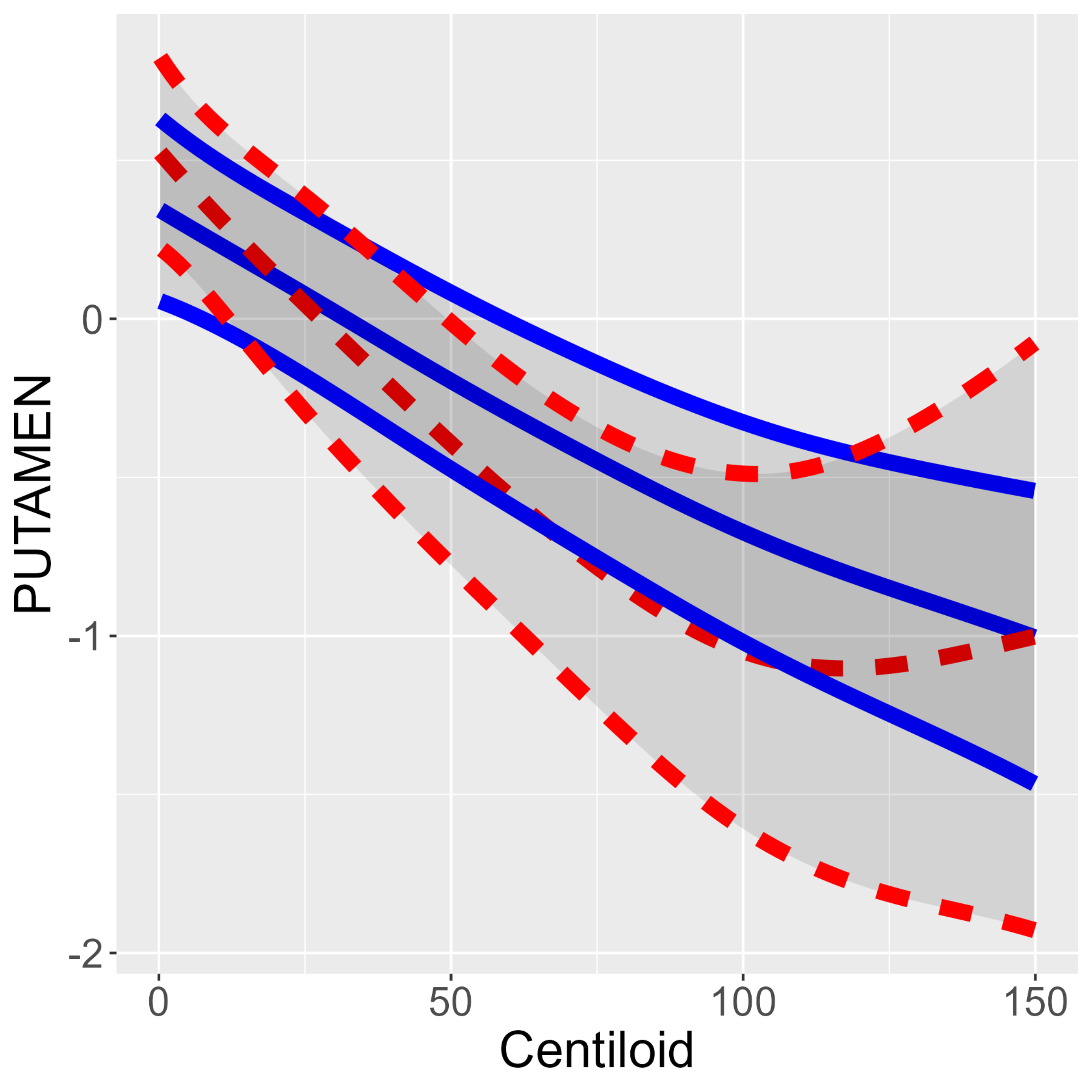

PUTAMEN
0
-1
-2
0
50
100
150
Centiloid

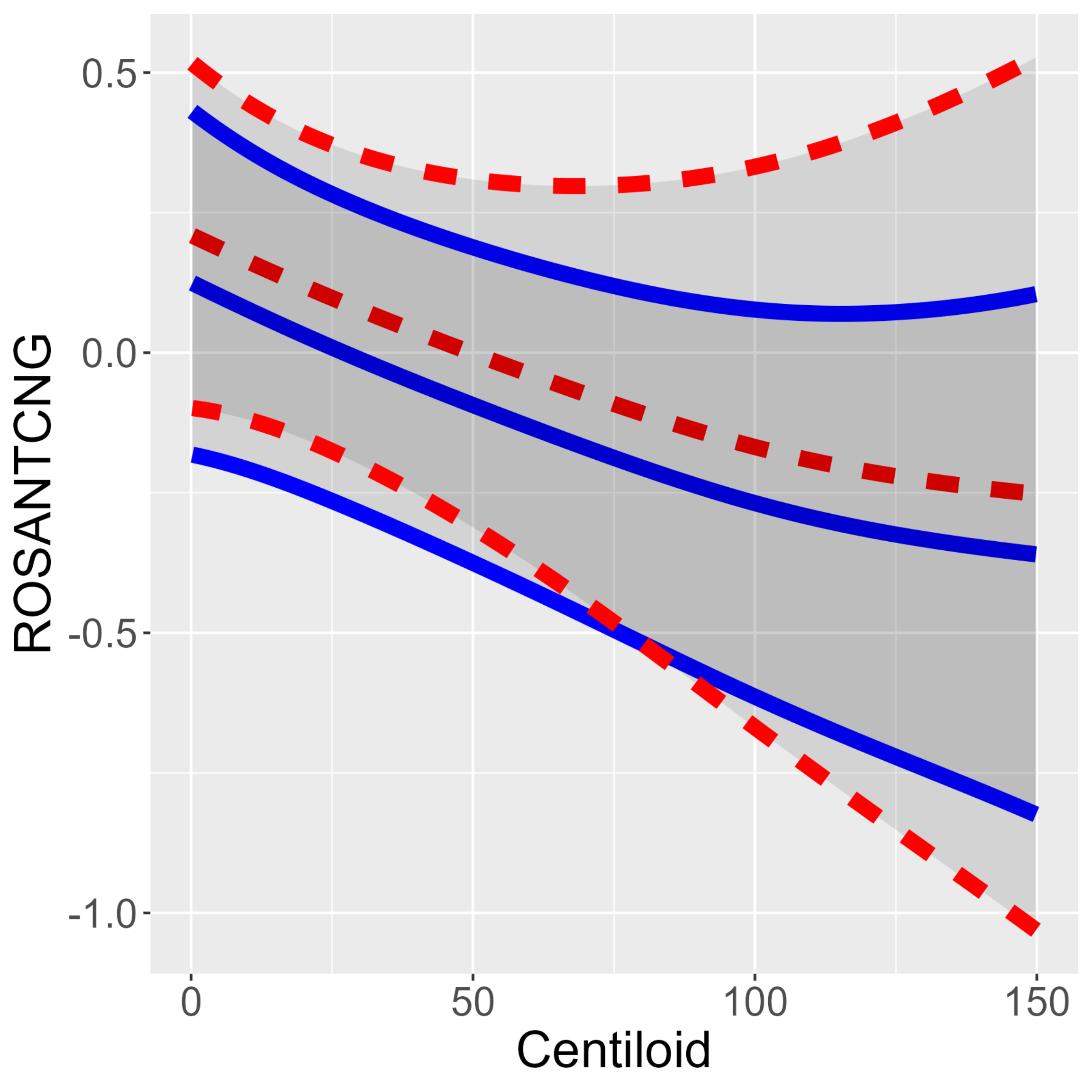

0.5
0.0
-0.5
-1.0
ROSANTCNG
0
50
100
150
Centiloid

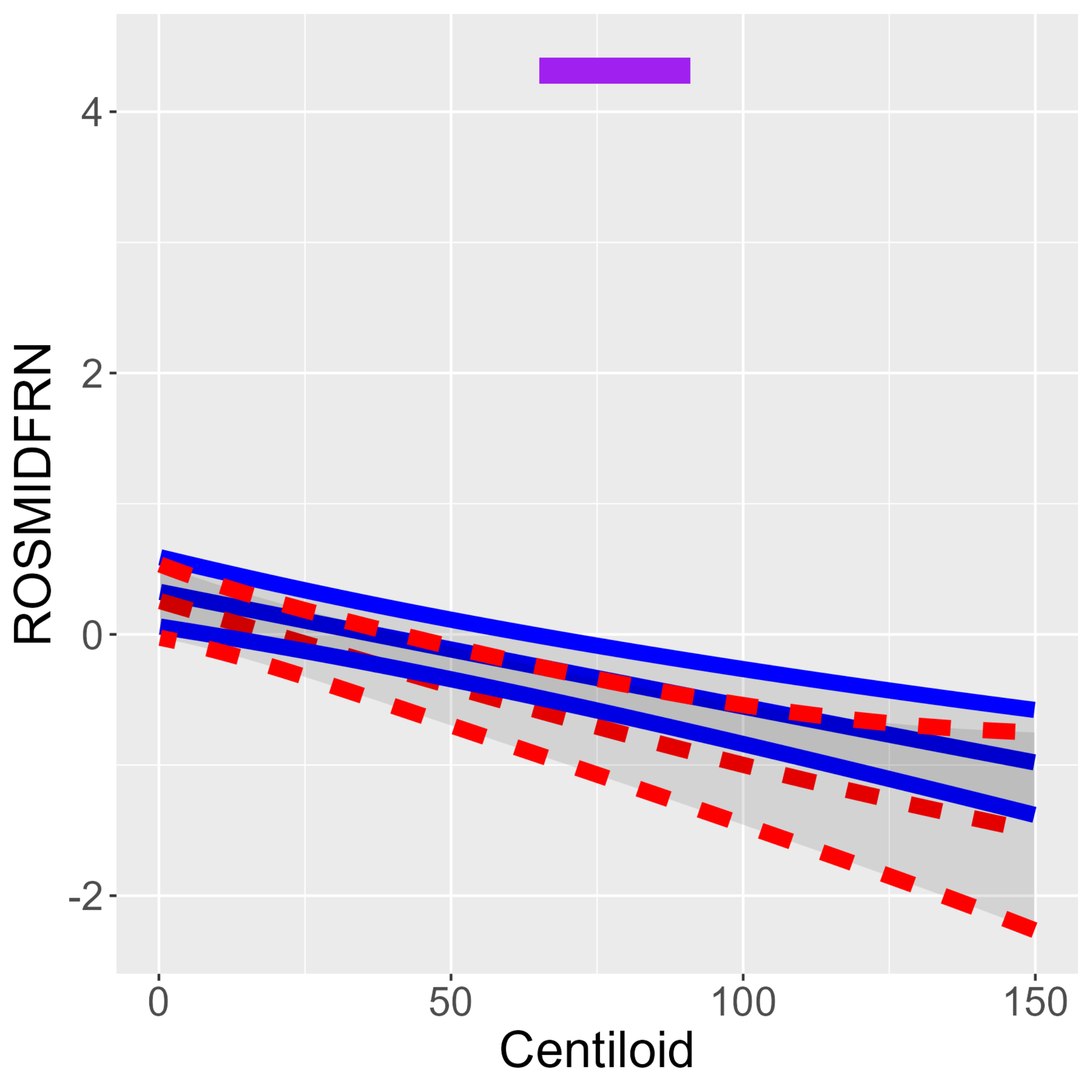

ROSMIDFRN
4
2
0
-2
0
50
100
150
Centiloid

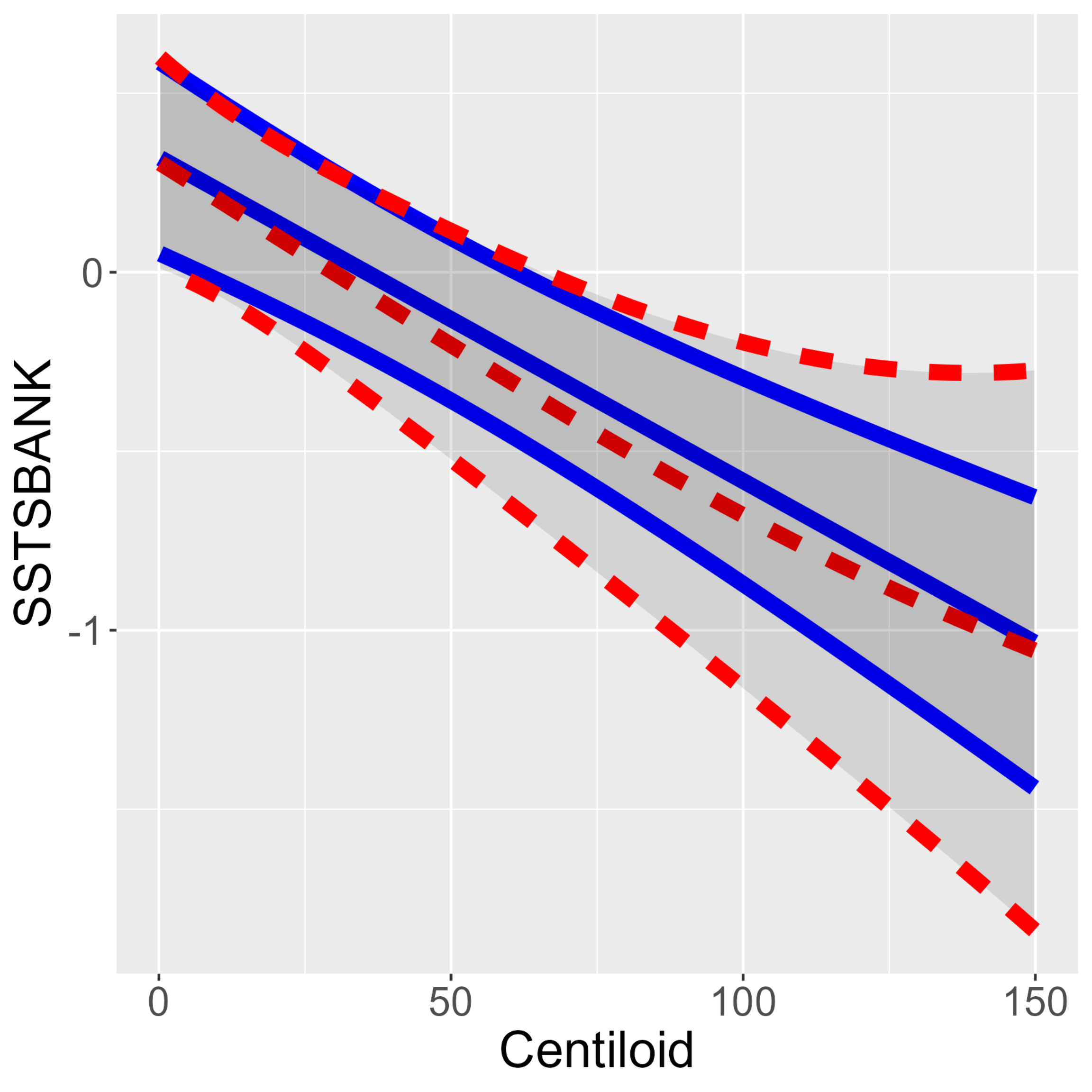

SSTSBANK
0
-1
0
50
100
150
Centiloid

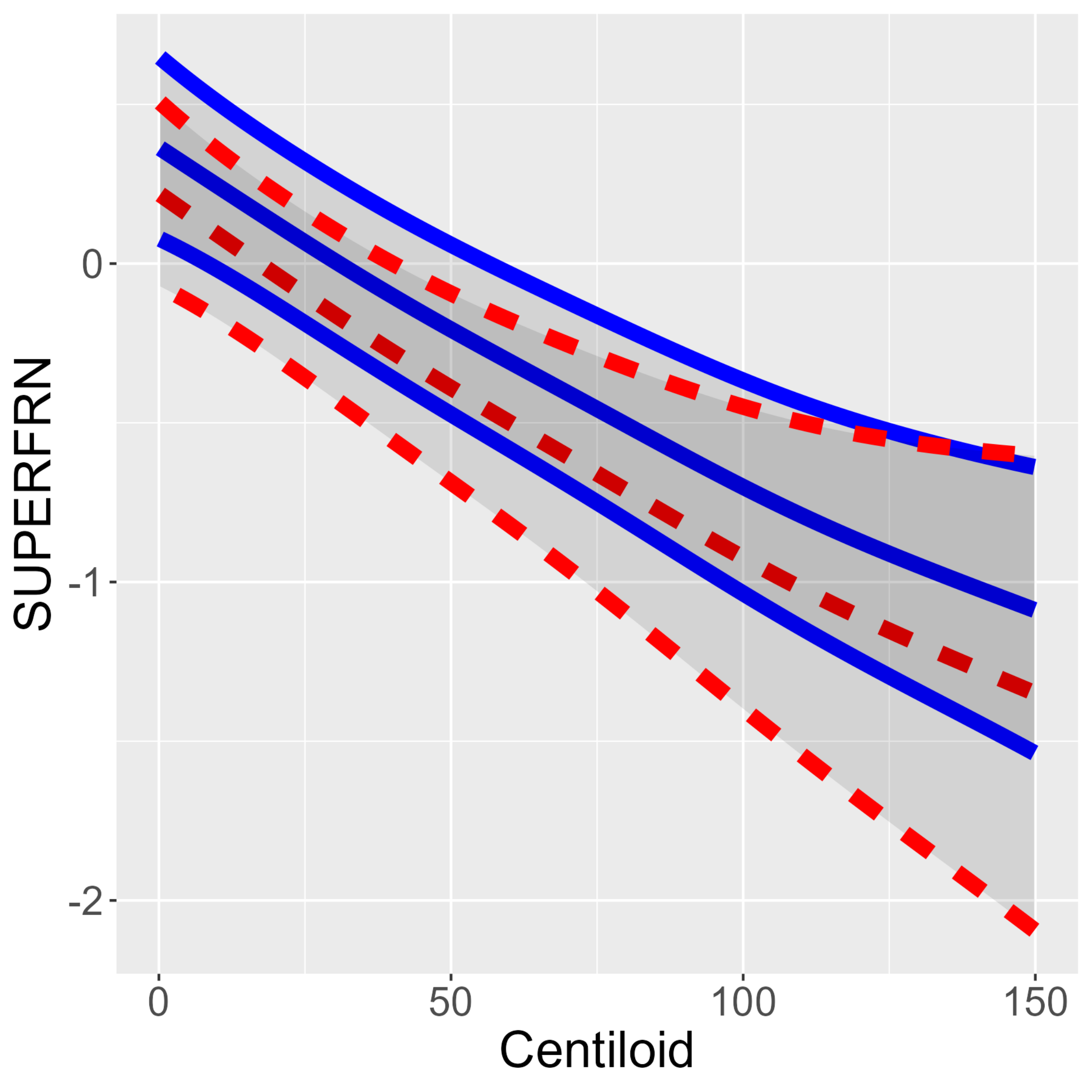

SUPERFRN
0
-1
-2
0
50
100
150
Centiloid

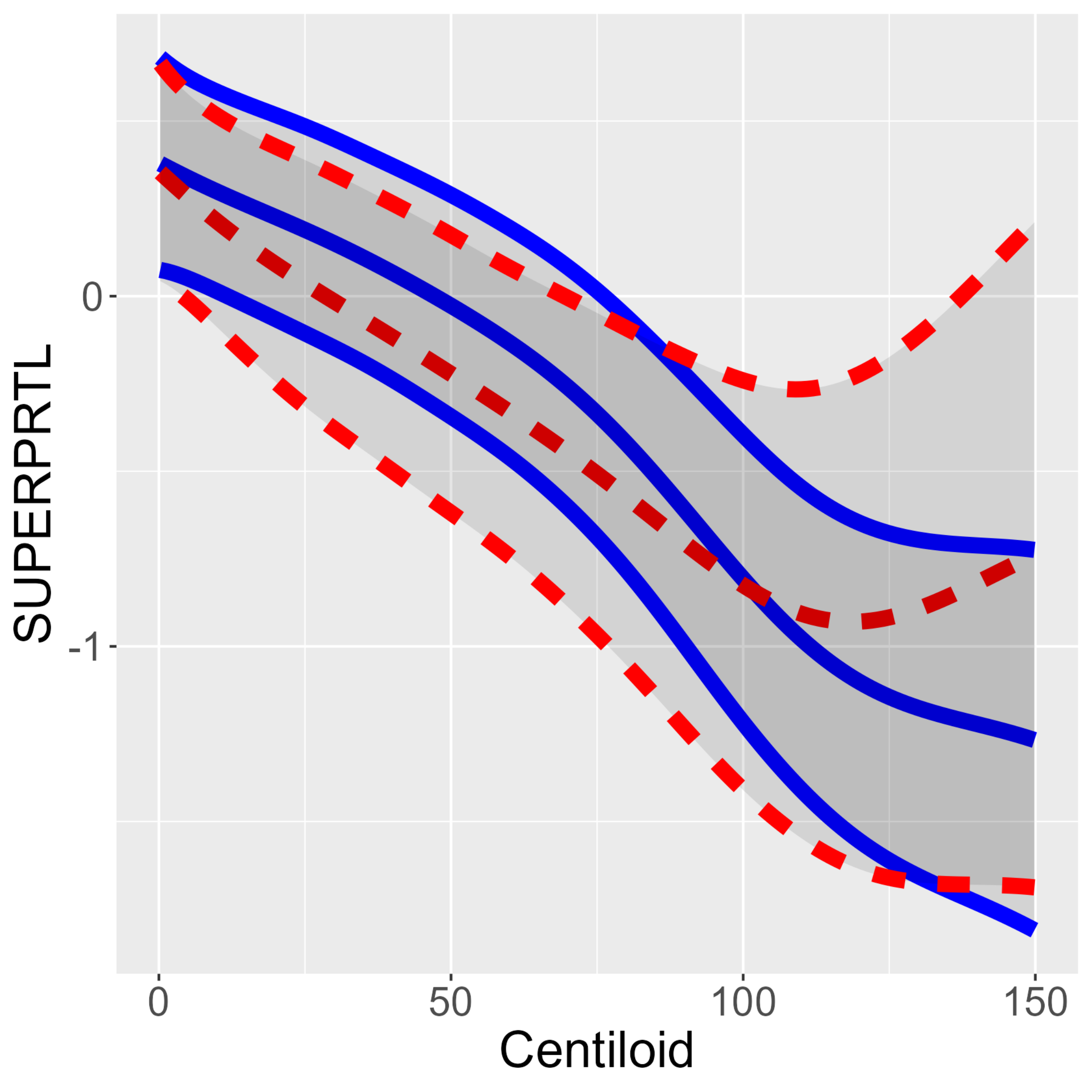

0
-1
SUPERPRTL
0
50
100
150
Centiloid

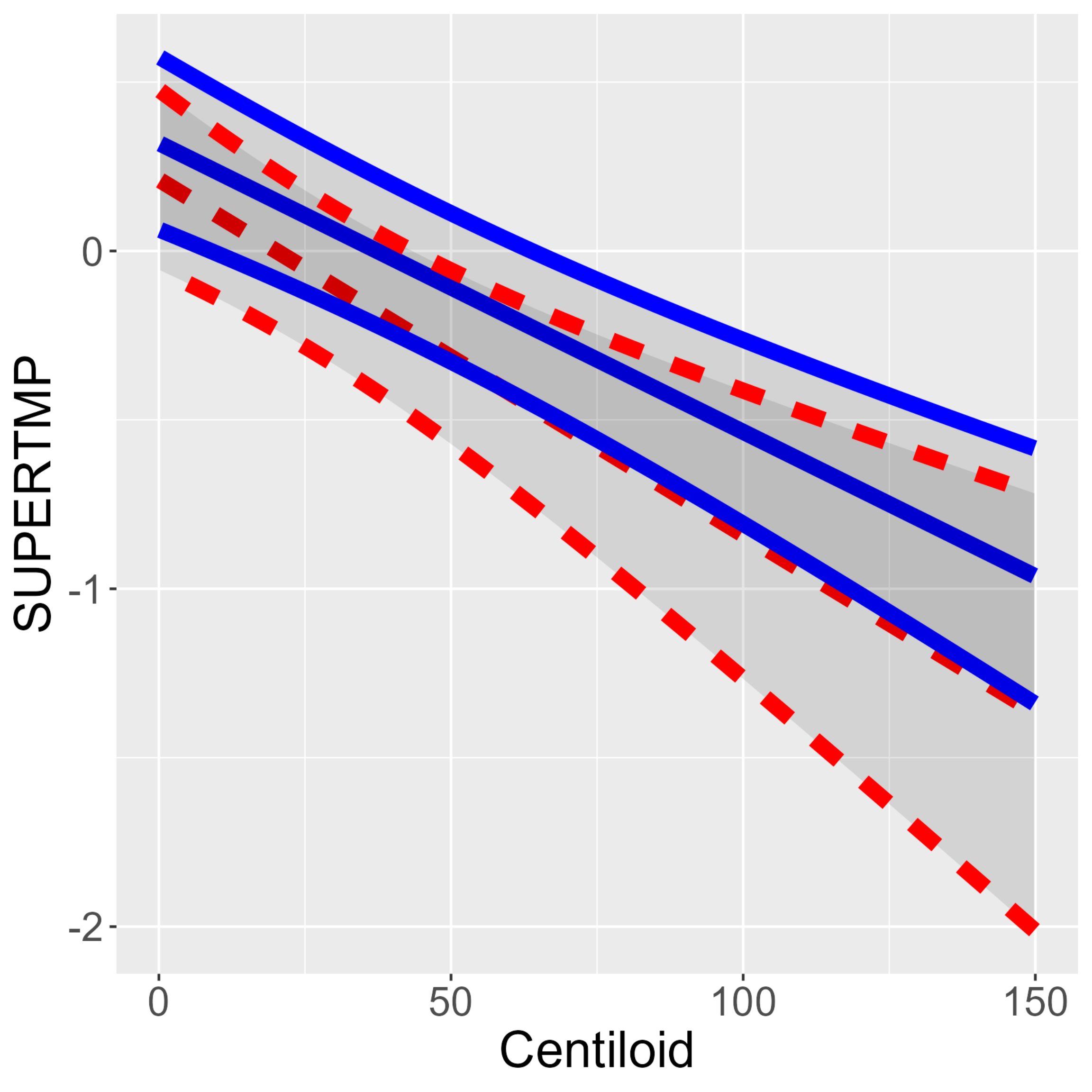

SUPERTMP
0
-1
-2
0
50
100
150
Centiloid

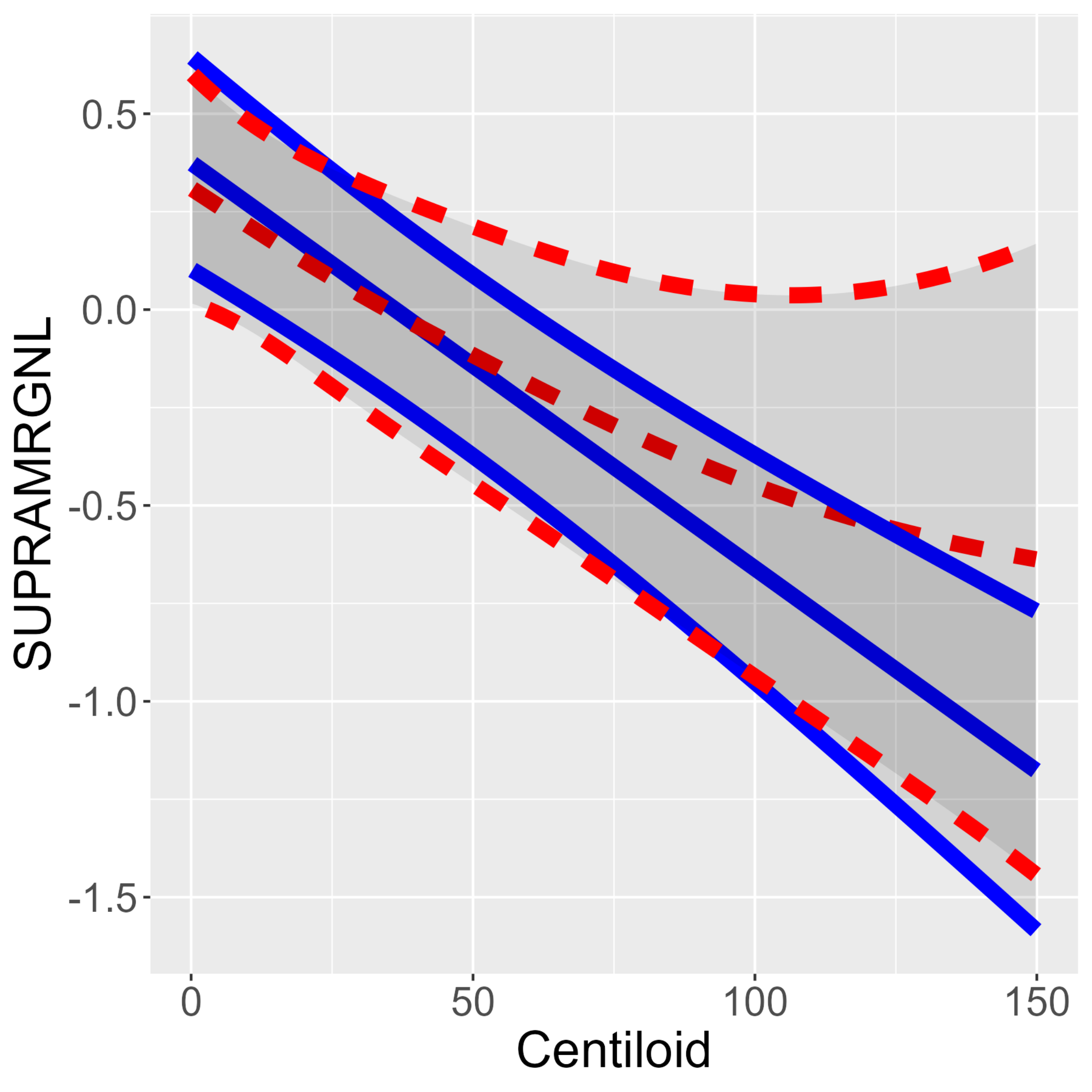

0.5
0.0
-0.5
-1.0
-1.5
SUPRAMRGNL
0
50
100
150
Centiloid

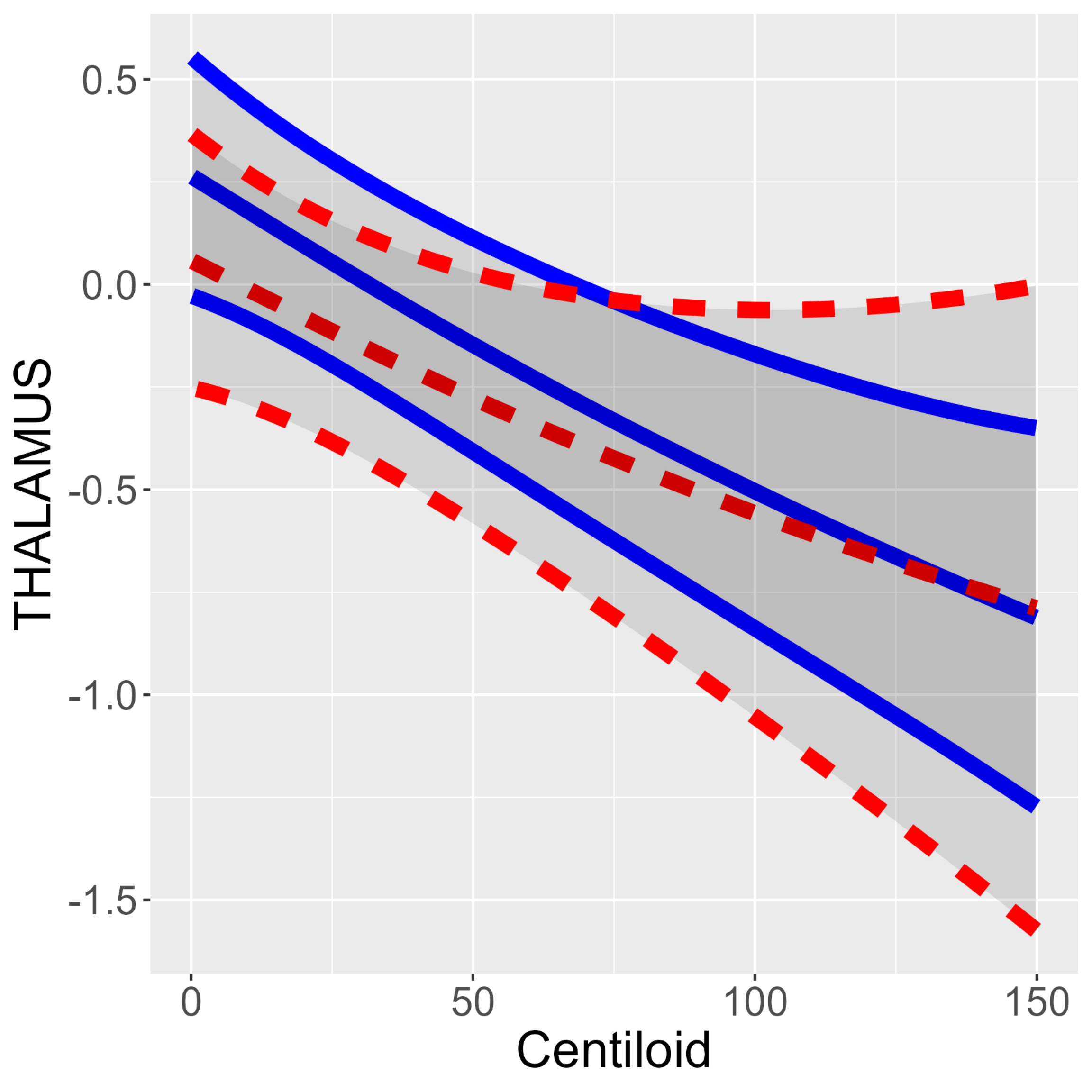

0.5
0.0
-0.5
-1.0
-1.5
THALAMUS
0
50
100
150
Centiloid

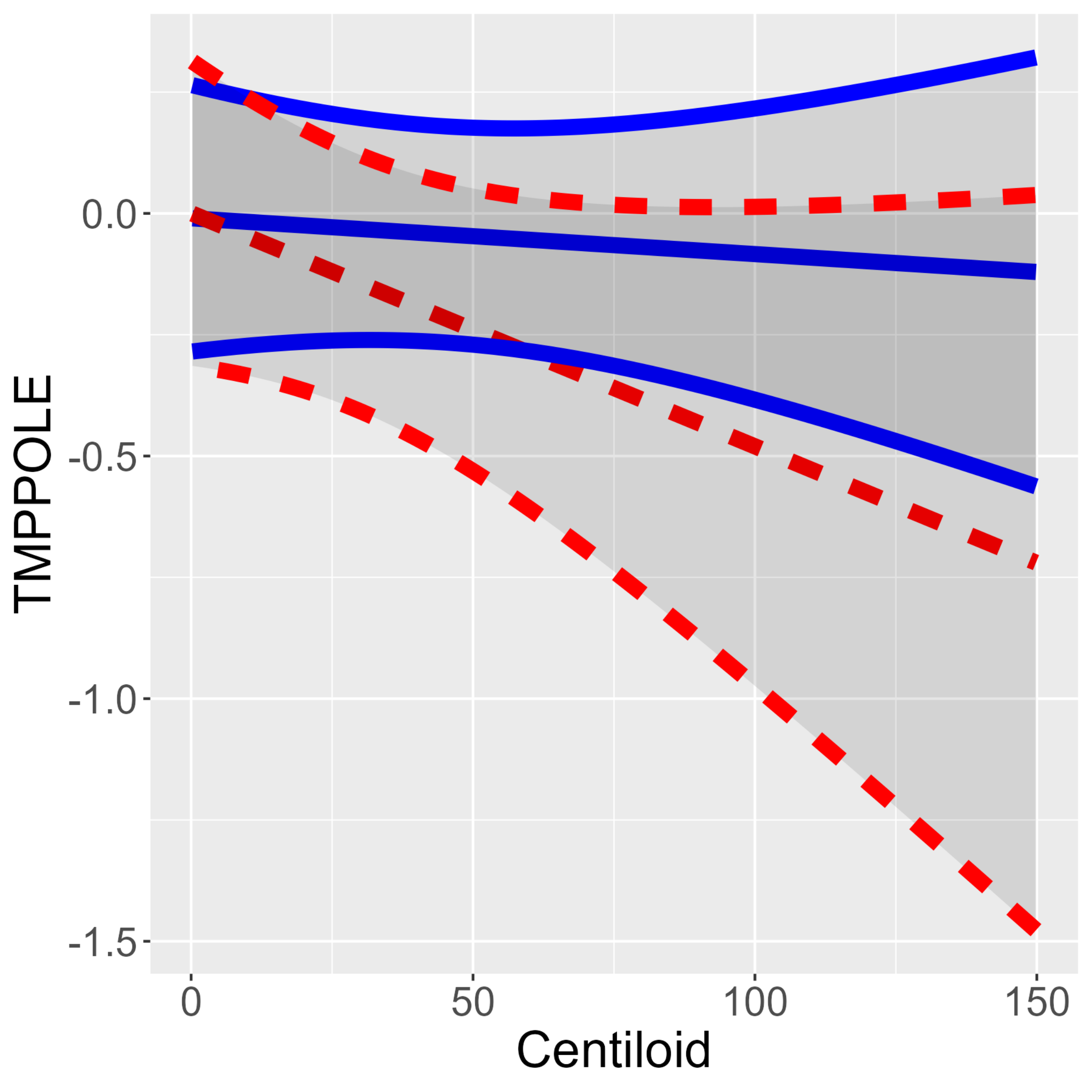

0.0
-0.5
-1.0
-1.5
TMPPOLE
0
50
100
150
Centiloid

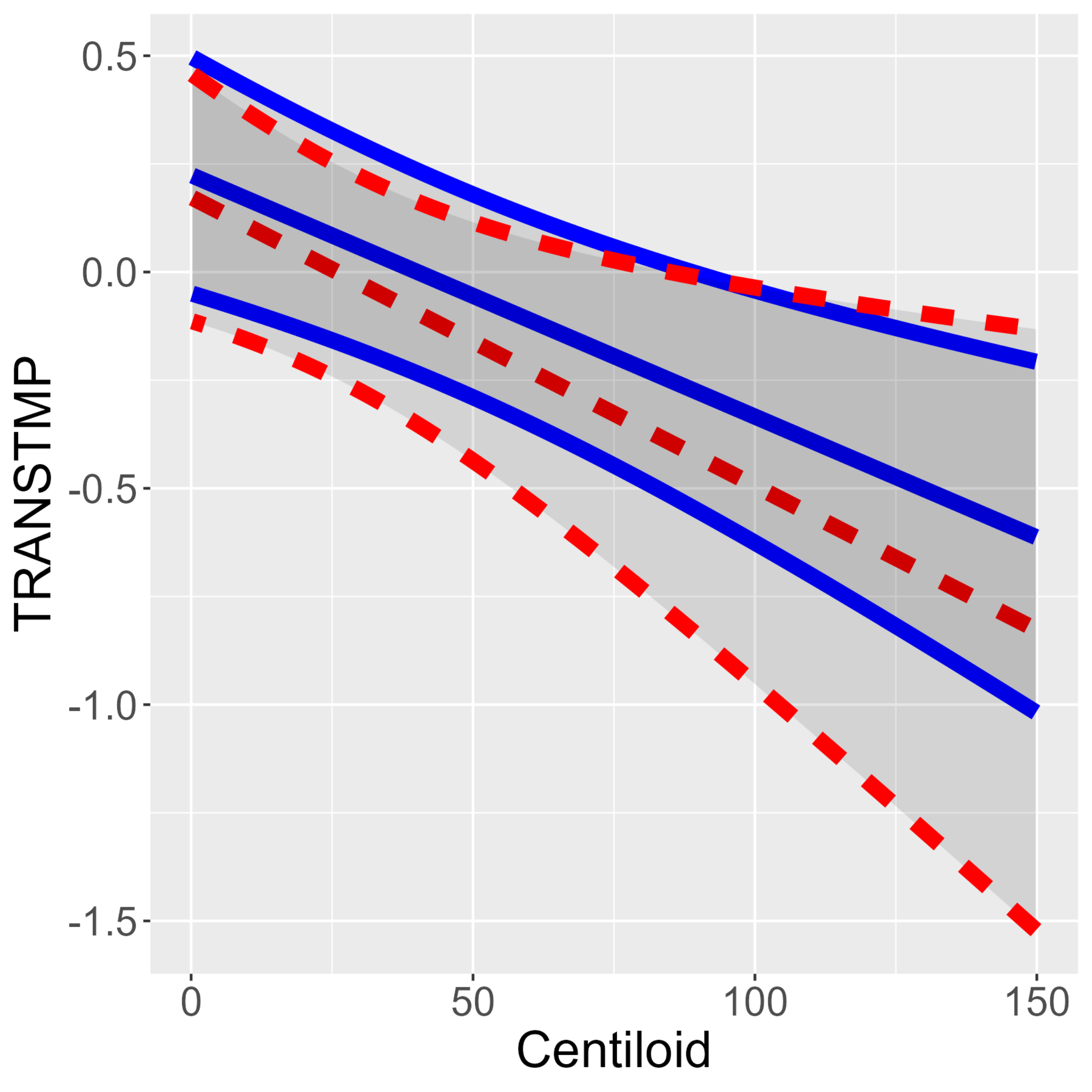

0.5
0.0
-0.5
-1.0
-1.5
TRANSTMP
0
50
100
150
Centiloid

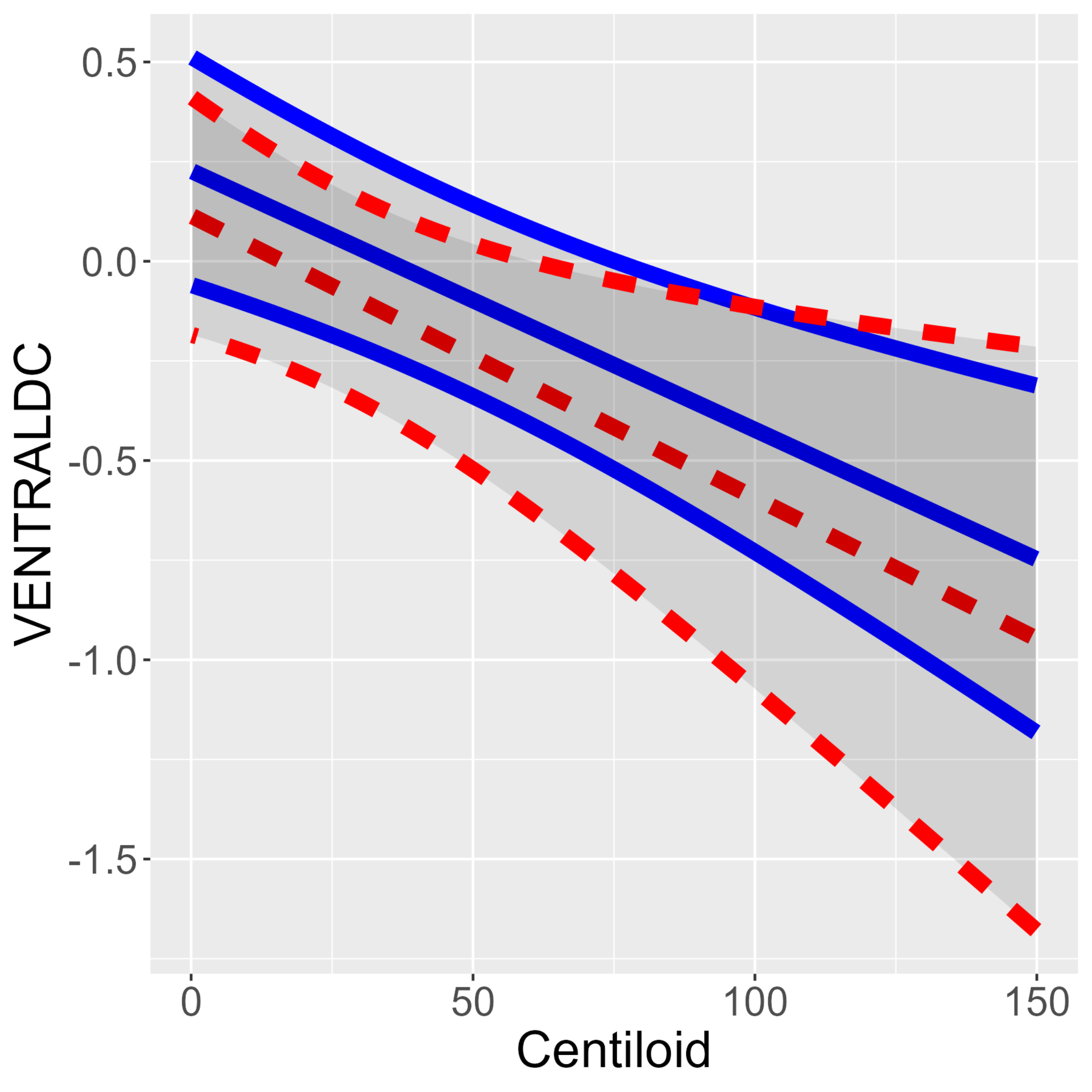

VENTRALDC
0.5
0.0
-0.5
-1.0
-1.5
0
50
100
150
Centiloid
