# Supplementary material for: Brain volume trajectories in Down syndrome and autosomal dominant Alzheimer's disease
Source: Alzheimers Dement. 2026 Jan 18;22(1):e71103. doi: 10.1002/alz.71103 (PMC12812856; doi:10.1002/alz.71103)
Supplement: Supplementary file 10 — Supporting information [file ALZ-22-e71103-s015.pdf]

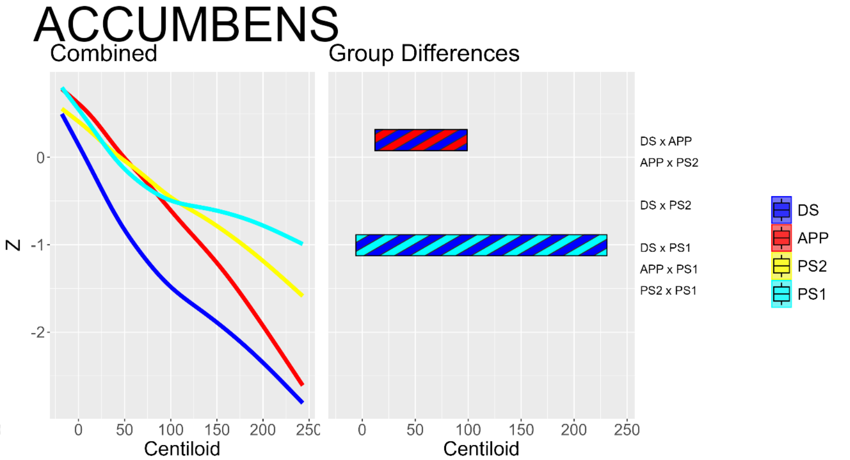

ACCUMBENS
Combined
Group Differences
Z
0
-1
-2
0
50
100
150
200
250
Centiloid
DS x APP
APP x PS2
DS x PS2
DS x PS1
APP x PS1
PS2 x PS1
DS
APP
PS2
PS1

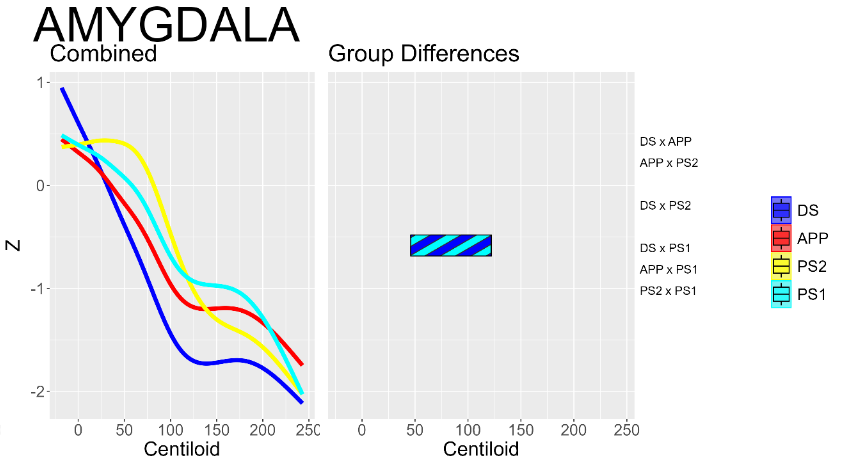

AMYGDALA
Combined
Group Differences
Z
1
0
-1
-2
0
50
100
150
200
250
Centiloid
DS x APP
APP x PS2
DS x PS2
DS x PS1
APP x PS1
PS2 x PS1
DS
APP
PS2
PS1

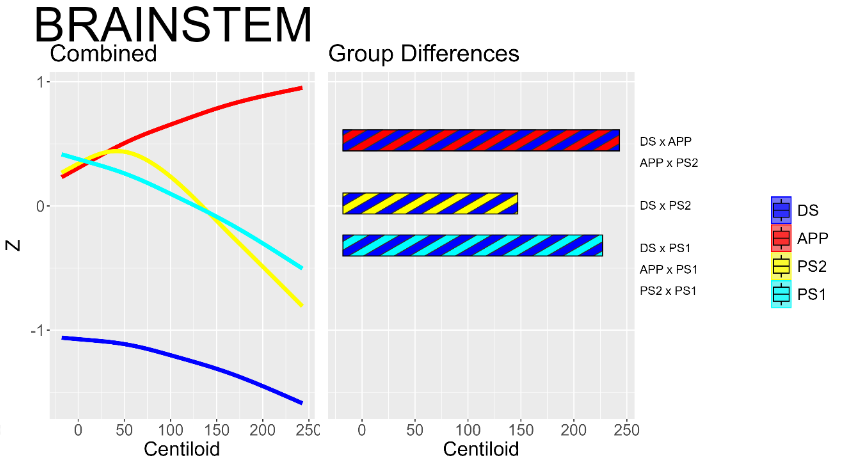
BRAINSTEM
Combined
Group Differences
Z
1
0
-1
0
50
100
150
200
250
Centiloid
0
50
100
150
200
250
Centiloid
DS x APP
APP x PS2
DS x PS2
DS x PS1
APP x PS1
PS2 x PS1
DS
APP
PS2
PS1

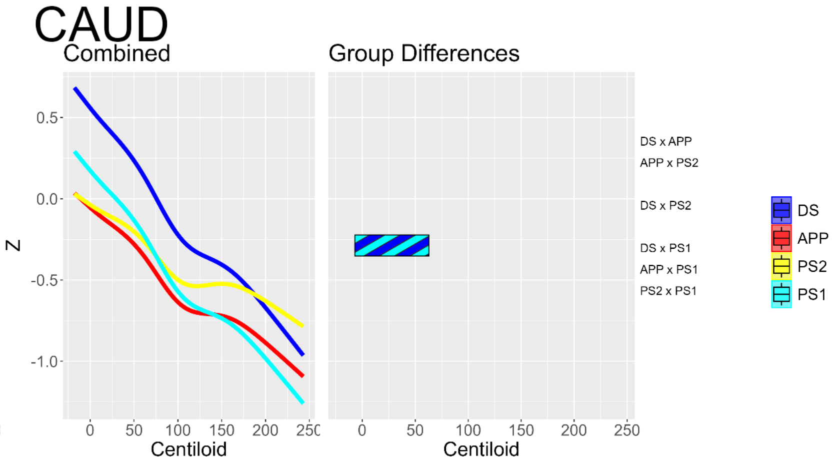

CAUD
Combined
Group Differences
Z
0.5
0.0
-0.5
-1.0
0
50
100
150
200
250
Centiloid
Centiloid
DS x APP
APP x PS2
DS x PS2
DS x PS1
APP x PS1
PS2 x PS1
DS
APP
PS2
PS1

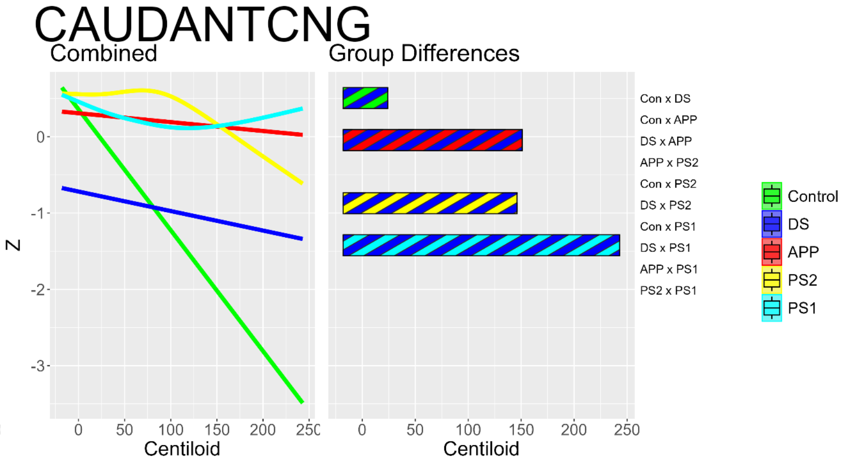
CAUDANTCNG
Combined
Group Differences
Z
0
-1
-2
-3
0
50
100
150
200
250
Centiloid
Con x DS
Con x APP
DS x APP
APP x PS2
Con x PS2
DS x PS2
Con x PS1
DS x PS1
APP x PS1
PS2 x PS1
Control
DS
APP
PS2
PS1

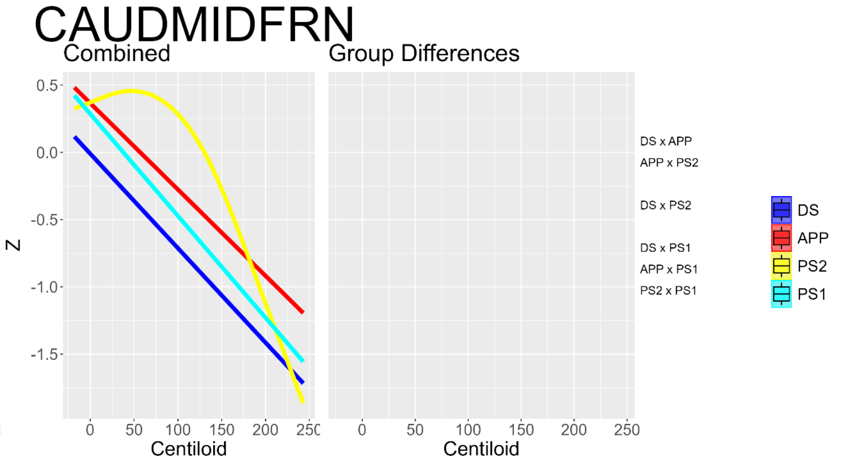
CAUDMIDFRN
Combined
Group Differences
Z
0.5
0.0
-0.5
-1.0
-1.5
0
50
100
150
200
250
Centiloid
DS x APP
APP x PS2
DS x PS2
DS x PS1
APP x PS1
PS2 x PS1
DS
APP
PS2
PS1

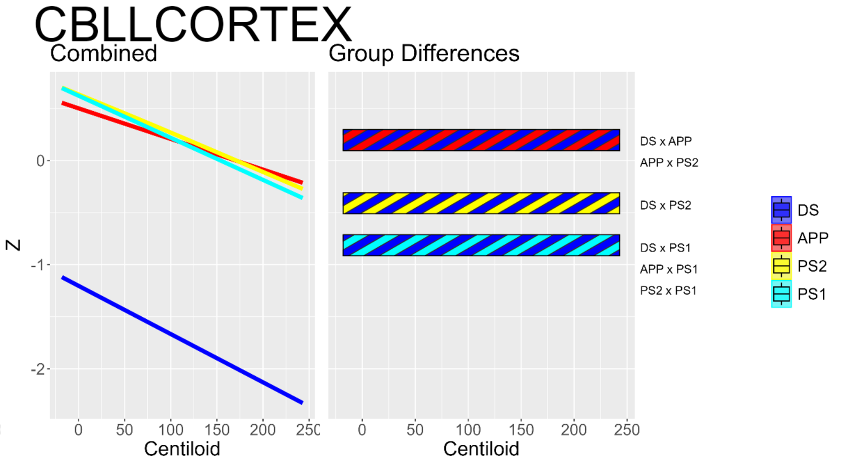
CBLLCORTEX
Combined
Group Differences
Z
0
-1
-2
0
50
100
150
200
250
Centiloid
DS x APP
APP x PS2
DS x PS2
DS x PS1
APP x PS1
PS2 x PS1
DS
APP
PS2
PS1

CUNEUS

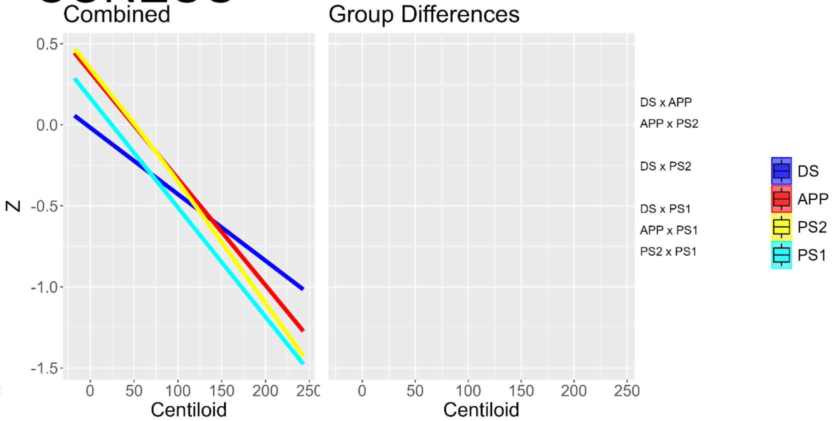

Combined
Group Differences
0.5
0.0
-0.5
-1.0
-1.5
Z
0
50
100
150
200
250
Centiloid
DS x APP
APP x PS2
DS x PS2
DS x PS1
APP x PS1
PS2 x PS1
DS
APP
PS2
PS1

ENTORHINAL

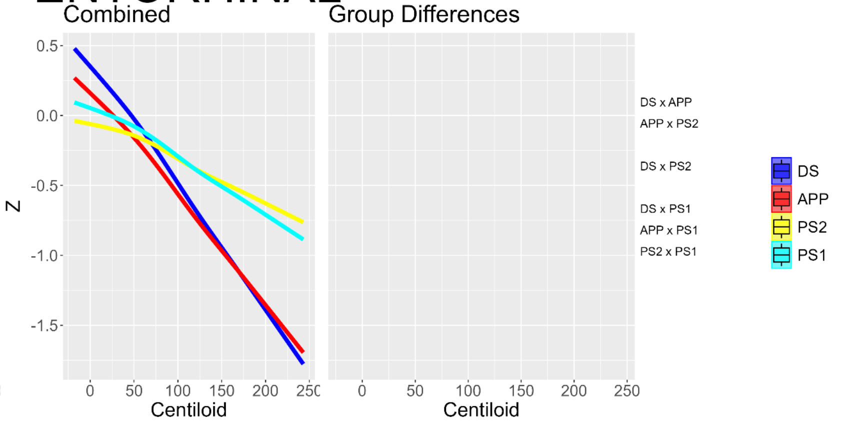

Combined
Group Differences
0.5
0.0
-0.5
-1.0
-1.5
Z
0
50
100
150
200
250
Centiloid
DS x APP
APP x PS2
DS x PS2
DS x PS1
APP x PS1
PS2 x PS1
DS
APP
PS2
PS1

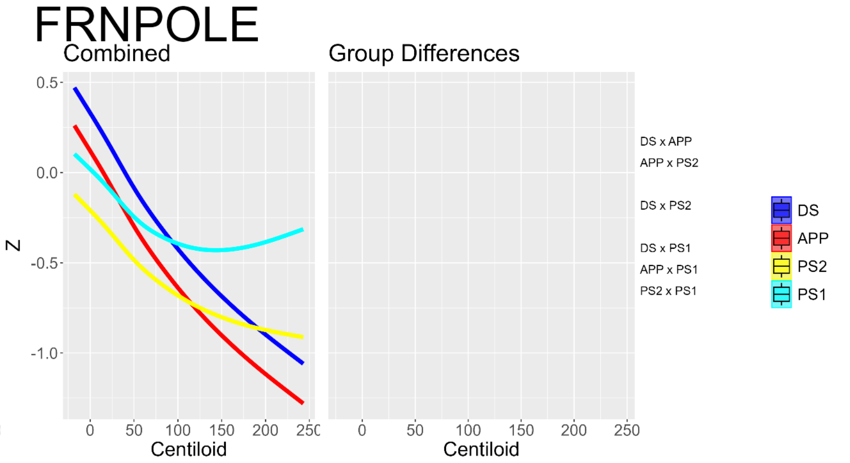
FRNPOLE
Combined
Group Differences
0.5
0.0
-0.5
-1.0
Z
0
50
100
150
200
250
Centiloid
0
50
100
150
200
250
Centiloid
DS x APP
APP x PS2
DS x PS2
DS x PS1
APP x PS1
PS2 x PS1
DS
APP
PS2
PS1

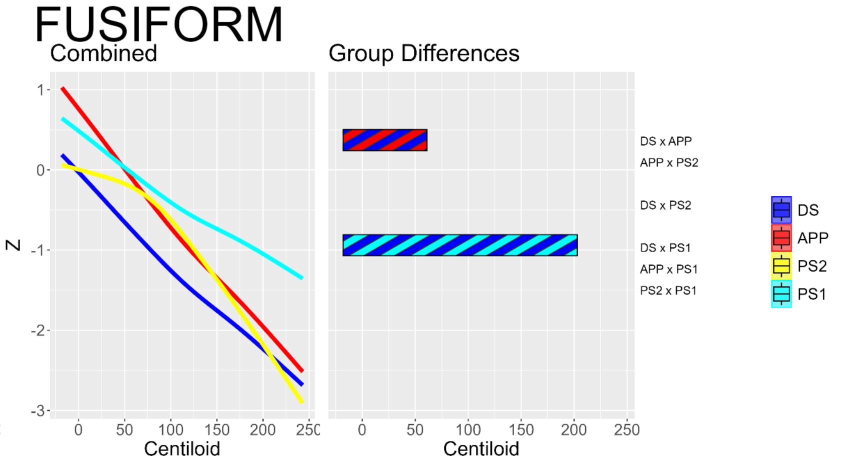
FUSIFORM
Combined
Group Differences
1
0
-1
-2
-3
Z
0
50
100
150
200
250
Centiloid
0
50
100
150
200
250
Centiloid
DS x APP
APP x PS2
DS x PS2
DS x PS1
APP x PS1
PS2 x PS1
DS
APP
PS2
PS1

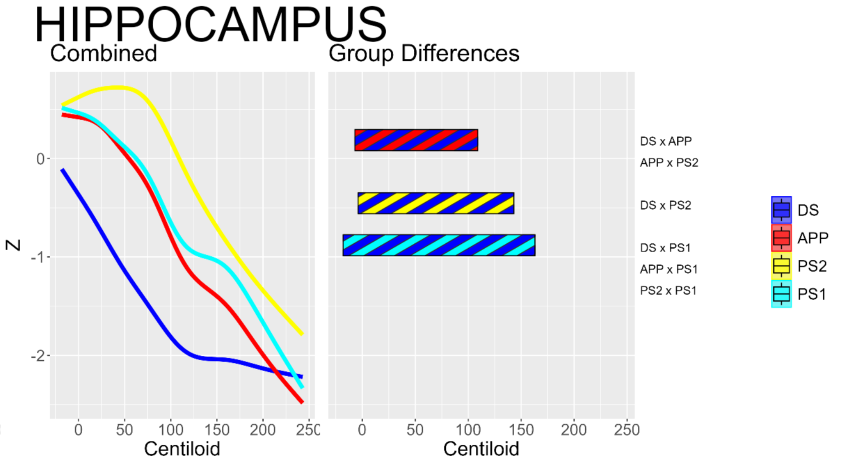
HIPPOCAMPUS
Combined
Group Differences
Z
0
-1
-2
0
50
100
150
200
250
Centiloid
DS x APP
APP x PS2
DS x PS2
DS x PS1
APP x PS1
PS2 x PS1
DS
APP
PS2
PS1

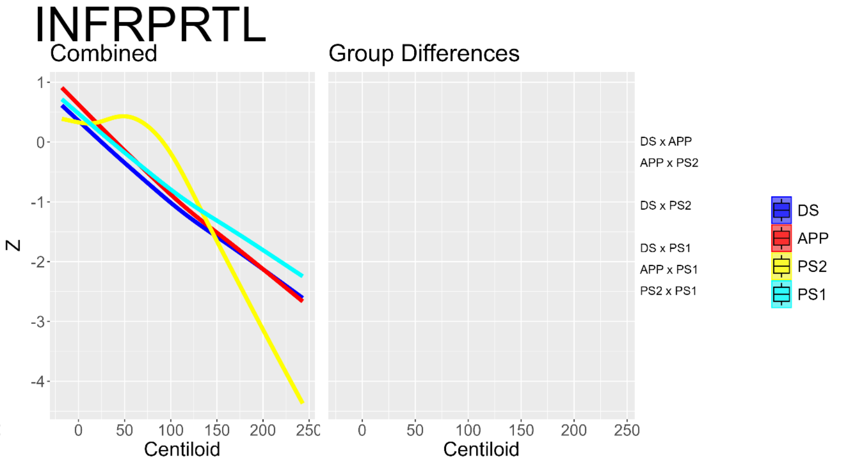

INFRPRTL
Combined
Group Differences
Z
1
0
-1
-2
-3
-4
0
50
100
150
200
250
Centiloid
DS x APP
APP x PS2
DS x PS2
DS x PS1
APP x PS1
PS2 x PS1
DS
APP
PS2
PS1

INFRTMP

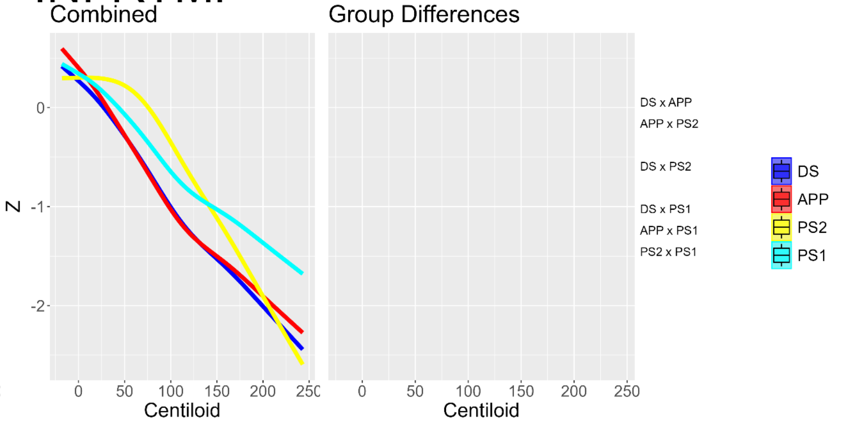

Combined
Group Differences
Z
0
-1
-2
0
50
100
150
200
250
Centiloid
0
50
100
150
200
250
Centiloid
DS x APP
APP x PS2
DS x PS2
DS x PS1
APP x PS1
PS2 x PS1
DS
APP
PS2
PS1

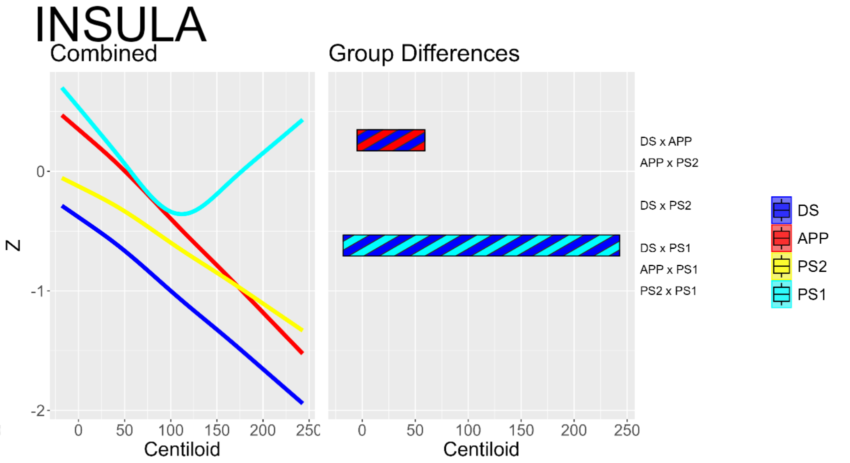

INSULA
Combined
Group Differences
0
-1
-2
Z
0
50
100
150
200
250
Centiloid
0
50
100
150
200
250
Centiloid
DS x APP
APP x PS2
DS x PS2
DS x PS1
APP x PS1
PS2 x PS1
DS
APP
PS2
PS1

ISTHMUSCNG

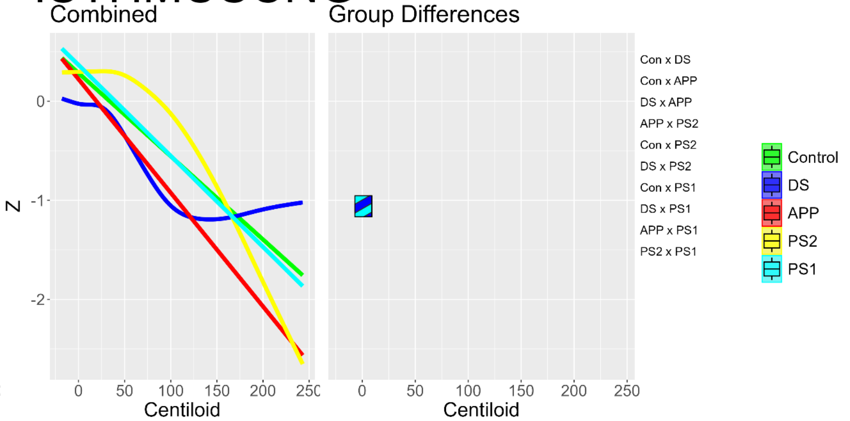

Combined
Group Differences
0
-1
-2
Z
0
50
100
150
200
250
Centiloid
Con x DS
Con x APP
DS x APP
APP x PS2
Con x PS2
DS x PS2
Con x PS1
DS x PS1
APP x PS1
PS2 x PS1
Control
DS
APP
PS2
PS1

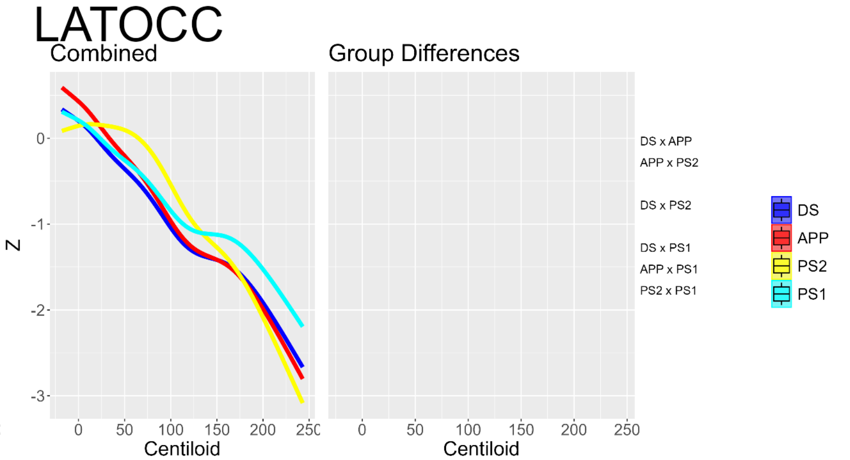

LATOCC
Combined
Group Differences
Z
0
-1
-2
-3
0
50
100
150
200
250
Centiloid
0
50
100
150
200
250
Centiloid
DS x APP
APP x PS2
DS x PS2
DS x PS1
APP x PS1
PS2 x PS1
DS
APP
PS2
PS1

LATORBFRN

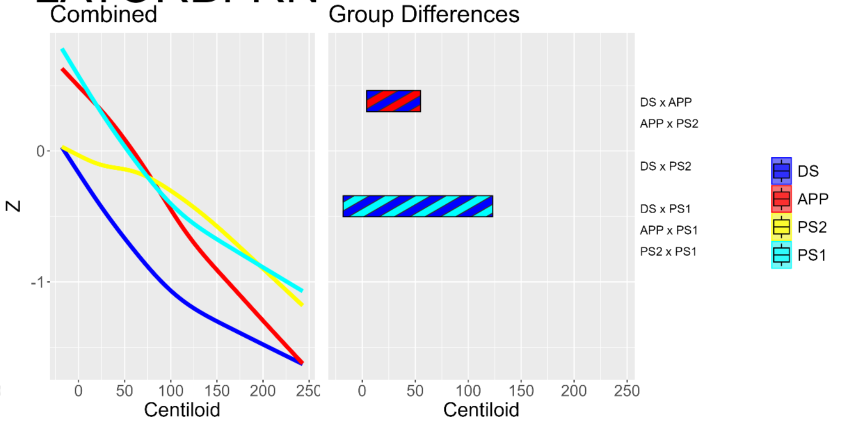

Combined
Group Differences
0
-1
Z
0
50
100
150
200
250
Centiloid
DS x APP
APP x PS2
DS x PS2
DS x PS1
APP x PS1
PS2 x PS1
DS
APP
PS2
PS1

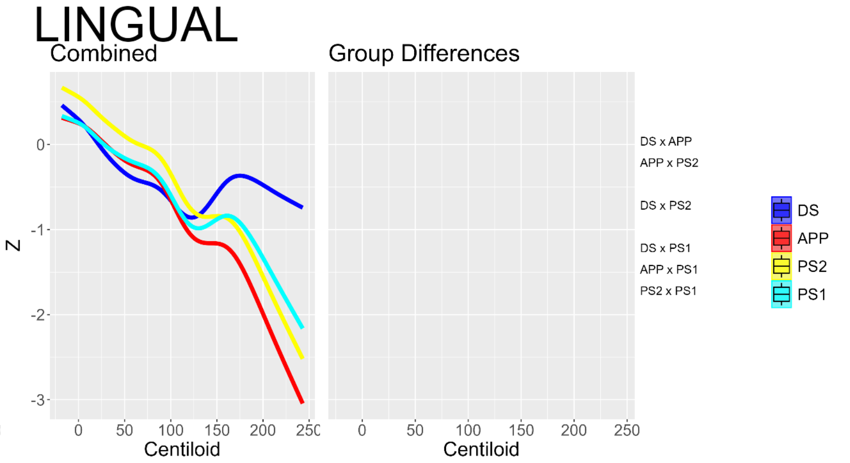
LINGUAL
Combined
Group Differences
0
-1
-2
-3
Z
0
50
100
150
200
250
Centiloid
0
50
100
150
200
250
Centiloid
DS x APP
APP x PS2
DS x PS2
DS x PS1
APP x PS1
PS2 x PS1
DS
APP
PS2
PS1

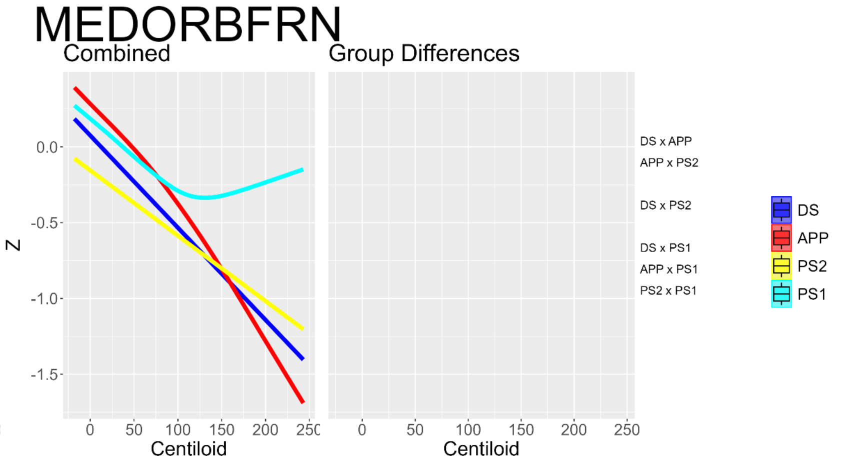

MEDORBFRN
Combined
Group Differences
0.0
-0.5
-1.0
-1.5
Z
0
50
100
150
200
250
Centiloid
0
50
100
150
200
250
Centiloid
DS x APP
APP x PS2
DS x PS2
DS x PS1
APP x PS1
PS2 x PS1
DS
APP
PS2
PS1

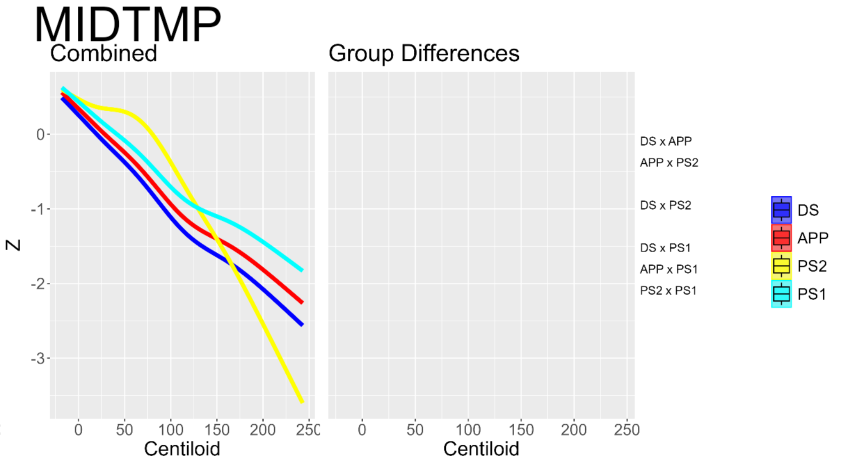
MIDTMP
Combined
Group Differences
Z
Centiloid
Centiloid
DS x APP
APP x PS2
DS x PS2
DS x PS1
APP x PS1
PS2 x PS1
DS
APP
PS2
PS1

PALLIDUM

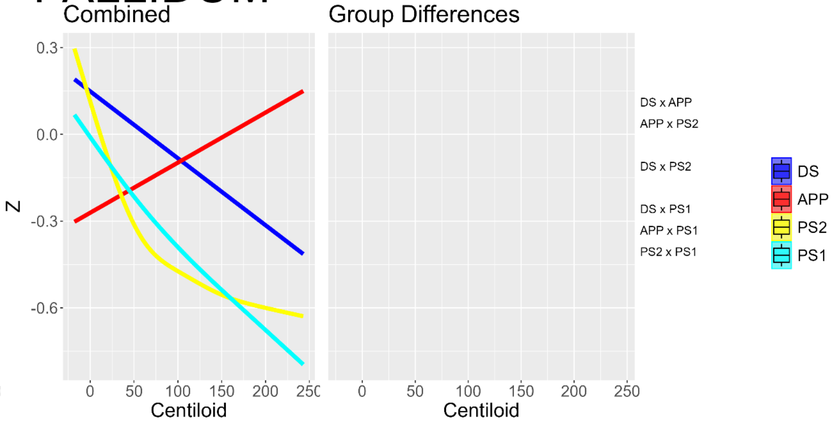
Combined
Group Differences
0.3
0.0
-0.3
-0.6
Z
0
50
100
150
200
250
Centiloid
0
50
100
150
200
250
Centiloid
DS x APP
APP x PS2
DS x PS2
DS x PS1
APP x PS1
PS2 x PS1
DS
APP
PS2
PS1

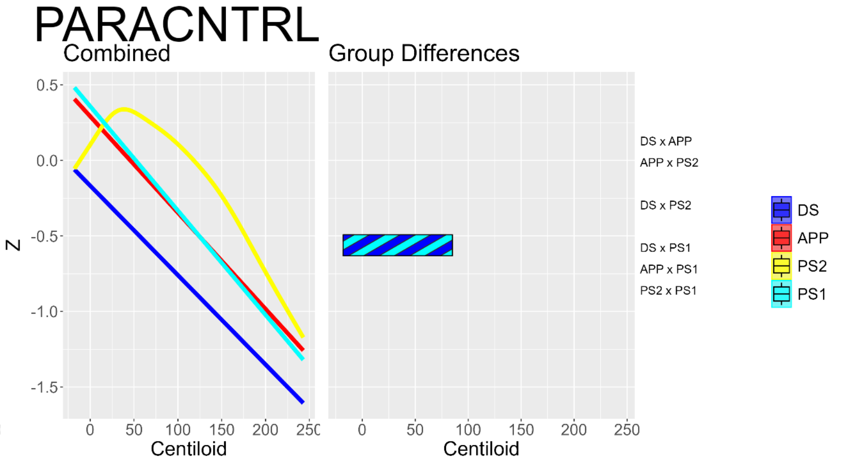

PARACNTRL
Combined
Group Differences
Z
0.5
0.0
-0.5
-1.0
-1.5
0
50
100
150
200
250
Centiloid
DS x APP
APP x PS2
DS x PS2
DS x PS1
APP x PS1
PS2 x PS1
DS
APP
PS2
PS1

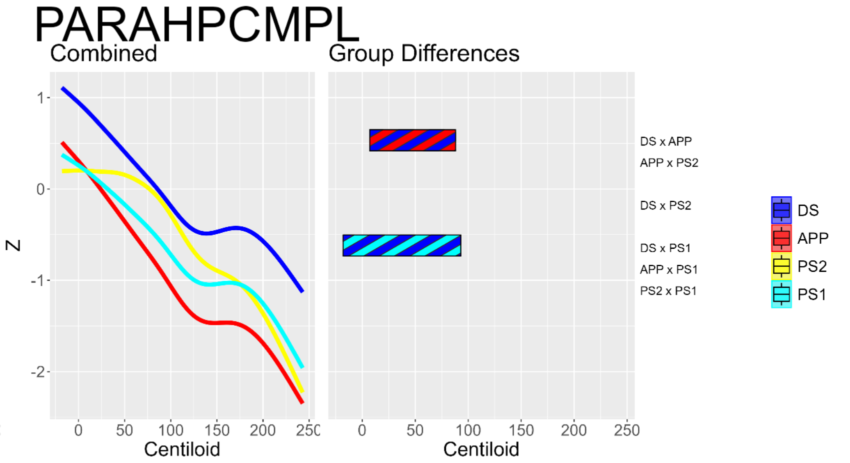
PARAHPCMPL
Combined
Group Differences
1
0
-1
-2
Z
0
50
100
150
200
250
Centiloid
0
50
100
150
200
250
Centiloid
DS x APP
APP x PS2
DS x PS2
DS x PS1
APP x PS1
PS2 x PS1
DS
APP
PS2
PS1

PARAOPRCLRS

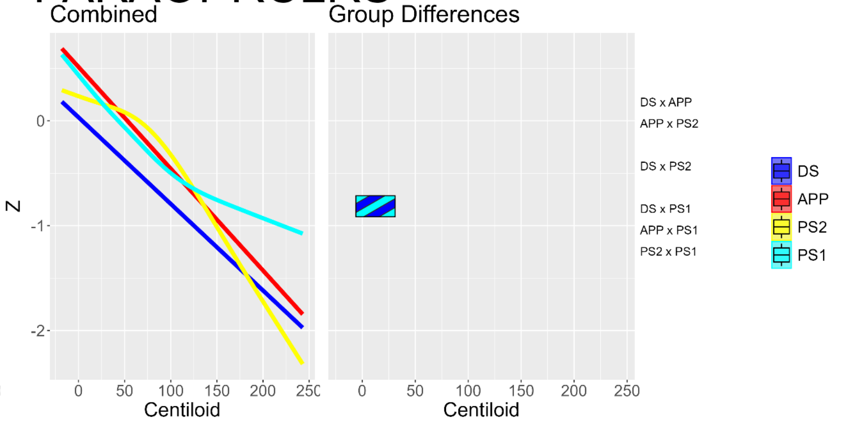

PARSORBLS

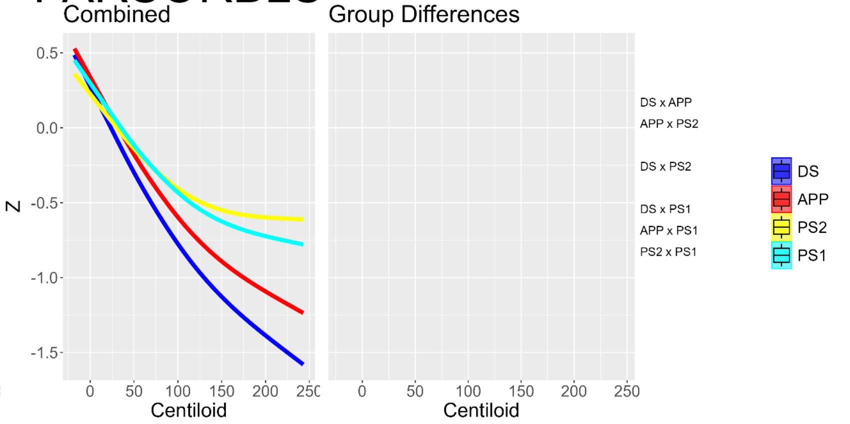

Combined
Group Differences
0.5
0.0
-0.5
-1.0
-1.5
Z
0
50
100
150
200
250
Centiloid
DS x APP
APP x PS2
DS x PS2
DS x PS1
APP x PS1
PS2 x PS1
DS
APP
PS2
PS1

PARSTRNGLRS

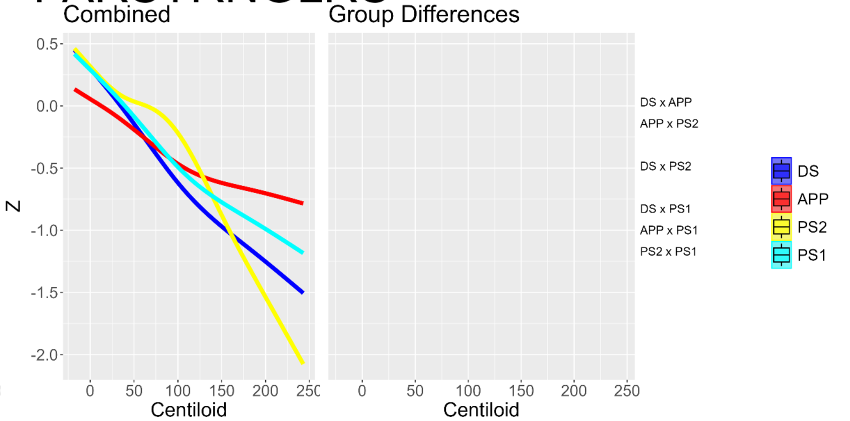

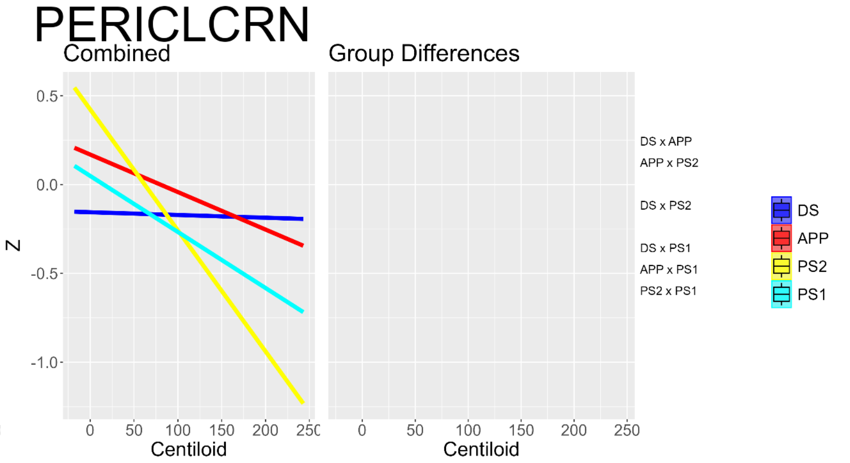
PERICLCRN
Combined
Group Differences
0.5
0.0
-0.5
-1.0
Z
0
50
100
150
200
250
Centiloid
0
50
100
150
200
250
Centiloid
DS x APP
APP x PS2
DS x PS2
DS x PS1
APP x PS1
PS2 x PS1
DS
APP
PS2
PS1

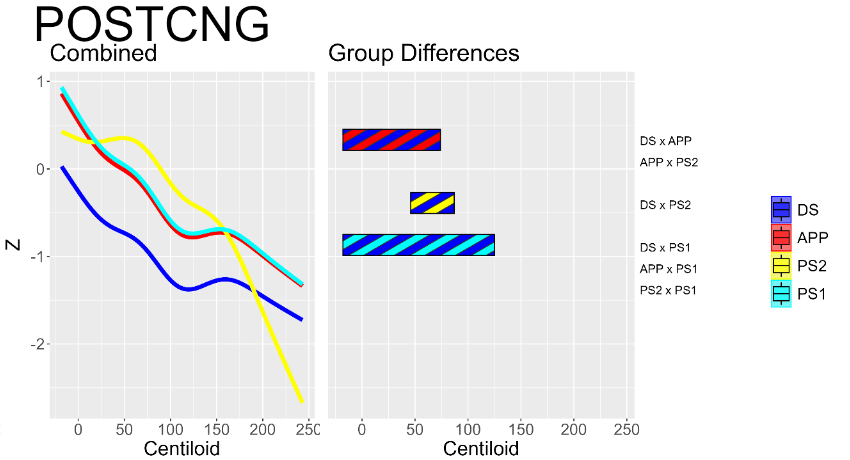
POSTCNG
Combined
Group Differences
1
0
-1
-2
Z
0
50
100
150
200
250
Centiloid
0
50
100
150
200
250
Centiloid
DS x APP
APP x PS2
DS x PS2
DS x PS1
APP x PS1
PS2 x PS1
DS
APP
PS2
PS1

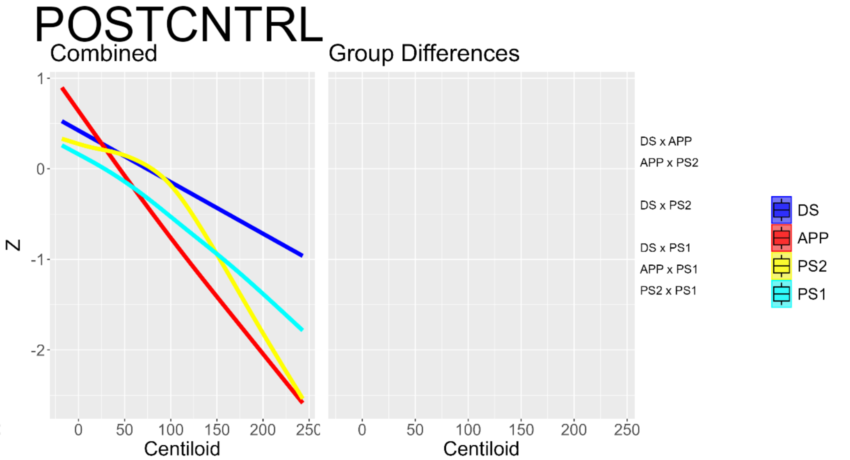
POSTCNTRL
Combined
Group Differences
Z
1
0
-1
-2
0
50
100
150
200
250
Centiloid
0
50
100
150
200
250
Centiloid
DS x APP
APP x PS2
DS x PS2
DS x PS1
APP x PS1
PS2 x PS1
DS
APP
PS2
PS1

PRECNTRL

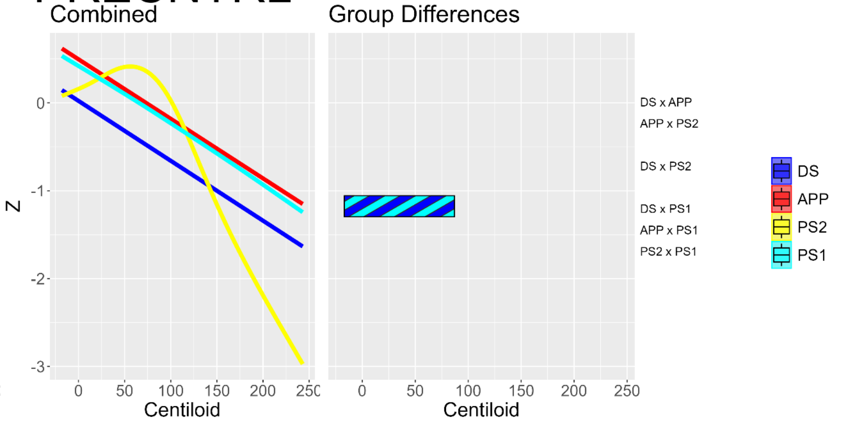

Combined
Group Differences
0
-1
-2
-3
Z
0
50
100
150
200
250
Centiloid
DS x APP
APP x PS2
DS x PS2
DS x PS1
APP x PS1
PS2 x PS1
DS
APP
PS2
PS1

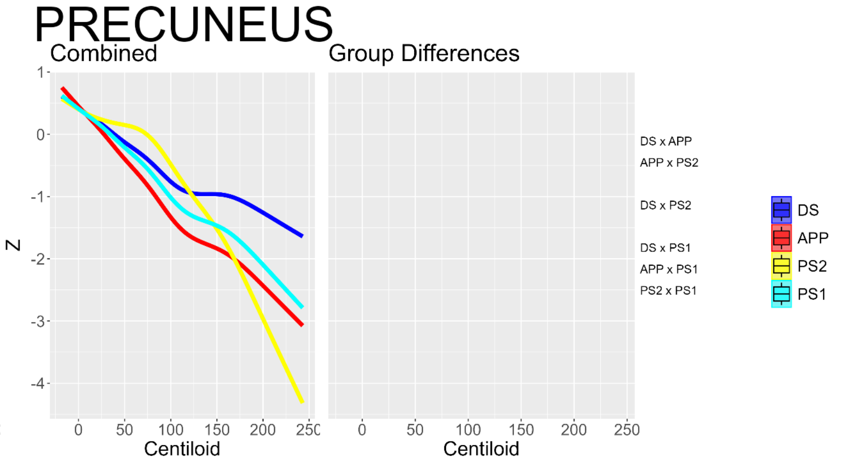
PRECUNEUS
Combined
Group Differences
Z
1
0
-1
-2
-3
-4
0
50
100
150
200
250
Centiloid
DS x APP
APP x PS2
DS x PS2
DS x PS1
APP x PS1
PS2 x PS1
DS
APP
PS2
PS1

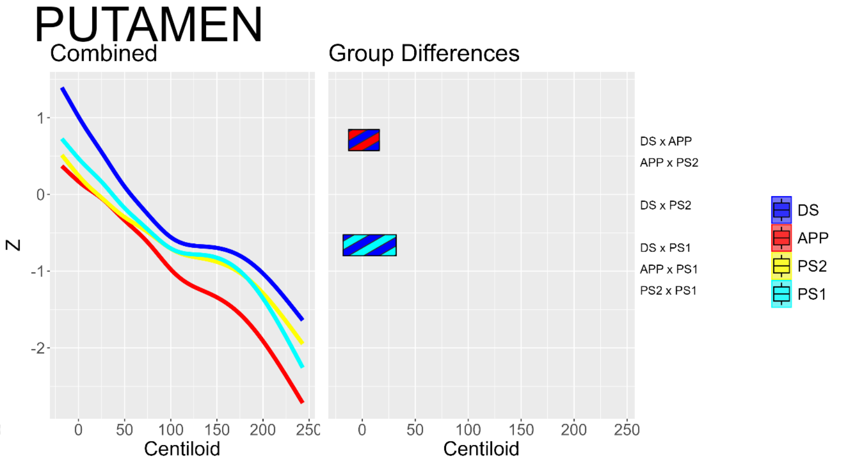
PUTAMEN
Combined
Group Differences
Z
1
0
-1
-2
0
50
100
150
200
250
Centiloid
0
50
100
150
200
250
Centiloid
DS x APP
APP x PS2
DS x PS2
DS x PS1
APP x PS1
PS2 x PS1
DS
APP
PS2
PS1

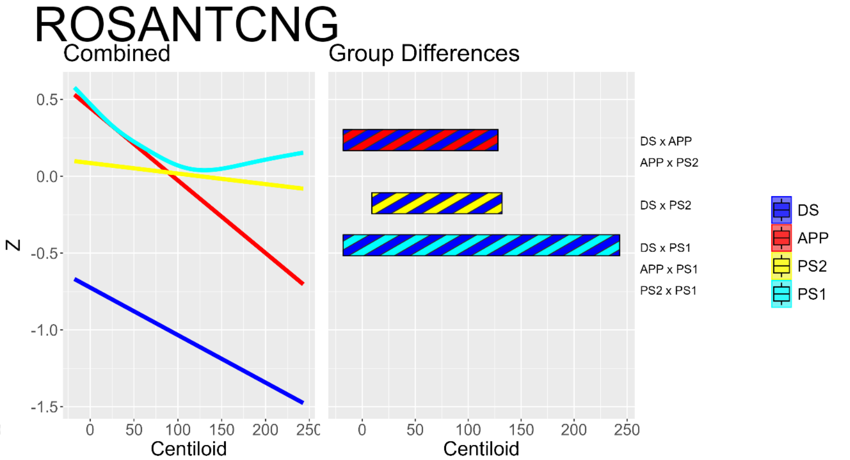
ROSANTCNG
Combined
Group Differences
0.5
0.0
-0.5
-1.0
-1.5
Z
0
50
100
150
200
250
Centiloid
DS x APP
APP x PS2
DS x PS2
DS x PS1
APP x PS1
PS2 x PS1
DS
APP
PS2
PS1

ROSMIDFRN

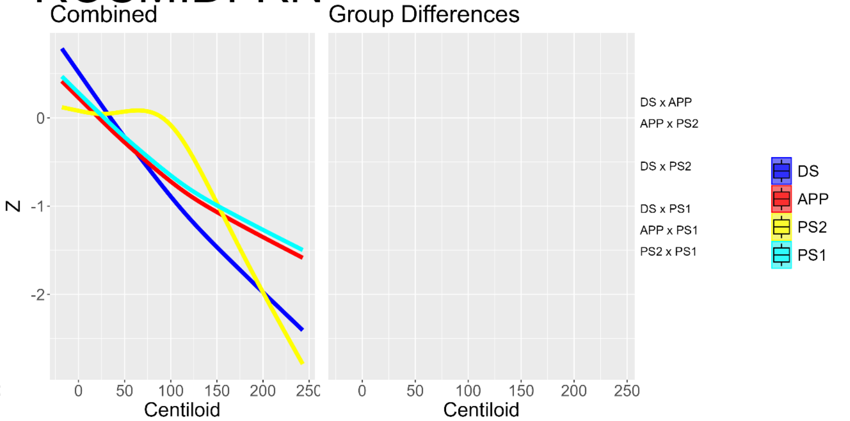

Combined
Group Differences
Z
0
-1
-2
0
50
100
150
200
250
Centiloid
DS x APP
APP x PS2
DS x PS2
DS x PS1
APP x PS1
PS2 x PS1
DS
APP
PS2
PS1

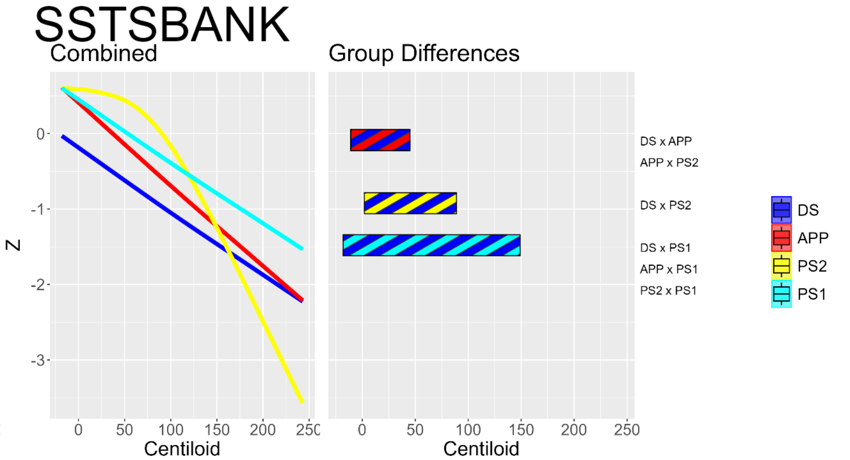
SSTSBANK
Combined
Group Differences
0
-1
-2
-3
Z
0
50
100
150
200
250
Centiloid
0
50
100
150
200
250
Centiloid
DS x APP
APP x PS2
DS x PS2
DS x PS1
APP x PS1
PS2 x PS1
DS
APP
PS2
PS1

SUPERFRN

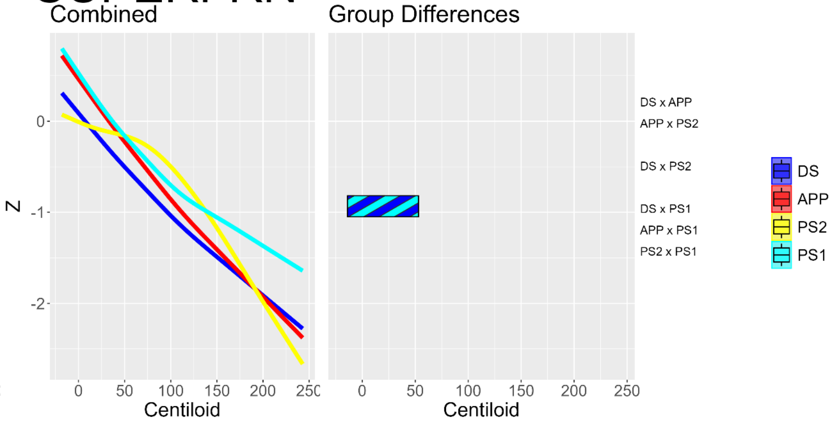

Combined
Group Differences
0
-1
-2
Z
0
50
100
150
200
250
Centiloid
DS x APP
APP x PS2
DS x PS2
DS x PS1
APP x PS1
PS2 x PS1
DS
APP
PS2
PS1

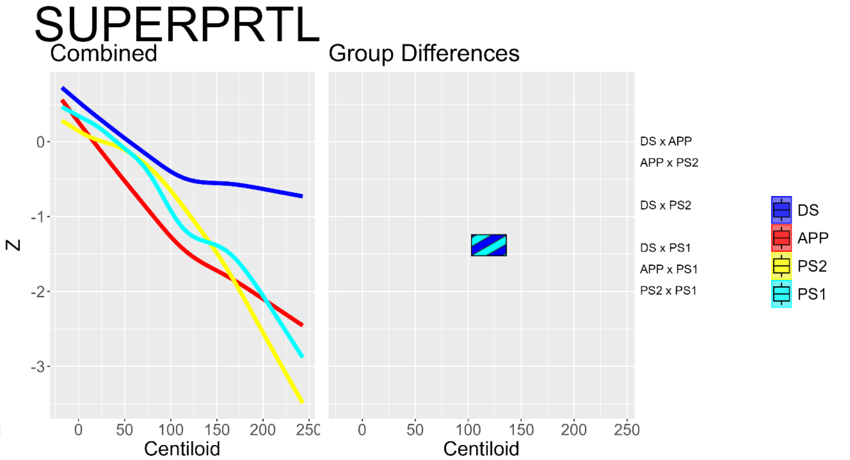
SUPERPRTL
Combined
Group Differences
Z
0
-1
-2
-3
0
50
100
150
200
250
Centiloid
0
50
100
150
200
250
Centiloid
DS x APP
APP x PS2
DS x PS2
DS x PS1
APP x PS1
PS2 x PS1
DS
APP
PS2
PS1

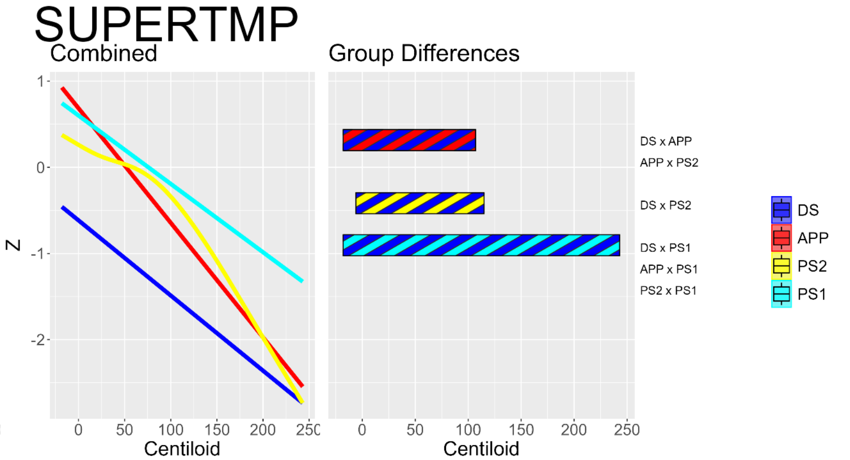
SUPERTMP
Combined
Group Differences
Z
1
0
-1
-2
0
50
100
150
200
250
Centiloid
DS x APP
APP x PS2
DS x PS2
DS x PS1
APP x PS1
PS2 x PS1
DS
APP
PS2
PS1

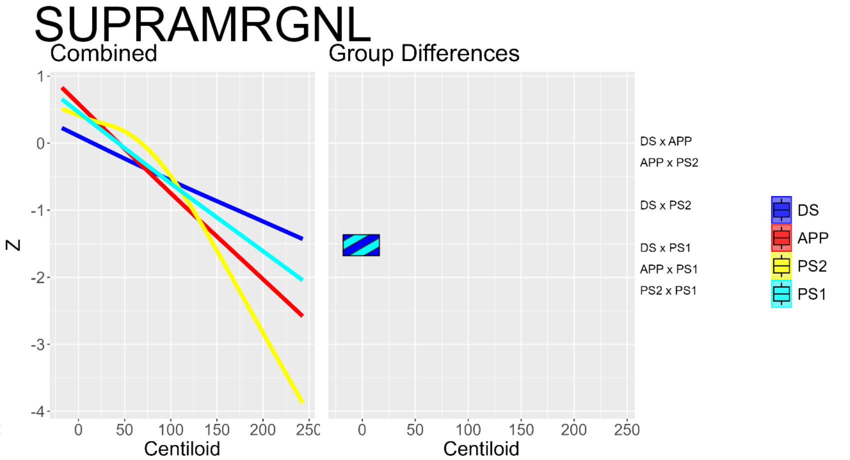
SUPRAMRGNL
Combined
Group Differences
Z
Centiloid
Centiloid
DS x APP
APP x PS2
DS x PS2
DS x PS1
APP x PS1
PS2 x PS1
DS
APP
PS2
PS1

THALAMUS

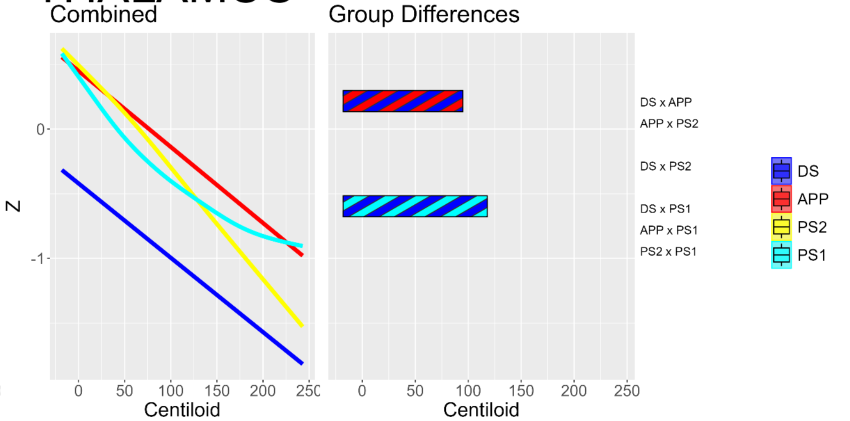
Combined
Group Differences
Z
0
-1
0
50
100
150
200
250
Centiloid
0
50
100
150
200
250
Centiloid
DS x APP
APP x PS2
DS x PS2
DS x PS1
APP x PS1
PS2 x PS1
DS
APP
PS2
PS1

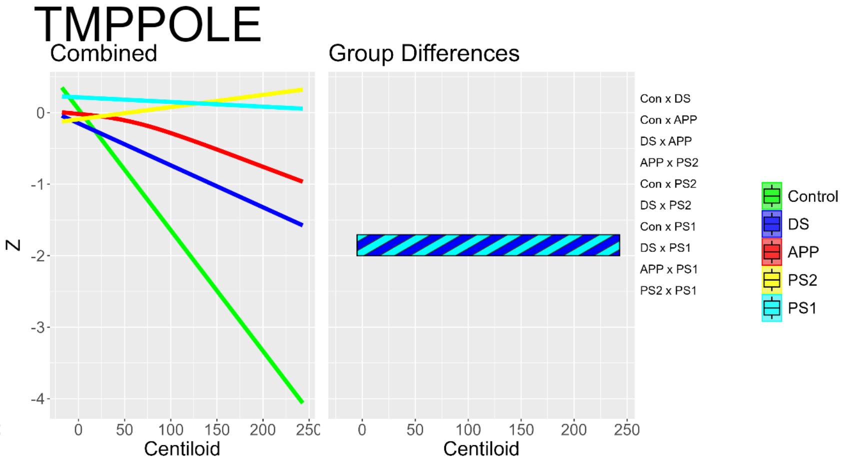

TMPPOLE
Combined
Group Differences
Z
0
-1
-2
-3
-4
0
50
100
150
200
250
Centiloid
Con x DS
Con x APP
DS x APP
APP x PS2
Con x PS2
DS x PS2
Con x PS1
DS x PS1
APP x PS1
PS2 x PS1
Control
DS
APP
PS2
PS1

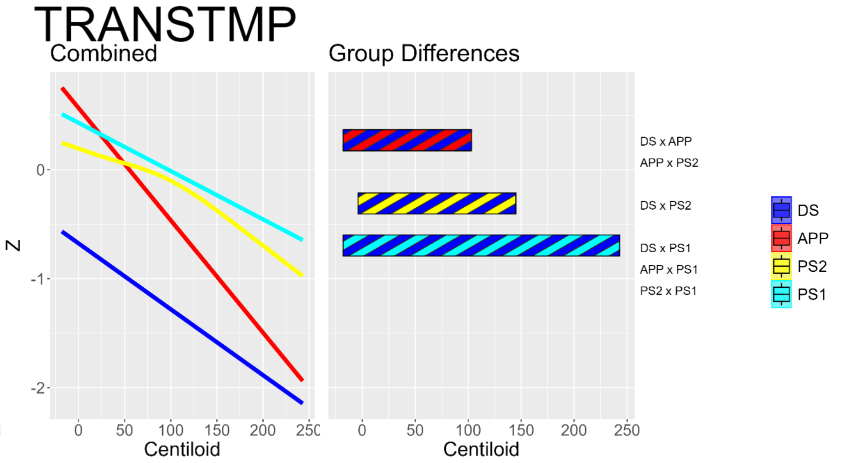

TRANSTMP
Combined
Group Differences
Z
0
-1
-2
0
50
100
150
200
250
Centiloid
0
50
100
150
200
250
Centiloid
DS x APP
APP x PS2
DS x PS2
DS x PS1
APP x PS1
PS2 x PS1
DS
APP
PS2
PS1

VENTRALDC

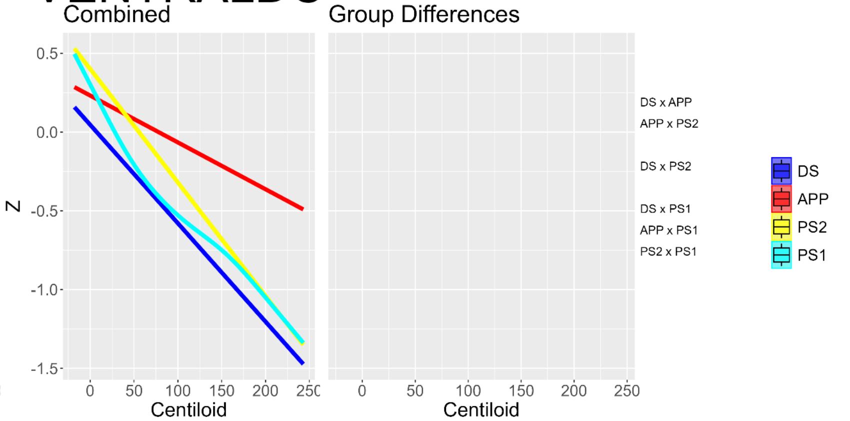

Combined
Group Differences
0.5
0.0
-0.5
-1.0
-1.5
Z
0
50
100
150
200
250
Centiloid
0
50
100
150
200
250
Centiloid
DS x APP
APP x PS2
DS x PS2
DS x PS1
APP x PS1
PS2 x PS1
DS
APP
PS2
PS1
