# Supplementary material for: Brain volume trajectories in Down syndrome and autosomal dominant Alzheimer's disease
Source: Alzheimers Dement. 2026 Jan 18;22(1):e71103. doi: 10.1002/alz.71103 (PMC12812856; doi:10.1002/alz.71103)
Supplement: Supplementary file 11 — Supporting information [file ALZ-22-e71103-s014.pdf]

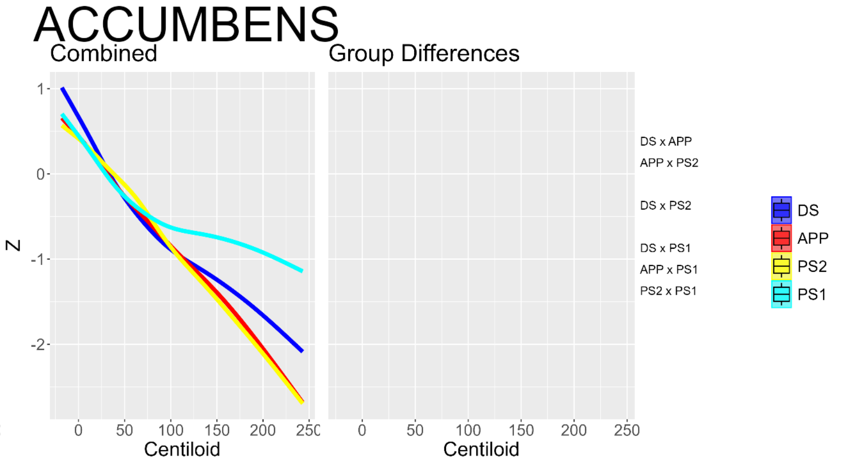

ACCUMBENS
Combined
Group Differences
Z
1
0
-1
-2
0
50
100
150
200
250
Centiloid
0
50
100
150
200
250
Centiloid
DS x APP
APP x PS2
DS x PS2
DS x PS1
APP x PS1
PS2 x PS1
DS
APP
PS2
PS1

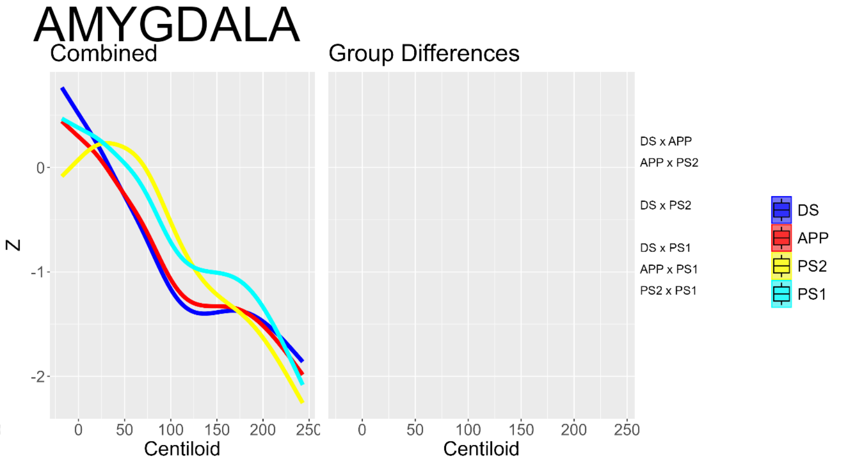
AMYGDALA
Combined
Group Differences
Z
0
-1
-2
0
50
100
150
200
250
Centiloid
0
50
100
150
200
250
Centiloid
DS x APP
APP x PS2
DS x PS2
DS x PS1
APP x PS1
PS2 x PS1
DS
APP
PS2
PS1

BRAINSTEM

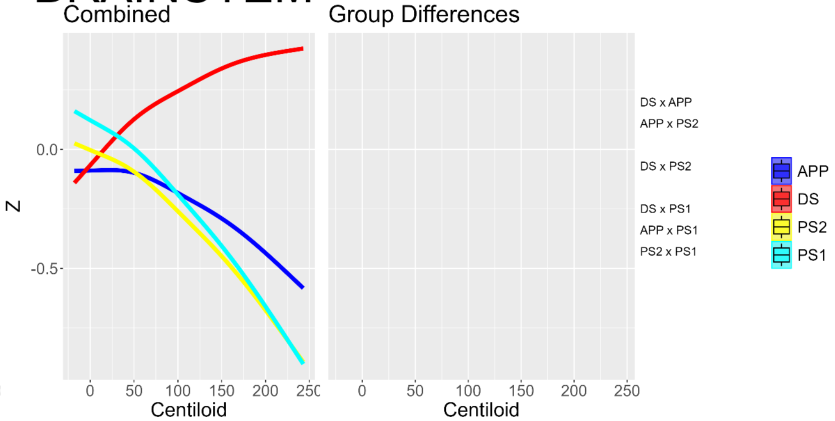

Combined
Group Differences
0.0
-0.5
Z
0
50
100
150
200
250
Centiloid
0
50
100
150
200
250
Centiloid
DS x APP
APP x PS2
DS x PS2
DS x PS1
APP x PS1
PS2 x PS1
APP
DS
PS2
PS1

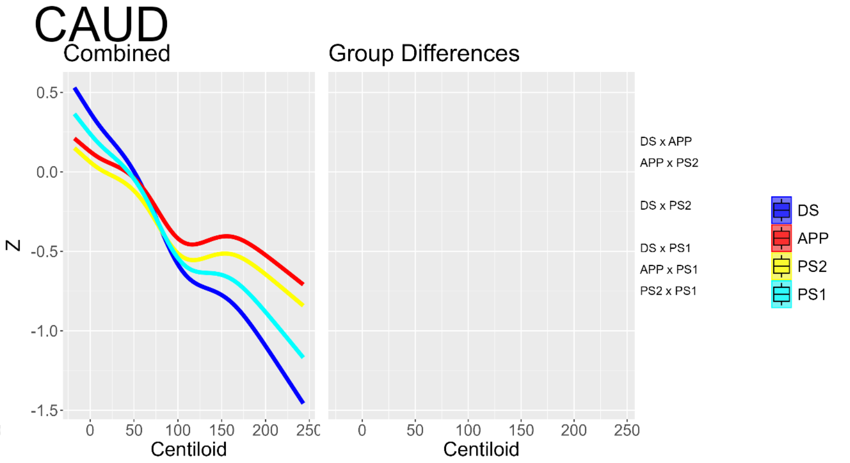

CAUD
Combined
Group Differences
0.5
0.0
-0.5
-1.0
-1.5
Z
0
50
100
150
200
250
Centiloid
DS x APP
APP x PS2
DS x PS2
DS x PS1
APP x PS1
PS2 x PS1
DS
APP
PS2
PS1

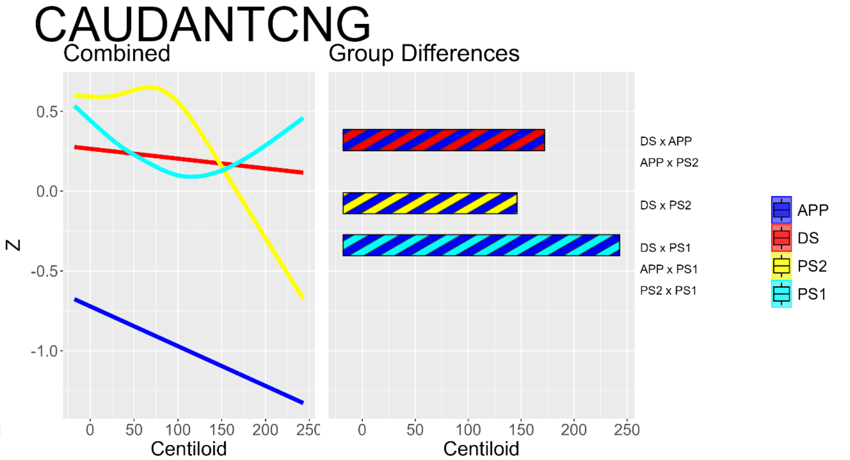

CAUDANTCNG
Combined
Group Differences
0.5
0.0
-0.5
-1.0
Z
0
50
100
150
200
250
Centiloid
0
50
100
150
200
250
Centiloid
DS x APP
APP x PS2
DS x PS2
DS x PS1
APP x PS1
PS2 x PS1
APP
DS
PS2
PS1

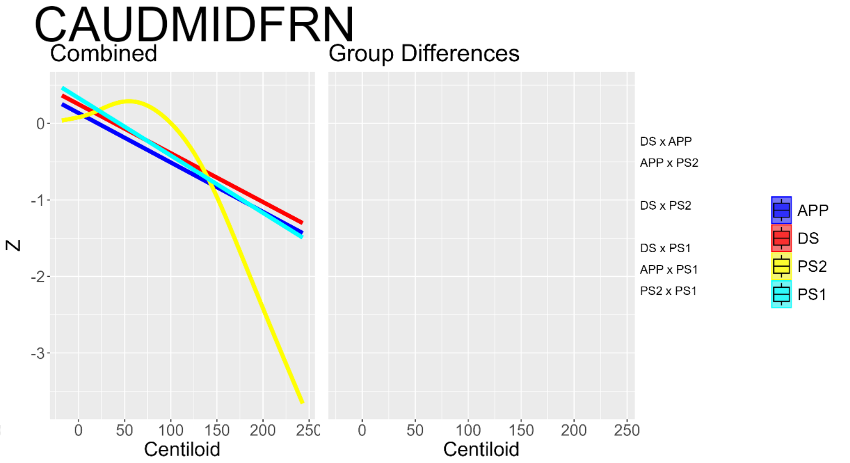
CAUDMIDFRN
Combined
Group Differences
Z
0
-1
-2
-3
0
50
100
150
200
250
Centiloid
0
50
100
150
200
250
Centiloid
DS x APP
APP x PS2
DS x PS2
DS x PS1
APP x PS1
PS2 x PS1
APP
DS
PS2
PS1

CBLLCORTEX

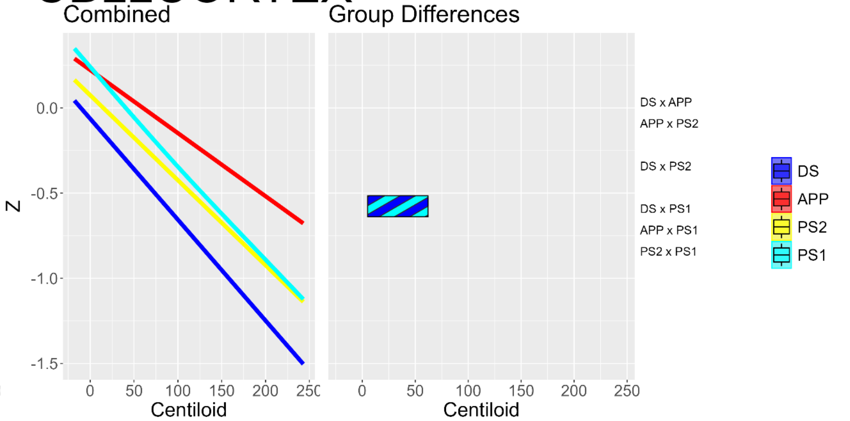

Combined
Group Differences
Z
0.0
-0.5
-1.0
-1.5
0
50
100
150
200
250
Centiloid
DS x APP
APP x PS2
DS x PS2
DS x PS1
APP x PS1
PS2 x PS1
DS
APP
PS2
PS1

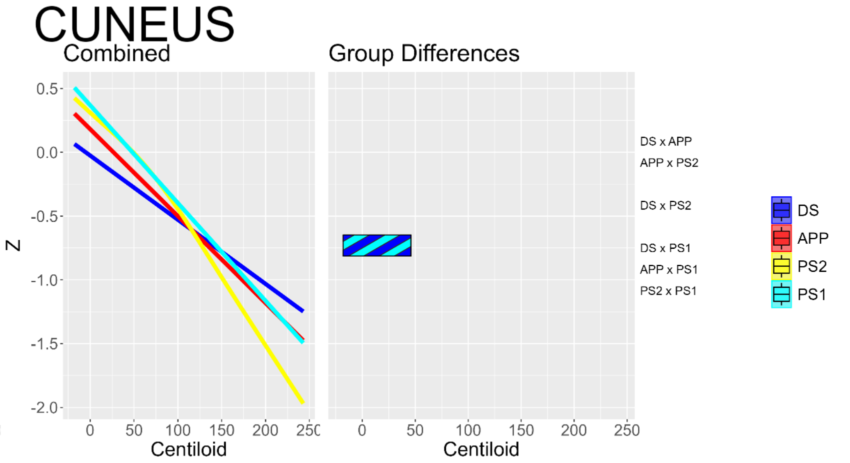
CUNEUS
Combined
Group Differences
0.5
0.0
-0.5
-1.0
-1.5
-2.0
Z
0
50
100
150
200
250
Centiloid
DS x APP
APP x PS2
DS x PS2
DS x PS1
APP x PS1
PS2 x PS1
DS
APP
PS2
PS1

ENTORHINAL

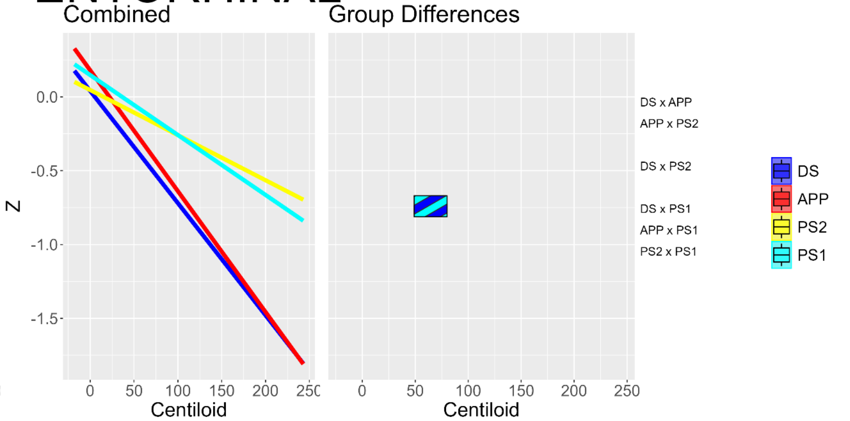
Combined
Group Differences
0.0
-0.5
-1.0
-1.5
Z
0
50
100
150
200
250
Centiloid
DS x APP
APP x PS2
DS x PS2
DS x PS1
APP x PS1
PS2 x PS1
DS
APP
PS2
PS1

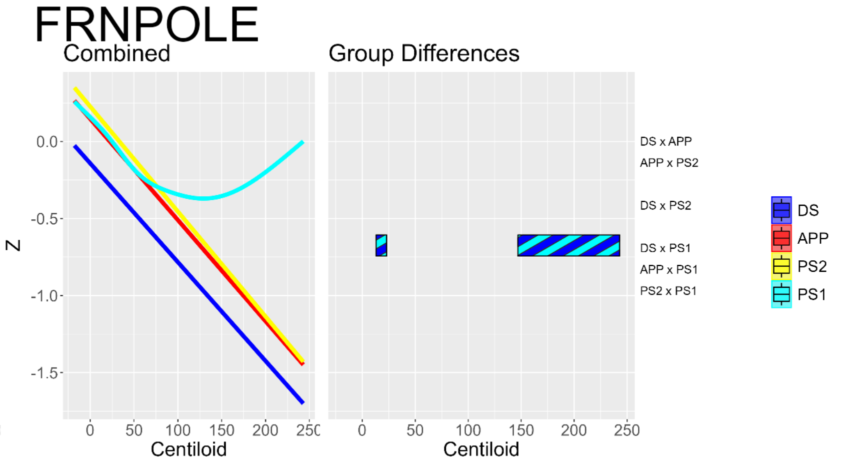
FRNPOLE
Combined
Group Differences
0.0
-0.5
-1.0
-1.5
Z
0
50
100
150
200
250
Centiloid
0
50
100
150
200
250
Centiloid
DS x APP
APP x PS2
DS x PS2
DS x PS1
APP x PS1
PS2 x PS1
DS
APP
PS2
PS1

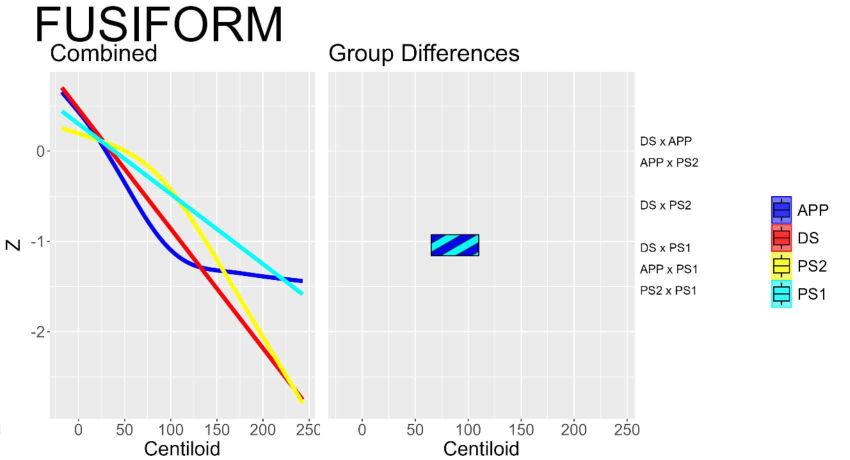
FUSIFORM
Combined
Group Differences
Z
0
-1
-2
0
50
100
150
200
250
Centiloid
DS x APP
APP x PS2
DS x PS2
DS x PS1
APP x PS1
PS2 x PS1
APP
DS
PS2
PS1

HIPPOCAMPUS

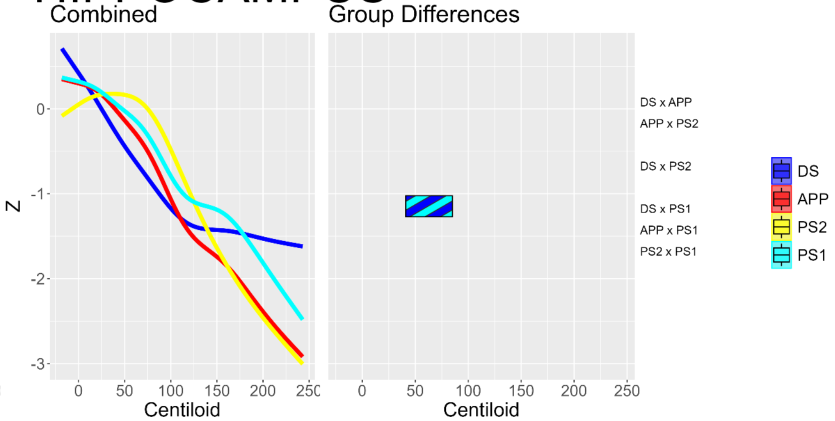

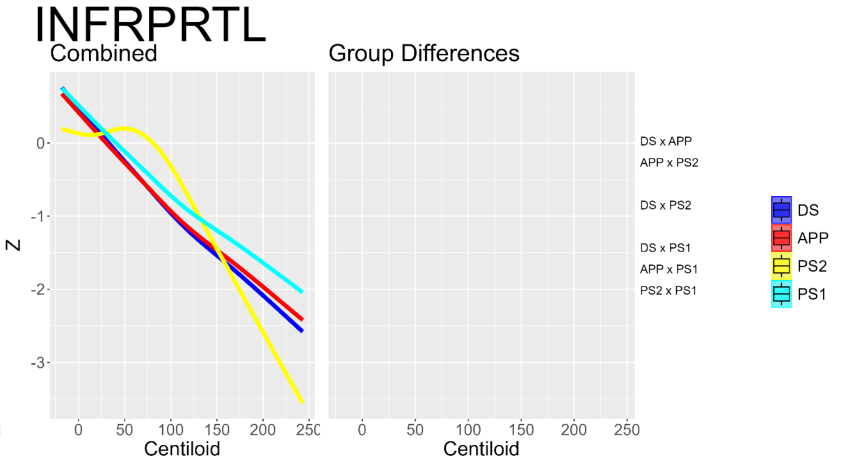

INFRPRTL
Combined
Group Differences
Z
0
-1
-2
-3
0
50
100
150
200
250
Centiloid
0
50
100
150
200
250
Centiloid
DS x APP
APP x PS2
DS x PS2
DS x PS1
APP x PS1
PS2 x PS1
DS
APP
PS2
PS1

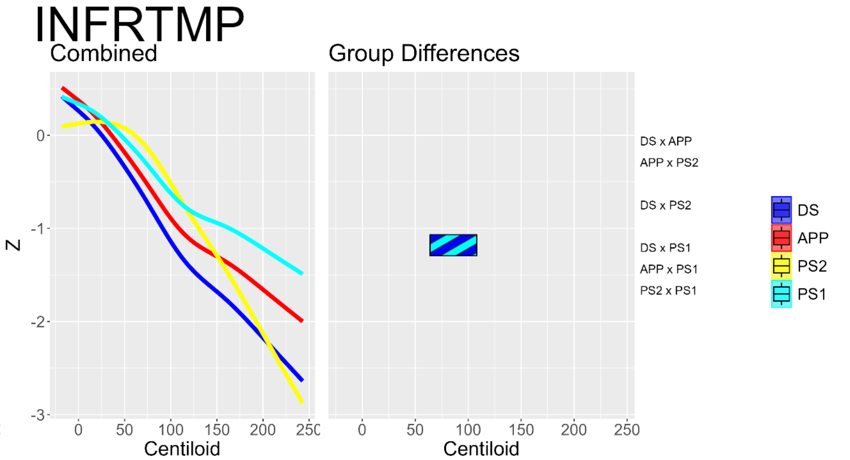
INFRTMP
Combined
Group Differences
Z
0
-1
-2
-3
0
50
100
150
200
250
Centiloid
DS x APP
APP x PS2
DS x PS2
DS x PS1
APP x PS1
PS2 x PS1
DS
APP
PS2
PS1

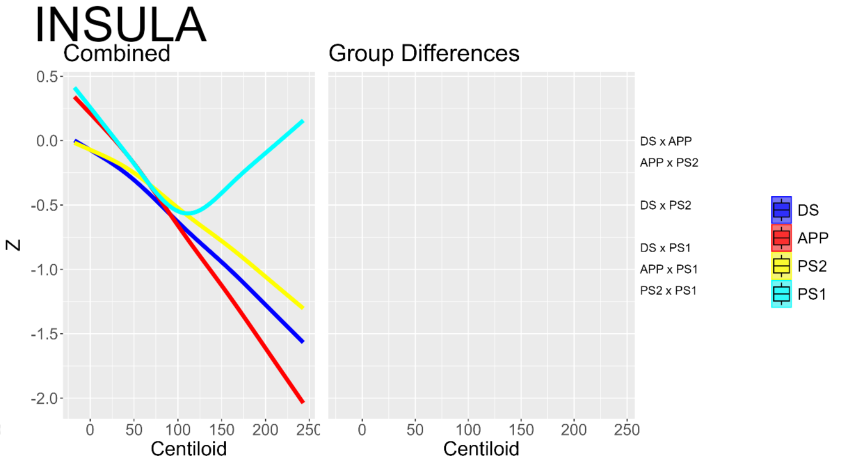
INSULA
Combined
Group Differences
0.5
0.0
-0.5
-1.0
-1.5
-2.0
Z
0
50
100
150
200
250
Centiloid
0
50
100
150
200
250
Centiloid
DS x APP
APP x PS2
DS x PS2
DS x PS1
APP x PS1
PS2 x PS1
DS
APP
PS2
PS1

ISTHMUSCNG

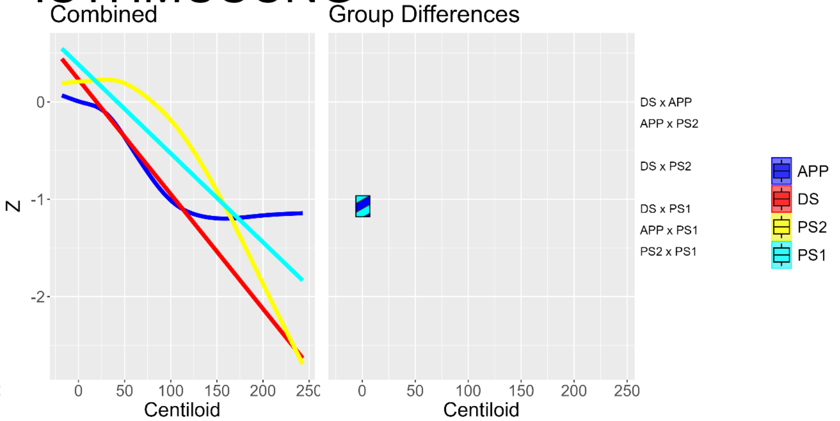
Combined
Group Differences
0
-1
-2
Z
0
50
100
150
200
250
Centiloid
0
50
100
150
200
250
Centiloid
DS x APP
APP x PS2
DS x PS2
DS x PS1
APP x PS1
PS2 x PS1
APP
DS
PS2
PS1

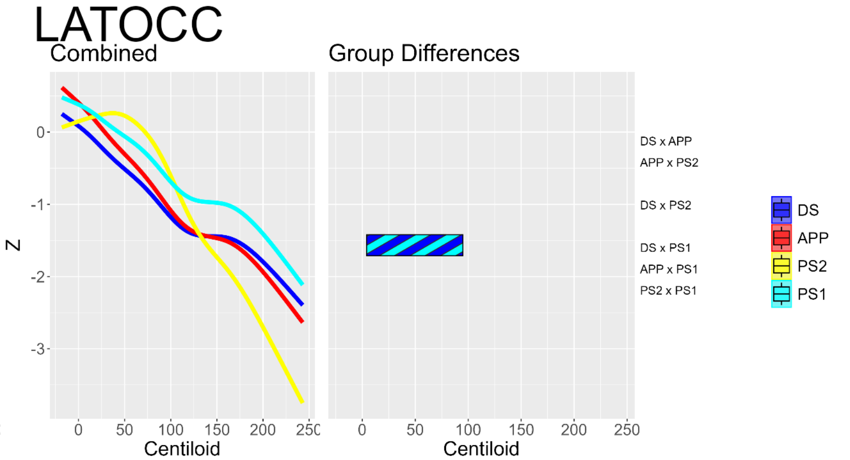
LATOCC
Combined
Group Differences
Z
0
-1
-2
-3
0
50
100
150
200
250
Centiloid
0
50
100
150
200
250
Centiloid
DS x APP
APP x PS2
DS x PS2
DS x PS1
APP x PS1
PS2 x PS1
DS
APP
PS2
PS1

LATORBFRN

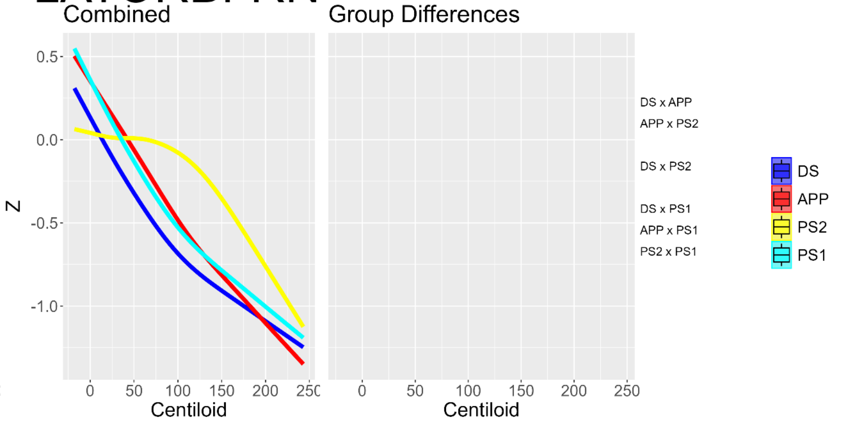

Combined
Group Differences
0.5
0.0
-0.5
-1.0
Z
0
50
100
150
200
250
Centiloid
0
50
100
150
200
250
Centiloid
DS x APP
APP x PS2
DS x PS2
DS x PS1
APP x PS1
PS2 x PS1
DS
APP
PS2
PS1

LINGUAL

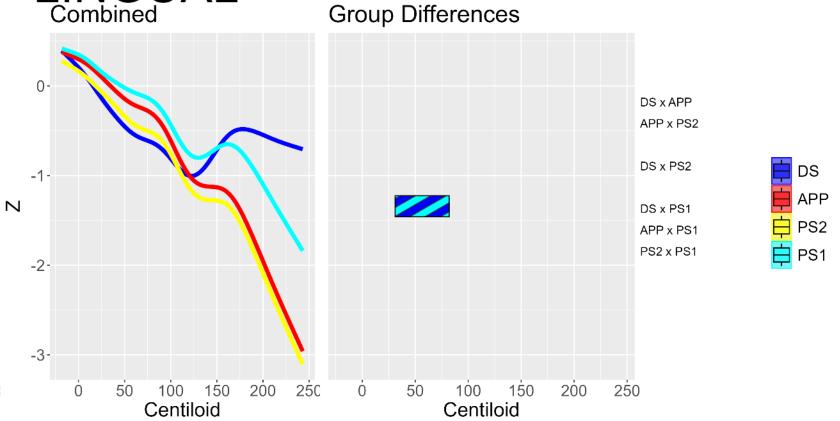
Combined
Group Differences
0
-1
-2
-3
Z
0
50
100
150
200
250
Centiloid
0
50
100
150
200
250
Centiloid
DS x APP
APP x PS2
DS x PS2
DS x PS1
APP x PS1
PS2 x PS1
DS
APP
PS2
PS1

MEDORBFRN

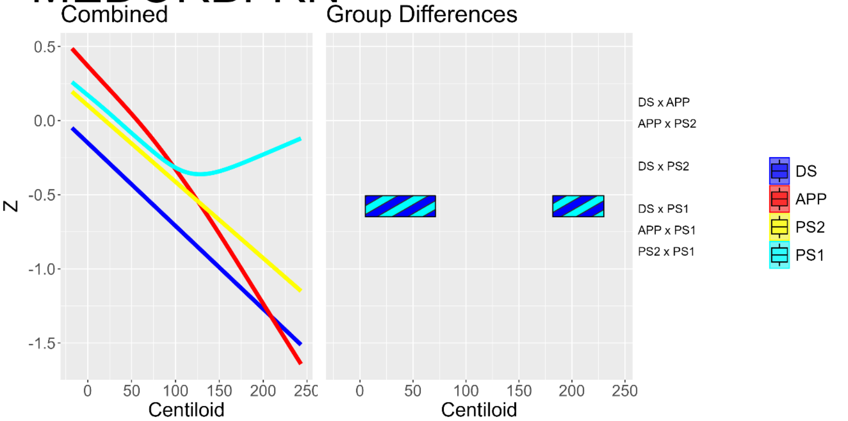

Combined
Group Differences
0.5
0.0
-0.5
-1.0
-1.5
Z
0
50
100
150
200
250
Centiloid
0
50
100
150
200
250
Centiloid
DS x APP
APP x PS2
DS x PS2
DS x PS1
APP x PS1
PS2 x PS1
DS
APP
PS2
PS1

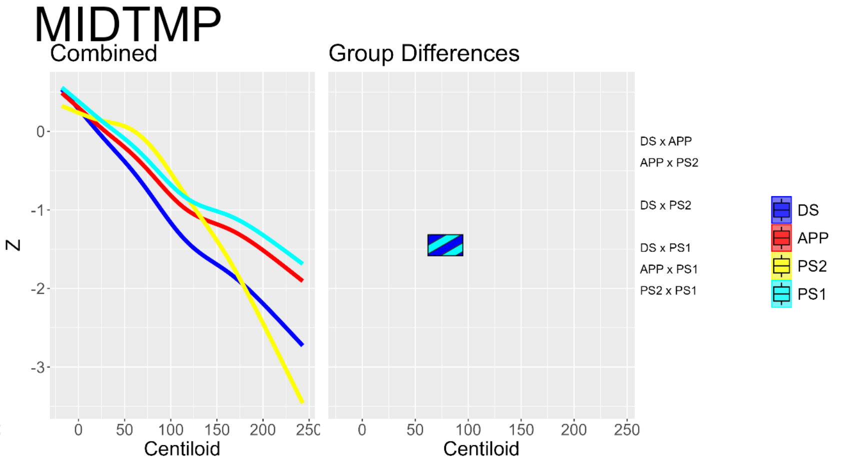
MIDTMP
Combined
Group Differences
0
-1
-2
-3
Z
0
50
100
150
200
250
Centiloid
0
50
100
150
200
250
Centiloid
DS x APP
APP x PS2
DS x PS2
DS x PS1
APP x PS1
PS2 x PS1
DS
APP
PS2
PS1

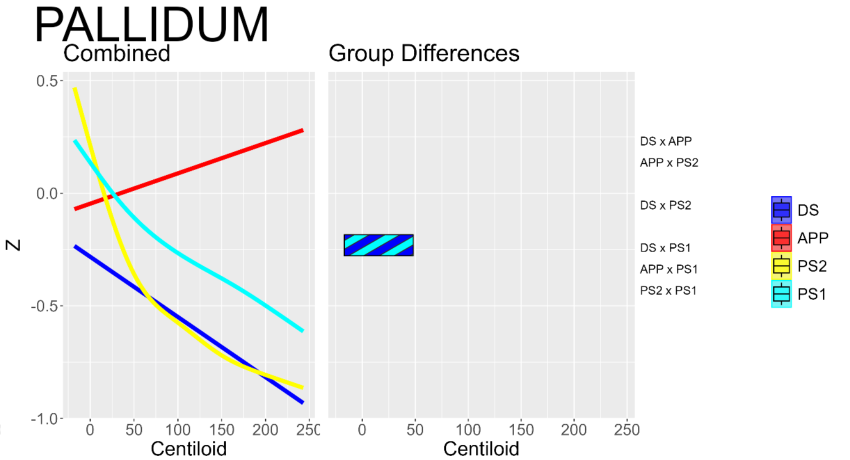
PALLIDUM
Combined
Group Differences
0.5
0.0
-0.5
-1.0
Z
0
50
100
150
200
250
Centiloid
DS x APP
APP x PS2
DS x PS2
DS x PS1
APP x PS1
PS2 x PS1
DS
APP
PS2
PS1

PARACNTRL

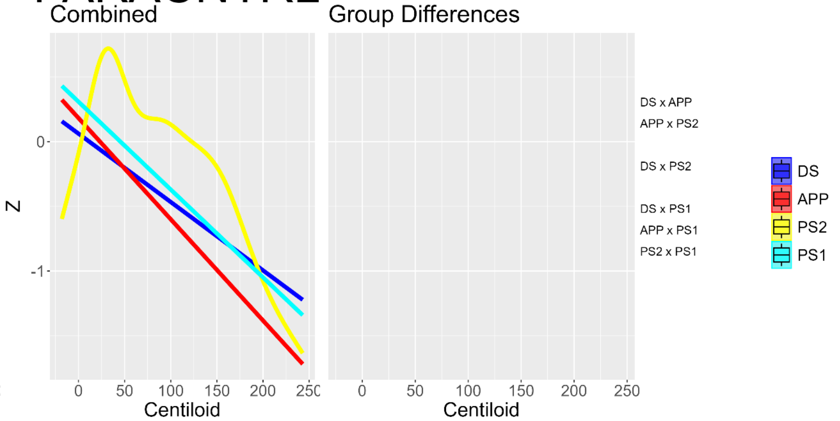
Combined
Group Differences
Z
0
-1
0
50
100
150
200
250
Centiloid
0
50
100
150
200
250
Centiloid
DS x APP
APP x PS2
DS x PS2
DS x PS1
APP x PS1
PS2 x PS1
DS
APP
PS2
PS1

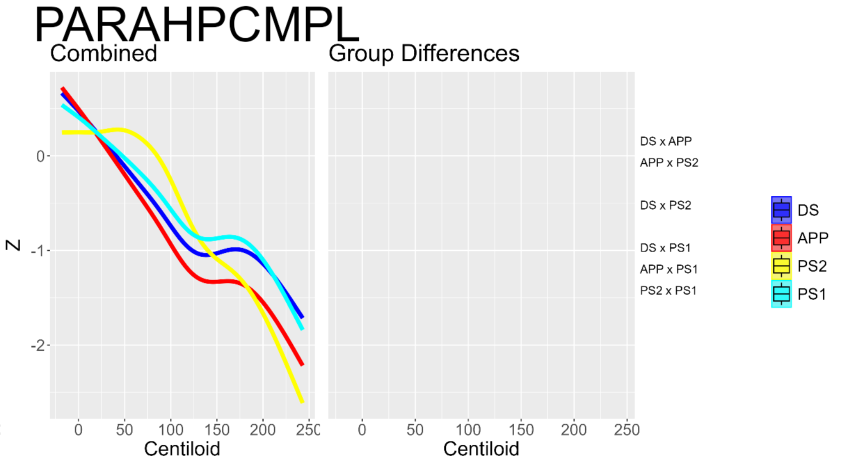
PARAHPCMPL
Combined
Group Differences
Z
0
-1
-2
0
50
100
150
200
250
Centiloid
0
50
100
150
200
250
Centiloid
DS x APP
APP x PS2
DS x PS2
DS x PS1
APP x PS1
PS2 x PS1
DS
APP
PS2
PS1

PARAOPRCLRS

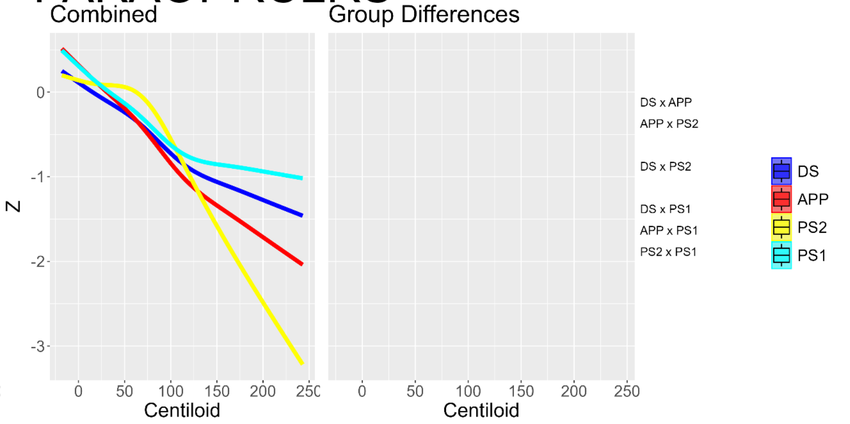

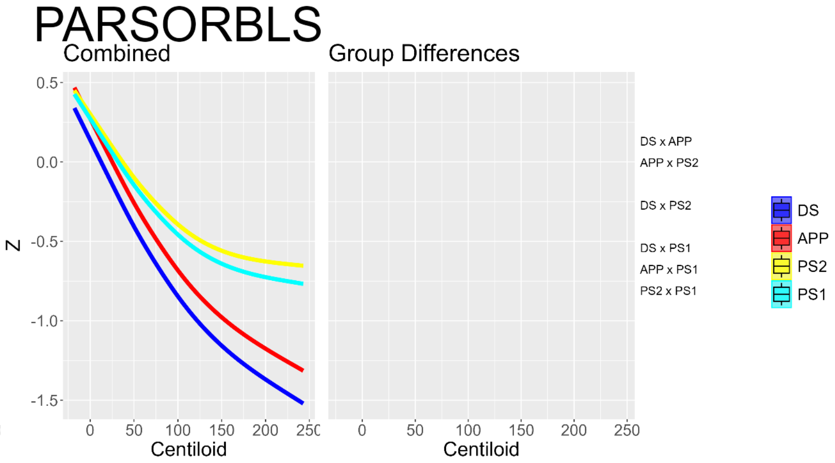
PARSORBLS
Combined
Group Differences
0.5
0.0
-0.5
-1.0
-1.5
Z
0
50
100
150
200
250
Centiloid
0
50
100
150
200
250
Centiloid
DS x APP
APP x PS2
DS x PS2
DS x PS1
APP x PS1
PS2 x PS1
DS
APP
PS2
PS1

PARSTRNGLRS

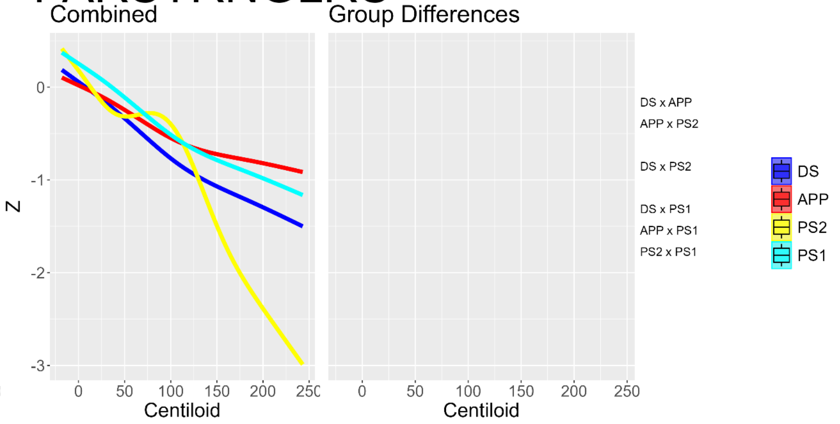

PERICLCRN

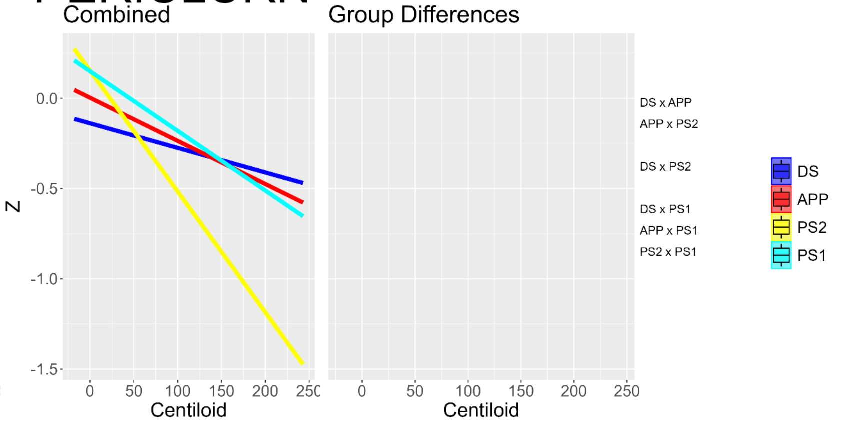

Combined
Group Differences
0.0
-0.5
-1.0
-1.5
Z
0
50
100
150
200
250
Centiloid
0
50
100
150
200
250
Centiloid
DS x APP
APP x PS2
DS x PS2
DS x PS1
APP x PS1
PS2 x PS1
DS
APP
PS2
PS1

POSTCNG

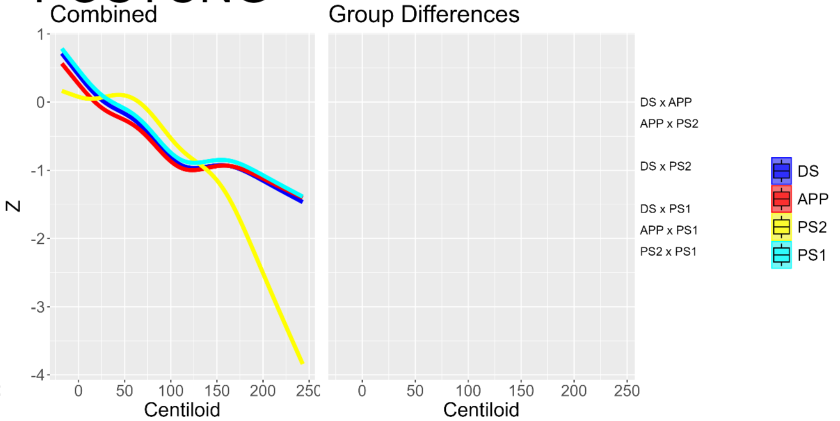

Combined
Group Differences
Z
Centiloid
Centiloid
1
0
-1
-2
-3
-4
0
50
100
150
200
250
DS x APP
APP x PS2
DS x PS2
DS x PS1
APP x PS1
PS2 x PS1
DS
APP
PS2
PS1

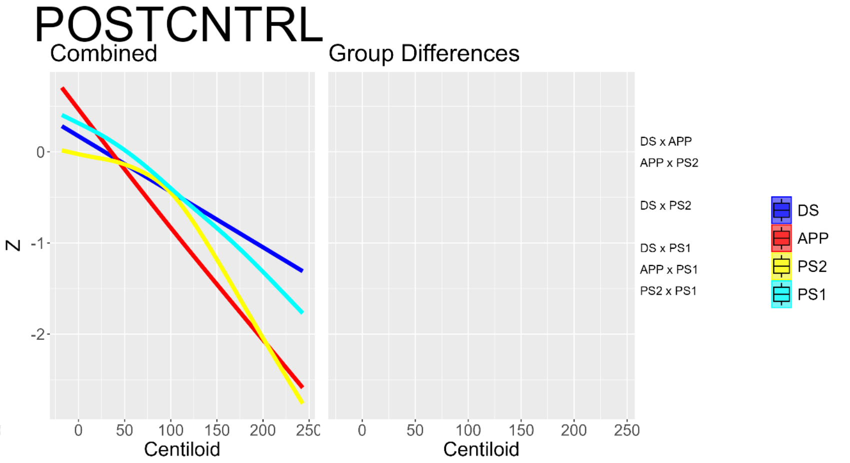
POSTCNTRL
Combined
Group Differences
0
-1
-2
Z
0
50
100
150
200
250
Centiloid
0
50
100
150
200
250
Centiloid
DS x APP
APP x PS2
DS x PS2
DS x PS1
APP x PS1
PS2 x PS1
DS
APP
PS2
PS1

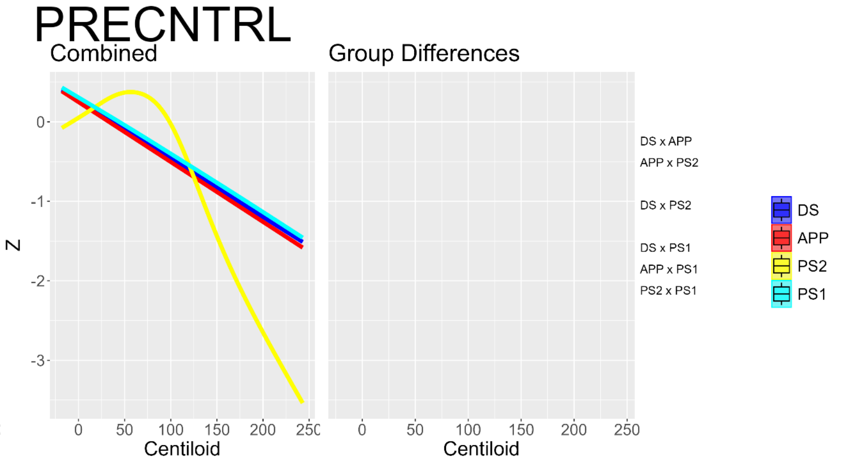
PRECNTRL
Combined
Group Differences
Z
0
-1
-2
-3
0
50
100
150
200
250
Centiloid
0
50
100
150
200
250
Centiloid
DS x APP
APP x PS2
DS x PS2
DS x PS1
APP x PS1
PS2 x PS1
DS
APP
PS2
PS1

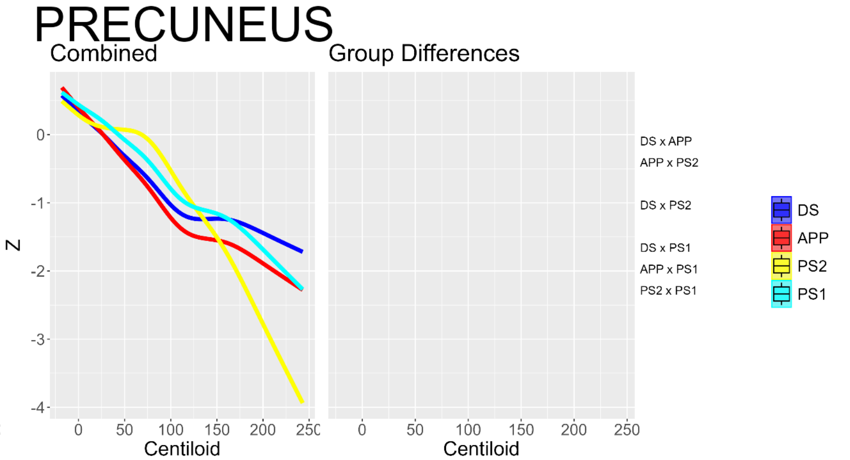

PRECUNEUS
Combined
Group Differences
Z
0
-1
-2
-3
-4
0
50
100
150
200
250
Centiloid
DS x APP
APP x PS2
DS x PS2
DS x PS1
APP x PS1
PS2 x PS1
DS
APP
PS2
PS1

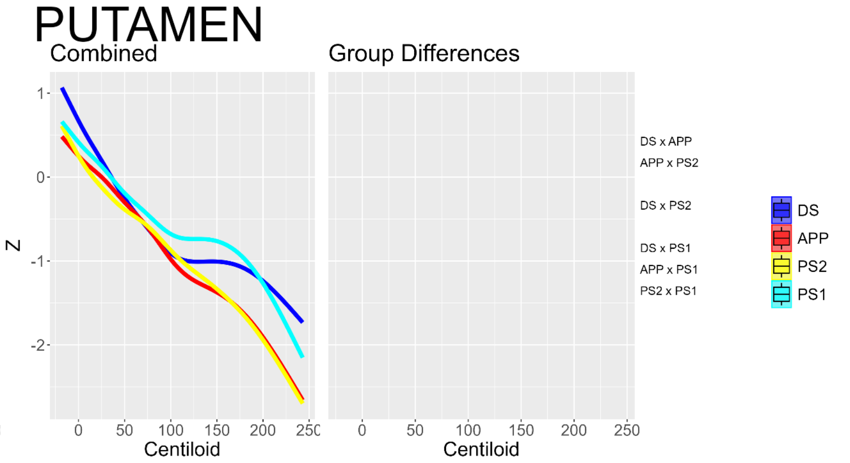
PUTAMEN
Combined
Group Differences
1
0
-1
-2
Z
0
50
100
150
200
250
Centiloid
0
50
100
150
200
250
Centiloid
DS x APP
APP x PS2
DS x PS2
DS x PS1
APP x PS1
PS2 x PS1
DS
APP
PS2
PS1

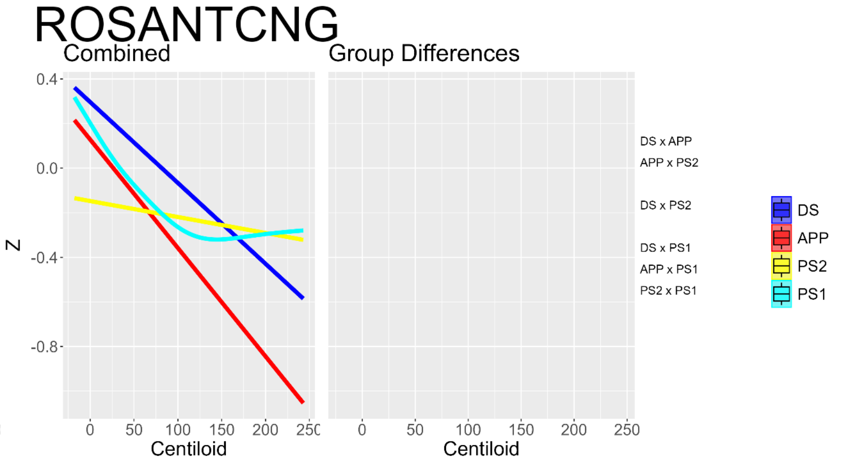
ROSANTCNG
Combined
Group Differences
0.4
0.0
-0.4
-0.8
Z
0
50
100
150
200
250
Centiloid
0
50
100
150
200
250
Centiloid
DS x APP
APP x PS2
DS x PS2
DS x PS1
APP x PS1
PS2 x PS1
DS
APP
PS2
PS1

ROSMIDFRN

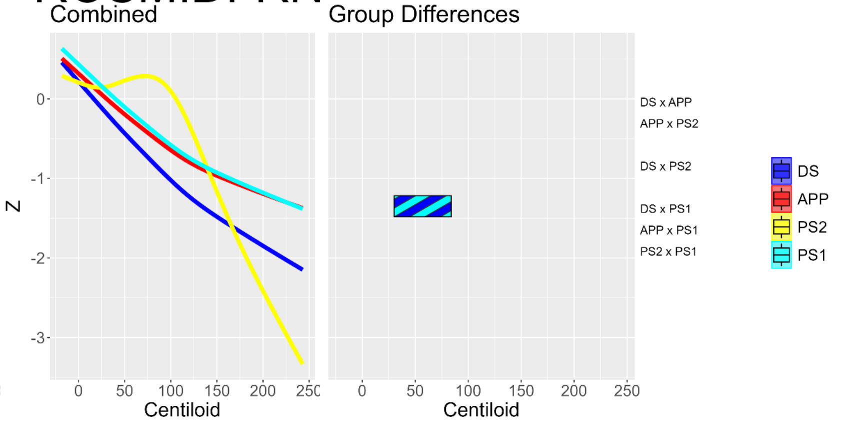

Combined
Group Differences
0
-1
-2
-3
Z
0
50
100
150
200
250
Centiloid
0
50
100
150
200
250
Centiloid
DS x APP
APP x PS2
DS x PS2
DS x PS1
APP x PS1
PS2 x PS1
DS
APP
PS2
PS1

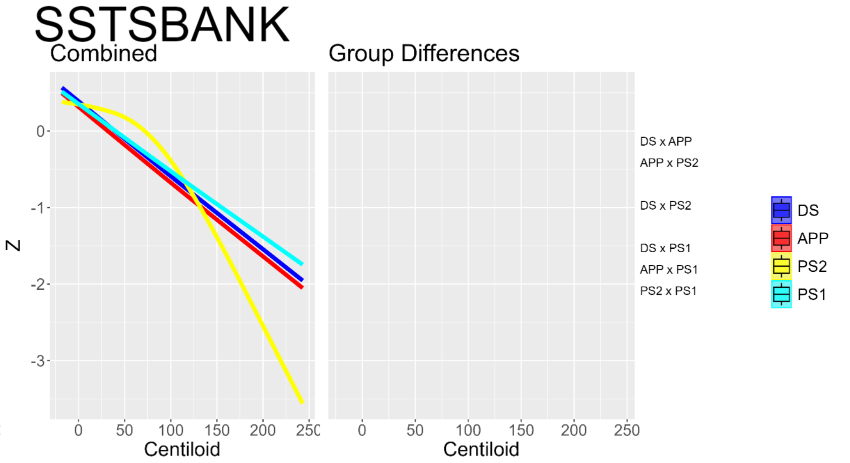
SSTSBANK
Combined
Group Differences
Z
0
-1
-2
-3
0
50
100
150
200
250
Centiloid
DS x APP
APP x PS2
DS x PS2
DS x PS1
APP x PS1
PS2 x PS1
DS
APP
PS2
PS1

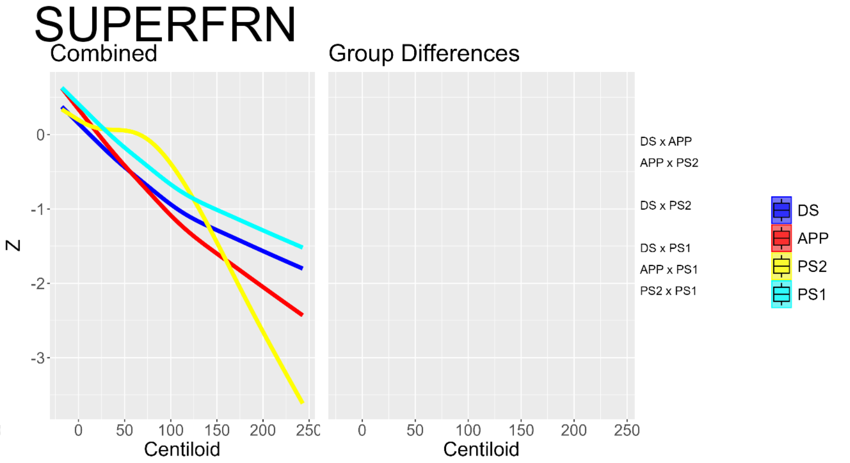

SUPERFRN
Combined
Group Differences
Z
0
-1
-2
-3
0
50
100
150
200
250
Centiloid
DS x APP
APP x PS2
DS x PS2
DS x PS1
APP x PS1
PS2 x PS1
DS
APP
PS2
PS1

SUPERPRTL

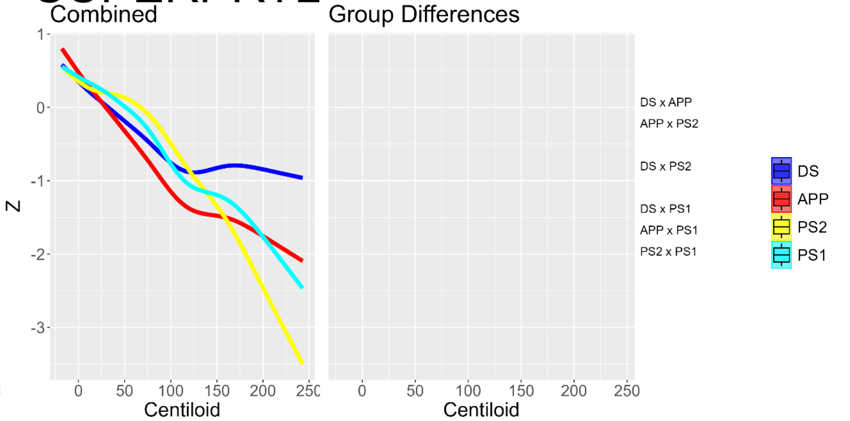
Combined
Group Differences
Z
1
0
-1
-2
-3
0
50
100
150
200
250
Centiloid
DS x APP
APP x PS2
DS x PS2
DS x PS1
APP x PS1
PS2 x PS1
DS
APP
PS2
PS1

SUPERTMP

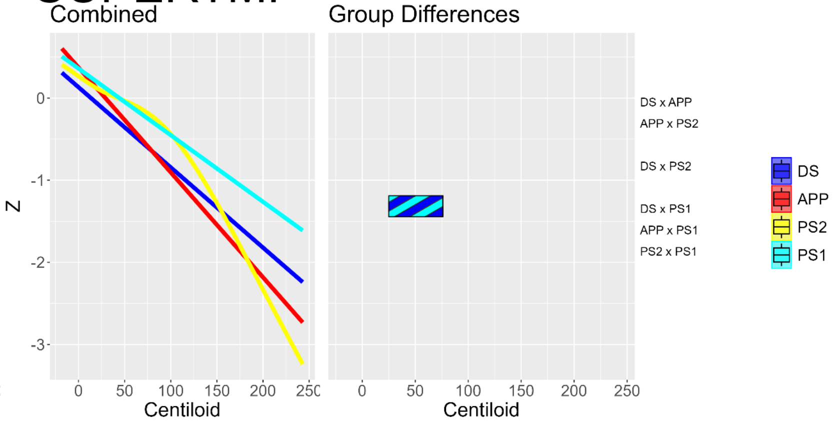

Combined
Group Differences
Z
0
-1
-2
-3
0
50
100
150
200
250
Centiloid
DS x APP
APP x PS2
DS x PS2
DS x PS1
APP x PS1
PS2 x PS1
DS
APP
PS2
PS1

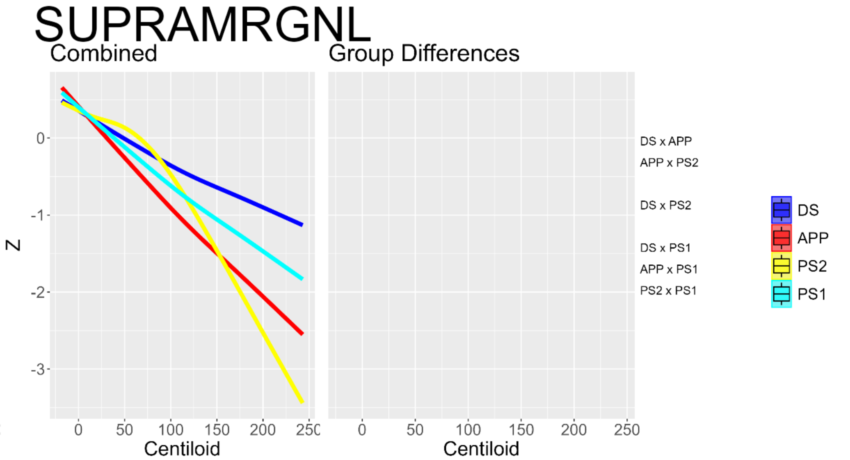

SUPRAMRGNL
Combined
Group Differences
Z
0
-1
-2
-3
0
50
100
150
200
250
Centiloid
DS x APP
APP x PS2
DS x PS2
DS x PS1
APP x PS1
PS2 x PS1
DS
APP
PS2
PS1

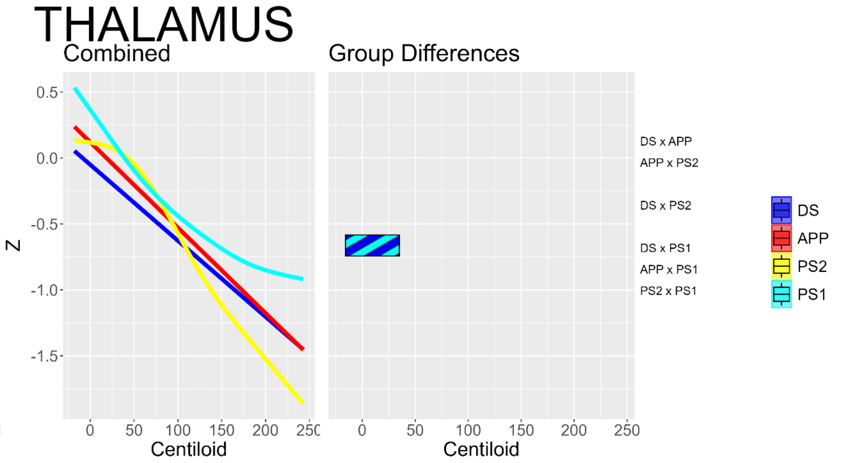

THALAMUS
Combined
Group Differences
0.5
0.0
-0.5
-1.0
-1.5
Z
0
50
100
150
200
250
Centiloid
Centiloid
DS x APP
APP x PS2
DS x PS2
DS x PS1
APP x PS1
PS2 x PS1
DS
APP
PS2
PS1

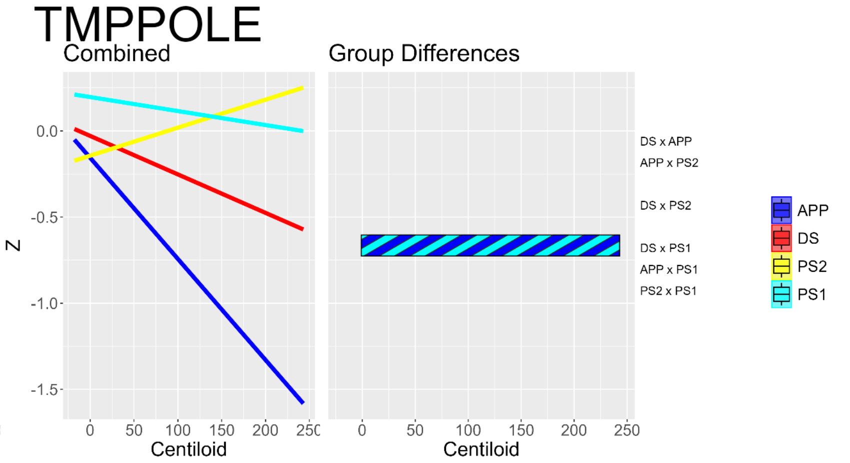
TMPPOLE
Combined
Group Differences
0.0
-0.5
-1.0
-1.5
Z
0
50
100
150
200
250
Centiloid
0
50
100
150
200
250
Centiloid
DS x APP
APP x PS2
DS x PS2
DS x PS1
APP x PS1
PS2 x PS1
APP
DS
PS2
PS1

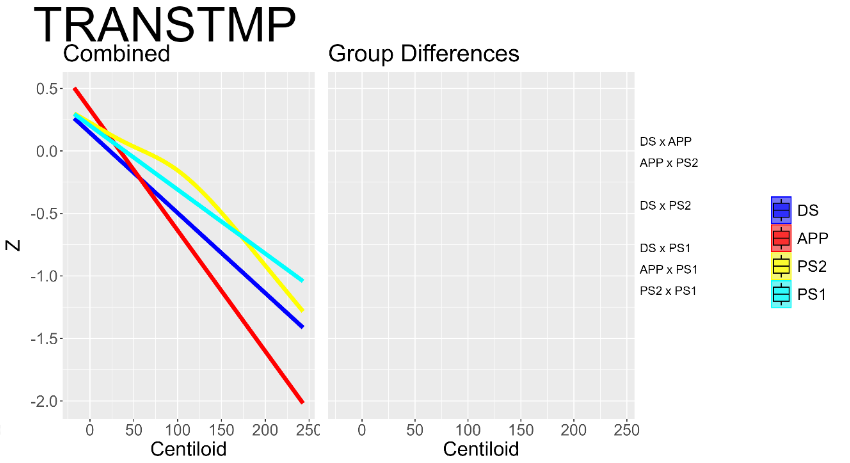
TRANSTMP
Combined
Group Differences
Z
0.5
0.0
-0.5
-1.0
-1.5
-2.0
0
50
100
150
200
250
Centiloid
0
50
100
150
200
250
Centiloid
DS x APP
APP x PS2
DS x PS2
DS x PS1
APP x PS1
PS2 x PS1
DS
APP
PS2
PS1

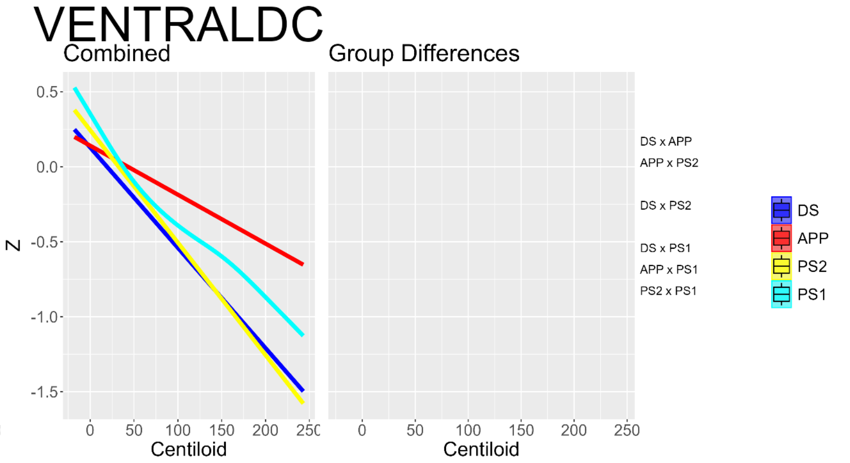
VENTRALDC
Combined
Group Differences
Z
0.5
0.0
-0.5
-1.0
-1.5
0
50
100
150
200
250
Centiloid
Centiloid
DS x APP
APP x PS2
DS x PS2
DS x PS1
APP x PS1
PS2 x PS1
DS
APP
PS2
PS1
